# International Society for Therapeutic Ultrasound Conference 2016: Tel Aviv, Israel. 14-18 March, 2016

**DOI:** 10.1186/s40349-016-0079-2

**Published:** 2017-03-17

**Authors:** Brian Fowlkes, Pejman Ghanouni, Narendra Sanghvi, Constantin Coussios, Paul C. Lyon, Michael Gray, Christophoros Mannaris, Marie de Saint Victor, Eleanor Stride, Robin Cleveland, Robert Carlisle, Feng Wu, Mark Middleton, Fergus Gleeson, Jean-Franҫois Aubry, Kim Butts Pauly, Chrit Moonen, Jacob Vortman, Pejman Ghanouni, Shirley Sharabi, Dianne Daniels, David Last, David Guez, Yoav Levy, Alexander Volovick, Javier Grinfeld, Itay Rachmilevich, Talia Amar, Zion Zibly, Yael Mardor, Sagi Harnof, Michael Plaksin, Yoni Weissler, Shy Shoham, Eitan Kimmel, Omer Naor, Nairouz Farah, Shy Shoham, Dong-Guk Paeng, Zhiyuan Xu, John Snell, Anders H. Quigg, Matthew Eames, Changzhu Jin, Ashli C. Everstine, Jason P. Sheehan, Beatriz S. Lopes, Neal Kassell, Thomas Looi, Vera Khokhlova, Charles Mougenot, Kullervo Hynynen, James Drake, Michael Slayton, Richard C. Amodei, Keegan Compton, Ashley McNelly, Daniel Latt, Michael Slayton, Richard C. Amodei, Keegan Compton, John Kearney, David Melodelima, Aurelien Dupre, Yao Chen, David Perol, Jeremy Vincenot, Jean-Yves Chapelon, Michel Rivoire, Wei Guo, Guoxin Ren, Guofeng Shen, Michael Neidrauer, Leonid Zubkov, Michael S. Weingarten, David J. Margolis, Peter A. Lewin, Nathan McDannold, Jonathan Sutton, Natalia Vykhodtseva, Margaret Livingstone, Thiele Kobus, Yong-Zhi Zhang, Natalia Vykhodtseva, Nathan McDannold, Michael Schwartz, Yuexi Huang, Nir Lipsman, Jennifer Jain, Martin Chapman, Tejas Sankar, Andres Lozano, Kullervo Hynynen, Michael Schwartz, Robert Yeung, Yuexi Huang, Nir Lipsman, Jennifer Jain, Martin Chapman, Andres Lozano, Kullervo Hynynen, Christakis Damianou, Nikolaos Papadopoulos, Alexander Volovick, Javier Grinfeld, Yoav Levy, Omer Brokman, Eyal Zadicario, Ori Brenner, David Castel, Shih-Ying Wu, Julien Grondin, Wenlan Zheng, Marc Heidmann, Maria Eleni Karakatsani, Carlos J. Sierra Sánchez, Vincent Ferrera, Elisa E. Konofagou, Christakis Damianou, Marinos Yiannakou, HongSeok Cho, Hwayoun Lee, Mun Han, Jong-Ryul Choi, Taekwan Lee, Sanghyun Ahn, Yongmin Chang, Juyoung Park, Nicholas Ellens, Ari Partanen, Keyvan Farahani, Raag Airan, Alexandre Carpentier, Michael Canney, Alexandre Vignot, Cyril Lafon, Jean-Yves Chapelon, Jean-yves Delattre, Ahmed Idbaih, Henrik Odéen, Bradley Bolster, Eun Kee Jeong, Dennis L. Parker, Pooja Gaur, Xue Feng, Samuel Fielden, Craig Meyer, Beat Werner, William Grissom, Michael Marx, Pejman Ghanouni, Kim Butts Pauly, Hans Weber, Valentina Taviani, Kim Butts Pauly, Pejman Ghanouni, Brian Hargreaves, Jun Tanaka, Kentaro Kikuchi, Ayumu Ishijima, Takashi Azuma, Kosuke Minamihata, Satoshi Yamaguchi, Teruyuki Nagamune, Ichiro Sakuma, Shu Takagi, Mathieu D. Santin, Laurent Marsac, Guillaume Maimbourg, Morgane Monfort, Benoit Larrat, Chantal François, Stéphane Lehéricy, Mickael Tanter, Jean-Franҫois Aubry, Maria Eleni Karakatsani, Gesthimani Samiotaki, Shutao Wang, Camilo Acosta, Eliza R. Feinberg, Elisa E. Konofagou, Zsofia I. Kovacs, Tsang-Wei Tu, Georgios Z. Papadakis, William C. Reid, Dima A. Hammoud, Joseph A. Frank, Zsofia i. Kovacs, Saejeong Kim, Neekita Jikaria, Michele Bresler, Farhan Qureshi, Joseph A. Frank, Jingjing Xia, Po-Shiang Tsui, Hao-Li Liu, Juan C. Plata, Samuel Fielden, Bragi Sveinsson, Brian Hargreaves, Craig Meyer, Kim Butts Pauly, Juan C. Plata, Vasant A. Salgaonkar, Matthew Adams, Chris Diederich, Eugene Ozhinsky, Matthew D. Bucknor, Viola Rieke, Ari Partanen, Andrew Mikhail, Lauren Severance, Ayele H. Negussie, Bradford Wood, Martijn de Greef, Gerald Schubert, Chrit Moonen, Mario Ries, Megan E. Poorman, Mary Dockery, Vandiver Chaplin, Stephanie O. Dudzinski, Ryan Spears, Charles Caskey, Todd Giorgio, William Grissom, Marcia M. Costa, Efthymia Papaevangelou, Anant Shah, Ian Rivens, Carol Box, Jeff Bamber, Gail ter Haar, Scott R. Burks, Matthew Nagle, Ben Nguyen, Michele Bresler, Joseph A. Frank, Scott R. Burks, Matthew Nagle, Ben Nguyen, Michele Bresler, Saejeong Kim, Blerta Milo, Joseph A. Frank, Nhan M. Le, Shaozhen Song, Kanheng Zhou, Ghulam Nabi, Zhihong Huang, Shmuel Ben-Ezra, Shani Rosen, Senay Mihcin, Jan Strehlow, Ioannis Karakitsios, Nhan Le, Michael Schwenke, Daniel Demedts, Paul Prentice, Sabrina Haase, Tobias Preusser, Andreas Melzer, Jean-Louis Mestas, Kamel Chettab, Gustavo Stadthagen Gomez, Charles Dumontet, Bettina Werle, Cyril Lafon, Fabrice Marquet, Pierre Bour, Fanny Vaillant, Sana Amraoui, Rémi Dubois, Philippe Ritter, Michel Haïssaguerre, Mélèze Hocini, Olivier Bernus, Bruno Quesson, Amit Livneh, Eitan Kimmel, Dan Adam, Justine Robin, Bastien Arnal, Mathias Fink, Mickael Tanter, Mathieu Pernot, Tatiana D. Khokhlova, George R. Schade, Yak-Nam Wang, Wayne Kreider, Julianna Simon, Frank Starr, Maria Karzova, Adam Maxwell, Michael R. Bailey, Vera Khokhlova, Jonathan E. Lundt, Steven P. Allen, Jonathan R. Sukovich, Timothy Hall, Zhen Xu, George R. Schade, Yak-Nam Wang, Tatiana D. Khokhlova, Philip May, Daniel W. Lin, Michael R. Bailey, Vera Khokhlova, Charlotte Constans, Thomas Deffieux, Mickael Tanter, Jean-Francois Aubry, Eun-Joo Park, Yun Deok Ahn, Soo Yeon Kang, Dong-Hyuk Park, Jae Young Lee, J. Vidal-Jove, E. Perich, A. Ruiz, A. Jaen, N. Eres, M. Alvarez del Castillo, Rachel Myers, James Kwan, Christian Coviello, Cliff Rowe, Calum Crake, Sean Finn, Edward Jackson, Robert Carlisle, Constantin Coussios, Antonios Pouliopoulos, Caiqin Li, Marc Tinguely, Meng-Xing Tang, Valeria Garbin, James J. Choi, Paul C. Lyon, Christophoros Mannaris, Michael Gray, Lisa Folkes, Michael Stratford, Robert Carlisle, Feng Wu, Mark Middleton, Fergus Gleeson, Constantin Coussios, Sandra Nwokeoha, Robert Carlisle, Robin Cleveland, Yak-Nam Wang, Tatiana D. Khokhlova, Tong Li, Navid Farr, Samantha D’Andrea, Frank Starr, Kayla Gravelle, Hong Chen, Ari Partanen, Donghoon Lee, Joo Ha Hwang, Sophie Tardoski, Jacqueline Ngo, Evelyne Gineyts, Jean-Pau Roux, Philippe Clézardin, David Melodelima, Allegra Conti, Rémi Magnin, Matthieu Gerstenmayer, François Lux, Olivier Tillement, Sébastien Mériaux, Stefania Della Penna, Gian Luca Romani, Erik Dumont, Benoit Larrat, Tao Sun, Chanikarn Power, Yong-Zhi Zhang, Jonathan Sutton, Eric Miller, Nathan McDannold, Oleg Sapozhnikov, Sergey Tsysar, Petr V. Yuldashev, Vera Khokhlova, Victor Svet, Wayne Kreider, Dongli Li, Antonio Pellegrino, Nik Petrinic, Clive Siviour, Antoine Jerusalem, Robin Cleveland, Peter V. Yuldashev, Maria Karzova, Bryan W. Cunitz, Barbrina Dunmire, Wayne Kreider, Oleg Sapozhnikov, Michael R. Bailey, Vera Khokhlova, Claude Inserra, Matthieu Guedra, Cyril Mauger, Bruno Gilles, Maxim Solovchuk, Tony W. H. Sheu, Marc Thiriet, Yufeng Zhou, Esra Neufeld, Christian Baumgartner, Davnah Payne, Adamos Kyriakou, Niels Kuster, Xu Xiao, Helen McLeod, Andreas Melzer, Christopher Dillon, Viola Rieke, Pejman Ghanouni, Dennis L. Parker, Allison Payne, Vera A. Khokhova, Peter V. Yuldashev, Ilya Sinilshchikov, Yulia Andriyakhina, Tatiana D. Khokhlova, Wayne Kreider, Adam Maxwell, Oleg Sapozhnikov, Ari Partanen, Andrey Rybyanets, Natalia Shvetsova, Alex Berkovich, Igor Shvetsov, Oleg Sapozhnikov, Vera Khokhlova, Caroline J. Shaw, Ian Rivens, John Civale, Dino Giussani, Gail ter Haar, Christoph Lees, Pierre Bour, Fabrice Marquet, Valery Ozenne, Solenn Toupin, Bruno Quesson, Erik Dumont, Eugene Ozhinsky, Vasant Salgaonkar, Chris Diederich, Viola Rieke, Elena Kaye, Sebastien Monette, Majid Maybody, Govindarajan Srimathveeravalli, Stephen Solomon, Amitabh Gulati, Tobias Preusser, Sabrina Haase, Mario Bezzi, Jürgen W. Jenne, Thomas Lango, Yoav Levy, Michael Müller, Giora Sat, Christine Tanner, Stephan Zangos, Matthias Günther, Andreas Melzer, Cyril Lafon, Au Hoang Dinh, Emilie Niaf, Flavie Bratan, Nicolas Guillen, Rémi Souchon, Carole Lartizien, Sebastien Crouzet, Olivier Rouviere, Jean-Yves Chapelon, Yang Han, Shutao Wang, Elisa E. Konofagou, Thomas Payen, Carmine Palermo, Steve Sastra, Hong Chen, Yang Han, Kenneth Olive, Elisa E. Konofagou, Johanna M. van Breugel, Martijn de Greef, Charles Mougenot, Maurice A. van den Bosch, Chrit Moonen, Mario Ries, Matthieu Gerstenmayer, Rémi Magnin, Benjamin Fellah, Denis Le Bihan, Benoit Larrat, Matthieu Gerstenmayer, Rémi Magnin, Sébastien Mériaux, Denis Le Bihan, Benoit Larrat, Steven P. Allen, Luis Hernandez-Garcia, Charles A. Cain, Timothy Hall, Erasmia Lyka, Delphine Elbes, Christian Coviello, Robin Cleveland, Constantin Coussios, Kanheng Zhou, Nhan M. Le, Chunhui Li, Zhihong Huang, Satoshi Tamano, Hayato Jimbo, Takashi Azuma, Shin Yoshizawa, Keisuke Fujiwara, Kazunori Itani, Shin-ichiro Umemura, Christakis Damianou, Marinos Yiannakou, Nicholas Ellens, Ari Partanen, Dan Stoianovici, Keyvan Farahani, Zulfadhli Zaini, Ryo Takagi, Shin Yoshizawa, Shin-ichiro Umemura, Shenyan Zong, Guofeng Shen, Ron Watkins, Aurea Pascal-Tenorio, Matthew Adams, Juan C. Plata, Vasant Salgaonkar, Peter Jones, Kim Butts-Pauly, Chris Diederich, Donna Bouley, Andrey Rybyanets, Guoxin Ren, Wei Guo, Guofeng Shen, Yazhu Chen, Chung-Yin Lin, Han-Yi Hsieh, Kuo-Chen Wei, Hao-Li Liu, Camille Garnier, Gilles Renault, Navid Farr, Ari Partanen, Ayele H. Negussie, Andrew Mikhail, Reza Seifabadi, Emmanuel Wilson, Avinash Eranki, Peter Kim, Bradford Wood, Dennis Lübke, Jürgen W. Jenne, Peter Huber, Matthias Günther, Dennis Lübke, Joachim Georgii, Michael Schwenke, Caroline V. Dresky, Julian Haller, Matthias Günther, Tobias Preusser, Jürgen W. Jenne, Avinash Eranki, Navid Farr, Ari Partanen, Pavel Yarmolenko, Ayele H. Negussie, Karun Sharma, Haydar Celik, Bradford Wood, Peter Kim, Guofeng Li, Weibao Qiu, Hairong Zheng, Meng-Yen Tsai, Po-Chun Chu, Hao-Li Liu, Taylor Webb, Urvi Vyas, Kim Butts Pauly, Matthew Walker, Jidan Zhong, Thomas Looi, Adam C. Waspe, James Drake, Mojgan Hodaie, Feng-Yi Yang, Sin-Luo Huang, Yuval Zur, Alexander Volovick, Benny Assif, Christian Aurup, Hermes Kamimura, Shutao Wang, Hong Chen, Camilo Acosta, Antonio A. Carneiro, Elisa E. Konofagou, Alexander Volovick, Javier Grinfeld, David Castel, Sven Rothlübbers, Julia Schwaab, Christine Tanner, Senay Mihcin, Graeme Houston, Matthias Günther, Jürgen W. Jenne, Eugene Ozhinsky, Matthew D. Bucknor, Viola Rieke, Haim Azhari, Noam Weiss, Jacob Sosna, S. Nahum Goldberg, Victor Barrere, David Melodelima, Kee W. Jang, Scott R. Burks, Zsofia I. Kovacs, Tsang-Wei Tu, Bobbi Lewis, Saejeong Kim, Matthew Nagle, Neekita Jikaria, Joseph A. Frank, Yufeng Zhou, Xiaotong Wang, Yun Deok Ahn, Eun-Joo Park, Dong-Hyuk Park, Soo Yeon Kang, Jae Young Lee, Visa Suomi, Elisa E. Konofagou, David Edwards, Robin Cleveland, Zahary Larrabee, Matthew Eames, Arik Hananel, Jean-Franҫois Aubry, Boaz Rafaely, Alexander Volovick, Javier Grinfeld, Eitan Kimmel, Rasha Elaimy Debbiny, Carmel Zeltser Dekel, Michael Assa, Eitan Kimmel, George Menikou, Christakis Damianou, Petros Mouratidis, Ian Rivens, Gail ter Haar, José A. Pineda-Pardo, Marta Del Álamo de Pedro, Raul Martinez, Frida Hernandez, Silvia Casas, Carlos Oliver, Patricia Pastor, Lidia Vela, Jose Obeso, Paul Greillier, Ali Zorgani, Rémi Souchon, David Melodelima, Stefan Catheline, Cyril Lafon, Vyacheslav Solovov, Michael O. Vozdvizhenskiy, Andrew E. Orlov, Chueh-Hung Wu, Ming-Kuan Sun, Tiffany T. Shih, Wen-Shiang Chen, Fabrice Prieur, Arnaud Pillon, Jean-Louis Mestas, Valerie Cartron, Patrick Cebe, Nathalie Chansard, Maxime Lafond, Cyril Lafon, Claude Inserra, Pauline Muleki Seya, Wen-Shiang Chen, Jean-Christophe Bera, Tanguy Boissenot, Benoit Larrat, Elias Fattal, Alexandre Bordat, Helene Chacun, Claire Guetin, Nicolas Tsapis, Kazuo Maruyama, Johan Unga, Ryo Suzuki, Cécile Fant, Maxime Lafond, Bernadette Rogez, Jacqueline Ngo, Cyril Lafon, Jean-Louis Mestas, Mercy Afadzi, Ola Finneng Myhre, Siri Vea, Astrid Bjørkøy, Petros Tesfamichael Yemane, Annemieke van Wamel, Sigrid Berg, Rune Hansen, Bjørn Angelsen, Catharina Davies

**Affiliations:** 1 Basic Radiological Sciences Division, Department of Radiology, University of Michigan, Ann Arbor, Michigan USA; 2 Stanford School of Medicine, Stanford, California USA; 3 SonaCare Medical, Indianapolis, Indiana USA; 4 Institute of Biomedical Engineering, University of Oxford, Oxford, UK; 5 Department of Radiology, Churchill Hospital, Oxford, UK; 6 Department of Oncology, University of Oxford, Oxford, UK; 7 HIFU Unit, Churchill Hospital, Oxford, UK; 8 Institut Langevin, Paris, France; 9 Stanford School of Medicine, Stanford, California USA; 10 Center for Imaging Sciences, Imaging Division, University Medical Center, Utrecht, The Netherlands; 11 InSighTec, Haifa, Israel; 12 Stanford School of Medicine, Stanford, California USA; 13 Sheba Medical Center, Ramat-Gan, Israel; 14 Tel-Aviv University, Tel-Aviv, Israel; 15 InSighTec, Haifa, Israel; 16 Faculty of Biomedical Engineering & Russell Berrie Nanotechnology Institute, Technion – Israel Institute of Technology, Haifa, Israel; 17 ELSC Center for Brain Sciences, Hebrew University, Jerusalem, Israel; 18 Biomedical Engineering, Technion- Israel Institute of Technology, Haifa, Israel; 19 Faculty of Life Sciences, Bar Ilan University, Ramat Gan, Israel; 20 Focused Ultrasound Foundation, Charlottesville, Virginia USA; 21 Neurosurgery, University of Virginia, Charlottesville, Virginia USA; 22 Ocean System Engineering, Jeju National University, Jeju, Republic of Korea; 23 Biology, University of Virginia, Charlottesville, Virginia USA; 24 Pathology, University of Virginia, Charlottesville, Virginia USA; 25 Hospital for Sick Children, Toronto, Ontario Canada; 26 University of Washington, Seattle, Washington USA; 27 Sunnybrook Research Institute, Toronto, Ontario Canada; 28 University of Toronto, Toronto, Ontario Canada; 29 Philips Healthcare, Markham, Ontario Canada; 30 Guided Therapy Systems, LLC, Mesa, Arizona USA; 31 School of Medicine, University of Arizona, Tucson, Arizona USA; 32 Guided Therapy Systems, Mesa, Arizona USA; 33 The CORE Institute, Phoenix, Arizona USA; 34 LabTAU - U1032, INSERM, Lyon, France; 35 Centre Leon Berard, Lyon, France; 36 Department of Oral Maxillofacial and Head Neck Oncology, Shanghai 9th People’s Hospital, Shanghai, China; 37 The School of Biomedical Engineering, Science and Health Systems, Drexel University, Philadelphia, Pennsylvania USA; 38 Department of Surgery, College of Medicine, Drexel University, Philadelphia, Pennsylvania USA; 39 Biostatistics and Epidemiology, University of Pennsylvania, Philadelphia, Pennsylvania USA; 40 Radiology, Brigham and Women’s Hospital, Boston, Massachusetts USA; 41 Neurobiology, Harvard Medical School, Boston, Massachusetts USA; 42 Radiology and Nuclear Medicine, Radboud University Medical Center, Nijmegen, Netherlands; 43 Radiology, Brigham and Women’s Hospital, Boston, Massachusetts USA; 44 Surgery (Neurosurgery), University of Toronto, Toronto, Ontario Canada; 45 Sunnybrook Health Sciences Centre, Toronto, Ontario Canada; 46 Surgery (Neurosurgery), University of Alberta, Edmonton, Alberta Canada; 47 Sunnybrook Health Sciences Centre, Toronto, Ontario Canada; 48 Surgery (Neurosurgery), University of Toronto, Toronto, Ontario Canada; 49 Cyprus University of Technology, Limassol, Cyprus; 50 City University, London, UK; 51 INSIGHTEC, Tirat Carmel, Israel; 52 Weizmann Institute, Rehovot, Israel; 53 Sheba Medical Center, Ramat Gan, Israel; 54 Biomedical Engineering, Columbia University, New York, New York USA; 55 Radiology, Columbia University, New York, New York USA; 56 Neuroscience, Columbia University, New York, New York USA; 57 Cyprus University of Technology, Limassol, Cyprus; 58 Daegu-Gyeongbuk Medical Innovation Foundation, Daegu, Republic of Korea; 59 Kyungpook National University, Daegu, Republic of Korea; 60 Radiology, Johns Hopkins University, Baltimore, Maryland USA; 61 Philips, Andover, Massachusetts, USA; 62 National Cancer Institute, Bethesda, Maryland USA; 63 CarThera, Lyon, France; 64 INSERM, U1032, LabTau, Lyon, France; 65 Assistance Publique Hopitaux de Paris, Hopital de la Pitie Salpetriere, Department of Neurosurgery, Paris, France; 66 Universite Paris, UPMC, Paris, France; 67 Assistance Publique Hopitaux de Paris, Hopital de la Pitie Salpetriere, Department of Neuro-Oncology, Paris, France; 68 Siemens Healthcare, Salt Lake City, Utah USA; 69 Radiology, University of Utah, Salt Lake City, Utah USA; 70 Vanderbilt University, Nashville, Tennessee USA; 71 University of Virginia, Charlottesville, Virginia USA; 72 University Children’s Hospital, Zurich, Switzerland; 73 Radiology, Stanford University, Stanford, California USA; 74 Radiology, Stanford University, Stanford, California USA; 75 The University of Tokyo, Tokyo, Japan; 76 Centre de NeuroImagerie de Recherche - CENIR, Paris, France; 77 Inserm U 1127, CNRS UMR 7225, Sorbonne Universités, UPMC Univ Paris 06 UMR S 1127, Institut du Cerveau et de la Moelle épinière, ICM, Paris, France; 78 Supersonic Imagine, Aix-en-Provence, France; 79 Institut Langevin, CNRS, Paris, France; 80 Institut Langevin, Université Denis Diderot, Paris, France; 81 Institut Langevin, INSERM, Paris, France; 82 Biomedical Engineering, Columbia University, New York, New York USA; 83 Biological Sciences, Columbia University, New York, New York USA; 84 Radiology, Columbia University, New York, New York USA; 85 Frank Laboratory, Radiology and Imaging Sciences, National Institute of Health, Bethesda, Maryland USA; 86 Center for Infectious Disease Imaging (CIDI), Radiology and Imaging Sciences, National Institute of Health, Bethesda, Maryland USA; 87 National Institute of Biomedical Imaging and Bioengineering, National Institute of Health, Bethesda, Maryland USA; 88 Frank Laboratory, Radiology and Imaging Sciences, National Institute of Health, Bethesda, Maryland USA; 89 National Institute of Biomedical Imaging and Bioengineering, National Institute of Health, Bethesda, Maryland USA; 90 Department of Medical Imaging and Radiological Sciences, Chang Gung University, Taoyuan, Taiwan; 91 Department of Electrical Engineering, Chang Gung University, Taoyuan, Taiwan; 92 Radiology, Stanford University, Stanford, California USA; 93 Bioengineering, University of Virginia, Charlottesville, Virginia USA; 94 Radiology, Stanford University, Stanford, California USA; 95 Radiation Oncology, University of California San Francisco, San Francisco, California USA; 96 Radiology and Biomedical Imaging, University of California San Francisco, San Francisco, California USA; 97 Clinical Science MR Therapy, Philips, Andover, Massachusetts USA; 98 Center for Interventional Oncology, Department of Radiology and Imaging Sciences, Clinical Center, National Institutes of Health, Bethesda, Maryland USA; 99 Philips Healthcare, Vantaa, Finland; 100 Imaging Division, University Medical Center, Utrecht, Netherlands; 101 Biomedical Engineering, Vanderbilt University, Nashville, Tennessee USA; 102 Institute of Imaging Science, Vanderbilt University, Nashville, Tennessee USA; 103 Chemical and Physical Biology, Vanderbilt University, Nashville, Tennessee USA; 104 Radiology, Vanderbilt University, Nashville, Tennessee USA; 105 The Institute of Cancer Research, Department of Physics, Sutton, UK; 106 Radiology and Imaging Sciences, NIH Clinical Center, Bethesda, Maryland USA; 107 Radiology and Imaging Sciences, NIH Clinical Center, Bethesda, Maryland USA; 108 Mechanical Engineering, University of Dundee, Dundee, Angus UK; 109 University of Dundee, Dundee, UK; 110 University of Washington, Seattle, Washington USA; 111 Action-Physics, Pardes-Hanna, Israel; 112 CByond ltd., Nesher, Israel; 113 School of Medicine, IMSaT, Dundee, UK; 114 Fraunhofer, Mevis, Bremen Germany; 115 U1032, INSERM, Lyon, Rhone-Alpes France; 116 Bioaster, Lyon, Rhone-Alpes France; 117 U1052, INSERM, Lyon, Rhone-Alpes France; 118 Université de lyon, Lyon, Rhone-Alpes France; 119 IHU Institut de Rythmologie et de Modélisation Cardiaque, Bordeaux, France; 120 INSERM U1045 CRCTB, Université de Bordeaux, Bordeaux, France; 121 CHU de Bordeaux, Bordeaux, France; 122 Department of Biomedical Engineering, Technion-Israel Institute of Technology, Haifa, Israel; 123 Institut Langevin, ESPCI ParisTech, CNRS UMR 7587, INSERM U979, Université Paris Diderot, Paris, France; 124 Institut Langevin, ESPCI ParisTech, CNRS UMR 7587, INSERM U979, Université Paris Diderot, Paris, France; 125 Department of Urology, University of Washington, Seattle, Washington USA; 126 Applied Physics Laboratory, University of Washington, Seattle, Washington USA; 127 Physics Department, M.V. Lomonosov Moscow State University, Moscow, Russian Federation; 128 Department of Medicine, University of Washington, Seattle, Washington USA; 129 Biomedical Engineering, University of Michigan, Ann Arbor, Michigan USA; 130 Medicine, University of Washington, Seattle, Washington USA; 131 Urology, University of Washington, Seattle, Washington USA; 132 Appied Physics Lab, University of Washington, Seattle, Washington USA; 133 Physics, M.V. Lomonosov Moscow State University, Moscow, Russian Federation; 134 Institut Langevin, INSERM, Paris, France; 135 Institut Langevin, Université Paris Diderot, Paris, France; 136 Institut Langevin, CNRS, Paris, France; 137 Radiology, Seoul National University Hospital, Seoul, Republic of Korea; 138 Surgical Oncology, HIFU Unit, Hospital Universitari Mutua Terrassa (HUMT), Barcelona, Spain; 139 Research Unit, Hospital Universitari Mutua Terrassa (HUMT), Barcelona, Spain; 140 Medical Direction Department, Hospital Universitari Mutua Terrassa (HUMT), Barcelona, Spain; 141 Interventional Oncology Unit, Institut Khuab, Barcelona, Spain; 142 HIFU Onco & Radiology Department, Clínica Santa Elena, Madrid, Spain; 143 OxSonics Ltd, Oxford, UK; 144 Institute of Biomedical Engineering, University of Oxford, Oxford, UK; 145 Bioengineering, Imperial College London, London, UK; 146 Chemical Engineering, Imperial College London, London, UK; 147 University of Oxford & Oxford University Hospitals NHS Foundation Trust, Oxford, UK; 148 Institute of Biomedical Engineering, University of Oxford, Oxford, UK; 149 University of Washington, Seattle, Washington USA; 150 Philips, Bethesda, Maryland USA; 151 LabTAU - U1032, INSERM, Lyon, France; 152 Lyos - U1033, INSERM, Lyon, France; 153 CEA/DSV/I2BM/NeuroSpin, Gif sur Yvette, France; 154 Department of Neuroscience, G. D’Annunzio University, Chieti, Italy; 155 Image Guided Therapy, Pessac, France; 156 Université Lyon 1, Lyon, France; 157 Department of Radiology, Brigham and Women’s Hospital, Harvard Medical School, Boston, Massachusetts USA; 158 Department of Electrical and Computer Engineering, Tufts University, Medford, Massachusetts USA; 159 Physics Faculty, Moscow State University, Moscow, Russian Federation; 160 Center for Industrial and Medical Ultrasound, Applied Physics Laboratory, University of Washington, Seattle, WA USA; 161 N.N. Andreyev Acoustics Institute, Moscow, Russian Federation; 162 Department of Engineering Science, University of Oxford, Oxford, UK; 163 Physics Faculty, Moscow State University, Moscow, Russian Federation; 164 CIMU, Applied Physics Laboratory, University of Washington, Seattle, WA USA; 165 LabTAU Inserm U1032, Lyon, France; 166 INSA de Lyon, Villeurbanne, France; 167 Institutes of Biomedical Engineering and Nanomedicine, National Health Research Institutes, Zhunan, Taiwan; 168 National Taiwan University, Taipei, Taiwan; 169 Sorbonne Universities, UPMC Univ Paris 06, Paris, France; 170 Nanyang Technological University, Singapore, Singapore; 171 IT’IS Foundation for Research on Information Technologies in Society, Zurich, Switzerland; 172 Swiss Federal Institute of Technology (ETHZ), Zurich, Switzerland; 173 Institute for Medical Science and Technology, Dundee, UK; 174 Ninewells Hospital, Dundee, UK; 175 Radiology, University of Utah, Salt Lake City, UT USA; 176 Radiology and Biomedical Imaging, University of California San Francisco, San Francisco, CA USA; 177 Radiology, Stanford University, Stanford, CA USA; 178 Physics Faculty, M.V. Lomonosov Moscow State University, Moscow, Russian Federation; 179 Center for Industrial and Medical Ultrasound, University of Washington, Seattle, WA USA; 180 Clinical Science MR Therapy, Philips, Andover, MA USA; 181 Department of Gastroenterology, School of Medicine, University of Washington, Seattle, WA USA; 182 Department of Urology, School of Medicine, University of Washington, Seattle, WA USA; 183 Southern Federal University, Rostov on Don, Russian Federation; 184 Saint Petersburg Polytechnical University, Saint Petersburg, Russian Federation; 185 Moscow State University, Moscow, Russian Federation; 186 Department of Physiology, Development and Neuroscience, University of Cambridge, Cambridge, UK; 187 Joint Department of Physics, The Institute of Cancer Research, London, UK; 188 Institute of Developmental and Reproductive Biology, Imperial College London, UK, London, UK; 189 IHU-Liryc, Pessac, France; 190 Image Guided Therapy, Pessac, France; 191 Radiology and Biomedical Imaging, University of California San Francisco, San Francisco, CA USA; 192 Radiation Oncology, University of California San Francisco, San Francisco, CA USA; 193 Memorial Sloan Kettering Cancer Center, New York, NY USA; 194 Fraunhofer MEVIS, Bremen, Germany; 195 Jacobs University, Bremen, Germany; 196 Universita Degli Studi Di Roma La Sapienza, Rome, Italy; 197 Stiftelsen SINTEF, Trondheim, Norway; 198 InSightec, Haifa, Israel; 199 IBSmm, Brno, Czech Republic; 200 ETH, Zurich, Switzerland; 201 Johann Wolfgang Goethe University, Frankfurt, Germany; 202 IMSat, University of Dundee, Dundee, UK; 203 Mediri GmbH, Heidelberg, Germany; 204 GE Medical Systems, Haifa, Israel; 205 LabTAU, INSERM, Lyon, France; 206 University of Lyon, Lyon, France; 207 CREATIS, Lyon, France; 208 EDAP-TMS, Vaux-en-Velin, France; 209 Hospices Civils de Lyon, Lyon, France; 210 Department of Biomedical Engineering, Columbia University, New York, NY USA; 211 Department of Radiology, Columbia University, New York, NY USA; 212 Biomedical Engineering, Columbia University, New York, NY USA; 213 Herbert Irving Comprehensive, Cancer Center, Columbia University, New York, NY USA; 214 Radiology, Columbia University, New York, NY USA; 215 Radiology, University Medical Center Utrecht, Utrecht, Netherlands; 216 Imaging Department, University Medical Center Utrecht, Utrecht, Netherlands; 217 Philips, Ontario, Ontario, Canada; 218 NeuroSpin, CEA Saclay, Gif-sur-Yvette, France; 219 NeuroSpin, CEA Saclay, Gif sur Yvette, France; 220 Biomedical Engineering, University of Michigan, Ann Arbor, MI USA; 221 FMRI Lab, University of Michigan, Ann Arbor, MI USA; 222 Engineering Science, University of Oxford, Oxford, UK; 223 University of Dundee, Dundee, UK; 224 Graduate School of Engineering, Tohoku University, Sendai, Miyagi Japan; 225 Hitachi Aloka Medical, Ltd., Kokubunji, Tokyo Japan; 226 Department of Mechanical Engineering, The University of Tokyo, Bunkyo, Tokyo Japan; 227 Graduate School of Biomedical Engineering, Tohoku University, Sendai, Miyagi Japan; 228 Cyprus University of Technology, Limassol, Cyprus; 229 Radiology, Johns Hopkins University, Baltimore, MD USA; 230 Philips, Andover, MA USA; 231 Urology, Johns Hopkins University, Baltimore, MD USA; 232 National Cancer Institute, Bethesda, MD USA; 233 Biomedical Engineering, Tohoku University, Sendai, Japan; 234 Communication Engineering, Tohoku University, Sendai, Japan; 235 School of Biomedical Engineering, Shanghai Jiao Tong University, Biomedical Instrument Institute, Shanghai, China; 236 Shanghai Jiao Tong University, Med-X Research Institute, Shanghai, China; 237 Radiology, Stanford University, Stanford, CA USA; 238 Comparative Medicine, Stanford University, Palo Alto, CA USA; 239 Thermal Therapy Research Group, UCSF, San Francisco, CA USA; 240 Radiology, Stanford University, Palo Alto, CA USA; 241 Thermal Therapy Research Group, UCSF, San Francisco, CA USA; 242 Thermal Therapy Research Group, UCSF, San Francisco, CA USA; 243 Radiology, Stanford University, Palo Alto, CA USA; 244 Thermal Therapy Research Group, UCSF, San Francisco, CA USA; 245 Comparative Medicine, Stanford University, Palo Alto, CA USA; 246 Radiology, Stanford University, Palo Alto, CA USA; 247 Bioengineering, UC Berkeley-UCSF Joint, San Francisco, CA USA; 248 Southern Federal University, Rostov on Don, Russian Federation; 249 Shangai Jiao Tong University, Shanghai, China; 250 Chang Gung University, Taoyuan, Taiwan; 251 Université Paris 7, Paris, France; 252 Institut Cochin, Paris, France; 253 Institut Curie, Paris, France; 254 National Institutes of Health, Bethesda, MD, USA; 255 Clinical Science MR Therapy, Philips, Andover, MA USA; 256 Children’s National Medical Center, Washington, DC USA; 257 MRI Physics, Fraunhofer MEVIS, Bremen, Germany; 258 Deutsches Krebsforschungszentrum, Heidelberg, Germany; 259 Fraunhofer MEVIS, Bremen, Germany; 260 PTB Braunschweig, Braunschweig, Germany; 261 Children’s National Medical Center, Washington, DC USA; 262 National Institutes of Health, Bethesda, MD USA; 263 Clinical Science MR Therapy, Philips, Andover, MA USA; 264 Shenzhen Institutes of Advanced Technology, Chinese Academy of Sciences, Shenzhen, China; 265 Department of Electrical Engineering, Chang Gung University, Taoyuan, Taiwan; 266 Electrical Engineering, Stanford University, Palo Alto, CA USA; 267 Radiology, Stanford University, Palo Alto, CA USA; 268 Institute of Medical Science, University of Toronto, Toronto, Ontario Canada; 269 Krembil Research Institute, University Health Network, Toronto, Ontario Canada; 270 Centre for Image Guided Innovation and Therapeutic Intervention, Hospital for Sick Children, Toronto, Ontario Canada; 271 Department of Biomedical Imaging and Radiological Sciences, National Yang-Ming University, Taipei, Taiwan; 272 General Electric, Tirat Carmel, Israel; 273 InSighTec, Tirat Carmel, Israel; 274 Columbia University, New York, NY USA; 275 Universidade de São Paulo, São Paulo, Brasil; 276 InSighTec, Tirat Carmel, Israel; 277 Sheba Medical Center, Ramat Gan, Israel; 278 mediri GmbH, Heidelberg, Germany; 279 Fraunhofer MEVIS, Bremen, Germany; 280 ETH Zürich, Zürich, Switzerland; 281 IMSAT, Dundee, UK; 282 Radiology and Biomedical Imaging, University of California San Francisco, San Francisco, CA USA; 283 Biomedical Engineering, Technion, IIT, Haifa, Israel; 284 Department of Radiology, Hadassah, Hebrew University, Medical Center, Jerusalem, Israel; 285 Radiology, Beth Israel Deaconess Medical Center, Boston, MA USA; 286 Inserm, Lyon, France; 287 Université de Lyon, Lyon, France; 288 Frank Laboratory, Radiology and Imaging Sciences, National Institutes of Health, Bethesda, MD USA; 289 Nanyang Technological University, Singapore, Singapore; 290 Radiology, Seoul National University Hospital, Seoul, Republic of Korea; 291 University of Oxford, Oxford, UK; 292 Columbia University, New York City, NY USA; 293 Focused Ultrasound Foundation, Charlottesville, VA USA; 294 Radiation Oncology, University of Virginia, Charlottesville, VA USA; 295 Institut Langevin, Paris, France; 296 CNRS, Paris, France; 297 Department of Electrical and Computer Engineering, Ben-Gurion University of the Negev, Beer-Sheva, Israel; 298 InSighTec, Tirat Carmel, Israel; 299 Biomedical Engineering, Technion – Israel Institute of Technology, Haifa, Israel; 300 Biomedical Engineering, Technion, Haifa, Israel; 301 City University, London, UK; 302 Cyprus University of Technology, Limassol, Cyprus; 303 Radiotherapy and Imaging, The Institute of Cancer Research, Sutton, London, Surrey UK; 304 HM CINAC. Hospital Puerta del Sur, Móstoles, Spain; 305 Hospital Puerta del Sur, Móstoles, Spain; 306 LabTau - U1032, INSERM, Lyon, France; 307 Samara Oncology Center, Samara, Russian Federation; 308 Physical Medicine & Rehabilitation, National Taiwan University Hospital, Taipei, Taiwan; 309 Medical Imaging, National Taiwan University Hospital, Taipei, Taiwan; 310 Biomedical Engineering and Nanomedicine, National Health Research Institute, Zhunan, Taiwan; 311 University of Oslo, Oslo, Norway; 312 U1032, LabTau, INSERM, Lyon, France; 313 Centre de Recherche en Oncologie Experimentale, Institut de Recherche Pierre Fabre, Toulouse, France; 314 Caviskills SAS, Vaulx-en-Velin, France; 315 LabTAU Inserm U1032, Lyon, France; 316 Laboratoire de Mécanique et d’Acoustique CNRS - UPR 7051, Marseille, France; 317 National Taiwan University Hospital, Taipei, Taiwan; 318 Institut Galien Paris-Sud, CNRS, Univ. Paris-Sud, Université Paris Sacla, Châtenay-Malabry, France; 319 CEA, Neurospin, Saclay, France; 320 Department of DDS Research, Teikyo University, Tokyo, Japan; 321 LabTAU, INSERM U1032, Lyon, France; 322 Physics, Norwegian Univeristy of Science and Techonlogy, Trondhiem, Norway; 323 Circulation and Medical Imaging, Norwegian Univeristy of Science and Techonlogy, Trondhiem, Norway; 324 SINTEF Technology and Society, Trondhiem, Norway

## ORAL PRESENTATIONS

### O1 The role of bubbles and cavitation in therapy ultrasound

#### Brian Fowlkes

##### Basic Radiological Sciences Division, Department of Radiology, University of Michigan, Ann Arbor, Michigan, USA

When exposed to sufficiently high ultrasound pressures, microbubbles can be generated spontaneously in tissue and undergo inertial cavitation where collapses result in physical effects. These effects range from petechial haemorrhage to complete cellular disruption, termed Histotripsy, depending on ultrasound parameters. This presentation will explore the mechanisms associated with histotripsy along with the tissue effects and the wide range of potential applications for this mechanical disruption method.

### O2 Challenges for clinical trials in therapeutic ultrasound, the need for an evidence base, & trial design

#### Pejman Ghanouni

##### Stanford School of Medicine, Stanford, California, USA

In this lecture, we will compare the design of clinical trials that led to approval of MR guided focused ultrasound for the treatment of uterine fibroids and osseous metastases. The impact of these trials on the evidence base, and thus on adoption by users and coverage by insurers will be compared. We will also review the process of expanding approved FUS applications, either via investigator- or industry-initiated studies or through off-label clinical use.

### O3 Prostate HIFU – current status

#### Narendra Sanghvi

##### SonaCare Medical, Indianapolis, Indiana, USA

In this lecture, present status of focused ultrasound for the treatment of localized prostate cancer ablation will be discussed. High intensity focused ultrasound (HIFU) has been used for the ablation of prostate over two decades and has treated thousands of prostate cancer patients. Meanwhile, prostate cancer management is undergoing significant improvements as molecular markers, targeted biopsy and advanced multi-parametric MRI are routinely used to accurately localize the prostate cancer. These advances offer a unique opportunity for focal ablation of localized prostate cancer with HIFU as it plays a significant role in reducing morbidity and treatment cost. This presentation will focus on hardware design, software architecture and HIFU features of the devices. Presentation will demonstrate localization of prostate with ultrasound imaging, treatment planning with 3D volumetric rendering of the prostate with ultrasound and MRI fusion techniques for focal treatment and finally HIFU dose setting with guidance using real time Tissue Change Monitoring (TCM) with the Sonablate device. The presenter will encourage exchange of ideas and discussion for research topics.

### O4 Enhancement of drug delivery - clinical challenges and solutions

#### Constantin Coussios^1^, Paul C. Lyon^1^, Michael Gray^1^, Christophoros Mannaris^1^, Marie de Saint Victor^1^, Eleanor Stride^1^, Robin Cleveland^1^, Robert Carlisle^1^, Feng Wu^4^, Mark Middleton^3^, Fergus Gleeson^2^

##### ^1^Institute of Biomedical Engineering, University of Oxford, Oxford, United Kingdom; ^2^Department of Radiology, Churchill Hospital, Oxford, United Kingdom; ^3^Department of Oncology, University of Oxford, Oxford, United Kingdom; ^4^HIFU Unit, Churchill Hospital, Oxford, UK

There are four key clinical challenges in optimizing drug based strategies: (i) achieving prolonged blood circulation of the therapeutic to enable active or passive accumulation in the target tissue; (ii) mediating triggered release or activation, or active accumulation of the therapeutic, to maximize its concentration at the target site whilst reducing off target side effects; (iii) enabling successful transport of the therapeutic from the blood stream into the target tissue, and achieving a homogenous distribution in that target tissue and (iv) where necessary, further enabling penetration of the therapeutic into the cell. The potential of therapeutic ultrasound with, or without, sonosensitive microparticle or nanoparticle formulations to address these challenges will be explored.

### O5 Neuromodulation with ultrasound for beginners

#### Jean-Franҫois Aubry

##### Institut Langevin, Paris, France

In this lecture, the use of transcranial low intensity focused ultrasound for neuromodulation will be discussed. A historical review will be presented, with an emphasis on the experimental setups and the acoustical parameters. Models ranging from slice cultures to intact rodents and primates will be presented, together with recent trials on humans. Potential mechanisms will be described. Based on our own experience, exciting successful neuromodulation as well as disappointing failures will be presented.

### O6 Thermometry in ultrasound fields, challenges & solutions *in vivo*, *ex vivo* & everywhere else!

#### Kim Butts Pauly

##### Stanford School of Medicine, Stanford, California, USA

This talk will cover image-based thermometry methods for guiding focused ultrasound. MR Thermometry based on the proton resonance frequency shift with temperature is linear and reversible in aqueous tissues and is utilized in clinical practice with common temperature resolutions of 1°C. Ultrasound based thermometry based on the speed of sound change with temperature can be used to 45°C in aqueous tissue. Both methods are sensitive to the presence of fat within the aqueous tissue, as well as motion. This talk will cover these basic concepts as well as their use in clinical practice.

### O7 Motion compensation

#### Chrit Moonen

##### Center for Imaging Sciences, Imaging Division, University Medical Center, Utrecht, the Netherlands

Motion leads to several challenges for HIFU treatment. In this lecture, the effects of respiratory, cardiac, and peristalsis related motion on MRI thermometry will be discussed. In addition, methods for tracking the moving target with the HIFU will be described, as well as gating strategies.

### O8 MRgFUS crossing the chasm from proof of concept to mainstream treatment alternative

#### Jacob Vortman

##### InSighTec, Haifa, Israel

MRgFUS treatment is a disruptive, non-invasive, outpatient treatment alternative that is capable to treat tumours and functional disorders under real-time monitoring and control via MR thermometry.

Transforming this breakthrough technology from the lab to a mainstream treatment alternative requires gaining the support and the agreement to change by a whole array of stakeholders in different areas some interrelated and some conflicting.

The stakeholders’ current position, changes they will need to go through and possible changes engines are mentioned below:

▪ Physicians (surgeons) need to transform into image guided surgeons where knowledge and understanding of the disease play a dominant role in the procedure outcome. The benefit is the confidence in the safety and efficacy coupled with income that wouldn’t decline. The change engine could be the patients and the payers.

▪ Payers should benefit from covering MRgFUS by saving cost and addressing patients’ demands. The change engine in this case should be the patients, governments and physicians.

▪ Governments should see the benefit of very fast recovery, very low level of adverse events and productivity enhancement. In this case patients and physicians should drive the change.

▪ Patients should benefit from safer treatment, fast recovery next day back to your life, minimal trauma and morbidity. The significant benefit to them should transform them to the dominant driver of this change. They will need to influence physicians, payers and providers to adopt this new treatment.

▪ Providers should adopt the technology and provide this treatment since the data exist proving safety and efficacy, proven cost savings and physicians and patients demand.

The current Medical ecosystem is biased against new technologies since the incumbent system/treatments are reimbursed while the new technologies are not. Could governments perform economic analysis and if found beneficial (example: saving money and improving productivity) decide on limited 2 years reimbursement during which RCT data will be collected based on which private insurance will decide to cover. This model should incentivize the physician, payers and providers to try the new technology.

**Fig. 1 (abstract O8).**
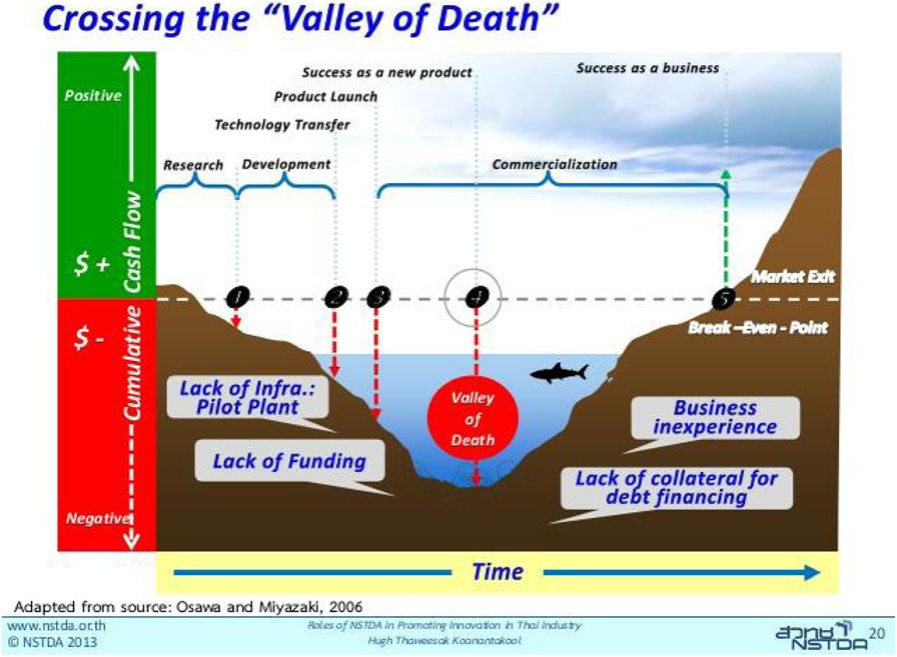
Overcoming the resistant to change - is there a strategy that could bring all the different stakeholders to combine and align efforts?

### O9 MR guided focused ultrasound treatment of soft tissue tumours of the extremities

#### Pejman Ghanouni

##### Stanford School of Medicine, Stanford, California, USA

In this lecture, the use of MR guided focused ultrasound for the treatment of soft tissue and osseous tumours will be discussed. Results of treatment of these different types of tumours will be reviewed, including lessons learned from challenging patient treatments. Technical aspects of all parts of a treatment, including patient preparation, positioning, imaging, planning, thermometry, and methods of evaluation, will be described. The talk will also focus on methods developed to address these current challenges and opportunities for future development.

### O10 Non-invasive, non-destructive FUS-induced neuro-modulation assessed by recording auditory evoked potentials – initial experience in small/large animals

#### Shirley Sharabi^1,2^, Dianne Daniels^1,2^, David Last^1^, David Guez^1^, Yoav Levy^3^, Alexander Volovick^3^, Javier Grinfeld^3^, Itay Rachmilevich^3^, Talia Amar^3^, Zion Zibly^1^, Yael Mardor^1,2^, Sagi Harnof^1,2^

##### ^1^Sheba Medical Center, Ramat-Gan, Israel; ^2^Tel-Aviv University, Tel-Aviv, Israel, ^3^InSighTec, Haifa, Israel

**Objectives**

MR guided focused ultrasound (MRgFUS) has been extensively studied in recent years as a non-invasive treatment modality. Initial clinical trials have indicated promising treatment response to ablative FUS treatments of patients with brain tumours, neuropathic pain, essential tremor, obsessive compulsive disorder, and Parkinson’s disease. Apart from the ablative applications of FUS, this technology has been extensively evaluated for less destructive applications such as thrombolysis, blood–brain barrier disruption for increased drug delivery, and recently also neuro-modulation.

The objective of the presented study was to demonstrate non-invasive, non-destructive, reversible FUS-induced neuro-modulation in small (rats) and large (pigs) animals by inducing temporary suppression of auditory evoked potentials.

**Methods**

**Fig. 2 (abstract O10).**
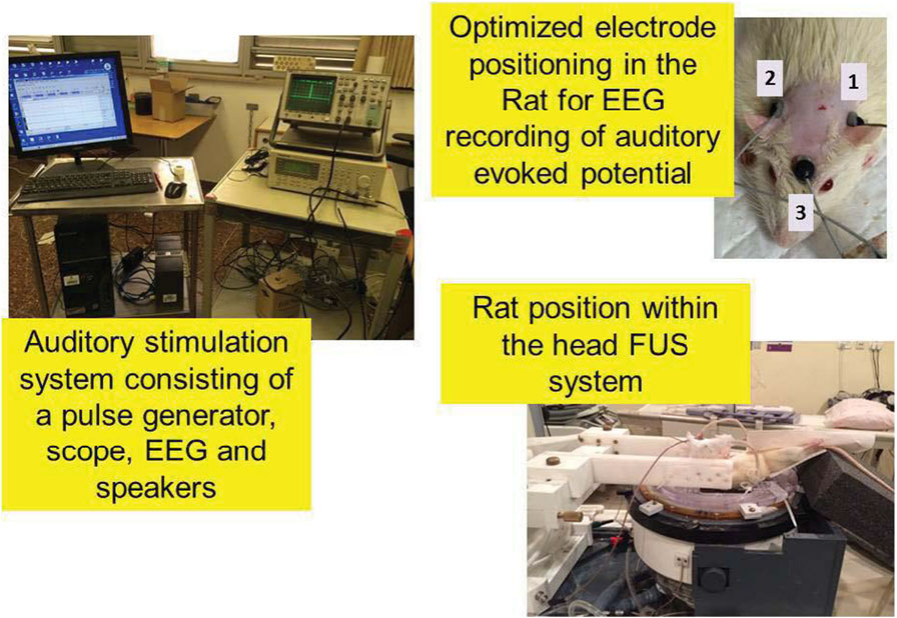
See text for description

All animal experiments were performed under full anaesthesia. Rats were anesthetized by Xylazine/Ktalar and pigs were anesthetized by Propofol. Rat’s heads were shaved prior to treatment but they did not undergo craniotomy. Pigs underwent craniotomy to avoid FUS reflection/aberration by the skull. EEG was recorded using 3 small cup-shaped electrodes attached to the skin/dura with metal particles-containing gel for optimal sound conductivity (Fig. 2).

The audio stimulation system consisted of a pulse generator connected to the EEG trigger input and to speakers placed near the animal ears, producing a square-wave form at 10 KHz, resulting in 150 “click” sounds per min (Fig. 2). Each measurement consisted of 200 repetitions enabling acquisition of a full measurement in 1’20” min.

The ExAblate Neuro system (InSightec, Tel Aviv, Israel) is a combination of a standard MRI scanner and a FUS delivery system. The FUS device is in the shape of a helmet consisting of 1024 transducers which deliver US energy in the form of “sonications”. The system is designed to provide real-time therapy, planning, thermal dosimetry, and closed loop therapy control. Treatment starts with conventional MRI scans, displayed on the ExAblate computer, used to determine regions of interest of the target volume. During the procedure, the beam path is periodically reviewed to confirm the planned direction through the tissue. The set of sonication volumes is sequentially applied to cover the entire planned volume. The current experiments were performed with a modified ExAblate version developed for neuro-modulation as part of the MAGNET programs supported by the Israeli Ministry of Commerce.

Baseline auditory evoked potentials were recorded by EEG prior to FUS treatment, with the animals in the prone position. The animals were then placed in the supine position, with the skull dipped in degassed water at the centre of the FUS system, for localization MRI scanning followed by FUS treatment. The animals were then returned to the prone position for continuous post-treatment EEG recordings. Rats which did not show recovery of the auditory evoked potentials 30–60 min post treatment were monitored again 48 hours or 1 week post treatment.

The animals were treated by FUS for 52 sec using the Exablate Neuro system at 220 KHz, 12 W, and 100 ms on/ 2900 ms off pulses. Two rats were treated in the thalamus region (targeted at deep auditory tracks) and another two in the frontal cortex region (targeted at peripheral auditory tracks). Two sham rats underwent a similar procedure without activation of the FUS system. One pig was treated in the thalamus region and another in the right motor cortex region.

**Results**

Auditory evoked potential EEG signals shapes varied from one animal to the other but all were detected 2–10 ms after the trigger. The maximal peak-to-peak height was calculated for each measurement.

The sham rats showed no significant change in the auditory evoked potentials EEG signal.

The rats treated in the thalamus regions showed 50% and 65% suppression of the baseline auditory evoked potentials EEG signal. The first showed no recovery 2 hours post treatment with full recovery measured 1 week post treatment. The second showed no recovery for 1 hour post treatment and full recovery 48 hours post treatment. The rats treated in the cortex regions showed 50% and 67% suppression of the baseline EEG signal. The first showed no recovery 30 min post treatment with full recovery measured 1 week post treatment. The second showed initial recovery 14 min post treatment reaching full recovery within 28 min post treatment.

**Fig. 3 (abstract O10).**
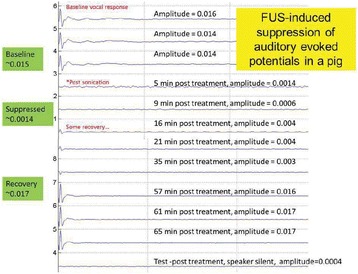
See text for description

The pig treated in the thalamus region showed 90% suppression of the baseline signal with no recovery 30 min post treatment. The second pig, treated in the cortex region, showed complete suppression of the baseline signal immediately post treatment with initial recovery noted 18 min post treatment, reaching full recovery 63 min post treatment (Fig. 3).

**Conclusions**

Our preliminary results suggest that reversible neuro-modulation by non-invasive FUS is feasible. Full recovery was noted in all 4 treated rats and in 1 of the 2 treated pigs. Unfortunately we were not able to monitor the first pig for more than 30 min post treatment.

### O11 Biophysical dissection of ultrasonic neuromodulation mechanisms

#### Michael Plaksin, Yoni Weissler, Shy Shoham, Eitan Kimmel

##### Faculty of Biomedical Engineering & Russell Berrie Nanotechnology Institute, Technion – Israel Institute of Technology, Haifa, Israel

**Objectives**

Low intensity US can noninvasively suppress or excite central nervous system (CNS) activity using different combinations of stimulation parameters. While applications are already emerging, the underlying biophysics remains unclear regarding the relative contribution of possible mechanisms: extracellular bubble cavitation, thermal effects, acoustic radiation pressure and US-induced intramembrane cavitation within the bilayer membrane (the bilayer sonophore or BLS model). Interestingly, both radiation pressure and intramembrane cavitation can induce plasma membrane capacitance changes. Here, we use detailed predictive modelling and find that only intramembrane cavitation can explain all the observed aspects of ultrasonic neuromodulation.

**Methods**

We analyzed the relevant experimental literature using modified Rayleigh–Plesset intramembrane cavitation BLS biomechanics and acoustic radiation pressure gradients (RPG) - induced membrane dynamics. By coupling these biomechanical models to biophysical membrane models we predict dynamical biophysical responses of artificial bilayer membranes, and of three common neocortical single cell Hodgkin-Huxley type models: i) Regular Spiking (RS) cortical pyramidal neuron, ii) Fast Spiking (FS) cortical inhibitory neuron and iii) Low Threshold Spiking (LTS) cortical inhibitory neuron, RS-FS-LTS Hodgkin-Huxley based network model and CNS axon model. In addition, live brain tissue RPG subjected areal strains were evaluated in a viscoelastic brain model.

**Results**

Only the Neuronal Intramembrane Cavitation Excitation (NICE) models were able to explain US-induced action potential generation through BLS-type pulsating nano-bubbles inside the bilayer plasma membrane: the leaflets' periodic vibrations induce US-frequency membrane capacitance and potential oscillations, leading to slow charge accumulation across the membrane (on a time scale of tens of milliseconds), until action potentials are generated. In contrast, the analysis of RPG-induced membrane capacitance variations associated with membrane area changes explain artificial membrane results, but were found to be highly unlikely sources for neural excitation, when considering the areal strains expected to form in brain tissue during normal sonication. Further, the NICE-LTS inhibitory neurons show a much higher relative sensitivity to sparse ultrasonic stimulation compared to the other neurons, resulting from their T-type voltage gated calcium channels. This model-based prediction was found to explain the results of a significant body of suppression and excitation experimental studies, including in humans.

**Conclusions**

These results provide a unified theoretical framework for a large body of experiments in multiple preparations across the field of US neuromodulation, lending further support to the hypothesis that intramembrane cavitation is responsible for ultrasonic neuromodulation. They could thus pave the way towards new CNS therapeutic protocols, using the only method that currently allows targeted non-invasive neuromodulation with millimetre spatial resolution essentially anywhere in the brain.

### O12 Ultrasonic stimulation of mammalian retina *in-vitro*

#### Omer Naor^1,2^, Nairouz Farah^3^, Shy Shoham^2^

##### ^1^ELSC Center for Brain Sciences, Hebrew University, Jerusalem, Israel; ^2^Biomedical Engineering, Technion- Israel Institute of Technology, Haifa, Israel; ^3^Faculty of Life Sciences, Bar Ilan University, Ramat Gan, Israel

**Objectives**

Following previous in vivo stimulation of the retina, we aimed to achieve a first direct measurement of the response of mammalian retinal neurons to ultrasonic (US) stimuli, and to study and characterize this response.

**Methods**

We coupled a high-density phased array (986 elements on a 25x35 mm^2^ area) to a system for multi-electrode-array (MEA) recording with 256 contacts. Mouse retinas were dissected and placed on the MEA, and sonicated at 2.3 MHz, applying varying durations and intensities, as well as stimulated by light. The acquired data were processed to detect action potentials (spikes) elicited by retinal ganglion cells, and analysed to reveal the relations between the stimuli and the responses.

**Results**

We found prominent spike responses for stimuli in the range of 4.3-7.3 W/cm^2^ and 0.5-1 s, which disappeared when the focus was steered 1.5 mm away. Furthermore, we found that the relation between the response strength and the stimulation intensity, or duration, followed a logistic sigmoid curve, while the response latency was described by a decreasing exponent. Lastly, we found indications that the observed responses to US stimuli are related to the "2^nd^ OFF" component in the responses to light stimuli.

**Conclusions**

These findings are the first direct demonstration of the response of the mammalian retina to US stimulation. The properties of the US transducer and the stimulation frequency indicate that non-invasive US stimulation of human retina is feasible, and may potentially evolve as an important tool for diagnosis and treatment of retinal diseases.

### O13 Motor response elicitation and pupil dilation using megahertz-range focused ultrasound neuromodulation

#### Christian Aurup^1^, Hermes Kamimura^2,1^, Shutao Wang^1^, Hong Chen^1^, Camilo Acosta^1^, Antonio A. Carneiro^2^, Elisa E. Konofagou^1^

##### ^1^Columbia University, New York, New York, USA; ^2^Universidade de São Paulo, São Paulo, Brasil

**Objectives**

Using transcranial focused ultrasound for the modulation of brain activity has been identified as a possible non-invasive means of treating neurological disorders. Most studies involving sedate rodents use frequencies in the kilohertz range, which allow for optimal transmission of acoustic power through the skull. The trade-off of using lower frequencies involves a lack of target specificity. Higher frequencies must be used in order to modulate activity in a more highly-specified manner. This study demonstrates that focused ultrasound in the megahertz range can be used to evoke motor- and cognitive-related responses in mice under deep anaesthesia by targeting specific brain structures. Contralateral-paired hind limb movements were observed when stimulating cortical regions, demonstrating the ability of MHz-range FUS to stimulate activity in highly-localized brain regions. Additionally, pupil dilation was observed when deep-seated anxiety-related structures were targeted, demonstrating the ability of FUS to modulate cognitive activity in a highly-specified manner.

**Methods**

For this study, wild-type adult male mice were anesthetized with intraperitoneal injections of sodium pentobarbital (65 mg/kg) and fixed in a stereotaxic frame. A single-element FUS transducer with fundamental frequency of 1.94 MHz was fixed to a 3D positioning system for accurate navigation through the brain. A 6x6 mm grid centred +2 mm rostral of the lambda skull suture was sonicated in a random order using a centre frequency of 1.9 MHz, pulse repetition frequency of 1 kHz, 50% duty cycle, 1 second pulse duration, 1 second inter-pulse interval for a total of 10 pulse repetitions. The acoustic pressure applied was varied in order to evaluate thresholds for eliciting physiological responses like motor movement, eye movement, or pupil dilation. Motor movements were validated using video recordings and electromyography via needle electrodes implanted into the biceps femoris of both hind limbs. Videos were recorded using a high-resolution camera focused at the right eye and processed to measure eye movements or changes in pupil size.

**Results**

The minimum acoustic pressure required to elicit motor movements was 1.45 MPa when targeting the somatosensory cortex, calibrated using an excised mouse skull. Higher pressures increased the success rate from 20% (at the 1.45 MPa threshold) to 70% (1.79 MPa). Targeting eye-motor and anxiety related regions of the brain elicited eye movements and pupil dilations up to 20%. Sonicating the superior colliculus resulted in both eye movement and pupil dilation at a lower threshold pressure (1.20 MPa) than the hippocampus and locus coeruleus which required pressures greater than 1.80 MPa.

**Conclusions**

This study successfully demonstrated that MHz-range transcranial focused ultrasound can be used to elicit motor- and cognitive-related physiological responses with high specificity in mice in vivo. It was also shown that the success rate of stimulation increased with acoustic pressure for motor movements associated with cortical activity modulation but highly depends on the region of the brain targeted. These findings emphasize the complex and yet to be determined mechanism of action involved in ultrasonic neuromodulation.

**Fig. 4 (abstract O13).**
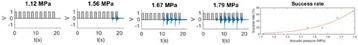
Evaluation of the pressure threshold and success rate associated with applying FUS to location within the somatosensory cortex. This location resulted in contralateral hind-limb movement relative to the sonication site. Moving the transducer symmetrically about the midline resulted again in contralateral movement relative to the new sonication site

**Fig. 5 (abstract O13).**
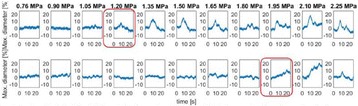
Superior colliculus (*top*) threshold determined to be approximately 1.2 MPa while the locus coeruleus (*bottom*) was evaluated to be greater than 1.8 MPa

### O14 Thermal dose effects by MR-guided focused ultrasound on the pig brain tissue - preliminary results

#### Dong-Guk Paeng^1,3^, Zhiyuan Xu^2^, John Snell^1^, Anders H. Quigg^1^, Matthew Eames^1^, Changzhu Jin^3^, Ashli C. Everstine^4^, Jason P. Sheehan^2^, Beatriz S. Lopes^5^, Neal Kassell^1^

##### ^1^Focused Ultrasound Foundation, Charlottesville, Virginia, USA; ^2^Neurosurgery, University of Virginia, Charlottesville, Virginia, USA; ^3^Ocean System Engineering, Jeju National University, Jeju, Korea (the Republic of); ^4^Biology, University of Virginia, Charlottesville, Virginia, USA; ^5^Pathology, University of Virginia, Charlottesville, Virginia, USA

**Objectives**

The objective of this research is to investigate the effects of thermal dose (TD) delivered by magnetic resonance-guided focused ultrasound (MRgFUS) on in vivo pig brain tissue. In current clinical applications of transcranial MRgFUS systems, continuous acoustic wave emission is used to heat brain tissue to peak temperatures over 58°C. However, there are some situations where it has proven difficult to reach the desired peak temperature due to high absorption of acoustic energy by skull bone. There are reports that thermal effects on tissue are well correlated with thermal dose, which suggest that treatment delivery could be prescribed in terms of thermal dose rather than peak temperature or electric/acoustic power. It is also been demonstrated that the thermal dose threshold for permanent tissue damage is about 240 cumulative equivalent minutes (CEM) at 43°C for most of tissue. Currently available transcranial MRgFUS systems only allow the prescription of acoustic power and duration. In order to investigate the effects of thermal dose on in vivo brain tissue, we have developed a closed-loop control system to allow prescription thermal dose. This system monitors tissue heating via MR thermometry and provides pulse width modulation of output acoustic power in order to hold target tissue at a fixed temperature, and hence receives a nearly constant dose rate.

**Methods**

A FUS system (ExAblate 4000 Neuro 650 kHz system, InSightec) was used for sonication and an MRI system (Discovery MR75-3.0T, GE Medical systems) was used for thermometry and pre- and post-imaging. A closed-loop control system was implemented on a personal computer to control pulse width modulation of the FUS system acoustic power in order to maintain a specified temperature based on the MR thermometry. Accumulated thermal dose was calculated in real time and used to stop the sonication so that a prescribed thermal dose was delivered to the targeted tissue. Phantom studies were performed to test the control system to prepare for animal experiments. One acute and six chronic experiments (with three day survival) were conducted to observe the effects of TD on pig brain by behaviour observation and post MR imaging of the brain (1 hour and 70 hours post procedure). Craniotomy was performed to create an acoustic access window, and sonication was applied on 4 spots in the thalamus of each pig. Histology was also performed to compare it with MR imagery. Temperature in the pig brain tissue was estimated by rectal temperature for the MR thermometry baseline. TD was varied from 7 to 200 CEM while the target temperature was changed from 46 to 52 °C with appropriate acoustic power depending on target position and individual pig. This study was approved by the University of Virginia Institutional Animal Care and Use Committee.

**Results**

From the acute experiment, we could observe the lesions on MR images after 1 hour of sonication and histology subsequently confirmed the lesions. For the chronic experiments, no obvious problem was observed in the behavior of any of the six animals. Eighteen sonication spots in 5 pigs were analyzed through MR images. One pig experiment failed to control temperature due to introduction of air bubbles between the brain and scalp during surgery procedure, and 2 sonication spots were excluded due to technical problems. Large tissue changes were observed in MR images in all 6 spots over 100 CEM.

**Fig. 6 (abstract O14).**
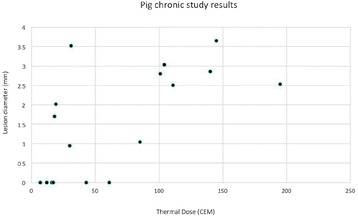
Pig chronic study results showing the relations of lesion diameter (mm) based on enhancing region appearing in T2-weighted axial MR images with applied thermal dose in CEM.

The diameter of those tissue changes in MR T2-weighted axial images were measured and averaged to 2.9 ± 0. 4 mm. There is inconsistency in generating lesions for TD below 100 CEM. No lesion was shown in some lower TD from 7 CEM and 61 CEM, while some smaller lesions (<2 mm in lesion diameter) were shown in TD from 18 CEM to 85 CEM except one large tissue change of 3.5 mm in diameter at 31 CEM. Some tissue changes were shown in both post MR images after 1 hour and 70 hours of sonication, while some were visible only at the 70 hour time point. Histology of 3 pig experiments is now available and the histology reports support the tissue changes and lesions in MR images. Lesion diameters in MR T2-weighted axial images versus TD in CEM are shown in Fig. 6 for all the results from the chronic pig study.

**Conclusions**

These preliminary results from pig brain tissue generally confirmed the previous results from rabbit brain tissue in generating tissue changes over a certain TD, even though there are some differences in the FUS systems and the experimental procedures and analysis. For lower thermal dose below 61 CEM, there is significant variability in generating of tissue changes, while large tissue changes whose average diameter is 2.9 mm were observed in MR T2-weighted axial images for higher TD over 100 CEM, which were reported with similar tendency but a little difference in TD from the rabbit brain study. These results may contribute to open the way to prescribe the thermal dose rather than peak temperature or acoustic power for brain treatments, and expand the treatment envelope beyond the current limitations in selecting targets and patients. This project is ongoing and will be further pursued with additional experiments for consolidation of the results and analysis.

### O15 In vivo feasibility study of boiling histotripsy with clinical Sonalleve system in a neurological porcine model

#### Thomas Looi^1,4^, Vera Khokhlova^2^, Charles Mougenot^5^, Kullervo Hynynen^3^, James Drake^1^

##### ^1^Hospital for Sick Children, Toronto, Ontario, Canada; ^2^University of Washington, Seattle, Washington, USA; ^3^Sunnybrook Research Institute, Toronto, Ontario, Canada; ^4^University of Toronto, Toronto, Ontario, Canada; ^5^Philips Healthcare, Markham, Ontario, Canada

**Objectives**

To determine if a clinical focused ultrasound system (Philips Sonalleve) can be used to perform mechanical liquefaction of brain tissue for neurological lesioning through a simulated fontanelle in a porcine model (simulating neo-natal patients). This work will determine the power of the system required to induce lesions using a boiling histotripsy (BH) pulsing protocol. Post-treatment, the lesion volume and border will be measured with MRI imaging and histological examination.

**Methods**

A porcine model was used as the in vivo model with a maximum weight < 6.8 kg (4.9 - 6.8kg). A horse-shoe incision and blunt dissection was used to expose the skull. A craniotomy was performed to create a 4–5 cm^2^ opening in the skull simulating the fontanelle in a neonatal patient. A degassed mixture of ultrasound gel and water (ratio 10:1) was poured on top of the dura to ensure good acoustic coupling. The scalp was sutured closed with 2–0 Vicryl cutting needle. The animal was placed supine feet first with the craniotomy centred about the Sonalleve V2 system with Flex-M surface coils. Pre-treatment T1-weighted (T1-w), T2-weighted (T2-w) and T2*-weighted (T2*-w) MRI imaging was conducted as a baseline. Each animal was treated at four cluster locations where each cluster consisted of seven sonication points; one point in the centre and six points uniformly distributed over a 4-mm diameter circle. The clusters were located approximately 15 mm deep in the brain, 7 mm off the midline, and separated by 14 mm in a rectangular geometry. In initial treatment on the first animal, the power was increased from 100 to 500 W for each cluster. After initial analysis, the treatment was repeated on second animal with refined power levels of 325, 350, 375, and 425 W. The treatment sequence consisted of 12000 pulses of 1.2 MHz frequency, 1 and 10 ms pulse duration, and 1% duty cycle for both 1 and 10 ms pulse duration. These protocols have been shown to generate BH lesions in ex vivo bovine liver in another Sonalleve system. During treatment, MR thermometry was used to monitor for surface, focal, and far field heating. A dedicated MATLAB-based interface was connected to the Sonalleve cavitation sensor to detect the signal generated during treatment points. After treatment, post T1-w, T2-w and T2*-w MRI scans were completed for comparison. The animals were euthanized, perfusion fixated and their brains were removed for histology. The brain specimens were cut at the centre for the treatment clusters to get a cross-sectional coronal view where each slice was 5 microns. The slides were stained with haematoxylin and eosin (H&E) and examined for lesion presence, blood and border definition.

**Results**

A total of 4 piglets were sonicated with the following configuration: 1 piglet with 5 clusters (100 – 500 W), 2 piglets with 4 clusters (325-425 W) using 10-ms long pulses and 1 piglet with 4 clusters and 1-ms long pulses. For all power levels, the MR-measured temperature in the near or far field of the treatment was below the noise level. For 10-ms long pulses, 100 and 200 W acoustic powers, no noticeable imaging change was observed during sonication and post-treatment MR evaluation. As power was increased from 300 to 400 W, a temperature increase of up to 5C was measured at the focus. With more discrete power levels, it appeared at 375W that the lesion was more contained whereas higher power levels created wider areas of tissue change. It was observed the timing of MR magnitude of the target cluster changed as the power level increased where the tissue change occurred at 15 s at 300 W, 12 s at 350 W, 10 s at 375 W and 5 s at 425 W. After sonication was completed, the detected temperature rise decreased immediately versus dissipating over time. This would indicate the detected thermometry was due to a phase change of the tissue rather than temperature increase. During treatment, a high amount of lower broad band emissions at frequency < 1.2MHz were detected by a cavitation sensor. Post-treatment MR imaging showed that at power levels between 300 and 400 W, there were areas of hypointensity indicating the lesion. H&E staining confirmed the presence of the mechanical lesion where various anatomical targets were fractionated. Power levels were sufficient to rupture vessels and cause a focused area of haemorrhage at the treatment cluster. It showed that BH treatment dissolved the anterior ventricle wall with presence of elements of blood. H&E staining also showed that maximum lesion diameter was approximate 7 mm in coronal plane therefore the treatment borders matched the treatment plan. For the piglet treated with 1-ms long pulses, the post-treatment imaging change was not noticeable. However, at 375 and 400 W power, H&E slides showed two areas where there was a perforation of the anterior ventricle wall with a lesion size of up to 2 mm. It appeared that the shorter pulse duration generated smaller but more focused lesions.

**Conclusions**

This pilot study shows that the clinical Sonalleve system is capable of generating mechanical ablation of a brain tissue in an in vivo porcine model using boiling histotripsy pulsing scheme. The power threshold to initiate lesions in brain using 10 ms pulses (375 W) was found to be similar the power levels used in BH studies in ex vivo bovine liver and porcine kidney tissue at a similar depth in tissue (250 – 300 W) in a Sonalleve V2 system at the University of Washington. The treatments can be accelerated by using higher power outputs and shorter pulses. H&E histological evaluation showed that BH treatment caused rupture of vessels focally while also creating wider well defined areas of mechanical ablation with no damage to surrounding tissue. Additional work is underway to characterize the pressure levels generated by the Sonalleve to correlate the power and pressure for treatment.

**Fig. 7 (abstract O15).**
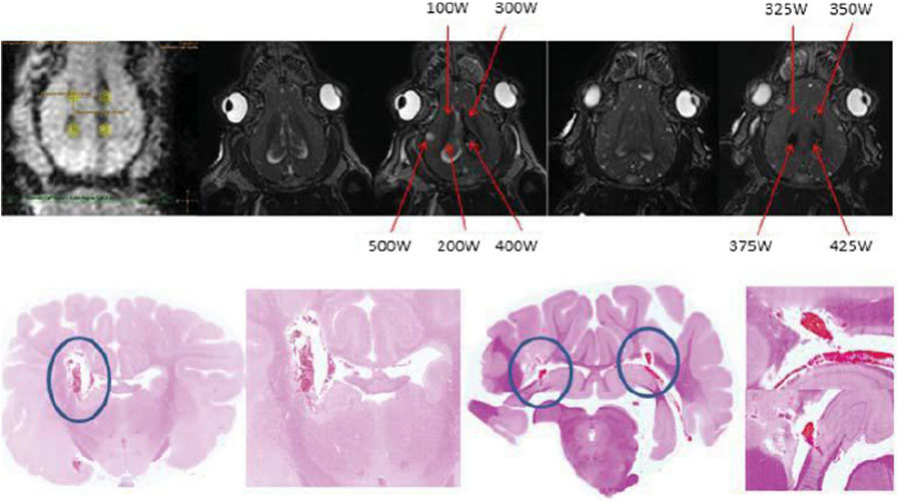
Top Row: Left to Right: Treatment area, Pre T2-w (100–500 W), Post T2-w (100–500 W), Pre- T2-w (325–425) and Post T2-w (325–425); Bottom Row: Histology Left to Right: 10 ms treatment (375W), 10 ms treatment - zoomed (375W), 1ms treatment (375W and 425W), 1 ms treatment – zoomed.

### O16 Musculoskeletal clinical applications of intense therapy ultrasound (ITU): part 1. Clinical study for plantar fasciitis

#### Michael Slayton^1^, Richard C. Amodei^1^, Keegan Compton^1^, Ashley McNelly^2^, Daniel Latt^2^

##### ^1^Guided Therapy Systems, LLC, Mesa, Arizona, USA; ^2^School of Medicine, University of Arizona, Tucson, Arizona, USA

**Objectives**

Chronic Plantar fasciitis (CPF) is a common cause of plantar heel pain that is a result of a degenerative process of the plantar fascia and its surrounding perifascial structures [1]. It is the most common cause of heel pain, affecting 10% of the U.S. population, and one of the most common foot and ankle problems.

More than twenty different treatments have been used for plantar fasciitis. Conservative treatments (rest, ice, stretching and NSAIDs) have been shown to effectively treat symptoms but 10% of patients fail conservative management and continue to have symptoms within 12 months and beyond. Surgery consisting of partial PF release is often considered with 50% of patients having residual symptoms, in addition to surgical risk exposure.

High frequency ITU, a novel potential approach to treating Plantar Fasciosis, was studied for the creation of small thermal injuries noninvasively inside symptomatic Plantar Fascia (PF). It has been shown to initiate a tissue repair cascade and promote collagen generation in musculoskeletal tissue [2, 3]. A double blinded, randomized, sham controlled clinical study for ITU treatment of chronic Plantar Fasciitis has been conducted by IRB approved clinical protocol to access clinical efficacy of the procedure.

**Methods**

Custom 3.2 MHz high intensity (10 kW/cm^2^) ultrasound therapy system was designed and fabricated (GTS, Mesa, AZ, USA). Field simulations, testing and Schlieren images verified intensity, high focal pressure (17.3 MPa) and focal distance of 13–15 mm.

Each treatment consisted of 250–320 100 ms pulses creating matrices of small ablative thermal lesions of 4–5 joules at pre-programmed pitch of 1.6 mm. Each patient underwent two treatment sessions in 2 weeks, each treatment time did not exceed 12 minutes. ITU placebo group consisted of the same treatment with energy set to 0.

Treatment effects were assessed with diagnostic imaging ultrasound at 12 MHz (Spark, Ardent Sound, Mesa, AZ, USA) by a certified sonographer. Ultrasound images were analysed to determine symptomatic hypoechoic lesion size with PF.

Patient reported outcomes consisted of PROMIS physical function computer adaptive test (PF-CAT), PROMIS global health, Foot Function Index pain subscale (FFIPS) [4, 5] and a non-validated heel pain specific questionnaire.

Clinical protocol included (35) patients diagnosed with chronic heel pain due to Plantar Fasciitis (more than 3 months) and failed conventional therapy treatments.

Patients were randomized to standard therapy (anti-inflammatory pills, stretching and gel heel cups) plus ITU (“Treatment” group, n=26) of standard therapy plus sham ITU (“Control” group, n=9).

Primary investigator, sonographer and study coordinator administering the study were blinded to group assignments. P-values were calculated via 2-tailed paired T-tests for both treatment and control groups.

**Results**

Patient-Reported Outcome Measures: Compared to the baseline assessment of Pain, the Treatment Group showed significantly improved pain scores compared to the Control (sham treatment) Group in follow-up visits including 12 weeks after the initial treatment.

Foot Function Index Pain Score: Compared to the baseline assessment, the treatment group pain scores also showed significant improvement compared to the sham group.

Diagnostic Ultrasound Imaging: During the 12 week follow-up period changes to the overall thickness of the PF were not statistically significant, while calculated volume size of hypoechoic lesions within the PF, just distal to the Calcaneus, showed significant change. For the experimental group (n=28) the average hypoechoic lesion volume reduction was followed and compared to the baseline measurements just before the first Treatment; 2 week follow-up and 2nd treatment date (−28%), 4 weeks (−50%), 6 weeks (−66%) and 12 weeks (−80%).

For the control group (n=10), the average hypoechoic lesion volume was followed and compared to the baseline just before the first Treatment; 2 weeks and 2nd sham treatment (+9%), at 4 weeks (+16%), 6 week (+29%) and 12 weeks (+31%). Unlike the experimental group, these lesions grew in size during the follow-up period.

P-values calculated for all outcome results discussed above for both treatment and control groups were below .01, showing the statistical significance of the results.

**Conclusions**

1. Results of the double blinded randomized, sham controlled study for the treatment of Plantar Fasciitis with ITU appeared to have statistically significant positive results within 12 weeks post-treatment in 80% of treated subjects.
2. Both quantitative measurements from diagnostic ultrasound imaging and applied standardized assessment protocols consisting of PROMIS PF-CAT, FFIPS along with Patient Reported Outcome Measures showed statistically significant coincidental improvements in treated subjects vs. control group.
3. Intense Therapeutic Ultrasound has shown potential for effective treatment of Chronic Plantar Fasciitis. Better designed studies with increased # of subjects will be considered to support ITU as an effective tool for the proposed clinical treatment.

**References**

[1] Neufeld S.K. and Cerrato R.; *Plantar fasciitis: evaluation and treatment.* J Am Acad Orthop Surg, June 2008, 16(6): p. 338–46.

[2] Slayton M. and Barton J.; *Healing tissue response with ITU (Intense Therapy Ultrasound) in musculoskeletal tissue, feasibility study.* Ultrasonics Symposium (IUS), 2014 IEEE International, Chicago, USA, p.1654–1657.

[3] Slayton M. H., Amodei R. C., McNelly A. and Latt D. L.; *Intense Therapy Ultrasound (ITU) for the treatment of Chronic Plantar Fasciitis: Preliminary Results of Clinical Study.* 37th International Conference of the IEEE Engineering in Medicine and Biology; Milan, Italy, August 2015.

[4] Rose, M., Bjorner, J.B., Becker J., Fries J.F. and Ware J.E.; *Evaluation of a preliminary physical function item bank supported the expected advantages of the Patient-Reported Outcomes Measurement Information System (PROMIS)*. J. Clin. Epidemiology, January2008, 61(1): p. 17–33.

[5] Budiman-Mak E., Conrad K. J. and Roach K. E.; *The Foot Function Index; a measure of foot pain and disability.* J. Clin. Epidemiology, 1991, 44(6): p. 561–579.

**Fig. 8 (abstract O16).**
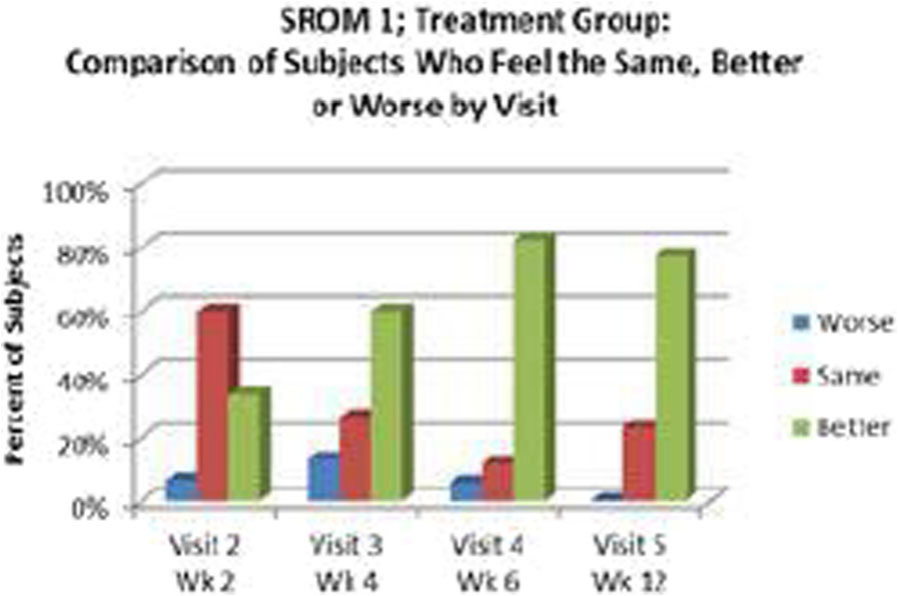
Treatment Group SROM 1

**Fig. 9 (abstract O16).**
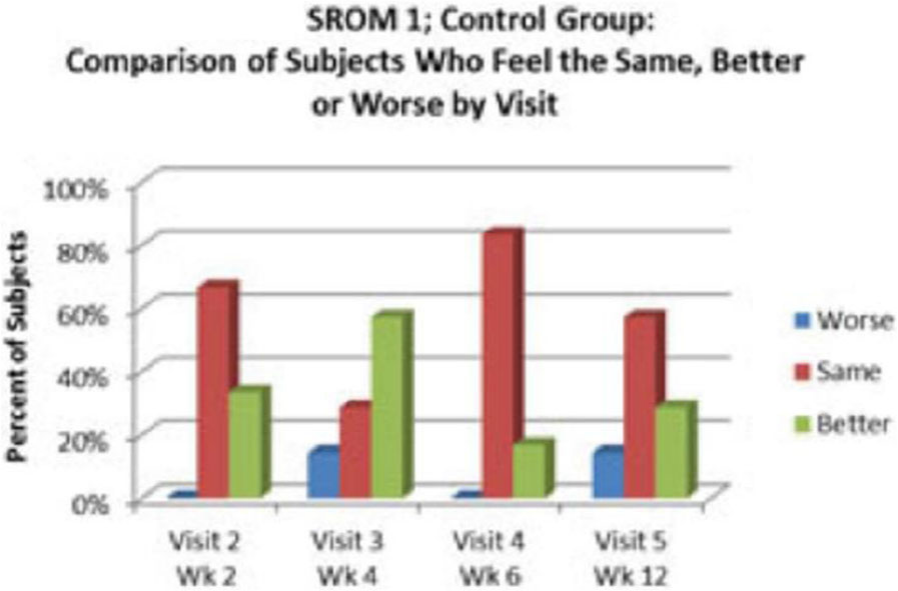
Control Group SROM 1

**Fig. 10 (abstract O16).**
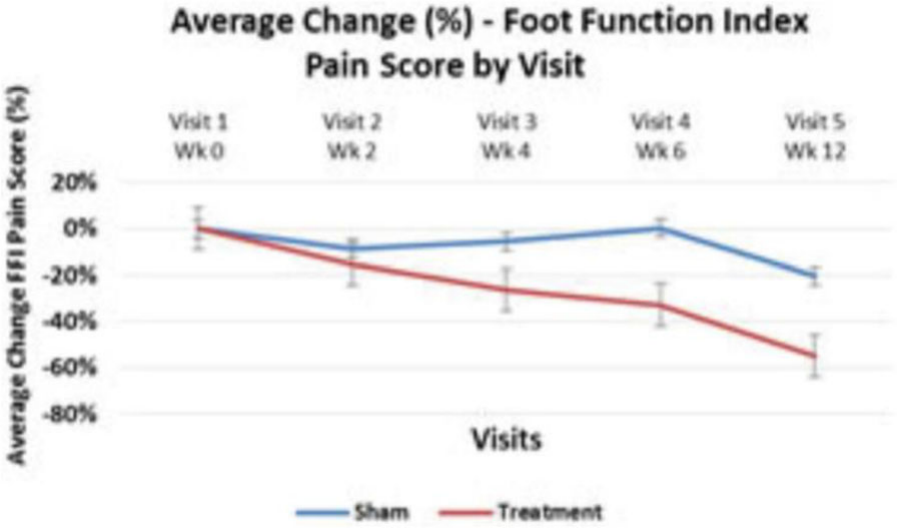
FFIP Score by Visit

**Fig. 11 (abstract O16).**
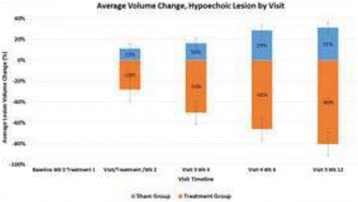
Average Lesion Volume change by Visit. SE applied.

### O17 Musculoskeletal clinical applications of intense therapy ultrasound (ITU): part 2. Initial results of clinical study for lateral epicondylitis

#### Michael Slayton^1^, Richard C. Amodei^1^, Keegan Compton^1^, John Kearney^2^

##### ^1^Guided Therapy Systems, Mesa, Arizona, USA; ^2^The CORE Institute, Phoenix, Arizona, USA

**Objectives**

Acute and Chronic pain of the Common Extensor Tendon (CET) region, lateral epicondylitis or tennis elbow is a common pathology of both athletes and non-athletes affecting up to 3% of the population at large [1], while the prevalence of chronic problems caused by overuse in tennis players can be as high as 40%. Elbow tendinopathy represents an important set of pathologies that account for lost recreation time, decreased quality of life, and work-related disability claims. Conservative treatment of the Epicondylitis or -osis is recommended as the initial strategy by most authors. This strategy includes identification and correction of possible etiological factors, and a symptom related approach. Generally, the initial treatment consists of a multifactorial approach that may include a combination of rest (complete or modified activity), medication (NSAIDs for Epicondylitis), stretching and strength training. More aggressive treatments for CET include: Cortisone injection, Plasma Rich Platelets (PRP), Tenotomy, ESWT.

High frequency ITU, a novel potential treatment for CET, was studied for the creation of small thermal injuries noninvasively inside symptomatic Common Extensor Tendon (CET). It has been shown to initiate a tissue repair cascade and promote collagen generation in musculoskeletal tissue [2–5]. A blinded, randomized, clinical study for ITU treatment of chronic Lateral Epicondylitis has been conducted by IRB approved clinical protocol to assess clinical efficacy of the procedure.

**Methods**

Custom 4.5 MHz high intensity (47.9 KW/cm^2^) ultrasound therapy system was designed and fabricated (GTS, Mesa, AZ, USA). Field simulations, testing and Schlieren images verified intensity, high focal pressure (37.9 MPa) and focal distance of 6 mm.

Each treatment consisted of 80 14 ms pulses creating matrices of small ablative thermal lesions of 1 joule at manually targeted area set by diagnostic ultrasound imaging. Each subject underwent two treatment sessions 4 weeks apart. Each treatment time did not exceed 10 minutes. Treatment effects were assessed with diagnostic imaging ultrasound at 20 MHz (Spark, Ardent Sound, Mesa, AZ, USA) by a certified sonographer. Ultrasound images were analysed to determine changes in the peri-tendon region, including hypoechoic areas, calcifications and dependent free fluid.

Subject reported outcomes consisted of PRTEE survey [6], physical examination, Universal Analog Visual Pain Score17 and a Patient Reported Satisfaction Survey [7].

Clinical protocol includes 25 subjects diagnosed with chronic Tennis Elbow, or Lateral Epicondylosis (more than 3 months) and failed conventional therapy treatments.

Subjects were subjected to standard therapy (stretching and strength exercises, hot and cold compresses and compression support) plus ITU.

Primary investigator, sonographer and study coordinator administering the study were blinded to group assignments. P-values were calculated via 2-tailed paired T-tests at each visit of the clinical study.

**Results**

The results presented below are initial findings for the first 12 subjects currently being followed through the study.

**Fig. 12 (abstract O17).**
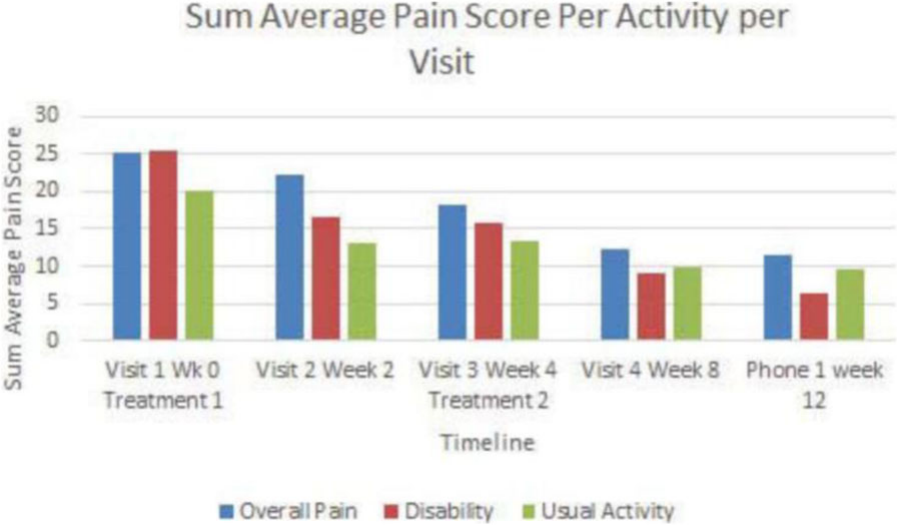
See text for description

PRTEE: Patient Reported Tennis Elbow Evaluation Final Score is a weighted Pain Score based on 15 questions grouped into 3 categories: Overall Pain, Functional Disability and Usual Activities. Subjects respond to each question with a Pain Score of 0–10. Each category is then summed and weighted with a maximum score of 100 (Overall Pain 100%, Functional Disability 50% and Usual Activities (50%), n=12, Fig. 12.

**Fig. 13 (abstract O17).**
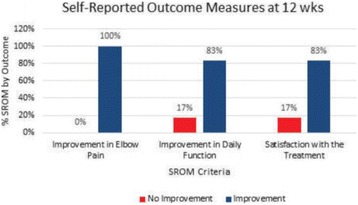
See text for description

Self-Reported Outcome Measures Surveys show a significant improvement and treatment satisfaction with Subjects reporting improvements in elbow pain 100%, improvement in Daily function 83% (vs. 17% no improvement) and treatment satisfaction 83% (vs. 17% not satisfied), n=6, Fig. 13.

**Fig. 14 (abstract O17).**
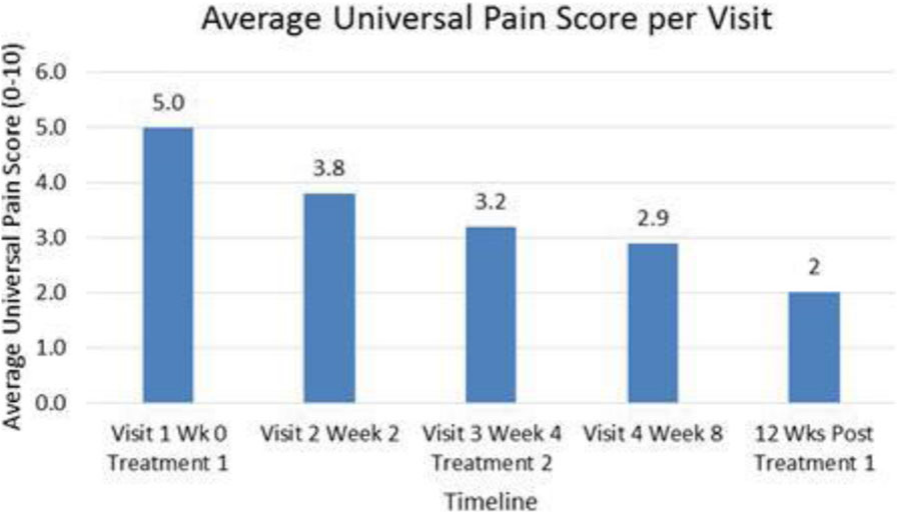
See text for description

Universal Analog Pain Scores also show progressive reduction (−3 on a 10 point scale) throughout the same period, n=12, Fig. 14.

Diagnostic Ultrasound Images: Diagnostic Ultrasound Images show a consistent increase of free fluid 2 weeks after the first treatment, with a progressive reduction in free fluid at 8 weeks in subjects with no to mild peri-tendon calcifications. These subjects correlated well with improving PRTEE survey scores. Subjects with little or no improvement in PRTEE scores consistently presented with moderate to severe peri-tendon calcifications.

P-values calculated for the above reported outcomes were not statistically significant for Visits 2 and 3 (P>0.05) while results for Visits 4 and 5 demonstrated P< 0.05.

**Conclusions**

1. Feasibility of Intense Therapeutic Ultrasound treatments of chronic pain in CET region has been established with the initial results (n=12) of the ongoing clinical study.
2. Significant reduction of pain scores per activities (PRTEE) and Self-Reported Outcome Measures (83% improvement) with average Universal Pain Scores reduction from 5.0 to 2.0 were statistically significant (p<0.05) at 8 and 12 weeks post treatment.

**References**

[1] Hong QN, Durand MJ, Loisel P. *Treatment of lateral epicondylitis: where is the evidence? Joint Bone Spine 2004; 71(5):369–373.*

[2] White, W. M., I. R. Makin, et al. (2007). Selective creation of thermal injury zones in the superficial musculoaponeurotic system using intense ultrasound therapy: a new target for noninvasive facial rejuvenation." Arch Facial Plast Surg 9(1): 22–29.

[3] Gliklich R, White WM, Barthe PG, Slayton MH, Makin IRS. Clinical pilot study of intense ultrasound (IUS) therapy to deep dermal facial skin and subcutaneous tissues. Arch Facial Plast Surg 2007; 9:88–95.

[4] Slayton M., Barton J, Feasibility of Modulating Healing Tissue Response by ITU (Intense Therapy Ultrasound) in Musculoskeletal Tissue ASLMS 2014 Annual Conference.

[5] Slayton M., Barton J., Healing tissue response with ITU (Intense Therapy Ultrasound) in musculoskeletal tissue, feasibility study, Ultrasonics Symposium (IUS), 2014 IEEE International, pp. 1654–1657. DOI 10.1109/ULTSYM.2014.010

[6] Rompe JD1, Overend TJ, MacDermid JC. Validation of the Patient-rated Tennis Elbow Evaluation Questionnaire.J Hand Ther. 2007 Jan-Mar; 2007 (1):3–10: quiz 11.

[7] Rose, M., et al., *Evaluation of a preliminary physical function item bank supported the expected advantages of the Patient-Reported Outcomes Measurement Information System (PROMIS).* J Clin Epidemiol, 2008. 61(1): p. 17–33.

### O18 Clinical experience of intra-operative high intensity focused ultrasound in patients with colorectal liver metastases. Results of a phase ii study.

#### David Melodelima^1,2^, Aurelien Dupre^2^, Yao Chen^2^, David Perol^2^, Jeremy Vincenot^1^, Jean-Yves Chapelon^1^, Michel Rivoire^2,1^

##### ^1^LabTAU - U1032, INSERM, Lyon, France; ^2^Centre Leon Berard, Lyon, France

**Objectives**

Managing colorectal liver metastases (CLM) is a major clinical challenge, and surgery remains the only potentially curative treatment. Five-year survival rates of up to 51% have recently been reported. However, only 10–20% of patients are eligible for surgery, which is often precluded by the number, size and/or location of metastases, or because the necessary resection will leave insufficient volume of functional liver. Radiofrequency ablation (RFA) is the main technology that has been used in association with surgery as a tool to expand the number of patients who may be candidates for liver directed therapy. However, RFA has several limitations. There is a risk of inadequate treatment due to the heat sink effect of blood flow, RFA does not allow reliable real-time monitoring, and it require intra-parenchymal introduction of a probe. Moreover, only small hepatic volumes can be targeted. These limitations could explain the high rates of local recurrence seen after RFA.

High intensity focused ultrasound (HIFU) has been proven effective in a wide range of clinical applications, especially prostate cancer. The ablation achieved by conventional HIFU is small and ellipsoidal. The dimensions vary according to transducer characteristics but are typically 1–3 mm (transverse) and 8–15 mm (along beam axis). In clinical practice, hundreds of superimposed ablations are required; and the procedure may take up to two hours. Even so, HIFU has several potential advantages in the treatment of liver tumours: there is no need to puncture the parenchyma, the extent of the thermal lesions achieved is not reduced by hepatic perfusion, and it is possible to monitor the effects of therapy in real time. However, extra-corporeal treatment of the liver is difficult because presence of the ribcage may stop propagation of ultrasound waves and respiratory motion may cause targeting problems. HIFU treatment of CLM needs to be improved, and reducing the duration of surgical intervention by increasing the size of ablated fields is particularly important. A HIFU device enabling destruction of larger liver volumes has been developed based on toroidal transducers.

Preliminary in vitro and preclinical work demonstrated the potential, feasibility and safety of such HIFU ablations. During laparotomy in a porcine model we demonstrated that this HIFU device achieves reproducible ablations with an average volume of 7 cm3 (with 20 mm diameter and 25 mm long axis) in 40 seconds. Such preclinical work has to be translated into clinical practice through controlled trials, and the aim of this study was to assess the feasibility and safety of HIFU ablation in patients undergoing hepatectomy for CLM, as well to collect efficacy and accuracy data. This study is registered with Clinical-Trials.gov (NCT01489787).

**Methods**

This study was a prospective, single-centre phase I/II study evaluating the feasibility, safety and accuracy of HIFU during surgery in patients with CLM. The protocol was reviewed and validated by a national ethics committee (CPP Sud-Est IV) according to French and European directives. Since this study was the first use in man of intra-operative hepatic HIFU, ablations were made only in areas of liver scheduled for resection. This allowed real-time evaluation of HIFU ablation while protecting participating patients from any adverse effects related to this new technique. The transducer has a toroidal shape 70 mm in diameter and is divided into 32 ultrasound emitters of 0.13 cm^2^ operating at 3 MHz. The radius of curvature is 70 mm. A 7.5 MHz ultrasound imaging probe was placed in the centre of the device and was used to guide the treatment. The imaging plane was aligned with the HIFU focal zone.

Six patients were included in the Phase I. Two single thermal ablations were created in each patient. Thirteen patients were included in Phase IIa and two HIFU ablations were to be placed precisely in a target previously identified in ultrasound images (step 1) and then at distance (step 2) from a target. Five patients were included in Phase IIb until now. HIFU ablations were created to ablate metastases (20 mm maximal diameter) with safety margins in all directions. The exposure time varied from 40 s to 370 s according to the diameter of the metastases to be treated.

**Results**

In agreement with preclinical studies, the demarcation between ablated and non-ablated tissue was clearly apparent in ultrasound images and histology. The dimensions measured on ultrasound imaging were correlated (r=0.88, p<0.0001) with dimensions measured during histological analysis. All HIFU ablations were obtained in 40 seconds. The average dimensions obtained from each HIFU ablation were a diameter of 21.0 ± 3.9 mm and a depth of 27.5 ± 6.0 mm. The phase IIa study showed both that the area of ablation could be precisely targeted on a previously implanted metallic mark and that ablations could be created deliberately to avoid such a mark. Ablations were achieved with a precision of 1–2 mm. In Phase IIb, one metastasis of 10 mm in diameter was ablated in 40 seconds with safety margins. Using electronic focusing metastases of 2 cm in diameter were ablated with safety margins (>3 mm in all directions) in 370 seconds. The dimensions of HIFU ablations created in 370 s were a diameter of 48 mm and a long axis of 51 mm.

**Conclusions**

This new HIFU device safely achieved large volume liver ablations in 40 s, with a precision of one to two millimetres under real-time monitoring. HIFU ablations of small metastases (<20 mm) and peri-lesional healthy liver were successfully created with planned safety margins of at least 3 mm in all directions.

### O19 Chemotherapy in oral cancer

#### Wei Guo, Guoxin Ren, Guofeng Shen

##### Department of Oral Maxillofacial and Head Neck Oncology, Shanghai 9th People's Hospital, Shanghai, China

**Objectives**

To evaluate the efficacy and the main side-effects during the clinical trial of this new ultrasound hyperthermia system combined with chemotherapy in oral cancer, meanwhile, to observe the preliminary clinical response of this combined therapeutic modality.

**Methods**

Thirty four cases of oral squamous cell carcinoma entered this clinical trial, from which 23 had advanced oral carcinoma and were treated with new ultrasound hyperthermia system combined plus docetaxel–cisplatin–fluorouracil regimen (test group). Eleven patients only received chemotherapy with docetaxel–cisplatin–fluorouracil regimen (control group). The thermo-index were detected during the course of hyperthermia, the chief-complain of the patients were also recorded. The systemic physiological, biochemical and immunological index were tested before and after the treatment respectively. The therapeutic response was estimated 1 month after 2 cycles of the treatment.

**Results**

Twenty three cases of oral squamous cell carcinoma enrolled the clinical trial of local ultrasound hyperthermia combined with chemotherapy. 230 times of ultrasound hyperthermia in total were performed. The ultrasound hyperthermia system operated smoothly, no malfunction was found. The main thermo-index were: the maximum heating temperature was108 F, the average heating temperature was 106 F, the minimum heating temperature was 104 F, the fraction of heating time more than 108 F was 0.46, the average treatment time was 37.74±8.88min. PR+CR was 71% (test group). The main local side-effects were low-grade pain (6/23). The incidence of adverse effects was similar between both study groups, no bone marrow suppression (over III).

**Conclusions**

The system combined with docetaxel–cisplatin–fluorouracil regimen is effective and safe in the treatment of advanced oral cancer. The main side-effects of local ultrasound hyperthermia combined with chemotherapy are low-grade pain or tolerable pain. There is no immune function and obtains satisfying short-term response.

### O20 Non-thermal, non-cavitational, 20kHz Ultrasound applicators in wound healing

#### Michael Neidrauer^1^, Leonid Zubkov^1^, Michael S. Weingarten^2^, David J. Margolis^3^, Peter A. Lewin^1^

##### ^1^The School of Biomedical Engineering, Science and Health Systems, Drexel University, Philadelphia, Pennsylvania, USA; ^2^Department of Surgery, College of Medicine, Drexel University, Philadelphia, Pennsylvania, USA; ^3^Biostatistics and Epidemiology, University of Pennsylvania, Philadelphia, Pennsylvania, USA

**Objectives**

This talk examines the challenges associated with the design of clinically viable ultrasound applicators operating at the relatively low frequency (20 kHz) and intensity (<100 mW/cm^2^, spatial peak, temporal peak) levels, and tailored to treatment of chronic wounds, such as venous or diabetic ulcers. These challenges were associated with the architecture and weight, and principle and efficiency of operation, including electrical power consumption. The ultimate goal of this work was to test the efficacy of the applicators in human subjects.

**Methods**

A fully wearable Band-Aid™-like, dial-in delivery, battery-operated ultrasound applicator was designed. The applicator included light weight (<25g) piezoelectric flexural transducer and was powered by 10-12V fully rechargeable lithium-ion batteries (total weight <200g); it was able to operate for up to 4 hours between re-charging. To emphasize the uniqueness of the design, it might be useful to note that typically, the thickness of the capacitive piezoelectric element is inversely proportional to the frequency, therefore a 20 kHz element would need to be 10 cm thick. Such element would be bulky and require hundreds of volts (demanding a large power amplifier), in excitation signal thus eliminating any chance of being a portable design. To overcome this, a mechanical displacement amplifier, which translates 2 MHz ultrasound waves into 20 kHz output at the desired pressure amplitude (55 kPa; i.e. 100 mW/cm^2^) with only 12 volts excitation was chosen as a preferred solution.

The applicators were extensively tested to ensure that the ultrasound field energy was below the level needed to generate inertial cavitation and any temperature elevation that would exceed 1°C. Also, the uniformity of the acoustic field distribution was verified. The pilot study included 32 individuals between ages of 18 and 80 having venous (n=23) or diabetic (n= 9) wounds that remained open for a minimum of 8 weeks. In compliance with the IRB study protocol the subjects were randomly assigned to either treatment or control group, with an equal chance of being assigned to receive active ultrasound treatment or sham (current standard care). Treatment sessions lasted 15 minutes and were administered once a week for a period of 12 treatments, or until the wound’s closure. Clinical efficacy was evaluated by measuring the reduction in wound area over time. For both etiologies, i.e. both venous and diabetic wounds the rate of closure was statistically faster (p<.05) in the treated group compared to the control group.

**Results**

The study findings show that the ultrasound treated venous ulcer group had statistically improved (p<0.04) rate of wound size change (reduction of 14.3%/week) compared to the rate of wound size change for the control group (increase of 3.6%/week on average). Diabetic wound closure was achieved typically after 4 sessions for treated wounds, as opposed to 7 sessions for the control group. Time to heal was also statistically faster (p< .05) for treated wounds (~5 weeks) when compared to non-treated wounds (~12 weeks).

**Conclusions**

Overall, the results from this study support the notion that low frequency ultrasound treatment can successfully improve healing outcomes in chronic wounds with different morphology and etiology. The evaluated device used safe levels (<100mW/cm^2^ ISPTP) of ultrasound energy and featured unique portability, which opens possibility for personalized home treatment of chronic wounds in the future.

**Acknowledgements**

*NIH Grant R01 EB9670, NSF 1064802, Wallace H. Coulter Foundation.*

### O21 Tests of thermal ablation with a 230 kHz transcranial MRI-guided focused ultrasound system in a large animal model

#### Nathan McDannold^1^, Jonathan Sutton^1^, Natalia Vykhodtseva^1^, Margaret Livingstone^2^

##### ^1^Radiology, Brigham and Women's Hospital, Boston, Massachusetts, USA; ^2^Neurobiology, Harvard Medical School, Boston, Massachusetts, USA

**Objectives**

Thermal ablation with transcranial MRI-guided focused ultrasound (TcMRgFUS) is being tested clinically for as an alternative to surgery for functional neurosurgery and brain tumour resection. The current TcMRgFUS system, which operates at 650–670 kHz, is limited by skull heating to a small central region in the brain. Use of a lower acoustic frequency will reduce skull heating, but at the same time the focal heating will decrease and the risks of uncontrolled cavitation (the formation of microbubbles) increase. The purpose of this study was to evaluate the feasibility of thermal ablation in nonhuman primates using a system that operates at a lower acoustic frequency and to determine whether it can increase the “treatment envelope” for TcMRgFUS.

**Methods**

The experiments were approved by our institutional animal committee. Thermal ablation with the 230 kHz ExAblate Neuro system (InSighTec) was tested over five sessions in three rhesus macaques. In each session a target in the thalamus was sonicated transcranially at 40–50 s at acoustic power levels ranging from 90–560 W. The TcMRgFUS system software modulated the acoustic power in real time with a closed-loop controller that maintained a low-level of acoustic emissions, which are correlated with cavitation activity. MR temperature imaging (MRTI) was acquired at 3T (LX, GE) in a single plane using a 14 cm surface coil (TR/TE: 29/13 ms; flip angle: 30°). Measurements of the peak temperature rise at the focus and on the outer brain surface were compared for the different animals as a function of the applied acoustic energy. For the brain surface we measured the average temperature at the hottest 5% of the voxels and over a two voxel wide strip and we also normalized the measurement by the outer skull area. Temperature measurements were used to calculate the accumulated thermal dose, which was then compared to post-sonication T2-weighted, T2*-weighted, and contrast-enhanced T1-weighted MRI. The focal and skull-induced heating on the brain surface were compared to an earlier study performed in macaques with a 650 kHz version of this system.

**Results**

**Fig. 15 (abstract O21).**
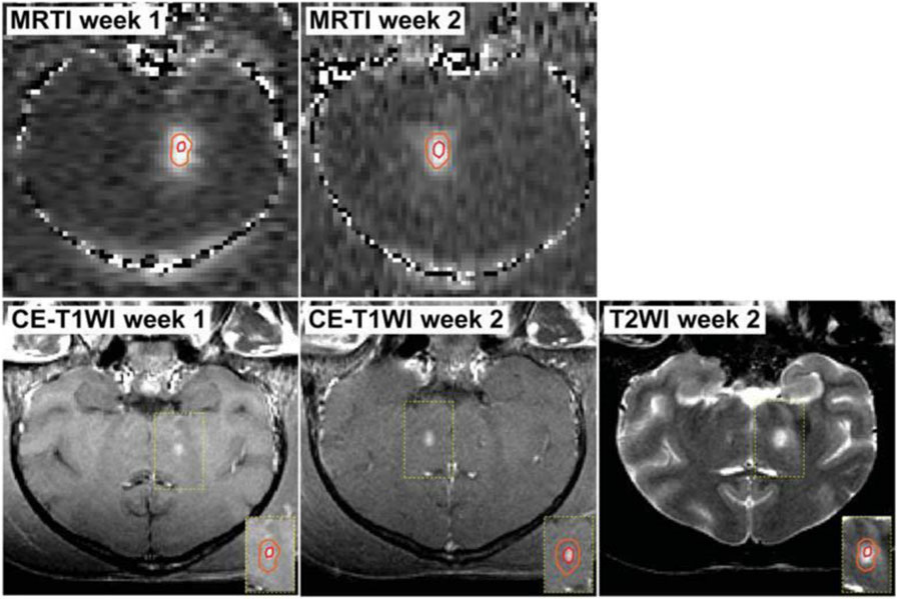
MRTI (*top*) and post-FUS imaging (*bottom*) obtained in two sessions in Monkey 1. Thermal dose contours at 30 (orange) and 240 (red) CEM43C were calculated from the MRTI. Immediately after each session, a small lesion was observed in contrast-enhanced T1-weighted MRI (CE-T1WI). The dimensions of this area were consistent with the 240 CEM43 contours. The lesion produced in session 1 was largely non-enhancing in in CE-T1WI at week 2. It was visible in T2-weighted imaging (T2WI) and increased in size

Focal heating sufficient to create an MRI-evident thermal lesion was achieved in 4/6 targets; the peak thermal dose exceeded 240 CEM43°C at these targets (Fig. 15). Heating at the focus was slightly higher than that measured on the brain surface. The focal heating increased linearly as a function of the applied energy at a rate of 3.2 ± 0.4°C per kJ (R2: 0.81). The surface area of the outer skull ranged from 47–55 cm2. For the hottest 5% of the voxels on the brain surface included in the MRTI imaging plane, the temperature rise increased linearly as a function of temperature at a rate of 126.6 ± 7.3°C per kJ/cm^2^. For a two voxel wide strip over the entire brain surface, this increase was 62.7 ± 7.5°C per kJ/cm^2^. The extent of MRI-evident changes (apparent oedema in T2-weighted MRI, BBB disruption post-contrast, no petechiae in T2*-weighted MRI) were consistent with 240 CEM43°C contours. One lesion imaged one week after FUS increased in size.

**Conclusions**

Analyses of the MRTI and post-sonication MRI suggest that the lesions were consistent with thermal mechanisms. The temperature rise increased linearly with the applied energy, and no evidence of cavitation-related petechiae were evident after sonication. The MRI-evident lesions were consistent with isodose contours drawn at 240 CEM43°C, a conservative threshold often used to guide thermal ablation. However, since it is known that thermal damage can take several hours to manifest in MRI and the lesion we imaged at one week increased in size, it is likely that the size of the lesion was underestimated by this dose value.

Similar tests in macaques with a version of this system operating at 670 kHz (Hynynen et al., Eur J Radiol 2006; 59: 149–56) measured skull-induced heating of 130°C per kJ/cm^2^ of outer skull surface, more than twice of that measured here (63°C per kJ/cm^2^). While no or minimal focal heating was observed at 670 kHz, with this 230 kHz system we were able to reach ablation-level thermal dose values. Thus, these preliminary results thus suggest that this low frequency system can expand the area of the brain that can be targeted for thermal ablation without overheating the skull. The closed-loop feedback system successfully maintained a low level of acoustic emission (and presumably microbubble activity) and immediately stopped the sonication when excessive levels were detected. However, additional work is needed to understand whether low-level cavitation activity played a role in the focal heating, to characterize the lesions in histology, and to examine whether safe cavitation levels can be maintained in tumours where the cavitation threshold may vary.

### O22 Growth slowdown in a brain metastasis model by antibody delivery using focused ultrasound-mediated blood–brain barrier disruption

#### Thiele Kobus^1,2^, Yong-Zhi Zhang^2^, Natalia Vykhodtseva^2^, Nathan McDannold^2^

##### ^1^Radiology and Nuclear Medicine, Radboud University Medical Center, Nijmegen, Netherlands; ^2^Radiology, Brigham and Women's Hospital, Boston, Massachusetts, USA

**Objectives**

HER2-targeting antibodies prolong survival in patients with HER2-positive breast cancer metastases outside the brain. However, the response of brain metastases to these drugs is poor and it is hypothesized that the blood–brain barrier (BBB) limits drug delivery to the brain. We aim to improve delivery by temporary disruption of the BBB using focused ultrasound (FUS). Here we evaluate the treatment benefit of combining two antibody therapies that target the HER2-receptor with FUS-mediated BBB disruption in a breast cancer brain metastasis model.

**Methods**

MDA-MB-361 HER2-positive human cancer cells were injected in the right brain hemisphere of nude rats. The animals were divided in three treatment groups of 10 animals each: the control-group received no treatment; the antibody-only group received trastuzumab and pertuzumab (antibodies that target the HER2-receptor); and FUS+antibody-group received trastuzumab and pertuzumab in combination with FUS-mediated BBB disruption. The six weekly treatments started five weeks after tumour implantation, when the tumour diameter was around 2 mm. The FUS treatments took place in a 7T MR-scanner using a single-element, spherically-focused 690 kHz-transducer. Trastuzumab and pertuzumab were injected before the first sonication. At the start of each sonication (duration 60s, 10-ms bursts, burst repetition frequency 1 Hz), the ultrasound contrast agent Optison (100 μl/kg) was injected. The complete tumour was treated in 4 to 14 sonications that were separated 1 to 1.5 mm. Peak negative pressure amplitudes in water between 0.46 and 0.62 MPa were used.

Before and after the sonications, MR imaging was performed consisting of T2-weighted (T2w), T1w and T2*w imaging to locate the tumour, confirm BBB disruption and study the presence of hemorrhages. In two animals tumour leakiness was studied by comparing T1w imaging before and after gadolinium injection before the tumours were sonicated. The difference in signal intensity change in pre- and post-contrast T1w images was determined between the tumour and contralateral brain region (= SI%). In all FUS-treated animals BBB disruption was confirmed with contrast-enhanced T1w imaging and quantified using the same method as for the tumour leakiness. Pre- and post-sonication T2*w images were inspected for hypo-intense regions, which can indicate extravasated erythrocytes.

Every other week, high-resolution T2w imaging was performed to determine tumour volume. The growth rate (r) was determined by fitting the tumour volumes to the following formula: volume(t)=a*exp(r*t), in which t is the time in days. The growth rate of each tumour was determined for the treatment period (week 5 to 11) and the follow-up period (week 11 till sacrifice). An animal was classified as ‘responder’ if the growth rate was lower than the mean growth rate of the control animals minus two standard deviations.

The animals were euthanized if the tumour size exceeded 13 mm in diameter or if the condition of the animal was poor. From nine animals, histology was obtained (hematoxylin and eosin (H&E)) and the brains of five animals were stained for HER2.

**Results**

**Fig. 16 (abstract O22).**
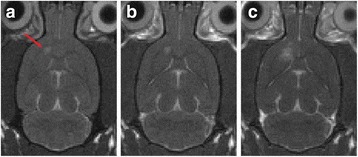
**a** T1weighted image before contrast administration. The *red arrow* indicates the tumour. **b** No difference in enhancement of the tumour is observed after contrast administration (SI=0.4%). **c** After focused ultrasound-mediated blood–brain barrier disruption, the tumour enhances after contrast administration (SI=30.1%)

BBB disruption was successful in all sessions with an average SI% of 21.2% (range 4.5 – 77.6%). The mean SI% of two tumours before BBB disruption during the six treatment weeks were 0.4% and 0.6%, indicating that the tumours were not leaky before disruption (Fig. 16). In 33% (20/60) of the FUS-sessions, regions were present that were clearly more hypo-intense on post- than on pre-sonication T2*w images, suggesting hemorrhages. In the remaining 67% of the sessions, no or a small difference in hypo-intensity was observed.

In the FUS+antibody-group, 4/10 animals were classified as responders during the treatment period (week 5 to week 11) with an average growth rate of 0.010±0.007, compared to 0.043±0.013 for the non-responders. There was no difference in the average SI% of the responding rats (21.8%±16.7) and the non-responding rats (20.7%±9.7). None of the control or antibody-only animals were classified as responder. When the FUS+antibody-animals are grouped, no significant differences in mean growth rates were observed between the control, antibody-only and FUS+antibody animals for the treatment period, nor for the follow-up period.

High-resolution T2w imaging showed that the tumour was homogenous in almost all animals till week 13–15, when cystic and necrotic areas started to develop. The tumours showed also a heterogeneous appearance on H&E stained sections and the complete tumour was expressing the HER2-receptor in the examined animals.

**Conclusions**

In this study, we demonstrate that FUS-mediated BBB disruption in combination with antibody therapy can slow down the growth of brain metastasis from breast cancer. As the tumours were not leaky before BBB disruption and no difference in growth rates was observed in the antibody-only group, the disruption of the BBB is necessary for drug delivery to these brain metastasis. Interestingly, only part of the rats responded to the treatment, the other animals had the same growth rate as the control-group. This is in line with a previous study (Park et al. 2012, J. Control. Release), where antibody therapy was combined with FUS in a different brain metastasis model and in part of the animals a strong response was observed, while the other animals did not respond. We did not observe a difference in tumour volume at the start of the treatment, in HER2 expression on histopathology, or in contrast-enhancement on MR images between the responders and non-responders to explain this. Better understanding of why certain animals respond is needed and will help in translating this technique to the clinic.

### O23 Long term follow up of 6 essential tremor patients treated with MR-guided focused ultrasound thalamotomy

#### Michael Schwartz^1,2^, Yuexi Huang^2^, Nir Lipsman^1^, Jennifer Jain^2^, Martin Chapman^2^, Tejas Sankar^3^, Andres Lozano^1^, Kullervo Hynynen^2^

##### ^1^Surgery (Neurosurgery), University of Toronto, Toronto, Ontario, Canada; ^2^Sunnybrook Health Sciences Centre, Toronto, Ontario, Canada; ^3^Surgery (Neurosurgery), University of Alberta, Edmonton, Alberta, Canada

**Objectives**

To determine the factors influencing outcome after MR-guided focused ultrasound (MRgFUS) thalamotomy for essential tremor.

**Methods**

Between May 2012 and February 2013, 6 patients were treated with MRgFUS thalamotomy. The first 4 patients have been reported (Lipsman et al., Lancet Neurol. 2013 May; 12(5): 462–8). Prospective recording of data from preoperative screening to 3 month follow up according to an experimental protocol using the clinical rating scale for tremor (CRST), and then a 2 year follow up examination and assessment were done. Maximum temperature at the focus of sonication was determined and post-treatment day 1 MRI scans were reviewed for lesion size and location. A point derived by our current method of determining the probable location of the ventral intermediate nucleus (VIM) of the thalamus was taken as the starting point for each treated case and a deviation vector from that point to the centre of each lesion produced was plotted.

**Results**

For the 6 patients, the mean CRST A scale (rest, posture and action) for the treated hand and arm prior to treatment was 7.2. At 1 week post-treatment, the mean CRST A was 0.67. At 3 months, the mean CRST A was 0.83 and at 2 years approximately 3.33. (At 2 years, the CRST A for 2 patients was estimated from a narrative account). Mean lesion size, excluding one very large lesion with a volume 2.7 times the mean volume of the other 5 patients, was 90.2 mm^3^. Only 2 patients had no decline in thalamotomy effect. Of these, one had a relatively large lesion of 107.5 mm^3^, which was located at the predicted location of the VIM nucleus. The other patient’s lesion was located 2.4 mm lateral to the predicted VIM location. He had prominent ataxia following his treatment. This subsided by 3 months. The patient with the large lesion, treated for left hand tremor, had persistent tingling of the left side of his mouth, and his left thumb and 3 fingers but excellent early relief of tremor. Although there was some persisting reduction of tremor at 2 years, he could no longer write, nor drink without spilling. The centre of his lesion was 1.3 mm lateral and 0.3 mm posterior to the expected VIM location. In its superior-inferior dimension his lesion measured 9.4 mm. The maximum temperature achieved at treatment was 63°C, higher than the mean of 58°C. This decline may, in part, be due to the progress of his condition, with increasing tremor on his untreated side and the onset of tremor in both legs. Two of the patients with a decline in function had relatively small lesions.

**Conclusions**

Not all patients have lasting benefit from MRgFUS thalamotomy. The effect of relatively large lesions may be more durable, but lesioning temperatures of greater than 60°C should likely be avoided.

### O24 Skull bone marrow injury caused by MR-guided focused ultrasound for cerebral functional procedures

#### Michael Schwartz^1,2^, Robert Yeung^1^, Yuexi Huang^1^, Nir Lipsman^2^, Jennifer Jain^1^, Martin Chapman^1^, Andres Lozano^2^, Kullervo Hynynen^1^

##### ^1^Sunnybrook Health Sciences Centre, Toronto, Ontario, Canada; ^2^Surgery (Neurosurgery), University of Toronto, Toronto, Ontario, Canada

**Objectives**

To determine the factors that lead to bone marrow injury in the skulls of patients undergoing MRgFUS cerebral ablations for the treatment of movement disorders such as ventral intermediate (VIM) nucleus thalamotomy for tremor and pallidotomy for L-dopa induced dyskinesia (LID), and to follow the course of healing.

**Methods**

All patients undergoing functional cerebral ablations produced for the mitigation of movement disorders are followed with serial MRI scans according to study protocol to assess the evolution of the cerebral lesions. In one patient subjected to very high power sonication in the attempt to produce a pallidotomy for LID, skull lesions were noticed on a follow up MRI scan, produced 4 months after his MRgFUS procedure. A review of other treated patients is currently underway.

**Results**

**Fig. 17 (abstract O24).**
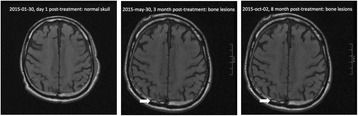
See text for description

Multiple ovoid lesions throughout the calvarium, new since the immediate post-treatment MRI scan done January 30, 2015, were seen on the MRI scan done May 12, 2015 (Fig. 17). Their appearance resembles that of bone infarcts. The MRI scan was repeated on October 2, 2015. Many of the ovoid lesions were still visible. This patient underwent sonication increasing to a maximum power of 1100 W for 31 seconds. During the procedure, the scalp and skull were constantly cooled with flowing degassed water at 13 °C. Despite this sonication, the target locus in the globus pallidus reached only 48 °C.

**Conclusions**

High power and duration sonication for functional cerebral lesions may cause injury to skull bone marrow. A review of all patients treated with MRgFUS for movement disorders is currently underway to determine whether there have been other cases, and to determine the threshold for bone marrow injury.

### O25 *In vitro* study using MR-guided focused pulsed ultrasound for destroying clots using thrombolytic drugs

#### Christakis Damianou^1^, Nikolaos Papadopoulos^2^

##### ^1^Cyprus University of Technology, Limassol, Cyprus; ^2^City University, London, United Kingdom

**Objectives**

In this paper an extensive study of using MR-guided focused pulsed ultrasound system is presented for the treatment of stroke using thrombolytic drugs in a model in vitro.

**Methods**

A single element spherically focused transducer of 4 cm diameter; focusing at 10 cm and operating at 1 MHz was used. The transducer was mounted in an MR compatible robotic system of 3 axes. The artery was modelled using a silicone tube. Tissue was modelled using agar-evaporation-silica gel. Coagulated blood was used to model thrombus. A thermocouple was placed in the thrombus in order to measure the thrombus temperature.

**Results**

The effect of power, presence of bubbles, temperature, presence of agar-evaporation milk-siligal gel, time of sonication, pulse repetition frequency, presence of standing waves, flow velocity were investigated. The goal was to maintain a temperature increase of less than 1°C during the application of pulsed ultrasound (called safe temperature). With the application of ultrasound alone or thrombolytic drug alone there was no notable destruction of the thrombus.

**Conclusions**

With the combination of ultrasound and thrombolytic drugs sufficient destruction occurred after 30 min, but the rate of destruction of thrombus (mL/min) is considered low. Thus, the clinical use of focused ultrasound for sonothrombolysis despite the full parametric study that we performed is considered pessimistic.

### O26 Low frequency in-vivo cavitation mapping

#### Alexander Volovick^1^, Javier Grinfeld^1^, Yoav Levy^1^, Omer Brokman^1^, Eyal Zadicario^1^, Ori Brenner^2^, David Castel^3^

##### ^1^INSIGHTEC, Tirat Carmel, Israel, ^2^Weizmann Institute, Rehovot, Israel, ^3^Sheba Medical Center, Ramat Gan, Israel

**Objectives**

Current trans-cranial Magnetic Resonance guided Focused Ultrasound Surgery (tcMRgFUS) treatments are limited to the targets that are located at the centre of the skull. Superficial and peripheral targets are prone to lower acoustic intensity reaching the focal point due to the geometry of the skull and the ultrasound transversal through it. A possible solution for this problem is reduction of the transmitting frequency. Reducing the frequency is a trade-off between higher skull penetration and lower tissue absorbing. In addition, lower frequency has a lower threshold to induce cavitation. Previously it was reported ([1]) that the transient cavitation threshold for the muscle tissue acts linearly with frequency as where P_th_ is the cavitation pressure threshold and f is the transmitting frequency. However this threshold was not reported to cause any unintentional pathological damage. In the presented work, different levels of acoustic intensities resulting in various occurrences of cavitation were applied to an in-vivo pig brain model, reaching various temperature levels and causing various pathological effects.

**Methods**

36 subjects underwent craniotomy and were sonicated using various ultrasonic parameters (ExAblate Neuro low frequency system, 220 Khz, by INSIGHTEC, Ltd). The temperature rise was measured using MR thermometry (1.5T GE HDx MRI by GE Healthcare, with an integrated Head Coil, by InSighTec) and cavitation signal was measured and recorded using integrated hydrophones. 20 subjects underwent 2 weeks follow up with post procedural MR imaging one, five, seven and fourteen days after the procedure. The brains were harvested, fixed in formalin and sliced to 3 mm slices. Macro-pathological slices for randomly selected subjects were also performed.

**Results**

**Fig. 18 (abstract O26).**
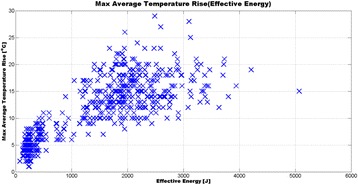
Maximal average temperature as a function of effective energy. The graph is based on the data of 502 sonications

**Fig. 19 (abstract O26).**
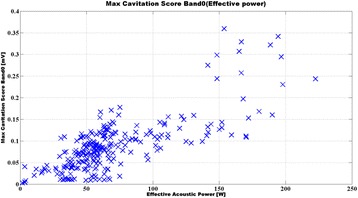
Maximal cavitation activity as a function of effective power. The graph is based on the data from 253 sonications

**Fig. 20 (abstract O26).**
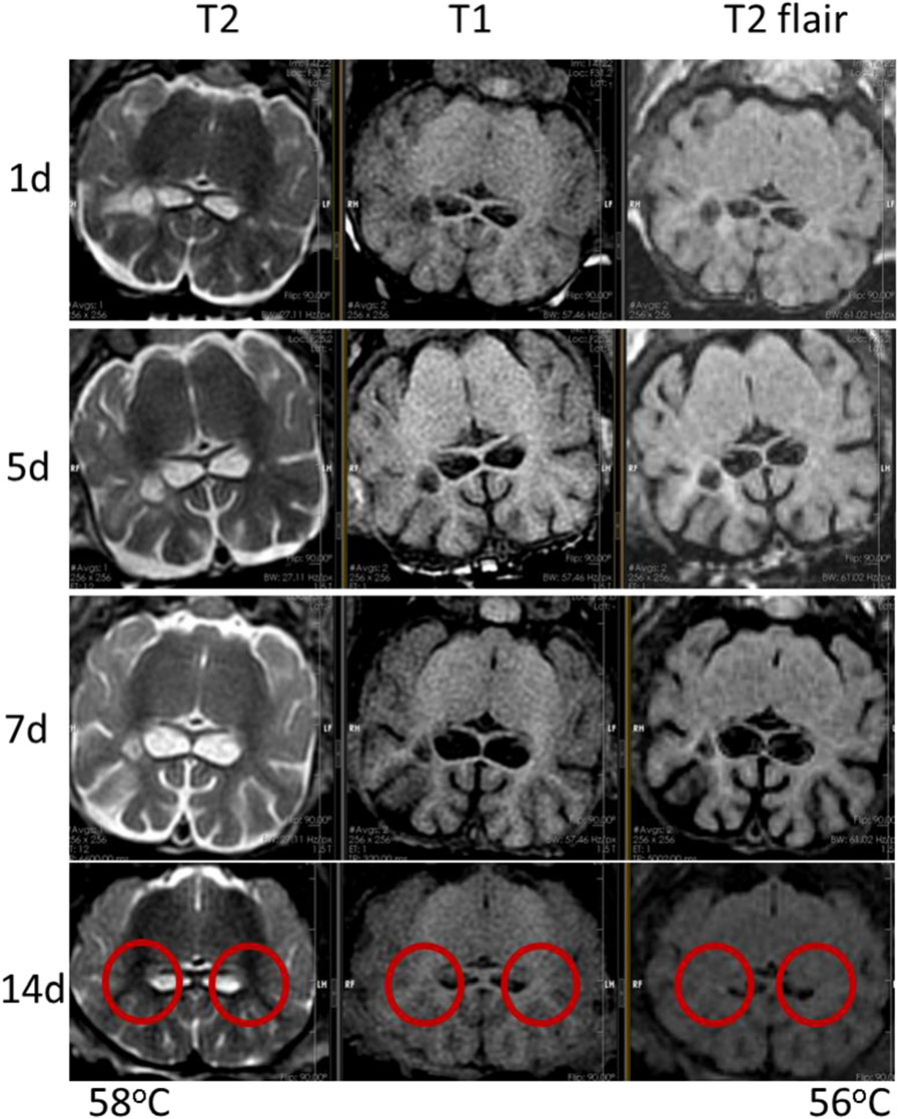
An example for two weeks follow up imaging for T2, T1 and T2 flair images presented. Two lesions are visible on the 1 and 5 days follow up images, single lesion is seen for the 7 days follow up and no lesion is visible at the 14 days follow up imaging. The lesions locations are marked by red circles at the 14 days follow up images in order not to hide the lesion and edemic tissue for the earlier follow up images. The lesion on the left was produced by the sonication reaching 58°C, whereas the lesion on the right reached temperature of 56°C

**Fig. 21 (abstract O26).**
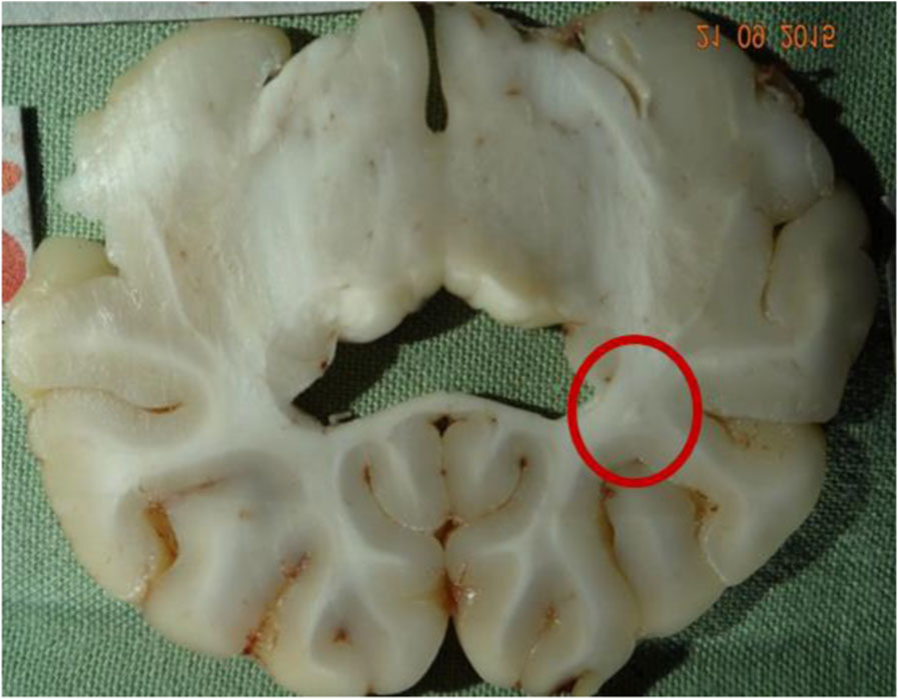
Macro pathology of the slides presented on Fig. 20. Only one lesion was detected

Figure 18 represents the graph of temperature rise as a function of applied energy. Figure 19 represents the graph of cavitation activity as a function of maximal acoustic power. Figure 20 shows a follow up imaging for 2 lesions performed on a single subject, Fig. 21 is a macro pathology slide for the subject presented in Fig. 20. In general the temperature rise grew linearly with the applied acoustic energy; the cavitation activity was linearly dependent to the maximum applied acoustic power. For sonications that were in the central cavitation activity area as presented on Fig. 19 the lesions were well defined and increased in size till the 5^th^ day follow up and then reduced in size becoming scarf tissue as presented on Figs. 20 and 21. Macro pathology revealed that tissue rapture is seen on the micro level; however it is well defined and limited within the lesion area.

**Conclusions**

Cavitation threshold levels that were observed in brain agree with the levels reported in literature. Additional levels of cavitation were observed and associated with effects on tissue as seen in MRI and histology. The data collected suggests cavitation levels that can be applied while keeping lesioning effect to the confined area and avoiding haemorrhages in tissue. Integrating a real time control over the level of cavitation and keeping the level below the safety threshold results in safe and effective tissue ablation in brain.

### O27 Real-time, transcranial passive cavitation mapping for monitoring of the focused ultrasound-induced blood–brain barrier opening in primates

#### Shih-Ying Wu^1^, Julien Grondin^1^, Wenlan Zheng^1^, Marc Heidmann^1^, Maria Eleni Karakatsani^1^, Carlos J. Sierra Sánchez^1^, Vincent Ferrera^3^, Elisa E. Konofagou^1, 2^

##### ^1^Biomedical Engineering, Columbia University, New York, New York, USA; ^2^Radiology, Columbia University, New York, New York, USA; ^3^Neuroscience, Columbia University, New York, New York, USA

**Objectives**

Real-time cavitation monitoring during the focused ultrasound (FUS) induced blood–brain barrier (BBB) opening is crucial in assessing and controlling the BBB opening outcomes and safety. Currently, passive cavitation detection using a single-element detector for quantification of the stable and inertial cavitation doses has shown good correlation with the opening volume and the molecular delivery efficiency in nonhuman primates (NHPs). However, an off-line magnetic resonance imaging (MRI) is required for confirming the targeting or opening area in the brain after the FUS procedure. It is therefore essential to develop transcranial cavitation mapping in providing the spatial distribution of cavitation intensity in real time in order to precisely assess and control the BBB opening outcomes with accurate targeting during the procedure. The aim of this study is to develop real-time cavitation mapping using time-exposure acoustics and passive sparse matrix beamforming with the performance evaluated in both the in vitro primate skull (NHP and human) experiments and the in vivo NHP experiments during BBB opening.

**Methods**

Similar to array-based passive cavitation detectors, a linear probe (L7-4, Philips) with a programmable data acquisition system (Vantage, Verasonics) was used to acquire cavitation signal passively during the sonication (frequency: 0.5 MHz, pulse length: 10 ms, PRF: 5 Hz, pressure: 150–600 kPa) with in-house made microbubbles (lipid-shelled and monodisperse with a diameter of 4–5 μm) and a single-element FUS transducer with a coaxially aligned flat-band hydrophone as a single-element passive cavitation detector. Time-exposure acoustics for an integration of a series of passive cavitation images over the exposure time reconstructed by dynamic receive beamforming using sparse matrix calculation in graphic processing unit (GPU; Tesla K40, NVIDIA) were developed for the reconstruction of passive cavitation maps in real time. For the in vitro experimental setup, a phantom with a channel of 4 mm in diameter and an infusion pump was used to mimic the vessel with microbubble circulation (a concentration of 2×10^5^ bubbles/mL with a flow rate of 1 mL/min), and the FUS transducer and the linear array were both focused at the channel orthogonally. B-mode imaging with the linear array was performed before acquiring the passive cavitation signal in order to confirm the alignment of both the linear array and the FUS transducer to the channel. Three sets of passive cavitation maps were acquired using this in vitro setup: 1) without the skull, 2) with the NHP skull and 3) with the human skull placed between the phantom and the linear probe. The effects of acoustic pressure, exposure time and aperture size to the intensity and focal size of the cavitation maps were all evaluated, as well as the computational cost in GPU and the sensitivity through the skull. Furthermore, the in vivo cavitation maps were acquired during the sonication for BBB opening in NHPs (duration: 2 min) and compared with the BBB opening outcomes in the MRI.

**Results**

The results of the in vitro experiments showed that the cavitation location corresponded to the microbubble disruption in B-mode images, and both the intensity and the focal area of the cavitation maps increased with pressure. Increasing the exposure time eliminated the interference outside of the focus and enhanced the focalization by minimizing the focal area, while both the intensity and the focal area reached a plateau at the exposure time of 62.5 μs. The focal size especially in the axial direction increased with decreasing aperture size, suggesting an improved focalization by using a larger aperture size of the array-based PCD. The computational time for the exposure time of 62.5 μs was 9.5 s, which can be decreased to 0.27 s in achieving real-time monitoring by decreasing the exposure time to 1.44 μs. By placing the skull, it was found that the cavitation signals were still detectable through the NHP and human skulls at 300 kPa and 600 kPa with the mapping system, respectively. For the in vivo experiments, the BBB opening in NHP were successfully monitored with passive cavitation mapping targeting at the caudate and the hippocampus, the deep subcortical structure in the brain.

**Conclusions**

A real-time cavitation mapping technique using time-exposure acoustics and passive sparse matrix beamforming has been developed with the performance and the sensitivity through the primate skull evaluated, and was used for monitoring of the BBB opening in NHPs. This novel transcranial monitoring technique providing both the spatial and intensity information of cavitation in real time during the FUS procedure is promising in assessing and controlling the targeting, treatment efficacy, and safety precisely.

### O28 Blood brain barrier opening using focused ultrasound for the reduction of amyloid beta plaques in synergy with antibodies in a rabbit model fed with high cholesterol diet

#### Christakis Damianou, Marinos Yiannakou

##### Cyprus University of Technology, Limassol, Cyprus

**Objectives**

An animal model that creates Amyloid beta (Ab) plaques in the brain was implemented by delivering high cholesterol diet in rabbits for 3 months. The goal was to reduce the plaques using focused ultrasound (FUS) in combination with external antibodies.

**Methods**

A single spherically focused transducer was used which operates at 1 MHz, has focal length of 10 cm and diameter of 3 cm. The rabbit was placed in a custom made MRI compatible positioning device. Theoflavin staining was used in order to measure the plaque load at the end of each experiment.

**Results**

Using pulse FUS the blood brain barrier (BBB) was opened repeatedly up to 5 times at three day intervals. The opening of the BBB disruption was imaged using contrast-enhanced T1-weighted fast spin echo. By increasing the number of sessions, the number of plaques decreases (both for internal and external antibodies). With the use of FUS only (internal antibodies) the drop of average number of plaques/mm^2^ was reduced by 20% (in 5 sessions). The effect of external antibodies was more drastic. With 5 BBB sessions the average number of plaques/mm^2^ was reduced by 60%.

**Conclusions**

This study demonstrated that by opening the BBB, it will be possible to deliver internal and external antibodies to the brain, which eliminates Alzheimer disease (AD) plaques. More important by opening the BBB frequently (up to 5 times in this study) the reduction in the number of plaques is enhanced. Therefore FUS has the potentials to be used non-invasively for the treatment of AD.

### O29 Correlation between down-regulation of p-glycoprotein and blood–brain barrier disruption in rat brain by mri-guided focused ultrasound and microbubbles

#### HongSeok Cho^1^, Hwayoun Lee^1^, Mun Han^2^, Jong-Ryul Choi^1^, Taekwan Lee^1^, Sanghyun Ahn^1^, Yongmin Chang^2^, Juyoung Park^1^

##### ^1^Daegu-Gyeongbuk Medical Innovation Foundation, Daegu, Republic of Korea; ^2^Kyungpook National University, Daegu, Korea (the Republic of )

**Objectives**

Blood–brain barrier (BBB) is composed of both physical barrier with tight junctions and functional barrier with active efflux transporters. Mechanism of the functional barrier is mediated by P-glycoprotein (P-gp) and breast cancer resistance protein (BCRP) in brain endothelial cells. Past studies have shown that focused ultrasound (FUS) combined with microbubbles can disrupt the physical barrier of the BBB by exerting mechanical stress on the tight junctions; however, no study was performed to investigate the impact of FUS and microbubbles on the functional barrier of the BBB. Therefore, this study investigated the impact of BBB disruption induced by FUS and microbubbles on the expression of P-gp. We also investigated correlation between magnitude of the BBB opening and the down-regulation of P-gp.

**Methods**

A single target region was sonicated (0.5~0.7 MPa) transracially in one hemisphere in 3 rats using a 1 MHz FUS transducer; the other hemisphere served as a control. For the BBB disruption, 10 ms bursts were applied at 1Hz pulse repetition frequency (PRF) for 120 s and combined with IV injection of a microbubble ultrasound contrast agent (Definity 0.1 ml/kg). An MR contrast agent (Magnevist 0.4 mM/kg) and Evans Blue (0.15 ml/kg) were injected immediately after the sonication to indicate area of the BBB disruption in MR image and fluorescence spectroscopy, respectively. In order to measure the P-gp expression using a confocal fluorescence microscopy, the brains were fixed after perfusion and then stained immunohistochemically with a monoclonal antibody (C219) which reacts with a P-gp epitope.

**Results**

**Fig. 22 (abstract O29).**
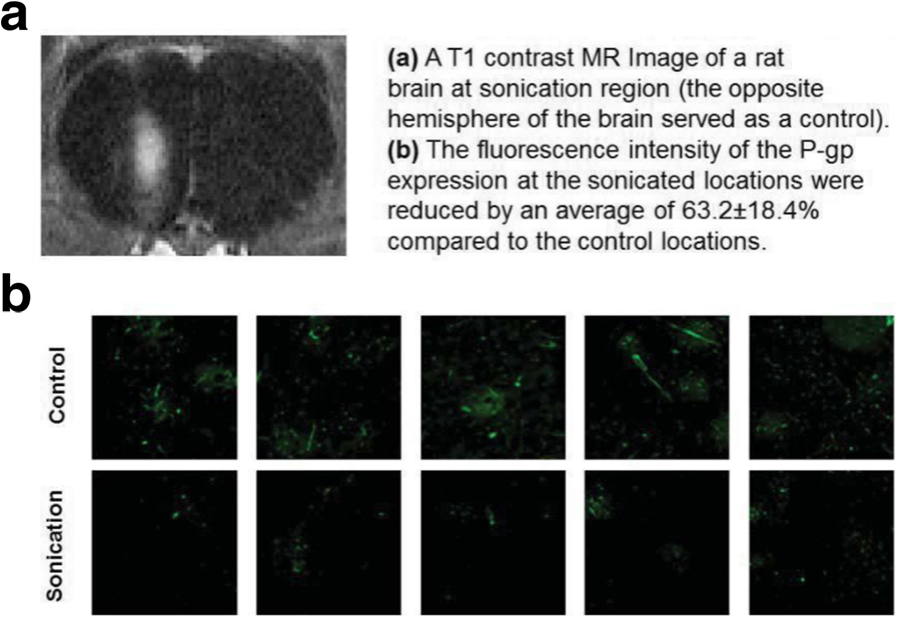
**a** A T1 contrast MR Image of a rat brain at sonification region (the opposite hemisphere of the brain served as control). **b** The fluorescence intensity of the P-gp expression at the soicated locations were reduced by an average of 63.2±18.4% compared to the control locations

A T1 contrast enhanced MR image and Evans Blue fluorescent intensity at the sonicated regions indicated localized BBB disruption (Fig. 22). Both the MR contrast intensity and the Evans Blue fluorescent intensity were significantly increased in the targeted regions compared to the control regions (p<0.001). The fluorescence intensity of the P-gp expression at the confirmed locations of the BBB disruption was reduced by an average of 63.2±18.4% compared to the control area in all three rats. From the three sonicated regions, a total of 31 locations were selected and the P-gp fluorescence intensities were measured to observe the correlation between the degree of the BBB opening and the P-gp expression. Both the Evans Blue intensity and the MR contrast intensity were significantly correlated with the P-gp expression intensity (r=−0.72, p<0.001; r=−0.62, p<0.001, respectively). Histologic analysis on the sonicated region of the brain tissue revealed no apparent damage in the endothelial cells, and no significant amount of extravasated red blood cells was observed.

**Conclusions**

This study demonstrates that the BBB disruption induced by FUS and microbubbles reduces the expression of P-gp, and the level of the down-regulation of P-gp is significantly correlated with the magnitude of the BBB opening. These results suggest FUS + microbubble as a promising mean for the brain drug delivery through the BBB by overcoming both the physical and the functional barrier of the BBB.

### O30 MR-guided focused ultrasound blood–brain barrier disruption through an intact human skull in a rat model using a clinical body system

#### Nicholas Ellens^1^, Ari Partanen^1,2^, Keyvan Farahani^1,3^, Raag Airan^1^

##### ^1^Radiology, Johns Hopkins University, Baltimore, Maryland, USA; ^2^Philips, Andover, Massachusetts, USA; ^3^National Cancer Institute, Bethesda, Maryland, USA

**Objectives**

MR-guided focused ultrasound (MRgFUS) can be used for a large range of non-invasive therapies using mechanical and thermal mechanisms. For instance, it has been demonstrated that a combination of low intensity focused ultrasound and synthetic microbubble scan be used to safely, locally, and transiently disrupt the blood–brain barrier (BBB)[1].

Typically, transcranial MRgFUS thermal ablation requires large aperture phased arrays with many elements operating at lower frequencies and higher powers than body systems due to high transmission losses through the skull. However, in low pressure applications at low duty cycles, such as BBB disruption or neuromodulation, it may be feasible to use 'body' systems that have smaller aperture transducers with fewer elements [2]. The objectives of this study were to use a clinical body MRgFUS system to:

1. Quantify the ultrasound transmission and focal distortion through a human cranium at different orientations and in different focus locations inside the skull to assess the possible treatment envelope with such a device.
2. Demonstrate the ability to disrupt the blood–brain barrier (BBB) in rat neurovasculature through a human cranium.

**Methods**

**Fig. 23 (abstract O30).**
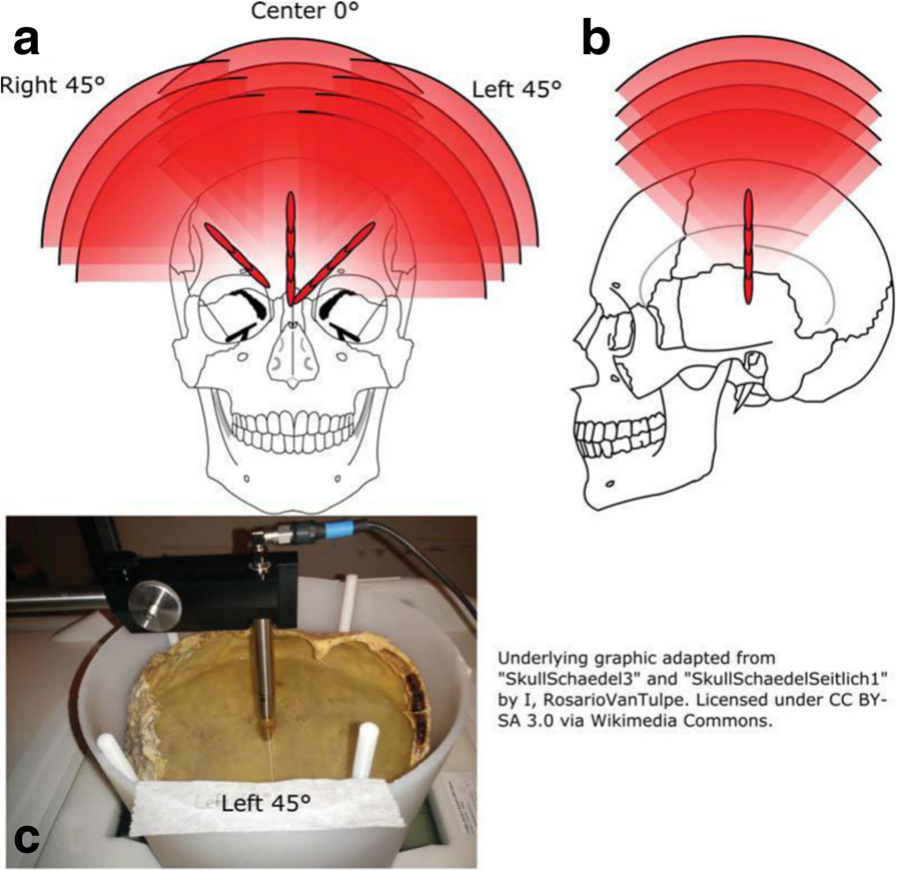
**a** and **b**: Coronal and sagittal representations of the locations examined. **c**: Picture of the hydrophone and skull arrangement for one of the 'Left 45°' orientations. The transducer is below the table

A clean, degassed human cranium (gift of Dr. Quiñones-Hinojosa, JHU Neurosurgery) was mounted in degassed water above a 256-element phased-array transducer (14 cm focal length) of a clinical body MRgFUS system (Sonalleve V2, Philips, Vantaa, Finland). Hydrophone measurements were made with a lipstick hydrophone (Onda Corp., Sunnyvale, USA) mounted to a 3D stage, both in water and through the human skull in 11 locations (three orientations, 3–4 depths at each, ranging between the centre of the cranium and 2 cm from the skull surface). 40-cycle sonications at 1 MHz were applied at different acoustic powers ranging from 5 to 20 W (water) and 20 to 500 W (trans-skull). In each location, the full-width at half-maximum (FWHM) of the ultrasound focal point (in three dimensions) and its peak negative pressure were measured. This setup and the locations sampled are shown in Fig. 23.

**Results**

**Fig. 24 (abstract O30).**
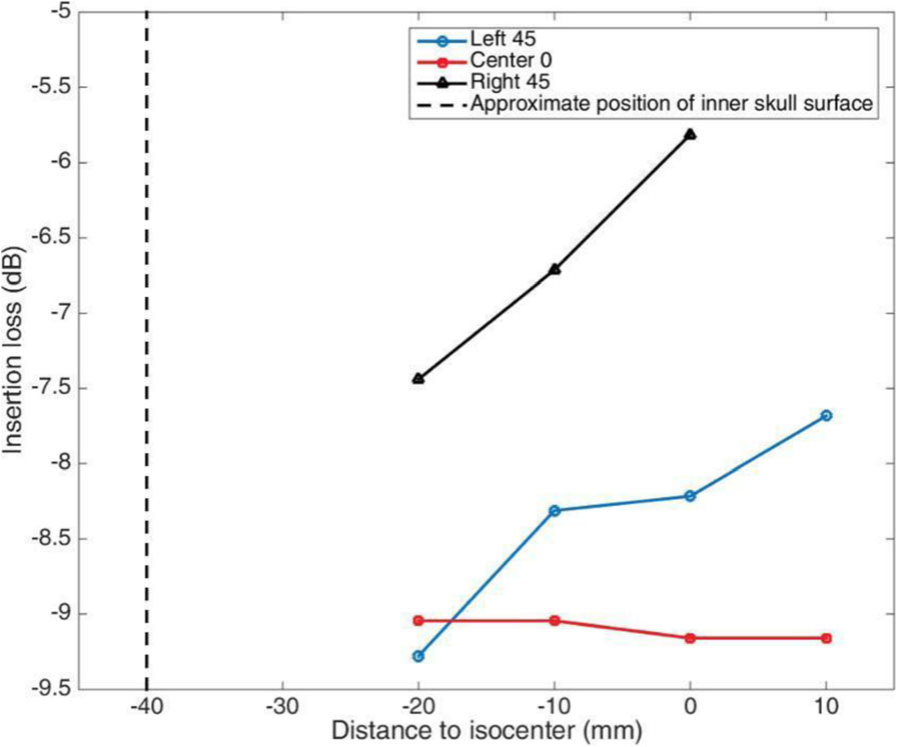
Insertion losses for different sonication orientations and depths

Hydrophone measurements demonstrated a great deal of variation in ultrasound transmission with changing transducer/skull incidence angle and skull thickness. The pressure attenuation ranged from −5.8 dB to −9.3 dB (mean +/−standard deviation, −8 dB +/− 1 dB), and the FWHM varied between 1.83 mm and 3.79 mm (2.2 mm +/− 0.3 mm in the anterior/posterior direction, 2.7 +/− 0.7 mm in the left/right direction). The insertion losses as a function of depth at different orientations are shown in Fig. 24. The dorsal midline skull (with the ultrasound beam passing through the sagittal and coronal sutures) was thicker than either of the angled approaches (passing through the parietal and temporal bones) and experienced a greater insertion loss for most sonication target depths. For both angled approaches, the insertion loss decreased as the focus was moved away from the skull surface. For the centered approach, the insertion loss increased slightly as the focus moved closer to the skull surface. It appears that the intervening skull thickness dominates the attenuation, though the angle of incidence seen by transducer elements also affects transmission.

**Fig. 25 (abstract O30).**
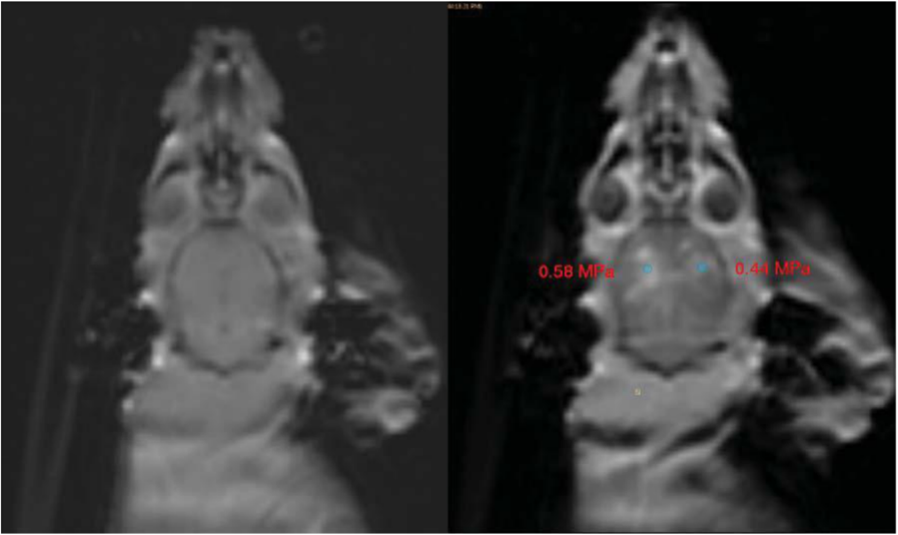
Baseline T1-weighted coronal image, *left*, and post-contrast post-BBB disruption image, *right*, showing two locations of BBB disruption with the estimated in situ pressures labelled

**Fig. 26 (abstract O30).**
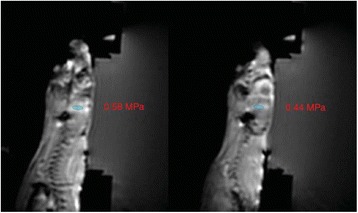
Sagittal images showing columns of BBB disruption produced by the labelled estimated in situ pressures. Ultrasound propagation is from the right to the left of these images.

Gadolinium enhancement in the brain on post-sonication T1-weighted MRI indicated successful BBB disruption. Disruption accuracy is shown in Figs. 25 and 26. For sonications of sufficient pressure to produce BBB disruption, the region of gadolinium enhancement did not appear to be shifted at all from the desired target. For higher estimated in situ pressures (>0.55 MPa), the disruption region was larger and less uniform, as evident in the 0.58 MPa point in Fig. 25. Even without element-by-element refocusing, the focusing quality and targeting precision appeared to be adequate. Post-experiment, there were no signs of gross damage to the animals to suggest significant off-target sonication.

**Conclusions**

Transcranial FUS focal point distortion was minimal despite the lack of element-by-element transducer refocusing. The clinical MRgFUS body transducer and driving electronics had sufficient power and aperture to generate the in situ pressures for BBB disruption through a human skull, using a variety of clinically practical approaches and patient orientations.

Though transcranial thermal ablation typically requires high acoustic powers, a lower operating frequency, and large aperture arrays with high number of elements, this study demonstrates that a clinical body MRgFUS system with a smaller transducer may be a safe and feasible alternative for non-invasive BBB disruption and other low-pressure therapeutic ultrasound transcranial applications, potentially offering a wide treatment envelope.

**References**

[1] Hynynen, K., *et al.* (2001). Radiology, 220(3), pp. 640–646.

[2] Airan, R. D., *et al.* (2015). 23^rd^ ISMRM.

### O31 Interim results from a phase 1 clinical trial to disrupt the blood–brain barrier by pulsed ultrasound

#### Alexandre Carpentier^3,4^, Michael Canney^1^, Alexandre Vignot^1^, Cyril Lafon^2^, Jean-Yves Chapelon^2^, Jean-yves Delattre^4,5^, Ahmed Idbaih^5^

##### ^1^CarThera, Lyon, France; ^2^INSERM, U1032, LabTau, Lyon, France; ^3^Assistance Publique Hopitaux de Paris, Hopital de la Pitie Salpetriere, Department of Neurosurgery, Paris, Paris, France; ^4^Universite Paris, UPMC, Paris, France; ^5^Assistance Publique Hopitaux de Paris, Hopital de la Pitie Salpetriere, Department of Neuro-Oncology, Paris, France

**Objectives**

Pulsed ultrasound, coupled with peripheral injection of microbubbles, has been shown in pre-clinical studies to be an effective method for enhancing the delivery of chemotherapy to the brain. In this work, an intra-skull implantable ultrasound device, SonoCloud®, was developed for temporarily disrupting the BBB. The device was implanted in patients with recurrent glioblastoma (GBM) undergoing systemic carboplatin chemotherapy in a Phase 1 clinical trial and the safety of repeated BBB disruption was assessed.

**Methods**

A Phase 1 clinical trial began in July 2014 at a single centre at the Hospital Pitie Salpetriere in Paris, France. Patients with recurrent GBM with an enhancing volume of less than 35 mm in diameter were included in the trial. Participants were implanted with a 11.5-mm diameter biocompatible 1 MHz ultrasound transducer, which was fixed into the skull bone thickness. The device was either implanted during a regular surgical resection of the enhancing region or during a unique ambulatory procedure under local anaesthesia. Once a month, the device was connected to an external radiofrequency generator using a transdermal needle, and patients received a two minute pulsed ultrasound exposure in combination with systemic administration of an ultrasound contrast agent (7 min mean total duration procedure). BBB disruption was assessed immediately after sonications using dynamic T1-weighted imaging with a gadolinium based MR contrast agent. Systemic intravenous injection of carboplatin chemotherapy was delivered immediately following MR imaging. Patients followed a progression of ultrasound dose in which the pressure was increased from 0.5 to 0.8 MPa throughout the course of the study.

**Results**

As of July 2015, eleven patients had been included in the study with a total of 25 BBB disruption procedures performed. No adverse effects were observed in patients treated. BBB opening was clearly observed in 12/25 treatments on contrast-enhanced T1w imaging. The procedure was safely tolerated in all patients. No evidence of acute haemorrhage, petechia, ischemia or oedema was observed in post-sonication SWAN T2*, Diffusion or FLAIR MRI sequences.

**Conclusions**

The BBB was safely opened by pulsed ultrasound using an implantable ultrasound device in patients with recurrent glioblastoma. Additional follow up and recruitment will be used to further evaluate the safety and potential efficacy of such an approach.

**Acknowledgments**

*Work supported by CarThera and the Hospital Pitie Salpetriere.*

### O32 Investigation of temperature dependent changes in MR signal intensity, t1 and t2* in cortical bone

#### Henrik Odéen^2^, Bradley Bolster^1^, Eun Kee Jeong^2^, Dennis L. Parker^2^

##### ^1^Siemens Healthcare, Salt Lake City, Utah, USA; ^2^Radiology, University of Utah, Salt Lake City, Utah, USA

**Objectives**

For MR guided focused ultrasound treatments in or close to bone, such as for transcranial applications focusing through the intact skull bone and treatments of bone metastases, significant heating can occur in the bone due to its high ultrasound absorption. MR imaging of bone is in general challenging due to the short T2 relaxation time of bone. For MR temperature imaging the short T2 also severely decreases the accuracy that can be achieved with the standard proton resonance frequency shift method. Instead researchers have investigated the temperature dependence of the MR signal intensity (SI) and T1 relaxation time for temperature monitoring (1–4). Miller (1) and Fielden (3) et al. showed that the SI from cortical bone decreases with increasing temperature using ultrashort echo time (UTE) pulse sequences, and Ramsay et al. (2) found that, contrary to what Miller and Fielden observed, the SI increases with increasing temperature using a short TE gradient recalled echo (GRE) pulse sequence. Han et al. (4) further showed that T1 increases with temperature, also using UTE.

In this work we investigate the temperature dependence of the SI (dSI/dT) and the T1 and T2* relaxation times (dT1/dT and dT2*/dT, respectively) using a 3D UTE pulse sequence to investigate which parameter has the highest sensitivity to temperature change.

**Methods**

All imaging was performed on a 3T MRI scanner (MAGNETOM PrismaFit, Siemens Healthcare, Erlangen, Germany) using a 3D UTE pulse sequence. The sequence utilizes radial, ramped sampling of k-space in 3D starting at the k-space centre after a 80 μs non-selective hard RF pulse, allowing TEs down to 50 μs. T2* was measured by performing an exponential fit to data acquired at TE = 50, 90, 130, 170, and 250 μs (other scan parameters are listed in Table 1). The 50 μs TE was also used for dSI/dT calculations. T1 was measured using the variable flip angle (VFA) method (5) with TR = 11 ms and FA = 8 and 36° (other scan parameters are listed in Table 1).

**Fig. 27 (abstract O32).**
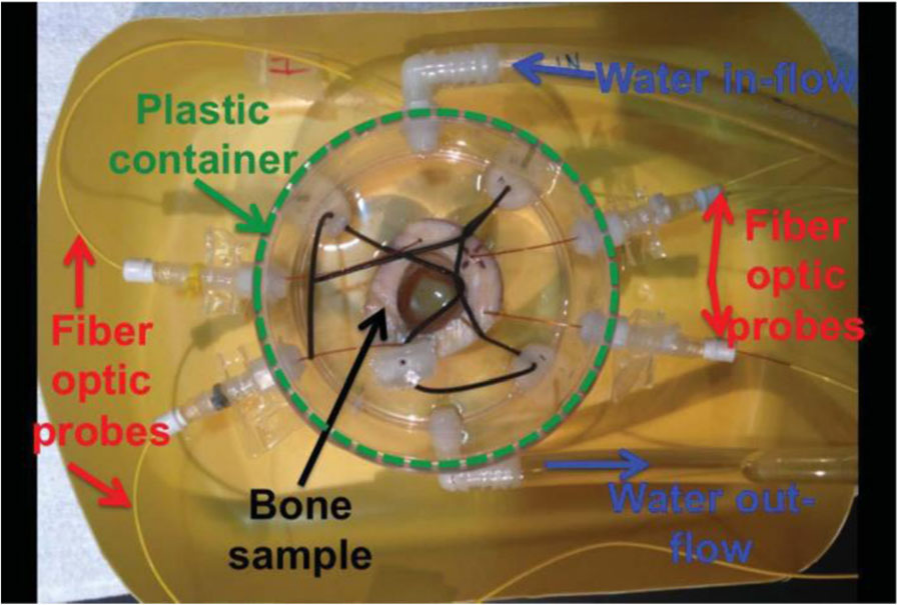
Scan setup. A ~4 cm long bone sample was placed in a phantom holder that allowed water circulation to homogenously heat the bone. 4 fiber optic probes were used; 1 in the water and 3 in the bone sample.

An approximately 4-cm long bovine femur (marrow and connective tissue removed) was placed in a phantom holder that allowed heated water to circulate around the bone, Fig. 27. One fiber optic probe measured the water temperature, and three probes were inserted in 1-mm diameter, 2-cm deep, holes drilled in the bone to measure the temperature of the bone. The water was warmed to ~22, 35, 50, and 65°C and data was collected when all 4 probes measured within 1°C. The whole setup was places in a 20-channel RF head coil for signal detection.

**Results**

**Fig. 28 (abstract O32).**
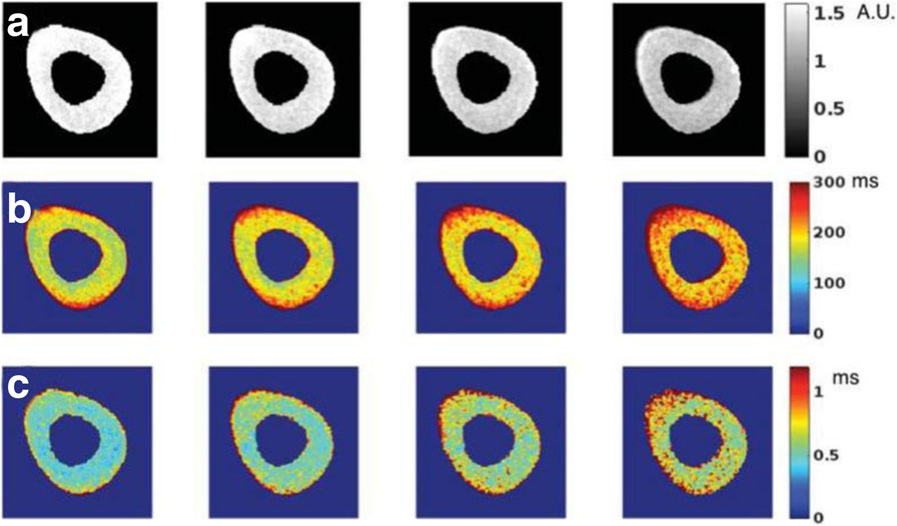
2D maps of SI and relaxation times for the 4 different temperatures (~22, 35, 50, and 65 °C), **a** SI, **b** T1, and **c** T2*

**Fig. 29 (abstract O32).**
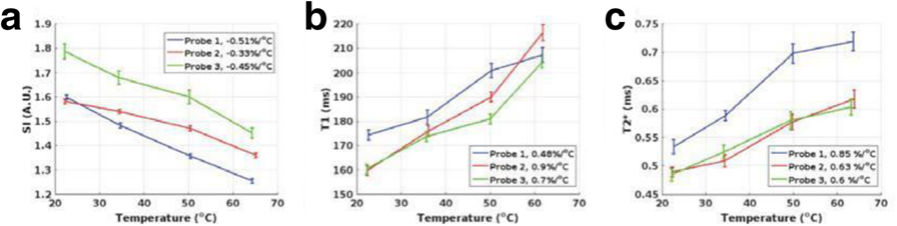
Changes versus temperature for **a** SI, **b** T1, and **c** T2*. Mean and standard error value from a 9x9 voxel ROI close to each probe is shown

Figure 28 shows 2D maps of SI and T1 and T2* relaxation times for the 4 different temperatures. Mean and standard error values from a 9x9 ROI close to each probe is shown in Fig. 29, together with calculated changes in %/°C. A decrease in SI of 0.3-0.5%/°C, and increases in T1 and T2* of 0.5-0.9%/°C and 0.6-0.9%/°C, respectively, was observed. Using the temperature dependent spoiled GRE signal equation (6) and the observed values for dT1/dT and dT2*/dT, dSI/dT can be closely predicted.

**Conclusions**

The decrease in SI is in accordance with previously published results by Miller and Fielden. The measured change in T1 using UTE agrees well with the 0.6%/°C reported by Han, although we measured higher absolute T1 values (~160-175 ms at 25°C, compared to ~115-125 ms as reported by Han).

The effect of T1 and T2* on SI are counter-acting each other (both increasing), which reduces the sensitivity of dSI/dT. This may suggest that dT1/dT and dT2*/dT are more suitable candidates for bone MR thermometry, and Fig. 29 also shows higher sensitivity for relaxation times that for SI. However, it should be noted that SI can be detected from a single image, whereas T1 and T2* measurements utilize two or more images, therefor resulting in longer scan times.

Future studies will acquire data with longer TEs (out to ~10 ms) to investigate the difference in temperature sensitivity for shorter and longer T2 components in the bone. These results will be compared to the increase in SI with temperature observed by Ramsay using a TE ≈ 1 ms. The temperature dependence of the long and short T2* components can be found by a multi-exponential fit. Flip angle mapping will also be implemented to improve the accuracy of the T1 measurements (7).

**Table 1 (abstract O32).** Scan parameters

	FOV (mm)	Res (mm)	TR (ms)	TE (ms)	FA (deg)	BW (Hz/px)	Scan time (s)	# views
UTE T1	160×160×160	1.0×1.0×1.0	11	0.05	8, 36	1008	167	15000
UTE T2*	160×160×160	1.0×1.0×1.0	6	0.05, 0.09, 0.13, 0.17, 0.25	15	1008	122	20000

*FOV* Field on view, *Res* Resolution, *TR* Repetition time, *TE* Echo time, *FA* Flip angle, *BW* Bandwidth (read out), # views – number of radial views/rays acquired

### O33 Spatially-segmented MRI brain and water bath reconstruction for undersampled transcranial mr-guided focused ultrasound thermometry

#### Pooja Gaur^1^, Xue Feng^2^, Samuel Fielden^2^, Craig Meyer^2^, Beat Werner^3^, William Grissom^1^

##### ^1^Vanderbilt University, Nashville, Tennessee, USA; ^2^University of Virginia, Charlottesville, Virginia, USA; ^3^University Children's Hospital, Zurich, Switzerland

**Objectives**

**Fig. 30 (abstract O33).**
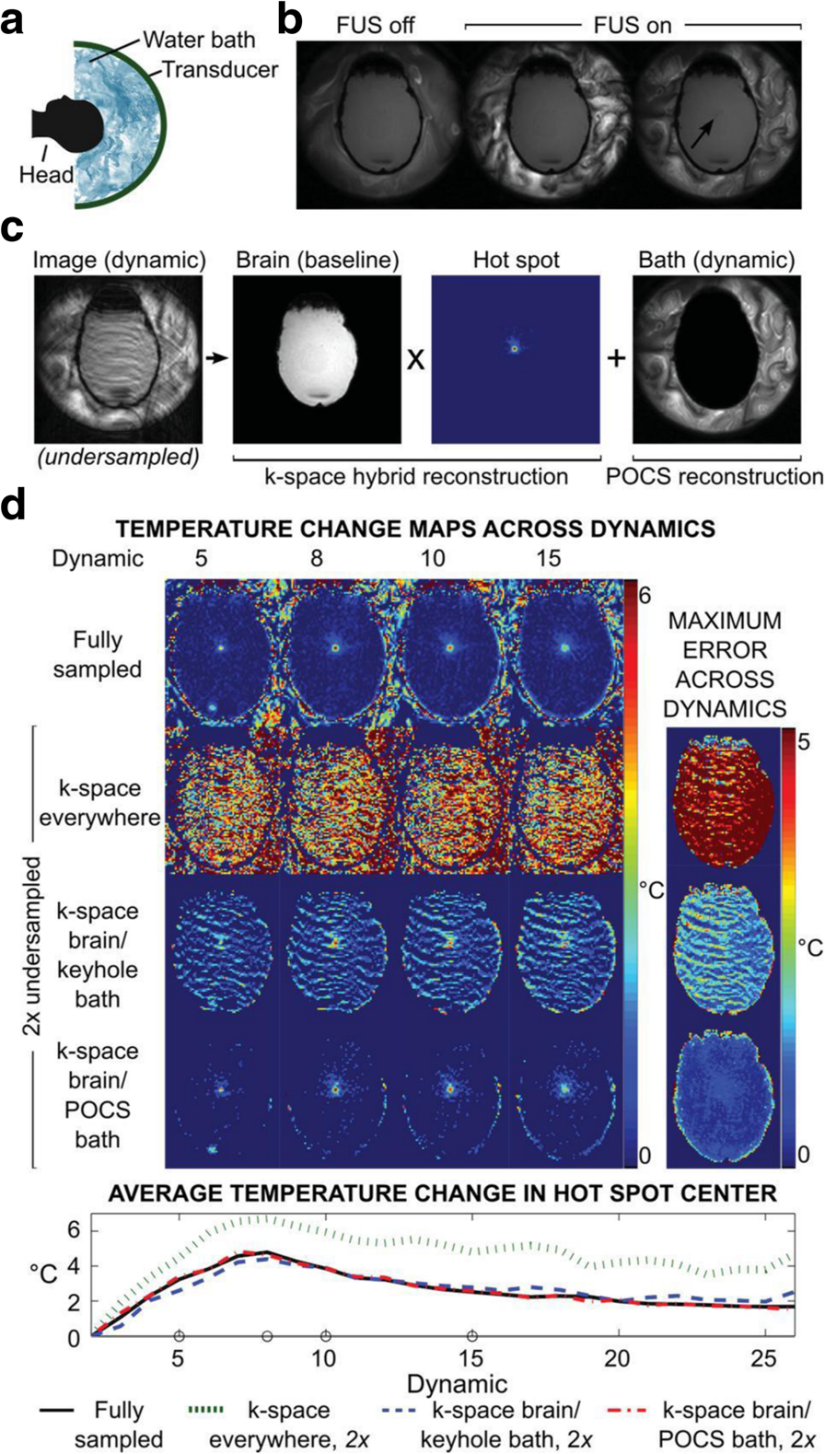
**a** During MRgFUS treatment, the patient’s head is immobilized in the transducer and circulating water bath. **b** The water bath signal varies significantly during a single focused ultrasound (FUS) sonication (*arrow* indicates sonication target). **c** In the proposed method, undersampled dynamic data are reconstructed using the k-space hybrid method in the brain and a POCS reconstruction in the bath. **d** Reconstructed temperature changes and maximum temperature errors in the brain with 2x undersampling. Temperature change averaged over the hot spot center is plotted at the bottom for each reconstruction. Circles on the x-axis indicate dynamics for which temperature maps are displayed above

MR-guided focused ultrasound (MRgFUS) brain systems deliver targeted thermal energy into the brain using a hemispheric array of transducers that surround the head with an intervening water bath (Fig. 30a). During treatment the localized heating (hot spot) is measured from a change in image phase between baseline (pre-treatment) and dynamic (during treatment) images. Accelerating temperature mapping by undersampling k-space is desirable to increase spatiotemporal resolution and coverage, but is difficult to do with parallel imaging since coils must be placed outside the transducer, far away from the head. Multiple groups have instead developed accelerated temperature mapping methods that exploit temporal correlations between baseline and dynamic images [1, 2]. However, circulation of the water bath to cool the skull causes dynamic signal changes that are not captured by baseline images (Fig. 30b), which breaks those correlations and results in artefacts throughout the temperature maps. We propose a spatially-segmented approach for reconstructing temperature maps in brain MRgFUS, in which we separately estimate a water bath image without a baseline, and a temperature map in the brain with a baseline. The method can estimate artefact-free temperature maps from undersampled data during brain MRgFUS treatments using a single receive coil.

**Methods**

Our iterative approach alternates between updating the parameters of a k-space hybrid signal model which is fit in the brain region of the image [1], and a baseline-free estimate of the water bath image. The fitting of k-space hybrid brain model results in a phase drift-corrected brain image without the temperature phase shift and a sparse temperature phase shift map. An algorithm to fit the model is described in [1]. The water bath is reconstructed using a POCS algorithm that alternately enforces data consistency, consistency with a water bath support mask (brain and water bath masks are obtained from a baseline image), and sparsity in the Coiflet domain using soft thresholding [3]. Figure 30c illustrates the overall undersampled dynamic image model.

To test the method, a gel-filled human skull phantom was sonicated by an InSighTec ExAblate Neuro 4000 transcranial MRgFUS system (InSighTec Ltd, Haifa, Israel) while imaging with a GE 3T MR750 scanner (GE Healthcare, Waukeshaw, WI). 27 single-slice 2DFT gradient echo images were collected with the body coil and 28 x 28 x 0.3 cm3 field of view, 256 x 128 acquisition matrix, 30° flip angle, 13 ms TE, and 28 ms TR. Images and maps were reconstructed to a 128 x 128 matrix and retrospectively randomly undersampled by 2x, with full sampling over 22 central k-space lines. Temperature maps were reconstructed by fitting the k-space hybrid model to the entire image, or to the brain only with keyhole or POCS methods used to reconstruct the water bath image.

**Results**

Figure 30d shows the temperature reconstruction results. When the k-space hybrid model is fit to the entire image without distinguishing between the brain and water bath, phase artefacts obscure the hot spot in the reconstructed temperature map and (in this case) lead to an overestimation of the temperature rise in the sonicated region across image dynamics (RMSE across dynamics: 0.0121°C). Restricting the temperature reconstruction to within the brain, in combination with keyhole reconstruction of the water bath image (using the baseline image’s k-space to fill in missing k-space lines), produces temperature maps with lower errors in the hot spot but still large errors outside (RMSE across dynamics: 0.0039°C). The proposed k-space brain/POCS bath approach yields a more accurate estimate of the water bath image (not shown), resulting in much lower in-brain temperature artefacts (RMSE across dynamics: 0.0029°C).

**Conclusions**

Unpredictable water bath motion confounds model-based approaches to accelerated MR temperature mapping, resulting in large temperature artefacts due to aliased water bath signal. We demonstrated that a spatially-segmented reconstruction that applies a model-based reconstruction in the brain and a POCS reconstruction in the water bath can reconstruct temperature maps without undersampling artefacts at a moderate acceleration factor using a single receive coil. Future work will focus on integrating the approach with other accelerated temperature mapping methods [2] and extending it to non-Cartesian trajectories [4]. The method is compatible with multiple receive coils.

***Acknowledgements***

*This work was supported by the Focused Ultrasound Foundation and NIBIB T32EB014841.*

**References**

[1] Gaur P *et al.* Magn Reson Med 2015;73: pp. 1914–1925.

[2] Todd *et al*. Magn Reson Med. 2009;62: pp. 406–19.

[3] Lustig M *et al*. Magn Reson Med 2007;58: pp. 1182–1195.

[4] Fielden *et al*. Proc Intl Soc Mag Reson Med 23. 2015:1631.

### O34 Efficient volumetric thermometry for MR-guided FUS brain treatment monitoring, using multiple-echo spirals and mixed update rates

#### Michael Marx, Pejman Ghanouni, Kim Butts Pauly

##### Radiology, Stanford University, Stanford, California, USA

**Objectives**

MR-guided focused ultrasound (MRgFUS) brain treatments are currently guided by one thermometry sequence: single-slice 2DFT MR thermometry. In this work, we divided treatment monitoring into two tasks, with different thermometry design goals for each, and developed sequences optimized for these goals: “Focal Spot Localization” and “Monitoring”. These sequences achieve greater imaging performance by utilizing multi-echo spiral thermometry, region-specific update rates, and MASTER slice interleaving.

Currently, focal spot targeting confirmation requires several low-power sonications to obtain high-resolution measurements in three dimensions. We developed a Focal Spot Localization sequence that obtains high-resolution measurements in-plane, at improved temperature precision compared to 2DFT, while also providing multiple-slices for through-plane characterization. This would reduce the number of sonications required, improving treatment time. Additionally, even lower-power sonications could be detected, improving patient safety.

During ablative treatment sonications, single-slice monitoring cannot detect through-plane shifts of the ultrasound focus, or unexpected out-of-plane heating. We developed a multi-rate thermometry Monitoring sequence that interleaves different sequences at different update rates to simultaneously achieve fast and precise focal monitoring, 3-dimensional focal spot measurement, and full brain monitoring. Fast and precise multi-slice monitoring of the focus ensures accurate thermal dose estimates for treatment feedback, while (slower) full-brain monitoring ensures patient safety.

**Methods**

All sequences were implemented using RTHawk (HeartVista, Menlo Park, CA) on a GE 3T 750 Signa scanner (GE Healthcare, Milwaukee, WI) equipped with the InSighTec Exablate Neuro (InSighTec, Haifa, Israel). All imaging was performed with the body coil, as is normally used with the Exablate system. Conventional 2DFT was implemented as a gold standard for comparison. The new sequences all use a 36 cm FOV to ensure that the transducer and water bath do not alias. Volunteer imaging was done with informed consent under IRB approval. Multi-frequency reconstruction was performed on all spiral data. Temperature uncertainty was measured as the per-voxel temporal standard deviation of temperature measurements, and averaged within manually segmented ROIs. Performance was also tested inside the transducer, using a gel phantom. Sequence parameters are compiled in Table 2.

The Focal Spot Localization sequence is a 5-slice 3-echo thermometry sequence, with doubled in-plane resolution as compared to conventional 2DFT (1.1x1.1 mm vs 1.09x2.18 mm), and acquisition time of 7 s. The Monitoring sequence interleaves 3 distinct sequences at different rates to monitor 29 total slices. The “Focus” is monitored using 3-slice 3-echo spiral imaging, for high-speed high-precision measurement of focal heating. Two adjacent slices, the “Boundary”, are monitored at half the temporal rate (also using 3-echo spiral) to fully characterize the focal spot. The remaining 24 slices, “Background”, were acquired using 8 blocks of 3-slice MASTER, with spiral readouts. Use of MASTER improves temperature uncertainty, compared to traditional slice interleaving, by increasing echo time. Limiting each MASTER block to 3 slices reduces inherent diffusion and motion-encoding artefacts. The three component sequences were interleaved such that Focus utilized 45% (15% per-slice) of the timeline, Boundary used 15% (7.5% per-slice), and Background the remaining 40% (1.7% per-slice).

**Results**

**Fig. 31 (abstract O34).**
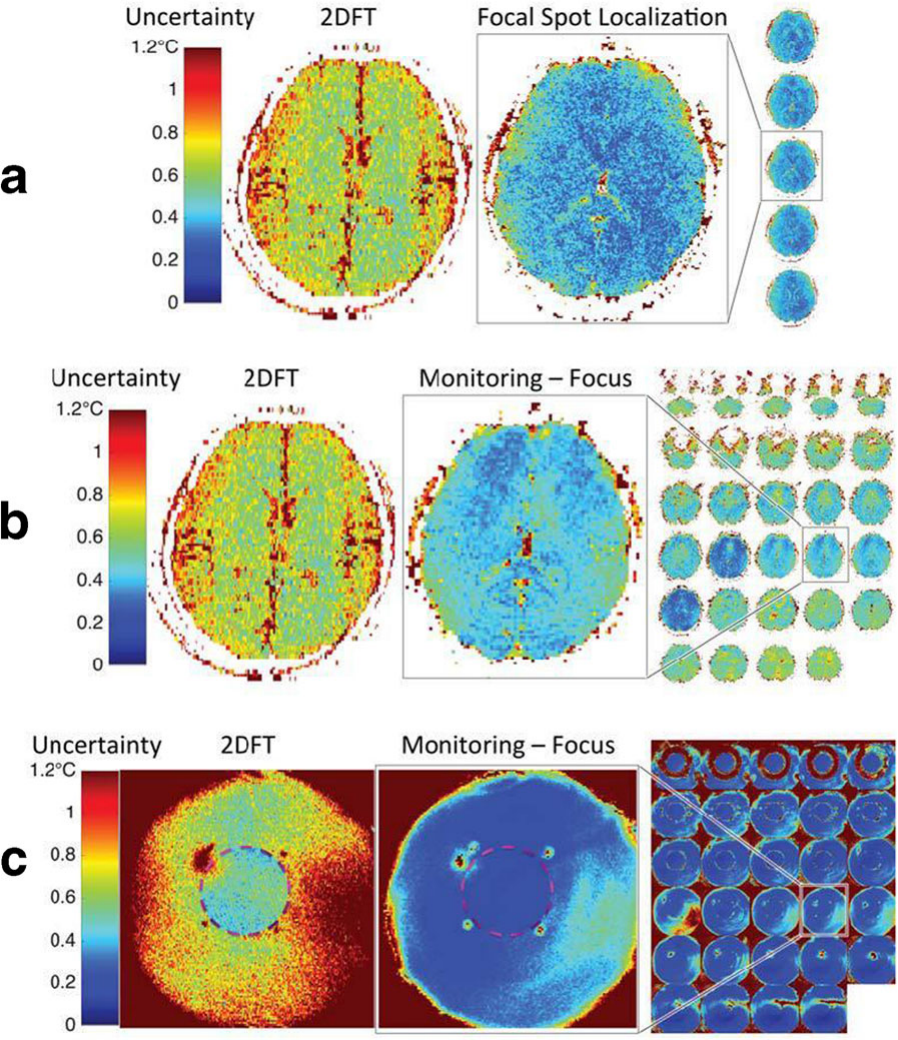
Temperature uncertainty comparisons between 2DFT and **a** Focal Spot Localization, in vivo; **b** Monitoring, in vivo; **c** Monitoring, phantom within transducer. Large images compare centre slices, while stack of images at right show additional slices. *Dotted outline* in 1C delineates the phantom

Figure 31a compares temperature uncertainty in vivo between 2DFT and Focal Spot Localization while Fig. 31b compares 2DFT with Monitoring for the same volunteer. Large images compare the centre slice, while stacks of images at the right show additional slices monitored by the new sequences. Figure 31c compares 2DFT and Monitoring uncertainty within the transducer. The new sequences obtained better uncertainty than 2DFT, with average values compiled in Table 2. Relative “Efficiency” is also listed in Table 2, which accounts for differences in speed and voxel volume. Each multi-echo spiral sequence is more than 150% as efficient as 2DFT. Background is 69% as efficient (but collects 3 slices per TR, for an effective 120% efficiency).

**Conclusions**

In this work, we have shown that multi-slice multi-echo spiral thermometry is an effective imaging approach for volumetric treatment monitoring. Improved imaging performance was successfully used to achieve imaging objectives for different aspects of ablative treatments. Focal spots may be localized faster and with reduced heating using the higher-resolution higher-precision Focal Spot Localization sequence. The mixed update rate Monitoring sequence successfully delivers high-speed high-precision monitoring of the targeted focus, while improving safety by simultaneously monitoring the full brain at a lower update rate. These sequences have also been validated within the transducer, to help ensure they will work in the clinical setting.

**Table 2 (abstract O34).** Implemented Sequence Performance Parameters. tSNR efficiency is proportional to (δ_xyz_*√(T_seq_)*σ_T_)^−1^ . For Monitoring - Background, median slice uncertainty reported

	Speed (s)	FOV (cm)	Slices	Res (mm)	Slice Thickness	Uncertainty (phantom)	Uncertainty (in vivo)	*in vivo* tSNR Efficiency
2DFT	3.82	28	1	1.09×2.19	3 MM	0.48°C	0.66°C	100%
Focal Spot Localization	7.00	36	5	1.10×1.10	3 mm	0.16°C	0.38°C	253%
Monitoring - Focus	2.39	36	3	2.00×2.00	2 mm	0.15°C	0.41°C	182%
Monitoring - Boundary	4.78	36	2	2.00×2.00	2 mm	1.03°C	0.33°C	160%
Monitoring - Background	9.56	36	24	2.00×2.00	2 mm	0.15°C	0.54°C	69%

### O35 Towards MR-guided focused ultrasound treatments near metallic hardware

#### Hans Weber, Valentina Taviani, Kim Butts Pauly, Pejman Ghanouni, Brian Hargreaves

##### Radiology, Stanford University, Stanford, California, United States

**Objectives**

To demonstrate how MRI can be used in FUS treatments near metallic hardware for treatment planning, sonication monitoring and treatment assessment.

**Methods**

Using standard MRgFUS product sequences, we treated a 73-year-old patient with a metastasis in the right femur that was painful despite prior radiation and surgical stabilization with a metallic rod, demonstrating the inability of both echo-planar imaging (EPI) and gradient-recalled echo (GRE) imaging to be used for conventional proton resonance frequency (PRF) shift thermometry.

**Fig. 32 (abstract O35).**
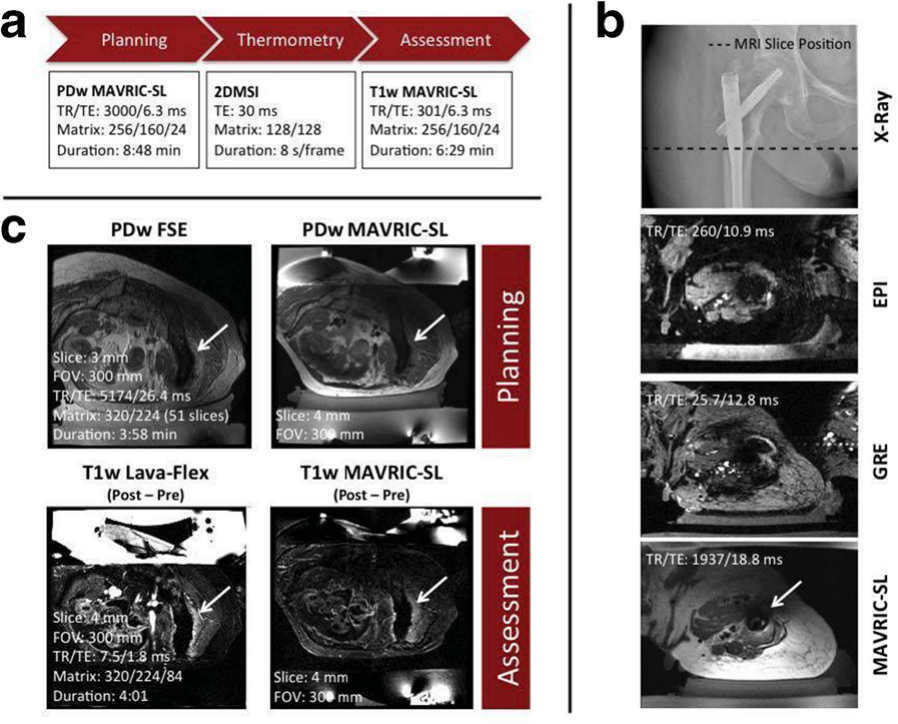
**a** Components of the proposed imaging strategy for MRgFUS near metallic hardware. **b** GRE and EPI images underlying conventional PRF thermometry and a MAVRIC-SL image, all acquired in a patient with a femur stabilized with a metallic rod. The *dashed line* in the x-ray image depicts the location of the MRI slices. **c** Comparison of both planning images and difference images (pre and post contrast injection) for treatment assessment, acquired in a patient without metallic hardware

Based on the experience from this patient treatment, we have proposed an imaging strategy for MRgFUS near metallic hardware using multi-spectral imaging (MSI) techniques (Fig. 32a). Both MAVRIC-SL [Koch et al.; MRM 2011; pp. 65:71] and 2DMSI [Hargreaves et al.; ISMRM 2014, #615] are spin-echo-train-based imaging approaches that enable artefact-reduced imaging near metal. MAVRIC-SL compensates for distortions induced by field inhomogeneities by additional encoding along the slice dimension. Its ability to adjust the image contrast makes it a promising candidate both for treatment planning and assessment. 2DMSI limits the excitation to finite spectral and spatial regions (“frequency bins”) that can be imaged with minimal artefact quickly enough to be used for sonication monitoring.

We preliminarily tested the feasibility of MAVRIC-SL for treatment planning and assessment in a 65-year-old patient without metal hardware and with a metastasis in the right pelvis undergoing MRgFUS treatment for pain palliation. We used a GE 3T MRI system equipped with an InSightec ExAblate2000 FUS system. Proton density (PD) weighted MAVRIC-SL images were acquired prior to the treatment in addition to standard 2D fast-spin-echo (FSE) images. After treatment, T1-weighted MAVRIC-SL images were acquired before and after gadolinium injection in addition to standard 3D fast RF-spoiled GRE images with 2-point-Dixon fat suppression (LAVA-Flex, see Fig. 32c for details).

We tested the feasibility of 2DMSI to monitor a 25 s sonication in an acrylamide egg-white phantom containing the CoCr stem of a knee replacement and a pork loin sample containing a CoCr augment plate from the same hardware, each placed in a container filled with water. We acquired a time series of 10 single-slice 2DMSI images with a temporal resolution of 8 s/frame and the sonication starting after the third image. Each 2DMSI image was composed of 12 frequency bins ranging from −4.5 kHz to +5.4 kHz, and was acquired with TE = 30 ms, (bin) TR = 500 ms, 280 x 280 mm FOV, 5 mm slice thickness and 128 x 128 matrix size (effective number of phase encodings after half Fourier). To monitor the temperature-induced signal change, we subtracted the mean signal of the 3 baseline images from all 10 images voxel-by-voxel.

**Results**

Figure 32b shows the GRE and EPI image acquired in the patient with metallic hardware. With both techniques, the stabilized femur and its surrounding area are not visible due to distortions and signal dephasing. For comparison, the MAVRIC-SL image depicts the anatomy including the bone marrow surrounding the metallic rod.

Figure 32c presents the acquired images for treatment planning and assessment in the patient without metal. PD-weighted MAVRIC-SL achieves a contrast comparable that of the conventional FSE image and allows for localization of the metastasis in the lower part of the right pelvis. The T1-weighted MAVRIC-SL pre/post contrast difference image reveals the treatment area similar to the LAVA-Flex water difference image.

**Fig. 33 (abstract O35).**
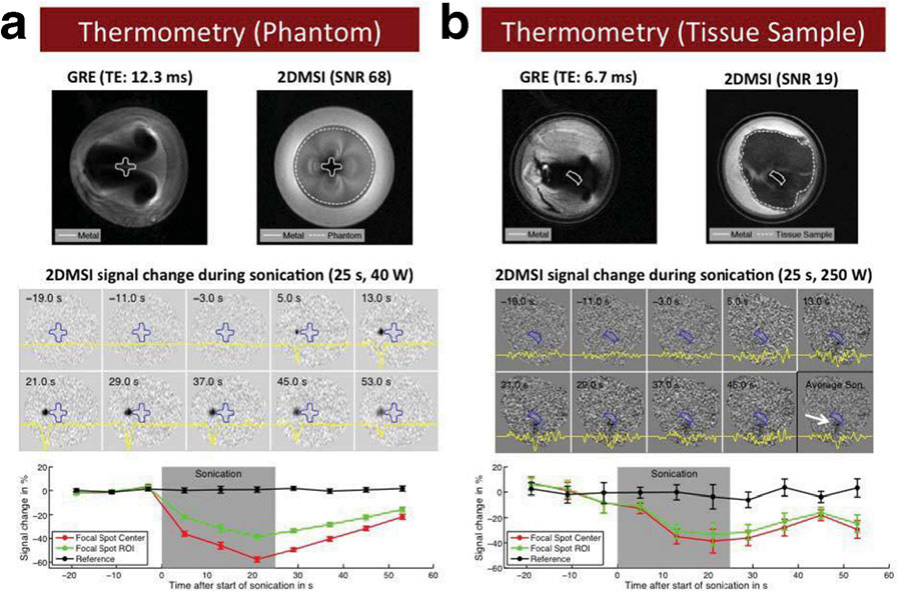
GRE and 2DMSI image and 2DMSI signal change during sonication in the phantom (**a**) and the ex vivo tissue sample (**b**). The 2DMSI difference images are cropped and masked to the dashed area. For the tissue sample, the 2DMSI difference image in the lower right shows the average over the three time frames during sonication

Figure 33 shows the thermometry results in the phantom and the tissue sample. In both cases, metal-induced field inhomogeneities of up to ± 4 kHz cause strong distortions and signal loss in the GRE image, whereas 2DMSI clearly depicts the area around the metal. In the phantom, the 2DMSI difference images yield a clear signal change at the focal spot next to the metallic stem and a noticeable change in signal at the focal spot next to the metallic plate in the tissue sample, despite a 70% reduction in SNR due to the lower water content. Averaging over the frames during sonication improves the localization of the focal spot.

**Conclusions**

We have presented initial results for our proposed imaging strategy for MRgFUS in the presence of metallic hardware.

MAVRIC-SL is an established technique for artefact-reduced imaging near metal. Here, we have shown that the image contrast can be adjusted to yield the relevant information for both planning and assessment of MRgFUS treatments. The reduced image resolution compared to the standard FSE protocol (to keep the scan duration at an acceptable length) did not noticeably reduce diagnostic image quality for the treating radiologist.

We have also demonstrated that 2DMSI enables the measurement of temperature-induced signal changes in close proximity to metallic hardware and thus in regions where conventional PRF shift thermometry fails. The bin-selective approach allows for a temporal resolution of 8 s/frame, which is sufficiently high to resolve the temperature evolution in sonications as short as 20 s, which is the typical duration in MRgFUS treatments. In case of less severe metal-induced artefacts, the number of frequency bins could be reduced to increase the temporal resolution. Averaging over time frames could facilitate the detection of the focal spot in lower SNR cases. For the given temporal resolution and the low temperature sensitivity of the T2 relaxation time of aqueous tissues, the measured signal change is expected to be highly dominated by the temperature sensitivity of the PD, whereas a higher temporal resolution is expected to increase the T1 weighting. While the latter provides higher temperature sensitivity and thus lowers the SNR requirements, PD weighting offers the benefit of a tissue-independent temperature mechanism that could facilitate quantitative thermometry.

In conclusion, the proposed imaging strategy has the potential to enable MRgFUS treatments near metallic hardware. The patient population at greater risk for cancers, and hence osseous metastases, overlaps with the elderly demographic more likely to have metallic hardware such as joint replacements. Further, orthopaedic bone stabilization is often used as a treatment for osseous metastases at risk for fracture. Overcoming these technical limitations is therefore important to allow the use of MRgFUS in a larger patient population.

### O36 Thermal monitoring of HIFU using thermal memory effect of phase-change nano droplet

#### Jun Tanaka, Kentaro Kikuchi, Ayumu Ishijima, Takashi Azuma, Kosuke Minamihata, Satoshi Yamaguchi, Teruyuki Nagamune, Ichiro Sakuma, Shu Takagi

##### The University of Tokyo, Tokyo, Japan

**Objectives**

Phase change nano droplets (PCND), whose diameters are 200–400 nm, are droplets of perfluorocarbon (PFC) covered with phospholipid layers. Since they can be vaporized by ultrasound and transformed to microbubbles, they will be utilized as ultrasound contrast agents and ultrasound therapy sensitizers. There are several types of PCND which are often used in research, whose internal compositions are different, such as perfluoropentane (PFP) and perfluorohexane (PFH).Its boiling point can be adjusted by changing the mixture ratio of PFP and PFH.

The lifetimes of generated microbubbles changed from PCND are different from type to type. In this study, the lifetime dependence to ambient temperature at the moment of its vaporization was investigated. If remaining efficiency depends on the ambient temperature, this drug has a potential to be used as indicator of thermal memory effects and temperature monitoring agents.

**Methods**

**Fig. 34 (abstract O36).**
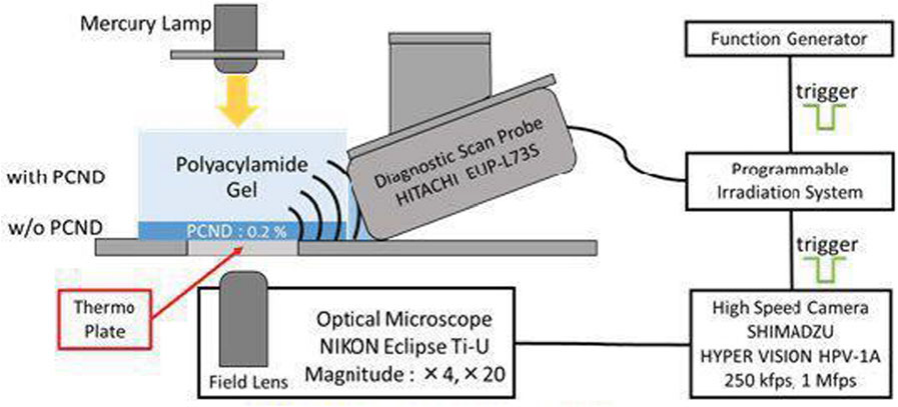
Experimental setup

Experimental setup for ultrasound exposure and high-speed imaging are shown in Fig. 34.In this study, we used the two types of PCND, whose internal compositions were PFP and the mixture (MIX; PFP: PFH = 1:1), respectively. Their main differences are boiling points (PFP: 29°C, MIX: 40°C), and the boiling point of MIX is estimated by thermodynamic calculation.

For the observation of the vaporization of PCND, we used two-layer structure of polyacrylamide gel phantom (layer with PCND and layer without PCND), and set the layer with PCND at the focal point of the transducer. PCND were vaporized by ultrasound (5 MHz centre frequency, 5 cycle bursts, Peak Negative Pressure = 3.5 MPa), which were irradiated with an arbitrary ultrasound beam controller (Verasonics) and a linear array transducer (EUP-L73S, Hitachi Aloka Medical). Time-lapse behaviours of PCND through phase changes were recorded with the high-speed camera (HPV-1A, SHIMADZU), coupled with inverted microscope (NIKON Eclipse Ti-U). The ambient temperature (gel phantom temperature) conditions were controlled with a hot water bath and thermo plate (TOKAI HIT) on the stage of the microscope.

**Results**

**Fig. 35 (abstract O36).**
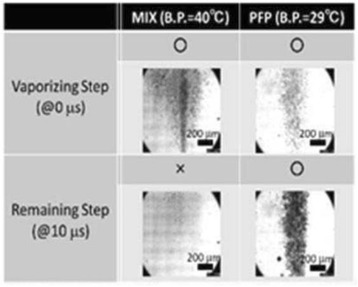
High Speed Images of Vaporization at 37 °C

First, we observed the vaporization of the two types of PCND at 37 °C. The high-speed images of vaporization at 37 °C are shown in Fig. 35.

**Fig. 36 (abstract O36).**
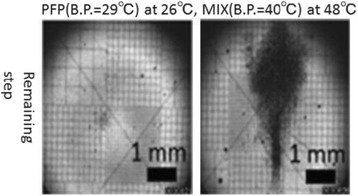
High speed images of vaporization at 26, 48 °C

As to MIX (B.P. = 40 °C), generated microbubbles disappeared soon (within 10 μs) after the ultrasound exposure. On the other hand, as to PFP (B.P.=29 °C ), generated microbubbles remained for a while (more than thirty seconds). Because the main difference of these two types of PCND is the boiling point, we assumed that temperature is the key factor, and tried to control the difference between the boiling point and the ambient temperature. Then, we did some vaporization experiment at various temperatures. Some of the high-speed images of vaporization at 26 °C, 48 °C are shown in Fig. 36.

**Fig. 37 (abstract O36).**
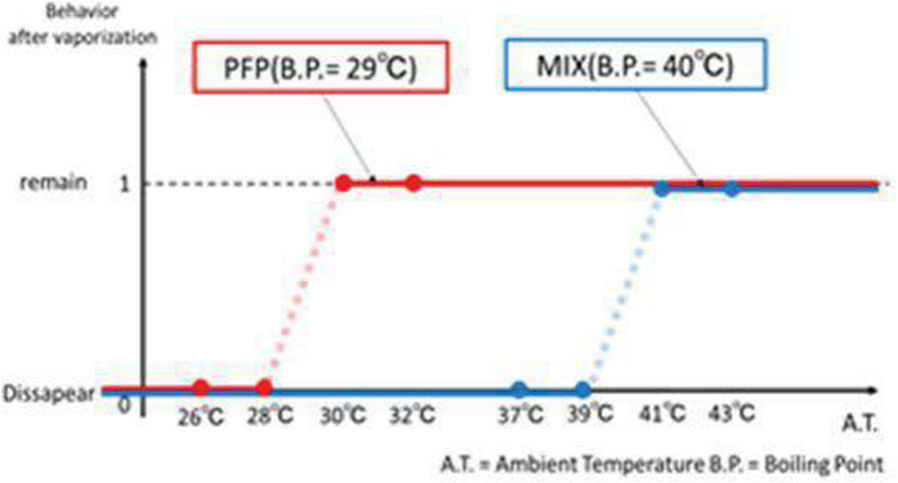
Behavior after vaporization at 26~48°C

As to PFP (B.P. = 29 °C), generated microbubbles disappeared soon after the sonication at 26 °C, although they remained for some time at 37 °C. As to MIX (B.P.=40 °C), generated microbubbles had long lifetimes at 48 °C, although they had very short lifetimes at 37 °C. It can be considered that the lifetimes of generated microbubbles are greatly affected by not only the internal composition, but also the ambient temperature. Besides, behaviours after vaporization at 26~48 °C are shown in Fig. 37. Whether generated microbubbles will remain or disappear was switched around the boiling point.

**Conclusions**

We found that the lifetime of microbubble highly depended on the ambient temperature at the moment of vaporization. This effect has a potential to be used as indicator of thermal memory effects and temperature monitoring agents.

*Acknowledgements*

*Authors thanks to Dr. Kawabata and Mrs. Asami in Hitachi Central Research Laboratory.*

### O37 Localized blood brain barrier opening of the macaque brain using a high frequency 512-elements FUS transducer and ultrasound contrast agent

#### Mathieu D. Santin^1,2^, Laurent Marsac^3,4^, Guillaume Maimbourg^5^, Morgane Monfort^2^, Benoit Larrat^4^, Chantal François^2^, Stéphane Lehéricy^1,2^, Mickael Tanter^6^, Jean-Franҫois Aubry^4^

##### ^1^Centre de NeuroImagerie de Recherche - CENIR, Paris, France; ^2^Inserm U 1127, CNRS UMR 7225, Sorbonne Universités, UPMC Univ Paris 06 UMR S 1127, Institut du Cerveau et de la Moelle épinière, ICM, Paris, France; ^3^Supersonic Imagine, Aix-en-Provence, France; ^4^Institut Langevin, CNRS, Paris, France; ^5^Institut Langevin, Université Denis Diderot, Paris, France; ^6^Institut Langevin, INSERM, Paris, France

**Objectives**

Blood Brain Barrier (BBB) protects the brain from most of the pathogen circulating within the bloodstream. Thus, the BBB renders a difficult brain penetration for most of the drugs used for brain therapy. Focused ultrasound has the ability to bypass the BBB in small regions considered as targets for various drugs such as chemotherapies. Being able to open the BBB in a restricted focal zone is a necessary step before using this method for therapies such as drug delivery. Here we developed an in-house primate-dedicated stereotactic frame mounted on a multielement focused ultrasound array to perform BBB opening, using combination of FUS and ultrasound contrast agent (UCA). Stereotactic images were loaded in a planning software that allows controlling the localization of the BBB opening. Sonications were real-time monitored using a passive cavitation detector (PCD) to detect signatures of stable or transient inertial cavitation originating from the UCA.

**Methods**

Experiments were conducted on an anesthetized macaque (Macaca fascicularis) each month during one year. Animal was anesthetized with a mixture of Ketamine (3mg/kg) and dexmedetomidine (15μg/kg) and the anaesthesia was maintained by an infusion of Alfaxan (Alfaxalone, 0.2mg/kg/min). The temperature of the animals was maintained at ~37°C using a heated water blanket. Animal physiology was monitored during the whole experiment. A homemade stereotactic frame holding the monkey head was affixed to a 512-element transducer resonating at 1 MHz (SuperSonic Imagine, France). Images were imported in a planning software in which all the positions of the head of the monkey in regard to the transducer were stored, along 6 axis of freedom (2 rotations and one translation for the transducer and one rotation and two translations for the head). Once the coordinate of the target was chosen in the stereotactic frame coordinates, the planning software allowed determining the position of the frame and the transducer. MRI was performed using a 3T Siemens Verio system (Siemens, Germany). Body coil was used for excitation and an 8-channel phased-array coil (Life Services LLC, USA) dedicated to primates was used for reception. T1 longitudinal relaxation time was obtained at baseline using an MP2RAGE sequence prior to BBB opening1. Ultrasound excitation consisted of a 0.6 MPa Peak Negative pressure (as estimated at focus in the brain) sinusoidal tone burst of 10 ms, with a pulse repetition frequency of 1 Hz during 120s. Excitation followed a bolus injection of 1.5 mL of the UCA (Sonovue, Bracco, Switzerland) and lasted for 2 minutes.

The backscattered signal from microbubbles during insonification was recorded using a wideband (−6dB bandwidth: 4.5 - 14.4 MHz) transducer (Imasonic, France) acting as a PCD. The PCD was fixed at the right temporal bone window of the monkey perpendicularly to the FUS beam. This minimizes signal contamination from the main excitation field.

A bolus of 1.5 mL of an MRI contrast agent (Dotarem, Guerbet, France) was injected 5 minutes after the end of ultrasound excitation. A second MP2RAGE dataset was obtained 10 minutes after contrast agent injection to monitor the localization of the BBB opening resulting in a T1 decrease in the region of interest (ROI) induced by the contrast agent.

A conventional clinical scan consisting on a T1-, T2- and T2*-weighted MRI and DTI was performed at the end of the experiments to assess clinical status of the animal.

**Results**

**Fig. 38 (abstract O37).**
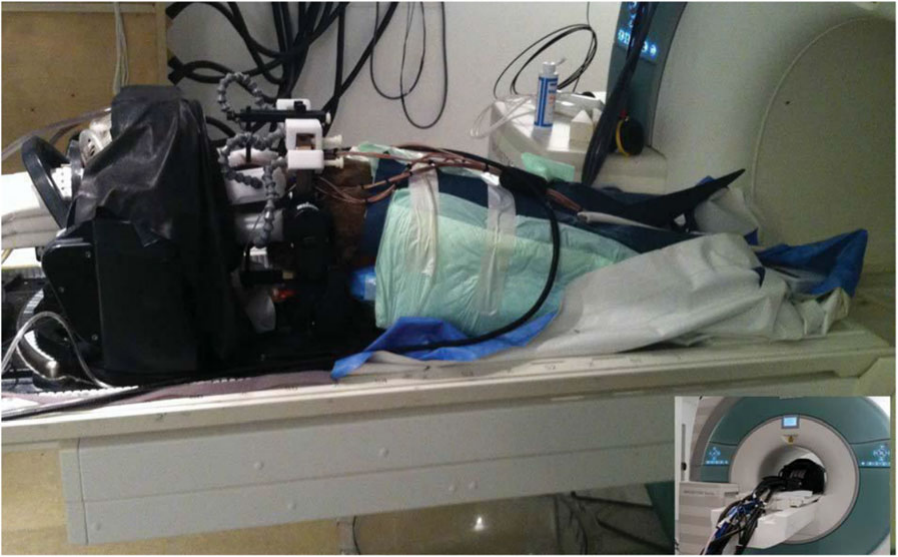
Picture showing the animal within the setup and inside the MRI

**Fig. 39 (abstract O37).**
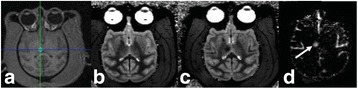
**a**. Planning software and target ROI definition. **b**. T1 map before BBB opening. **c**. T1 map after BBB opening. **d**. T1 difference between both images, arrow indicates the BBB opening on the targeted ROI

This study was conducted in a living macaque during one year. Figure 38 shows the apparatus in position with the monkey and inside MRI. After BBB opening, T1 decrease was obtained in the ROI defined by the planning software (example on Fig. 39), indicating that BBB was opened in the targeted ROI. T1 values were 1000 +/− 70 ms before and 662 +/ -31 ms after the HIFU procedure resulting in a ~33% decrease in T1. The size of the area of BBB opening was 3.2 mm in diameter, and 5.6 mm along the axis of the beam.

**Fig. 40 (abstract O37).**
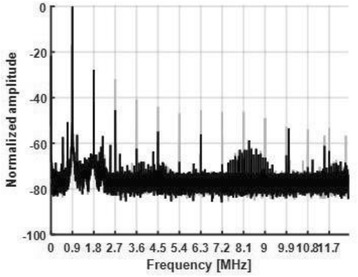
Representative spectra obtained with the PCD. Black color represents the spectrum before injection of UCA, grey colour represents the spectrum with UCA. More and higher harmonics are identified when UCA is present. Broadband noise level is not different with or without UCA

**Fig. 41 (abstract O37).**
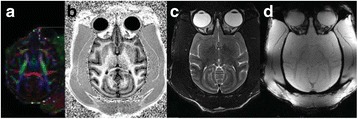
**a**. Colour Coded fractional anisotropy. **b**. T1-weighted image. **c**. T2-weighted image **d**. T2*-weighted image. No signs of oedema, haemorrhages or bleeding could be seen 3 weeks after BBB opening

Figure 40 shows the typical spectra obtained before and during infusion of gas microbubbles. Level of harmonics increased significantly for third (+13.6 dB) and fourth harmonics (+21.6 dB), but no significant increase in broadband noise was detected, suggesting that no transient inertial cavitation occurred during the insonification. After the experiment, the animal recovered and no side effects were observed during the 3 weeks following each BBB opening procedure. At the end of all the BBB opening procedures, T1-, T2- and T2*-weighted scans and DTI did not show any evidence of tissue damage (oedema, haemorrhages or bleeding) induced by the ultrasound procedures (Fig. 41).

**Conclusions**

The procedure allowed successful repeated transient opening of the BBB in a small ROI in a living primate with no side effects. No signatures of transient inertial cavitation were detected during experiments. This suggests that this method is safe for the animal. This study will be replicated in other animals with the long-term objective of developing a system suitable for human applications.

**Acknowledgements**

*This work was supported by the Bettencourt Schueller Foundation and the "Agence Nationale de la Recherche" under the program “Future Investments” with the reference ANR-10-EQPX-15.*

### O38 Enhanced neurorestoration through triple treatment with focused-ultrasound facilitated delivery of the neurotrophic factor neurturin

#### Maria Eleni Karakatsani^1^, Gesthimani Samiotaki^1^, Shutao Wang^1^, Camilo Acosta^1^, Eliza R. Feinberg^2^, Elisa E. Konofagou^1,3^

##### ^1^Biomedical Engineering, Columbia University, New York, New York, USA; ^2^Biological Sciences, Columbia University, New York, New York, USA; ^3^Radiology, Columbia University, New York, New York, USA

**Objectives**

Currently, the existing Central Nervous System (CNS) drug delivery techniques are confined to either targeted but invasive or to non-targeted and non-invasive methods. Focused Ultrasound (FUS) coupled with the systemic administration of microbubbles has been proven to open the Blood Brain Barrier (BBB) locally, transiently and non-invasively, thus facilitating the diffusion of neurotrophic factors. Neurturin, a member of the glial derived neurotrophic factors (GDNF) family has been demonstrated to have restorative effects on the depleted by Parkinson’s disease dopaminergic neurons (DA). Moreover, our group has shown the bioavailability and downstream signalling of Neurturin in wild type mice and the restorative effect in Parkinsonian mice. Despite the promising results, the potential of multiple treatments with Neurturin in reversing the disease phenotype is still to be determined. The aim of the current study was to investigate the neurorestorative effect of triple delivery sessions of the neurotrophic factor Neurturin in a Parkinsonian mouse model.

**Methods**

For this study, twelve wild type mice (12 months old) were infused with sub-acute dosages of MPTP causing apoptotic degeneration in the nigrostriatal pathway. After the stabilization of the lesions and the decontamination period, the entire cohort was sonicated on the left hemisphere (ipsilateral side) targeting twice the Caudate Putamen region (CPu), to cover the entire area, and once the Sabstantia Nigra region (SN). Half of the mice received an IV injection of 0.5mg Neurturin accounting for the treated group, FUS+/NTN+, while the rest constituted the control group, FUS+/NTN-. Magnetic resonance imaging (MRI) was performed after each sonication to verify the accuracy of the BBB opening in terms of targeting. The procedure was repeated once biweekly to a total of three treatments. Following the third treatment, the survival period lasted for 28 days letting the neurotrophic factor to develop its restorative effects. On the 29th day, the mice were sacrificed and coronally sectioned for tissue processing. The brain slices of both the SN and the CPu were stained for tyrosine hydroxylase positive cells (TH+) with a custom protocol. The stained slices were imaged to count the TH+ nerve cell nuclei on the SN while the axons and dendrites were quantified by a custom MATLAB algorithm. For each mouse the contralateral side was compared to the ipsilateral side to eliminate inter-animal variation in the number of nuclei and projections. A quantification algorithm was used to compute the percentage of the relative difference (RD) between the two hemispheres, i.e., RD = (Ipsilateral – Contralateral)*100%. The process was repeated for all slices that cover the entire SN region and averaged across the mice. The error of the technique was measured as the standard deviation from the mean.

**Results**

There was no significant difference in the number of neurons between the ipsi- and contralateral sides. This result was in accordance with our knowledge of Neurturin restoring impaired neurons and not regenerating them. The RD was found to be significantly higher for the FUS+/NTN+ compared to the FUS+/NTN- group. This significance strengthens with the negative percentage of the FUS+/NTN- group implying a possible sensitivity of the Parkinsonian brain in multiple sonications.

**Conclusions**

These findings indicate a potential of multiple treatments on the reversal of the Parkinsonian phenotype as is the first time significance is reported. To strengthen this argument a second cohort of 20 mice is currently undergoing the same treatment aiming to apply various other imaging and quantification techniques to investigate the restoration of the functionality of the previously depleted neurons. Nonetheless, the current findings are essential considering the therapeutic effect of multiple treatments with FUS enhanced drug delivery in patients.

**Fig. 42 (abstract O38).**
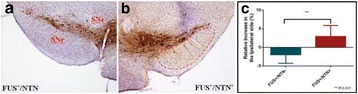
**a** Contralateral side (control) of TH+ stained neuronal cells. **b** Ipsilateral side (treated) of TH+ stained neuronal cells. **c** Statistical analysis of the averaged TH+ stained neuronal cells of the two groups

### O39 Long term effects of single *vs* repeated low intensity pulsed focused ultrasound treatment with microbubbles

#### Zsofia I. Kovacs^1^, Tsang-Wei Tu^1^, Georgios Z. Papadakis^1,2^, William C. Reid^2^, Dima A. Hammoud^2^, Joseph A. Frank^1,3^

##### ^1^Frank Laboratory, Radiology and Imaging Sciences, National Institute of Health, Bethesda, Maryland, United States; ^2^Center for Infectious Disease Imaging (CIDI), Radiology and Imaging Sciences, National Institute of Health, Bethesda, Maryland, United States; ^3^National Institute of Biomedical Imaging and Bioengineering, National Institute of Health, Bethesda, Maryland, United States

**Objectives**

One potential issue for using MR-guided pulsed Focused Ultrasound (pFUS) to open the blood brain barrier (BBB) is the lack of data on the long term effects. Safety determination in the brain have been limited to the MR characterization after repeated BBB opening that can be achieved without haemorrhage, oedema and behavioural changes in non-human primates [1,2]. We use multimodal imaging technics to characterize long term effects of pFUS + MB in the rat brain to evaluate the effects of repeated BBB opening by pFUS and microbubbles (MB) on morphology to the rat striatum and hippocampus as monitored by magnetic resonance imaging (MRI), positron emission tomography (PET) and histology over 12 weeks.

**Methods**

Female rats were divided into two groups and received either pFUS + MB (OptisonTM, GE Healthcare, Little Chalfont, UK) once or six times targeting the striatum and the contralateral hippocampus. 200 μl of MB were administered intravenously over 1 minute starting 30 sec before pFUS. Rats received 3 daily doses of 300 mg/kg 5-Bromo-2′-deoxy-uridine (BrdU, Sigma Aldrich, St. Louis, MO) intraperitoneally before sonication to label proliferating cells in vivo.

0.3 MPa acoustic pressure was applied in 10 ms burst length and 1% duty cycle (9 focal points, 120 sec/9 focal points - striatum, 120 sec/4 focal points - hippocampus) using a single-element spherical FUS transducer (centre frequency 589.636 kHz; focal number 0.8; aperture 7.5 cm; FUS Instruments, Toronto, Ontario, Canada). T2, T2* and Gd-enhanced T1-weighted images were obtained by 3.0 T MRI (Philips, Amsterdam, Netherlands), T2, T2*, diffusion tensor imaging (DTI) and chemical exchange saturation transfer (CEST) imaging was performed by 9.4 T MRI (Bruker, Billerica, MA). Parameters for DTI: 3D spin echo EPI; TR/TE 700 ms/37 ms; b-value 800 s/mm2 with 17 encoding directions; voxel size 200 μm, isotropic. Diffusion weighted images were corrected for B0 susceptibility induced EPI distortion, eddy current distortions, and motion distortion with b-matrix reorientation using Tortoise. Parameter for glucoCEST: 2D fast spin echo with (MT) and without (M0) magnetization transfer (MT) pulses (TR/TE 3.5 s/11.5 ms; in plane resolution: 200 μm, thickness: 0.8 mm; MT pulse: 3 μT, 1 s). The MT offset frequencies (Δω) were set from −2 kHz to +2 kHz with 100 Hz stepping to detect the proton metabolites of glucose (1.2 ppm, 2.1 ppm, 2.9 ppm). Fractional anisotropy (DTI-FA) and the asymmetry of magnetization transfer ratio (MTRasym) were derived for mapping structural injury and glucose metabolism.

Quantitative of glucose uptake was performed with FDG-PET (Siemens, Munich, Germany). Each rat received under anaesthesia (O2 3–4 L/min & Isoflurane at 3–3.5 4%) 0.7 – 1.1 mCi of 18F-FDG via tail vein injection and was allowed to regain consciousness for an uptake period of 30 minutes in total. They were anesthetized again and a PET/CT study was acquired using Siemens Inveon Multimodality scanner (Siemens Medical Solutions USA, Inc.). CT scan was performed for localization and attenuation correction purposes. PET images were reconstructed using OSEM3D/MAP algorithm, with Ramp projection filter, scattered corrected, 2 OSEM3D iterations, 18 MAP iterations, 128 × 128 image size, and approximately 0.5mm resolution at the centre of the field of view (FOV).

Animals were euthanized 6 or 12 weeks after the first pFUS treatment. Histological evaluation of brain and tracking of BrdU tagged cells was performed at different time points. Values were compared to baseline.

**Results**

**Fig. 43 (abstract O39).**
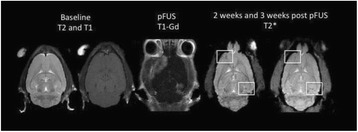
3.0T MR images of a rat brain show Gd-extravasation immediately after pFUS + MB and delayed haemorrhage at the sonicated tissue (left striatum and right hippocampus) associated with BBB opening

**Fig. 44 (abstract O39).**
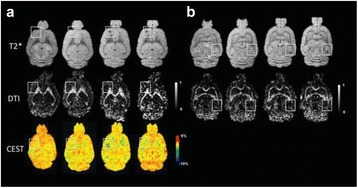
DTI and glucoCEST (9.4T MRI) of the rat brain (baseline, 1 day, 2 weeks and 3 weeks post pFUS) show changes in the grey and white matter tract at both sonicated locations. 2 weeks after pFUS + MB increased fractional anisotropy on DTI suggests astrogliosis in the striatum (**a**) and axonal injury in the external capsule (**b**). Decreased signal intensity in glucoCEST indicates lower glucose metabolism at the site of the sonication (**a**)

Preliminary results showed contrast enhancement on T1-weighted MRI in rats receiving a single sonication, indicating BBB disruption in the striatum and the hippocampus. Gd-extravasation or T2 and T2* abnormalities were not seen in the brain 1 day post pFUS + MB at 9.4 T MRI. Hypointense regions appeared on T2* MRI 2 weeks after pFUS + MB (Figs. 43 and 44) consistently with microhemorrhage within the parenchyma that decreased in volumes by week 3. White matter fiber structure- and gray matter-abnormalities on DTI MRI were detected in regions with T2* abnormalities (Fig. 44) suggestive of increased astrogliosis (Fig. 44a) and transient axonal damage (Fig. 44b). GlucoCEST showed loss of contrast as early as 1 day post pFUS and these changes persisted up to week 3 (Fig. 44a).

Qualitative analysis of MRI and GlucoCEST as well as ^18^F-FDG uptake with PET showed no difference between the sonicated region and the contralateral hemisphere 6 weeks post sonication.

**Conclusions**

We have observed a complex graded molecular and cellular sterile inflammatory response in the brain up to 24 hrs after pFUS + MB. However, little is known about the long term effects in rats using advanced imaging techniques. The DTI data showed that pFUS caused a low degree of structural injury at the location of sonication. However, the decrease in glucose concentration revealed by glucoCEST indicated that the pFUS could cause hypo-metabolism in the brain even after 3 weeks post sonication. These preliminary results suggest the importance of long term monitor of the brain following low intensity pFUS + MB.

Further research investigations are in process to evaluate changes following multiple targeted treatments in the brain.

**References**

[1] Arvanitis, C. D., et al., *Cavitation-enhanced nonthermal ablation in deep brain targets: feasibility in a large animal model*. J Neurosurg:1–10, 2015.

[2] Downs, M. E., et al., *Long-Term Safety of Repeated Blood–brain Barrier Opening via Focused Ultrasound with Microbubbles in Non-Human Primates Performing a Cognitive Task*. PLoS One 10(5):e0125911, 2015.

### O40 Low intensity pulsed focused ultrasound and microbubbles results in sterile inflammatory response in the rat brain

#### Zsofia i. Kovacs^1^, Saejeong Kim^1^, Neekita Jikaria^1^, Michele Bresler^1^, Farhan Qureshi^1^, Joseph A Frank^1,2^

##### Frank Laboratory, Radiology and Imaging Sciences, National Institute of Health, Bethesda, Maryland, USA; ^2^National Institute of Biomedical Imaging and Bioengineering, National Institute of Health, Bethesda, Maryland, USA

**Objectives**

Very little is known about the graded cellular and molecular responses in the brain following pulsed Focused Ultrasound (pFUS) coupled with microbubbles (MB) exposures being advocated to increase drug or gene delivery through the disruption of the blood brain barrier (BBBD). We investigated the proteomic changes in the brain in response to Pulsed Focused Ultrasound (pFUS) + intravenous (IV) ultrasound contrast agent MB associated with BBBD.

**Methods**

MRI-guided pFUS was performed at 0.3 MPa acoustic pressure, 10 ms burst length and 1% duty cycle (9 focal points, 120 sec/9 focal points) using a single-element spherical FUS transducer (centre frequency: 589.636 kHz; focal number: 0.8; active diameter: 7.5 cm; FUS Instruments, Toronto, Ontario, Canada). 200 μl of OptisonTM MB (GE Healthcare, Little Chalfont, UK) were administered IV over 1 minute starting 30 sec before pFUS. Gd-enhanced T1-weighted images were obtained with a 3.0 T MRI (Philips, Amsterdam, Netherlands). Quantitative protein and mRNA expression in the brain following pFUS + MB were analysed with Bio-Plex ProTM Assay (Bio-Rad Laboratories, Inc., CA), enzyme-linked immunosorbent assay (ELISA), Western blot, Real-Time PCR (RT-PCR) or immunofluorescent staining. Proteomics were normalized to sham and statistical analysis was performed by one-way ANOVA corrected for multiple comparisons. 2 fold increase in mRNA expression was determined as significant. Rats were injected with 8 mg/kg Rhodamine encapsulated magnetic polymers (Biopal Inc., Worcester, MA) 3 days prior to pFUS to tag splenic macrophages. No evidence of damage or microhaemorrhage was observed on histology.

**Results**

**Fig. 45 (abstract O40).**
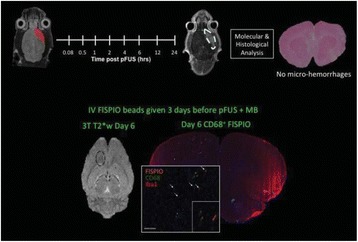
T2*wMRI at 3.0T performed on day 6 post pFUS + MB. Recruited Fluorescent (Fl) SPIO labelled splenic macrophages homing to sonicated brain. FlSPIO (*orange*), CD68 macrophages (*green*) and microglia Iba1 (*red*) are consistent with injury to brain

The results of harvested brains at various times post sonication were as follows:

1. pFUS + MB resulted in BBBD by T1wMRI and by histology (albumin staining) without evidence of microhaemorrhages;
2. pFUS + MB induced a rapid (within 5 minutes) increased expression of pro-inflammatory and anti-inflammatory cytokines, chemokines and trophic factors originating from components of the neurovascular unit lasting up to 24 hours post sonication;
3. Proteomic analysis revealed increased heat shock protein 70 (HSP70), tumour necrosis alpha (TNFa), interferon gamma (IFNg) and interleukin (IL) 1a, 1b, 2, 5, 6, 17 and 18 consistent with damage associated molecular patterns (DMAP) (Chen and Nunez 2010) and activation of nuclear factor kappa-light-chain-enhancer of activated B cells (NFkB) inflammatory pathways observed with sterile inflammatory response to injury or insult (Fig. 45);
4. RT-PCR demonstrated activation of inflammatory genes associated with NFkB pathway along with anti-apoptotic genes, immune cell chemoattractants, selectins and cell adhesion molecule (CAM);
5. Evidence of influx of fluorescent bead labelled splenic macrophages in the brain by day 6 post pFUS along with activated astrocytes and microglia consistent with mild injury to the parenchyma.

**Conclusions**

The temporal molecular response to pFUS + MB is indicative of sterile inflammatory response (Gadani, et al. 2015) in the parenchyma originating from neurovascular unit. The pattern of pro-inflammatory cytokines immediately after pFUS + MB exposure is consistent with sterile inflammation initiated by DAMP that are released in response to ischemia or trauma associated with sterile inflammation observed with mild trauma or ischemia [1,2]. Increases in monocyte chemoattractant protein (MCP-1), vascular endothelial growth factors (VEGF), stromal derived factor 1 (SDF-1), erythropoietin (EPO) and brain derived neurotropic factor (BDNF) are associated with BBBD as well as stimulating angiogenesis, neurogenesis and stem cells migration consistent with mild injury following pFUS + MB exposure to the brain. These results indicate that pFUS + MB rapidly effects to the cerebral vasculature as evident by BBBD in addition to the shockwave from MB collapse induces mild stress within various cellular elements in the neurovascular unit.

**References**

[1] Chen, G. Y., and G. Nunez, *Sterile inflammation: sensing and reacting to damage*. Nat Rev Immunol 10(12): pp. 826–37, 2010.

[2] Gadani, S. P., et al., *Dealing with Danger in the CNS: The Response of the Immune System to Injury*. Neuron 87(1): pp. 47–62, 2015.

**Fig. 46 (abstract O40).**
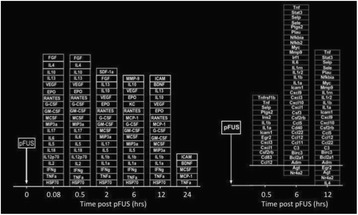
Stackplot of significantly increased expression of both pro- & anti-inflammatory factors over time following pFUS + MB to the brain. Proteomic profile is observed with sterile inflammatory response in the brain (*Left*). Stackplot of significant mRNA >2 fold mRNA expression at various time points (0.5, 6 and 12 hrs) post pFUS + MB in the brain. Peak mRNA expression was observed at 6 hrs post sonication that persisted to 12 hrs (*Right*)

### O41 Pulsed focused ultrasound wave reconstruction and mapping for blood–brain barrier opening

#### Jingjing Xia^2^, Po-Shiang Tsui^1^, Hao-Li Liu^2^

##### ^1^Department of Medical Imaging and Radiological Sciences, Chang Gung University, Taoyuan, Taiwan; ^2^Department of Electrical Engineering, Chang Gung University, Taoyuan, Taiwan

**Objectives**

Burst-mode low-pressure focused ultrasound (FUS) has been shown to induce transient blood–brain barrier (BBB) opening, and has high potential for use in non-invasive and targeted delivery of therapeutic molecules into the brain. FUS-BBB opening requires imaging guidance mean during the intervention, yet current imaging technology only enables postoperative outcome confirmation. In this study, we propose an approach to visualize short-burst low-pressure focal beam distribution that allows to be applied in FUS-BBB opening intervention guidance.

**Methods**

An backscattered acoustic-wave reconstruction method based on synchronization between emission from focused ultrasound and receiving diagnostic ultrasound elements and passively beam formed processing were developed. FUS transducers with the frequency ranging from 0.5-2 MHz were employed, and a commercialized diagnostic ultrasound was synchronously integrated with short-burst FUS excitation (burst length ranging from 0.01 to 10 ms). In-vitro phantom experiments were conducted to evaluate the constructed mapping, to quantitatively analyse its performance, and to evaluate the focal beam reconstruction limit. In vivo experiments with prior MRI anatomical scans were conducted to verify the feasibility of guiding the transcranial FUS-BBB opening procedure as well as its BBB-opened accuracy and reliability on small animals.

**Results**

A focal beam can be successfully visualized at all FUS frequency exposures (0.5 – 2 MHz) without involvement of microbubbles or acoustic cavitation triggering. The detectable level of FUS exposure with 0.467 MPa 0.05 ms single-burst exposure was identified. The signal intensity (SI) of the reconstructions was linearly correlated with the FUS exposure both in-vitro and *in-vivo* (r^2^ both higher than 0.9).

**Conclusions**

We confirmed that focal beam pattern can be visualized and allow successful guidance of FUS-BBB opening in small animals, with the SI level of the reconstructed focal beam correlated with the success and level of BBB-opening. The proposed approach provides a feasible way to perform real-time and closed-loop control of FUS-based brain drug delivery.

**Fig. 47 (abstract O41).**
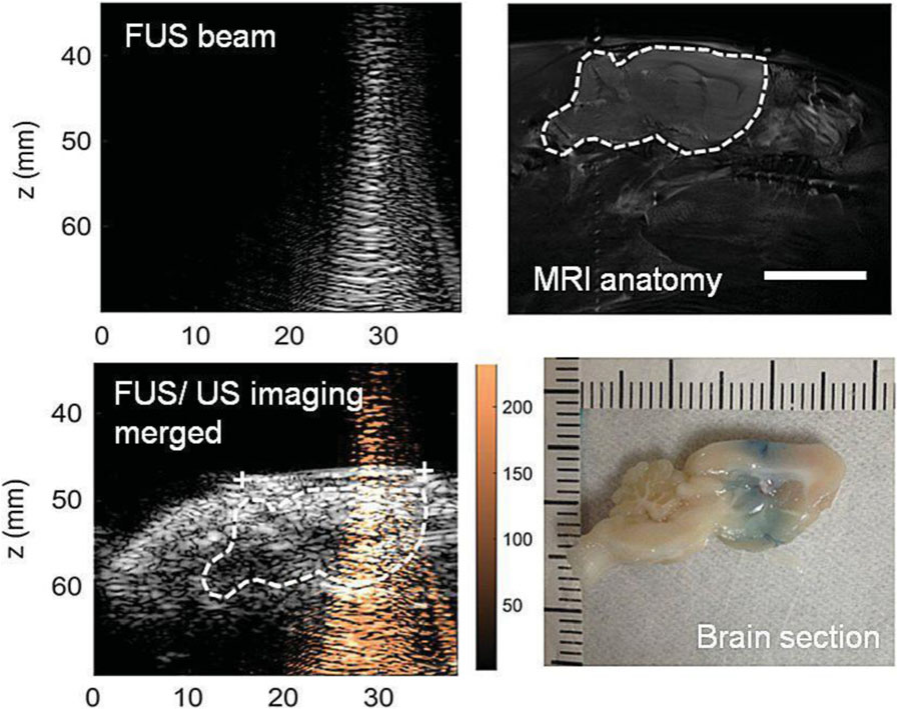
*In vivo* treatment example showing the use of selected FUS exposure level to perform FUS-BBB opening

### O42 Early assessment of mr-guided fus thalamotomy using a diffusion weighted steady state MRI sequence in an in-vivo porcine model

#### Juan C. Plata^1^, Samuel Fielden^2^, Bragi Sveinsson^1^, Brian Hargreaves^1^, Craig Meyer^2^, Kim Butts Pauly^1^

##### ^1^Radiology, Stanford University, Stanford, California, USA; ^2^Bioengineering, University of Virginia, Charlottesville, Virginia, USA

**Objectives**

Diffusion-weighted imaging has been used to evaluate tissues ablated tissues using MR-guided focused ultrasound (MRgFUS), including uterine fibroids, prostate, and brain tissue [1–4]. Quantitative studies in canine prostate found a 36% reduction in the apparent diffusion coefficient (ADC) after either high intensity ultrasound ablation or cryoablation of the prostate, despite differences in histology [4]. More recently we studied the evolution of the ADC decrease and found that the time-course for the onset of ADC decrease after ablation of the canine prostate was inversely correlated to the thermal dose achieved [5]. As a result, areas that saw high levels of thermal dose saw a more rapid irreversible decrease in ADC, making ADC an early marker for loss of tissue viability in the prostate. Diffusion-weighted EPI inside of the InSighTec ExAblate 4000 Neuro System following thalamotomy demonstrates poor image quality. As a result, T2-weighted imaging is the method of choice for lesion detection inside the transducer although it may not be the earliest marker for ablation. More recently, a double echo in steady state (DESS) pulse sequence has been proposed to monitor lesion development. DESS generates two images, the first echo is mostly a gradient echo (GRE), the second echo is mostly a spin echo with some diffusion weighting [7]. The purpose of this work was to investigate the time course of lesion contrast in a pig model of thalamotomy on a diffusion-weighted steady state sequence in comparison to T2-weighted FSE. In addition, we probe the thermal dose dependence of the contrast by evaluating thermal lesions of two different peak temperatures.

**Methods**

MRgFUS thalamotomy was performed in a porcine model (n=2) under MR thermometry guidance. In one lesion in one animal, image collection began approximately 40 minutes after a low peak temperature sonication T_peak_ = 52°C in the thalamus. In a second animal, two high peak temperature lesions T_peak_ = 60°C were created in the thalamus, and image collection began immediately. In all cases, double-echo in steady-state (DESS) and fast spin echo (FSE) T2-weighted imaging acquisitions were interleaved. The parameters for both sequences are summarized in Table 3. Contrast to surrounding tissue was computed for all time points using regions of interest determined after lesion detection.

**Results**

**Fig. 48 (abstract O42).**
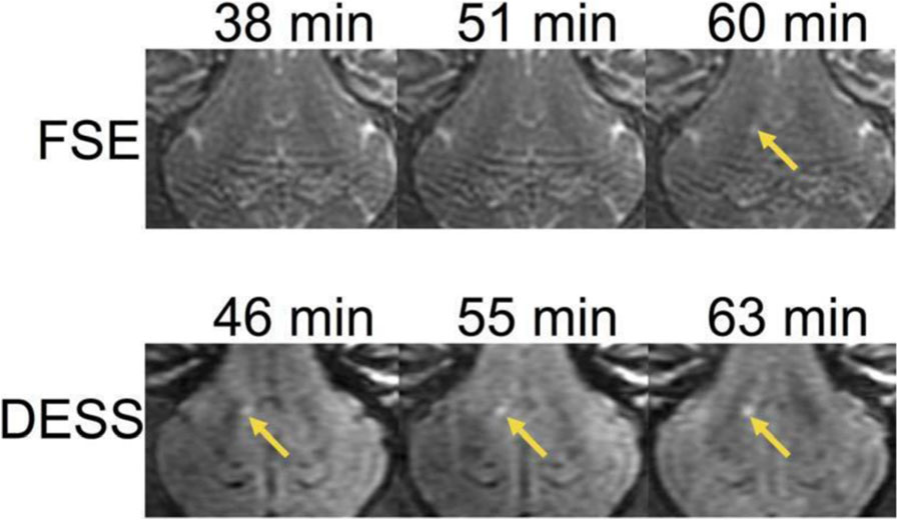
Lesion Detection Using Fast spin echo (FSE) and Double Echo Steady State (DESS) images. Lesion is not visible with FSE until 60 min while the lesion is detected using DESS at 46min

Example images demonstrating the lesion on DESS and on FSE after the lower peak temperature sonication are shown in Fig. 48. The lesion demonstrates higher conspicuity in DESS than FSE.

**Fig. 49 (abstract O42).**
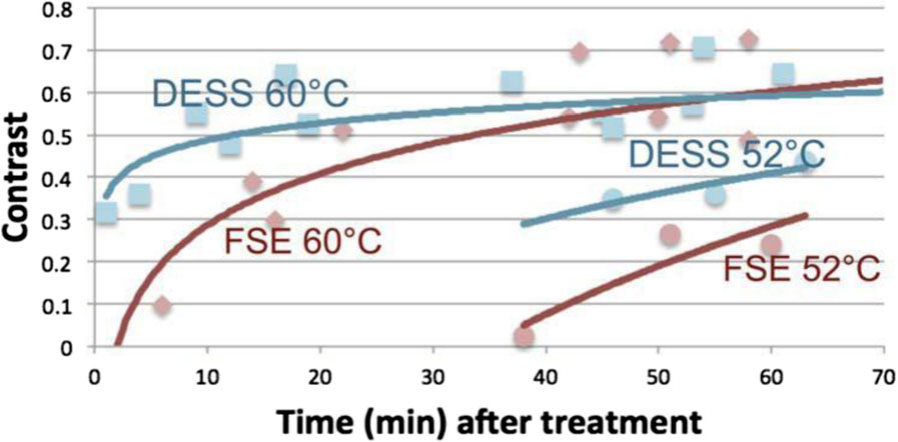
Contrast between lesion and surrounding healthy tissue as a function of time for fast spin echo (FSE) and Double Echo Steady State (DESS) images. Contrast for DESS is initially higher for both treatments indicating DESS can serve as an early indicator of lesion formation in brain treatments

In the quantitative analysis, in all three lesions, DESS provided superior contrast to T2-weighted FSE images at the early time points (Fig. 49), which equilibrated at the later time points. This is presumably due to the mixed diffusion and T2 contrast for the steady state sequence. As edema increases, the steady state sequence loses its advantage over T2-weighted FSE.

Higher peak temperature lesions demonstrate a faster time-course than the lower peak temperature lesion, seen in Fig. 49. In fact, both high temperature sonication lesions were conspicuous within minutes on the DESS sequence.

**Conclusions**

DESS provides a higher contrast between the lesion and the surrounding healthy tissue early after treatment is completed. This will allow for an earlier treatment evaluation while the patient is still in the brain transducer. Future work will include an in-depth simulation analysis on how both the diffusion weighting and T2-weighting contribute to the lesion detection time-course in FSE and DESS.

Acknowledgements

PO1 CA159992, RO1 CA111981, FUS Foundation, UVA-Coulter Translational Research Partnership.

**References**

[1] Jacobs MA, et al. JMRI 2009; 29:649–656.

[2] MacDannold N, et al. Radiology 2006;240:263–272.

[3] Wintermark M, et al. AJNR 2014;35:891–896.

[4] Chen J, et al. MRM 2008;59:1365–72.

[5] Plata J, et al. 2015. Med Phys 2015:09:5130.

[6] Chen L, et al. JMRI 1999;10:146153.

[7] Plata J, et al. 2015. ISMRM:1652.

**Table 3 (abstract O42).** Imaging Parameters

Pulse Sequence	Readout	Flip (°)	TE (ms)	TR (ms)	Miscellaneous
FSE	3D	90	76	2500	ETL=100
DESS	3D	15	4.5/37	20.8	Spoiler=10cyc/voxel

*FSE* Fast Spin Echo, *DESS* Double Echo Steady State, *TE* Echo Time, *TR* Reception Time, *ETL* Echo Train Length

### O43 Monitoring thermal lesion formation with a steady state mri sequence in an in-vivo porcine muscle model

#### Juan C. Plata^1^, Vasant A. Salgaonkar^2^, Matthew Adams^2^, Chris Diederich^2^

##### ^1^Radiology, Stanford University, Stanford, California, USA; ^2^Radiation Oncology, University of California San Francisco, San Francisco, California, USA

**Objectives**

**Fig. 50 (abstract O43).**
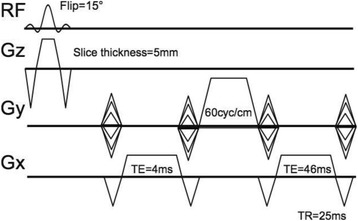
DESS Pulse Sequence Schematic. The Sequence consisted of two echoes a gradient echo (TE = 4 ms) and a spin echo (TE = 46 ms). A spoiler gradients of 60 cycles/cm or 6 cycles/voxel was used

The apparent diffusion coefficient computed from diffusion weighted imaging has been shown to have a 36% signal drop following tissue destruction with high intensity ultrasound and cryoablation [1]. Since this MR contrast mechanism is endogenous, previous studies have looked at monitoring the apparent diffusion coefficient (ADC) [1], or temperature and ADC [2], during treatment in order to study the progression of the ADC signal to assess when tissue viability is lost. More recently, a double echo steady state (DESS) sequence (Fig. 50) has been proposed to achieve a similar goal faster and with improved registration between the temperature images and the lesion monitoring images [3]. Due to its sensitivity to ADC changes and its short TR, DESS could serve as a monitoring platform for both temperature and lesion formation. Since DESS is a steady-state sequence, there are multiple echo pathways that also contribute to the measured signal. The first echo, Echo1, is effectively a gradient echo dominated by free-induction decay from to the preceding RF pulse. Although there are multiple echo pathway contributions for the second echo, Echo2, it is mainly a spin echo of Echo1 of the previous repetition with an effective echo time of TE2=2TR-TE1. The purpose of this work was to assess whether DESS imaging can provide lesion formation information during thermal ablation of in vivo porcine muscle.

**Methods**

In order to assess the DESS sequence in vivo, interstitial ablations were performed within 40–45 kg farm pigs under MRI guidance. All animal experiments were reviewed and approved by our institution’s Administrative Panel on Laboratory Animal Care (APLAC). MRI compatible interstitial ultrasound applicators, consisting of a two element array of 1.5 mm x 10 mm tubular ultrasound transducers, each with independent power control, were used to generate thermal ablative lesions within in vivo porcine muscle. The ultrasound applicators, with integrated water-cooling, were inserted within 13g (2.4 mm OD) Celcon plastic implant catheters which were placed free-hand 10–12 cm deep into the inner thigh muscles. For each experiment, two distinct thermal lesions were planned. For the first trial, thermal ablation was performed using the distal transducer only; after imaging and allowing time for the cool-down, the ultrasound applicator was translated back 2 cm within the stationary catheter for repositioning prior to the second trial. A second thermal lesion was then produced using the proximal element. This translation and sequencing from distal to proximal element were used to isolate the thermal lesions. All ablative sonications (n=4) were 10 min in length, with approximately 5–6 W acoustic power at 7.45 MHz delivered. Applicator and sonication parameters were chosen to generate circumferential lesions with an extent of approximately 1cm away from the active transducer segment. A Double Echo Steady State (DESS) sequence was developed using HeartVista’s SpinBench and RTHawk platforms in order to monitor thermal lesion development. The phase of the first echo of the image was used to obtain temperature information using PRF thermometry while the magnitude of the second echo was used to detect lesion induced changes in signal intensity DESS magnitude images. At the end of the treatment DESS magnitude images were compared to contrast enhanced (CE-MRI) and gross histology.

**Results**

**Fig. 51 (abstract O43).**
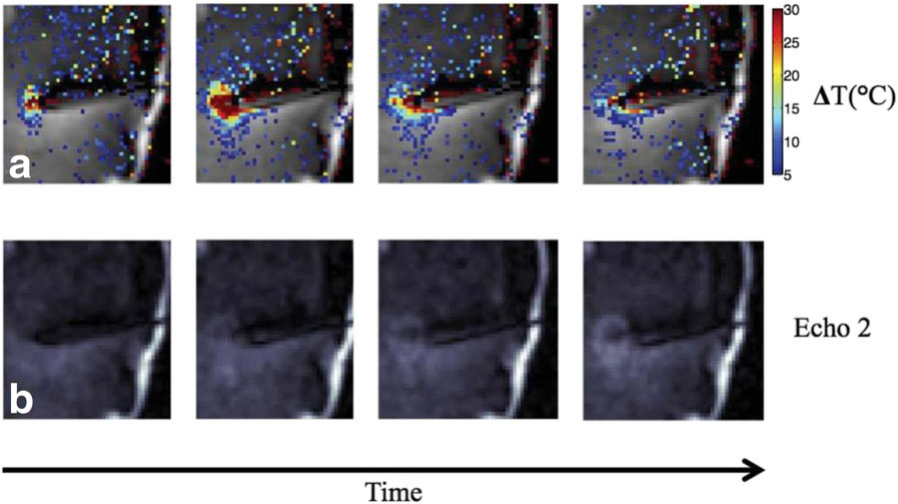
DESS Lesion Development. Magnitude images with a temperature change overlay are shown on the (row **a**) and magnitude images in the second echo (row **b**) are presented. Lesion development is clearly noticeable in Echo 2 and is present early during heating with contrast becoming stronger over time

**Fig. 52 (abstract O43).**
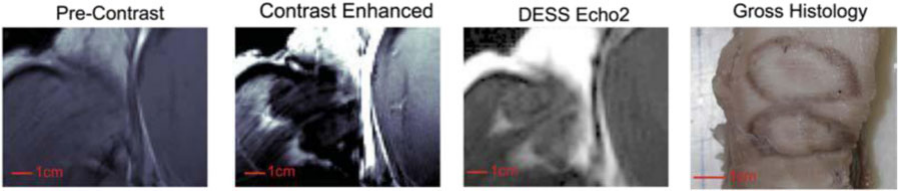
Lesion Extent Comparison. Magnitude images before and after contrast are compared to the magnitude image of the final Echo 2 image prior to contrast injection and gross histology

Temperature information was successfully obtained from the first echo, and the lesion was monitored using the second echo (Fig. 51). Lesion extent obtained from the magnitude of the second echo correlated well with CE-MRI and gross histology (Fig. 52).

**Conclusions**

Lesion formation was visible using the proposed DESS sequence, allowing for more direct lesion monitoring during treatment.

**Acknowledgements**

*PO1 CA159992, RO1 CA111981, GE Healthcare.*

**References**

[1] Chen J, et al. MRM 2008; 59: pp. 1365–72.

[2] Plata J, et al. 2015. Med Phys 2015:09:5130.

[3] Plata J, et al. 2015. ISTU Annual Meeting Abstract: 2173929.

### O44 T2-based temperature monitoring in bone marrow for mr-guided focused ultrasound

#### Eugene Ozhinsky, Matthew D. Bucknor, Viola Rieke

##### Radiology and Biomedical Imaging, University of California San Francisco, San Francisco, California, USA

**Objectives**

MR-guided focused ultrasound (MRgFUS) is a non-invasive technique for the treatment of painful bone metastases. Proton resonant frequency shift (PRF) thermometry is the standard method for monitoring temperature during MRgFUS interventions. It can precisely measure the changes in temperature in water-based tissues, but fails to detect temperature changes in bone and in tissues with high lipid content, such as bone marrow.

Current clinical protocols for bone treatments rely on measurement of the temperature change of adjacent muscle to estimate the temperature of the bone. This approach carries a significant risk of overtreatment in that more energy might be used than is needed to ablate the target. In fact, we observe in HIFU treatments of bone metastases that the highest temperature in soft tissue is only reached 10–15 seconds after the end of the sonication. Collateral treatment of the near-field soft tissues during MRgFUS increases the risk for muscle and vascular injury, which can result in significant perioperative or chronic pain.

Deeper penetration of the ablation through the cortical bone into the bone marrow or tumor is often desired for local control of osseous lesions. In the treatment of osteoid osteomas, complete ablation of the nidus is required for pain relief and to avoid recurrence, but the thickened cortical bone makes ultrasound penetration difficult. Therefore, temperature measurement within the bone is desirable.

Previous studies have shown a change in T2 of subcutaneous fat, red and yellow bone marrow in controlled calibration experiments and during treatments with focused ultrasound (Ozhinsky, et al. J Ther Ultrasound 2015; Baron et al. Magn Reson Med 2014). The goal of this study was to determine if T2 based thermometry could be used to monitor the temperature change in ex-vivo and in-vivo bone marrow during focused ultrasound ablation of intact bone.

**Methods**

All experiments were performed using an ExAblate 2100 system (InSighTec, Haifa, Israel) integrated with a 3.0 Tesla MR scanner (GE Healthcare, Waukesha, WI, USA). Bone marrow T2 was quantified with a double-echo fast spin-echo sequence with water suppression (TE = 35/186 ms, TR = 1500 ms, echo train length = 40, FOV = 32 cm, 128 x 128 matrix size, 10mm slice thickness, 15 sec/slice).

For ex-vivo validation, we performed MRgFUS ablation in an ex-vivo porcine femur (sonication: 20 sec, acoustic power: 30 W). The focus of the sonication was placed in the middle of the marrow, but due to the high ultrasound absorption of cortical bone most of the energy is absorbed in the cortical bone.

Focused ultrasound ablation was also performed in a swine model. All experimental procedures were done in accordance with NIH guidelines for humane handling of animals and received prior approval from the local Institutional Animal Care and Use Committee. Each of the three animals received 12–14 sonications on femur and ilium bones (acoustic power: 10-35W, duration: 20–40 sec). As in the ex vivo validation, the focus was placed in the middle of the marrow. At the end of the focused ultrasound, pre- and post-contrast 3D Fast SPGR images were acquired.

**Results**

**Fig. 53 (abstract O44).**
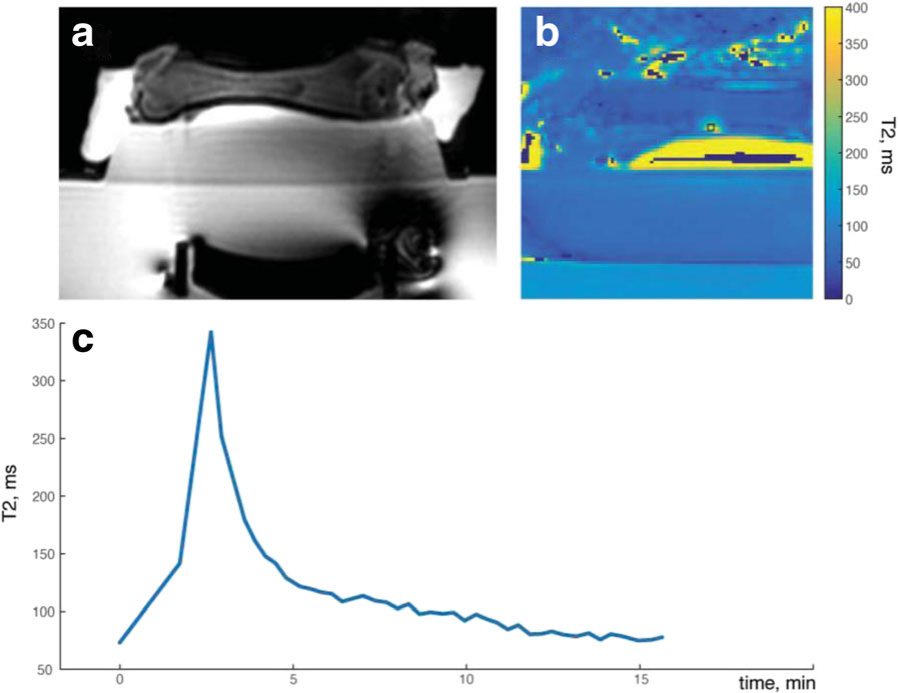
T2 Measurement in ex-vivo bone marrow during and after the heating: **a** Localizer image showing the ultrasound transducer in the table; **b** T2 map during heating, showing the ROI; **c** Plot of T2 values within the ROI over time

Figure 53 shows the results of the ex-vivo experiment, where we measured a T2 elevation of 269 ms. Assuming the T2/temp coefficient of 7 ms/°C (Ozhinsky, et al. ISMRM 2014), this corresponds to a temperature rise of 38°. The ex-vivo experiment shows that it takes on the order of 15 minutes for the marrow to return to the baseline temperature.

**Fig. 54 (abstract O44).**
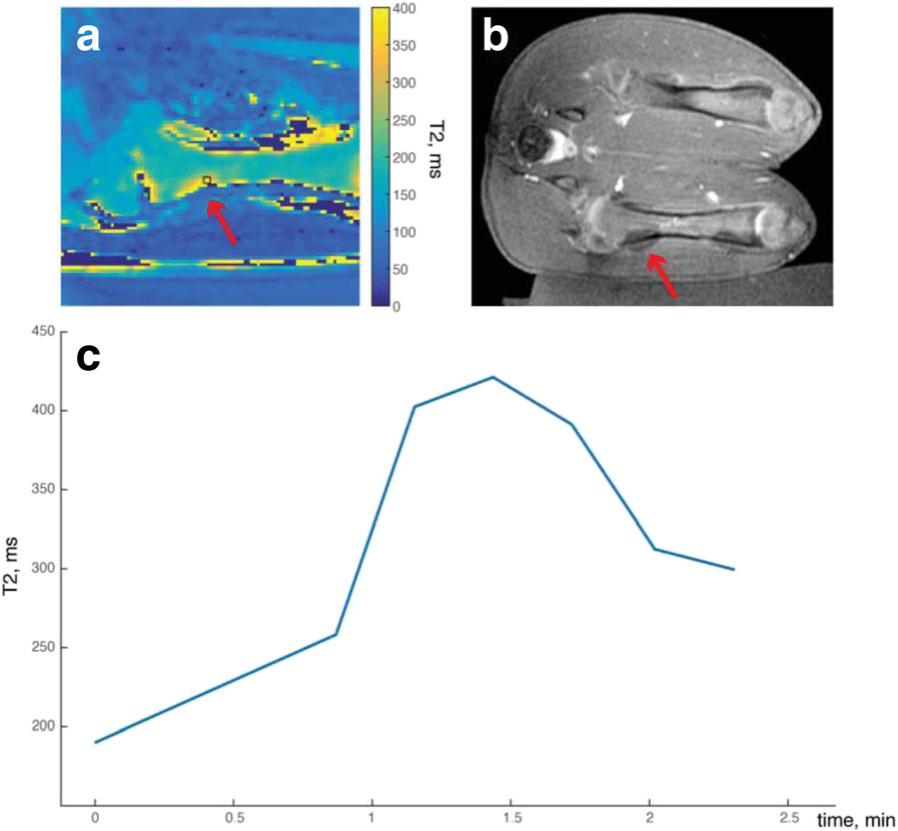
T2 Measurement in in-vivo bone marrow: **a** T2 map during ablation of a single sonication, showing the ROI; **b** post-contrast 3D Fast SPGR image after ablation (total of six sonications per location) **c** plot of T2 values within the ROI over time

Figure 54 shows the results of the in vivo experiment in a swine model. We measured a T2 rise of 231 ms within the bone marrow, which corresponds to temperature change of 33°C from baseline. The in-vivo experiment showed excellent correspondence between the area of T2 elevation in marrow during the ablation and the resulting non-enhancing area in the post-contrast images.

**Conclusions**

In this study we have demonstrated for the first time that T2-based thermometry can be used in vivo to measure the heating in the marrow during bone ablation. The ability to monitor the temperature within the bone marrow allowed visualization of the heat penetration into the bone, which is important for local lesion control and treatment of osteoid osteomas. Therefore, T2 based temperature mapping, in addition to PRF-based thermometry could be used to monitor heating during the bone focused ultrasound treatments and improve safety and efficacy of MRgFUS bone applications.

### O45 Tissue-mimicking thermochromic phantom for characterization of hifu devices, heating methods, and sonication parameters

#### Ari Partanen^1,2^, Andrew Mikhail^2^, Lauren Severance^2^, Ayele H. Negussie^2^, Bradford Wood^2^

##### ^1^Clinical Science MR Therapy, Philips, Andover, Massachusetts, USA; ^2^Center for Interventional Oncology, Department of Radiology and Imaging Sciences, Clinical Center, National Institutes of Health, Bethesda, Maryland, USA

**Objectives**

Tissue mimicking phantoms (TMPs) are routinely used for calibration and quality assurance of medical devices including thermal therapy applicators prior to their use in clinic. TMPs are also used in thermal therapy research as alternatives to ex vivo soft tissues and organs as they possess several advantages including greater availability and shelf life, high uniformity, and customizability. The efficacy of thermal ablation therapies depends on several factors including targeting accuracy and temperature elevation in the treated tissue. Thus, an ideal TMP for thermal therapy applications should have the capacity to report ablated volumes and geometries as well as absolute temperatures. Magnetic Resonance Imaging -guided High Intensity Focused Ultrasound (MR-HIFU) is a therapeutic technique that can be used to precisely target and heat tissue non-invasively to induce thermal ablation or mild hyperthermia, among other applications. The objective of this study was to develop a novel, MR compatible tissue-mimicking thermochromic (TMTC) phantom for studying and characterizing HIFU devices, heating methods, and sonication parameters. Specifically, the intent of this work was to develop a phantom that reports on targeting accuracy, thermal energy deposition, and spatial heat distribution following HIFU. Additionally, the objective was to employ the TMTC phantom in characterization of two different HIFU devices, and to assess the temperatures and distribution of heating post-HIFU in relation to the treatment plan.

**Methods**

Polyacrylamide gel phantoms containing silica particles (1.0% w/v), bovine serum albumin (BSA, 3% w/v), and thermochromic ink (5.1% v/v, colour change temperature threshold of 60 °C) were produced. Both a preclinical Therapy Imaging Probe System (TIPS, Philips Research, Briarcliff Manor, NY) and a clinical MR-HIFU system (Sonalleve V2, Philips, Vantaa, Finland) were used for HIFU exposures targeted within the TMTC phantoms. The TIPS system contains an 8 element annular array with an 80 mm focal length, as well as a 2-axis motion control system to move between targets. The Sonalleve system contains a 256-element phased array transducer (focal length = 140 mm), as well as a motion control system with 5 degrees of freedom. HIFU exposure parameters for the TIPS were: frequency 1.0 MHz, acoustic power 30 W, with sonication durations of 60, 120, and 180 s. HIFU exposure parameters for the Sonalleve were: frequency 1.2 MHz, acoustic power 100 W, and duration 20–70 s, targeted to regions of 4–16 mm in diameter using electronic steering of the focal point. Together with the Sonalleve system, a clinical 1.5T MR scanner (Achieva, Philips Healthcare, Best, the Netherlands) was used for exposure planning and real-time thermometry utilizing the proton resonance frequency shift (PRFS) method. In addition, T2-weighted MR imaging and quantitative T2 mapping were performed to visualize and characterize thermal lesions within the TMTC phantoms after both Sonalleve and TIPS sonications. Post-MRI, HIFU-induced colour changes within the phantoms were identified, photographed, and compared to the sonication plan as well as to MRI T2 and temperature maps.

**Results**

Tissue-mimicking thermochromic phantoms were developed, produced, and validated for use in characterizing HIFU devices and sonication methods. HIFU thermal ablations (maximum temperature > 60 °C) resulted in permanent colour changes at targeted locations within the phantoms. These colour changes corresponded to maximum temperatures recorded using real-time MRI thermometry. A gradual colour change from yellow to magenta was visible between 40 °C and 64 °C. At temperatures above 64 °C, no further colour change was observed. In addition, heated volumes were visible on T2-weighted MRI and T2 maps as regions of permanent hypointensity and of lower T2 values, respectively, due to BSA coagulation. Increased HIFU energies and target diameters lead to greater colour change and a larger volume of colour change, respectively, as well as, correspondingly, to greater absolute change in T2 and a larger region of T2 change.

**Conclusions**

A tissue-mimicking thermochromic phantom was developed to assess the spatial targeting accuracy, maximum temperatures, and temperature uniformity of HIFU exposures. This TMTC phantom changes colour (over a range of temperatures that is relevant to ablative HIFU procedures) upon heating, allowing for quantitative measurements of absolute temperature and delineation of heated regions, and thus may be useful in HIFU device characterization, parameter optimization, quality assurance, and user training. TMTC phantoms can also provide volumetric temperature information in experiments where MRI-based real-time thermometry is not feasible, as the stepwise nature of the colour change associated with changes in temperature allow for assessment of temperature gradients within and at the periphery of the heated region.

**Fig. 55 (abstract O45).**
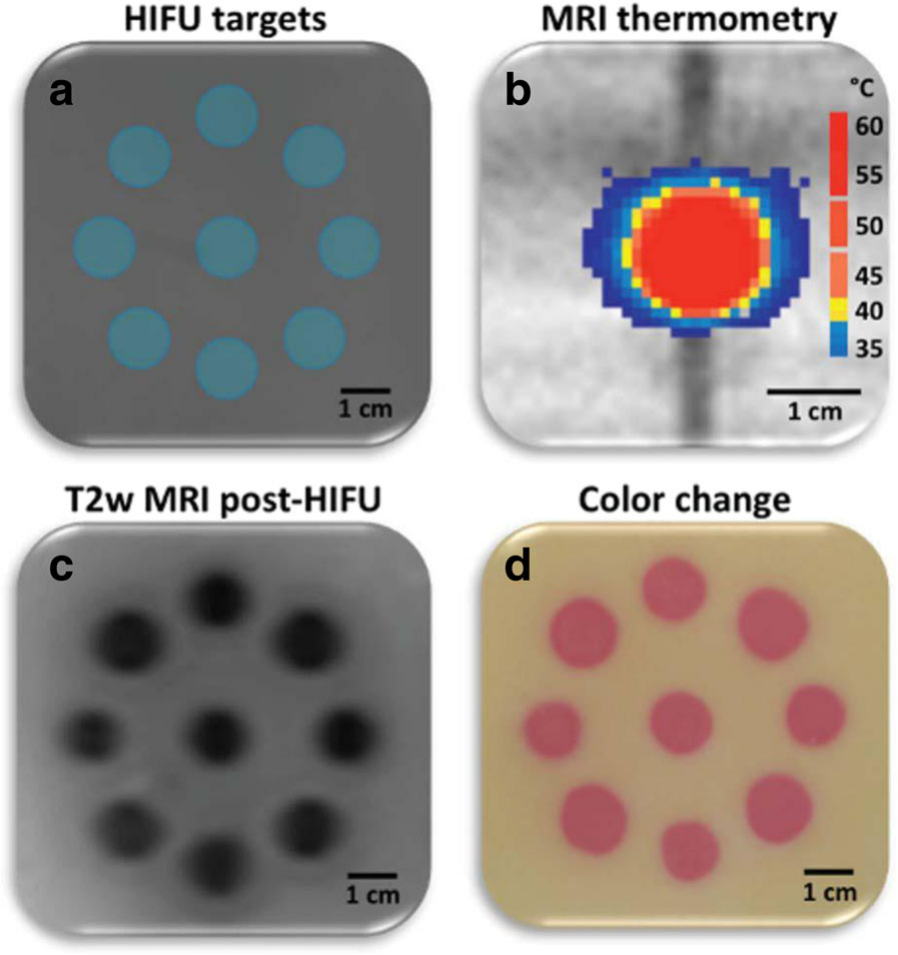
**a** HIFU target planning within a TMTC phantom performed on T1-weighted MR images using the Sonalleve therapy planning software. Each of the nine target locations is 12 mm in diameter. **b** HIFU thermal ablations produced temperatures above 60 °C as seen on real-time MRI thermometry. A coronal temperature map for a single sonication location is shown. **c** Intensity changes (due to BSA coagulation) on T2-weighted MRI with high spatial accuracy relative to the treatment plan. **d** HIFU thermal ablations resulted in permanent colour changes at the targeted locations within the TMTC phantom, correlating with the T2 changes and MR thermometry, and with high spatial accuracy relative to the treatment plan

### O46 Reduced field-of-view MR thermometry in adipose tissues using zoomed apparent T2-mapping

#### Martijn de Greef^2^, Gerald Schubert^1^, Chrit Moonen^2^, Mario Ries^2^

##### ^1^Philips Healthcare, Vantaa, Finland, ^2^Imaging Division, University Medical Center, Utrecht, Utrecht, Netherlands

**Objectives**

During HIFU ablation of abdominal and pelvic lesions, a balance needs to be established between ablation speed and heat accumulation in the pre-focal subcutaneous layers, resulting in a safe but time-efficient intervention. As the de facto standard form of magnetic resonance thermometry, which is based on the proton resonance frequency shift, is ill-suited for temperature monitoring in the adipose tissues, several alternatives have been proposed. Baron et al. (Baron, MRM, 2014) have demonstrated the feasibility of monitoring heating in adipose tissue layers using apparent T2 mapping based on dual echo fast spin echo imaging. A linear (5.2 ms/○C) and reversible T2 - temperature dependency was shown reproducible over a relevant temperature range.

In this study, the strategy developed by Baron et al. was combined with reduced field-of-view (FOV) imaging using perpendicular selection gradients, in the literature referred to as zonally-magnified or local-look imaging (Mansfield, J. of Phys.,1988). Reducing the FOV in phase-encoding (PE) direction while preventing fold-over allows to improve the spatio-temporal resolution of apparent T2-based thermometry. This opens new possibilities such as near-field monitoring during sonication at relevant temporal resolutions using sequence interleaving but also high resolution inter-sonication monitoring of accumulative heating.

**Methods**

All imaging experiments were performed on a 1.5-T MR Scanner (Achieva, Philips Healthcare, Best, The Netherlands) using a research version of the Sonalleve HIFU Platform (Philips Healthcare, Vantaa, Finland) that is supplied with an integrated four-channel loop coil and a 16-channel back coil.

Two imaging experiments were performed in this study. Under normothermic conditions, a volunteer was imaged using a dual echo fast spin echo sequence, with a single slice placed at the location of the subcutaneous fat layer (coronal orientation, TE1/TE2: 11/140 ms, train length: 24 lines/excitation, FOV: 400 x 105 mm2, matrix size: 168 x 48). The purpose of these experiments was to demonstrate the feasibility of apparent T2-mapping using a reduced FOV in PE direction.

A next experiment aimed at demonstrating the feasibility of resolving a temperature gradient along the subcutaneous fat layer of a volunteer, comparing a cooled and slightly heated state. Heating (37○C skin temperature) and cooling (14○C skin temperature) were achieved using a water-filled cushion together with a circulation unit, which was placed outside of the MR room. Skin temperatures were monitored using a fibre optic probe (Luxtron, LumaSense, Santa Clara, CA), placed between skin and cushion. Imaging parameters were the following: single slice, sagittal orientation, TE1/TE2: 11.8/130 ms, train length: 20 lines/excitation, FOV: 200 x 52 mm2, matrix size: 200 x 40.

In both imaging experiments, the radio-frequency pulse generating the initial transverse magnetization was implemented as a fat-selective binomial pulse (1-2-1) and the corresponding selection gradient was oriented parallel and perpendicular to the imaging slice, respectively. As a consequence, the volume experiencing the spectrally non-selective refocussing pulses was limited to the subcutaneous tissue layers. This aims at preventing disturbance of the magnetization in the water compartment at the location of the focus.

**Results**

**Fig. 56 (abstract O46).**
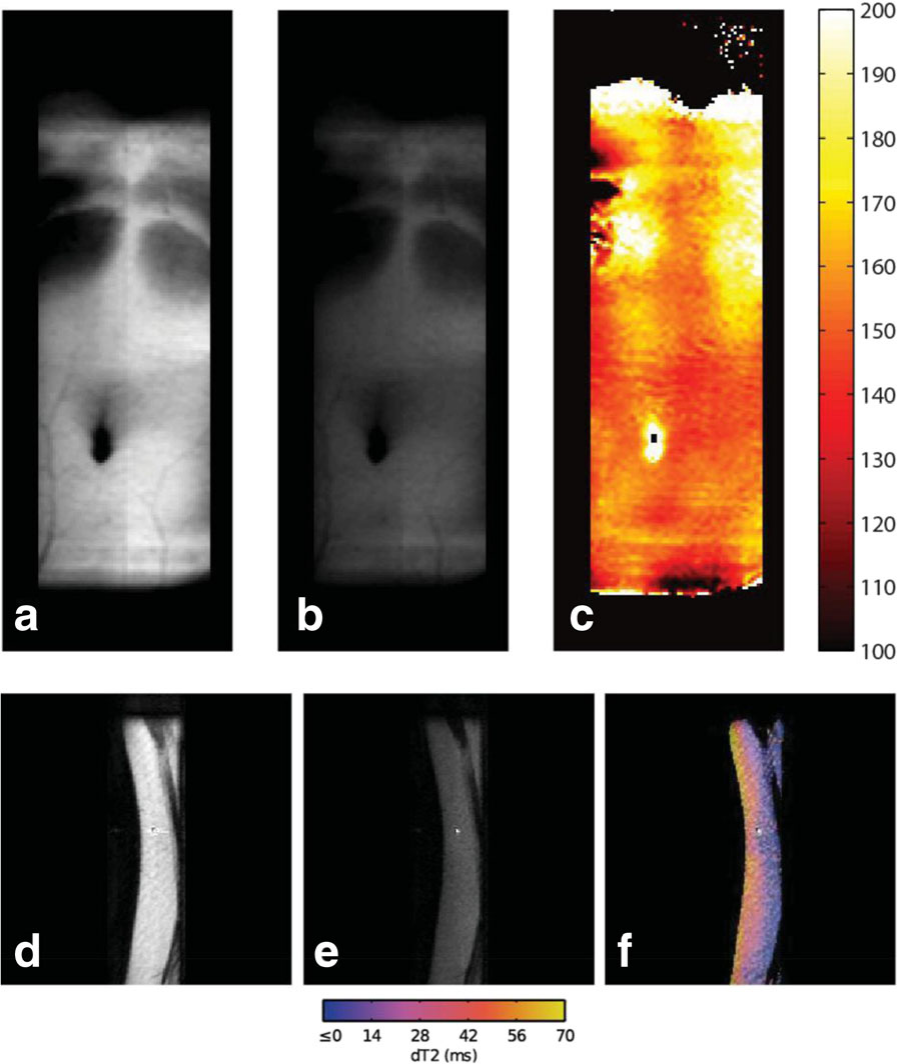
Overview of the result obtained in the two imaging experiments. The results obtained under normothermic conditions are shown in panels **a**-**c**, where panel a shows the signal magnitude at the first echo time, panel b the signal magnitude at the second echo time, and panel c the apparent T2. Panels d-f give an overview of the results obtained in a sagittal slice under cooled and slightly heated conditions. Panel **d** and **e** show again the signal magnitude at the first and second echo time, respectively, while panel **f** shows the difference in apparent T2 between the heated and cooled state

An overview of the results of the imaging experiment under normothermic conditions is shown in Fig. 56(a-c). Reduction of the FOV in phase-encoding direction (left-right in the image) was achieved without apparent fold-over artefacts.

Figure 56(d-f) shows the signal intensity at the first echo time (panel d), the signal intensity at the second echo time (panel e) and the difference in apparent T2 between the heated and cooled state as an overlay (panel f, background: intensity at the first echo time). A gradual change along the anterior-posterior direction (left-to-right in the image) is observed in the difference of the apparent T2.

**Conclusions**

Dual echo fast spin echo imaging in combination with reduced FOV imaging using perpendicular selection gradients was successfully shown to allow for apparent T2-mapping with no/minimal fold-over artefacts under normothermic conditions. Furthermore, comparing a cooled and slight heated state, a gradient in the apparent T2 difference could be resolved at high resolution (~1mm). Both examples show the potential of reduced FOV apparent T2-mapping, enabling near-field monitoring at improved spatio-temporal resolution. The potential interference in interleaved T2/PRFS imaging scenarios is currently under investigation.

### O47 Time-resolved *in vivo* measurements of FUS immunomodulation in a novel reporter mouse model of breast cancer

#### Megan E. Poorman^1,2^, Mary Dockery^1^, Vandiver Chaplin^3,2^, Stephanie O. Dudzinski^1^, Ryan Spears^1^, Charles Caskey^4,2^, Todd Giorgio^1^, William Grissom^1, 2^

##### ^1^Biomedical Engineering, Vanderbilt University, Nashville, Tennessee, USA; ^2^Institute of Imaging Science, Vanderbilt University, Nashville, Tennessee, USA; ^3^Chemical and Physical Biology, Vanderbilt University, Nashville, Tennessee, USA; ^4^Radiology, Vanderbilt University, Nashville, Tennessee, USA

**Objectives**

Focused Ultrasound (FUS) therapy is a promising approach for treating cancerous lesions in the body. In addition to cell destruction, FUS hyperthermia has been shown to have immunomodulatory effects, increasing dendritic cell infiltration and activating the body's immune response to reduce metastases and future recurrence [1–2]. However, the development and understanding of FUS immunomodulation has been limited by an inability to characterize the immune response *in vivo*. This ability would enable optimal timing of *ex vivo* immunophenotyping, resulting in more efficient and more statistically powerful studies with fewer animals. Here we describe and validate a novel double-transgenic murine model of breast cancer that we have developed to meet this need.

**Methods**

A double transgenic PyNGL murine model was bred to express a nuclear factor-kappaB (NF-kB) reporter transgene (NGL) into the polyoma virus middle T oncogene model. This mouse exhibits spontaneous mammary tumour formation comparable to that of human breast cancer as well as a spatially-resolved, detectable change in bioluminescence with NF-kB activation, a key factor in immunomodulatory inflammation. By monitoring bioluminescence with *In Vivo* Imaging Systems (IVIS) imaging, tissue collection for immunophenotyping can be optimally timed. Experiments were performed to validate the mouse model when treated with FUS. Baseline luminescence maps were first obtained with IVIS prior to treatment. Subsequently, FUS thermal treatment was applied with either hyperthermia (CEM43 < 20) or ablative (CEM43 > 200) doses with a custom-designed MRgFUS system built in-house [3]. IVIS was used to monitor the change in luminescent inflammatory response every 12 hours after undergoing thermal therapy until the collection of tissues. This enabled the time of maximum immune response post-FUS treatment to be localized. Subsequent mice were sacrificed 48 hours post-treatment (the optimum time point based on the IVIS data) and analyzed with flow cytometry for infiltration of immune markers such as T cells (CD3,CD4,CD8), cytokines, and macrophages. Histology sections were taken of the skin, tumours, and spleen to assess cellular damage and composition (results not shown).

**Results**

**Fig. 57 (abstract O47).**
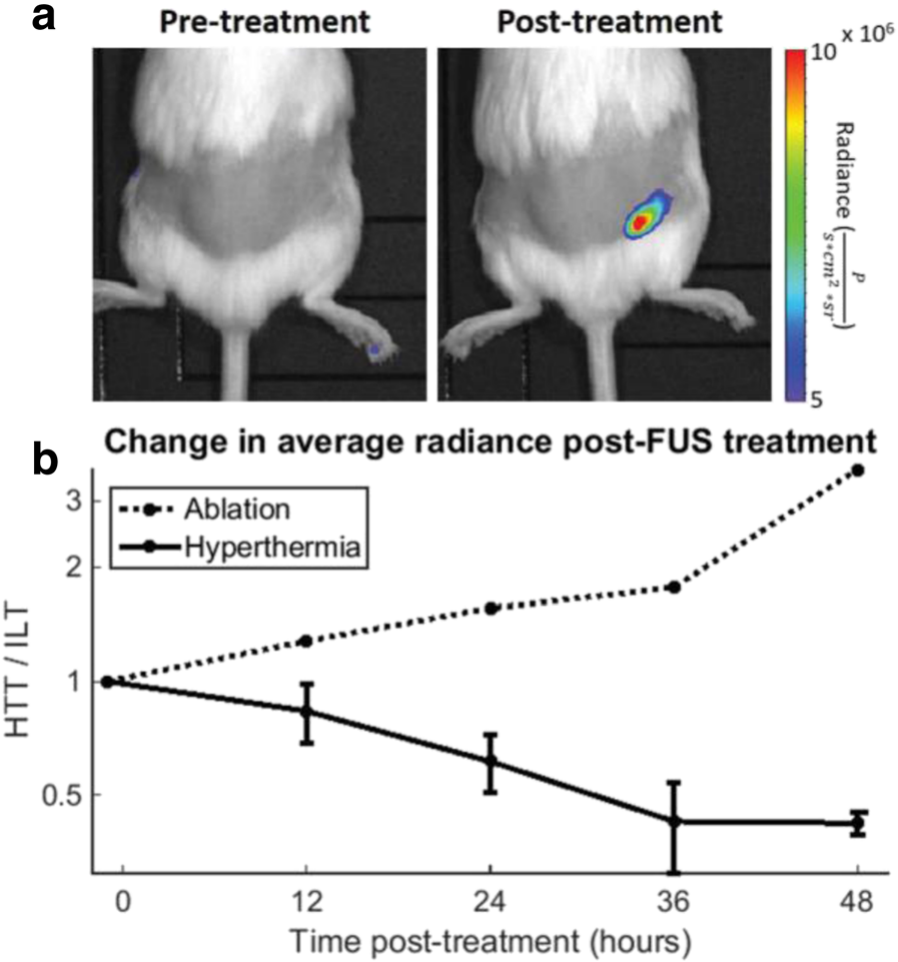
IVIS data from MRgFUS treatment of mice. **a** Sample IVIS images of an NGL mouse (no tumours) showing a spatially-localized increase in NF-kB activity post-treatment consistent with where the MRgFUS was applied. **b**) Ratio of the average radiance of the FUS-treated tumour (HTT) to the ipsilateral tumour (ILT) over time plotted on a log scale. Two distinct dose-dependent treatment groups can be observed. Both treatments result in a large difference in NF-kB activity from baseline at 48 hours post-treatment

**Fig. 58 (abstract O47).**
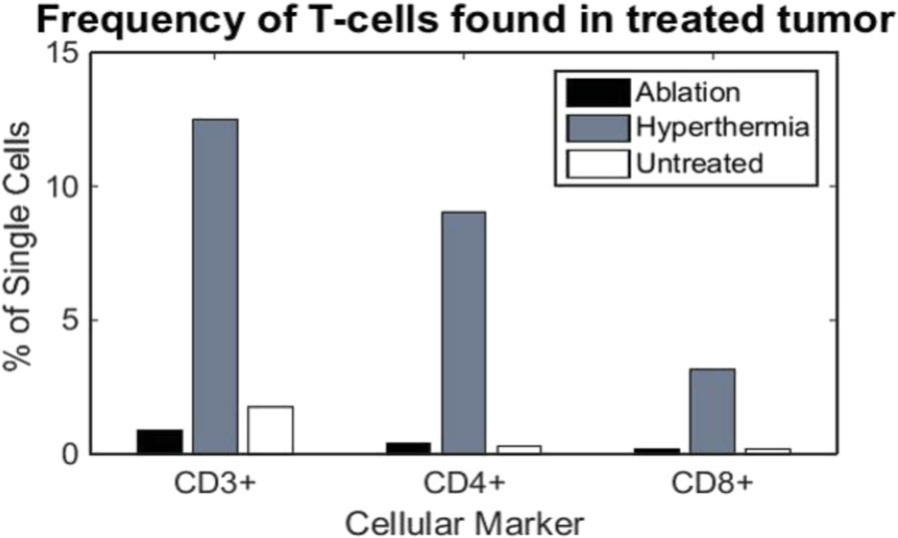
A preliminary cellular analysis of treated tumours excised 48 hours post MRgFUS treatment and analyzed with flow cytometry. CD3+, CD4+, and CD8+ are cellular markers for T-cells that are involved in the in vivo immune response. A substantial difference in immune cell infiltration can be observed between the treatment groups

For both treatment groups, a change NF-kB activation was observed with IVIS as soon as 24 hours post-treatment and reached a peak between 48 and 96 hours, consistent with the anticipated timeline for recruitment of inflammatory immune cells. Activation was spatially consistent with the area of MRgFUS treatment (Fig. 57a) and remained above baseline activation levels for the duration of luminescent imaging. The varied thermal doses were shown to have different effects on NF-kB activation - hyperthermia resulted in a consistent decrease in activation in the treated tumour (n=3) while ablation resulted in an increase in activation (n = 1) (Fig. 57b). Additional mouse studies are currently underway to confirm these results. Immunophenotyping revealed a large influx of T-cells 48 hours post-treatment in response to hyperthermia in comparison to the untreated control. Analysis of the ablated tumour tissue revealed no significant change in immune cell concentration compared to the control. (Figure 58). No superficial skin burns were observed on the treated mice in the area of applied MRgFUS treatment.

**Conclusions**

The use of a novel transgenic reporter mouse with spontaneous tumor generation enables spatiotemporally-resolved quantification of the immune response to FUS treatment *in vivo*. Cell analysis from excised tissue was supplemented by spatially-localized monitoring of the *in vivo* inflammatory immune response. Preliminary results showed that immune modulation measured by NF-kB activation depends on thermal dose. The increase in infiltrating T cells with hyperthermia, and lack thereof in the ablation case, suggests that the immune response may be more effectively activated by FUS treatment at a lower thermal dose. More experiments are ongoing to further explore the difference between thermal doses varying from hyperthermia to ablation as well as long term studies to investigate the effect of FUS-induced immune activation on metastases outside of the primary tumour.

**Acknowledgments**

*This work was supported by DoD W81XWH-12-BCRP-IDEA, NIH RO1 CA111981, and a Vanderbilt University Discovery Grant.*

**References**

[1] F Wu, Z Hu *et al.* Journal of Translational Medicine 5(34), 2007

[2] J Unga *et al.* Advanced drug delivery reviews 72:144–153, 2014

[3] M Poorman *et al.* ISMRM 2015 #1642

### O48 Targeting tumour hypoxia with HIFU: a promising new adjuvant cancer therapy

#### Marcia M. Costa, Efthymia Papaevangelou, Anant Shah, Ian Rivens, Carol Box, Jeff Bamber, Gail ter Haar

##### The Institute of Cancer Research, Department of Physics, Sutton, United Kingdom

**Objectives**

Hypoxia is a common feature of radioresistant tumours, resulting in a decreased efficiency of radiotherapy (Harada, 2011). Several approaches have been proposed to overcome this limitation, including the adjuvant use of hyperthermia and chemotherapy. We propose the use of High Intensity Focused Ultrasound (HIFU) to target these hypoxic regions, which lack perfusion and so provide good targets for thermal ablation.

One of the challenges when targeting hypoxia is the accurate detection of poorly oxygenated regions, ideally non-invasively, for treatment planning. Several imaging techniques may be used in clinical practice for this purpose. Recently Photoacoustic (PA) imaging has been proposed for distinguishing between oxy- and deoxy-haemoglobin inside tumours. The technique is based on the generation of acoustic waves by the tissue after being exposed to short pulses of light. These waves can be detected using a transducer and the distribution of optical absorption in tissue is then reconstructed with US spatial resolution.

For this study, we used PA imaging to detect hypoxia in a radioresistant head and neck tumour model, implanted subcutaneously in mice. Selected hypoxic regions were targeted with HIFU, under US-guidance, using a dedicated small animal system, and the outcome of the treatment was evaluated histologically.

**Methods**

Twelve female NCr nude mice (6 weeks old) were subcutaneously injected with 5x10^5^ CAL^R^ cells (head and neck tumour model) in the right flank. Tumours were measured up to 3 times a week, until they reached a volume of 200–300 mm^3^.

Animals were imaged using a MultiSpectral Optical Tomography (MSOT, iThera Medical) device with an excitation wavelength range of 660–1350 nm (details in Morscher, 2014). Coupling gel is applied to the tumour and surrounding area, and the animal is placed in a horizontal position in a holder under isoflurane anaesthesia. Furthermore, they are enveloped in a thin polyethylene membrane as it provides acoustic coupling, before being submerged in a water tank. Multiple transverse 2D slices of the tumour were acquired at 680, 700, 715, 730, 760, 770, 800, 850 and 900nm, in 0.5mm steps (from head to tail), and were reconstructed using interpolated model-matrix inversion (Rosenthal, 2010). The reconstructed data were multispectrally unmixed using a linear regression technique to identify the distribution of oxy and deoxygenated haemoglobin.

Animals were US imaged 24h after PA imaging (injectable anaesthesia: mix of hypnovel, fentanyl and medetomidine), using an E-cube scanner with a phased array transducer (f=12MHz). Animals were imaged in the same direction as in the PA experiment, with a 0.5mm step between each image acquisition. For the HIFU exposures, a pre-clinical VIFU2000 (Alpinion) system was used (single element spherical-focused transducer, 1.5MHz). Although hypoxic regions are not visible in B-mode images, they are generally distributed around areas of necrosis that can be identified by their hypoechogenicity. We compared these regions with those in PA images to define the target regions, which were exposed using the VIFU system at different acoustic power levels in order to define the thermal lesioning threshold for these tumours. Six animals were HIFU-exposed, at 31.1 +/− 3.1 W (N=2), 26.3 +/− 2.6 W (N=3) and 23.0 +/− 2.3 W (N=1), for 8 seconds, one exposure per tumour. In addition, a Passive Cavitation Detection (PCD) system consisting of a Precision Acoustics broadband sensor, 20 mm outer diameter, weakly focused co-aligned to the HIFU focal peak and connected to a data acquisition system (Spectrum MI.2031, 8-bit) via a 1.5MHz notch filter. A 20dB pre amplifier was used to record data at a rate of 12x1.5MHz in 5 of the treatments. Broadband activity between 3-9MHz was analysed to detect inertial cavitation. Animals were allowed to recover for 24h after HIFU, at which point an i.p. injection of pimonidazole (a marker for hypoxia) was given and they were sacrificed 45min later. Tumour samples were collected, snapped frozen in cardice and stored at −80°C for histological analysis, which included pimonidazole and H&E staining.

**Results**

**Fig. 59 (abstract O48).**
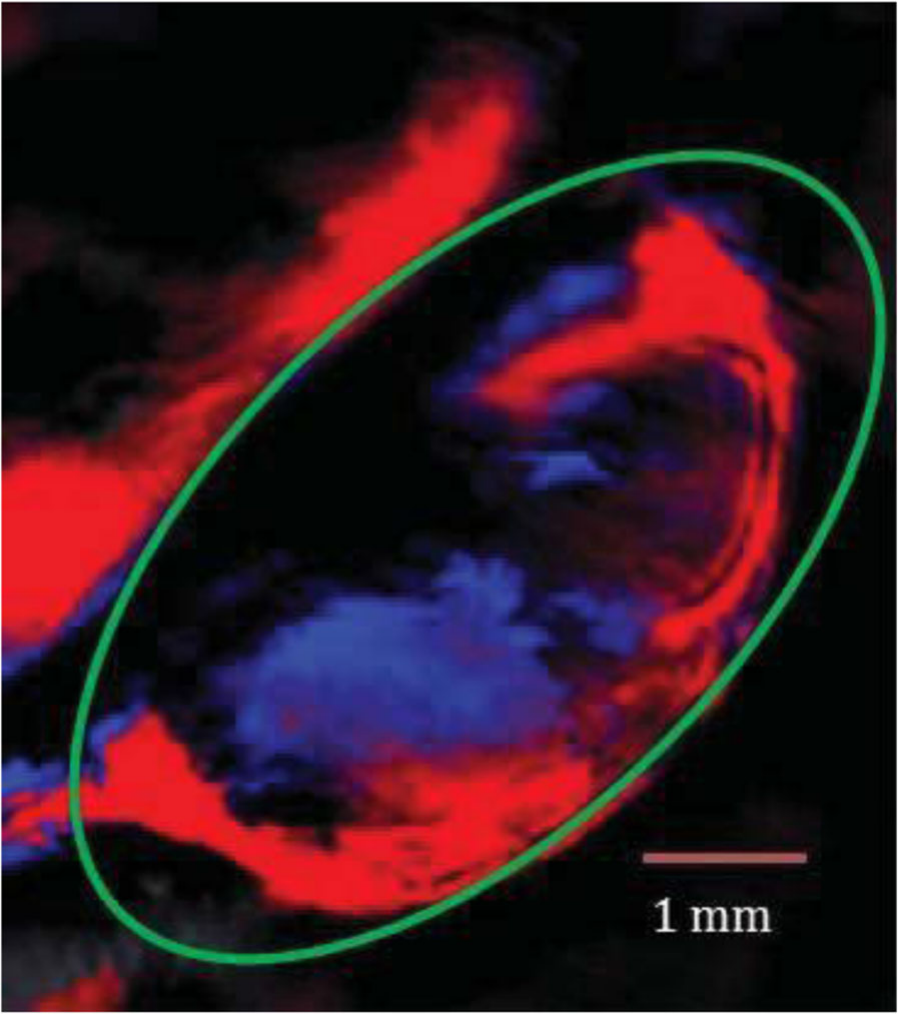
PA image of control tumour (*green ellipse*, 300mm^3^). *Red*: oxy-haemoglobin; *blue*: deoxy-haemoglobin

**Fig. 60 (abstract O48).**
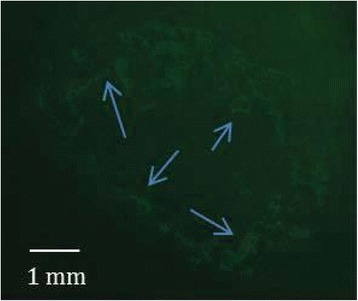
Pimonidazole staining section, corresponding to the tumour in Fig. 59. Hypoxic areas are characterised by *bright green* regions, surrounding a (*darker*) necrotic core, as exemplified by the *blue arrows*

PA imaging suggested that this tumour model develops a necrotic core surrounded by hypoxic areas, despite the well vascularised rim around the tumour, when the volumes reaches ~200 mm^3^, after ~14-26 days, (Fig. 59). This was confirmed by H&E and pimonidazole staining (Fig. 60), although the latter did not show as extensive hypoxic regions as did the PA imaging.

US imaging was able to reliably identify the largest necrotic regions, which we used to compare with PA images to identify target areas for treatment. Of the HIFU treatments performed, the lowest acoustic power was interrupted. The remaining exposures resulted in tissue lesioning, observed after H&E histological analysis, and were identified as further extensive areas of necrosis and haemorrhage. None of the exposures resulted in skin burns. One interesting effect was that treated tumours did not take up the pimonidazole dye, which suggests a significantly reduced tumour perfusion at 24h. Inertial cavitation was detected in both higher power exposures, but not at lower power exposures.

**Conclusions**

The hypoxia distribution observed with photoacoustic imaging correlated well with that expected from the literature on this tumour model (Box et al., 2013). For future studies, we intend to co-register the PA and US images for a more precise treatment plan and use a larger cohort of animals to validate the results obtained with both modalities, using the standard histological techniques - pimonidazole and H&E.

The pimonidazole results, intended to indicate the oxygen distribution, showed lack of penetration of pimonidazole, probably due to vascular occlusion, within 24 hours after HIFU treatment, whereas uptake in control animals processed at the same time was normal. Future studies will investigate this effect at different time points after treatment, with both post-exposure PA imaging and histological analysis, and investigating both hypoxia and perfusion staining. The effects of HIFU on vasculature have been studied before, but it is important to understand the time course of these effects in pre-clinical models as this may have a significant impact on the outcome of combined therapies, such as HIFU-chemotherapy and HIFU-radiotherapy.

### O49 Pulsed focused ultrasound stimulates the molecular responses necessary for stem cell homing through mechanical interactions with stretch-activated calcium channels

#### Scott R. Burks, Matthew Nagle, Ben Nguyen, Michele Bresler, Joseph A. Frank

##### Radiology and Imaging Sciences, NIH Clinical Center, Bethesda, Maryland, USA

**Objectives**

Stem cell therapies are promising regenerative medicine approaches. Pulsed focused ultrasound (pFUS) induces microenvironmental changes in normal and diseased tissues that can enhance local homing and efficacy of intravenously-infused mesenchymal stromal cells (MSC) and further improve disease outcomes. How pFUS interacts with tissues to produce the necessary molecular changes is unclear. Mouse muscle tissue was sonicated at increasing powers while passive cavitation was measured. Sonicated tissue was harvested for cyclooxygenase-2 (COX2) expression to correlate with physical ultrasound effects, as COX2 expression is critical for molecular signalling cascades that induce MSC homing. Lastly, mice were given inhibitors of mechanosensitive channels prior to pFUS to investigate their role in propagating physiological effects of pFUS.

**Methods**

C3H mice were treated with pFUS to the hamstring using a VIFU 2000 system. Under ultrasound imaging guidance, pFUS was delivered at 1 MHz, 5 Hz pulse repetition frequency, 5% duty cycle, and varying transducer output powers (ranging from 10–80 W). Passive cavitation detection was performed with a hydrophone. Mice were euthanized 16 hr post-pFUS and harvested muscle was homogenized and analyzed for COX2 expression by ELISA. For drug studies, mice were given GdCl_3_ (0.04 mmoles/kg) or ruthenium red (0.01 mmoles/kg) by intravenous injection at the beginning of sonications. Statistical comparisons were performed by one-way analysis of variance (ANOVA) using Bonferroni post-hoc tests with p values <0.05 considered significant.

**Results**

Statistically significant increases in COX2 expression were measured at 20, 40, 60, and 80 W compared to untreated muscle. COX2 expression measured after sonications at 60 and 80 W were significantly greater than COX2 expression after sonications at 20 or 40 W. Statistically significant increases in cavitation were not observed at 20 or 40 W, but were observed at 60 and 80 W. Increases in COX2 expression in were blocked at 20 or 40 W when either Gd or ruthenium red were administered during sonication. Forty watts was previously the maximum power that produced the necessary molecular changes without inducing tissue damage and therefore, was the maximum power investigated in this study.

**Conclusions**

Mechanical influences from pFUS drive molecular changes in tissue that are critical to stem cell homing processes. We have previously determined that COX2 expression is an acceptable proxy for molecular outcomes. At lower powers (20 and 40 W), cavitation from the sonications is not detectable, suggesting that cavitation-independent mechanical forces (i.e., acoustic radiation forces) drive COX2 expression. At higher powers (60 and 80 W), cavitation is detectable and COX2 expression is elevated compared to sonications at 20 and 40 W. It is unclear whether the cavitation detected at these powers drives the additional COX2 expression, or if it is the result of increased acoustic radiation forces at those powers. Regardless, 40 W was maximum power we previously determined not to cause detectable tissue damage and from the point of view of regenerative medicine, would be the maximum power used for those applications. At these powers, COX2 increases were blocked by Gd, a non-specific mechanostretch receptor blocker, and ruthenium red, a more specific blocker of transient receptor potential (TRP) channels. At the powers used for regenerative medicine, the acoustic radiation forces from pFUS activate TRP-class calcium channels to initiate the molecular cascade that necessary to induce stem cell homing.

### O50 Pulsed focused ultrasound increases renal expression of interferon-gamma to enhance potency of mesenchymal stem cells and further improve acute kidney injury outcomes

#### Scott R. Burks, Matthew Nagle, Ben Nguyen, Michele Bresler, Saejeong Kim, Blerta Milo, Joseph A. Frank

##### Radiology and Imaging Sciences, NIH Clinical Center, Bethesda, Maryland, USA

**Objectives**

Pulsed focused ultrasound (pFUS) enhances homing of IV-infused mesenchymal stem cells (MSC) to murine kidneys during cisplatin (CIS)-induced acute kidney injury (AKI). pFUS acts as a neo-adjuvant to MSC therapy and the combination leads to better AKI outcomes (renal function and survival) than MSC alone. In wild-type mice, nearly twice as many MSC home to diseased kidneys following pFUS, but >10 times as much interleukin (IL)-10 is produced by MSC that home to pFUS-treated kidneys. This suggests that pFUS sonications modify the renal microenvironment to increase potency of MSC that home to sonicated kidneys. Interferon-g (IFNg) has long been known to increase MSC potency and has been shown to be upregulated in kidneys after pFUS. This study investigates the role of IFNg released by kidneys in response to pFUS improving the therapeutic efficacy of IV-infused MSC.

**Methods**

IFNg knockout (KO) mice received CIS (15 mg/kg ip), kidney pFUS (4 MPa; 5% duty cycle) and/or MSC (10^6^ human MSC). Intravenous MSC injections were performed 3–4 hr post-pFUS. Groups included mice that had AKI only, AKI+pFUS, AKI+MSC, AKI+pFUS+MSC, and normal mice. Mice received CIS on Day (D) 0 and pFUS/MSC on D1. Some mice were euthanized on D2 and kidneys were harvested for molecular analyses. Other mice were euthanized on D4 to measure renal function (blood urea nitrogen [BUN]; serum creatinine [SCr]). Statistical comparisons were performed by one-way analysis of variance (ANOVA) using Bonferroni post-hoc tests with p values <0.05 considered significant.

**Results**

Following pFUS to the kidneys of IFNg KO mice, MSC homing to sonicated kidneys was enhanced ~2 fold compared to untreated contralateral controls. However, increased MSC homing did not lead to improved AKI outcomes compared to mice that received MSC injections alone. Levels of BUN and SCr, as well as expression of kidney injury molecule 1 (KIM1), were all significantly reduced by MSC treatment alone, but not further reduced by combination pFUS/MSC treatment like was previously observed in wild-type mice. Furthermore, significantly greater human IL-10 (IL-10 produced by MSC) was not observed in the pFUS+MSC group compared to mice that received MSCs alone.

**Conclusions**

pFUS creates a molecular zip code in AKI kidneys that enhance homing permeability and retention (EHPR) of infused MSC. While MSC infusions alone improve AKI to some extent in IFNg KO mice, the combination of pFUS+MSCs does not yield further improvements in disease outcomes like it did in wild-type mice. This demonstrates the pFUS-independent mechanism of AKI repair by MSCs does not require renal IFNg, but that the pFUS-dependent mechanism of improved repair/recovery does. It is likely that the IFNg released by pFUS is not solely responsible for potentiation of MSCs, but rather works in concert with a number of other immunological signaling molecules to achieve increased potency. However, IFNg appears to be the critical link for pFUS to function as a neo-adjuvant to MSC therapy in AKI as it is released and/or produced following pFUS. While functional outcomes correlate with lack of IL-10 production by MSCs in the IFNg KO mice, further studies will be necessary to elucidate its role in AKI recovery. These data provide molecular insight to justify using pFUS as a modality to improve MSC therapy during AKI, which often has limited therapeutic options clinically.

### O51 Shear-wave manipulation for tracking high-intensity focused ultrasound (HIFU)

#### Nhan M. Le^1^, Shaozhen Song^3^, Kanheng Zhou^1^, Ghulam Nabi^2^, Zhihong Huang^1^

##### ^1^Mechanical Engineering, University of Dundee, Dundee, Angus, United Kingdom; ^2^University of Dundee, Dundee, United Kingdom; ^3^University of Washington, Seattle, Washington, USA

**Objectives**

Focused ultrasound can induce shear-wave when certain requirements of acoustic power are met, e.g. pulse length, amplitude, frequency. Our group recently found that the characteristics of HIFU-induced shear-wave (HiSW) can be manipulated by changing different acoustic pulse-length and amplitude. By manipulating the HIFU pulse, we could control the wavelength and displacement of the HiSW. Our objective is to demonstrate that HiSW can be manipulated into either i) small and sharp propagating wave, or ii) long wavelength and large displacement propagating wave.

**Methods**

Optical Coherence Tomography with Phase-sensitive technique was utilized for shear-wave imaging. Phase-sensitive Optical Coherence Tomography performs a 256 (time axis) x A-scanlines (depth axis) over the period of 2 milliseconds at the same location for each HIFU pulse. This PhS-OCT-scan repeats in 256 different locations (width axis), forming a complete B-scan dataset over time (3D-dataset). The camera runs at 46200 kHz A-scan rate, exposure 17.4 μs, sensitivity 450 e/count.

Ex-vivo porcine skin is embedded inside 2%-agar phantom to ensure good contact with HIFU transducer. The HIFU transducer (2.09 MHz, 20 mm diameter, 13 mm focal length) is placed at the bottom of the sample. The scanning plane captures the top surface of the ex-vivo sample, defined by the centre point of the HIFU focus, and the axial- and lateral-direction of the HIFU beam.

HIFU-induced shear-wave (HiSW) is captured in reducing number of cycles per pulse, from 100 cycles/pulse to 20 cycles/pulse. The captured image is then processed offline for quantitative analysis of the HiSW, regarding the wavenumber of the HiSW signal.

**Results**

The displacement of HiSW correlates well with the reduction of HIFU cycles per pulse. Lowering HIFU cycles-per-pulse number would reduce HiSW displacement. A reduction of 80% HIFU cycles-per-pulse number (100 vs. 20 cycles/pulse) results in a reduction of less than 50% HiSW displacement (approximately 140 nm vs. 90 nm). Reducing the HIFU cycles-per-pulse parameter also leads to a sharper HiSW, regarding the peak wavenumber of HiSW (refer to Fig. 62, peak wavenumber of 4.2 mm^- 1^ in 100 cycles per pulse, as opposed to 3.7 mm^−1^ in 20 cycles per pulse). However, the sharper HiSW is greatly burdened by the relatively short propagation, as the high-frequency components quickly attenuate over travelling distance.

**Conclusions**

Understanding the response of HIFU-induced shear-wave (HiSW) under different acoustic settings, we can adapt HIFU into both diagnostic and treatment regime. In particular, our experimental setup would benefit the diagnostic and treatment of skin cancer at the same time. PhS-OCT can recover elasticity information from HiSW speed map. The acoustic power output is relatively low, with I_sata_ measuring 23.6 mW.cm^−2^ maximum (100 cycles per pulse, total acoustic power output of 3.72 W), which is suitable for diagnostic purposes. By manipulating the acoustic settings, we could either a) induce a sharp HiSW for tracking purposes, or b) induce a strong HiSW with long propagating distance for diagnostic purposes, i.e. elasticity measurement.

**Fig. 61 (abstract O51).**
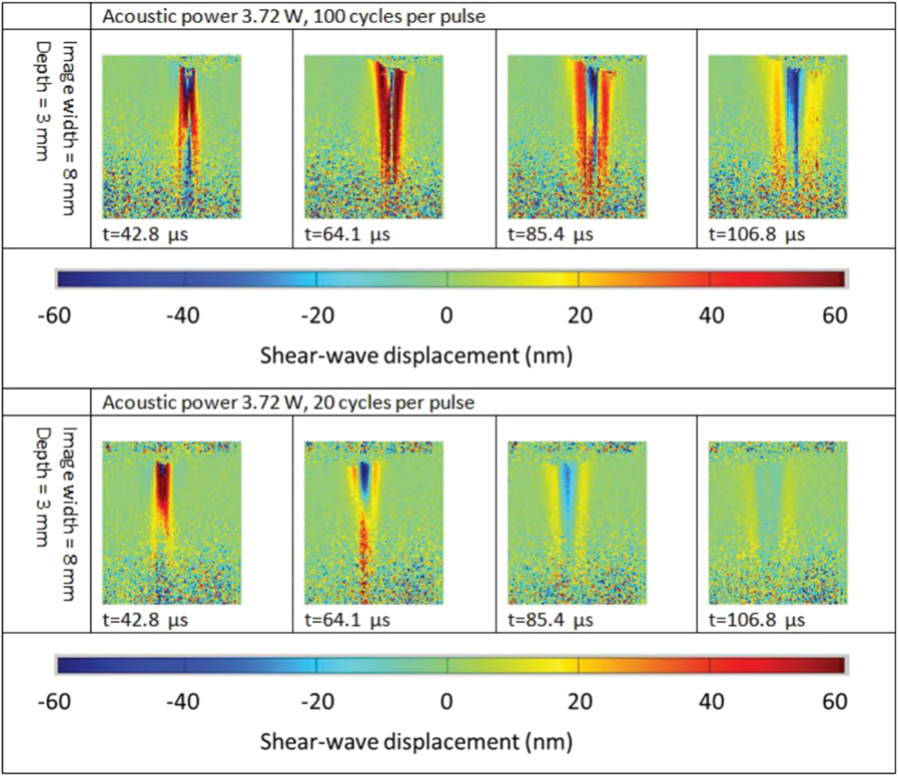
Displacement of HIFU-induced shear-wave at different time frame (after the first acoustic output pulse), with constant acoustic power output of 3.72 W, using a) 100 cycles per pulse, b) 20 cycles per pulse

**Fig. 62 (abstract O51).**
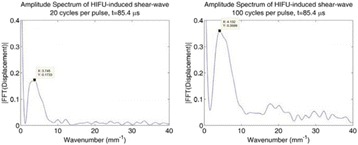
Wavenumber comparison between HIFU-induced shear-wave using 20 cycles per pulse versus 100 cycles per pulse, at constant time frame (85.4 μs after acoustic output pulse) and constant acoustic power of 3.72 W

### O52 The twin piezo motor: low frequency miniature transducer

#### Shmuel Ben-Ezra^1^, Shani Rosen^2^

##### ^1^Action-Physics, Pardes-Hanna, Israel; ^2^CByond ltd., Nesher, Israel

**Objectives**

Traditional transducer designs require a half-wavelength resonator, posing a lower bound for the size of the transducer. The Langevin type transducers are common for frequencies of about 30–60 kHz, comprising a stack of piezoelectric rings pressed to a metal resonator. Their size is about 50–100 mm, depending on frequency and speed of sound in the resonator material. This type of transducers is employed in ultrasonic cleaning baths, ultrasonic dental scalers, ultrasonic scalpel and in many other applications. The low frequency and the relatively large displacement-amplitude provided by the transducer make it ideal to generate cavitation, which may be useful, in turn, for the fragmentation of solid structures, plaque removal, tearing and cutting biological material etc.

In this paper we describe the development of the Twin Piezo Motor: a miniature low frequency, large displacement-amplitude transducer of a new design, in which the resonating element is a metallic, beam shaped tip, vibrating in a transverse (bending) mode. Geometry of the tip may be adjusted for a resonance in the required frequency range, while preserving small footprint.

The Twin Piezo Motor is assumed to be capable of generating cavitation in applications where space is heavily restricted. One possible application is Ultrasonic Lithotripsy (USL) - breaking kidney stones by cavitation. The required solution should pass through the urinary tract over a ureteroscope or a catheter, achieving full contact or getting very close (<2 mm) to the target stone. Upon activation, the transducer tip is assumed to generate a cavitation cloud on the adjacent stone surface, causing its fragmentation into small enough pieces.

**Methods**

We developed a series of transducer models, based on theory and finite element simulations (COMSOL Inc.). Some of the models had actually been built and tested. We used electric impedance analysis (LCR 3532–50, HIOKI Inc.) to locate resonances of the transducer; by comparison with simulation results we could identify the mode of vibration. Fast camera (Phantom V7.3 Turbo, Vision Research Inc.) equipped with a 200 mm lens (AF Micro-Nikkor 200mm f/4D IF-ED, Nikon Inc.) was used to record tip movement and cavitation dynamics in water. The setup enabled recording at frame rate of about 300,000 frame per second. Back illumination was used to enhance contrast. For driving the transducers we used a function generator (AFG 1022, Tektronix Inc.) and power amplifier (2100L, E&I ltd.), with custom transformers for impedance matching.

**Results**

We started by investigating the dental scaler (Selector U2 Plus, Apoza ltd.); we showed that it generates intense cavitation in water. Also, it can fragment a piece of chalk in water. A movie of 300,000 frames per second was produced, showing the dynamics of a cavitation cloud on a water-solid interface at a distance of 1 mm from the vibrating tip. Another interesting movie demonstrated the generation of mist by ultrasonic energy.

The transducer of the dental scaler is of the Langevin type; it is quite large, located inside the hand-piece, and the acoustic vibrations propagate along a shaft from the transducer to the active tip. We looked for a method to generate similar tip vibrations, producing the same results of cavitation, but with much smaller device.

The concept of the Twin Piezo Motor was developed, where the resonance is determined by transverse vibrations of the tip. Two piezoelectric bars of opposite polarity serve as piezoelectric engine such that when the left bar elongates, the right one contracts and vise versa. This combined motion is assumed to excite the transverse mode in the tip. Finite element simulations supported the design of the transducer, and first few samples were built, having overall length of 19 mm and width of 7 mm. The existence of the tip resonance was verified by impedance analysis: a minimum appeared in the anticipated frequency, and it was invariant under structural variations of the transducer.

The large amplitude vibration of the Twin Piezo Motor occurs at selective frequency and was demonstrated visually by the generation of mist. A nice movie at frame rate of 6688 frames per second was recorded.

**Conclusions**

The design of the Twin Piezo Motor is based on the resonance of the tip in transverse (bending) mode, with 2 piezoelectric bars at opposite polarities serving to generate the vibration. We demonstrated the resonative vibration of the tip and the generation of mist by tip vibrations. When the vibratory tip is in full contact with a wet stone, the device exhibits some grinding capabilities, reducing the size of the stone. Direct evidence for cavitation in water is still missing. The simple assembly of the transducer is done by one central bolt holding the components together. The first samples that we have built are too big; they have to be further diminished in a factor of 2 or 3 in order to pass through urinary tract.

**Fig. 63 (abstract O52).**
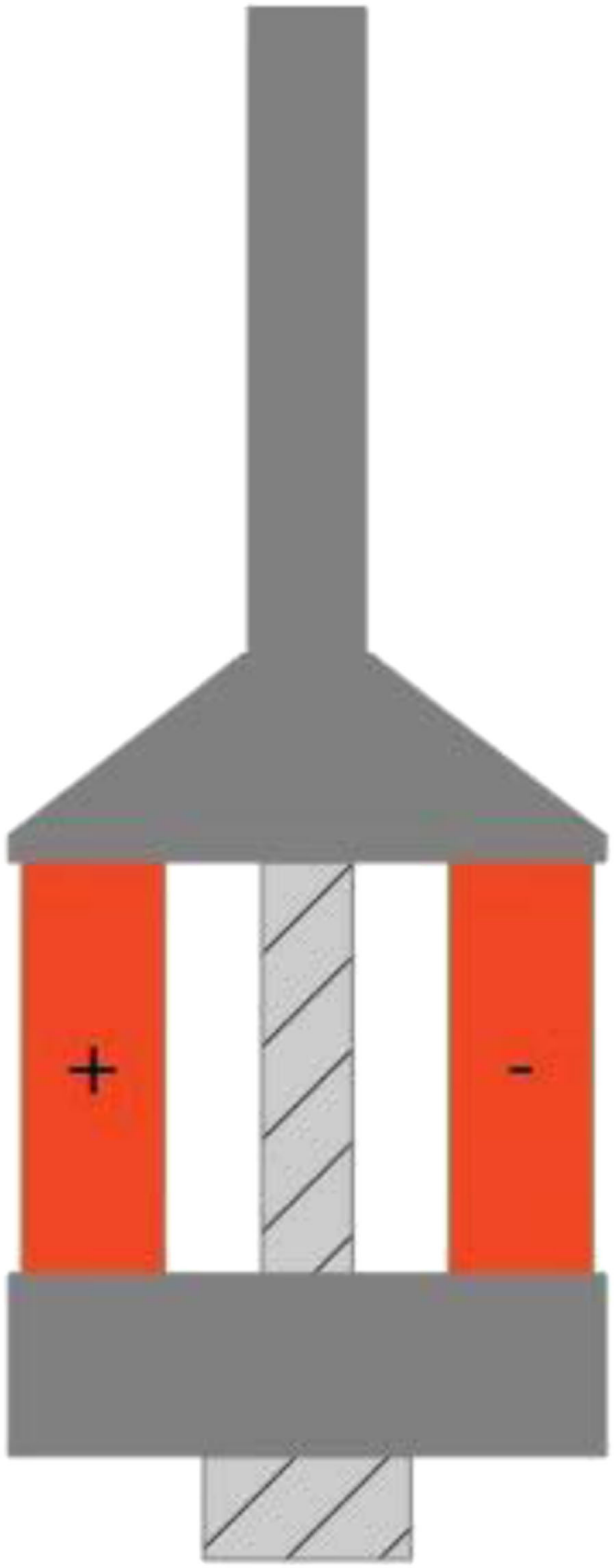
Schematics of the Twin Piezo Motor: 2 piezoelectric bars at opposite polarity are pressed by a central bolt between the back mass and the tip. The tip is designed to be in resonance in bending mode at the required frequency

**Fig. 64 (abstract O52).**
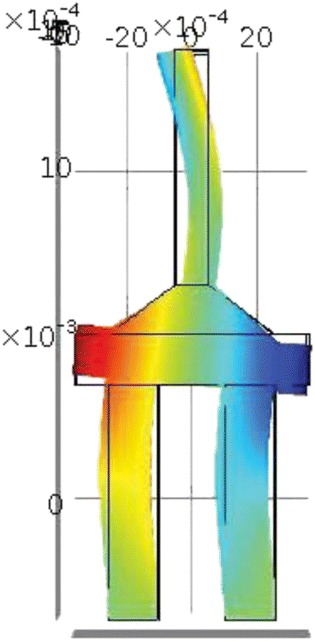
COMSOL simulation of the Twin Piezo Motor: The resonance frequency is determined by tip transversal vibrations

**Fig. 65 (abstract O52).**
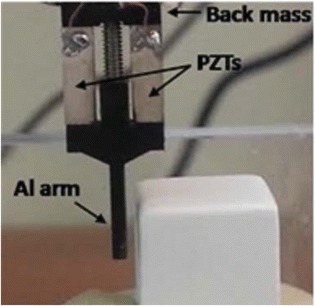
First prototype of the Twin Piezo Motor. The vibratory tip is located close to the surface of an artificial stone

### O53 Measurement of sonication duration for ablation of tumour in liver using trans-fusimo treatment system by using fiber-optic hydrophone

#### Senay Mihcin^1^, Jan Strehlow^2^, Ioannis Karakitsios^1^, Nhan Le^1^, Michael Schwenke^2^, Daniel Demedts^2^, Paul Prentice^1^, Sabrina Haase^2^, Tobias Preusser^2^, Andreas Melzer^1^

##### ^1^School of Medicine, IMSaT, Dundee, United Kingdom; ^2^Fraunhofer, Mevis, Bremen, Germany

**Objectives**

The application of Focussed Ultrasound (FUS) in upper abdominal organs is particularly challenging due to complexity of breathing motion, a multitude of risk structures and possible occlusions through the rib cage. TRANS-FUSIMO Treatment System (TTS) is a newly developed software (MEVIS, Fraunhofer, Bremen) enabling Magnetic Resonance (MR) guided FUS (MRgFUS) in upper abdominal organs. MR organ motion tracking data is used for a model-based motion compensation while monitoring the temperature [1]. Due to the complexity of the system, TTS demands thorough validation before its use in animal trials. One important system parameter in this evaluation of FUS is the duration of sonication. The delay when starting a sonication, the deviation from the planned sonication time, and the delay in the system after a sonication is stopped, needs to be measured accurately to quantify system performance.

**Methods**

**Fig. 66 (abstract O53).**
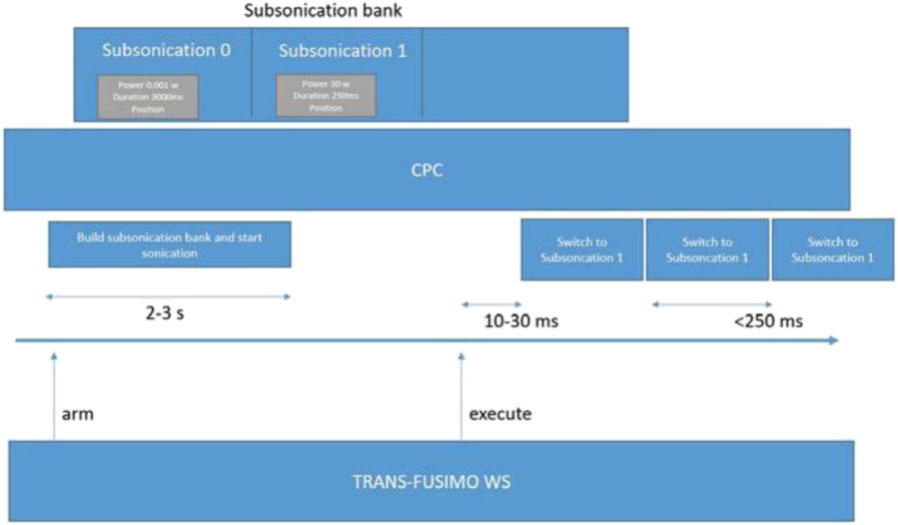
Sonication execution with subsonication bank algorithm using Trans-fusimo Treatment Software

The evaluated version of the TTS uses the software interface of Signa 1.5 T MR Scanner (GE Healthcare, UK ) and the transducer of Conformal Bone System 2100 (CBS) (INSIGHTEC, Israel) . On the CBS transducer system, steerable sonications are realized by so called *subsonications* that have an individual focus position, and duration. Subsonications are organized in *sonication banks* and the active subsonication can be switched during sonication rapidly (2ms). Starting a sonication on the CBS, however has a considerable delay of approximately 2–3 seconds. To enable precise control of the sonication time, TTS employs a sonication strategy that exploits the short switching times between subsonications. For example, to start a static sonication, it builds a sonication bank with two subsonications. The first is a subsonication with very low power (0.001 W) and a long duration. It is the default subsonication and used to start the sonication. In the TTS, this step is called *arming* the transducer. The second subsonication has the actually prescribed sonication focus position and power information. It is activated immediately after the user choses to start the sonication via the TTS *execute* command. For safety reasons, the second sonication’s duration is limited to 250 ms and it is actively looped by the software until the prescribed duration of the sonication has passed, or until the user stops the sonication via a *stop* command (Fig. 66).

The sonication is monitored via single shot EPI MR Sequence of 512 phases, with TE: 26.4, TR: 100, flip angle: 40, freq phase: 128 x 96 parameters on the MR machine. To synchronise the MR to the TTS, the MR is configured to start monitoring after a TTL-Trigger pulse sent from the TTS.

Testing To collect data during sonication, fiber optic hydrophone (Pa Ltd, UK) was used. The fiber optic hydrophone works on the principle of interferometric detection of changes in the optical thickness of a thin polymer film at the tip of the optical fiber sensor downlead. Changes in the thickness may be induced acoustically (through the acoustic pressure) or thermally.

The system is capable of differentiating between the two and making simultaneous measurements of both (Morris et al. 2009). However, in this study, the main purpose was to record the signal during sonication.

**Fig. 67 (abstract O53).**
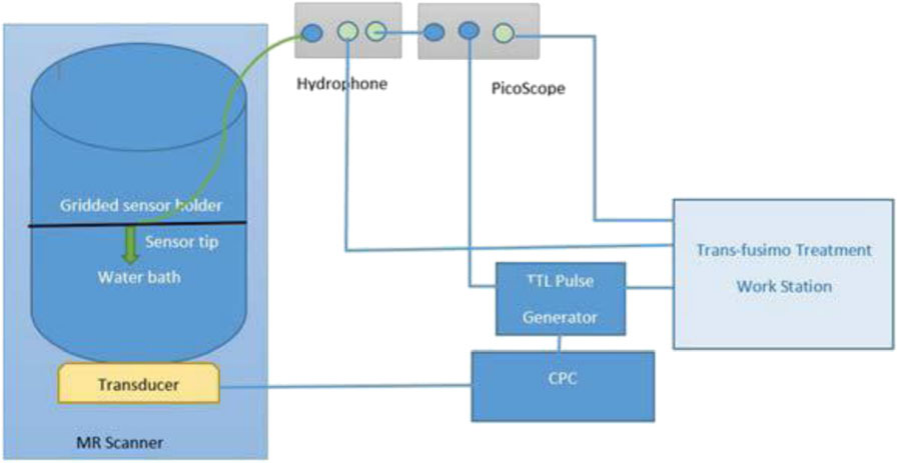
Schematic view of experimental set-up

**Fig. 68 (abstract O53).**
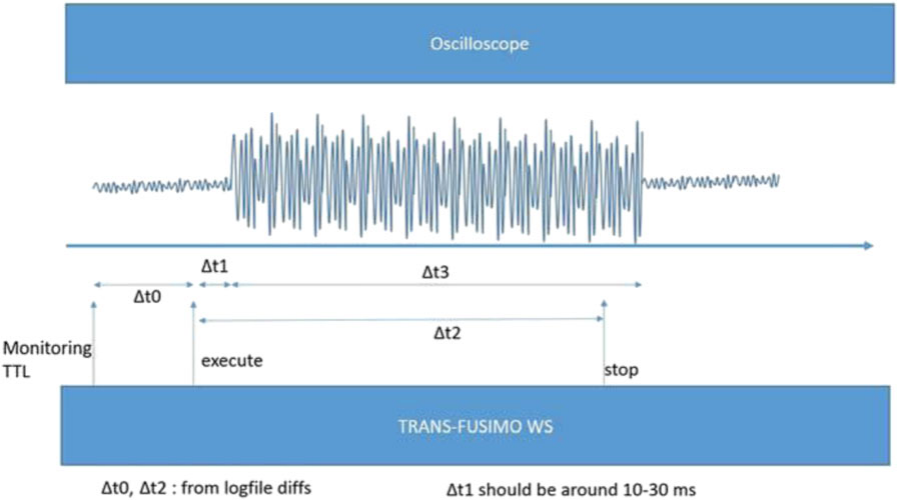
Time diagram for the execution of the sonication with Trans-fusimo Treatment System

Experiment set-up consisted of a water tank filled with degassed water. Gridded surface sensor holder was placed on the top of the water tank (Fig. 67). Fibre-optic hydrophone sensor was mounted on the grid surface. MR Scan was used to find the exact location of the tip of the sensor. This data was used to sonicate to the tip of the fibre-optic sensor by using TTS. Fibre-optic sensor was hard wired to Fibre-optic Hydrophone System control unit. Hydrophone system has its own software to control its hardware. To obtain reading from hydrophone, a computerised scope such as PicoScope (Picotech, UK) was connected via the “AC out” connector on the front panel. The system was designed to have an output impedance of 50 Ohm. PicoScope has two inputs, first is the output of the hydrophone, and second is the monitoring trigger pulse coming from TTS. In order to initiate recording of data, detected by hydrophone, a TTL pulse generated by TTS, was utilised. The time information for the TTL pulse, the ‘execute’ command and ‘stop’ commands were recorded as a tag line in the software (Fig. 68). Deviation during sonication was calculated as t2- t1 (1). Delay after sonication stop button pressed was calculated as shown below (2) length of the signal on PicoScope (t3) minus delay in the start time ( t1) during sonication duration.

1 $$ \mathrm{Deviation}=\mathrm{t}2-\mathrm{t}1 $$

2 $$ \mathrm{Delay}=\mathrm{t}3-\left(-\mathrm{t}1+\mathrm{t}2\right) $$

Picoscope was programmed using LabView (National Instruments, UK) with 100 ms of time resolution to record the signal simultaneously with the TTL pulse.

**Results**

With sonication power of 30W for 30 seconds, the deviation of the actual sonication duration from the planned duration was found smaller than 1 second. The time until the transducer stops sonicating, after the stop button was pressed, was calculated as less than 200 ms.

**Conclusions**

In this study, feasibility of measuring the deviation of the actual sonication and delay after stop button release was tested. The methodology described in this study proves that it is possible to quantify these parameters. With the established methodology, the next step is to quantify the repeatability and reliability of the system with different sonication timings and power values using the Transfusimo Treatment Software (TTS) and the conformal bone system transducer. Transfusimo Treatment System (TTS) is planned to be tested on animals based on these results.

**References**

[1] Schwenke et al. An integrated model-based software for FUS in moving abdominal organs, Int J Hyperthermia. Vol 31(3): 240–250, 2015.

[2] Morris et aI. A Fabry-Perot fibre-optic ultrasonic hydrophone for the simultaneous measurement of temperature and acoustic pressure”, J. Acous. Soc. Am. Vol 125(6) pp. 3611–3622, June 2009.

### O54 Cell transfection, yeast and bacteria transformation with a confocal ultrasound device

#### Jean-Louis Mestas^1^, Kamel Chettab^4^, Gustavo Stadthagen Gomez^2^, Charles Dumontet^3^, Bettina Werle^2^, Cyril Lafon^1^

##### ^1^U1032, INSERM, Lyon, Rhone-Alpes, France; ^2^Bioaster, Lyon, Rhone-Alpes, France; ^3^U1052, INSERM, Lyon, Rhone-Alpes, France; ^4^Université de lyon, Lyon, Rhone-Alpes, France

**Objectives**

Acoustic cavitation can be used for *in vitro* and *in vivo* gene delivery as an alternative to viral-based transfection methods.

Gene delivery into expression hosts is one of the first critical steps in recombinant DNA applications. Commonly used methods for DNA delivery into useful biotechnology organisms include, chemically mediated transformation, electroporation and viral transduction. These methods are often cumbersome, long and limited to a single or few specific hosts. This work aims at presenting our development on a confocal ultrasound device for transfecting eukaryote cells with increased efficiency and acceptable viability in a reproducible manner or for delivering functional DNA into *Kluyveromyces lactis* and *Escherichia coli*, respectively a yeast and a bacterium.

**Methods**

This device is based on two piezo ceramic spherical shells placed in a confocal manner (1.1 MHz; Diameter and curvature radius 50 mm, angular gap 90°). This particular configuration is favorable for the initiation and control of cavitation activity. The crossing of the two beams forms an interference patterns that traps the bubbles in the focal zone. The device also integrates a regulation process to control the cavitation activity by adjusting in real time the amplitude of the ultrasound signal as a function of the recorded acoustic response of the cavitation bubbles. With this control loop, the measured activity is within 5% of the desired value. The sonicated volume is placed in 2 ml Eppendorf tubes for cells and yeast (650μl) or in 0.2ml tubes for *E-Coli* (200μl)*.*

**Results**

For transfection, the device was evaluated on 11 adherent cell lines and 10 non-adherent cell lines. The presented results are limited on Jurkat and K562 cell lines considered difficult to transfect. The peGFP-C1 transfection efficiency and cell viability were evaluated 24h post sonication. Results show a proportional relation between transfection efficiency and cavitation activities for both cell lines. Optimal transfection rates were 77% and 49% for Jurkat and K562 respectively. The corresponding viabilities were 42% and 84%. These results are comparable to nucleofection method. On a third adherent cell line, A549, this exposure condition gave 80% transfection efficiency for 75% of cell viability.

For transformation, the efficiency was evaluated versus the cavitation index characterizing the cavitation activity level.

**Conclusions**

A user-friendly and cost-effective ultrasound device was developed. It is well adapted for routine in vitro high-yield transfection and transformation experiments as it does not require the use of any transfection reagent or gas micro-bubbles. It provides a well-adapted method for low cost routine pDNA *in vitro* delivery for both adherent and non-adherent cell lines yeast and bacteria. This method allows reducing cost for transformation by sonicating bacteria straight in their culture medium. Our results confirm ultrasound as an alternative of non-viral technology for the efficient transient transfection of a wide range of different cells including non-adherent cells or fresh human cells, and the preparation of stably transfected cells.

**Fig. 69 (abstract O54).**
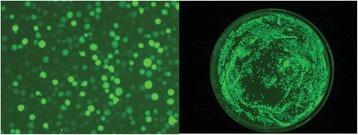
Stable GFP expression for K562 Cells and *e-coli* bacteria colonies upon sonication

**Fig. 70 (abstract O54).**
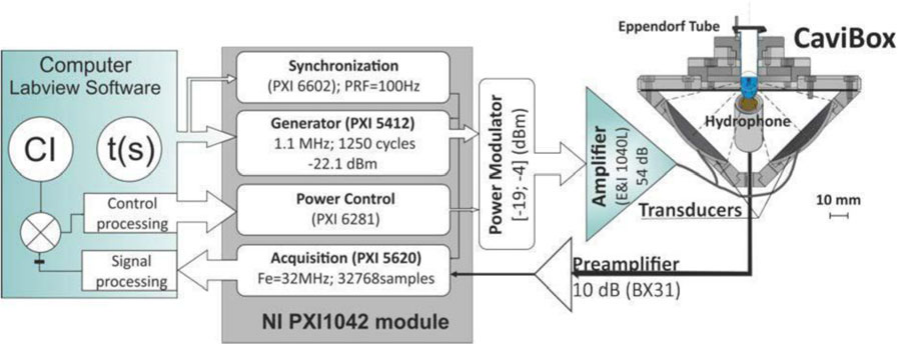
Experimental set-up

**Fig. 71 (abstract O54).**
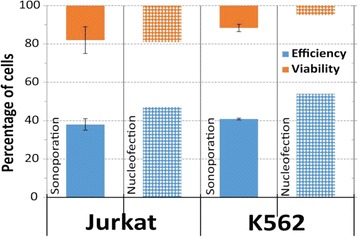
Comparison of sonoporation and electroporation transfection on 2 cell Lines

**Fig. 72 (abstract O54).**
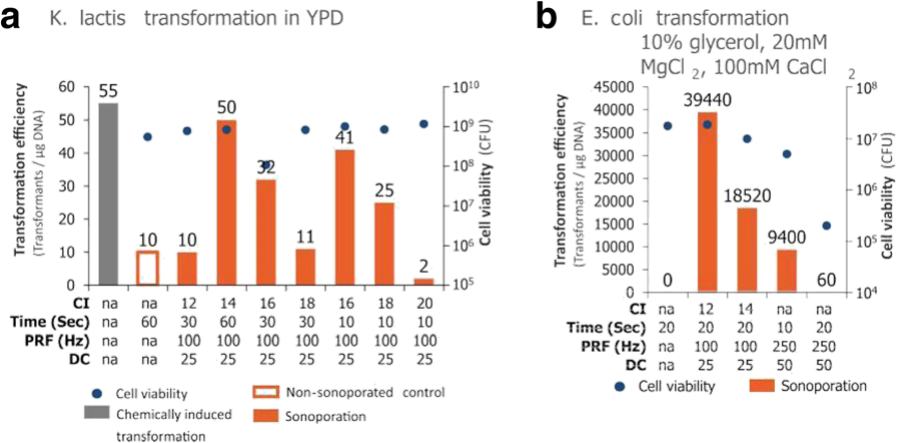
Yeast and E. coli transformation efficiency evaluation versus sonication parameters

### O55 Non-invasive cardiac pacing using image-guided focused ultrasound *ex vivo* and *in*-*vivo* in pigs

#### Fabrice Marquet^1,2^, Pierre Bour^1,2^, Fanny Vaillant^1,2^, Sana Amraoui^1,3^, Rémi Dubois^1,2^, Philippe Ritter^1,3^, Michel Haïssaguerre^1,3^, Mélèze Hocini^1,3^, Olivier Bernus^1,2^, Bruno Quesson^1,2^

##### ^1^IHU Institut de Rythmologie et de Modélisation Cardiaque, Bordeaux, France; ^2^INSERM U1045 CRCTB, Université de Bordeaux, Bordeaux, France; ^3^CHU de Bordeaux, Bordeaux, France

**Objectives**

Currently, no non-invasive cardiac pacing device acceptable for prolonged use in conscious patients exists. The main approach is invasive, employing intravascular catheters, which has associated risks. HIFU can be used to perform remote pacing using reversibility of electromechanical coupling of cardiomyocytes. This technique might be useful in the short term in the clinical settings in various conditions: temporary pacing for bradycardia or any clinical condition with risks of asystole; terminating or examining the inducibility of tachyarrhythmia; screening and optimization of cardiac resynchronization therapy. Here we described an extracorporeal cardiac stimulation device and study its efficiency and safety. We conducted experiments ex vivo and in vivo in a large animal model (pig) to evaluate clinical potential of such a technique.

**Methods**

Experiments were performed with an MR-guided HIFU platform combining a 1.5T MRI (Siemens Avanto, Germany) and a focused ultrasound device (Image Guided Therapy, France, 256 elements, 13/13 cm aperture/focal, operating at 1 MHz). MR images were recorded using a balanced steady-state free precession sequence (TE/TR/FA/BW = 1.36ms/493ms/80°/1149 Hz.pixel^−1^, spatial resolution 1x1 mm^2^, slice thickness 3 mm, 256x256, 40 slices, 3 stacks acquired in transverse, sagittal and coronal orientations) to select the location of the stimulation site and to adjust beam focusing characteristics (mechanical positioning and electronic beam steering). *Ex vivo* acoustic stimulation threshold was determined performing 756 sonications in the right atrium (83 sonications), the left (431 sonications) and the right ventricles (242 sonications) in 10 ex vivo beating hearts from pigs. *In vivo* non-invasive stimulation proof of concept was shown performing 314 sonications in 4 anesthetized pigs including 42 sonications without ultrasound contrast agent in the first two animals. The last two animals were injected with ultrasound contrast agents using SonoVue (Bracco, Italy, mean terminal half-life: 12 min, range from 2 min to 33 min). Two consecutive 0.1 mL.kg^−1^ boli intravenous injection were performed in each animal. Local cardiac electrograms (bipolar measurements) were continuously recorded by three MR-compatible pacemaker leads (CapSureFix MRI Model 5086, Medtronic, MN, USA) inserted into the right ventricle, the left ventricle and the right atrium and connected to a clinical electrophysiology recording system (Bard Inc., NJ, USA). At the end of each in vivo experiment, a navigated delayed inversion-recovery 3D Flash sequence was performed (TE/TR/TI/FA/BW = 3.93ms/714ms/320ms/13°/130Hz.pixel^−1^, spatial resolution 0.5x0.5 mm^2^, slice thickness 2.5 mm, 576x576, 52 slices). The animals were injected with 0.2 mmol.kg-1 gadoterate meglumine (Gd-DOTA, Dotarem®, Guerbet, Roissy, France) and scanned 15 minutes post injection. Gross examination of each heart was performed after the heart excision. Histological analysis was performed to assess acute damages screening from acoustic stimulation. Tissue samples of stimulated heart (N=40) as well as control regions (N=24) were collected in 4 *ex vivo* and 4 *in vivo* hearts.

**Results**

Using HIFU it was possible to perform ventricular continuous pacing (A) or to induce ventricular tachycardia (B). Consecutive stimulations of different heart chambers with a single ultrasonic probe was shown, allowing to modify the resulting atrio-ventricular delay (C-D). The results of the 756 stimulation sites performed in the right atrium (RA, 83 sonications), and the left and right ventricles (431 and 242 sonications respectively) in 10 *ex vivo* beating hearts from pigs were processed to determine stimulation threshold. For each HIFU pulse duration tested ranging from 30 μs to 10 ms, the success of stimulation increases with the acoustic pressure at focus. Two different pressure thresholds were highlighted: one around 4MPa peak negative for HIFU pulse durations above 1 ms and one around 6 MPa peak negative for HIFU pulses ranging from 50 μs to 1 ms (E). The same setup was used *in vivo* in 4 pigs to show clinical potential (F). Electrophysiological changes were confirmed by arterial pressure modifications (G). The minimal stimulation threshold of 4 MPa negative pressure at the focus (as determined from ex vivo experiments) could not be reached with our current in vivo setup. The maximal peak negative pressure was estimated to be around 2 MPa in situ, due to the limited acoustic window. At this pressure level, stimulation of the LV was observed but with an insufficient success rate. To overcome this limitation and demonstrate in vivo feasibility, ultrasound contrast agents were injected intravenously to enhance HIFU mechanical effects on tissue, hence decreasing the stimulation threshold. Using this protocol, consistent cardiac stimulation was achievable for up to 1 hour sessions in 4 different animals. No damage was observed in inversion-recovery MR sequences performed in vivo in the 4 animals. No signal increase could be seen in the myocardium in the delayed-enhancement MR images that would indicate irreversible injury. Gross pathology and Masson’s staining revealed no differences between stimulated and control regions, for all the *ex vivo* and *in vivo* cases.

**Conclusions**

To the best of our knowledge, this study is the first *ex vivo* and *in vivo* proof of feasibility of controlled noninvasive ultrasound-based cardiac stimulation in large animals. The *ex vivo* characterization demonstrated the potential of this technique in an environment where acoustic parameters were well-controlled and quantitatively determined the stimulation threshold as a function of ultrasound pulse duration and amplitude. The *in vivo* proof of feasibility performed in large animals showed that this novel technology offers good prospects for clinical developments. Encouraging safety results show that acute stimulation during hour-long sessions did not cause any detectable thermal and mechanical damage under the experimental parameters used.

**Fig. 73 (abstract O55).**
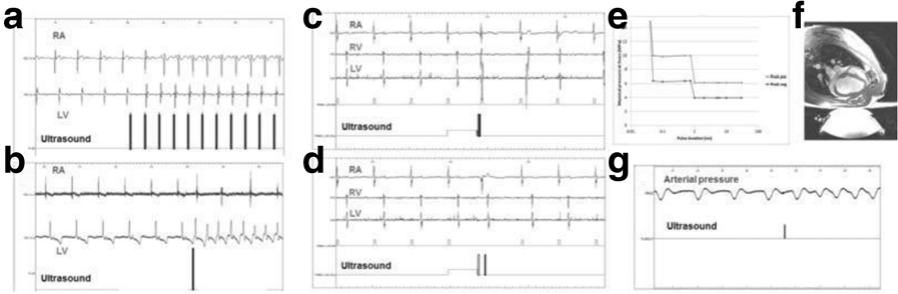
**a** Electrophysiological readings of continuous ultrasonic pacing of the heart at 120 min-1 (sinus rhythm: 100 min-1). **b** Electrophysiological readings of ultrasound-induced non-sustained ventricular tachycardia (165 min-1, sinus rhythm 85 min-1) performed by synchronizing the acoustic emission with the relative refractory period. **c**-**d** Example of atrioventricular stimulation with a single ultrasonic probe. Phased array transducer enables consecutive stimulations of the RA (yellow pulse) and the RV (red pulse) with a chosen delay. **e** HIFU pressure thresholds at the target (peak positive - blue curve- and peak negative -red curve)) vs ultrasound pulse duration to induce ventricle stimulation. **f** Transverse MR images of the anesthetized pig used during the in vivo proof of concept. **g** Example of basic electrophysiological and arterial pressure readings. Arterial pressure is reported to prove induction of premature ventricular contraction and non-sustained ventricular tachycardia

### O56 A study of the dominant mechanisms of extracorporeal acute cardiac pacing by high intensity focused ultrasound

#### Amit Livneh, Eitan Kimmel, Dan Adam

##### Department of Biomedical Engineering, Technion-Israel Institute of Technology, Haifa, Israel

**Objectives**

Extracorporeal acute cardiac pacing by high intensity focused ultrasound (HIFU) could be a disruptive technology in the field of cardiology. Two clinical applications in which acute cardiac pacing by HIFU may be valuable are: (1) preoperative patient screening in cardiac resynchronization therapy surgery where currently 20-40% of operations fail; (2) Emergency life support, which may prevent an event of cardiac arrest from causing sudden death. Both applications may better morbidity and mortality rates in heart failure patients. While ultrasonic cardiac stimulation was first applied 87 years ago, the mechanisms of ultrasonic cardiac pacing are yet unknown. Our work aims to unveil the dominant mechanisms of HIFU cardiac pacing, using a combined experimental and modeling approach.

Recently, we published results demonstrating HIFU extra systole induction in whole anesthetized rats. Sequences of multi harmony HIFU paced extrasystoles were obtained owing to adequate spatiotemporal control, which employed online ultrasound guidance and real-time vital signs signal processing. An illustration of a sequence of HIFU paced premature ventricular contractions (PVCs) is presented in panel A of the figure below. Visual inspection post pacing showed no indication of gross damage or petechia, histological evaluation didn’t show staining or signs of inflammation 24 hours post pacing. Panel B of the figure below shows a heart post HIFU pacing and histological staining results 24 hours post pacing. Extrasystole induction was demonstrated temporally throughout the entire cardiac cycle beyond the absolute refectory period and spatially across the entire left ventricle. Passive Cavitation Detection (PCD) was applied in conjunction with US imaging on a gel phantom, and on rats. The gel phantom was sonicated with a HIFU pacing sequence, PCD positive cavitation indication was correlated with observed hyperechoic imaging. Similar PCD indications were recorded during in-vivo HIFU pacing, while hyperechoic imaging was not observed. Analysis of these experimental results suggests membrane currents as the dominant cellular level mechanism and cavitation as the dominant ultrasound tissue interaction mechanism. The hypothesis we test here through modeling and simulation is that HIFU induced intramembrane cavitation could induce Premature Action Potentials (PAPs) in a model of a cardiomyocyte by altering the membrane capacitance.

**Methods**

The Livshitz & Rudy guinea pig LV cardiomyocyte model and O’Hara et al. human LV cardiomyocyte model were adapted to include variable capacitance induced ionic currents and membrane voltage alterations. Numerical simulation in Matlab was applied to calculate the temporal membrane capacitance changes due to simulated HIFU insonation, and the resulting ion and membrane voltage dynamics. The simulated HIFU insonation reconstructed the minimal peak negative pressure that was observed to be required for HIFU pacing in rats.

**Results**

Numerical simulation results demonstrated HIFU PAP induction throughout the entire diastole (evaluated by the temporal offset from the preceding AP peak of the membrane voltage trace). An illustration is shown in panels C-E of the figure below. The membrane potential is shown in blue. A baseline sinus rhythm was produced by electrical stimulation at 2Hz, the electrical stimulation is noted by the down facing black bars at 0 and 500ms. Ultrasonic pacing was applied at different times during the diastole, the ultrasonic pacing is noted by the upward facing red bars.

Membrane depolarization was gradual, and the ion dynamics composition was similar to that of normal sinus rhythm. The temporal offset between insonation onset and the resulting PAP replicated the in-vivo observations. Moreover, PAP induction was demonstrated to occur also during insonation.

**Conclusions**

The simulation results of a small animal model reproduced our in-vivo observations. This supports our hypothesis of the suggested dominant mechanisms. The simulation results of a human cardiomyocyte model share similar characteristics and attributes to those of the small animal model, offering the prediction that HIFU pacing could be performed in humans with the same pacing patterns that were applied on whole anesthetized rats.

The presented results offer new insights to the study of HIFU pacing and predict that HIFU pacing may be performed in human subjects without membrane disruption.

**Fig. 74 (abstract O56).**
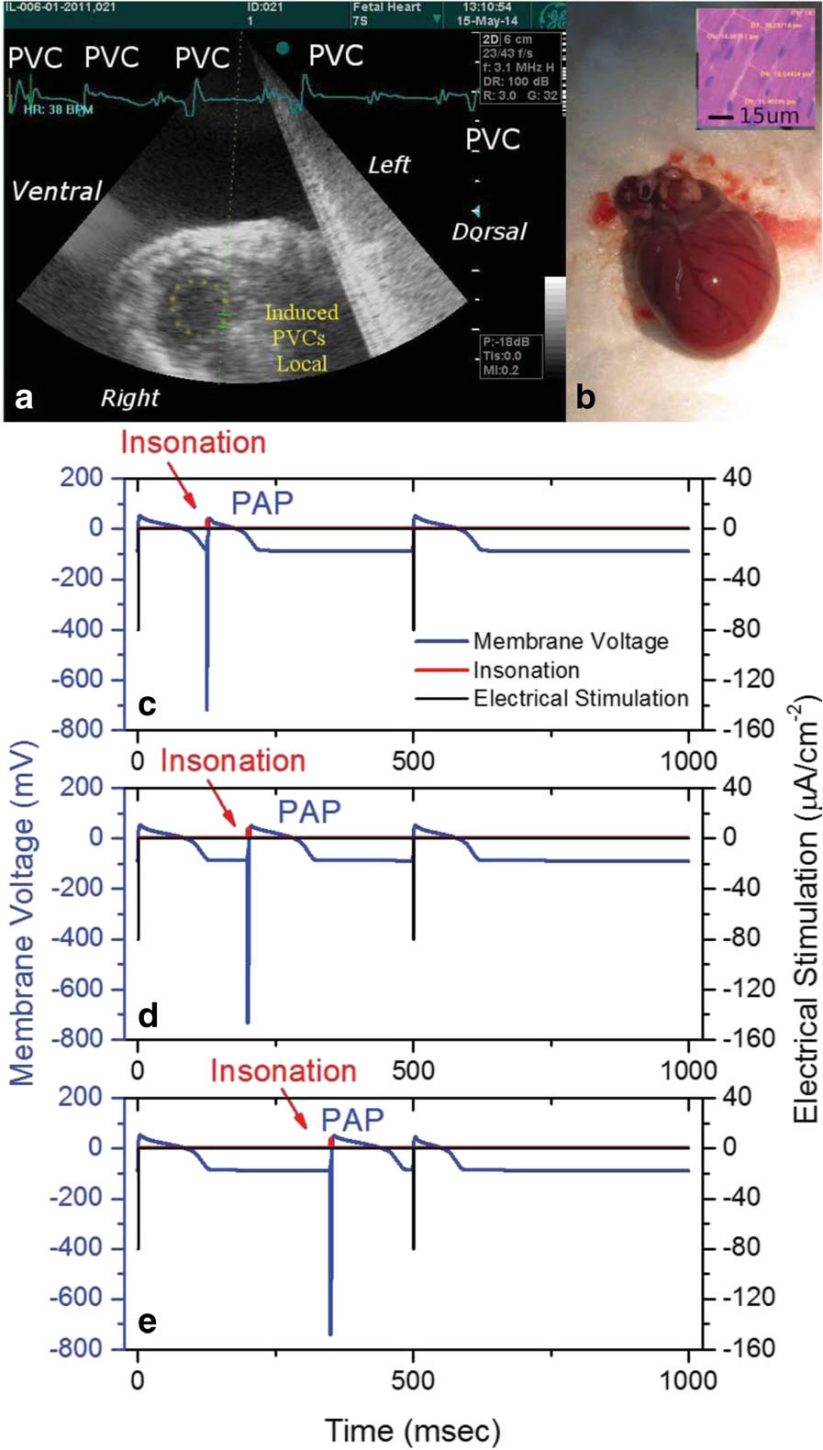
Results Highlights. **a** a Short axis ultrasound (US) imaging of noninvasive HIFU pacing in a rat. The HIFU focus is marked by the curser which is placed on the most dorsal part of the rat’s left ventricle. A sequence of premature ventricular contractions (PVCs), were successively paced by US guided multi harmony HIFU insonation. The PVC sequence is marked on the ECG trace. **b** A rat heart following HIFU pacing showing no sign of gross damage and an example of Eosin (H&E) staining showing no sign of inflammation 24 hours post pacing. **c**-**e** Numerical simulation results of multi harmony HIFU pacing in a small animal model cardiomyocyte demonstrating Premature Action Potentials (PAPs) induction throughout the entire diastole and showing PAP peak voltage was assumed within the same temporal delay from insonation onset as was demonstrated by extracorporeal HIFU pacing in rats. Contractions (PVCs), were successively paced by US guided multi harmony HIFU insonation. The PVC sequence is marked on the ECG trace. **b** A rat heart following HIFU pacing showing no sign of gross damage and an example of Eosin (H&E) staining showing no sign of inflammation 24 hours post pacing. **c**-**e** Numerical simulation results of multi harmony HIFU pacing in a small animal model cardiomyocyte demonstrating Premature Action Potentials (PAPs) induction throughout the entire diastole and showing PAP peak voltage was assumed within the same temporal delay from insonation onset as was demonstrated by extracorporeal HIFU pacing in rats

### O57 3D time reversal cavity for histotripsy over a large volume

#### Justine Robin^1^, Bastien Arnal^2^, Mathias Fink^1^, Mickael Tanter^1^, Mathieu Pernot^1^

##### ^1^Institut Langevin, ESPCI ParisTech, CNRS UMR 7587, INSERM U979, Université Paris Diderot, Paris, France; ^2^Institut Langevin, ESPCI ParisTech, CNRS UMR 7587, INSERM U979, Université Paris Diderot, Paris, France

**Objectives**

Ultrasound pulse therapy such as histotripsy or lithotripsy requires focusing very high pressures to mechanically fragment and liquefy tissues. Large spherical transducers are commonly used to achieve these pressures at the focal spot and mechanical steering is then required to treat large regions. Using 2 dimensional arrays of high power transducers is another possibility, but electronic steering is still highly limited by the number of elements that cannot exceed several hundreds for reasons of cost and complexity. In this study, using both numerical simulations and experiments, we have developed a 3-dimentional time reversal cavity (3D-TRC) to focus high intensity pulses over a large volume only using electronic steering, and keeping the number of elements to a minimum.

**Methods**

We designed a 3D-TRC by enclosing a 3D-multiple scattering medium (MSM) in a reverberating cavity. We used simulations with the k-wave software (pseudo-spectral calculation method, B. E. Treeby and B. T. Cox) and an experimental realisation of our device to optimise its focussing and steering capacity. In both simulations and reality, the cavity was 15x13x20 cm, with steel walls, and filled with water. MSM was either made of steel rods (diameter 0.8mm), or successive metal grids (wire diameter 0.8mm, size of cell 5 mm). Transducers were placed in the back of the cavity, opposite the aperture.

In the simulations, we defined a 119x204x506 matrix, with 0.5 mm grid steps, representing a water volume, in which we placed the steel cavity. A source was placed in front of the cavity in the centre or on the side of the aperture, and emitted a 2-cycle pulse at frequency 1 MHz. Signal was picked up by transducers in the back of the cavity, and stored. Time reversal focusing (TRF) then allowed us to refocus these signals on the initial source point. We explored different kinds of MSM and different sizes and shapes of transducer elements in the cavity. Particularly, we compared the performances of our cavity with either a 128-element linear transducer of high elevational width or an array of 128 square elements of different sizes.

For cavitation experiments, we chose to use 2 high power linear transducers (128-elements, 1 MHz, Imasonics, Besançon, France), placed orthogonally at the back of the cavity, sonicating the MSM with an angle of 60 degrees. The probes were driven by custom multi-channel electronics (Correlec, France)*.* 40 μs US pulses emitted through the cavity were temporally spread to up to 1 ms, picked up by a HGL 200 hydrophone (Onda, Sunnyvale, CA) and stored. Time reversal focusing (TRF) by compressing these signals in space and time then allowed us to reach the needed high negative pressures. Steering the focal spot over a large volume was achieved by moving the hydrophone. We reemitted the reversed signal at a pulse repetition frequency (PRF) of 260 Hz to form a bubble cloud, which was observed using an ultrasound scanner (Supersonic Imagine, Aix-en-Provence, France).

**Results**

**Fig. 75 (abstract O57).**
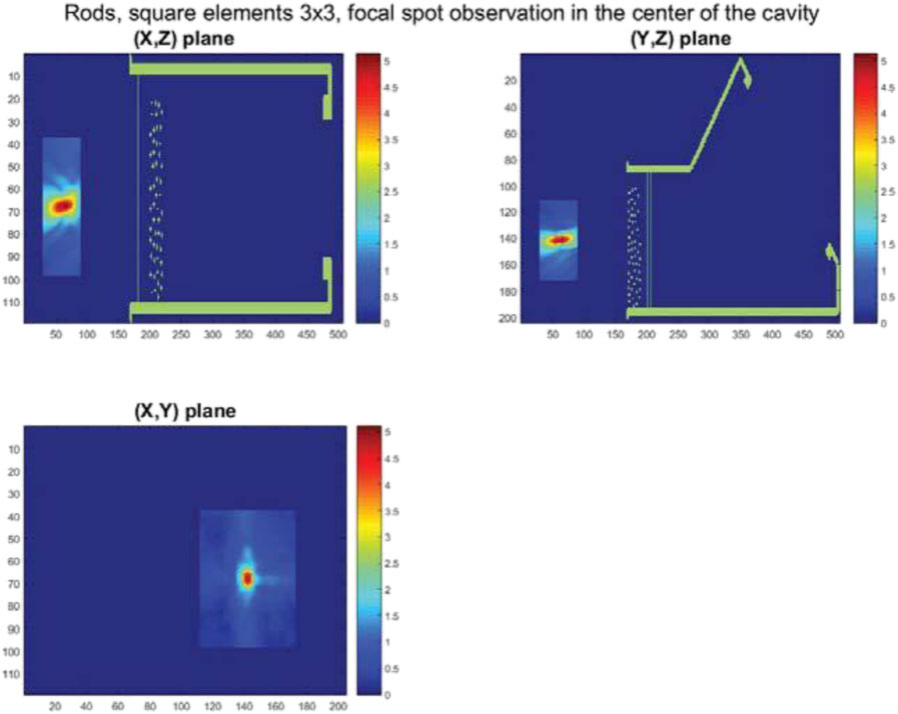
Focal spot observation in 3 orthogonal planes: local maximum pressure is given in arbitrary unit. Focal spot dimensions: 3x3x10 mm. Cross-sections of the time-reversal cavity and MSM are shown on the right in *light green*

Simulations showed that an array transducer of square elements 3 λ x 3 λ large, with an MSM made of 2 orthogonal very thin rod forests gave the best overall performances in terms of focus quality and steering. Figure 75 shows the focus quality in the centre of the cavity aperture. We thus tried to reproduce this configuration as well as possible in our experiments, but for practical reasons had to work with linear transducers instead.

**Fig. 76 (abstract O57).**
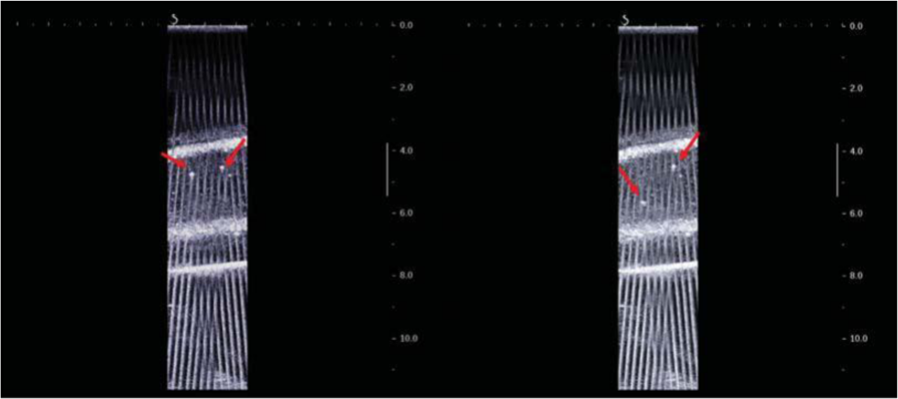
Bubble clouds formed by simultaneous targetting of 2 different focal spots on various positions

With our real device in a water tank, hydrophone measurements confirmed the spatio-temporal focalisation of the signal. Observations in a plane 10 cm away from the cavity showed a 1.2 x 1.2 mm focal spot, with a temporal peak less than 2 cycles long. At full power, the peak pressure obtained at the focus was about 40 MPa (linearly extrapolated value). These observations were consistent over a large area (−3 dB area 10x6 cm). The negative pressure obtained was sufficient to achieve cavitation. It was even possible to generate bubble clouds in various spots at the same time by emitting the stored signals corresponding to several locations successively at a PRF of 313 Hz between the different signals and between 16 and 260 Hz overall. Figure 76 shows the bubble clouds formed by the targeting of 2 simultaneous focal spots. We also succeeded in creating lesions in a slice of ham.

**Conclusions**

Through simulations and experiments, we designed and optimised a 3D-TRC that allowed us to very locally reach the high negative pressures needed to induce cavitation and create lesions in a simple target.

We are confident that we could further improve our experimental results if we fully exploit our simulation results, and move on to a transducer array.

### O58 Feasibility of transcutaneous volumetric boiling histotripsy ablation of liver and kidney in a pig model

#### Tatiana D. Khokhlova^4^, George R. Schade^1^, Yak-Nam Wang^2^, Wayne Kreider^2^, Julianna Simon^2^, Frank Starr^2^, Maria Karzova^3^, Adam Maxwell^1^, Michael R. Bailey^2^, Vera Khokhlova^2,3^

##### ^1^Department of Urology, University of Washington, Seattle, Washington, USA; ^2^Applied Physics Laboratory, University of Washington, Seattle, Washington, USA; ^3^Physics Department, M.V. Lomonosov Moscow State University, Moscow, Russian Federation; ^4^Department of Medicine, University of Washington, Seattle, Washington, USA

**Objectives**

Boiling histotripsy (BH) uses millisecond-long pulses of HIFU shock waves emitted at low duty cycle to induce localized boiling in tissue within each pulse. Further interaction of ultrasound with the vapor cavity for the rest of the pulse results in mechanical fractionation of tissue into subcellular debris. Our group is developing BH as a non-invasive treatment of renal and hepatic masses. The feasibility of producing single BH lesions *in vivo* in an exposed porcine liver has been demonstrated previously. The goal of the present work was to evaluate the feasibility and safety of transcutaneous, volumetric BH ablation of porcine liver and kidney in acute pig studies.

**Methods**

**Fig. 77 (abstract O58).**
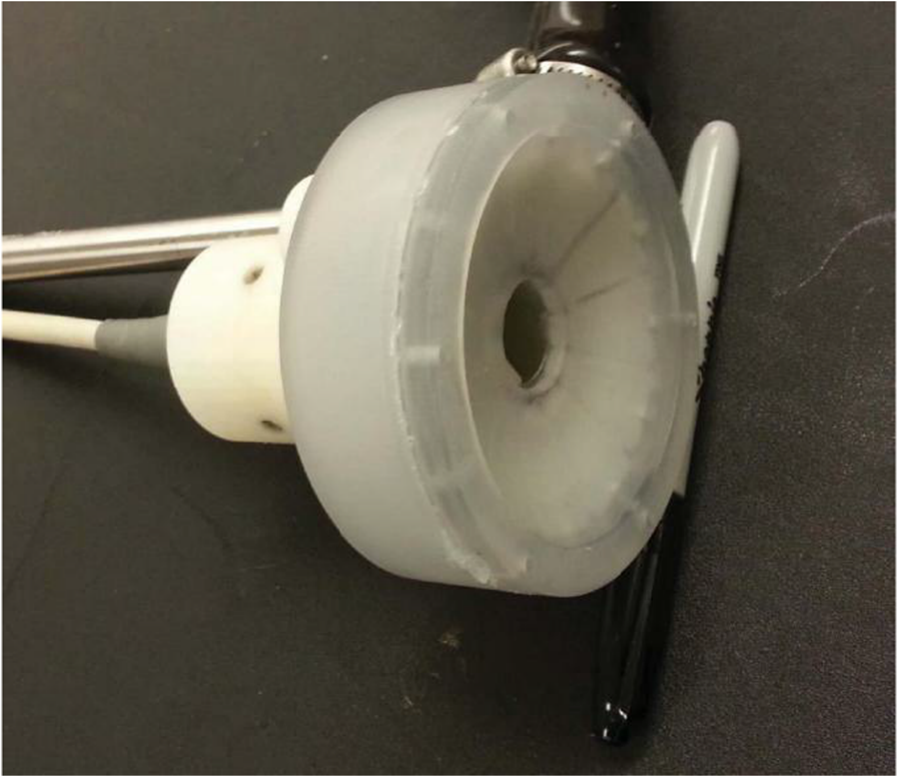
12-element 1.5 MHz HIFU sector array transducer integrated with an ultrasound imaging probe (ATL P7-4)

Pigs (37–40 kg, n=4) were anesthetized and placed on the surgical table in either lateral (for kidney treatment) or supine (for liver treatment) position. A 1.5 MHz HIFU transducer (12-element sector array of 7.5 cm aperture, F#=1.07) with a central opening (2 cm) to allow for ultrasound treatment guidance was attached to a 3D positioning system and submerged in a degassed water bath coupled to the abdomen (Fig. 77). The HIFU focus position, pre-recorded with the ultrasound imaging system, was aligned with the targeted region at the depth of 2.5-4.5 cm from the skin surface. The pulse-average power output threshold for initiating BH at each location was measured by sonicating the focal point with BH pulses at gradually increasing amplitude until a hyperechoic region was observed at the focus, indicating boiling. Prior to the *in vivo* experiments, similar measurements of threshold output power were performed in freshly harvested *ex vivo* porcine liver and kidney for comparison to the transcutaneous *in vivo* setting. The subsequent *in vivo* sonications were performed slightly above the threshold (10-15% increase in driving voltage). The following treatment parameters were used: pulse duration 5 or 10 ms, pulse repetition frequency (PRF) 1 or 2 Hz (with the duty factor fixed at 1%). A total of 10–30 pulses were delivered per focal point (this number will be further referred to as BH dose), with the focal points spaced 1–1.5 mm apart in a rectangular grid with 0.5-1.5 cm linear dimensions. The BH treatment was not gated by or synchronized with the respiratory motion. Following BH exposure, higher resolution ultrasound assessment of the treated regions was conducted. Necropsy was then performed and the treated portions of the liver and the kidneys were collected for gross and histologic assessment.

**Results**

**Fig. 78 (abstract O58).**
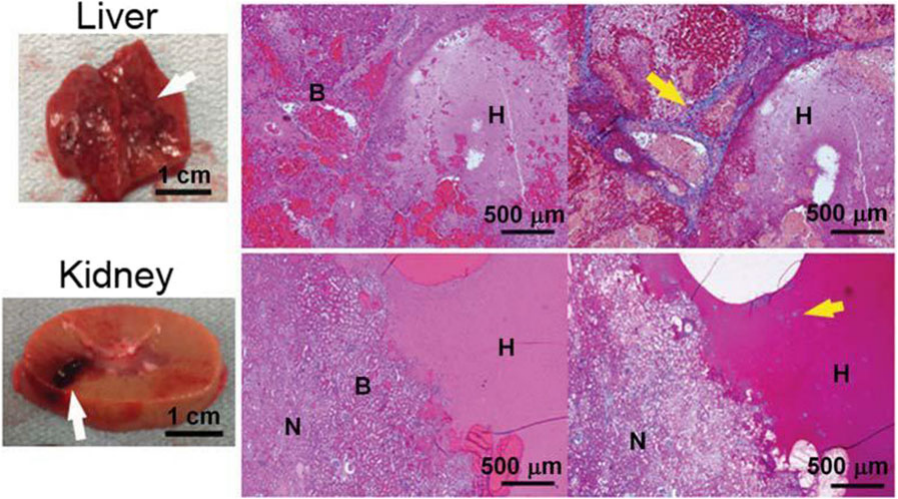
Representative photos (*left*) and tissue sections stained with hematoxylin and eosin (*middle*) and Masson’s trichrome (*right*) of porcine liver (*top*) and kidney (*bottom*) tissue treated transcutaneously *in vivo*. Photos show bisected BH lesions (white arrows) with the contents washed out. Histological evaluation reveals regions of completely homogenized tissue (H). Within the lesion, intact collagenous structures (*yellow arrow*) were present in both organs treated. In the kidney, distinct borders with normal tissue (N) were observed. Small haemorrhagic regions (b) were present in both tissues within and around the border of the lesions.

Kidney treatments. Lower poles of 7 kidneys were targeted and n=11 volumetric lesions containing cortex, medulla, and renal sinus were created. The transducer driving voltage required to initiate the subcostal transcutaneous treatment in the kidneys was 30 – 50% higher than that observed in the exposed *ex vivo* tissue; the partially transcostal exposures (30-40% of the beam obstructed by the ribs) required 120-150% larger driving voltage. The respiration-induced motion of the target did not appreciably interfere with the treatment Post-BH, higher resolution ultrasound images revealed well-defined hypoechoic cavities. At necropsy no gross evidence of collateral damage was appreciated within the beam path and no subjects had gross hematuria. On gross inspection of the kidney, small clots were seen within the collecting system in all treated kidneys with regions of petechial hemorrhage surrounding a centrally located fractionated volume of parenchyma. Histologically, all BH exposures produced completely fractionated cortex sharply demarcated from histologically normal untreated tissue (Fig. 78). In the medulla, blood was noted within the collecting ducts with areas of focally fractionated tissue at higher dose exposures (20–30 pulses per focal spot). Within the wall of the collecting system, focal petechial hemorrhage was visualized only at the higher dose exposures without disruption of the wall.

A treatment acceleration strategy was attempted, in which a smaller number (10 vs 30) of shorter (5 ms vs 10 ms) pulses were delivered per focal spot at higher PRF (2 Hz vs 1 Hz) at a larger driving voltage (15% increase). This strategy reduced the overall treatment time 6-fold (resulting in the lysis rate of 3.8 cc/hour), yet achieved the same degree of tissue fractionation as found with the slower treatment.

Liver treatments. Subcostal BH lesions were successfully produced in two out of three livers where treatment was attempted. The threshold for treatment initiation in terms of driving voltage was larger than in the *ex vivo* porcine liver by 70-200% and was also larger than in the transcutaneous kidney exposures despite very similar treatment depth and body wall thickness.

Most probably, the higher thresholds arose from the aberrative effects of fat within the HIFU beam, as the central section of the body wall contained a much thicker fat layer compared to that overlying the kidney (1.5 cm vs 0.5 cm). The respiration-induced motion of the target was much more pronounced compared to the case of kidney treatments, and led to a noticeable spread of the lesion relative to the planned shape. The hepatocytes in the central region of the lesion were completely fractionated, while at the lesion periphery the treatment effect was less demarcated. Connective tissue structure of the liver lobules, as well as the liver capsule remained intact, consistent with our *ex vivo* findings (Fig. 78). In the cases where higher power outputs were used (150-200% increase compared to the exposed *ex vivo* liver), bruising and thermal damage confined to the fatty layer of the body wall were observed.

**Conclusions**

These data indicate that transcutaneous and partially transcostal volumetric BH treatment of the kidney and liver is feasible in the porcine model. In the kidney, delivering shorter pulses at higher PRF and higher amplitude with constant duty cycle allowed for more rapid, yet equally efficacious tissue fractionation. In the liver, the lesions were successfully generated through a thicker fat layer, without control for respiratory motion. The treatment precision and efficacy can be further enhanced by implementing strategies for phase correction and gating based on respiratory motion. This work was supported by NIH R01 EB7643, K01 EB 015745, NSBRI through NASA NCC 9–58, and Urology Care Foundation.

### O59 Non-invasive, rapid ablation of large tissue volume using histotripsy

#### Jonathan E. Lundt, Steven P. Allen, Jonathan R. Sukovich, Timothy Hall, Zhen Xu

##### Biomedical Engineering, University of Michigan, Ann Arbor, Michigan, USA

**Objectives**

Current tumour ablation techniques are typically thermal-based, including radiofrequency (RF), microwave, and high intensity focused ultrasound (HIFU). RF and microwave ablation methods are limited to treating tumours no greater than 3 cm in diameter and at a rate of approximately 2 cm^3^/minute. While HIFU is capable of treating larger volumes, the treatment duration is excessive. Perfusion-mediated convection (commonly referred to as the “heat sink effect”) presents a major challenge for thermal ablation in highly vascularized tissues. The heat sink effect has been shown to prolong treatment times and result in heterogeneous tissue necrosis. Histotripsy is a noninvasive, non-thermal, ultrasound ablation method that uses high-amplitude, very low-duty cycle focused ultrasound pulses to generate controlled cavitation and thereby mechanically homogenize target tissues into liquid-appearing acellular debris. Our previous *in vivo* studies have shown that histotripsy is not affected by the heat sink effect and can produce homogenous tissue disruption in the highly vascular liver and kidneys noninvasively through the ribcage and other overlying tissues. Because histotripsy uses microsecond-duration pulses separated by up to seconds of off-time for a given focus, it is possible to electronically steer the focus of a phased array transducer to excite cavitation events throughout a large volume consisting of many overlapping foci during the off-time period. We hypothesize that histotripsy combined with electronic focal steering can achieve rapid ablation of a large target volume. As such, histotripsy can be used to treat tumours that cannot be treated by RF and microwave ablation at a rate exceeding that of these methods. This study presents the first investigation of this hypothesis.

**Methods**

Histotripsy was applied using a 250 kHz, 256-element phased array transducer with a 30 cm diameter aperture and 15 cm focal distance, generating 1.5-cycle, 6-microsecond acoustic pulses. First, to establish treatment parameters including pulse repetition frequency (PRF) and the number of pulses to deliver, a single-focus lesion was generated in tissue-mimicking phantoms. Tissue-mimicking agarose hydrogel phantoms containing a layer of red blood cells (RBC) allow direct visualization of cavitation and cavitation-induced damage. Cavitation activity and lesion progression during histotripsy treatment were monitored by high-speed optical imaging (Phantom V210, Vision Research) as a function of PRF and the number of pulses applied. Based on the RBC phantom results, *ex vivo* bovine hepatic tissue was treated by electronically scanning the therapy focus at 200 Hz over 1000 sites (or .2 Hz per focal site). 120 pulses were delivered per site to cover approximately 43 cm^3^ and 58 cm^3^ volumes of target tissue (equivalent to spheres 4.3 cm and 4.8 cm in diameter, respectively) over the course of a total treatment time of 10 minutes. The *in situ* peak rarefactional pressure amplitude was estimated to be 71 MPa at the geometric focus and 49 MPa at the most distal electronic steering foci. Lesion size and morphology were assessed by gross sectioning and magnetic resonance imaging (MRI). Tissue damage was examined by histology using haematoxylin and eosin (H&E) staining of 5-micron sections.

**Results**

**Fig. 79 (abstract O59).**
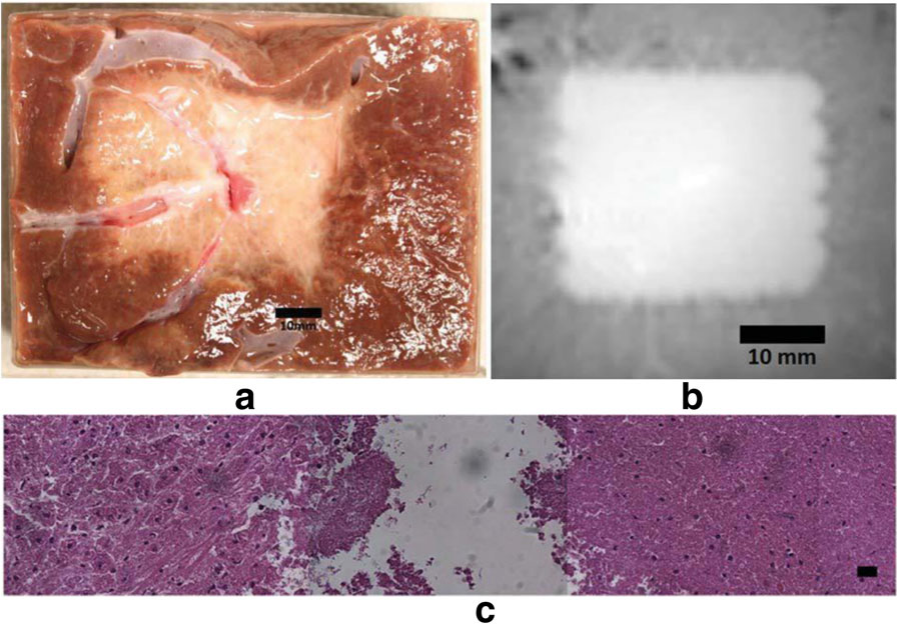
**a** Gross morphology of *ex vivo* bovine liver sample following treatment and irrigation of lesion with tap water. The acoustic axis of the transducer was oriented from left to right with respect to the image. Scale bar: 10 mm (**b**) MRI of *ex vivo* sample following treatment. The scan plane is orthogonal to the acoustic axis of the transducer and positioned at the geometric focus. Scale bar: 10 mm (**c**) H&E histology at 400X total magnification. Intact cells can been seen at the left of the image, fractionated cellular debris and nuclei at the right. Scale bar: 10 microns

RBC phantom results established that fractionation efficacy degraded at PRFs above 0.2 Hz and that 120 pulses were sufficient to homogenize material within the perimeter of a single lesion. Therefore, 0.2 Hz PRF and 120 pulses per single focus were selected for subsequent *ex vivo* tissue experiments using histotripsy with electronic focal steering. Morphology of the single lesion was approximated as an ellipsoid with an 8 mm major diameter and a 4 mm minor diameter. Based on the single lesion size, the center-to-center spacing between adjacent steering foci for electronic focal steering treatment was selected to be 2.5/3.2 mm in the lateral plane and 4.1 mm in the axial direction. A total of nine *ex vivo* bovine liver tissue samples were treated by histotripsy with electrical focal steering. Results from *ex* vivo experiments show that a completely homogenized and well-defined lesion was generated by histotripsy with electrical focal steering within 10 minutes for all nine treatments. Gross morphology sectioning (Fig. 79a) of *ex vivo* tissue shows a well-defined region of damage where the treated tissue was liquefied to homogenate. After rinsing away liquid-appearing material, only pale, fibrous structures and vessels larger than about 2 mm in diameter remain. MRI (gradient recalled echo sequence) (Fig. 79b) shows a distinct region of damage with sharp margins of individual foci clearly visible. Histology (Fig. 79c) shows a sharp transition zone (~50 microns) between cells with intact cell walls and a region of scattered cellular material and cell nuclei. For the smaller lateral spacing (2.5 mm), no intact cells remained in the treatment region, while a small fraction of scattered cells were observed in some treatments using larger spacing. MRI 3D volume measurements show the treatment volume to be 43 +/− 6 cm^3^ for the smaller spacing and 58 +/− 6 cm^3^ for the larger spacing (mean +/− standard deviation), yielding an average ablation rate of 4.3 and 5.8 cm^3^/minute, respectively.

**Conclusions**

Treatment of large and multiple tumour nodules remains a challenge for current tumour interventions, which are mostly thermal-based. This work demonstrates that histotripsy combined with electronic focal steering achieved homogenous and complete ablation of a large target volume at a rate two-fold faster than microwave and RF ablation. Since histotripsy is non-thermal, the treatment should not be affected by the heat sink effect and is expected to remain effective and efficient even in highly vascular organs. With the capability of achieving rapid, homogenous cell disruption, histotripsy has the potential to substantially improve upon current tumour ablation methods.

### O60 Assessment of boiling histotripsy dose in human ex vivo kidneys and renal tumours

#### George R. Schade^2^, Yak-Nam Wang^3^, Tatiana D. Khokhlova^1^, Philip May^2^, Daniel W. Lin^2^, Michael R. Bailey^3^, Vera Khokhlova^4,3^

##### ^1^Medicine, University of Washington, Seattle, Washington, USA; ^2^Urology, University of Washington, Seattle, Washington, USA; ^3^Appied Physics Lab, University of Washington, Seattle, Washington, USA; ^4^Physics, M.V. Lomonosov Moscow State University, Moscow, Russian Federation

###### **Correspondence:** Tatiana D. Khokhlova

**Objectives**

Histotripsy is a pulsed high intensity focused ultrasound (HIFU) technology that mechanically disrupts targeted tissue without a thermal effect. Our group has developed boiling histotripsy approach (BH), a technique that utilizes millisecond-long HIFU bursts to create bubbles at the focus via rapid shock-induced boiling. Interaction of subsequent shocks in the pulse with the ensuing vapor cavity mechanically homogenizes tissue into sub-cellular micron-sized debris in a process involving acoustic fountaining. As a noninvasive, non-thermal approach, BH may have several advantages over existing clinically available thermal ablative technologies for renal masses. Preliminary data suggests differential sensitivities to the effects of histotripsy of specific locations within the kidney, while the sensitivity of human renal tumours to the effects of BH is unknown. The aim of this study was to evaluate and compare the effect of BH on samples of freshly excised human renal tissues and associated tumours *ex vivo*.

**Methods**

Freshly excised human kidneys, benign renal tissue, and renal tumour tissue were obtained via IRB approved institutional rapid autopsy and tissue procurement programs. Tissue was obtained from n=11 patients: n=6 whole benign kidneys, n=5 fragments of benign parenchyma, and n=4 tumours (clear cell renal carcinoma (ccRCC): n=2, papillary RCC: n=1, oncocytoma: n=1). All specimens were acquired within 4 hours from death/nephrectomy. Tissue samples were degassed for over 30 minutes in phosphate buffered saline (PBS) and then embedded in low melting point agarose gel. Agarose embedded tissue was then placed in a holder in a bath of degassed PBS. BH exposures were performed under B-mode ultrasound guidance using a 1-MHz 7-element HIFU transducer (aperture 14.7 cm, F#=0.95) with the following pulsing protocol: pulse duration of 10 ms, pulse repetition frequency of 1 Hz, peak focal pressures of p+=88 MPa, p-=17 MPa, shock amplitude of 98 MPa. Single focal volumes within the tumour sample or the renal cortex, medulla, or collecting system were treated at various doses defined here as the number of pulses irradiated into a single focal spot (3–300 pulses/focus). Treated kidneys and tumour samples were evaluated grossly and/or formalin-fixed for histologic assessment with hematoxylin and eosin staining.

**Results**

**Fig. 80 (abstract O60).**
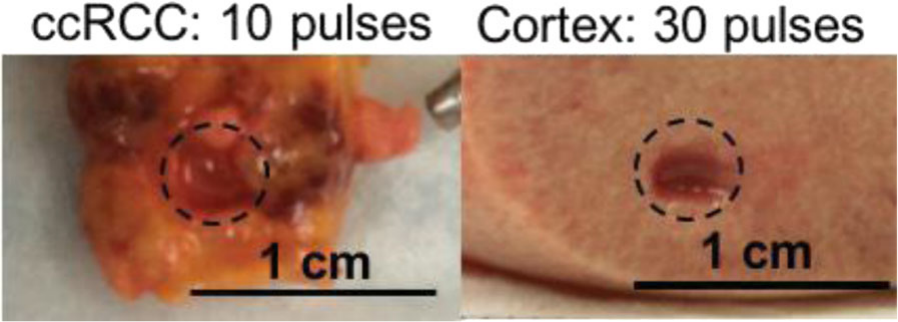
BH lesion produced in ex vivo human clear cell renal carcinoma (*left*) and benign human kidney cortex (*right*) with two different BH doses (10 and 30 pulses, respectively) are similar in size, demonstrating increased tumour tissue susceptibility to BH-induced damage compared to benign tissue

Bh pulses produced hyperechoic bubbles at the focus in all tissue types consistent with rapid boiling induced by each pulse. Treatment within the renal cortex and tumour tissue resulted in the development of progressively hypoechoic cavities apparent between pulses, consistent with historipsy treatment effect of homogenizing tissue, while the feedback was less pronounced in the medulla and collecting system. On inspection, tumour tissue appeared more susceptible to the effects of BH than benign tissue; lesions created in tumour tissue with 10 pulses were similar in size to those created with 30 pulses in the cortex (Fig. 80). Histologically, evidence of BH induced tissue homogenization was observed in tumours at much lower dose threshold of f 5 pulses/focus compared to those in benign tissues: 15–30 pulses/focus in the cortex, 45–60 pulses/focus in the medulla, and 90–120 pulses/focus in the collecting system.

**Conclusions**

BH mechanical ablation of human *ex vivo* renal tumours is feasible, yielding anticipated tissue homogenization. The observed increased resistance of benign renal tissue to the effects of BH compared to renal tumours, if confirmed *in vivo*, may help preserve renal function while providing a margin of safety when developing BH for clinical ablation of renal tumours. This work was supported by NIH R01 EB7643, K01 EB 015745, Urology Care Foundation and National Space Biomedical Research Institute (NSBRI) through NASA NCC 9–58.

### O61 A 200 kHz-1380 kHz multifrequency focused ultrasound transducer for neurostimulation in rodents: numerical study and transcranial *in-vitro* calibration

#### Charlotte Constans^2^, Thomas Deffieux^1^, Mickael Tanter^1^, Jean-Francois Aubry^3^

##### ^1^Institut Langevin, INSERM, Paris, France; ^2^Institut Langevin, Université Paris Diderot, Paris, France; ^3^Institut Langevin, CNRS, Paris, France

**Objectives**

In order to study the influence of the frequency on transcranial ultrasonic stimulation (TUS), we propose to take advantage of the harmonics of the transducer to investigate a large frequency span with one single element. We calibrated the transducer in the 200kHz-1380kHz range and performed numerical simulations to predict the pressure field that can be generated by this transducer in a rat brain for the same range of frequencies. The transducer was successfully used for neuromodulation experiments on rodents at 320kHz.

**Methods**

We calibrated a single element transducer with resonance peaks at 200kHz, 320kHz, 850kHz and 1380kHz (H115, Sonic Concepts, Bothel, USA) with a heterodyne interferometer. The pressure in the focal plane after transmission through pure degassed water was measured for each frequency with a power ranging from 18 to 75 electrical watts. Electrical power was generated by a function generator (Handyscope HS5, Tiepie Engineering Sneek, The Netherlands) connected to a 75 W amplifier (75A250A, Amplifier Research) and input voltage and current applied to the transducer were monitored with the channels of the Handyscope (Handyscope HS5, Tiepie Engineering Sneek, The Netherlands).

The acoustic propagation of focused ultrasound was then simulated in an entire rat head in order to investigate the pressure amplitude and spatial distribution as a function of frequency. The simulations were performed with k-Wave [1], a k-space pseudospectral method-based solver. 3D maps of the skull, brain and tissues were extracted from a rat microcomputed tomography scan. Brain and tissues were assumed to have the same sound-speed and density as water, and the transducer was modelled as a spherical section (63mm radius of curvature and 64mm active diameter) with the properties of ceramic. Rather than keeping the resolution constant when investigating the influence of frequency, we fixed the ratio of wavelength to the spatial step to approximately 12 for all simulations. Absorption was taken into account in the skull (2.7dB/cm/MHz) and in the brain (0.37dB/cm/MHz) with a 1.01 power law of frequency. A 230μs-long pulse was simulated, as was used in vivo with the same transducer [2]. Ultrasound propagate in a cone filled with water before entering the rat head, the geometrical focal point being located about 7mm deep from the surface, inside the brain. The simulations are first performed in pure water and compared to the amplitude measured experimentally: the scaling factor is used as a correction factor in order to estimate the absolute pressure in the rat head.

**Results**

The values of maximum pressure measured in degassed water and simulations in rat brain are summarized in Table 4. One can observe that the same setup is capable of producing more than 1MPa (respectively 1.4MPa) in pure water (respectively in the rat brain) for all frequencies ranging from 200 kHz to 1380kHz.

**Fig. 81 (abstract O61).**
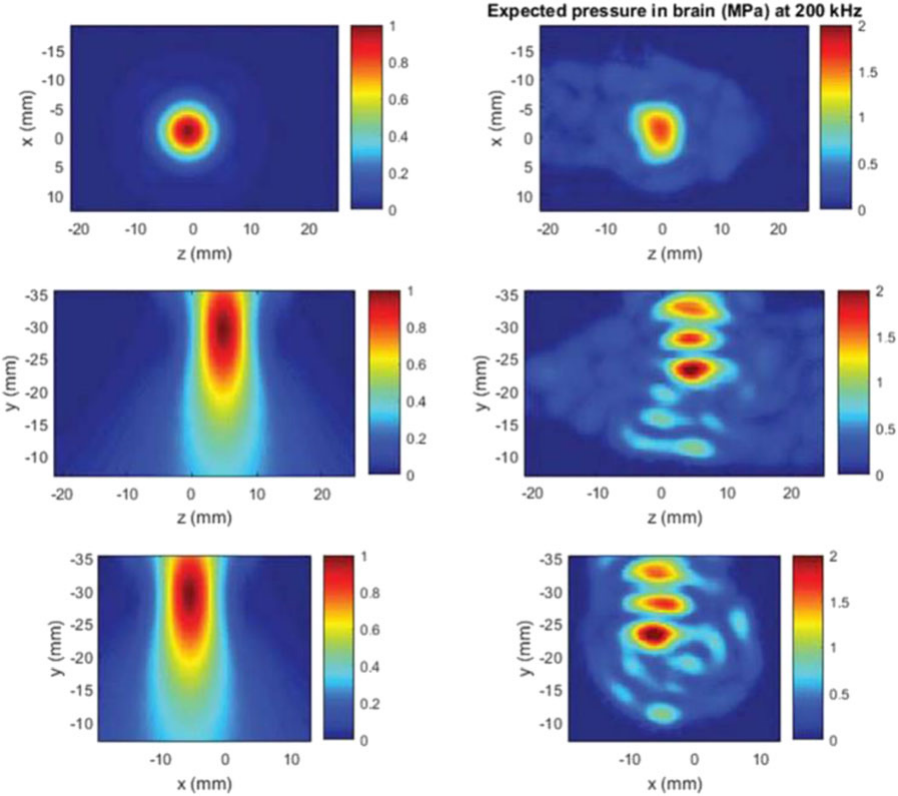
Peak pressure spatial distribution (MPa) in the coronal (*top*), sagittal (*middle*) and axial (*bottom*) planes, in water (*left*) and in rat brain (*right*) at 200kHz

The maximum pressure in the coronal (top), sagittal (middle) and axial (bottom) planes at the geometrical focal spot is displayed in Fig. 81 for 200 kHz (left) and 850 kHz (right) in a linear scale. As the propagation axis is along the y direction, top figures show the focal plane (view from above the animal) and middle and bottom ones include the propagation path. Stripes originating from standing waves can be seen for both frequencies (Fig. 81, middle and bottom) but are more confined in the 850 kHz simulations. In the axial plane the -6dB area is confined in a 27.5mmx49mm box at 200kHz and 9mm x 25mm at 850kHz.

**Conclusions**

Simulations show that the same transducer can be used to produce more than 1.4MPa in a rat brain at 200kHz, 320kHz, 850kHz and 1380kHz, which is higher than the threshold for in vivo TUS in rodents [2]. This work paves the way to exploratory work over a large bandwidth with one single experimental setup.

This work was supported by the Bettencourt Schueller Foundation and the "Agence Nationale de la Recherche" under the program “Future Investments” with the reference ANR-10-EQPX-15.

**References**

[1] B. Cox et al., k-space propagation models for acoustically heterogeneous media: Application to biomedical photoacoustics, J. Acoust. Soc. Am., 2007

[2] Y. Younan et al., Influence of the pressure field distribution in transcranial ultrasonic neurostimulation, Med. Phys., 2013

**Table 4 (abstract O61).** See text for description

	200 kHz	320 kHz	850 kHz	1380 kHz
Maximum pressure in water (MPa)	1.0	1.1	2.7	3.6
Estimated maximum pressure in rat brain (MPa)	2.2	1.4	2.8	3.9

**Fig. 82 (abstract O61).**
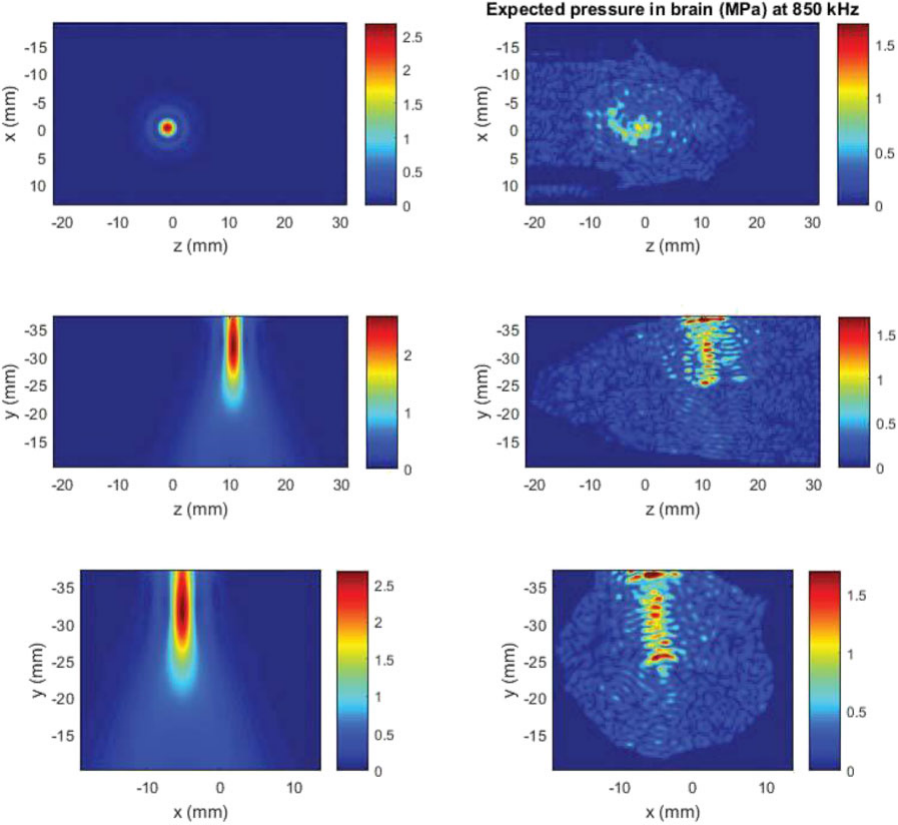
Peak pressure spatial distribution (MPa) in the coronal (*top*), sagittal (*middle*) and axial (*bottom*) planes, in water (*left*) and in rat brain (*right*) at 850kHz

### O62 *In vivo* study of enhanced chemotherapy combined with focused ultrasound for pancreatic cancer in animal model

#### Eun-Joo Park, Yun Deok Ahn, Soo Yeon Kang, Dong-Hyuk Park, Jae Young Lee

##### Radiology, Seoul National University Hospital, Seoul, Korea (the Republic of)

**Objectives**

As the effects of focused ultrasound (FUS) in anti-cancer drug delivery are widely studied, there is growing interest in the mechanism of how FUS enhances therapeutic effects of drug. In this study, *in vivo* experiment for pancreatic cancer in animal model was designed to investigate whether non-thermal effect of FUS more effectively enhances the chemo-treatment.

**Methods**

A pancreatic xenograft model was established by inoculating human pancreatic cancer cells (CFPAC-1) in BALB/c nude mouse. Animals were randomly assigned to the following six groups: control, gemcitabine (GEM) only, FUS1 only, FUS2 only, GEM with FUS1, and GEM with FUS2. Weekly treatments were performed for three weeks and post-treatment tumour size monitoring was followed for five weeks. For FUS treatment groups, animals were sonicated for 20 sec at 1MHz under the guidance of ultrasound images. In combined treatment of GEM and FUS, GEM was administered in IV immediately after the sonication. At the same total acoustic energy, acoustic power and the duty cycles were set in two FUS conditions. Acoustic power was 7.5 W for FUS1 and 80.5W for FUS2. Duty cycle for FUS1 and FUS2 was 50% and 5%, respectively.

**Results**

**Fig. 83 (abstract O62).**
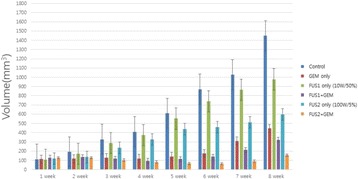
See text for description

Tumour growth rate of animals treated with FUS only (FUS1 & FUS2) was lower than the rate of control group while it was higher than the GEM only group. Animals treated with combination of FUS and GEM showed reduction of tumour growth after two treatments. In FUS2+GEM groups, tumour size reduced until five weeks after the treatment procedure was completed (Fig. 83).

**Conclusions**

In comparison to longer burst with relatively low acoustic pressure that might have thermal effects on tissue, short burst at high acoustic pressure more effectively control tumour growth in combination with chemo-agent. This result indicates that mechanical reaction induced by FUS can more effectively enhance chemotherapy for pancreatic cancer.

### O63 Focused ultrasound interventional oncology: local and regional control of advanced malignant disease is here to stay

#### Vidal-Jove, J.^1,4,5^; Perich, E.^4^; Ruiz, A.^1^; Jaen, A.^2^; Eres, N.^4^ ; Alvarez del Castillo, M.^3^

##### ^1^Surgical Oncology, HIFU Unit, Hospital Universitari Mutua Terrassa (HUMT), Barcelona, Spain; ^2^Research Unit, Hospital Universitari Mutua Terrassa (HUMT), Barcelona, Spain; ^3^Medical Direction Department, Hospital Universitari Mutua Terrassa (HUMT), Barcelona, Spain; ^4^Interventional Oncology Unit, Institut Khuab, Barcelona, Spain; ^5^HIFU Onco & Radiology Department, Clínica Santa Elena, Madrid, Spain

**Objectives**

Interventional Oncology has been proposed as a group of therapeutic procedures that are useful at obtaining local disease control with minimal invasive techniques. We describe our experience with Focused Ultrasound in different tumor settings as well as with different HIFU devices. The 8 years experience of the HIFU Surgical Oncology Unit of Hospital University Mutua Terrassa (Barcelona, Spain) and the 3 years experience of the Interventional Oncology Unit of Institute Khuab Barcelona treating malignant tumors have recently added a new setting for benign & malignant tumors in Santa Elena Clinic in Madrid (Spain). We compare our experience with the reported data in the literature with conventional treatments. We underline some considerations about the role of tumor ablation in the Western World stage.

**Methods**

From February 2008 to December 2015 we have treated more than 150 tumor cases. Of those, more than 50 cases of non-resectable pancreatic tumors were treated, and we include the first 45 patients from March 2010 to April 2015 to the analysis. All of them also underwent systemic chemotherapy with a standard combination. Devices employed were the JC system as well as the JC200 of USgHIFU from HAIFU Chongqing, China. We have recently added a Echopulse system from Theraclion, France.

**Results**

The distribution of the 150 cases treated reflects a majority of pancreatic and liver tumors. We specifically analyze the pancreatic tumors. Clinical responses (ablation obtained) were 82% in all cases. We obtained 11 complete responses (25%) at the end of the combined treatment. Major complications included severe pancreatitis (2), skin burning grade III that required plastic surgery (2), duodenal perforation (1). Median Survival is 18 month (6 mo – 3.4 year) and Overall Percent Survival is 33.5% at 5 year follow up.

**Conclusions**

Focused Ultrasound is an effective and safe Interventional Oncology ablation of benign & malignant tumors. Compared with reported data, it shows survival advantage in non- resectable stage III and IV pancreatic cancer. Interventional Oncology (tumor ablation) needs to be considered as a novel group of therapies along Medical, Radiation and Surgical Oncology and re-defined at the light of this experience.

### O64 Polymeric cups as nanoscale cavitation nuclei for active transport and enhanced delivery of nanomedicines into solid tumours

#### Rachel Myers^2,1^, James Kwan^1^, Christian Coviello^1^, Cliff Rowe^1^, Calum Crake^2^, Sean Finn^1^, Edward Jackson^1^, Robert Carlisle^2,1^, Constantin Coussios^2, 1^

##### ^1^OxSonics Ltd, Oxford, United Kingdom; ^2^Institute of Biomedical Engineering, University of Oxford, Oxford, United Kingdom

**Objectives**

All modern cancer nanotherapeutics, from antibodies and antibody-drug conjugates to oncolytic viruses, suffer from poor passive accumulation, limited penetration and non-uniform distribution in tumours. The present work seeks to exploit microstreaming mediated by sustained inertial cavitation to enable the active transport of biologics from the bloodstream deep into the tumour mass.

**Methods**

A new generation of nanoscale cavitation nuclei, known as polymeric cups, have been developed both for intra-tumoural and intravenous administration [1]. The cups have a mean diameter of 480 nm, and partially encapsulate and stabilize a single air nanobubble of typical diameter 200–300 nm. Upon exposure to 0.5 MHz ultrasound at in situ peak rarefactional pressures on the order of 1–2 MPa, the cups exclusively produce sustained broadband acoustic emissions associated with inertial cavitation, and generate sustained microstreaming capable of enhancing the transport of co-administered nanotherapeutics unbound to the cups. The cavitating cups can be mapped in real time using a conventional diagnostic ultrasound array and novel Passive Acoustic Mapping (PAM) algorithms capable of identifying sources of broadband acoustic emissions in real time during ultrasound exposure [2]. The present work investigates the usefulness of sustained cavitation mediated by sub-micron cavitation nuclei in enhancing the delivery of different types of oncolytic viruses and antibodies in vitro and to solid tumours *in vivo*.

**Results**

*In vitro* experiments consisted of a flow-through channel in an agar gel as previously described [3]. Little extravasation from the channel was observed in the absence of cavitation or in the presence of non-inertial cavitation mediated by ultrasound contrast agents. By contrast, the generation of sustained inertial cavitation activity mediated by the polymeric cups enables significant penetration of either small molecules, antibodies or viruses to >200 microns away from the vessel wall.

*In vivo* experiments were carried out using several cell lines and a variety of animal models, including CT-26 in BalbC mice, and HEPG-2 and SKOV in CD1-nude mice, first to quantify any enhancement in delivery and subsequent impact on survival for viruses and antibodies. Cavitation-enhanced delivery was found to enhance oncolytic virus activity in all cases, as quantified by both fluorescene/luminescence and qPCR, by 1–4 orders of magnitude depending on the type of virus being delivered. In separate experiments, the distribution of antibodies to tumours was found to be similarly enhanced, even though the intratumoural antibody dose could not be quantified accurately. Cavitation-mediated delivery significantly inhibited tumour growth both for viruses and for antibodies, and resulted in much more reproducible therapeutic responses across different subjects.

**Conclusions**

Cavitation-enhanced delivery using sub-micron cavitation nuclei, or polymeric cups, was found to significantly enhance the extravasation, delivery, intratumoural distribution and therapeutic efficacy of both antibodies and viruses for a given systemic dose. The ability to map cavitation activity in real time also offers significant opportunities for real-time monitoring and optimization of successful delivery. Future work will focus on optimizing combined drug and polymeric cup dosing regimes to maximize therapeutic benefit.

**References**

[1] Kwan et al., Small, 2015

[2] Coviello et al., JASA 2015

[3] Bazan-Peregrino *et al.*, J. Controlled Release 2012

### O65 Rapid short pulse (rasp) sequences improve cavitation dynamics for ultrasound therapy

#### Antonios Pouliopoulos^1^, Caiqin Li^1^, Marc Tinguely^2^, Meng-Xing Tang^1^, Valeria Garbin^2^, James J. Choi^1^

##### ^1^Bioengineering, Imperial College London, London, United Kingdom; ^2^Chemical Engineering, Imperial College London, London, United Kingdom

**Objectives**

Acoustic cavitation – the volumetric oscillation of a bubble due to an acoustic field – is a mechanical force harnessed in therapeutic ultrasound to treat diseases. It can dissolve clots, deliver drugs into cells, deliver drugs across capillaries (e.g., blood–brain barrier opening), and release drugs from liposomes, but it can also cause haemorrhage, kill cells, elicit an immune response, and damage tissue. Current ultrasound parameters have limited control over acoustic cavitation due to our lack of understanding of how microbubbles behave in therapeutically relevant acoustic fields. We introduce a rapid short pulse (RaSP) sequence that has better control of cavitation (e.g., its distribution) than conventional ultrasound parameters. A subset of this design has been previously shown to produce greater therapeutic benefits (e.g., improved drug distribution) in the context of blood–brain barrier opening (Choi et al., PNAS 2011). In order to demonstrate improved dynamics of our RaSP sequence over conventional parameters, we have performed a multi-dimensional analysis of cavitation (e.g., type, magnitude, duration, distribution) using passive acoustic mapping and a high-speed microscopy.

**Methods**

Traditional therapeutic parameters are composed of long pulses (10–100 ms), which result in microbubble displacement in the axial direction due to a primary acoustic radiation force, clustering due to secondary acoustic radiation forces, coalescence, rectified diffusion, fragmentation, and a variety of other effects due to acoustic cavitation. Many of these effects are undesired sources of mechanical stress that can cause damage. To avoid these effects, we have significantly reduced the length of our pulses (0.01 ms) while increasing the pulse repetition frequency (10,000 Hz). Our pulse shape and sequence exploits the presence of flow by facilitating microbubble movement between pulses. A 0.5MHz focused ultrasound transducer was used to sonicate (PRP: 146-900kPa, PRF: 0.62-10kHz, PL: 5, 25, 50, and 50,000 cycles) microbubbles (SonoVue) flowing within a 800-μm diameter tube, while a ATL L7-4 linear array was used to capture acoustic emissions generated by the cavitation activity. The type, magnitude, distribution, and duration of cavitation activity was analysed using passive acoustic mapping and spectral analysis while a smaller subset of parameters was analysed using a high-speed microscope (5,000 frames per second).

**Results**

Cavitation persistence during short-pulse excitation increased by 5-fold at low pressures (<150kPa) when compared to a 100-ms long pulse. High pressures and long pulse lengths produced high magnitude inertial cavitation during the first millisecond, which rapidly decreased in energy due to destruction of cavitation nuclei. Cavitation activity was then biased upstream from the focal point due to new microbubbles entering the focal volume. High-speed microscopy observations revealed rapid displacement, clustering, and coalescence at these parameters. Low pressures and short pulse lengths resulted in a more consistent magnitude and distribution of cavitation activity throughout the sequence (figure). High-speed microscopy observations revealed reduced clustering rates and reduced axial displacements.

**Conclusions**

In conclusion, low-pressure rapid short pulse sequences improved the uniformity of cavitation within the focal volume when compared to long pulses. This improvement was due to the increased lifetime and mobility of the microbubbles within the focal volume. Our demonstration of improved spatio-temporal control of cavitation may improve the efficacy of a wide range of therapeutic applications such as blood–brain barrier opening, sonoporation, and sonothrombolysis, by enhancing therapeutically relevant cavitation dynamics and eliminating unwanted mechanical stress.

**Fig. 84 (abstract O65).**
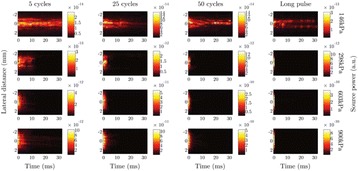
Improved magnitude and distribution of cavitation using rapid short pulse (RaSP) sequences. SonoVue microbubbles flowing through an 800-μm-in-diameter tunnel was exposed to a beam of ultrasound (laterial FWHM: 5 mm). Each image represents the lateral distribution of acoustic cavitation activity over time for different exposure conditions (peak-rarefactional pressures: 146–903 kPa, pulse lengths 5, 25, 50, and 16,666 cycles, pulse repetition frequencies: 1,250 Hz)

### O66 Early clinical experience of targeted delivery of lyso-thermosensitive liposomal doxorubicin (LTLD) by focused ultrasound to the liver

#### Paul C. Lyon, Christophoros Mannaris, Michael Gray, Lisa Folkes, Michael Stratford, Robert Carlisle, Feng Wu, Mark Middleton, Fergus Gleeson, Constantin Coussios

##### University of Oxford & Oxford University Hospitals NHS Foundation Trust, Oxford, United Kingdom

###### **Correspondence:** Paul C. Lyon

**Objectives**

The TARDOX study (Oxford, UK, NCT02181075) is a Phase I first-in-man proof-of-concept study which aims to demonstrate safety and feasibility of targeted drug delivery using lyso-thermosensitive liposomal systems in combination with mild hyperthermia mediated by focused-ultrasound (FUS) applied non-invasively. The primary endpoint of the study is demonstration of enhanced intratumoural delivery of doxorubicin to liver tumours for the same systemic dose of the drug, when given in liposomal form (ThermoDox®) and released locally by ultrasound-induced mild hyperthermia.

**Methods**

In March 2015, a patient with hepatocellular carcinoma and several liver lesions was recruited to the study and received a single treatment cycle of 50mg/m^2^ ThermoDox® and FUS. Following an ultrasound-screening session, a single tumour was selected for FUS-targeting, estimated to be of volume 13.7cm^3^ using a volumetric approximation based on MRI taken the day prior to treatment.

On the day of treatment, the patient was positioned supine over the water bath of the clinical ultrasound-guided extracorporeal FUS device (Model JC200 Focused Ultrasound Tumor Therapeutic System, Haifu Medical). General anaesthesia was induced using high-frequency jet ventilation to minimise respiratory movements of the liver. The water temperature was 14.5°C and patient normothermia was maintained using a controllable heated blanket (Bair Hugger™). Under portable ultrasound guidance, an 18-gauge co-axial needle was placed percutaneously (above the water level) into the core of the target tumour through a sterile field. The central co-axial needle was instrumented with a clinically approved thermistor, interchanged with a core biopsy device according to a treatment protocol. A second, peripheral co-axial needle was placed near to the tumour margin and was used only for thermometry.

Immediately following the 30-minute intravenous ThermoDox® infusion, the JC200 was used to induce hyperthermia in the target tumour using a transcostal approach under conditions of real time thermometry. Thermometry data was acquired via a data acquisition unit (Agilent HP34970A) connected to a PC running a custom LabView client.

The FUS hyperthermia strategy and parameters were selected based on thermometry obtained from previous *ex vivo* liver tissue using the same system (presented at 3rd European Symposium on FUS Therapy, London 2015). Alignment of the JC200 to the central thermistor was confirmed by low-power test shots. Once the target tumour was contoured through the intercostal space, FUS coverage was planned automatically using single shot mode with a 2mm dot interval, over 11 slices each of 2mm thickness, giving a prescribed volume of 10.5cm^3^ . The tumour was treated at 50 Watts, 100% duty cycle, row-by-row and slice-by-slice. Rather than heating slices contiguously, a slice separation of four was used in an attempt to dissipate heat more rapidly across the volume and achieve a more uniform bulk temperature rise.

Core tumour biopsies were taken a) prior to drug infusion, b) following completion of drug infusion, and, c) following FUS, for analysis of intratumoural doxorubicin concentration. Biopsy samples were taken in pre-weighed and watertight eppendorfs, which were reweighed before analysis. Samples were frozen at −80°C until the day of analysis, when they were thawed and homogenized. Daunorubicin was added into each sample pot before extraction as an internal standard. Solvent extraction was used to obtain both anthracyclines and their metabolites. A validated high performance liquid chromatography (HPLC) assay with fluorescence detection was used to establish the intra-tumoural concentration of doxorubicin. The ratio of the area under the curve for doxorubicin : daunorubicin was used to calculate the doxorubicin concentrations per gram of tissue. Chromatograms were obtained using Gemini C6-Phenyl guard and analytical columns with Waters 2695 separations Module and 474 fluorescence detector (Watford, UK) with excitation 480nm and emission 560nm.

Dynamic contrast enhanced (DCE) MRI, perfusion CT and 18F-FDG PET-CT imaging was performed the day prior to treatment and at day 17 and 29 post-treatment. An additional DCE MRI was performed the day following treatment. Response evaluation was performed using principles of RECIST & CHOI and the SUVmax metric for the target lesion.

**Results**

The patient recovered smoothly from anaesthesia and was discharged the following day following clinical review, MRI and blood tests. During the 30-day follow-up period, blood tests were taken at two and four weeks and no adverse events over grade two were reported (NCI CTCAE V4 toxicity criteria).

On the treatment day, following the drug infusion, one complete treatment cycle (354 units) was delivered, followed by a partial cycle (68 units) to maintain hyperthermia over 30 minutes. Post-drug, the JC200 reported an output of 21.1KJ (50W x 422s), taking 32 minutes in real time. Thermometry analysis showed the bulk intratumoural temperature was maintained >40°C for 14m 13s, and >41°C for 50s. During the FUS treatment period, mean and maximal temperatures were 39.8°C and 41.8°C respectively. The peripheral temperature was maintained >38°C for 2m 42s with mean and maximal temperatures of 37.7°C and 38.8°C respectively.

HPLC analysis for the biopsy taken prior to drug administration demonstrated absence of a doxorubicin peak. Following FUS, HPLC revealed a greater than two-fold increase in intra-tumoural doxorubicin concentration, from 2.56 to 5.32μg/g.

Both intra-procedural ultrasound and day 1 MRI demonstrated absence of any changes suggestive of thermal ablation. Subsequent radiological analysis of the target tumour over the four-week period revealed a decrease in attenuation from 75 to 28 Hounsfield units on CT and a reduction in longest axial dimension from 35mm to 25mm on CT and 34mm to 26mm on MRI. PET analysis demonstrated a drop in SUVmax from 4.5 to 3.4-3.8. No such response was seen in control tumours.

**Conclusions**

The use of LTLD with extra-corporeal FUS hyperthermia for targeted drug delivery in human liver tumours is feasible and may enhance delivery of doxorubicin and its therapeutic efficacy over LTLD alone. Further cases treating tumours of a variety of histological types, size and anatomical locations are needed to support this hypothesis and collate safety data.

### O67 RNA-based gene delivery using clinical lithotripter shock waves

#### Sandra Nwokeoha, Robert Carlisle, Robin Cleveland

##### Institute of Biomedical Engineering, University of Oxford, Oxford, United Kingdom

**Objectives**

Nucleic acid-based therapies provide a powerful approach to the treatment of genetic diseases but are challenged by limited delivery. Thus efficient gene delivery strategies are continually being sought. Here mRNA delivery is investigated as, in contrast to the commonly delivered plasmid DNA, mRNA does not require opening of the nuclear envelope, thereby reducing the level of cell injury necessary for transfection. We present for the first time an investigation on the efficacy of lithotripter shock waves (SW) assisted mRNA delivery, based on optimised post-exposure RNA stability and shock wave parameters, in a tissue mimicking system. In addition, we compare SW induced gene augmentation to gene inhibition through the delivery of siRNA.

Furthermore we aimed to determine the transfectability of dissimilar cell types as a function of acoustic pressure and number of SW, to provide insights into the breadth of applicability of SW in the mediation of various gene therapies.

**Methods**

To optimise SW treatment for optimal transfection, a baseline set of 24 SW conditions was established (n=9 per condition). Three cell lines varying by disease, organ and organism origin were cultured, suspended in continually degassed water and spatially subsumed by the focal zone of a clinical Storz Modulith SLX-F2 electromagnetic shock wave source. The focal volume was measured to be 7.85 mm x 7.85 mm x 42.40 mm. The acoustic exposure parameters comprised peak positive (8.6 – 37.0 MPa) and negative (4.1 – 7.0 MPa) pressures, 125 –1000 shock waves and 1–2 Hz PRF (up to 4 Hz at lower energies due to the capacitance of the shock wave source). Transfectability was assessed as the proportion of permeabilised cells (as assayed by propidium iodide (PI) inclusion) above the proportion of non-viable cells (as assessed using the MTS assay). The structural and biological stability of eGFP RNA was determined by gel electrophoresis and a cell-free in vitro translation method, respectively. Transfections were measured by fluorometry and conducted using a 2 mL tissue mimicking system in which cells embedded in 1% purified agar gels were compartmentalised from a 2.5 mm radius RNA-incorporated channel.

**Results**

**Fig. 85 (abstract O67).**
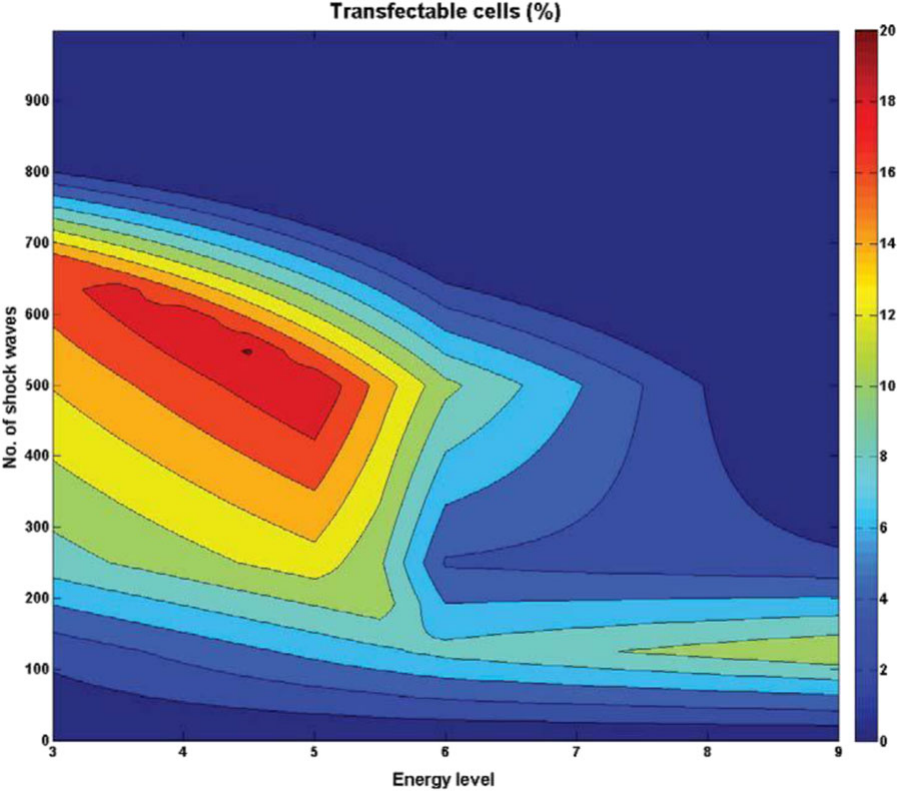
Transfectability of murine colorectal carcinoma cells as a function of the number of shock waves (0 – 1000) and lithotripter energy level 3 (P^+^ = 8.6 MPa) to 9 (P^+^ = 37.0 MPa) at 2 Hz

**Fig. 86 (abstract O67).**
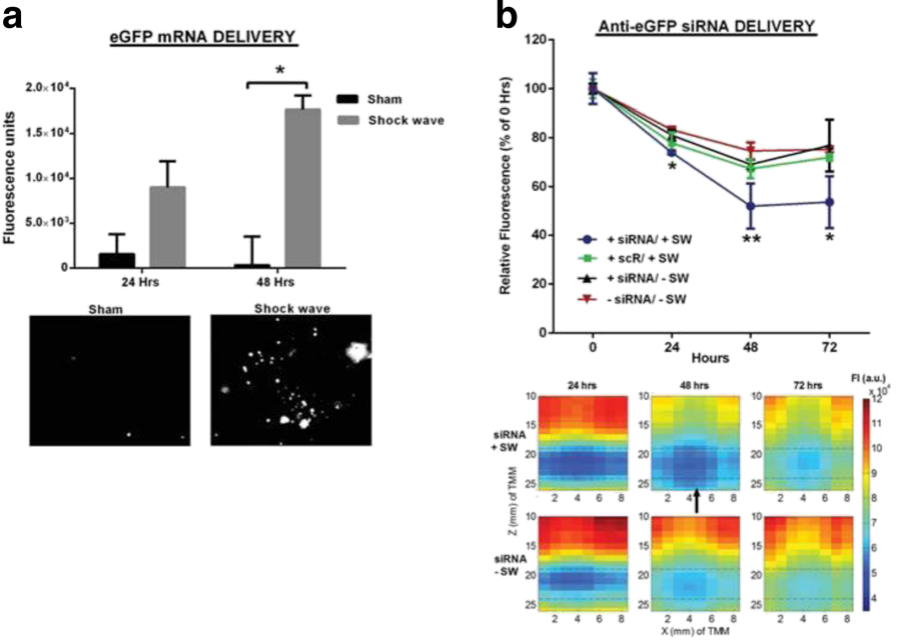
Enhancement of lithotripter SW induced RNA nucleotide delivery. **a** GFP mRNA delivery using optimal shock wave parameters resulted in a 6- and 52- fold increase in GFP fluorescence intensity relative to sham at 24 and 48 hours respectively. A statistically significant difference (*) was found at the p< 0.05 level. The flourescence were taken at 48 hours. **b** representative GFP intensities of GFP siRNA shock wave treated cells (+ siRNA/ +SW) scramble siRNA shock wave treated cells (+ scR/ + SW), sham cells (+ siRNA/ - SW) and controls ( - siRNA/- SW). Below are the flourescence intensities across the tissue phantom ROI; the dashed lines demarcate the RNA- incorporated channel. The black arrow represents the direction of SW propagation.

Transfectability was determined at the tested shock wave conditions and 2-D interpolation used to determine the optimal SW dose for maximal cellular uptake per cell type (murine colorectal carcinoma cells shown in Fig. 85). Permeabilisation of normal human kidney cells showed little correlation to SW parameters while poor cell viability recovery at 24 hours for human breast cancer cells resulted in marked cell damage. No statistically significant difference (p<0.05) was found between stabilities of sham RNA and optimal SW exposed RNA. Delivery of eGFP mRNA as measured by expression was enhanced 52-fold by SWs relative to sham treatment (Fig. 86a). A 2-fold decrease in GFP expression was achieved following SW–mediated eGFP siRNA delivery to human breast cancer cells stably expressing GFP (Fig. 86b).

**Conclusions**

While SWs did not discriminate between normal cells and the characteristically permeability-enhanced cancer cells, optimal SW treatment was cell type specific. Transfection results suggested that SWs may be a mechanism for achieving gene augmentation, by allowing RNA stability and significantly enhanced target protein expression in the absence of external cavitation.

### O68 Focused ultrasound enhancement of drug delivery in a pancreatic cancer mouse model

#### Yak-Nam Wang^1^, Tatiana D. Khokhlova^1^, Tong Li^1^, Navid Farr^1^, Samantha D'Andrea^1^, Frank Starr^1^, Kayla Gravelle^1^, Hong Chen^1^, Ari Partanen^2^, Donghoon Lee^1^, Joo Ha Hwang^1^

##### ^1^University of Washington, Seattle, Washington, USA; ^2^Philips, Bethesda, Maryland, USA

**Objectives**

Pancreas cancer remains one of the deadliest of all types of cancer and the most difficult to treat. It is currently the fourth leading cause of cancer death in the United States, and is anticipated to become the second by 2020. Unlike many other cancers, the survival rate for pancreas cancer has not improved substantially, with the five-year survival rate over the past few decades only increasing from 2 to 6%. Current treatment with conventional chemotherapeutics is ineffective due to the presence of extensive stromal desmoplasia and reduced vascular network impeding delivery of chemotherapeutic agents. Our group has evaluated two effects of focused ultrasound (cavitation and mild hyperthermia) to enhance the penetration of chemotherapeutic drug doxorubicin in a genetically engineered mouse model (KPC mouse) of pancreatic ductal adenocarcinoma. This abstract presents the culmination of this work to date.

**Methods**

All experimental procedures were approved by the Institutional Animal Care and Use Committee of the University of Washington. A KPC transgenic mouse model was used in all these studies. This model closely recapitulates the genetic mutations, clinical symptoms and histopathology found in human pancreas cancer. The tumours have a differentiated ductal morphology with extensive dense stromal matrix and poorly developed vasculature. Doxorubicin was used as the chemotherapeutic agent as a proxy to gemcitabine (current standard of care), due to the ease of detection and the range of clinically approved available forms.

Cavitation. A preclinical focused ultrasound system (VIFU 2000, Alpinion Medical Systems), was used for treatment planning, to apply pHIFU exposures, and monitor cavitation during treatment. The system used either a 1.1 or 1.5-MHz transducer, both of which had a circular central opening of 38-mm diameter fitted with a focused ring-shaped transducer for PCD and an ultrasound imaging probe (C4-12 phased array, center frequency: 7-MHz, Alpinion Medical Systems) for in-line targeting of the tumour. HIFU focal pressures were applied between 1.6–12.4 MPa and 2.2–17 MPa for the 1.1- and 1.5-MHz transducers, respectively. Passive cavitation detectors (PCD), aligned confocally with the HIFU transducers, were used to record broadband emissions from bubble activity during treatment. The following cavitation metrics were calculated from the acquired PCD signals: cavitation probability, cavitation persistence and broadband noise level. Doxorubicin (Dox) was administered during or post pHIFU treatment. The enhancement of drug uptake in the treated area of the tumour was evaluated by multispectral imaging, fluorescence microscopy and high-pressure liquid chromatography (HPLC). The untreated area of the same tumour was used as an internal control. Control animals were not treated with pHIFU.

Mild Hyperthermia. A clinical Magnetic Resonance-guided High Intensity Focused Ultrasound (MR-HIFU) system (Sonalleve V1, Philips, Vantaa, Finland) with a 256-element phased array transducer (focal length 12 cm, frequency 1.2 MHz) was used to apply the focused ultrasound exposures. Therapy planning and real time temperature monitoring was performed using a clinical magnetic resonance imaging (MRI) system (Achieva 3T, Philips, Best, the Netherlands) and a dedicated small animal MR receive coil. The MR sequence was a 2D echo planar fast field echo (FFE-EPI) pulse sequence (TR = 50 ms, TE = 20 ms, flip angle = 20°, voxel = 0.9 x 0.9 x 4.0 mm^3^, FOV = 100 x 100 mm^2^, EPI-factor = 7, parallel imaging (SENSE) factor = 2 (RL), saturation bands = 3, dynamic scan time = 1.8 s). Mild hyperthermia treatments were applied (continuous wave ultrasound, acoustic power 7 W) for 10 – 15 minutes, with a binary feedback control algorithm keeping the temperature between pre-defined threshold temperatures (Tmin = 41°C, Tmax = 42.5 °C).

Non-liposomal doxorubicin (Dox) or doxorubicin in the form of a low temperature sensitive liposome (LTSL-Dox) was administered before treatment. The enhancement of drug uptake in the treated area of the tumour was evaluated by fluorescence microscopy and HPLC. Mice not treated with mild hyperthermia were used as controls.

**Results**

**Fig. 87 (abstract O68).**
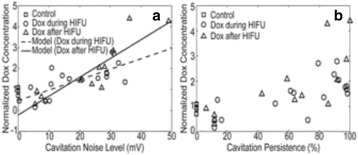
Scatter plot of normalized Dox concentration (the outcome) versus cavitation noise level (**a**) and cavitation persistence (**b**). The outcomes tend to increase with both the persistence and the noise level. The data from control group (*squares*), outcomes from the simultaneous treatment group (*circles*) as well as from sequential treatment group (*triangles*) are shown. The result lines from generalized estimating equation (GEE) model of cavitation noise level (**a**) and cavitation persistence (**b**) are also plotted for the simultaneous pHIFU treatment and Dox administration (*dash line*) and the Dox administration after pHIFU treatment (*solid line*)

Cavitation. Above the cavitation threshold, the doxorubicin concentration in the treated regions of the tumour was significantly greater compared to the controls. The normalized doxorubicin concentrations were found to be associated with the cavitation metrics (Fig. 87). The pHIFU exposures associated with high cavitation activity resulted in disruption of the stromal matrix and enhanced the concentration by up to 4.5-fold compared to control animals. The increase in drug concentration was supported by both multi-spectral imaging and fluorescence microscopy.

**Fig. 88 (abstract O68).**
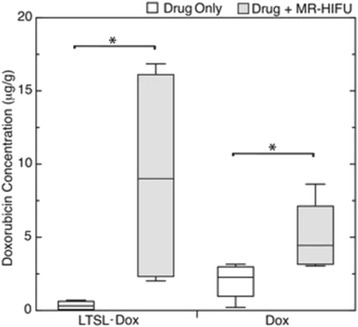
Box-and-whisker plot of doxorubicin (Dox) concentration in tumours of KPC mice treated with low temperature sensitive liposomal doxorubicin (LTSL-Dox) and non-liposomal doxorubicin (Dox), with and without the application of MR-HIFU. * denotes significance at the p < 0.05 level

Mild Hyperthermia. The MR-HIFU system enabled localized upkeep of mild hyperthermia within a tight temperature range (41.2 ± 1.3°C) to a target tissue area 6 mm in diameter. Hyperthermia induced by ultrasound increased the median doxorubicin concentration within tumour tissue (Fig. 88) when applied in combination with the systemic administration of low temperature sensitive liposomal doxorubicin (up to 15-fold) or non-liposomal doxorubicin (up to 2-fold) with no significant differences in cardiac levels of doxorubicin. The increase in drug concentration in LTSL-Dox + hyperthermia and Dox + hyperthermia treated animals was supported by fluorescence microscopy. None of the tumours showed damage caused by the application of hyperthermia.

**Conclusions**

Focused ultrasound can be used to induce cavitation or mild hyperthermia to significantly increase the concentration of doxorubicin into pancreas tumours in the KPC mouse model. The promising results in these studies demonstrate two separate ultrasound-induced techniques that can be used to overcome the barriers to drug penetration in pancreas tumours. This work was supported by the Focused Ultrasound foundation (grant AM01) and US National Institutes of Health (NIH R01CA154451) from the National Cancer Institute (NCI).

### O69 Antitumoural effect of bisphosphonates in breast cancer xenografts and bone metastasis is promoted by low-intensity ultrasound

#### Sophie Tardoski^1^, Jacqueline Ngo^1^, Evelyne Gineyts^2^, Jean-Pau Roux ^2^, Philippe Clézardin^2^, David Melodelima^1^

##### ^1^LabTAU - U1032, INSERM, Lyon, France; ^2^Lyos - U1033, INSERM, Lyon, France

**Objectives**

Bisphosphonates (BP) like zoledronic acid (ZOL) have demonstrated clinical utility in the treatment of patients with bone metastases. However, ZOL exhibits antitumour effects only at high doses incompatible with a clinical use due to renal toxicity. Bisphosphonates exhibit a high affinity for bone mineral, which reduces their bioavailability for tumour cells. We examined if low intensity ultrasound could enhance the effects of a clinically relevant dose of ZOL in experimental breast cancer and bone metastasis murine models.

**Methods**

A plane transducer working at a frequency of 2.9 MHz was used. The free field acoustic power was adjusted between 8 and 13 watts applied for 30 minutes in order to produce and maintain mild hyperthermia (43°C). These parameters enhance locally the temperature in mice and produce mechanical stimulation without creating cavitation. In vivo experiments were performed in a bone metastases model and on a subcutaneous tumour xenograft model. Animals were randomly assigned to different groups (vehicle, ZOL, US, ZOL+US). Clinically relevant dose of ZOL was used (100 μg/kg). Osteolytic lesions were detected by radiography. Tumour angiogenesis and tumour cells proliferation were assessed by immunohistochemistry. Unprenylated Rap1A, a surrogate marker of the penetration of ZOL into tumour cells, was observed by Western Blotting. A quantification of remaining bisphosphonate in bone after ultrasonic treatment was performed using a fluorescent bisphosphonate (FAM-RIS).

**Results**

With the acoustic parameters used, no signs of cavitation were found. Temperature in tumours was 42.0 ± 2.8°C during US treatment. No lesion was observed in surrounding tissues. US alone did not have any effect on bone metastasis and tumour outgrowth. In the bone metastasis model, mice treated with ZOL+US had osteolytic lesions that were 58% smaller than those of ZOL-treated animals (p<0.01). ZOL+US also significantly decreased skeletal tumour burden. In the animal model of primary tumours, ZOL+US treatment reduced by 42% the tumour volume, compared with ZOL-treated animals (p<0.01). In all cases tumour angiogenesis and tumour cell proliferation were reduced. Using a fluorescent bisphosphonate, it was demonstrated that US forced the release of bisphosphonate from the bone surface, enabling a continuous impregnation of the bone marrow. Additionally, US forced the penetration of ZOL within tumours, as demonstrated by the intratumoural accumulation of unprenylated Rap1A.

**Conclusions**

In conclusion, our results demonstrate the potential of low intensity ultrasound as an effective strategy to force bisphosphonate desorption from bone and its penetration through tumour tissue, enabling bisphosphonate antitumour activity (both in bone and outside bone). Our findings made US a promising modality in oncology to trigger anticancer therapy with bisphosphonates.

### O70 Characterization of the diffusion properties of different gadolinium-based MRI contrast agents after ultrasound induced blood–brain barrier permeabilization

#### Allegra Conti^1,2^, Rémi Magnin^1,3^, Matthieu Gerstenmayer^1^, François Lux^4^, Olivier Tillement^4^, Sébastien Mériaux^1^, Stefania Della Penna^2^, Gian Luca Romani^2^, Erik Dumont^3^, Benoit Larrat^1^

##### ^1^CEA/DSV/I2BM/NeuroSpin, Gif sur Yvette, France; ^2^Department of Neuroscience, G. D'Annunzio University, Chieti, Italy; ^3^Image Guided Therapy, Pessac, France; ^4^Université Lyon 1, Lyon, France

**Objectives**

The *in vivo* characterization of Gadolinium (Gd) based MRI Contrast Agents (MR-CA) diffusion within brain extracellular space is of great interest for the understanding of drug transport in brain parenchyma in the framework of new pharmaceutical developments for Central Nervous System diseases. We present here a new method to study the diffusion process of different MR-CAs after a transient and local Blood–brain Barrier (BBB) permeabilization induced by ultrasound. By estimating the Free Diffusion Coefficients from *in vitro* studies, and the Apparent Diffusion Coefficients from *in vivo* experiments, an evaluation of the tortuosity (λ) in the right striatum of 11 Sprague–Dawley rats has been performed.

**Methods**

**Fig. 89 (abstract O70).**
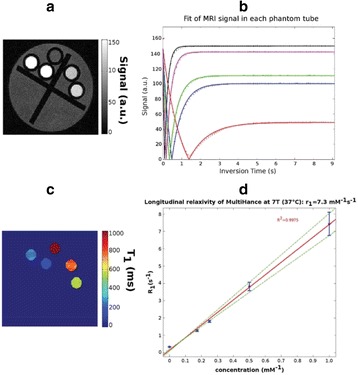
For each compound the r_1_ value was estimated by fitting the IR-FGE signals as a function of TI(S(TI) = |A ‐ B × exp(‐ TI/T_1_*)|, where T_1_ = T_1_* × [B/A ‐ 1]). Fig. **a** shows an IR-FGE MR image for one particular TI, and Fig. **b** shows the signals fits in each tube. From the T_1_, and Fig. **b** shows the signal fits in each tube. From the T_1_-maps (Fig. **c**) the longitudinal relaxivity was extracted by the linear fit: R_1_ = 1/T_1_ = 1/T_10_ + r_1_ × [CA]

Four Gd-chelates with different hydrodynamic diameters (d_H_) were tested: three commercially available MR-CA (MultiHance, Gadovist and Dotarem) and a new class of Gd-based nanoparticles (AGuIX, Nano-H). These latest compounds are composed of a core of polysiloxane, grafted with two or three Gadolinium chelates. They are sufficiently small (d_H_ < 5 nm) to escape hepatic clearance. Diffusion Light Scattering (DLS) measurements were performed to estimate the hydrodynamic diameter of all compounds (Table 5). The MRI acquisitions were performed with a 7T/90 mm Pharmascan scanner (Bruker). The contrasting power of each CA is characterized by its longitudinal relaxivity, r_1_. To evaluate the CAs’ longitudinal relaxivities at 7T and 37°C, bundles of tubes containing different CA-concentrations in 0.3% w/w agar gel were prepared. The T_1_ values of these tubes were measured using an IR-FGE sequence, by fitting the signals as a function of TI (S(TI) = | A-Bexp(−TI/T_1_*) |, where T_1_= T_1_*x[B/A–1]). Figure 89(a) shows an IR-FGE MR image for one particular TI, and Fig. 89b shows the signal fits in each tube. From the T_1_-maps (Fig. 89c) the longitudinal relaxivity was extracted by the linear fit: R_1_ = 1 / T_1_ = 1 / T_10_ + r_1_ x [CA], where T_10_ is the T_1_ of the media without Gd. Relaxivity values r_1_ are summarized in Table 5 for all compounds. The Free Diffusion Coefficients (D_Free_) of these compounds were then estimated by injecting 10 μL of a 5 mM solution in a tube filled with 0.3% w/w agar gel. The diffusion was followed for 1 hour by acquiring five T_1_-maps. The tubes were kept at 37°C during the acquisition. A T_10_-map acquired before the injection was used as a reference.

MR-CA concentration maps were then calculated from T_1_ maps using the previous equation. On each CA map, the following 2D Gaussian function (3) was fitted:

3 $$ \mathrm{C}\mathrm{A}\left(\mathrm{x},\mathrm{y}\right)=\mathrm{A}\ast \exp \left(-\mathrm{a}{\left(\mathrm{x}-{\mathrm{x}}_0\right)}^2-\mathrm{b}\left(\mathrm{x}-{\mathrm{x}}_0\right)\left(\mathrm{y}-{\mathrm{y}}_0\right)-\mathrm{c}{\left(\mathrm{y}-{\mathrm{y}}_0\right)}^2\right) $$

where A is the Gaussian amplitude and (x_0_,y_0_) are the coordinates of its center along the absolute axes (x,y). *a*, *b* and *c* are functions of the Gaussian spreads (σ_x_ and σ_y_) along its main axes (X and Y) and of the angle θ between these axes and (x,y).

By taking σ^2^_x_ and σ^2^_y_ as the molecules mean square displacements along X and Y, the diffusion coefficients along these axes, D_x,vitro_ and D_y,vitro_, are given by D_x,y,vitro_ = σ^2^_x,y_/2t, where t is the time after injection, i.e. the diffusion time. The Free Diffusion Coefficient was then calculated as the mean value of D_x,vitro_ and D_y,vitro_. Hydrodynamic diameter of the CA was deduced from D_free_ using the Stokes-Einstein formula (4):

4 $$ {\mathrm{D}}_{\mathrm{Free}}=\mathrm{k}\mathrm{T}/\left(3{\uppi \upeta \mathrm{d}}_{\mathrm{H}}\right) $$

where k is the Boltzmann constant, T is the temperature in Kelvinand η is the viscosity of the agar gel (6.92 x 10^−4^ Pa.s). Focused ultrasound induced BBB permeabilization was then performed in the right striatum of 11 Sprague–Dawley rats (120 g, Janvier, France). To do so, we used our previously developed motorized MR guided transcranial FUS system (Image Guided Therapy, France) [1]. It enables to position the ultrasound beam in the rat brain at high precision. A 1.5MHz, 8 channel MR compatible concave transducer was calibrated and mounted on the system for this study (Imasonic, France). It was coupled to the shaved rat head with echographic gel. After reference anatomy, T_1_ weighted and T_1_ mapping scans, the BBB opening protocol was performed as follows: sonovue microbubble intravenous injection (Bracco, Italy), pulsed ultrasound were shot at 0.5MPa (3ms/100ms for 60s). Then, 200μL of 5mM MR-CA were injected intravenously. The Gd chelates diffusion starting from the BBB disruption site was followed by repeatedly acquiring T_1_-maps for about 1 hour, as for the *in vitro* measurements. Animals were kept under general anesthesia during the whole procedure (1.5% isoflurane).

The same Gaussian fitting procedure was applied and the Apparent Diffusion Coefficients (ADC) of all compounds in the striatum were estimated as the average ADC = (D_x,vivo +_ D_y,vivo_)/2.

**Results**

**Fig. 90 (abstract O70).**
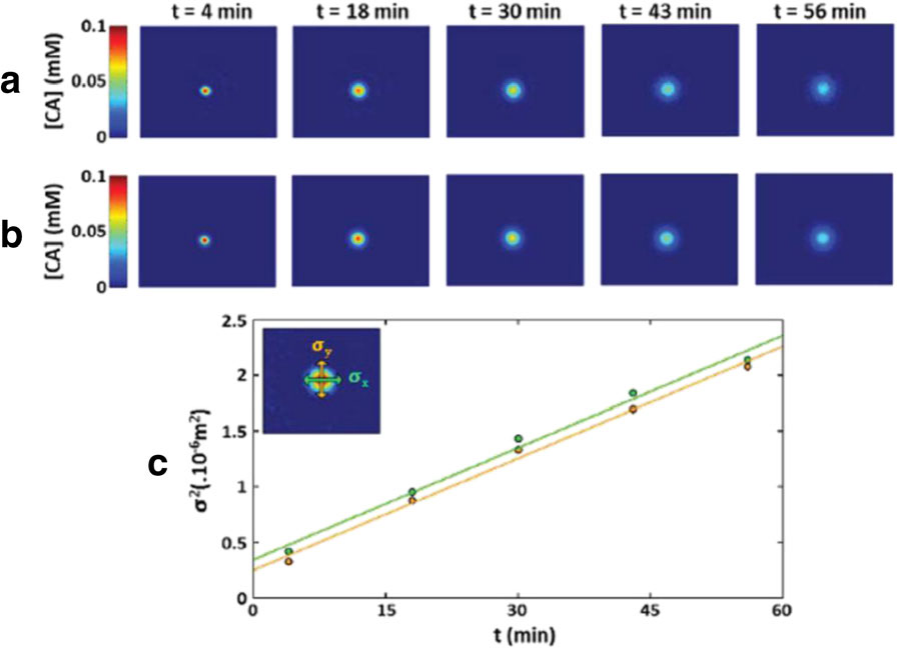
*In vitro* diffussion of MultiHance: **a** concentration maps and their repective 2D Gaussian fits (**b**) acquired during 1 hour CA injection in 0.3% w/w agar gel. Fig. **c** shows the squared Gaussian spreads as a function of time and their fits (*σ* ^2^_x,y_ = 2*t* × D_x,y vitro_)

**Fig. 91 (abstract O70).**
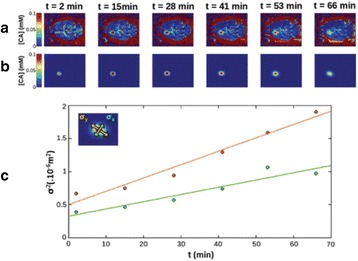
In vitro diffusion of Dotarem in the frontal hemisphere of a rat brain after ultrasound-induced BBB permeabilization (**a**) and the corresponding 2D Gaussian fits of CA-sports (**b**). Fig. C shows the linear fits of the squared Gaussian spreads as a function of time after CA injection

Figure 89 presents the steps to measure CA longitudinal relaxivities. Figure 90a shows examples of *in vitro* CA-maps obtained for MultiHance. The fitted 2D-Gaussian functions are presented in Fig. 90b, whereas in Fig. 90c the linear fit on their spreads is plotted. Figure 91 shows an example of *in vivo* dataset acquired by injecting Dotarem in one rat after focal BBB disruption. In Fig. 91a the original CA maps acquired within 66 minutes after the CA injection are pictured, and their respective Gaussian fits are shown in Fig. 91b. The linear fit over these Gaussian spreads is given in Fig. 91c. As can be noticed in Table 5, both D_Free_ and ADC are decreasing with increasing hydrodynamic diameters (Dotarem > Gadovist > MultiHance > AGuIX). Furthermore, quantitative values of hydrodynamic diameters deduced from D_free_ measurements are really consistent with DLS measurements. The ADC values have been used to estimate tissue tortuosity λ=(D_Free_/ADC)^0.5^ (Table 5), showing a very good agreement with the tortuosities evaluated with more standard techniques [2].

**Conclusions**

The agreement between the values of λ found after the blood–brain barrier permeabilization and the known values typical of healty brain tissue confirms the validity of this method to estimate the ADC values in the tortuous regime, but also that the diffusion properties of the tissue are not altered by the ultrasound induced BBB permeabilization protocol unlike by direct intracerebral injection [3]. This should be taken into account for CNS drug development since pharmacodynamics might be modified by direct injection.

**References**

[1] Magnin *et al., ISTU conference proceeding* 2014

[2] Nicholson *et al.*,*Trends Neurosci*. 1998, 21: 207–215

[3] Marty *et al.*, *CMMI* 2013, 8: 12–9

**Table 5 (abstract 70).** Extracted parameters for each compound (Dotarem, Gadovist, MultiHance and AGuIX). Notably, the apparent d_H_ estimated from the Stokes-Einstein equation is in agreement with DLS measurements, and both D_Free_ and ADC values decrease when molecular size increases. The tortuosities are consistent for all compounds and in agreement with literature

	Number of Rats	r_1_ at 7T (s^−1^·mM ^−1^)	D_Free_ (10^−11^ m^2^·s^−1^)	ADC (10^−11^ m^2^·s^−1^)	Stokes Einstein d_H_ (nm)	DLS d_H_ (nm)	λ
Dotarem	3	4.7	45	16	1.5	1.4	1.7
Gadovist	3	5.5	39	15	1.7	1.8	1.6
MultiHance	3	7.3	28	11	2.3	2.3	1.6
AGuIX	2	8.0	11	6	5.8	3.5	1.5

### O71 Closed-loop control of targeted drug delivery across the blood–brain barrier

#### Tao Sun^1,2^, Chanikarn Power^1^, Yong-Zhi Zhang^1^, Jonathan Sutton^1^, Eric Miller^2^, Nathan McDannold^1^

##### ^1^Department of Radiology, Brigham and Women’s Hospital, Harvard Medical School, Boston, Massachusetts, USA; ^2^Department of Electrical and Computer Engineering, Tufts University, Medford, Massachusetts, USA

**Objectives**

Microbubble-mediated focused ultrasound (FUS) can induce localized and reversible blood–brain barrier disruption (BBBD), aiding in targeted drug delivery to the brain. Acoustic cavitation is well-accepted as the primary mechanism in opening the BBB. In addition to its therapeutic potential, inducement of inertial cavitation by FUS can result in permanent damage to the brain tissue. Previous studies have shown the utility of cavitation detection for monitoring all bubble activities during treatment; however translation of FUS induced BBBD requires the development of a closed-loop, real-time control system that can tailor the opening while simultaneously keeping the brain damage-free. Here, we propose an acoustic emissions-based controlling paradigm that can not only modulate the BBBD outcomes based on the feedback from stable cavitation responses (harmonic emission, HE), but also suppress the likelihood of brain damage by monitoring the inertial cavitation components (broadband emission, BE).

**Methods**

This controlling system has been designed and tested in a preclinical dual-transducer setup. Two FUS single-element transducers were driven at different frequencies (F = 274.3 KHz, F = 30 Hz), and oriented at 120° to create a sub-centimeter focal depth-of-field. Cavitation activities were monitored using a passive detector (central frequency: 650 kHz).

Injections of Optison^TM^ into the tail vein in rats (*n* = 43) were performed with a computer-controlled injector that constantly rotated the syringe to keep the microbubbles mixed. HE and BE were monitored in real time and used as the basis for control of the next FUS pulse. The impact of multiple pulse parameters, including acoustic pressure, pulse repetition frequency (PRF), and total duration, was studied. To minimize the potential damage caused by inertial cavitation, the exposure level was reduced if BE was detected and terminated in the event it crossed a set threshold. The performance of the control system was assessed by BBBD in rats *in vivo*. Trypan Blue dye was injected as our model drug for visualizing and quantifying BBBD by fluorescent imaging 1-h post FUS treatment.

**Results**

A pilot study investigated the microbubble-dose effects on the linearity of the HE-pressure dependence and on the inertial cavitation threshold (ICT). The linearity of the HE was confirmed, and the ICT was found to decrease as microbubble dose (100, 200, and 400 μl/kg) was augmented. Assessment of the controller performance demonstrated that: 1) Control of HE is possible while keeping BE-free; 2) The use of microbubble infusion after an initial bolus allows the proportional controller to rapidly converge and prevents the decline of emissions, thus reducing the likelihood of BE due to the limited number of cavitation nuclei at the later stage of sonication; 3) A higher PRF (1 vs. 4 Hz) significantly enhanced the percentage of received emissions within the preset goal (Good Burst Rate, GBR), both in bolus injection mode (2-fold, *P*<0.05) and in infusion injection mode (7-fold, *P*<0.001); 4) Using an infusion and a PRF=4 Hz, the GBR for HE of 85.6% ±4.0% during 120-pulse sonications was achieved. Statistical comparison of GBR between the 60-, 90- and 120-pulse sonication showed no significant difference (*P*>0.05), suggesting the robustness of the controller. Total HE was exponentially correlated with the radiant efficiency of epi-fluorescence emitted from the Trypan Blue dye (*R* ^2^ = 0.78).

**Conclusions**

We have designed a real-time, closed-loop FUS controller based on passive cavitation detection for microbubble-mediated ultrasound therapy. The controller performance has been optimized through microbubble infusion and changing PRF. Controlled BBBD in rat *in vivo* was achieved based on the guidance of the present controller.

### O72 Two-dimensional array synthesis using raster-scanned single hydrophone in application to acoustic holography and ultrasound imaging

#### Oleg Sapozhnikov^1,2^, Sergey Tsysar^1^, Petr V. Yuldashev^1^, Vera Khokhlova^1,2^, Victor Svet^3^, Wayne Kreider^2^

##### ^1^Physics Faculty, Moscow State University, Moscow, Russian Federation; ^2^Center for Industrial and Medical Ultrasound, Applied Physics Laboratory, University of Washington, Seattle, WA, United States; ^3^N.N. Andreyev Acoustics Institute, Moscow, Russian Federation

**Objectives**

Two-dimensional (2D) receiving arrays with ~10^4^ total number of elements would serve as a powerful tool for ultrasound imaging, especially in the situations when the region of interest is imaged through inhomogeneous layers like skull or ribs. While 2D arrays with such a large number of elements are not yet practically available, their imaging capabilities can be studied by replacing them with synthetic 2D receiving arrays that are made using a single hydrophone that is raster scanned by a computer-controlled positioner. The goal of this study is to illustrate such an approach experimentally by using a synthetic 2D array in two applications: acoustic holography of therapeutic ultrasound sources and ultrasound imaging through a skull phantom.

**Methods**

To create a synthetic array, a miniature ultrasound probe was moved point-to-point using a computer-controlled positioning system. Measurements were made in degassed water using several experimental arrangements, in which the ultrasound probes were either capsule or needle hydrophones with sensitive diameters of 0.15, 0.2, or 0.5 mm. The scans were executed using positioning systems with stepper motors and linear slides that provided a resolution of several microns per step. In each scan, the hydrophone was sequentially located at the nodes of a square grid with a pitch less than half the wavelength. Typical size of the scanning region was 100×100 points, which corresponded to the number of elements of the corresponding virtual synthetic array. At each hydrophone position, an ultrasound source emitted a tone burst which was received by the hydrophone. Then, a hydrophone was moved to a new location and the operation of emission-reception was repeated. The pressure waveform at each location was recorded using a digital oscilloscope and transferred to a computer.

**Results**

**Fig. 92 (abstract O72).**
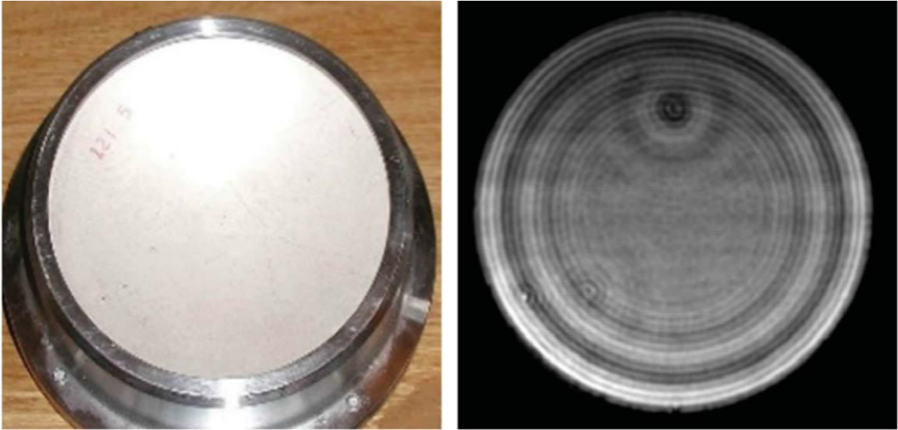
Piezoelectric transducer (*left*) and distribution of the particle velocity magnitude on the transducer surface while operating in the transient regime (*right*)

Synthetic arrays were used for two applications: The first was acoustic holography, which was shown in our previous work to be a powerful technique for characterizing ultrasound sources and the fields they radiate [see 1,2]. Beyond the CW version of holography that is appropriate for most regimes of ultrasound therapy, transient holography is directly relevant to imaging applications and therapies like histotripsy [3]. Here, a transient hologram was detected by a synthetic array (150×150 size, 0.7 mm pitch) in order to characterize a piezoelectric HIFU source (10 cm diameter, 10 cm focal length) excited by a 7-cycle tone burst at a frequency of 1 MHz. The reconstructed vibration velocity magnitude is shown in Fig. 92.

**Fig. 93 (abstract O72).**
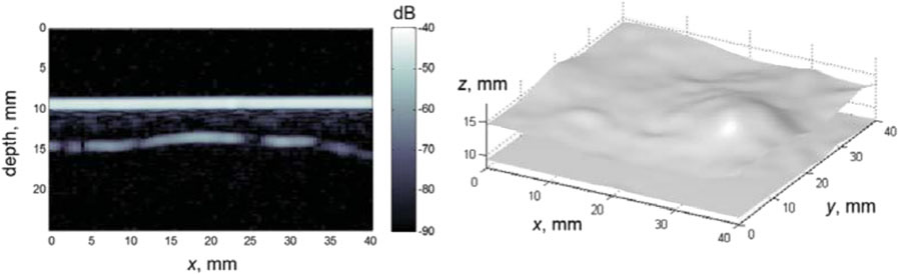
Skull phantom thickness pulse-echo measurements. *Left*: typical B-mode image in a transversal plane. *Right*: phantom back-side profile reconstructed from the pulse-echo measurements

**Fig. 94 (abstract O72).**
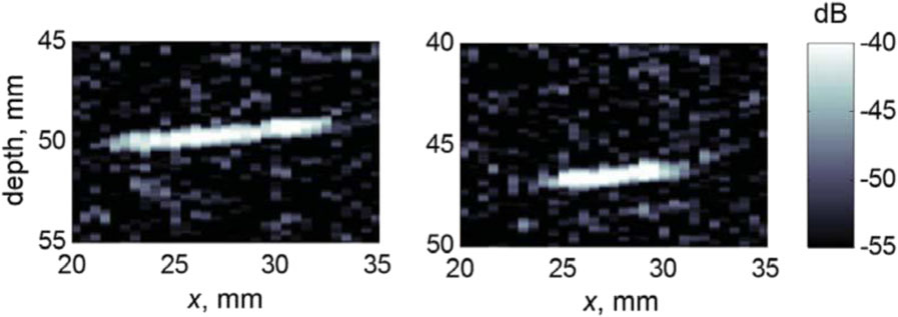
B-mode image of a 2-mm diameter spherical scatterer placed behind the skull phantom. *Left*: image constructed without account for the inhomogeneous layer. *Right*: image reconstructed with account for the layer

The second application of synthetic arrays was ultrasound pulse-echo imaging through an inhomogeneous layer mimicking a human skull. Transcranial ultrasound imaging remains problematic due to severe aberrations caused by the skull. Wide-aperture 2D arrays can help to achieve usable imaging resolution by compensating for aberration effects. In the experiments, a skull phantom was made from epoxy resin mixed with aluminum oxide powder. The phantom had the following parameters: density 1.4 g/cm^3^, longitudinal velocity 2.6 mm/μs, shear velocity 1.3 mm/μs, and absorption coefficient 4 dB/cm at 1 MHz, 7 dB/cm at 2 MHz. The phantom thickness was made nonuniform with one side being flat and the other side having profile variations similar to human skull. To simulate the “flash-mode” imaging regime, the skull phantom was insonified from the flat side by a short 2-MHz tone burst emitted by a broadband wide-aperture (several cm diameter) flat source. A needle hydrophone was raster scanned in a plane region proximal to the skull phantom. The corresponding synthetic array was of 100×100-element size and 0.5 mm pitch. Several mm-sized scatterers were placed in water at 3–4 cm distance from the other side of the phantom. The imaging consisted of two steps. In the first step, the skull phantom thickness was mapped using echo arrival time differences between the front and back surfaces (Fig. 93). Then the 3D image was built based on a delay-and-sum algorithm. The image was built both without and with account for the presence of the inhomogeneous skull phantom. Typical image improvement can be seen in Fig. 94: the lateral resolution was significantly improved when the aberrations were accounted for in the procedure.

**Conclusions**

Synthesizing a 2D array with a large number of elements can be effectively done even with a single hydrophone. To mimic the array, a hydrophone placed in the desired array elements’ locations by a computer-controlled positioner can be used. Array synthesis is possible and effective if the acoustic field under study can be generated repeatably with high accuracy. Capturing acoustic field measurements in 2D provides detailed information about 3D fields in both CW and transient regimes. This information has practical utility in both therapeutic and imaging applications. The work was supported by the Russian Science Foundation grant no. 14-15-00665 and NIH R21EB016118.

**References**

[1] Sapozhnikov *et al*. Acoust. Phys. 49(3), 354–360 (2003)

[2] J. Acoust. Soc. Am. 138 (3), 1515–1532 (2015)

[3] Sapozhnikov *et al*. Acoust. Phys. 52(3), 324–330 (2006)

### O73 Ultra-high speed imaging and modelling of shock wave interactions with cells

#### Dongli Li, Antonio Pellegrino, Nik Petrinic, Clive Siviour, Antoine Jerusalem, Robin Cleveland

##### Department of Engineering Science, University of Oxford, Oxford, United Kingdom

**Objectives**

Visualise and model cell deformation under the action of shock waves in order to optimise therapeutic efficacy while minimising side-effects during treatment.

**Methods**

Shock waves from a clinical shock wave source (Minilith, STORZ) were applied to a tissue-mimicking phantom with embedded cells. The pressure profile of the shock wave was measured inside the phantom using a PVDF needle hydrophone and the induced strain rate was estimated to be 10^5^-10^6^ s^−1^. The deformation of individual cells were tracked using an ultra-high speed camera with frame rate up to 20 Mfps. The cell response was analysed by its area and perimeter change over time. A Finite Element (FE) model was developed to compare to the experimental findings with the same setup using a combined non-linear fluid and hyper-viscoelastic framework.

**Results**

Under the compression phase of shock waves, cells showed 3-5% of area decrease and 1-2% of perimeter reduction, whereas under the tension phase, cells showed 15-20% of area increase and 6-8% of perimeter rise. Simulation results matched with the experimental findings by proposing a new constitutive model differentiating the compressive behaviour from its tensile counterpart.

**Conclusions**

The results of this study suggest that at high strain rate the cell appears as much stiffer in compression than in tension because of the intrinsic deformation mechanisms. The accurately characterised cell properties can thus help to predict cell response in order to optimise the therapeutic efficacy of shock wave applications such as lithotripsy, orthotripsy and cancer treatment.

**Fig. 95 (abstract O73).**
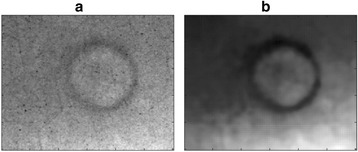
Imaging processing for the ultra-high speed images

**Fig. 96 (abstract O73).**
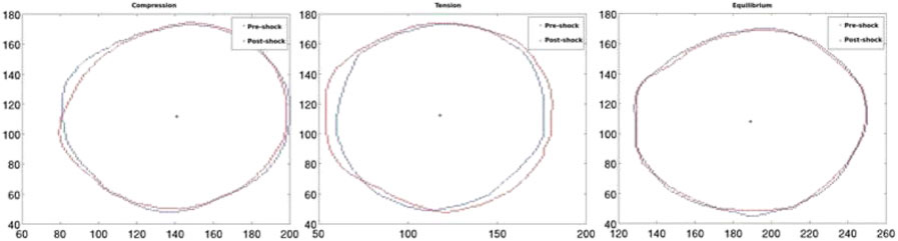
Comparison of cell contours during shock wave interactions

### O74 Comparison of derating methods for nonlinear ultrasound fields of diagnostic-type transducers

#### Peter V. Yuldashev^1^, Maria Karzova^1^, Bryan W. Cunitz^2^, Barbrina Dunmire^2^, Wayne Kreider^2^, Oleg Sapozhnikov^1,2^, Michael R. Bailey^2^, Vera Khokhlova^1,2^

##### ^1^Physics Faculty, Moscow State University, Moscow, Russian Federation; ^2^CIMU, Applied Physics Laboratory, University of Washington, Seattle, WA, United States

**Objectives**

There are therapeutic and diagnostic uses of imaging probes, which benefit from exceeding the Mechanical Index limits of diagnostic ultrasound and support that these benefits occur without negative bioeffects. Without imbedded software restrictions, the *in situ* pressure levels of these devices can exceed the typical diagnostic limits on Mechanical Index and spatial peak pulse average intensity (I_SPPA_). When calibrating imaging probes at these levels in water, nonlinear propagation effects are present, which complicates the derating process for estimating *in situ* fields. Different derating approaches have been proposed to predict pressures in tissue from measurements in water. One conventional derating method is to scale the focal pressures and another method is to scale the source amplitude to compensate for linear losses on the way to focus. This second method is described as nonlinear derating and has been shown to provide accurate results for strongly focused therapeutic transducers. However, applicability of these derating methods to diagnostic probes operating at therapeutic intensities is still in question. Here, the derating methods were tested for a diagnostic probe used in kidney stone propulsion technology.

**Methods**

**Fig. 97 (abstract O74).**
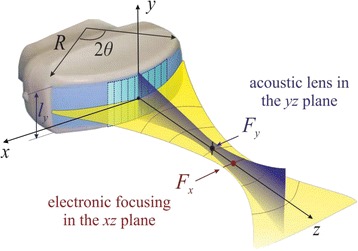
Geometry of focused ultrasound beam produced by C5-2 array probe with 64 active elements

A standard diagnostic ultrasound curved array probe operating at 2.3 MHz (C5-2, Philips Ultrasound, Andover, MA, USA) was considered. The array comprises 128 elements; however, the results presented hereafter were obtained by considering 64 central elements of the array to be active (Fig. 97). In the azimuthal plane the focus can be steered electronically, while a cylindrical acoustic lens focuses the field at a fixed depth in the elevation plane.

A combined measurement and modelling approach was used to establish an equivalent source boundary condition for nonlinear simulations of the array field in water based on the 3D Westervelt equation [1]. Simulations were performed for propagation entirely in water and in the presence of a tissue mimicking phantom placed at a distance of 1 cm in front of the probe surface. The acoustic properties of the phantom were set the same as in water, except for the frequency dependent absorption of α_0_ = 0.5 dB/cm/MHz with power exponent *n* = 1.2 and corresponding dispersion that were set according to the properties of the phantom.

Two derating methods were tested to estimate the *in situ* (*z* = 50 mm) pressure field in tissue from the waveforms simulated in water. Derated waveforms were then compared with direct simulation results in tissue. In the conventional derating method, the pressure field calculated in water at the focus was multiplied by the absorption exponent accounting for the propagation distance of 40 mm in tissue to the focus. In the nonlinear derating method, the pressure amplitude at the focus in tissue was assumed to be the same as in water for the lower source voltage scaled with the same absorption exponent value.

**Results**

**Fig. 98 (abstract O74).**
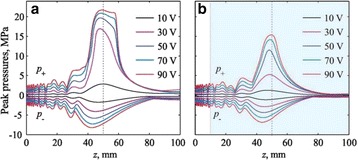
Axial distributions of the peak positive and peak negative pressures for **a** free field propagation in water and **b** propagation in the presence of a tissue phantom (shown in *blue*) for outputs of 10, 30, 50, 70 and 90 V. The water/phantom interface is positioned 1 cm from the array centre

Axial distributions of the peak positive and peak negative pressures are shown in Fig. 98 for several output voltages in the free field in water (a) and in the presence of the tissue phantom (b). The focal lobe of the probe (20 mm long) is relatively large in comparison with the focal length of 50 mm because the transducer has a relatively low linear focusing gain (9.3). Therefore, at high power outputs nonlinear propagation effects accumulate over the long propagation distance and are not localized near the focus as is the case for strongly focused therapeutic sources.

**Fig. 99 (abstract O74).**
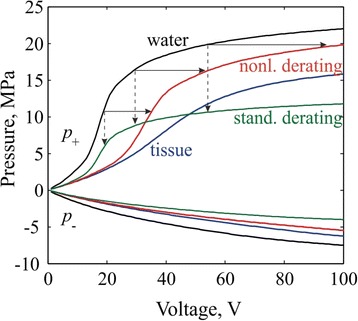
Peak positive and negative pressures at the focus (*z* = 50 mm) versus transducer output voltage. Propagation is simulated in water (*black curve*) and in a tissue phantom (*blue curve*). Horizontal and vertical arrows illustrate nonlinear derating by scaling the source amplitude (*red curve*) and conventional derating by scaling the focal pressures (*green curve*)

Peak positive and peak negative pressures at the focus, z = 50 mm, as functions of source voltage are shown in Fig. 99. The nonlinear saturation of the peak positive pressure is clearly seen for propagation in water (black curve) and in tissue (blue curve), though at higher voltage levels. The conventional derating process of scaling focal pressures is illustrated by the green curve in Fig. 99. As denoted by the vertical dashed arrows, this method overestimates peak positive pressure at moderate voltages (up to 50 V) and underestimates it at higher voltages. Nonlinear derating is illustrated by the red curve. As shown by the horizontal arrows, peak positive pressures are significantly overestimated (by up to 50%) for source voltages higher than 20 V. For lower voltages, the nonlinear derating matches results in tissue within 10% of accuracy. Peak negative pressure magnitudes estimated by derating methods are smaller than those obtained in direct numerical simulations. Peak negative pressures predicted by the conventional derating method can be 50% smaller than in simulations, while the discrepancy remains less than 20% for the nonlinear derating method.

**Fig. 100 (abstract O74).**
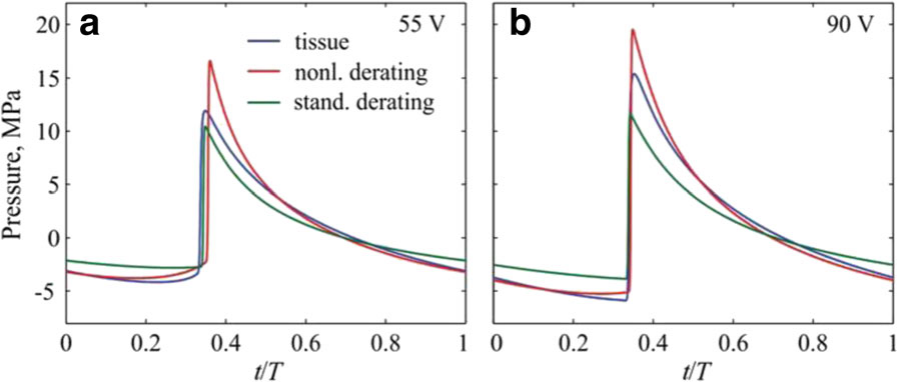
Focal waveforms (*z* = 50 mm) obtained by direct modelling of propagation in the tissue phantom (*blue curve*) as well as by derating from free field water simulations (*red curve* for nonlinear derating, green curve for conventional derating). Results are shown for output levels corresponding to **a** 55 V and **b** 90 V

Focal waveforms obtained in simulations in tissue (blue curve) and using the derating methods (red and green curves) are compared in Fig. 100 for 55 V (a) and 90 V (b) outputs. At 55 V the nonlinear derating method predicts 40% higher peak positive pressure than simulations, while peak negative pressures are in closer agreement. The waveform resulting from conventional derating is fortuitously close to the simulated waveform. At 90 V all waveforms are significantly different; the peak positive pressure obtained in simulations is approximately in the middle between the results of the two derating processes.

**Conclusions**

Nonlinear acoustic fields generated by a diagnostic ultrasound probe are simulated in water and in a tissue phantom using the 3D Westervelt equation. Two derating approaches were applied to estimate the pressure field in tissue using the results obtained in water. It was shown that the conventional derating method can either overestimate (up to 50%) or underestimate (up to 25%) peak positive pressure depending on the source voltage, while it underestimates peak negative pressures by up to 50%. The nonlinear derating method provides accurate results at low intensities (here up to 20 V); however, it overestimates peak positive pressures by up to 50% at higher intensity levels. These simple derating procedures therefore cannot substitute direct numerical modelling to provide reasonable accuracy for nonlinear in situ pressures for diagnostic probes. The study was supported by the grants RSF 14-12-00974, NIH EB007643 and DK043881.

**Reference**

[1] Karzova *et al*., AIP Conf. Proc. (1685) 040002–1, 2015

### O75 Linear and nonlinear radial oscillations of an acoustic bubble submitted to a dual-frequency excitation: analytical and experimental studies

#### Claude Inserra^1^, Matthieu Guedra^1^, Cyril Mauger^2^, Bruno Gilles^1^

##### ^1^LabTAU Inserm U1032, Lyon, France; ^2^INSA de Lyon, Villeurbanne, France

**Objectives**

It is commonly admitted that the use of multifrequency acoustic excitations, and particularly a dual-frequency signal, can stimulate the cavitation activity resulting in a decrease of the inertial cavitation threshold for instance. However, past works were usually dedicated to the experimental characterization of the influence of bifrequency excitations on the cavitation cloud activity, and only few researches focused on the nonlinear dynamics of a single bubble in a dual-frequency acoustic field. In this study we describe analytically the aperiodic oscillations of a single dual-frequency driven bubble by assuming weakly nonlinear oscillations. These results are compared to experimental studies on a single laser-nucleated bubble driven by bifrequency excitations with varying difference frequency.

**Methods**

The analytical study relies on the asymptotic expansion of the Rayleigh-Plesset equation in weakly nonlinear oscillations using the Bogolyubov-Krylov method. Approximate analytical solutions are obtained for bubbles excited by a dual-frequency signal for which both high-frequency components are close to the primary resonance frequency of the bubble. For studying experimentally the bubble radial oscillations, a laser-nucleated bubble is generated and trapped in a bifrequency acoustic pressure field centred at 33kHz, within a cubic water cavity. For typical bubble radii ranging between 50 and 85μm, radial oscillations are visualized with a high-speed camera (frame rate 180 kpps), allowing to probe the temporal evolution of the bubble radius as well as the maximal reached bubble radius.

**Results**

Theoretical results reveal that the maximal reached bubble radius can be higher for bifrequency excitation than for monofrequency excitation. The possible enhancement of the bubble radial dynamics is due to the multiple energy transfer and pumping from each frequency component of the dual-frequency signal to other spectral components that are favourably energetic. Results also highlight the existency of a specific difference frequency value above which the bifrequency excitation is no longer efficient. Linear and weakly nonlinear bubble radius evolutions are thus studied experimentally in the aim of demonstrating such enhancement. While linear radial oscillations of a bubble in a dual-frequency excitation well reveal the beating phenomenon, increasing the excitation amplitude leads to the regime of nonlinear oscillations where non-spherical radial oscillations are rapidly observed. The breathing of such non-spherical oscillations triggered on the difference frequency component is discussed.

**Conclusions**

The oscillations of a spherical bubble driven by a dual-frequency excitation have been analysed both analytically and experimentally. An asymptotic expansion of the bubble radial dynamics revealed that the dual-frequency excitation may lead to larger amplitudes of the bubble oscillations for sufficiently high driving amplitudes and for specific values of the frequency difference. This enhancement is related to the generation of high-order frequency harmonics by nonlinear mixing. These results allow describing the mechanisms underlying frequency energy transfer in multi-frequency excitations.

### O76 Patient specific simulation for liver tumor ablation

#### Maxim Solovchuk^1^, Tony W.H Sheu^2^, Marc Thiriet^3^

##### ^1^Institutes of Biomedical Engineering and Nanomedicine, National Health Research Institutes, Zhunan, Taiwan; ^2^National Taiwan University, Taipei, Taiwan; ^3^Sorbonne Universities, UPMC Univ Paris 06, Paris, France

**Objectives**

High intensity focused ultrasound is a rapidly developing technology for the ablation of tumours. Liver cancer is one of the most common malignancies worldwide. Since liver has a large number of blood vessels, blood flow cooling can reduce the necrosed volume and may cause regeneration of the tumour to occur. All cancer cells should be ablated without damaging of the critical tissues. Feasibility of ablating the tumour close to blood vessel wall is studied. Importance of nonlinear propagation effects, blood flow cooling and acoustic streaming effects is investigated during focused ultrasound therapy. This work is a step towards the development of surgical planning platform for a non-invasive HIFU tumour ablative therapy in a real liver geometry.

**Methods**

The developed computational model is based on the nonlinear Westervelt equation with relaxation effects taken into account, bioheat equations for the perfused tissue and blood flow domains. The nonlinear Navier–Stokes equations are employed to describe the flow in the large blood vessels. The effect of acoustic streaming is also taken into account. Three dimensional meshes for the hepatic artery, hepatic vein, vena cava and liver were reconstructed on the basis of the MRI image. The present numerical experiments are carried out in a patient specific liver model. The model has been validated by comparison with ex-vivo MRI temperature measurements.

**Results**

In this three-dimensional field-coupling study we illustrate how a computational model can be used to improve the treatment efficiency. It was also shown that relaxation effects can affect the temperature distribution. In large blood vessel both convective cooling and acoustic streaming can change the temperature considerably near blood vessel. Temperature elevation by HIFU in ex-vivo porcine muscle was also studied experimentally by MRI and numerically. We demonstrated that for peak temperatures below 85-90°*C* numerical simulation results are in excellent agreement with the experimental data in three dimensions.

If destruction of all tumour cells near the blood vessel boundary is necessary, a shorter sonication time with higher power deposition is suggested. The total simulated treatment time is only 30 seconds. Comparing with the treatment time of several hours we can see that computational fluid dynamics can sufficiently reduce the treatment time and improve the quality of treatment.

**Conclusions**

The proposed three dimensional physical model for HIFU study was conducted in an image-based liver geometry. It was shown that tumours near the blood vessel wall can be ablated without damaging blood vessel wall. These results can be further used to construct a surgical planning platform for a non-invasive HIFU tumour ablating therapy in real liver geometry from CT or MRI image and can lead in the future to a substantial improvement of the focused ultrasound ablation of liver tumour. The whole tumour ablation took only 30 seconds in the considered simulation case, which is very small comparing with the current treatment time of several hours. Through this study we are convinced that high ultrasound power and nonlinear propagation effects with appropriate treatment planning can sufficiently reduce the treatment time. The presented model can be used in planning tools for the thermal ablation of tumour in other organs and is also applicable to acoustic hemostasis treatment.

### O77 Quantitative assessment of nonlinear acoustic intensity in HIFU field by infrared thermometry: theoretical study

#### Yufeng Zhou

##### Nanyang Technological University, Singapore, Singapore

**Objectives**

Characterization of high-intensity focused ultrasound (HIFU) output is important for both efficacy and safety of clinical treatment. Acoustic intensity is one of the key parameters, and can be calculated from the measured pressure waveform. However, hydrophone scanning in the focal region is time consuming. Recently, an infrared camera has been used to measure the temperature elevation at the surface of an absorber, from which the relative distribution as well as the absolute intensity of the focused acoustic field can be determined. Its advantage is the rapid assessment of ultrasound beams, but only linear acoustics is considered by this approach. Derivation of nonlinear acoustic intensity with the significant generation of harmonics by infrared thermometry is investigated theoretically in this study.

**Methods**

Numerical simulation was carried out for wave propagation in the free field and thermal generation in an absorber. The pressure waveforms at the focus were simulated using KZK equation, and the corresponding spectra were calculated after fast Fourier transformation (FFT). A new model was established to describe the acoustic field in the absorber and the thermal conduction towards the interface, and then compared to the current two models (superposition and standing wave model), especially at high frequency and absorption. The temperature elevations on the surface of absorbers (either different materials or thickness) are calculated by the Heat Transfer equation using finite difference method, from which the acoustic intensity can be derived by changing the ultrasound exposure time.

**Results**

The amplitudes of harmonics are found to decay exponentially, which can be fitted by a simple equation with only three parameters, at different output power levels (up to 200 W). The difference of deposited acoustic energy in the absorber and heat conduction between the standing wave model and our model becomes significant at high frequency and attenuation. The acoustic intensities derived from the simulated temperature elevation at the interface at different conditions are found to be within 5% less than those calculated by KZK equation. There is a monotonic decrease of temperature elevation with the thickness of absorber, which results in similar derivation accuracy using our approach.

**Conclusions**

The proposed approach may provide an easy and rather accurate way of characterizing the HIFU field with significant nonlinear effects. Experimental work will be performed to further validate this approach.

### O78 Tissue properties database to support computational modeling of therapeutic ultrasound

#### Esra Neufeld^1^, Christian Baumgartner^1^, Davnah Payne^1^, Adamos Kyriakou^1^, Niels Kuster^1,2^

##### ^1^IT'IS Foundation for Research on Information Technologies in Society, Zurich, Switzerland; ^2^Swiss Federal Institute of Technology (ETHZ), Zurich, Switzerland

**Objectives**

Computational modelling of therapeutic ultrasound procedures supports device design, safety and efficacy analyses, mechanistic investigations, and (personalized) treatment planning. However, reliable modelling depends on the availability of good data about the acoustic and possibly thermal behaviours of relevant tissues and their variability, and comparison between different modelling efforts requires publicly available, standardized properties. The IT’IS database of tissue properties has been created to fulfil these specific requirements.

**Methods**

The IT’IS Foundation (www.itis.ethz.ch) has created a comprehensive, publicly available, and evolving database (http://www.itis.ethz.ch/virtual-population/tissue-properties/overview/) of tissue properties that includes dielectric properties and values of density, heat capacity, thermal conductivity, heat generation rate, transfer (perfusion) rate, low frequency electrical conductivity (including information about anisotropy in fibrous tissues), and viscosity for more than 100 biological tissues. The underlying values are based on an extensive review of the literature and have been carefully selected based on various criteria, including the origin of the data (e.g., species) and the quality of the measurement methodology (freshness of tissue, accuracy of method). In some cases, dispersion relationships (frequency dependence), temperature dependence, or anisotropy is also reported.

Since September 2015, the IT’IS database also includes acoustic properties, i.e., speed-of-sound, attenuation, and the non-linearity parameter (B/A). The non-linearity of the attenuation frequency-dependence was determined by fitting a power law to tissue-specific data, whenever these data were sufficient. Additionally, previously determined relationships for frequency-dependent attenuation have been geometrically sampled to provide input for the fitting of pooled data.

Quality assurance procedures have been implemented that automatically detect unexpectedly large changes between successive database versions and all modifications performed since the release of the first online database version are documented in a log file available online and tracked by a versioning system. Additionally, Digital Object Identifies (DOI) are issued for each database version to guarantee traceability.

**Results**

An online resource has been created, which provides information about recommended tissue properties and their variability (standard deviation, extreme values) for a large number of tissues. All values are available for online viewing and can be downloaded in various formats. Additional documents available for download are a) a summary of all general considerations, remarks, and known issues regarding the database; b) the rational for each tissue substitution; c) previous versions of each database; d) a log file documenting the changes between versions; e) the list of all publications used to generate the database; and f) tables including the specific references associated with each tissue and each property for which provide a value is provided. The suggested values are also available as part of the tissue properties database of the Sim4Life simulation platform (ZMT Zurich MedTech AG), which facilitates computational modeling of focused ultrasound and induced heating. Within Sim4Life, the database is automatically synchronized with the Virtual Population V1.x and V3.x anatomical models to maximize model usability and minimize human error when setting up simulations.

**Conclusions**

A publicly available online database encompassing acoustic and thermal tissue properties and their variability has been compiled and quality assurance measures implemented to support computational modelling of therapeutic ultrasound. This specific database has already served for a range of modelling projects in the context of therapeutic focused ultrasound. The previously assembled dielectric and thermal properties have been extensively used for dosimetric and thermal therapy modelling purposes, resulting in over 100 citations and enabling the replication and comparison of simulation results.

The IT’IS material parameter database is an evolving web resource. Accordingly, it relies on input from database users and on contributions via our online forum. This forum is designed for users to suggest additional references, to question the assumptions behind the current values, and to discuss possible improvements.

Additional data points are added as they become available in the literature or experimentally. Yet besides adding data to fill existing gaps and improve quality, we aim at including additional information that permits filtering according to specific criteria (e.g., considering only data measured in human tissue), adding properties (e.g., thermoregulatory), introducing distinctions between absorption and attenuation, and providing calculators (e.g., to relate CT bone density data to acoustic property maps.

**Fig. 101 (abstract O78).**
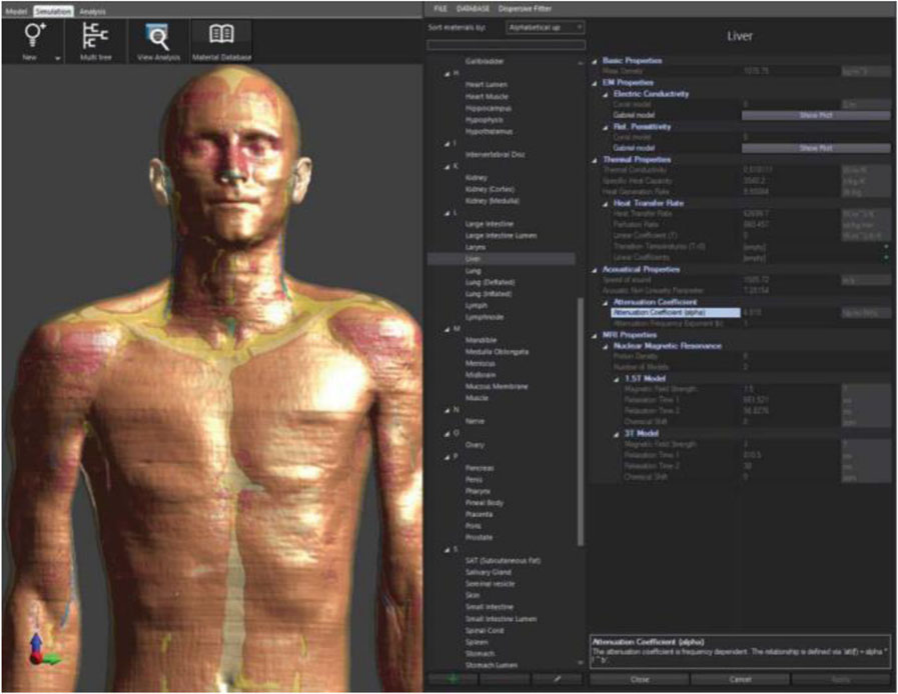
Automatic synchronization of the IT'IS database of tissue properties with the IT'IS Virtual Population Models in Sim4Life

### O79 Ex-vivo thiel embalmed porcine and human kidney model simulating blood perfusion in FUS therapy

#### Xu Xiao^1^, Helen McLeod^2^, Andreas Melzer^1^

##### ^1^Institute for medical science and technology, Dundee, United Kingdom; ^2^Ninewells Hospital, Dundee, United Kingdom

**Objectives**

The principle of focused ultrasound therapy (FUS) is to elevate the pathological tissue temperature rapidly therefore to ablate the target tissue. While absorbing the thermal energy from ultrasound beam, the target tissue also have thermal exchange with surround tissues by conduction and convection. The blood vessels can generate more effective convection on the proximity. If the target pathological tissue locate the proximity of blood vessel, the thermal convective effect from blood flow must be considered.

Thiel fluid is an advanced technology to store tissues, and it does not cause significant difference on thermodynamics properties. Therefore the Thiel kidney is the idea specimen for the perfusion and FUS therapy experiment.

Thiel swine kidneys are used to study the effect of blood flow on focused ultrasound (FUS) therapy. This article will demonstrate the preparation of a pair of perfused Thiel porcine kidney which is used to simulate the blood circulation. And the MRgFUS on perfused porcine and human kidneys is conducted to evaluate the effect of blood flow on FUS nearby tissues.

**Methods**

**Fig. 102 (abstract O79).**
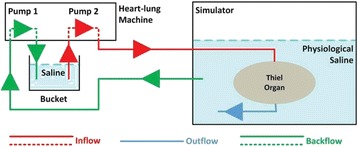
Diagram of the flow chart of the perfusion setup

The simulator consists of a pair of Thiel porcine kidneys or human kidneys, a waterproof plastic bag, and a heart lung machine (Maquet HL30, Germany). The Thiel kidneys are placed into the sealed waterproof plastic bag, and the renal artery is used as the inflow, while the renal vein as the outflow. A tube is connected to the plastic bag to extract the saline water back to the heart lung machine to complete the circulation. Cable ties are required to close the distal of the artery to guarantee the whole saline water is perfused into the kidney's blood circulation network (Fig. 102).

The simulator is placed under the image-guided FUS surgical environment. Multiple image technologies is used for the FUS therapy experiment. X-Ray (OEC9900, GE, USA), MR scan (1.5T, GE, USA) are used to image the perfusing model.

The ExAblate 2000 (Insighte, Haifa, Isreal) focused ultrasound is used to ablate the kidney perfusion model. The result will state the effect of blood flow on FUS therapy. The parameters for MRgFUS in this experiment are as follows: TE=100ms, TR=150ms, Flip Angle=60°, Slice Thickness=5mm. Acoustic Power=20W, Duration Time=20s. The target position were selected nearby main vessels, approximately 5mm away from the vessels. Each selected position were sonicated twice, one under the condition with blood flow, while the other without. The overall flow speed is controlled at 500 mL/min for porcine kidneys and 200 mL/min for human kidneys. The temperature rises are recorded.

**Results**

**Fig. 103 (abstract O79).**
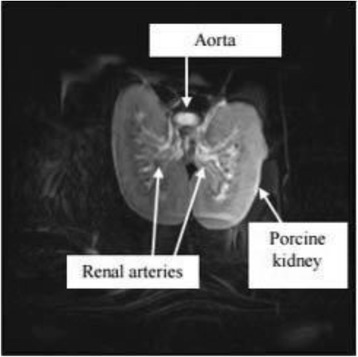
MR scan of perfused kidneys

**Fig. 104 (abstract O79).**
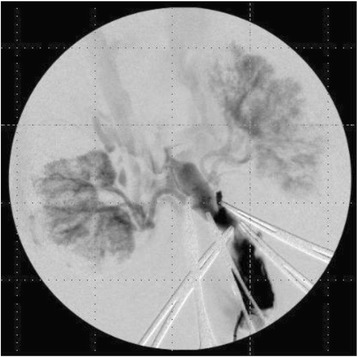
See text for description

The MR scanning protocol could provide clear image of the blood vessels inside the kidneys. Especially with blood flow, the vessels appear much brighter than surrounding tissues (Fig. 103). Under the X-ray scans, the perfusion process is monitored and recorded, clear blood flowing in to the kidneys and flowing away from the kidneys is clear (Fig. 104).

Three pairs of selected sonication showed reasonable results. A significant temperature drop was observed when a perfusion blood flow was available within the porcine or human kidneys. And there was a 2~4°C higher temperature rising in the no-flow condition.

**Conclusions**

From this early-stage experiment, it indicated that the blood flow have influence on FUS ultrasound therapy. The target tissue under FUS therapy has conductive thermal transfer to the surrounding, and the blood flow supplies extra thermal transfer, which has considerable contribution on the tissue cooling down. The experimental results indicate a clear temperature drops is observed when a perfusion blood flow is available within the kidneys, compared with the no-flow condition has a 2 ~ 4°C higher temperature rising. Therefore it is reasonable to consider the influence from blood flow when choose the thermal dose if the target tissue is nearby the vessels, especially the main vessels.

### O80 In vivo pre-clinical and clinical MRgFUS estimation of thermal diffusivity and perfusion

#### Christopher Dillon^1^, Viola Rieke^2^, Pejman Ghanouni^3^, Dennis L. Parker^1^, Allison Payne^1^

##### ^1^Radiology, University of Utah, Salt Lake City, UT, United States; ^2^Radiology and Biomedical Imaging, University of California San Francisco, San Francisco, CA, United States; ^3^Radiology, Stanford University, Stanford, CA, United States

**Objectives**

Thermal models for magnetic resonance-guided focused ultrasound (MRgFUS) therapies may be used to predict outcomes, improve monitoring, and inform real-time controllers that guide treatments. When accurate, these models have potential to identify which patients will or will not benefit from MRgFUS and to improve treatment safety, time-efficiency, and efficacy. However, accurate thermal models require accurate knowledge of properties including the tissue thermal diffusivity (*α*) and the Pennes perfusion parameter (*w*). This study presents the in vivo use of a new analytical method for estimating *α* and *w* from MRgFUS pre-clinical experiments in rabbit muscle and from clinical MRgFUS treatments of uterine fibroids.

**Methods**

The method utilizes an integral temperature solution for a Gaussian heating pattern with no axial conduction assuming constant, uniform tissue properties [1]. By replacing the perfusion term with its 3^rd^ order Taylor series approximation, instead of assuming it is zero [1–3], the integral can be evaluated to provide a closed-form analytical solution [4]. Using MR temperature data in the focal plane, a least-squares optimization of the analytical solution yields estimates of *α* and *w*. Experiments were performed on 3 New Zealand white rabbits with IACUC approval. Rabbits were positioned supine on the MRgFUS system and sonications were performed in the back muscle (5 heating locations, 28 sonications). Transducer power (P) was 8 W and heating time (HT) ranged from 21.7-23.6 s. MR temperatures (Siemens 3T Trio, 3D seg-EPI, TR/TE=35-44/11 ms, FA=15-30°, BW=738-744 Hz/pixel, voxel=1×1×3mm, FOV=128-192×80-132mm^2^, 8 slices, ETL=9, and t_acq_=3.6–5.4 s) were calculated by a 2D referenceless technique [5] using the proton resonance frequency (PRF) method.

To establish whether the estimation method works with clinical protocols, 7 sonications (InSightec Exablate 2000, P=14-24 W, HT=20 s) were performed in a quality assurance (QA) phantom using uterine fibroid MRgFUS protocols (GE Discovery, coronal single slice fast spoiled GRE, TR=25-26 ms, TE=12-13 ms, FA=30°, BW=44 Hz/pixel, matrix=256×128, FOV=280×280mm^2^, slice thickness=3-5 mm, and t_acq_ =3.2-3.3 s). Temperatures were calculated via a baseline reference reconstruction using the PRF method. In addition, retrospective analysis was performed on clinical fibroid MRgFUS data from 9 patients (n=12 sonications, P=32-138 W, HT=20 s) with imaging and reconstruction parameters described above.

Estimates of *α* and *w* were made using MRgFUS data from voxels within 6 mm of the beam axis and included all temperatures acquired during heating and the first temperature image acquired from cooling (t_fit_~ 24 s), i.e. the default acquisition scheme used by the clinical MRgFUS system. To assess the effect of additional cooling temperature data, 10–20 additional images were acquired when possible so that the fitting time could be extended to t_fit_~ 80 s.

**Results**

**Fig. 105 (abstract O80).**
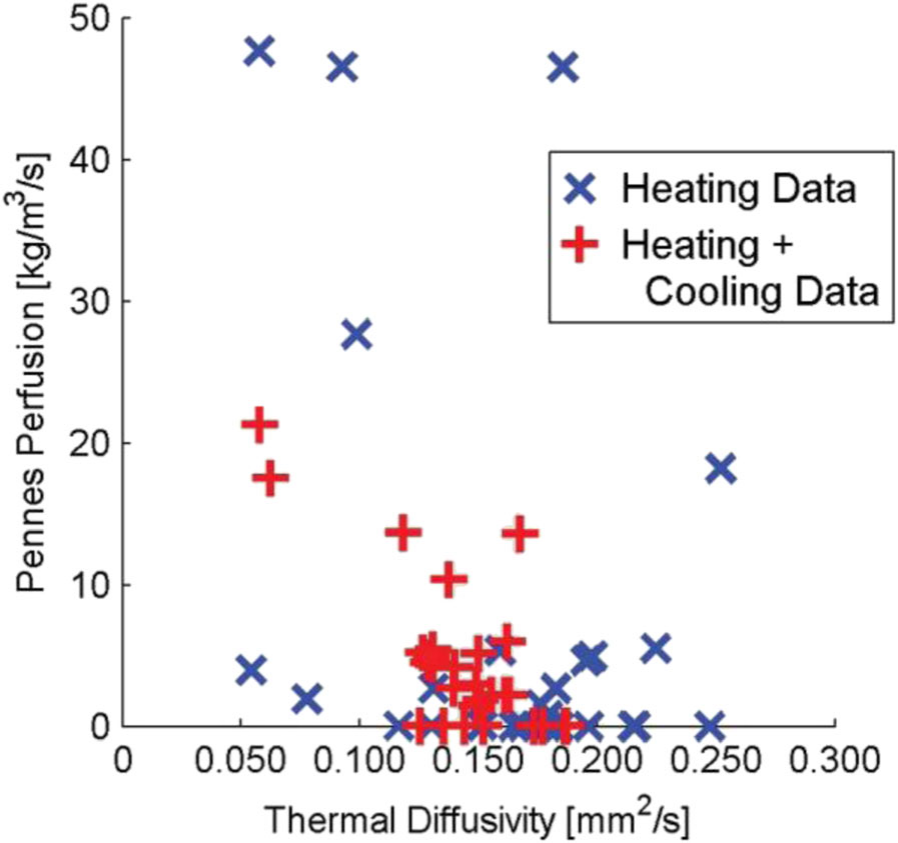
Estimates of thermal diffusivity and perfusion in rabbit back muscle. The shaded region indicates the range of thermal diffusivity values obtained by a standard invasive method

Figure 105 shows the results for all sonications in rabbit muscle. Blue × markers indicate t_fit_~ 24 s and red + markers utilize t_fit_~ 80 s. The shaded region identifies the range of *α* values obtained from invasive probe measurements (KD2 Pro, Decagon Devices, USA) applied after euthanasia. While there are no independent measures of *w* in this study, perfusion values for human resting muscle are 0.6 kg/m^3^/s [6]; higher values could be possible if FUS induces vasodilation, though not as high as the largest estimates. By including cooling data (red), results cluster more closely around the measured *α* range (shaded) and the anticipated low *w* levels.

**Fig. 106 (abstract O80).**
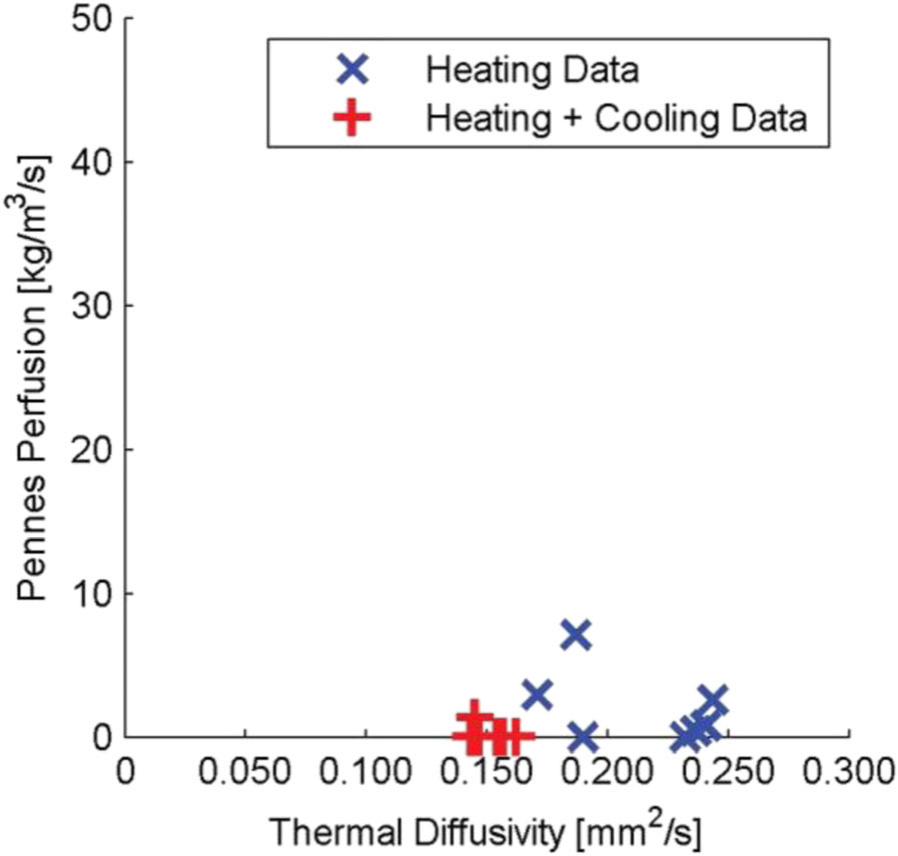
Estimates of thermal diffusivity and perfusion in a quality assurance phantom using clinical imaging protocols. The true value of perfusion is zero and that of thermal diffusivity is unknown

Estimates of *α* and *w* in a QA phantom are shown in Fig. 106. The phantom’s *α* is unknown, but, based upon similar phantoms, should be between 0.130-0.160 mm^2^/s. *w* should be 0 kg/m^3^/s due to no perfusion. Again, including the additional cooling data causes estimates to more closely converge anticipated property values. Thus, obtaining properties with clinical MRgFUS imaging is feasible, but it requires the acquisition of cooling images that are no longer allowed in some clinical systems.

**Fig. 107 (abstract O80).**
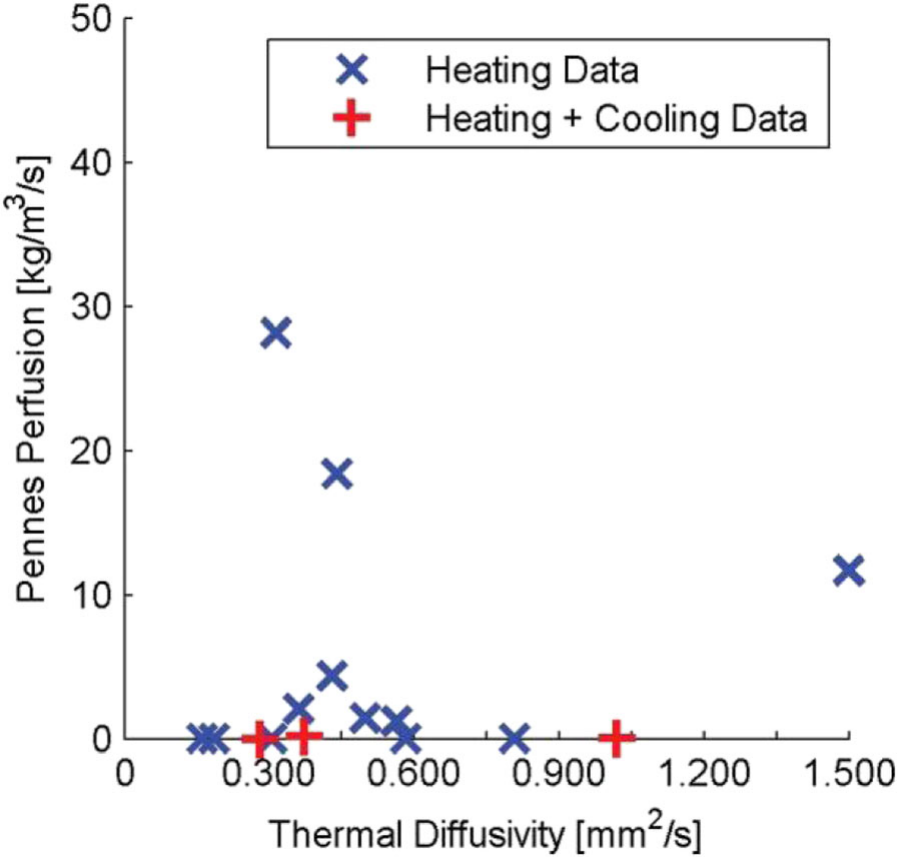
Estimates of thermal diffusivity and perfusion from clinical uterine fibroid treatments. The range of thermal diffusivity estimates is larger than expected. While known to vary greatly, there is limited quantitative information in the literature regarding fibroid perfusion, making it difficult to comment on the accuracy of perfusion estimates

Property estimates from clinical fibroid treatments are shown in Fig. 107. The magnitude and range of *α* estimates is higher than anticipated; one published study found uterine fibroid *α* from 0.066-0.176 mm^2^/s [7]. Only 2 of 12 estimates are within 10% of that range. When available (3 of 12 datasets), additional cooling data brought *α* estimates closer to expected values. Published data quantifying fibroid perfusion is limited, so it is difficult to comment on the accuracy of *w* estimates.

While the method can accurately estimate properties in vivo (rabbit data) and using clinical imaging with cooling data (phantom data), there is limited confidence in the clinical fibroid estimates. Possible explanations for this disconnect include increased noise and potential for chemical shift and motion artefacts when compared to the rabbit and phantom data.

Additionally, it is known that tissue attenuation, *w*, and *α* are temperature/dose dependent properties [8–10]. Assuming uniform, constant properties in clinical data where the temperature rise was as large as 54 °C is likely to introduce errors. In fact, the 2 clinical datasets providing the most realistic *α* estimates were those with the lowest temperature rise (9 and 11 °C). By utilizing the low temperature rise data from pretreatment sonications instead of therapeutic ablations, clinical estimates of *α* and *w* may improve. Future work will focus on investigating this possibility.

**Fig. 108 (abstract O80).**
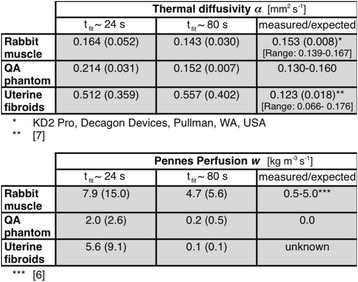
Summary of thermal diffusivity and perfusion estimates and measured/expected values presented as the mean with standard deviation in parentheses

A summary of all data is found in Fig. 108. Results are presented as the mean with standard deviation in parentheses.

**Conclusions**

This study shows that *a* and *w* can be estimated from in vivo data. Estimates improve when including cooling data beyond that which is clinically acquired and when the observed temperature change is less than 10 °C. Obtaining consistent property estimates from clinical data requires further investigation.

**References**

[1] Cline *et al.* 1994 *MRM* 31(6):628–636

[2] Dillon *et al.* 2012 *PMB* 57(14):4527–4544

[3] Dillon *et al.* 2014 *IJH* 30(6):362–371

[4] Dillon *et al.* 2015 *PMB* in press

[5] Rieke *et al.* 2004 *MRM* 51(6):1223–1231

[6] Hasgall *et al.* 2015 *http://www.itis.ethz.ch/virtual-population/tissueproperties/database*

[7] Zhang *et al.* 2014 *JMRI* 41(6):1654–1661

[8] Damianou *et al.* 1997 *J Acoust Soc Am* 102(1):628–634

[9] Prakash & Diederich 2012 *IJH* 28(1):69–86

[10] Choi *et al.* 2013 *J Heat Trans* 135(6):061302

### O81 Acceleration of thermal ablation of tissue volumes in high intensity focused ultrasound therapy using shock wave exposures

#### Vera A. Khokhova^1,2^, Peter V. Yuldashev^1^, Ilya Sinilshchikov^1^, Yulia Andriyakhina^1^, Tatiana D. Khokhlova^4^, Wayne Kreider^2^, Adam Maxwell^5^, Oleg Sapozhnikov^1,2^, Ari Partanen^3^

##### ^1^Physics Faculty, M.V. Lomonosov Moscow State University, Moscow, Russian Federation; ^2^Center for Industrial and Medical Ultrasound, University of Washington, Seattle, WA, United States; ^3^Clinical Science MR Therapy, Philips, Andover, MA, United States; ^4^Dept. of Gastroenterology, School of Medicine, University of Washington, Seattle, WA, United States; ^5^Dept. of Urology, School of Medicine, University of Washington, Seattle, WA, United States

**Objectives**

In high intensity focused ultrasound (HIFU) applications, nonlinear acoustic effects can result in the formation of high-amplitude shock fronts in focal waveforms, with amplitudes that can exceed 100 MPa. The presence of such shocks leads to increased tissue heating and initiation of boiling in milliseconds. Even though this shock-wave heating is very strong, shock fronts are highly focused and produce extreme heating effects in a very small focal volume. For single lesions, nonlinear heating thus can be utilized for rapid tissue ablation only within very small volumes before boiling starts to change the process. However, if the focus is steered, this enhanced heating combined with thermal diffusion can be used to accelerate thermal treatments over large volumes. The goal of this work was to evaluate the efficacy of using shock-wave heating to accelerate the thermal ablation of tissue volumes while keeping the same exposure conditions for intervening tissues.

**Methods**

**Fig. 109 (abstract O81).**
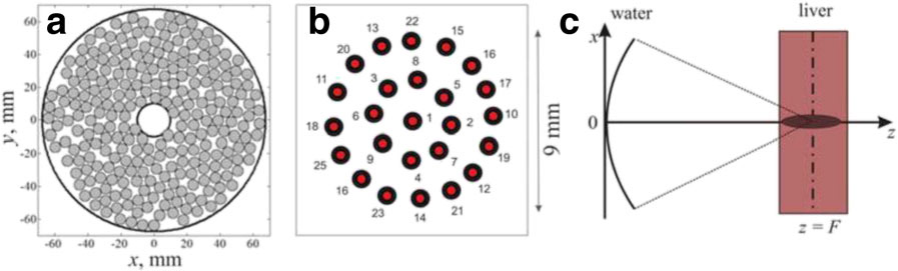
**a** Distribution of radiating elements on the surface of the therapeutic array; **b** Discrete trajectory of the beam's focus in the focal plane. **c** Geometry of the numerical experiment: ultrasound beam is focused within tissue layer (liver) of 5 cm thickness centred at the geometric focus of the spherical shell. Acoustical modelling was performed both in water and in liver sample, while temperature modelling was performed only in liver

Simulation studies were performed for a multi-element 1.2 MHz HIFU phased array of a clinical system (Fig. 109a, Sonalleve V1, Philips, Vantaa, Finland). Several acoustic power levels were considered within the possible range of array outputs from 104 W to 1300 W, corresponding to intensities at the array elements from 1.2 W/cm^2^ to 15 W/cm^2^. A pulsing scheme was combined with discrete electronic steering of the array focus over a series of targets arranged in circles with radii of 2 and 4 mm (Fig. 109b) to generate volumetric lesions in *ex vivo* bovine tissue. Point numbers in the sonication sequence are indicated in the Fig. 109b. The circles were positioned in a plane at 25 mm depth in a bovine liver tissue sample of 50 mm thickness (Fig. 109c), and the period between consecutive pulses was 40 ms.

Acoustic field in tissue was modelled using the Westervelt equation and a previously developed finite-difference algorithm. Temperature modelling in tissue was conducted using the bioheat equation with heat sources calculated from the acoustic modelling. Temperature simulations were optimized in the following way. First, the effect of a sonication at a single focus was computed in the time domain on a fine grid, which covered only the focal volume until the diffused temperature distribution was broad enough to be transferred to a sparser grid that covered the entire tissue sample. Second, volumetric modeling of the bioheat equation was conducted on the sparser grid in a spatial-frequency domain where an analytic solution is available. Based on the linearity of the bioheat equation, the temperature distribution calculated at the first step was added to the current temperature distribution in tissue at each consecutive steering position of the focus with the time delay equal to the heating and diffusion time of the sonication in a single focus.

Temperature simulations were conducted for a constant time-average intensity at the array elements, considering either a peak intensity of 1.2 W/cm^2^ over a HIFU pulse duration of 20 ms, 8 W/cm^2^ over a duration of 3 ms, or 15 W/cm^2^ over a duration of 1.6 ms. For comparison, the initial intensity of 1.2 W/cm^2^ corresponds to a total acoustic output power of 104 W. Sonication of targets was performed starting from the centre of the circles spiraling outward and continued until the minimum temperature rise inside the circle of 4 mm radius in the focal plane reached 45°C. Assuming *ex vivo* exposures with initial temperature of 20°C, this temperature rise is representative for tissue denaturation within the irradiated volume. Here we term the focal plane as the plane of the maximum heat deposition for each intensity output. To equalize the temperature over each circle, the distribution of sonication points was rotated with each consecutive cycle to irradiate locations in between the sonication points of the previous cycle (Fig. 109b).

**Results**

**Fig. 110 (abstract O81).**
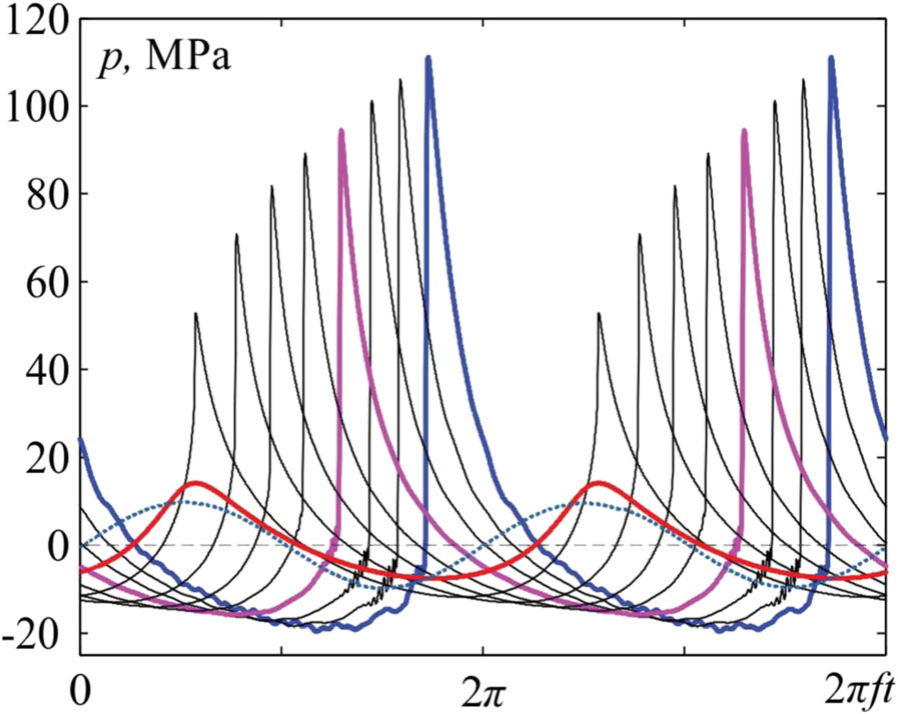
Acoustic pressure waveforms simulated at the maximum of heat deposition in tissue for different intensities at the array elements: 1.2 W/cm^2^ (red curve), 4, 5, 6, 7, 8 W/cm^2^ (pink curve), 10, 12, and 15 W/cm^2^ (*blue curve*). Dashed curve is the focal waveform for 1.2 W/cm^2^ simulated linearly. While quasilinear regime of focusing with slightly distorted focal waveform is realized for initial intensity of 1.2 W/cm^2^, high amplitude shocks are formed at intensities of 8 W/cm^2^ (95 MPa) and 15 W/cm^2^ (118 MPa)

Shown in Fig. 110 are waveforms simulated at the position of maximum heat deposition in tissue for different peak intensities at the array elements from 1.2 to 15 W/cm^2^. These output levels corresponded to quasilinear sonication (1.2 W/cm^2^, red curve), sonication with a fully developed shock of 95 MPa at the focus (8 W/cm^2^, pink curve), and sonication with a higher focal shock amplitude of 118 MPa (15 W/cm^2^, blue curve).

**Fig. 111 (abstract O81).**
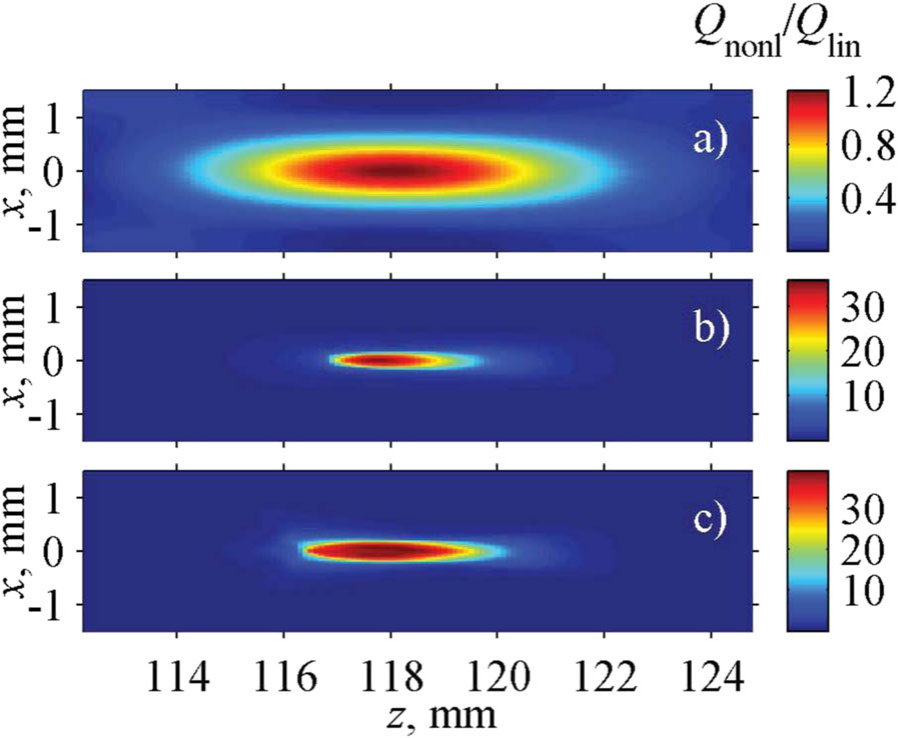
Spatial 2D distributions of heat deposition rates in tissue in the axial plane of the array for different intensities at the array elements: **a** 1.2 W/cm^2^, **b** 8 W/cm^2^, and **c** 15 W/cm^2^. Heat deposition rates are normalized to the heating rates calculated at the same intensities assuming linear wave propagation conditions. The distributions therefore illustrate nonlinear enhancement and better spatial localization of heating using shock-wave exposures

Shown in Fig. 111 are spatial distributions of heat deposition rates in tissue in the axial plane of the array simulated for different peak intensities at the array elements. Heat depositions are normalized relative to the heat deposition calculated for the same intensities assuming linear wave propagation. It is seen that at 8 W/cm^2^ with a shock front of about 100 MPa, the tissue heating at the focus is about 35 times higher than predicted linearly and is highly localized in space. With further increase of the intensity to 15 W/cm^2^, the shock front at the focus is about 120 MPa resulting in 40 times more effective heating. In addition, the region of the presence of high-amplitude shock and therefore enhanced heating is enlarged.

**Fig. 112 (abstract O81).**
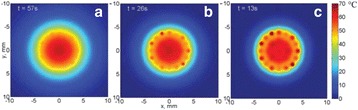
Spatial 2D distributions of temperature rise in tissue in the plane of maximum heat deposition for different peak intensities at the array elements balanced by the pulse length within 40 ms time window between the pulses: **a** 1.2 W/cm^2^ and 20 ms, **b** 8 W/cm^2^ and 3 ms, and **c** 15 W/cm^2^ and 1.6 ms. As indicated in each frame, temperature maps are shown at the time point when temperature rise everywhere inside the circle of 4 mm radius reaches 45°C. This temperature rise would ensure tissue denaturation for *ex vivo* exposures with initial tissue temperature of 20°C

Corresponding temperature distributions in the focal plane are shown in Fig. 112 at times when the temperature rise inside the circle of 4 mm radius reaches 45°C. For the lowest peak intensity (1.2 W/cm^2^) this temperature level was reached in 84 seconds. For higher outputs it required 34 and 13 seconds, correspondingly, showing significant acceleration of the treatment. At higher peak intensities, the volumetric temperature rise occurred more quickly while the boundary between treated and untreated tissue became more clearly defined.

**Conclusions**

HIFU irradiations of tissue volume in three different nonlinear regimes were considered. It was shown that with the same time-average power, the use of pulsing schemes with higher peak power leads to faster heating of the desired volume and less heat diffusion to the surrounding tissues. Such regimes therefore show clinical advantages to accelerate thermal HIFU therapy while keeping the same safe exposure conditions for surrounding tissues and sharper margins of treatment. The study was supported by the RSF 14-12-00974, NIH NIBIB EB007643, and student Global Internship Program from the Focused Ultrasound Foundation.

### O82 Ultrasonic hemostasis of deep arterial bleeding

#### Andrey Rybyanets^1^, Natalia Shvetsova^1^, Alex Berkovich^2^, Igor Shvetsov ^1^, Oleg Sapozhnikov^3^, Vera Khokhlova^3^

##### ^1^Southern Federal University, Rostov on Don, Russian Federation; ^2^Saint Petersburg Polytechnical University, Saint Petersburg, Russian Federation; ^3^Moscow State University, Moscow, Russian Federation

**Objectives**

Ultrasonic hemostasis may provide an effective method in surgery and prehospital settings for treating trauma and elective surgery patients. Application of HIFU therapy to hemostasis was primarily initiated in an attempt to control battlefield injuries on the spot. High-intensity focused ultrasound (HIFU) has been shown capable of coagulation of internal bleeding. The main drawback of the thermal hemostasis strategy is low ultrasound absorption ability of blood and, as a result, low heating and coagulation rate at real blood flow.

The purpose of this study was to evaluate the feasibility of HIFU thermal and combinational (cavitation, boiling, non-linear behaviors, and coagulation agents) effects for ultrasonic hemostasis of deep arterial bleeding.

**Methods**

In this paper, HIFU therapy and imaging transducer designs, nonlinear acoustic fields modeling and calculations, as well as in vivo hemostasis experiments on lamb’s femoral artery confirming enhanced ultrasonic hemostasis at deep arterial bleeding are described. For ex vivo and in vivo hemostasis experiments an ultrasonic applicator was designed and tested. The ultrasonic applicator design had HIFU therapy transducer and imaging probes and was configured to be compatible with 3D mechanical scanning system. HIFU transducers are comprised 1–2 MHz spherical elements made from porous piezoceramics with 80 mm aperture having radius of curvature 40–60 mm. Centre opening with 40 mm diameter was reserved for ultrasonic imaging and Doppler probes such as linear, convex or 3D arrays.

Acoustic measurements of ultrasonic transducers have been performed in 3D Scanning System (UMS3) using the fiber optic hydrophone (FOPH 2000) and using AFB from Precision Acoustics Ltd. Waveforms from the hydrophones and the driving voltage were recorded using a digital oscilloscope Lecroy. The transducer was driven by a function generator Agilent 33521B, a linear RF amplifier E&I model 2400L RF, and operates in a CW or burst modes. The acoustic intensity in the focal plane measured in water tank at 1000–5000 W/cm^2^ (ISAL) was kept for the objects treatment. The experiments were made on acoustic vascular phantoms, as well as on lamb’s femoral artery in vivo at different protocols. During ultrasound exposure of lamb’s femoral artery, arterial blood flow was temporarily stopped using intravascular balloon. In some protocols intravenous coagulation agents (liposomes) activated by HIFU at the point of bleeding were used. Targeting accuracy was assessed by necropsy and histologic exams and efficacy (vessel thrombosis) by angiography and histology.

**Results**

New effective HIFU therapy and imaging transducer designs and treatment protocols for ultrasonic hemostasis of deep arterial bleeding were developed and evaluated. The results of theoretical calculations and modelling along with the acoustic measurements of non-linear ultrasonic fields were presented. The results of ex vivo experiments on tissues and vascular phantoms allowed to choose the optimal HIFU treatment protocols. In vivo hemostasis experiments on lamb’s femoral artery confirmed enhanced thermal effect of HIFU in non-linear regimes. Using of HIFU transducers with resonant frequency of 1,6 MHz at intensity of 5000 W/cm^2^ (ISAL) allowed to stop bleeding from major blood vessels that were punctured with an 18- or a 14-gauge needles during 3–15 sec.

Thrombogenic evidence (blood clotting) and collagen denaturation (vessel shrinkage) were found in necropsy and histologically in all targeted arterial vessels with minimal damage to adjacent tissue structures. Coagulation cascade behaviors (postponed thrombosis) was also observed during next few hours after treatment. Thrombogenic efficiency of intravenous coagulation agents (liposomes) activated by HIFU at the point of bleeding was also demonstrated.

**Conclusions**

We have demonstrated that HIFU can be used to stop active bleeding from vascular injuries including punctures and lacerations. The coagulation strategy that employ thermal and combinational effects (cavitation, boiling, non-linear behaviours, and coagulation agents) for fast ultrasonic hemostasis of deep arterial bleeding has been proposed. The results of theoretical modelling, ex vivo experiments on tissues and vascular phantoms, as well as in vivo experiments in lamb’s femoral artery proved the efficacy, safety and selectivity of developed HIFU transducers and combinational treatment methods that can be used for various therapeutic, surgical and cosmetic applications.

**Fig. 113 (abstract O82).**
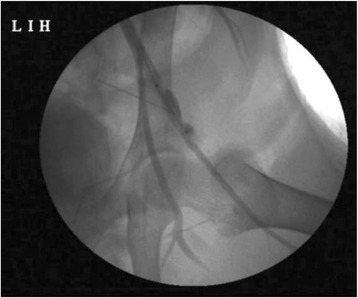
Angiography image of blood vessels

**Fig. 114 (abstract O82).**
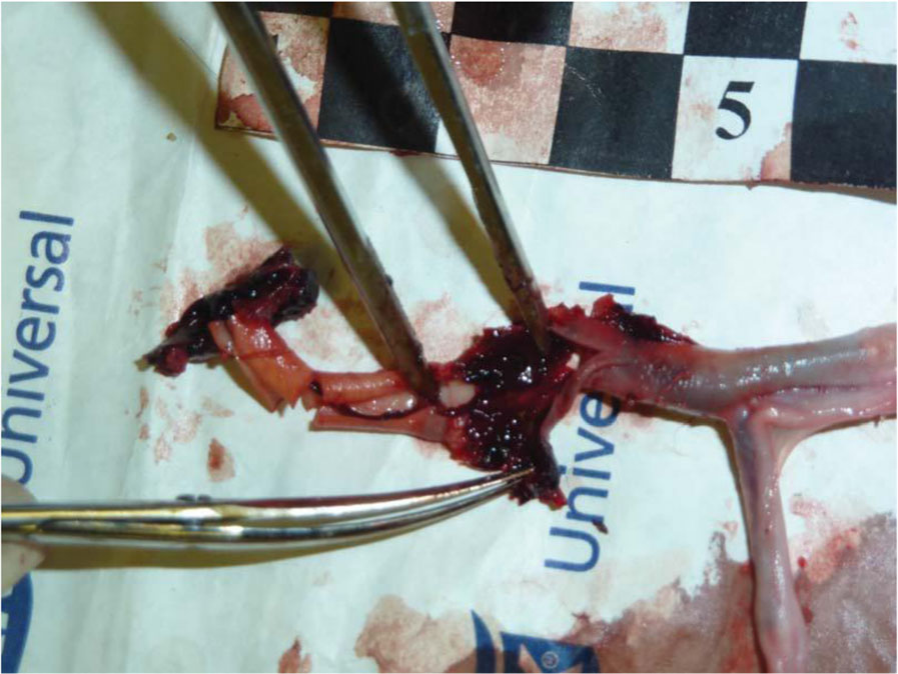
Photograph of vessel thrombus in dissected femoral artery

**Fig. 115 (abstract O82).**
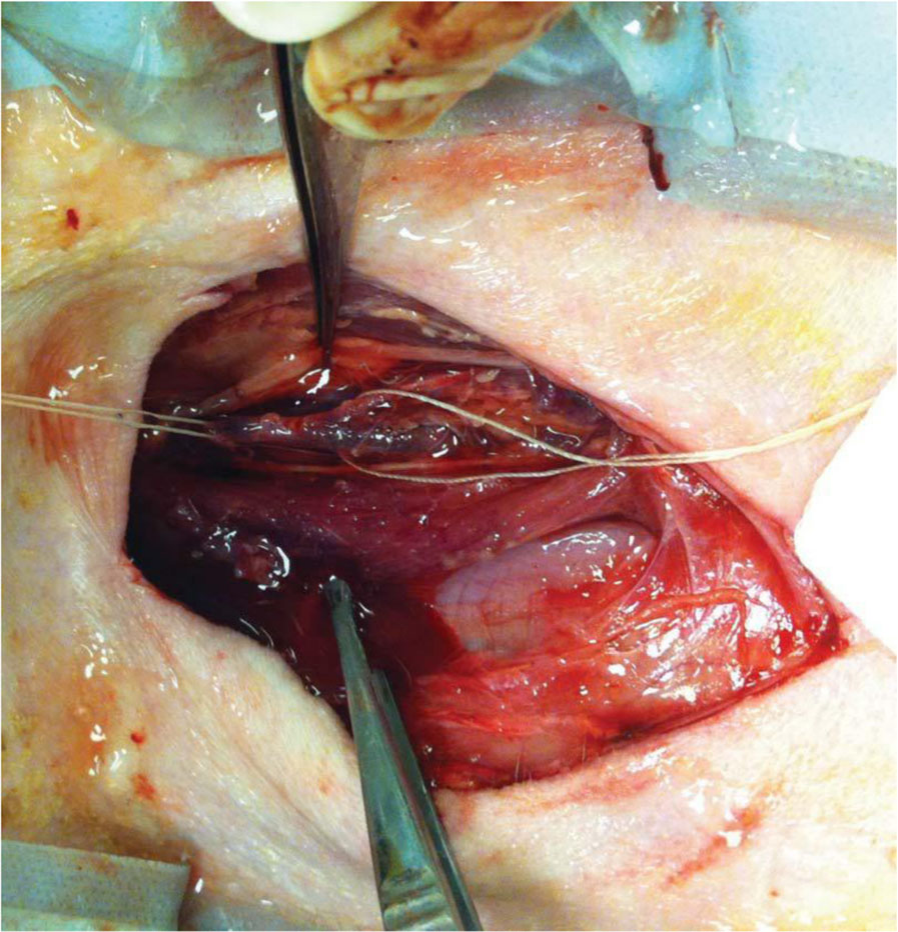
Lamb's femoral artery

### O83 High intensity focused ultrasound exposure of placental vasculature

#### Caroline J. Shaw^1,3^, Ian Rivens^2^, John Civale^2^, Dino Giussani^1^, Gail ter Haar^2^, Christoph Lees^3^

##### ^1^Department of Physiology, Development and Neuroscience, University of Cambridge, Cambridge, United Kingdom; ^2^Joint Department of Physics, The Institute of Cancer Research, London, United Kingdom; ^3^Institute of Developmental and Reproductive Biology, Imperial College London, UK , London, United Kingdom

**Objectives**

Twin-Twin Transfusion Syndrome (TTTS), resulting from vascular anastomoses within monochorionic placentae, is a major cause of prematurity, death and handicap in human twin pregnancy. Fetoscopic laser ablation of these anastomoses is an established therapy but carries significant fetal and maternal risks due to invasion of the intrauterine space and confers no survival advantage. High intensity focused ultrasound (HIFU) is a non-invasive treatment which can be used to ablate tissue and occlude blood vessels. In TTTS a non-invasive method of vascular ablation could reduce associated risks and increase the scope of treatment. We aimed to use existing HIFU technology to target and ablate placental vasculature in the pregnant sheep model, which has vessels similar to human monochorionic placental vascular anastomoses, to assess the clinical potential of HIFU. Previously, results of a preliminary study in which placental vessels were exposed through the uterine wall which had been surgically exteriorized to allow invasive instrumentation for monitoring of fetal and maternal physiology have been presented. In this study, non-invasive, trans-cutaneous, placental vessel occlusion was attempted using a standardised treatment protocol which was designed to study efficacy & safety

**Methods**

HIFU (1.66 MHz, 5 s) exposures in 5 anaesthetised ovine pregnancies were placed 5 s, and 2 mm, apart using a treatment depth dependent range of free-field exposure levels (Ispta from 2000 to 5000 Wcm-2) applied non-invasively through cleaned and depilated abdominal skin (coupled through a bag filled with degassed water. Up to 6 placental vasculature targets were identified using a colour Doppler ultrasound probe (P10-4, Z.One Zonare) mounted behind a 19 mm diameter central aperture in the HIFU transducer (Sonic Concepts H148MR: 64 mm diameter, 63 mm focal length). Exposures were delivered using a purpose written Matlab GUI which controlled the exposure parameters (frequency and power level by controlling the signal generator settings), exposure timing and position of an automated gantry holding the treatment head precisely using a signal generator. Between 4 and 7 exposures were placed in each placentome during a single mechanical ventilation pause of up to 90 seconds. Ventilation pauses were at least 3 minutes apart to allow normalisation of maternal oxygen saturation and end tidal CO_2_. Cavitation was detected passively during most exposures using a sensor mounted on the side of the therapy device and with its focal zone coaligned with the HIFU focal peak. Tissue harmonic B-mode imaging data were used to identify whether hyperecho occurred during exposure. Treatment success was determined as an absence of detectable flow on colour Doppler (on the minimum velocity setting) immediately post treatment. After exposure, animals were allowed to recover, and monitored for obstetric complications. They were sacrificed 21 days post-exposure and a post mortem was conducted to identify exposed placental vessels and any iatrogenic harm to mother or fetus. Exposed and control (unexposed) samples were fixed in 4% formalin for 5 days, embedded in paraffin wax, and histological examination was performed on unstained, and on Haematoxylin and Eosin stained 10 μm thick sections.

**Results**

Target vessels were identified in 28 sites, in 5 sheep, using colour Doppler measurements made using the imaging capability of the combined imaging and treatment head. HIFU occlusion was successful in 27 of 28 targeted vessels as indicated by an absence of flow previously detected using colour Doppler imaging performed at the pre-treatment target identification position. Two exposures were initially unsuccessful, one of which was retreated effectively, and in the other, retreatment was not attempted due to side effects in the overlying skin. The majority of exposures demonstrated significant levels of either broadband emissions indicative of inertial cavitation, or solely of half harmonic emissions, suggestive of stable cavitation activity. US imaging obtained during HIFU exposure often, but not always, demonstrated the creation of hyperechoic regions, usually towards the end of the exposure duration, and during more than one exposure. The exposure level chosen for the given treatment depth in the first animals resulted in skin burns. This was eradicated or reduced to mild erythema by (i) more thorough cleaning and depilation, with reduced depilation time to reduce inflammatory response, (ii) reduction of the exposure levels by approximately 1 dBm and (iii) the use of ice to cool the degassed water in the coupling bag. Post mortem examination demonstrated no evidence of iatrogenic harm to mother and fetus, with normal fetal growth and development and no evidence of obstetric complications. The exposed samples demonstrated macroscopic evidence of tissue damage surrounding placental vessels. Histological examination is currently on going, but initial results show evidence of clotting within exposed vessels

**Conclusions**

We report the first successful use of non-invasive, ultrasound guided, HIFU to target, ablate and monitor placental vasculature ablation in a pregnant sheep model. The exposure conditions used appear to be effective and safe. This proof of concept study demonstrates potential for the clinical translation of this technique.

### O84 Simultaneous monitoring of MR-ARFI and MR-thermometry during HIFU ablation

#### Pierre Bour^1,2^, Fabrice Marquet^1^, Valery Ozenne^1^, Solenn Toupin^1^, Bruno Quesson^1^, Erik Dumont^2^

##### ^1^IHU-Liryc, Pessac, France; ^2^Image Guided Therapy, Pessac, France

**Objectives**

MR-guided High Intensity Focused Ultrasound (MRgHIFU) allows non-invasive ablation of pathological tissue. Temperature measurement via the Proton Resonance Frequency Shift (−PRFS) along with online thermal dose calculation is commonly used as the principal monitoring parameters. Alternatively, the local displacement of soft tissues induced at the HIFU focal point by Acoustic Radiation Force Impulse (ARFI) can be encoded in the phase of the MR image with a motion-sensitive encoding gradient (MEG) synchronized with short ultrasound pulses. Although temperature monitoring provides essential information on thermal ablation advancement, no information are provided on tissue physical properties modifications that can alter the focusing quality of the HIFU beam during the treatment. In the present work, we propose a fast implementation of a simultaneous MR-ARFI/thermometry allowing subsecond multi-slice imaging of temperature and displacement during HIFU ablation. A rapid MR method providing simultaneous ARFI and temperature monitoring with sufficient spatial coverage would be beneficial to provide better control of the therapeutic process and improve patient safety.

**Methods**

**Fig. 116 (abstract O84).**
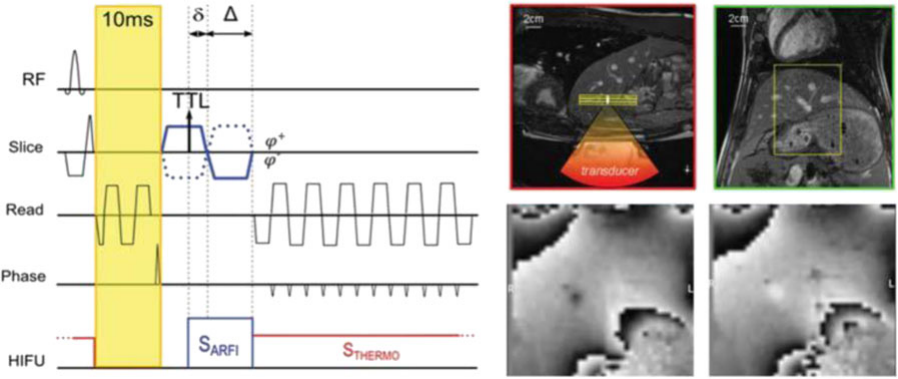
(*left*) Single-shot GRE-EPI MR-ARFI/thermometry sequence integrating MEG before echo train with alternating polarities (*blue*) to encode both temperature and ARFI. The 10ms delay (*yellow box*) represents tissue relaxation before the next ARFI encoding. Synchronization of HIFU sonication with MR sequence is depicted here by the external trigger TTL. (SARFI) and (STHERMO) HIFU shot schematically represented. (*right*) 2D multi slice balanced-SSFP images used for targeting and localization of HIFU transducer. Transverse orientation is in blue border, sagittal in red and coronal in green. Yellow box represents tree slices positioned around focus acquired with the modified EPI sequence

All experiments were performed at 1.5T (Siemens Avanto, Erlangen, Germany) with a MR compatible High Intensity Focused Ultrasound platform (Image guided Therapy SA, Pessac, France). It consisted of a 256 elements phase array transducer (focal length 13 cm, aperture 13 cm, 1 MHz operating frequency). A single shot echo planar imaging sequence was modified to insert a bipolar motion encoding gradient (MEG) before the echo train (Fig. 116). The MEG direction was chosen parallel to the acoustic propagation axis to encode longitudinal displacements induced by HIFU pulses, with adjustable duration (D) and amplitude (A). The polarities of the MEG were alternated for each dynamic to encode displacement into the phase image with either a positive or negative contribution. An analogic synchronization pulse (TTL) was generated from the sequence and sent to the trigger input of the HIFU generator. Its timing (δ) could be adjusted relative to the beginning of the second lobe of the MEG. The HIFU sonication was split in two independent shots with amplitudes noted S_ARFI_ and S_THERMO_.

Sequence parameters were:

*Ex-vivo*: 3 coronal slices with fat saturation, FOV= 92x147mm^2^, TR/TE/FA = 200ms/29ms/45°, voxel size=2.3x2.3x5mm^3^, with a bandwidth of 1500Hz per pixel, Grappa acceleration (factor = 2), A=25mT/m, =5.3ms and δ= 3ms for the MEG *-In-vivo:* 3 slices in the coronal plan with fat saturation and saturation slabs surrounding the FOV in the phase direction,

FOV=156x170mm^2^, TR/TE/FA = 500ms/28ms/50°, 2.3x2.3x5mm3 voxel size, with a bandwidth of 2003Hz per pixel, A=25mT/m, =5.3ms and δ= 3ms for the MEG.

Proof of feasibility was performed on one healthy volunteer using 6 elements brain array coil, 10 slices were acquired during 3min with FOV=156x170mm^2^, TR/TE/FA = 1000ms/28ms/60°, 1.6x1.6x3.mm^3^ voxel size, with a bandwidth of 1447Hz per pixel, A=25mT/m, =5ms for the MEG. 3 orthogonal orientations were successively acquired. Results were analysed by the calculation of the standard deviation of temperature and displacement along acquisition time on whole brain and on region in centre of the brain where HIFU ablation are currently performed.

**Results**

This sequence and the associated post-treatment allow real-time visualization of temperature and ARFI displacement with a temporal resolution up to 75ms with the minimum repetition time and a spatial resolution of 2.3x2.3x5 mm^3^.

For *ex-vivo* ablations (N=15) S_ARFI_ = 340W, S_THERMO_ = 255W and sonication duration was 60s at the natural focus position. At the end of sonication, a temperature increase of 15±0.9 °C was observed. Initial ARFI displacement measured was 30±1.1μm and progressively decreased to reach 21μm at the end of sonication.

**Fig. 117 (abstract O84).**
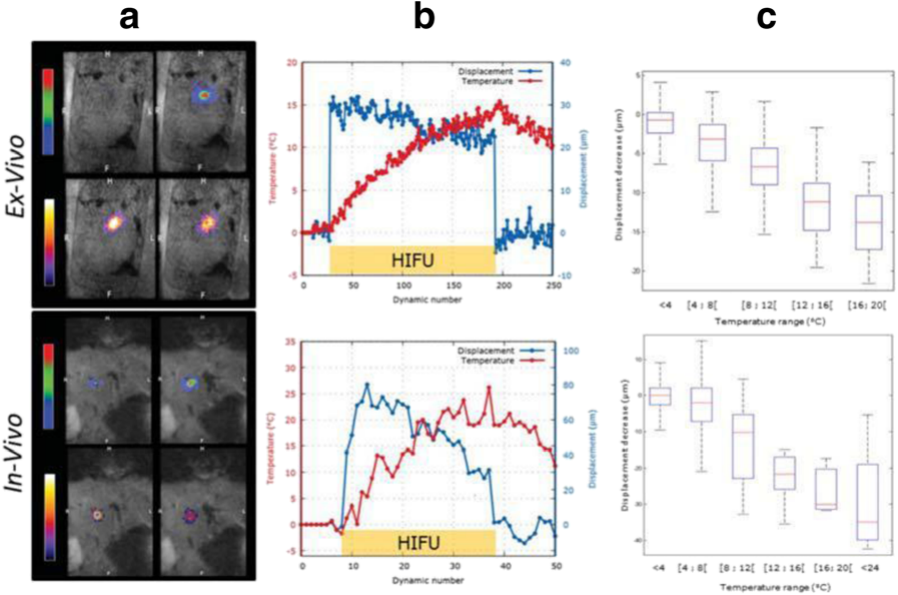
Results for in vivo and ex vivo. **a** Temperature (*top*) coronal images obtained at the beginning (*right*) and at the end of heating period (*left*). **b** Time course of ARFI displacement (*blue*) and Temperature (*red*) of a single pixel at the focus. **c** Represents a box plot of the displacement variation on populations of temperature increase of 4 degrees. A mask of 20x20 pixels around the focus was applied on data with a 3x3 Spatial Gaussian filter

For *in-vivo* ablations (N=10) S_ARFI_ = 425W, S_THERMO_ = 425W and sonication duration was 30s at the natural focus position. Apnea was maintained for 45s and no respiratory motion was observed. At the end of sonication, a temperature increase of 25±1.2 °C was measured. ARFI displacement measured exhibited an increase then decrease pattern with a maximum found for an absolute temperature of 49°C (see Fig. 117).

In both *in-vivo* and *ex-vivo* ablations the decrease of ARFI displacement (Fig. 117) described a linear curve fitted as y (T [°C]) =−0.90T+2.19 for *ex-vivo* and y (T [°C]) =−1.82T+9.46 *in-vivo.*

**Fig. 118 (abstract O84).**
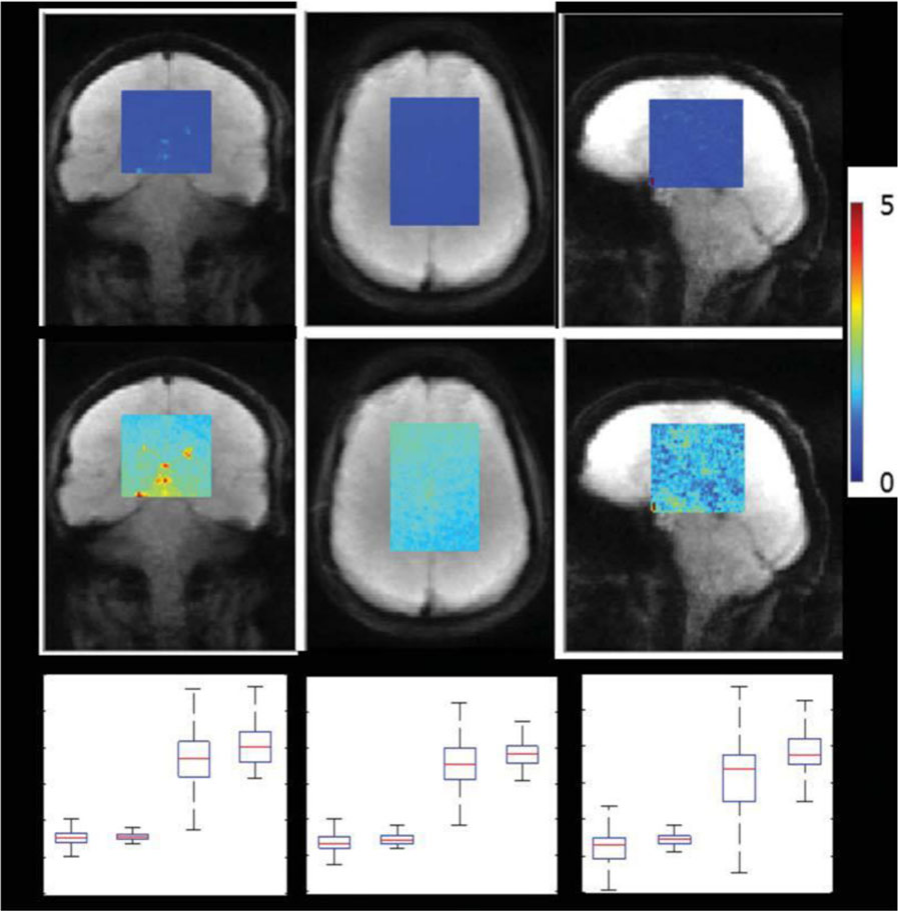
Results on healthy volunteer of standard deviation for temperature and ARFI displacement on 10 slices during 3min acquisition. In boxplot 1:T_STD_ in centre of the brain 2:D_STD_ in centre of the brain 3:T_STD_ on whole brain 4:D_STD_ on whole brain

On Fig. 118 standard deviation of temperature and ARFI displacement, in all orientations, are shown in a region in centre of the brain. The refresh rate was 1Hz for 10 slices.

**Conclusions**

In this study, a HIFU ablation monitoring method has been validated *in-vivo* in pig liver and *ex-vivo* in pig muscle. This fast sequence enabled multi-slice acquisitions while remaining at a sub second temporal resolution. In addition, improvement of the sonication duty cycle allowed fast ablation with the possibility to independently control the power for ARFI encoding and the amount of power to induce a heating. During HIFU ablation monitoring, this method allowed visualization of temperature and ARFI displacement time evolution. For *in-vivo* and *ex-vivo* ablations, a significant decrease in ARFI displacement was observed along the ablation attesting a stiffening of the tissue at the focus. This qualitative index of tissue stiffness could give additional information on ablation process.

$$ \begin{array}{l}\Delta {T}_i={\left(-1\right)}^i\frac{\left({\varphi}_i^{+}-{\varphi}_{ref}^{+}\right)+\left({\varphi}_{i-1}^{-}-{\varphi}_{ref}^{-}\right)}{2.\gamma .\alpha . TE. B0}\\ {}\Delta {D}_i={\left(-1\right)}^i\frac{\left({\varphi}_i^{+}-{\varphi}_{ref}^{+}\right)-\left({\varphi}_{i-1}^{-}-{\varphi}_{ref}^{-}\right)}{2.\gamma .\left| A\right|.\Delta}\end{array} $$

Equation 1: T and D represent temperature and displacement images at the i^th^ dynamic in the time series. δ denotes phase images and (+, −) stands for polarity of the MEG gradient, whereas “ref” stands for reference phase data acquired prior to sonication. Other parameters are the gyromagnetic ratio , the PRFS constant , the echo time TE and the static magnetic induction B_0_ = 1.5T, A and the duration and amplitude of the MEG gradient, respectively.

### O85 MR thermometry guided hyperthermia with real-time ultrasound beamforming and power control on a commercial ablation system

#### Eugene Ozhinsky^1^, Vasant Salgaonkar^2^, Chris Diederich^2^, Viola Rieke^1^

##### ^1^Radiology and Biomedical Imaging, University of California San Francisco, San Francisco, CA, United States; ^2^Radiation Oncology, University of California San Francisco, San Francisco, CA, United States

**Objectives**

Hyperthermia (HT, 40-45°C, 30–60 min) has been combined successfully with several cancer treatment modalities, such as radiation, chemotherapy and drug delivery [1,2] to enhance treatment efficacy.

We have implemented an MR-guided hyperthermia (MRgHT) platform by performing operational modifications to a commercially available MR-Guided Focused Ultrasound (MRgFUS) ablation system within its hardware and software constraints. This platform enables automated modulation of acoustic power and changes in sonication beam patterns based on real time MR thermometry feedback to produce long-duration hyperthermia in large contiguous volumes. It is currently demonstrated for an endorectal focused ultrasound array already in clinical trials for prostate ablation. With strong clinical rationale for HT augmented radiation and chemotherapy for several cancers, this MRgHT platform has potential for rapid clinical translation.

**Methods**

**Fig. 119 (abstract O85).**
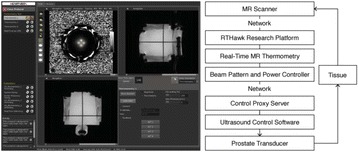
*Left*: user interface of the MR-guided hyperthermia platform. It includes multi-plane MR thermometry and phased array controller module. *Right*: diagram of the MR thermometry-based HT system

The real-time thermometry application (Fig. 119) was developed for the RTHawk real-time MRI system (HeartVista, Inc., Menlo Park, CA), connected to a 3T MR scanner (GE Healthcare, Waukesha, WI) and the 2.3-MHz ExAblate 2100 prostate array (InSightec, Haifa, Israel). The application included an SPGR pulse sequence (TE = 13.4 ms, FOV = 28–32 cm, 3 s/slice), a real-time PRFS thermometry reconstruction pipeline and a custom interface for data visualization and prescription. The system provided for interleaved simultaneous acquisition of multiple slices at different orientations, with customizable ROIs for temperature measurement and feedback control.

The beam controller module was developed for the RTHawk MR Thermometry application (Fig. 119). Its features included multi-point temperature sampling, feedback power control and real-time switching of custom beam patterns. To interface with vendor ultrasound control software, a Control Proxy Server application was developed. It accepted connections from the beam controller module over the local area network and translated the commands received over this connection into calls of the vendor-provided software interface.

The delivery of hyperthermia was performed in a tissue mimicking phantom with heating for 16 min at 1.3 W/cm^2^ array surface intensity. We implemented temperature monitoring in four regions of interest (ROI) in a diamond pattern (20 mm separation) and switching the ultrasound beam focus to the ROI with minimum temperature. Transducer output power was controlled not to exceed the target temperature of 7°C.

**Results**

**Fig. 120 (abstract O85).**
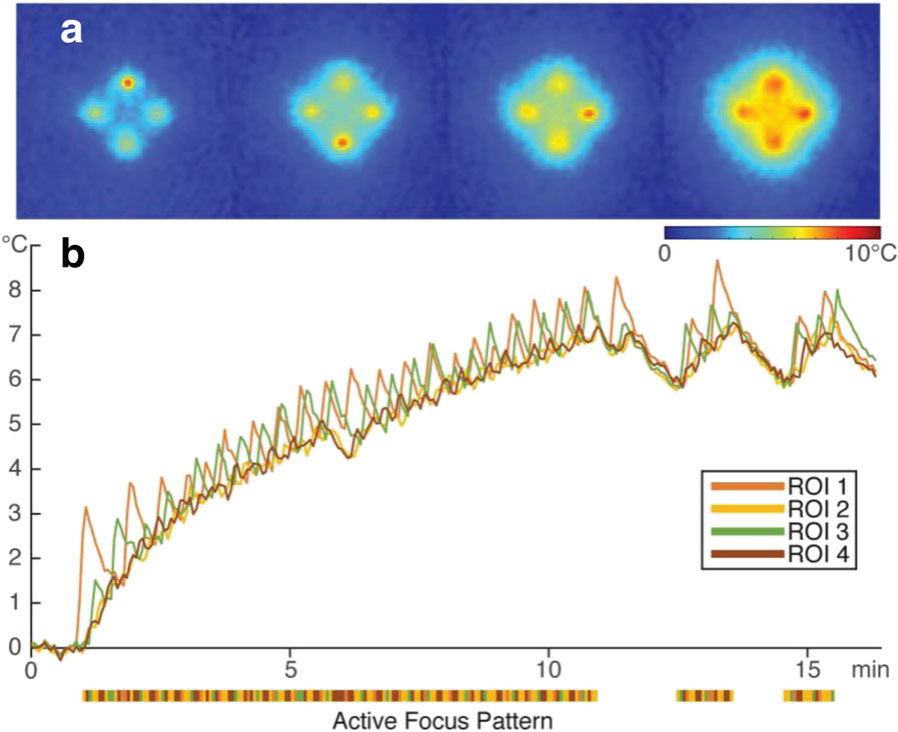
**a** Thermal images, captured during MR thermometry guided switching between single focus patterns and at steady state; **b** Plot of mean temperature within the four ROIs during the heating experiment. Colour bar below shows active single focus heating pattern matched to the corresponding ROI

Figure 120a shows MR thermometry images, acquired during the heating stage and after approaching steady state. Uniform temperature increases within a 1°C envelope were observed in all ROIs (Fig. 120b). HT uniformity could be further improved by using a larger number of focus points with independent control and custom multi-focus beam patterns.

**Conclusions**

We have implemented a real-time MR thermometry-guided ultrasound beam control system integrated with the ExAblate platform for long duration prostate hyperthermia therapy and validated it in phantom experiments. Future work will focus on implementing phased patterns for different treatment scenarios (Salgaonkar, et al. Med Phys 2014) and in-vivo validation.

This work was supported by Focused Ultrasound Foundation, NIH R01CA12276, R01CA111981, R00HL097030, UCSF-RAP.

**References**

[1] Wust, et al. The Lancet Oncology 2002

[2] Ponce, et al. Int J Hyperthermia 2006

### O86 Novel application of MR-HIFU for ablation of sacroiliac joint in sub-acute swine model

#### Elena Kaye, Sebastien Monette, Majid Maybody, Govindarajan Srimathveeravalli, Stephen Solomon, Amitabh Gulati

##### Memorial Sloan Kettering Cancer Center, New York, NY, United States

**Objectives**

The Sacroiliac Joint (SIJ) is a common pain generator in both arthritic and metastatic disease [1]. Successful denervation of SIJ pain requires complete interruption of the lateral branch nerves innervating the POLYMERIC CUPS AS NANOSCALE CAVITATION NUCLEI FOR ACTIVE TRANSPORT with the inability to directly visualize the targeted nerves, introduces substantial uncertainty in successful outcomes for current denervation approaches. Percutaneous radiofrequency neurotomy (RFN) requires insertion of multiple RF probes along the SIJ in order to achieve the desired lesion. However, clinically, the finite number of the RF probes makes creation of the continuous lesion challenging and elevates the risk of thermal lesions incompletely disrupting the targeted innervation. Magnetic Resonance guided High Intensity Focused Ultrasound (MRgHIFU) can potentially serve as a more reliable and non-invasive treatment of SIJ pain. The goal of this study was to study the feasibility of MRgHIFU denervation of the SIJ in a sub-acute swine model.

**Methods**

**Fig. 121 (abstract O86).**
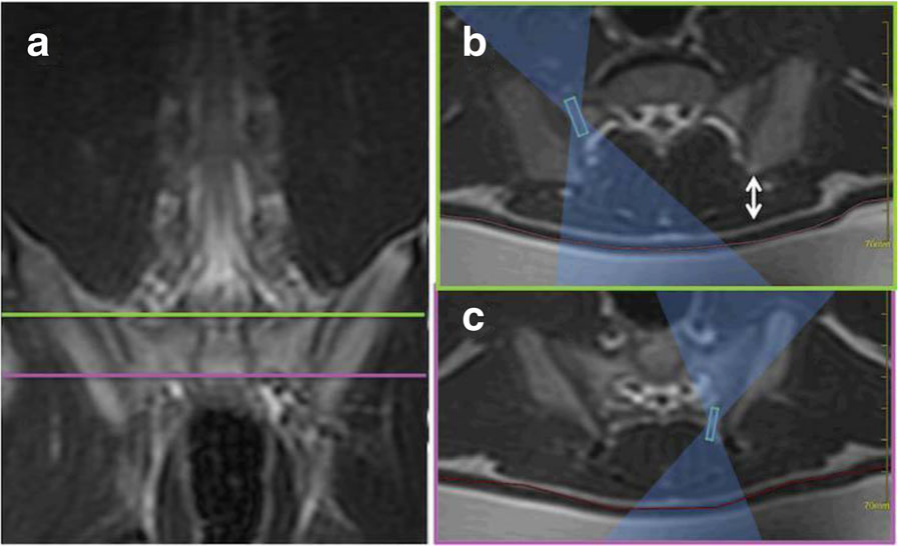
Example of planning MR images showing the SIJ in coronal and axial planes. **a** Green and magenta lines indicate the most superior (*green*) and inferior (*magenta*) slices where the sonications were prescribed as shown in (**b**) and (**c**). White arrow points out the short distance between the bone and the skin

Three swine (weight range 30–35 kg) underwent bilateral MRgHIFU ablation (ExAblate 2000; InSightec Ltd., 3 Tesla MRI) of the SIJ in the supine position. All procedures were carried out according to an approved Institutional Animal Care and Use Committee protocol. To create a thermal lesion cutting across the lateral branches, traveling from intervertebral and sacral foramen to the SIJ, individual sonications were stacked along the SIJ slightly medial to the joint from superior to inferior points as shown in Fig. 121. The beam angle was adjusted to achieve near-perpendicular incidence on the sacral bone or SIJ, and was angled away from the spine to avoid ablation of the sacral nerve roots. To efficiently interrupt the lateral branches, the diameter of which ranges from 0.2 to 1.5 mm [3], both near-field heating (right side, Fig. 121b) and direct heating (left side, Fig. 121c) approaches were applied. The resonant frequency was set to 1.35 MHz, sonication duration was 20 s. In the first experiment, 42 sonications with acoustic energy of 840–1300 J were applied using 90 s cooling time. These parameters were adjusted after the first exploratory experiment. In the subsequent two animals, the energy was lowered to 574–800 J, the number of sonications was reduced to 15, and the cooling time was increased from to 120–150 s. Thermometry imaging was performed in the axial plane in animal 1, and in sagittal plane in animals 2 and 3. Contrast enhanced (CE) imaging was performed immediately following treatment with MRI and 48 hours after treatment with CT. During those 48 hours, animals were maintained on preventative medications for potential pain. Veterinary staff performed assessment of pain and animal behavior twice a day to monitor for any unwanted side effects such as deep pain or impaired ambulation. The animals were euthanized with an IV injection of pentobarbital (100mg/kg) while under anaesthesia at the end of the CT scan. At necropsy, the sacrum (including the SIJ) was removed and fixed in 10% neutral buffered formalin, followed by decalcification. The SIJ was then sectioned transversely and analysed using hematoxylin and eosin (H&E) stain.

**Results**

**Fig. 122 (abstract O86).**
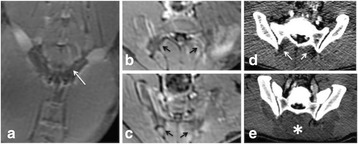
Example of CE-MR images obtained immediately following MR-HIFU treatment and follow-up CE-CT images. Lack of contrast uptake indicates the ablated regions (*arrows*). **a** Coronal image showing the continuous regions of ablation along the SIJs. **b**-**c** Axial CE-MR images of the most superior and inferior slices of the bilateral lesions. Axial CE-CT images showing a successful ablation confined to the SIJ and adjacent tissue (**d**), and unwanted necrosis of a large volume of muscle and skin (asterisk), which resulted from potentially insufficient cooling time between consecutive sonications (**e**)

**Fig. 123 (abstract O86).**
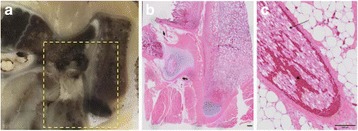
**a** Example of gross pathology section of the SIJ showing discoloration of muscle and bone tissue consistent with thermal changes. **b** Low magnification H&E histology of a boxed section in (**a**). Muscle, adjacent bone tissue, and two nerves (*black arrows*), show changes consistent with thermal necrosis. 1 mm scalebar. (**c**) High magnification H&E histology image of the nerve showing hemorrhage (asterisk), and loss of axons associated with dilation of the myelin sheath (*arrow*). 100 um scalebar

All treatments resulted in non-perfused regions surrounding the SIJ medial surface visible on CE-MR (Fig. 122a-c) and CT 48-hour follow-up images (Fig. 122d-e). Continuous non-perfused regions of ablation were achieved along the SIJ in animals 2 and 3 (Fig. 122a-c). In the first experiment, continuous muscle necrosis extended from the SIJ to the skin (Fig. 122e) and skin discoloration consistent with the burn approximately 10 mm in diameter was found. There were no skin burns or unwanted muscle necrosis in the other two animals. Gross pathology analysis showed discoloration of muscle and the surface layer of adjacent bone, as is associated with hemorrhage (Fig. 123a). Histological analysis (Fig. 123b-c) confirmed necrosis of muscle and adjacent bone, and thermal destruction of the nerves manifested as hyperemia of endoneurial vessels, and loss of axons associated with dilation of the myelin sheath. Neither superficial nor deep pain, nor changes in animals’ ambulation nor behaviour were reported by the veterinary stuff.

**Conclusions**

The present study shows that the lateral branch nerves innervating the SIJ can be successfully ablated using MRgHIFU by creating a continuous thermal lesion along the SIJs. Due to the overall short distance separating the surface of the sacral and iliac bones from the skin, and the previously reported slower cooling of tissue adjacent to cortical bone [4], sufficient cooling time needs to be allowed between the sonications to minimize excessive damage of adjacent muscle. Additionally, due to the “double-oblique” surface of the SIJ and the necessary angulation of the beam in right-left and superior-inferior planes, thermal imaging in the sagittal plane may better capture the largest temperature rise during a sonication compared to the axial plane imaging. In conclusion, this study provides preliminary evidence of feasibility of a novel, and potentially more effective, treatment technique for denervation of the SIJ in humans. Further study is needed to accurately determine if pain improvement can be achieved clinically.

**References**

[1] Cohen SP. Sacroiliac joint pain: a comprehensive review of anatomy, diagnosis, and treatment. Anesthesia & Analgesia. 2005;101(5):1440–53.

[2] Yin W, Willard F, Carreiro J, Dreyfuss P. Sensory stimulation-guided sacroiliac joint radiofrequency neurotomy: technique based on neuroanatomy of the dorsal sacral plexus. Spine. 2003;28(20):2419–25.

[3] Roberts SL, Burnham RS, Ravichandiran K, Agur AM, Loh EY. Cadaveric study of sacroiliac joint innervation: implications for diagnostic blocks and radiofrequency ablation. Regional anesthesia and pain medicine. 2014;39(6):456–64.

[4] Webb TD, Bitton RR, Ghanouni P, Butts Pauly K, editors. Spatial and temporal characteristics of soft tissue heating in MRgHIFU treatment of bone metastasis. ISMRM; 2015; Toronto.

### O87 Trans-fusimo - an integrative approach to model based treatment planning and conducting of FUS in moving abdominal organs

#### Tobias Preusser^1,2^, Sabrina Haase^1^, Mario Bezzi^3^, Jürgen W. Jenne^10^, Thomas Lango^4^, Yoav Levy^5^, Michael Müller^6^, Giora Sat^11^, Christine Tanner^7^, Stephan Zangos^8^, Matthias Günther^1^, Andreas Melzer^9^

##### ^1^Fraunhofer MEVIS, Bremen, Germany; ^2^Jacobs University, Bremen, Germany; ^3^Universita Degli Studi Di Roma La Sapienza, Rome, Italy; ^4^Stiftelsen SINTEF, Trondheim, Norway; ^5^InSightec, Haifa, Israel; ^6^IBSmm, Brno, Czech Republic; ^7^ETH, Zurich, Switzerland; ^8^Johann Wolfgang Goethe University, Frankfurt, Germany; ^9^IMSat, University of Dundee, Dundee, United Kingdom; ^10^Mediri GmbH, Heidelberg, Germany; ^11^GE Medical Systems, Haifa, Israel

**Objectives**

Treating liver tumours using FUS poses a great challenge due to the breathing motion of the target and the occlusion of the anatomical location of the malignancy by the rib cage. To perform safe, effective and efficient ablation of tumours, a thorough planning as well as precisely conducting of the treatment is essential. The EU projects FUSIMO (2011–2015) and TRANS-FUSIMO (www.trans-fusimo.eu) aim at the development and clinical translation of a sophisticated software system that supports the exploration of the full potential of extracorporeal FUS in the planning and execution stage of the treatment.

**Methods**

The enabling technology of the TRANS-FUSIMO software system is a set of dynamic organ models for the physical and biophysical processes involved in FUS treatment: (i) an abdominal motion model simulates the patient specific deformation of the organ and relevant anatomical structures during breathing; (ii) a patient specific tissue model represents the ultrasound propagation, the energy deposition as well as the tissue heating and cooling; (iii) an organ/tumour model captures the patient specific tissue’s response to the therapy. These model components are parameterized with patient specific data that is extracted from patient specific pre-interventional imaging data like MRI and/or US imaging data. Moreover, in TRANS-FUSIMO, the abdominal motion model is used with real-time MR and/or US data to track the motion of the target and steer the FUS beam accordingly.

**Results**

The model components are capable of simulating the patient specific motion of the organ under breathing, as well as the propagation of ultrasound, the diffusion of heat and the influence of blood perfusion in the FUSIMO planning system. The treatment system using the abdominal motion model and the other model components are being validated in phantom and ex vivo experiments as well as in Thiel soft embalmed human cadavers. Furthermore, in TRANS-FUSIMO, the safety, efficacy and efficiency of the software assistant will be evaluated in an in-vivo animal study. Moreover, a two-arm study (neoadjuvant TRANS-FUSIMO MRgFUS + resection, TRANS-FUSIMO MRgFUS only) for human patients with metastases or HCC will show the feasibility of the TRANS-FUSIMO treatment system for the clinical setting. The talk will give an update on the current progress of the project.

**Conclusions**

The FUSIMO software demonstrator comprises patient specific models for the simulation of FUS application in moving organs. It supports the assessment of the feasibility of the intervention, predicting and optimizing the outcome, detecting potential risks and avoiding them, as well as monitoring the progress and tracking deviations from the planned procedure. Our ex-vivo experiments show that the FUSIMO system is capable of compensating organ motion through real-time motion detection, motion modelling and real-time beam steering. In TRANS-FUSIMO a fully integrated system is being developed for which in-vivo animal studies and first patient study shall show that MRgFUS in moving organs can be performed safely, efficaciously and effectively.

**Acknowledgements**

*The research leading to these results has received funding from the European Union's Seventh Framework Programme (FP7/2007-2013) under grant agreements no. 270186 (FUSIMO) and no. 611889 (TRANS-FUSIMO).*

### O88 Guidance and assessment of HIFU transrectal treatment of prostate cancer

#### Cyril Lafon^1,2^, Au Hoang Dinh^1,2^, Emilie Niaf^3,1^, flavie bratan^5^, Nicolas Guillen^4^, Rémi Souchon^1,2^, Carole Lartizien^3^, sebastien crouzet^5^, olivier rouviere^1,5^, Jean-Yves Chapelon^1,2^

##### ^1^LabTAU, INSERM, Lyon, France; ^2^University of Lyon, Lyon, France; ^3^CREATIS, Lyon, France; ^4^EDAP-TMS, Vaux-en-Velin, France; ^5^Hospices Civils de Lyon, Lyon, France

**Objectives**

Prostate cancer is the most frequently diagnosed non-skin cancer in the United States and the third leading cause of cancer deaths. Recent progresses in the diagnosis of prostate cancer open the gate to focal therapy. New HIFU devices have been specifically designed for that purpose. They combine the necessary tools to visualize, target, treat and validate the focal treatment. The goal of the presentation is to describe some of these developments made for improving the cancer diagnosis, the guidance of the treatment and the assessment of its efficacy.

**Methods**

A computer-assisted diagnosis (CAD) system was developed for determining a likelihood measure of prostate cancer presence in the peripheral zone (PZ) based on multiparametric magnetic resonance (MR) imaging. The evaluation database consisted of 30 sets of multiparametric MR images acquired from radical prostatectomy patients. Both cancer and non-malignant (but suspicious) tissues were annotated in consensus on all MR images by four experts. Histologic sections were considered as the gold standard.

Focal treatments by HIFU were performed with Focal One® (EDAP-TMS, Lyon France). The process can be divided in 4 steps: treatment preparation with import of MR images and fusion with the real-time ultrasound volume, focal target definition, HIFU delivery and validation of effectively de-vascularized volume with contrast-enhanced ultrasound (CEUS). Pretreatment MR images with contoured prostate and annotated suspect ROI can be imported in the HIFU device. The urologist contours similarly the prostate on the live ultrasound images acquired with the transrectal probe. The software automatically registers the two volumes and proceeds to an “elastic fusion”. For treatment guidance, the same 3D elastic transformation is applied to the ROIs initially indicated on the MR image so they appear at the adequate position on the ultrasound Image.

For monitoring purpose and assessing the treated volume, a systematic CEUS with SonoVue TM is performed at the end of the procedure. A treatment completion can be performed if necessary in the same HIFU session.

**Results**

For the CAD, a restrictive set of about 15 highly informative features coming from all MR sequences was discriminated, thus confirming the validity of the multiparametric approach. Quantitative evaluation of the diagnostic performance yielded a maximal area under the ROC curve (AUC) of 0.89 (0.81-0.94) for the discrimination of the malignant versus nonmalignant tissues and 0.82 (0.73-0.90) for the discrimination of the malignant versus suspicious tissues.

A feasibility study was performed on 10 patients focally treated with Focal One to evaluate the precision and the efficacy of the focal therapy. Concerning the precision of the treatment, control CEUS-guided biopsies inside and around the necrotic zone at one month showed 100% negative biopsies in the treated area and viable tissue immediately around the treated area. For the monitoring, this feasibility study demonstrated that the post-operative necrosis observed on early MRI (2–7 days after HIFU) corresponded to the devascularized area observed on CEUS at the end of the HIFU procedure. Urinary continence and erectile function were fully preserved for the 10 patients.

**Conclusions**

Last generation of transrectal HIFU represents an interesting option for focal treatment of prostate cancer, combining imaging techniques, treatment preciseness and treatment validation. The cancer must be localized precisely and CAD based on multiparametric magnetic resonance (MR) imaging can be helpful. The developed CAD assists both non-experts and expert uro-radiologists in the detection of aggressive cancerous foci in the PZ. This development is particularly interesting in the context of overtreatment of prostate cancer. The MRI/US fusion facilitates the targeting of the localized prostate cancer and allows a precise and efficient focal treatment. Post-HIFU CEUS is demonstrated to be a good method for evaluating the quality of the treatment.

### O89 Real-time monitoring of high-intensity focused ultrasound treatment using harmonic motion imaging for focused ultrasound

#### Yang Han^1^, Shutao Wang^1^, Elisa E. Konofagou^1,2^

##### ^1^Department of Biomedical Engineering, Columbia University, New York, NY, United States; ^2^Department of Radiology, Columbia University, New York, NY, United States

**Objectives**

Harmonic Motion Imaging for Focused Ultrasound (HMIFU) has been shown to image and monitor HIFU ablation based on the stiffness change of the tissue. In this study, the feasibility of HMI for real-time monitoring and the formation of the lesion during HIFU treatment is explored.

**Methods**

The HMIFU setup consists of a 93-element, 4.5-MHz HIFU transducer confocally aligned with a 64-element 2.5-MHz phased array to transmit and receive through a 4-board VDAS system. All HIFU channels were synchronously excited by a 50 Hz amplitude modulation to vibrate the tissue at 100 Hz. In vitro experiments were performed on 9 canine liver specimens. The acoustic power and ablation duration was 20 W and 90 s respectively. Peak-to-peak HMI displacement map were calculated. The displacement map at 1s was used as a reference frame to subtract the following frames during ablation. The last frame of ablation subtracted from the reference frame was used to quantify the size of HMIFU lesion. The dimension of HMIFU lesions were compared to the gross pathology. Both depth and width of the lesions were compared between lesion-segmented images obtained for both techniques.

**Results**

The average peak-to-peak displacement in the ROI of canine liver before ablation was found to equal 16.32±3.02 μm. After ablation, the average displacement of canine liver after ablation was decreased to 3.84±1.94 μm. The figure shows representative HMI displacement contrast maps in canine liver during ablation and a photography of the gross pathology section after ablation. Yellow indicates the area where displacement decreasing during ablation. The depth and width of the 5 lesions’ measured by HMI were equal to 10.9 ±3.4 mm and 8.4 ±2.9 mm respectively while depth of 11.5 ±2.4 mm and width of 9.8 ±1.5 mm was found in gross pathology. Good agreement between the lesion’s depths and widths determined with gross pathology and HMI. HMIFU can successfully monitor thermal lesions development in *in vitro* canine liver specimens.

**Conclusions**

HMIFU can successfully monitor thermal lesions development in *in vitro* canine liver specimens.

*Supported by R01EB014496-01.*

**Fig. 124 (abstract O89).**
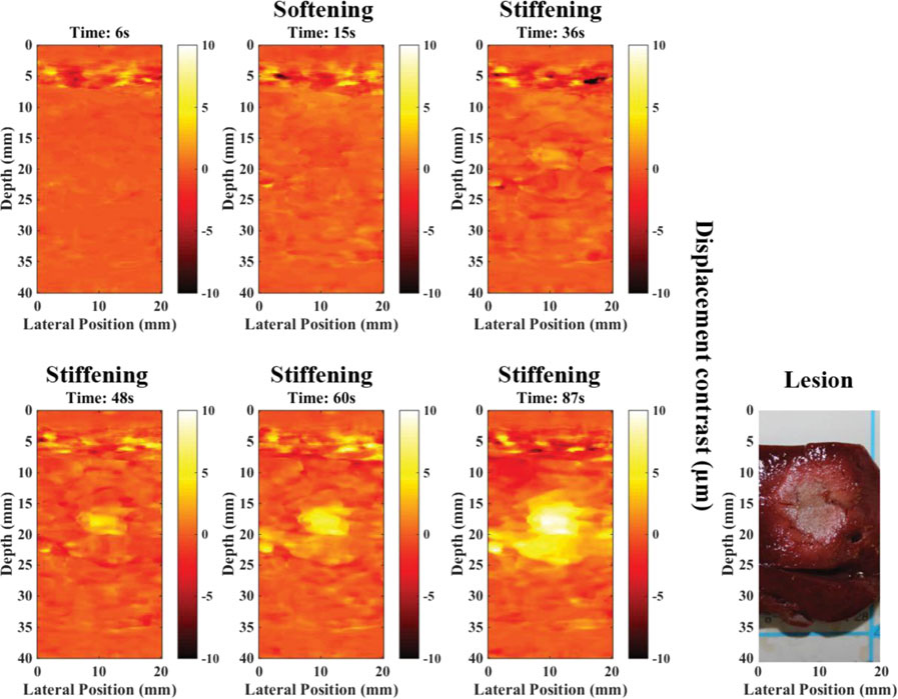
HMI displacement contrast maps in canine liver during ablation and a photography of the gross pathology section after ablation (*down right*)

### O90 Harmonic motion imaging for focused ultrasound (HMIFU) for pancreatic tumour monitoring and treatment in a transgenic mouse model

#### Thomas Payen^1^, Carmine Palermo^2^, Steve Sastra^2^, Hong Chen^1^, Yang Han^1^, Kenneth Olive^2^, Elisa E. Konofagou^1,3^

##### ^1^Biomedical Engineering, Columbia University, New York, NY, United States; ^2^Herbert Irving Comprehensive, Cancer Center, Columbia University, New York, NY, United States; ^3^Radiology, Columbia University, New York, NY, United States

**Objectives**

Harmonic Motion Imaging (HMI) assesses tissue mechanical properties by inducing localized oscillation resulting from a periodic acoustic radiation force. The amplitude of the induced displacement is directly related to the underlying tissue stiffness. The sonication can be short for imaging (HMI) or prolonged for simultaneous HMI and HIFU treatment (Harmonic Motion Imaging for Focused Ultrasound or HMIFU).

The objective of this study was to assess a murine pancreatic tumour model during its growth in terms of elasticity with HMI, then treat it using HMIFU. HMI measurements are then resumed to monitor the mechanical changes resulting from the treatment.

**Methods**

A 4.5-MHz focused ultrasound transducer (FUS) generated an amplitude-modulated beam resulting in harmonic tissue oscillations at its focus. Axial tissue displacement was estimated using 1D cross-correlation of RF signals acquired with a confocally aligned, 7.8-MHz diagnostic transducer (P12-5, ATL) using a plane-wave beam sequence at a framerate of 1 kHz. Pancreatic tumour growth was monitored using HMI in a transgenic mouse model to acquire elasticity maps with 0.2 s sonication for each scan position. When the tumour reached a size of approximately 5 mm, HMIFU was performed over a period up to 120 s, which was shown to generate a lesion in this model according to previous work by our group (Chen et al., IEEE TUFFC, 2015). The success of the treatment was assessed by measuring both the tumour area and its elasticity up to 14 days with regular histological measurements.

**Results**

**Fig. 125 (abstract O90).**
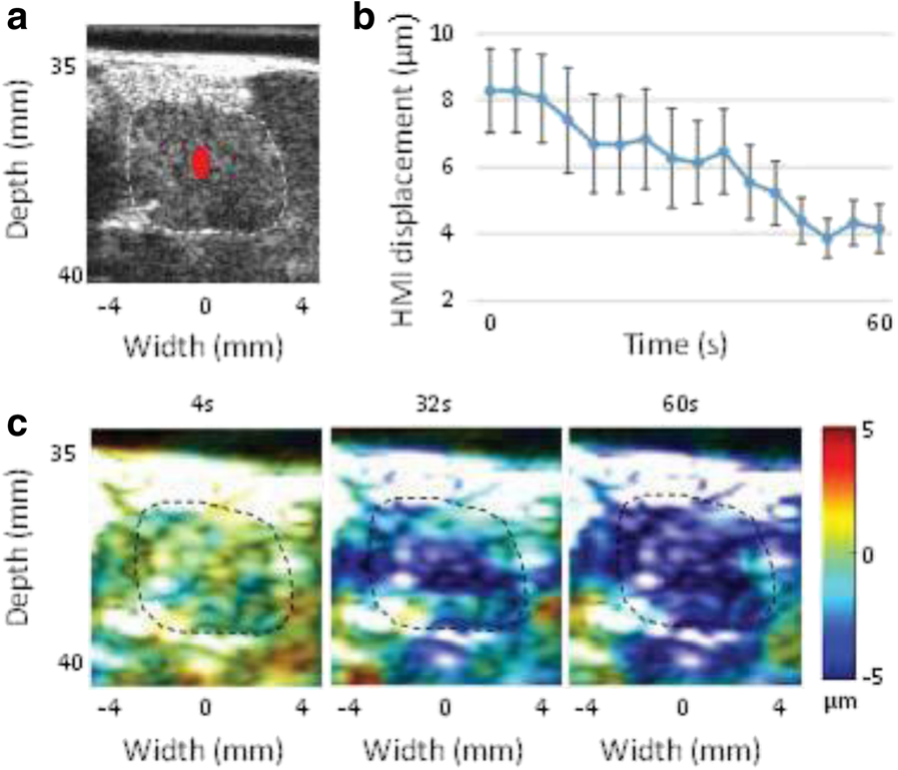
HMIFU results in a murine pancreatic tumour model. The tumour is delineated on the Bmode image (**a**) with the FUS focal shown in red. The mean HMI displacement in the tumour during ablation is shown in (**b**). Corresponding HMI maps during with values relative to the baseline at t = 0 are shown for t = 4 s, 32 s and 60 s (**c**). The decrease reflects the progressive stiffening of the ablated tissue

HMI demonstrated its capability to provide reproducible elasticity measurements in murine pancreatic tumours. Measurements show that stiffening occurs progressively during pancreatic tumour growth from the very early stages. When plotting the HMI displacement against the tumour size, an exponential trend was fitted to the data with R^2^ > 0.7. During ablation with HMIFU, the tumour was shown to stiffen (Fig. 125). After 60 s of treatment, a 51.4% decrease in HMI displacement was observed compared to baseline value at t = 0 (Fig. 125b and c). The lesion was confirmed by histology. The follow-up of the HMIFU treatment is performed with HMI to assess long-term tissue mechanical changes after treatment.

**Conclusions**

This work shows that the HMI technique can provide elasticity measurements in the murine pancreatic tumour during its growth with HMI as well as treat the tumour with simultaneous monitoring using HMIFU. The follow-up was performed with HMI to assess post-treatment mechanical changes. This study underlines the potential of HMI for monitoring tumour growth, treatment and follow-up changes in elasticity.

### O91 Magnetic resonance acoustic radiation force imaging for interventional planning of HIFU therapy in the kidney

#### Johanna M. van Breugel^2^, Martijn de Greef^2^, Charles Mougenot^3^, Maurice A. van den Bosch^1^, Chrit Moonen^2^, Mario Ries^2^

##### ^1^Radiology, University Medical Center Utrecht, Utrecht, Netherlands; ^2^Imaging department, University Medical Center Utrecht, Utrecht, Netherlands; ^3^Philips, Ontario, Ontario, Canada

**Objectives**

The precision of MRI based interventional guidance of High-Intensity Focused Ultrasound relies i.a. on the stereotactic correspondence between the MR-image and the location of the HIFU ablation area. However, incongruities between the planned focal point location and the actual focal position of the HIFU beam are routinely observed, predominantly due to acoustic wave-front aberrations induced by dissimilarities of the acoustic impedance in the beam propagation path. The current solution in clinical practice is to employ a “low power test shot” before the start of the ablation of particular region to provide a 3D correction of the precise focus location. The low power generally allows observing a distinct temperature elevation without causing irreversible tissue damage. In spite of being safe and accurate in low perfused target tissues such as uterine fibroids, the high heat extraction rate renders this approach of limited value in high-perfused organs such as liver or kidney. Moreover, further discrepancy is added between a linear test shot and a therapeutic sonication for therapeutic HIFU sonications in the non-linear pressure range [1].

Recently, MR-acoustic radiation force imaging (MR-ARFI) has been proposed as an alternative method to locate the focal point non-invasively without giving rise to non-desired tissue damage [2–5]. MR-ARFI evidences the tissue displacement due to the acoustic radiation force of the HIFU beam instead of the temperature. However, since MR-ARFI employs a highly motion sensitized sequence and displays limited sensitivity, it is challenging in abdominal organs and *in vivo* experience with this type of this approach is currently limited to the liver.

The first aim of this study was to investigate the feasibility of respiratory-gated MR-ARFI on clinical HIFU equipment for the exact beam localization in the kidney. Of particular interest was hereby potential interference with other motion sources, such as cardiac pulsations in the vicinity of larger vessels such as the renal artery. The second goal was to validate if MR-ARFI can perform this task for both linear and non-linear acoustic energy delivery.

**Methods**

A porcine model (85 kg) was chosen for its similarities in size and perfusion of the kidney to the human kidney. The local animal welfare committee approved the study The pig was anesthetized and mechanically ventilated at a frequency of 13/min. A clinical Philips Sonalleve MR-HIFU therapy system (Philips Healthcare, Finland) integrated with a 1.5T Achieva MRI (Philips Healthcare, The Netherlands) with minor modifications was used for MR-HIFU. An MR-HIFU treatment cell was positioned at 4.5cm from the skin in the cortex of the kidney. A gradient-recalled echo planar imaging 2D dynamic scan (TR 100ms, TE 30ms, flip angle 20°, FOV 168x168mm^2^, matrix 112x108, voxel size 1.51x1.51mm^2^, slice thickness 7mm) was performed to visualize displacement due to the acoustic radiation force similar to [2]. Thermometry data was obtained using a gradient echo with echo planar imaging (TR 100ms, TE 15ms, flip angle 20°, matrix 160x160, FOV 400x400mm^2^, voxel size 2.5x2.5mm^2^, slice thickness 5mm). All MR sequences, including anatomical images, and the acoustic energy delivery were respiratory gated based on pencil beam navigator images along the transition of the contralateral kidney to the perinephric fatty tissue. For comparison of linear and non-linear effects, four experiments at two different power levels were performed: Two thermal ablations with 450 W continuous wave and 1000 W pulsed (30% duty cycle, tone-burst of 10500 cycles), and two MR-ARFI experiments at equivalent acoustic intensity (tone-burst of 2400 cycles, bipolar MR-ARFI gradient 20mT/m, 3ms).

**Results**

**Fig. 126 (abstract O91).**
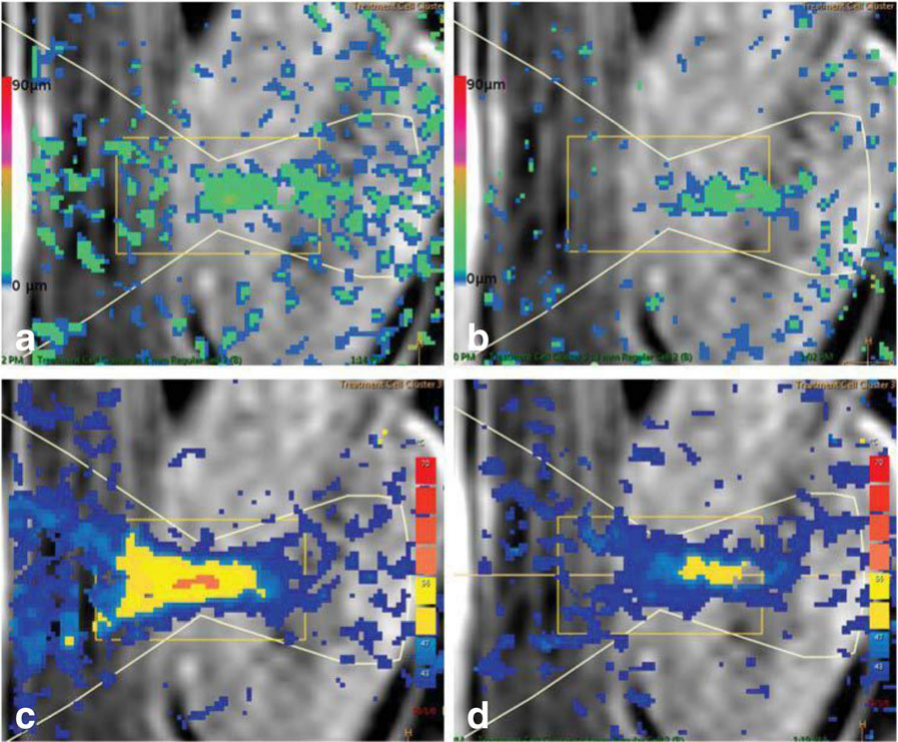
**a** and **b** show displacement maps after MR-ARFI at 450W and 1000W, respectively. **c** and **d** show temperature maps af ablations at 450W and 1000W. All images are overlayed on anatomical T_1_-weighted MR images. ARFI-maps and temperature maps coincide when equivalent power levels are used. Note the spacial shift between the 450W and 1000W experiments

Displacements due to the radiation force could be reproducibly measured for both acoustic intensities and coincided with the location of the temperature elevation of the equivalent 450W and 1000W ablations, respectively (Fig. 126). No interference/artifacts with the cardiac cycle were observed during MR-ARFI. The focal point of the beam cone was evidenced more posteriorly than planned for the 1000 W, most likely due to shock-wave formation and non-linear absorption. No significant heating was observed during the MR-ARFI experiments (<2°C).

**Conclusions**

The employed respiratory gated MR-ARFI sequence in combination with a 450 W excitation tone-burst of 2400 cycles is sensitive enough to exceed the noise level and to clearly display the focal point of the HIFU beam at a fraction of the energy compared to the 450W thermal ablation. Both at 450W and at 1000W the displacement due to the radiation force coincided with the location of the temperature rise due to thermal ablation at equivalent power. Hence, radiation force in combination with a pencil beam navigator to compensate for respiratory motion is a reliable indicator of the location of the thermal lesion and might be an alternative to the low power thermal test shot in highly perfused organs such as the kidney.

### O92 Magnetic resonance acoustic radiation force imaging for in vivo estimation of ultrasonic transmission factor through rat skulls

#### Matthieu Gerstenmayer, Rémi Magnin, Benjamin Fellah, Denis Le Bihan, Benoit Larrat

##### NeuroSpin, CEA Saclay, Gif-sur-Yvette, France

**Objectives**

When performing transcranial focused ultrasound procedures, the knowledge of the *in situ* acoustic pressure is key in order to ensure both efficacy and safety. This is particularly true for blood–brain barrier (BBB) disruption studies where only a narrow range of peak negative pressure (PNP) is acceptable, sufficient stable cavitation of the microbubbles without reaching inertial cavitation responsible for irreversible damages.

More specifically, the literature is missing data on rat transmission factors [O’Reilly et al. *Ultrasound Med Biol.* 2012] while this animal model is widely used for HIFU in brain [Dervishi et al. *Int. J. Hyperthermia.* 2013]. The transmission factor seems to depend on both age and skull location. This makes it difficult to compare studies performed on rats of different weights and - more concerning - it could bias some of the PNP values published in the literature. It is therefore useful to provide more *ex vivo* data. However, the real grail would be to be able to measure this transmission factor (or directly the PNP) *in vivo*. To do so, magnetic resonance acoustic radiation force imaging seems a promising technique [Larrat et al. *Phys Med Biol.* 2010] since it is based on the MR encoding of displacements induced by the ultrasound beam at focus which are quantitatively related to acoustic intensity.

In this work, we present new *in vitro* measurements through rat skulls that go beyond already available data and we introduce a way to use MR-ARFI for *in situ* acoustic calibration in the rat brain. Firstly, this coefficient was measured *ex vivo*, in a water tank, for rats of different ages and for different positions of the focal acoustic spot in the skull and different frequency. Secondly, the transmission factor was estimated *in vivo*, under MRI, thanks to an Acoustic Radiation Force Imaging (ARFI) sequence.

**Methods**

Prior to all studies, an 8 channels concave annular array transducer (Imasonic) was first calibrated in open field in a water tank. A calibrated hydrophone was used to measure the peak negative pressure of the transducer at focus as well as to measure the size of the focal spot.

Then, in a first study, the ultrasonic transmission factors through rat skulls were measured as a function of animal weight and ultrasound frequency. These measurements were performed with the ultrasound beam intersecting the skulls at three different locations in the head-foot direction: front, middle and back, for differrent frequencies: 1, 1.25, 1.5, 1.5 and 2 MHz. The skulls of 11 Sprague–Dawley rats (Janvier, France) of different weights (100–500 g) were used. The acoustic transmission factor was then measured as the ratio between the PNP at the focal spot in the presence and in the absence of skull.

In a second study, 4 rats were installed in our motorized MR guided transcranial focused ultrasound system (Image Guided Therapy and Bruker) using the same transducer as previously. MR-ARFI signals are acquired at 38 different locations in the brain separated by 1 mm so that to cover one whole skull hemisphere. Under our imaging conditions, the ARFI phase difference between the center of the focal spot and the background can be assumed to be proportional to the acoustic intensity. Therefore, the relative transmission factor through the skull is proportional to the square root of the ARFI phase difference. After sacrifice of the animals, the transmission factors through the same hemisphere were mapped in a water tank at high spatial resolution (millimeter by millimeter).

**Results**

**Fig. 127 (abstract O92).**
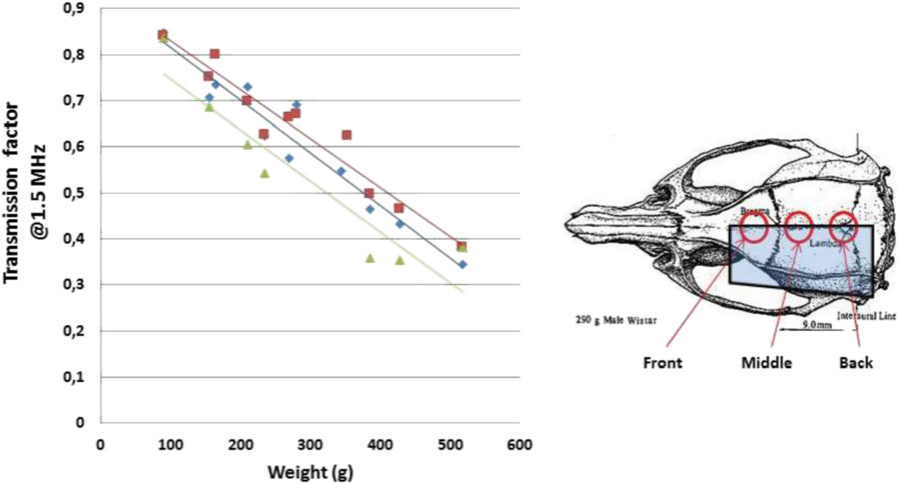
*Left*: acoustic transmission factor, in pressure, of the rats’ skull as a function of animal weight. Measurement done at 1.5 MHz and at the three positions: back (*green*), middle (*blue*), front (*red*), as drawn with red circles on the skull. *Right*: the three positions on the skulls

The main result of the first study is the almost linear dependence of the transmission factors on the animal weights (see Fig. 127) at all frequencies. This is explained by the fact that the heavier animals are older and have thicker skulls. Our measurements also show that the skull is not homogeneous in the head-foot direction, the front and middle parts transmitting less than the back. Finally, as expected, the transmission factors decrease with increasing ultrasound frequency.

**Fig. 128 (abstract O92).**
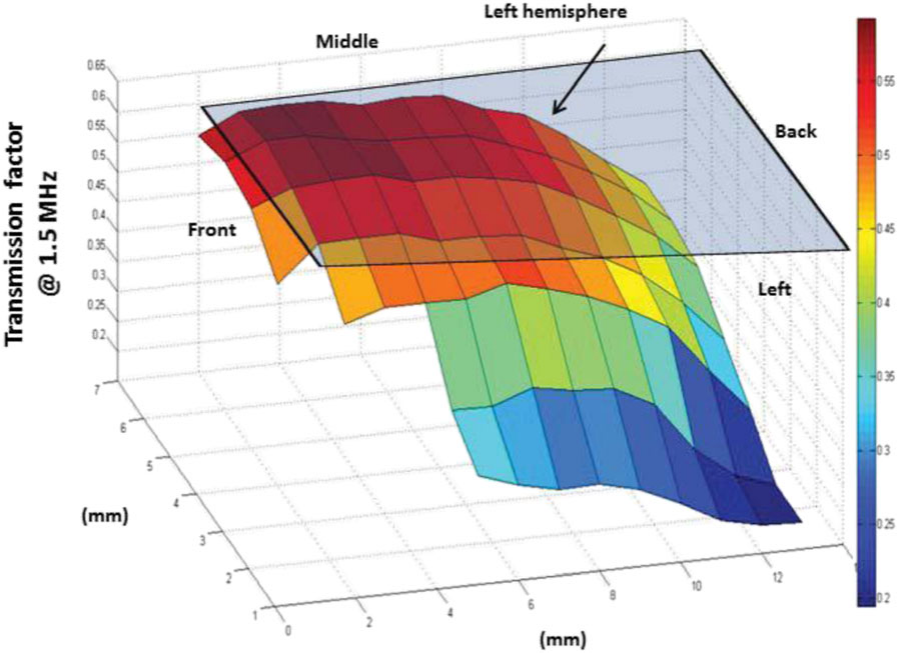
Acoustic transmission factor mapping, in pressure, of the left half skull of a 320 g rat. The corresponding region is drawn as a black rectangle on Fig. 127

The full mapping of left skull hemispheres (see Fig. 128) confirms the strong variations in the head-foot direction but it also shows that the transmission factor dramatically drops on the sides of the skulls.

**Fig. 129 (abstract O92).**
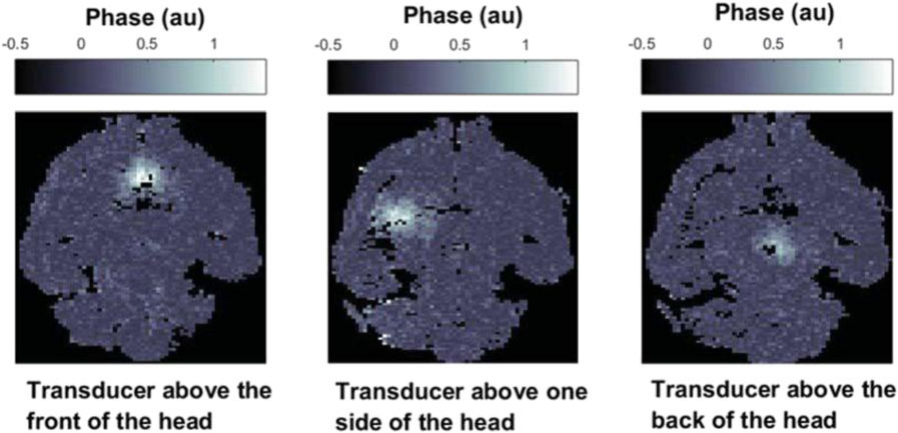
Example ARFI images for three positions of the transducer on top of the head showing that the transmission factor decreases on the back and on the sides

The best way to remove background phase on the ARFI images was proved to be the following: mask the brain excluding the focal spot, find the best 2D polynomial (order 5 in x and y directions) surface that fits the background and then remove this modelled background. Qualitatively, the ARFI signals (see Fig. 129) is in agreement with the one in the water tank: the transmission factor is maximum in the middle and falls on skull sides. The encoded displacement of the media due to the radiation force depends on the viscoelastic behavior of this media, so for a given PNP, ARFI signals will differ between two regions if the viscoelastic response is too different. This is particularly true in ventricles where water can flow easily. To take this into account, a more refined data analysis was developed. High resolution T_2w_ images are segmented into 3 compartments of known elasticity and viscosity: grey matter, white matter and cerebro-spinal fluid. The Green function and the MR-ARFI encoding are simulated at each ARFI location to recover the estimated acoustic pressure [Larrat et al. *Phys Med Biol.* 2010, Bercoff et al. *IEEE Trans UFFC.* 2004]. This new analysis is still on going.

**Conclusions**

Those results are very interesting in order to gain control in the transcranial FUS experiments in rats. The transmission factor varies a lot between small and big rats. The power delivered to the transducer has to be tuned to take this into account. This is particularly relevant in longitudinal studies where the animals can grow significantly.

The “high resolution” mapping of acoustic transmission shows a strong dependence on the part of skull that is intersected by the beam. This knowledge allows to correct the power to ensure uniform pressure distribution along motorized trajectory such as the one performed for our BBB openings protocols. More power has to be delivered to the back and lateral parts of the brain.

The ARFI *in vivo* estimation technique is promising in order to calibrate the beam *in situ*. However, from our preliminary results, it is clear that one has to take viscoelasticity into account to get reliable quantitative pressure estimations.

### O93 Relationship between acoustic pressure used for BBB disruption and amount of MRI contrast agent delivered to the brain

#### Matthieu Gerstenmayer, Rémi Magnin, Sébastien Mériaux, Denis Le Bihan, Benoit Larrat

##### NeuroSpin, CEA Saclay, Gif sur Yvette, France

**Objectives**

The acoustic intensity needs to be very well known to efficiently open the Blood–brain Barrier (BBB) since it is mainly ruled by the mechanical index [1]. Previous studies have shown that a narrow range of peak negative pressure (PNP) is acceptable, corresponding to sufficient stable cavitation of injected microbubbles without reaching inertial cavitation responsible for irreversible damages. Within this range, only few data are available about how the pressure affects the strength of BBB disruption that is to say the amount of delivered drugs.

We propose two new methods to evidence the opening threshold in vivo and measure this relationship after a MRI controlled protocol of the BBB opening: 1) Several different pressures are used at several brain locations during the same BBB opening session 2) A single point focal opening is performed and the brain uptake of contrast agent is correlated in each voxel with the pressure estimated from a MR-acoustic radiation force image [2].

**Methods**

We use our previously introduced motorized MR-compatible FUS system [3] (Image Guided Therapy, France). An 8 channel annular array transducer operating at 1.5MHz (Imasonic, France) was calibrated in a water tank and acoustic transmission factors through rat skulls were measured in another study. This ability to shrewdly control the pressure we deliver *in vivo* in the brain is crucial for the two proposed methods. Fisher F344 rats (n=5, body weight=220g) were shaved and installed in the MRI bed of a 7T MRI scanner (Bruker) under isoflurane anesthesia. The transducer was coupled to the head with echographic gel.

In a first experiment, after intravenous injection of microbubbles (Sonovue), a square trajectory was played with the transducer continuously shooting. 4 different pressures (0.25 0.35 0.45 0.55 MPa) were applied on the 4 sides of the square. The transducer was moving at 10 mm/s, the size of the square was 5 mm and the trajectory was played 60 times. Then a 200μL Gadolinium based MRI contrast agent (Dotarem, Guerbet, France) was intravenously injected, at different time after the opening (10 minutes, 4 hours and 24 hours), and its uptake into the brain was quantitatively measured thank to T_1_ maps.

In a second experiment, the BBB was focally open with 0.6 MPa peak negative pressure at focus. Before the opening session, an Acoustic Radiation Force Image was acquired in order to estimate *in vivo* the pressure applied in each voxel during the BBB opening. After the opening, Dotarem was intravenously injected and the contrast agent concentration was measured in each voxel by acquiring T_1_ maps [4]. The concentration in each voxel is then correlated to the pressure which has been used in this voxel to open the BBB.

**Results**

**Fig. 130 (abstract O93).**
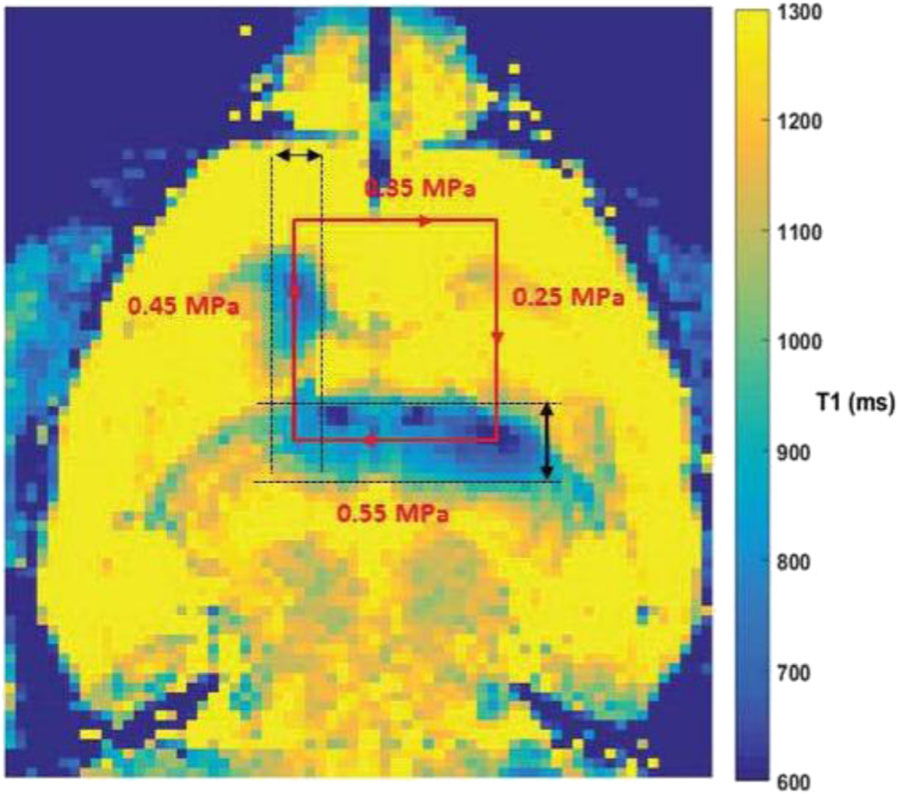
T1 map after the square trajectory, acquired 26 minutes after the opening of the BBB. The focal spot of the ultrasound was moved along the red lines. The dot black lines indicates the width of the opening for the two open sides

The first experiment with the square trajectory gives a rough estimation of the pressure threshold. Indeed it appears clearly (see Fig. 130) that the BBB is not open on the 0.25 and 0.35 MPa sides while it is open on the 0.45 and 0.55 MPa sides. The acoustic threshold must be between 0.35 and 0.45 MPa. With the second injection, we also found that the BBB was already close as soon as 4 hours after the opening even for the 0.55 MPa region.

**Fig. 131 (abstract O93).**
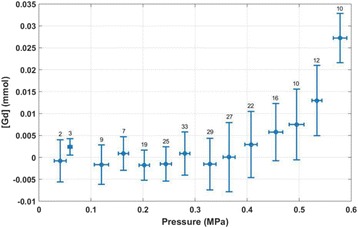
Correlation between the Gd concentration after BBB opening and the estimated in situ pressure using MR-ARFI before BBB opening in each voxel. Data acquired 3 minutes after the opening of the BBB

The second experiment gives a more accurate estimation of the pressure threshold. By plotting the concentrations in contrast agent in the voxel versus the pressure used in these voxel to open the BBB (see Fig. 131), two trends are observables. In a first pressure range, from 0 to 0.4 MPa, the pressure is not sufficient to open the BBB so the concentration is not significant. In a higher pressure range, from 0.4 MPa to 0.6MPa, the pressure is sufficient to open the BBB and the concentration increases as the pressure increases in a roughly linear way. With this experiment the found pressure threshold is about 0.4 MPa.

A third way of determining the opening threshold is to study the width of the opening on the sides [LB1] of the square of the first experiment. Indeed it is possible to plot the concentration profile across the lines of the opening and to compare it to the Gaussian profile of the pressure which has been studied in water tank. It appeared that the width where the BBB is open matches with the width on the Gaussian profile where the pressure is higher than 0.4 MPa.

**Conclusions**

The two different methods proposed for the study of the dependency of drug uptake after BBB disruption with acoustic pressure give consistent results. We find an acoustic pressure threshold for the BBB opening of 0.4 MPa, for a 1.5 MHz working frequency. This result is in good accordance with the literature [5].

By acquiring T2 weighted images after those experiments, we are confident we caused no damages up to 0.6 MPa. This pressure is close to the one for which adverse effects started to appear in our previous studies thought. As a consequence, the pressure window for a safe and efficient BBB opening is quite narrow which highlights that the pressure *in vivo* has to be very well controlled. [LB1] Above the opening threshold, the relationship between drug uptake and acoustic pressure is linear. The knowledge of this relationship is key in order to predict drug pharmacokinetics after BBB disruption.

**References**

[1] MacDannold et al. *Ultrasound Med Biol* 2008

[2] Larrat et al. *Phys Med Biol.* 2010

[3] Magnin et al. *Under review* 2015

[4] Marty et al. *J Cereb Blood Flow Metab.* 2012

[5] Konofagou et al. *Theranostics* 2012

### O94 Response of MR contrast parameters in tissues and tissue mimicking phantoms to histotripsy

#### Steven P. Allen^1^, Luis Hernandez-Garcia^2,1^, Charles A. Cain^1^, Timothy Hall^1^

##### ^1^Biomedical Engineering, University of Michigan, Ann Arbor, MI, United States; ^2^FMRI Lab, University of Michigan, Ann Arbor, MI, United States

**Objectives**

Histotripsy is a non-invasive ablation surgery which uses high intensity acoustic pulses to stimulate a cavitation cloud and homogenize a tissue target. Magnetic resonance imaging (MRI) is a useful tool for assessing lesions made by this therapy. MR contrast parameters such as R2 and the apparent diffusion coefficient (ADC) are sensitive to structural properties of cellular tissue. Changes to these properties, such as homogenization by histotripsy, should induce changes in MR image contrast. In this study we estimate R2 and the ADC of histotripsy lesions made in ex vivo brain, liver, kidney, muscle, blood clot, and various red blood cell (RBC) phantoms commonly used in histotripsy studies.

**Methods**

A 500 kHz, electronically steered, focused transducer (256 elements, f#: 0.5, focal distance: 15 cm, focal length: 3.5 mm, focal width: 1.5mm, PRF: 10 Hz) generated histotripsy bubble clouds in in vitro samples of porcine liver, kidney, bovine liver, brain, and blood clot as well as agar gels mixed with 3, 6, and 16%/wt red blood cells (RBC’s). Lesions were made by electronically steering the focal zone through a grid of points spaced 0.66 mm apart. For each steering point, the transducer emitted a single, two-cycle acoustic pulse (~5 us long) with a peak negative pressure that exceeded 35 MPa. This treatment pattern was repeated until 1, 3, 30, 100, 300, or 1000 pulses were deposited per grid point. A total of 4 lesions for each pulse number were formed in each tissue sample.

After treatment, each sample was placed in the bore of a 7-Tesla small animal MR scanner (Agilent Technologies, Walnut Creek, CA) and imaged using spin-echo sequences with various echo-times and diffusion-weighting b-values. Contrast parameters R2, and ADC were estimated from the resulting images by selecting a region of interest (ROI) within each lesion and performing a non-linear, least-squares fit of the mean signal within the ROI.

**Results**

**Fig. 132 (abstract O94).**
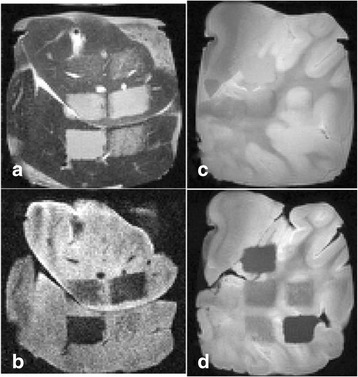
Example images of histotripsy lesions made in in vitro liver (**a**-**b**) and brain (**c**-**d**). Enhanced contrast indicating lesions made in liver can be observed in T2-weighted (**a**) and diffusion-weighted (**b**) images with identical slice plans. Lesions made in brain tissue are difficult to find in a T2-weighted image (**c**) but are readily observed in a diffusion-weighted image (**d**) with an identical slice plan

**Fig. 133 (abstract O94).**
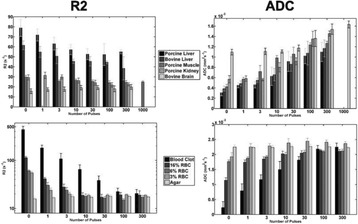
Estimated R2 rates and ADC’s of histotripsy lesions made in various tissues and phantoms plotted against the number of pulses applied to each lesion. The R2 rate decreases with pulse number for all samples except for brain. The ADC increases with pulse number for all samples. Note that the *y*-axis on the bottom left frame is on a logarithmic scale

**Fig. 134 (abstract O94).**
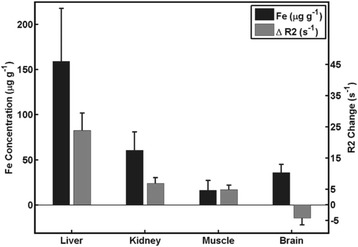
The mean and standard deviation of tissue iron concentrations published in Refs [1–7] as a function of tissue type. Also plotted are the mean and standard deviations of the total change in R2 observed in histotripsy lesions made in each tissue type. The change in R2 appears to correlate with tissue iron concentration

Example R2 and diffusion-weighted (DW) images of lesions made in porcine liver and bovine brain are displayed in Fig. 132. The R2 and ADC parameters measured in each tissue type are plotted as a function of pulse number in Fig. 133. In liver, kidney, muscle, blood clot, and the red blood cell phantoms, homogenization induced measurable decreases in the R2 rate. However, R2 did not change appreciably in brain with treatment. In the red blood cell phantoms, the magnitude of change of the R2 rate decreases with decreasing RBC concentration. In Fig. 134, tissue iron concentrations published in the literature [1–7] are plotted alongside the average maximum change in R2 observed in each tissue type. The total change in R2 appears to decrease with tissue iron concentration. The ADC for all samples increased measurably with treatment.

**Conclusions**

For most samples, the R2 relaxation rate and the ADC changed appreciably with increasing pulse numbers. Both contrast parameters asymptotically approach a final value such that further pulses cause marginal changes in the contrast parameters. Histotripsy induced changes in R2 appear to decrease with decreasing tissue iron concentration. R2 weighted imaging may be a good indicator of homogenization in samples with high iron content such as liver and RBC phantom.

The ADC appears to change with pulse number in all materials reported here, likely because homogenization removes cellular membranes. These results suggest that diffusion-weighted imaging is a good assessment tool for histotripsy therapy. However, R2-weighted imaging may suffice for histotripsy therapy in the body, where tissues contain more iron content and respiratory motion makes diffusion-weighted imaging difficult.

**References**

[1] Riederer P, Sofic E, Rausch WD, Schmidt B, Reynolds GP, Jellinger K, Youdim MB. Transition metals, ferritin, glutathione, and ascorbic acid in parkinsonian brains. J. Neurochem. 1989;52:515–20.

[2] Langkammer C, Krebs N, Goessler W, Scheurer E, Ebner F, Yen K, Fazekas F, Ropele S. Quantitative MR Imaging of Brain Iron: A Postmortem Validation Study 1. Radiology 2010;257:455–462.

[3] Hallgren B, Sourander P. The effect of age on the non-haemin iron in the human brain. J. Neurochem. 1958;3:41–51.

[4] López-Alonso M, Miranda M, Castillo C, Hernández J, García-Vaquero M, Benedito JL. Toxic and essential metals in liver, kidney and muscle of pigs at slaughter in Galicia, north-west Spain. Food Addit. Contam. 2007;24:943–54.

[5] Falandysz J. Some toxic and essential trace metals in swine from Northern Poland. Sci. Total Environ. 1993;136:193–204.

[6] Kongkachuichai R, Napatthalung P, Charoensiri R. Heme and nonheme iron content of animal products commonly consumed in Thailand. J. Food Compos. Anal. 2002;15:389–398.

[7] Coleman ME, Elder RS, Basu P, Koppenaal GP. Trace metals in edible tissues of livestock and poultry. J. AOAC Int. 1992.

### O95 Monitoring of non-cavitational HIFU thermal ablation in ex-vivo ox liver using passive acoustic mapping

#### Erasmia Lyka, Delphine Elbes, Christian Coviello, Robin Cleveland, Constantin Coussios

##### Engineering Science, University of Oxford, Oxford, United Kingdom

**Objectives**

Passive Acoustic Mapping (PAM) is a novel technique that enables spatiotemporal monitoring of sources of nonlinear acoustic emissions received by a multi-element array during ultrasound therapies. So far, it has primarily been used as a method of passive cavitation imaging, either by reconstructing broadband emissions attributable to inertial cavitation [1] or harmonic emissions due to boiling [2] or stable cavitation in the presence of ultrasound contrast agents. In the present work, PAM is used for the first time to spatially map sources of narrowband (harmonic) emissions arising purely due to nonlinear propagation and scattering of the incident ultrasound field, in order to enable real-time monitoring of non-cavitational HIFU thermal ablation. A key advantage of PAM over conventional B-mode imaging is that it enables much higher spectral resolution, making it potentially possible to detect subtle changes in the frequency response of tissue during thermal ablation.

**Methods**

**Fig. 135 (abstract O95).**
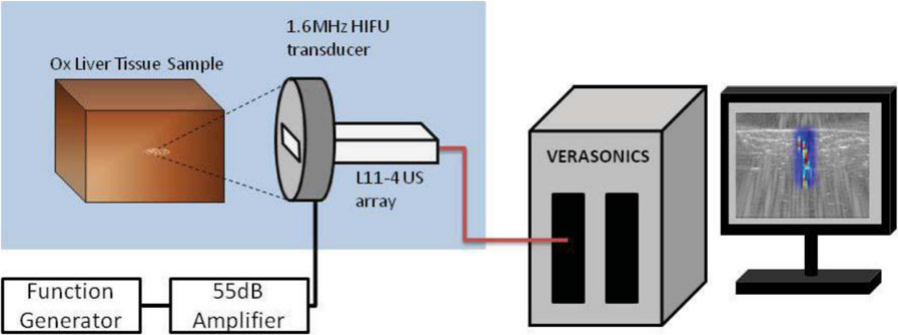
Experimental setup involving an ox liver tissue sample placed at the focus of a HIFU transducer driven at 3.3MHz. The HIFU transducer is co-aligned with a 4-11MHz array for B-mode imaging and passive recording of acoustic emissions. These emissions are filtered, isolating the 2nd and 3rd harmonic of the HIFU transmitting frequency, which are then processed using PAM

A single-element HIFU transducer (H102, Sonic Concepts, 64 mm active diameter, 63 mm focal length) was driven at 3.3MHz , and used to expose degassed ox liver to a range of peak positive pressures leading to varying heating regimes, from hyperthermia to boiling. A linear ultrasound array (L11-4v, Verasonics Inc., 128 elements, 6.25MHz central frequency) was positioned co-axially with the HIFU transducer through a central rectangular cutout, and used for B-mode ultrasound imaging and PAM during HIFU exposure (Fig. 135). The passively recorded acoustic emissions were filtered using a newly developed Sum-Of-Harmonics (SOH) data-adaptive parametric model [3] to accurately isolate the emissions corresponding to the 2nd harmonic (6MHz) and 3rd harmonic (9MHz), and then beamformed [4] to provide PAM maps of sources of nonlinear scattering.

**Results**

During HIFU exposure the spectrum of the passively recorded signals mainly comprises the first three harmonics of the HIFU transmitting frequency. No noticeable changes in the broadband component of the signal were observed for any of the exposure conditions, suggesting that inertial cavitation did not occur at 3.3 MHz over the range of pressures used here. In the case of over-treatment (i,e, treating until sustained boiling occurred), the boiling was successfully detected by both active B-mode imaging and PAM. However, for the vast majority of cases, a lesion was successfully created without any observable hyperecho on B-mode images, yet PAM evidenced a measurable change in the amplitude of the 2nd and 3rd harmonic that coincided spatially and temporally with the appearance of a lesion.

**Conclusions**

PAM has considerable potential as a tool for low-cost, real-time, non-invasive ultrasound-based lesion detection, even in the absence of any measurable cavitation activity. This study paves the way for PAM to be used as a safety tool to prevent both overtreatment due to boiling, and to achieve more efficacious tissue ablation.

**References**

[1] Jensen, C. R., Ritchie, R. W., Gyöngy, M., Collin, J. R. T., Leslie, T., and Coussios, C.-C. (2012). “Spatiotemporal monitoring of high-intensity focused ultrasound therapy with passive acoustic mapping,” Radiology, 262, 252–261.

[2] Choi, J. J. and C.-C. Coussios (2012). "Spatiotemporal evolution of cavitation dynamics exhibited by flowing microbubbles during ultrasound exposure." The Journal of the Acoustical Society of America 132(5): 3538–3549.

[3] Lyka, E., Coviello, C., Kozick, R., and Coussios, C.-C. (2015). “A sum-of-harmonics time-domain method to distinguish harmonic and broadband signals during passive acoustic mapping of ultrasound therapies,” J. Acoust. Soc. Am. 137, 2399.

[4] Coviello, C., Kozick, R., Choi, J., Gyöngy, M., Jensen, C., Smith, P. P., and Coussios, C.-C. (2015). “Passive acoustic mapping utilizing optimal beamforming in ultrasound therapy.

### O96 Diagnosis and treatment monitoring of skin cancer using phase sensitive optical coherence tomography (PHS-OCT) and high-intensity focused ultrasound (HIFU) induced shear wave

#### Kanheng Zhou, Nhan M. Le, Chunhui Li, Zhihong Huang

##### University of Dundee, Dundee, United Kingdom

**Objectives**

Clinical diagnosis of skin cancer relies on the visual examination and palpation by a trained surgeon. It is low in accuracy since it is largely depends on individual experiences. Mechanical properties are important tissue parameters of skin that are useful for understanding skin patho-physiology, and aiding disease diagnosis and treatment, especially in skin cancer diagnosis. To meet this requirement, a sensitive, non-destructive and non-invasive method that is capable of assessing the skin mechanical properties as well as geometry information, in diagnosis and treatment procedures, is needed. Optical coherence elastography (OCE) using phase sensitive optical coherence tomography (PhS-OCT) system has the advantages of a high spatial resolution to resolve skin problems. High intensity focused ultrasound (HIFU) is a well-known non-contact method and non-invasive method for shear wave inducer and cancer treatment. The objective of this study is to 1) combine PhS-OCT and HIFU, and 2) recover the elasticity map of skin from this new combination for diagnosis.

**Methods**

This system consists of shear wave generation part and detection part. Shear wave generation part includes miniature HIFU transducer, signal generator, power amplifier and impedance matching transformer. The miniature HIFU transducer (1.89 MHz, 20mm diameter, 16mm radius, and 12.5mm focal length) is produced in-house, pressure-field and calibrated. The driven signal for HIFU transducer was 1 volt burst sine-wave signal with 20 cycles/pulse at 2.091MHz, which was triggered by 125Hz external TTL. The acoustic power output by the HIFU transducer was 3.72w. For shear wave detection the system employs a PhS-OCT system. It performs a 46.8 kHz sampling rate with axial resolution of 0.5 μm, resulting in sampling the complete data with the format of 1024 depth x 256 frames x 256 scan-lines in approximately 2 seconds. Each frame was processed with respect to the previous one to determine the phase shift.

This study begun with the *ex-vivo* experiment on porcine abdomen skin, which was embedded in 2% agar phantom and put under the PhS-OCT for observation. There were 2 sets of experiments on 3 different samples to investigate the propagation process of shear-wave in different position; each was repeated three times.

**Results**

**Fig. 136 (abstract O96).**
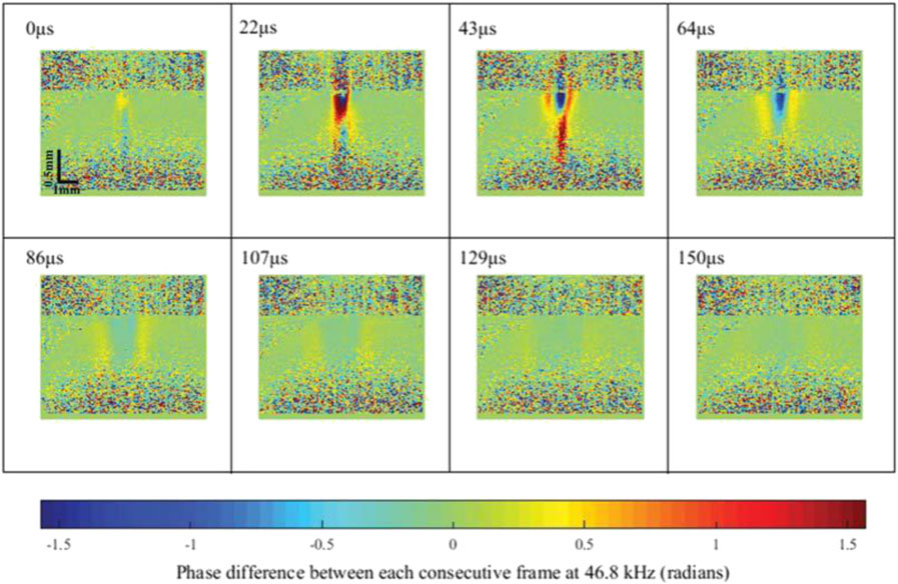
The propagation process of shear-wave in porcine skin

The propagation process of shear wave in porcine skin is shown in Fig. 136. The figure contains 2D maps of phase differences between each consecutive frame at 46.8 kHz with respect to the time after the first synchronized signal, at the shear-wave source (HIFU focus, 2.091MHz, 20 cycles/pulse at 3.72W acoustic power).

**Fig. 137 (abstract O96).**
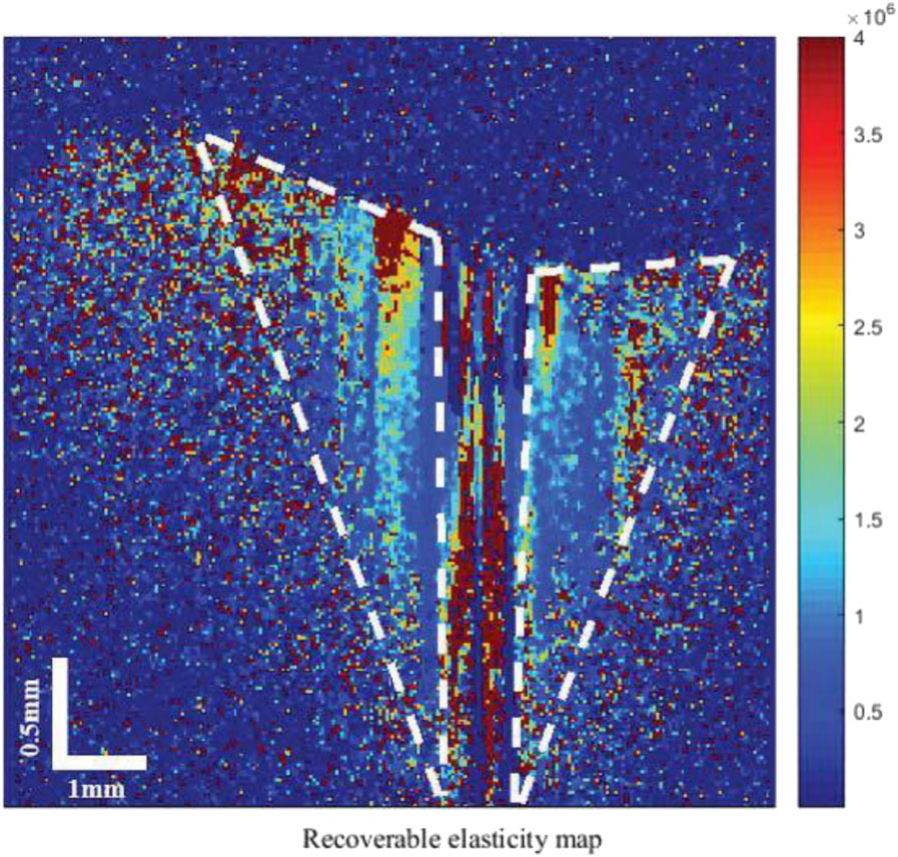
the elasticity map of porcine skin

The elasticity map of porcine skin is shown in Fig. 137. The elasticity of porcine skin was calculated according to the map of phase differences. The recoverable electricity area was annotated by the white dashed line in the figure.

The Young’s modulus of each set of experiment was also calculated according to the map of phase differences. The Young’s modulus of the first sample is 1.314±0.017 MPa, followed by 0.932±0.0171 MPa and 1.5815±0.2264 MPa for the second and the third sample respectively. This agrees well with the finding of Ankerson *et al*. 1999.

**Conclusions**

Our work combines PhS-OCT and HIFU for the diagnosis and treatment monitoring of skin cancer. This study demonstrates the feasibility of elasticity measurement on *ex-vivo* porcine skin using the proposed setup. The initial results are in agreement with the previous study on porcine skin. However, HIFU-induced shear-wave is required to have a large bandwidth (sharp pulse) in order to utilise PhS-OCT running at micron resolution. This requirement needs to be compensated for a high attenuation coefficient of high frequency shear-wave component.

## POSTERS

### P1 High-reliable high-voltage staircase drive circuit for high-intensity therapeutic ultrasound system

#### Satoshi Tamano^1,2^, Hayato Jimbo^1^, Takashi Azuma^3^, Shin Yoshizawa^1^, Keisuke Fujiwara^2^, Kazunori Itani^2^, Shin-ichiro Umemura^4^

##### ^1^Graduate School of Engineering, Tohoku University, Sendai, Miyagi, Japan; ^2^Hitachi Aloka Medical, Ltd., Kokubunji, Tokyo, Japan; ^3^Department of Mechanical Engineering, The University of Tokyo, Bunkyo, Tokyo, Japan; ^4^Graduate School of Biomedical Engineering, Tohoku University, Sendai, Miyagi, Japan

**Objectives**

Recently, in the treatment of diseases such as cancer, non-invasive or low invasive modality such as high-intensity focused ultrasound (HIFU) has been put into practice as an alternative to open surgery. HIFU induces thermal ablation of target tissue to be treated. To improve the efficiency of HIFU, we have proposed a "triggered-HIFU" technique, which uses the combination of a short-duration, high-voltage transmission and a long-duration, medium-voltage transmission. In this method, the transmission device has to endure the high peak voltage for the former and the high time average power for the latter. The triggered-HIFU sequence requires electronical scanning of HIFU focus to maximize its thermal efficiency. Therefore, the transmission device has to drive an array transducer with number of elements in the order of a hundred or more, which requires that each part of the device to drive each element has to be compact. The purpose of this presentation is to propose and construct such a transmission device by improving the staircase drive circuit, which we previously proposed. In the previous staircase HIFU drive circuit using MOSFETs, there is a risk of device damage due to the excess heating of the MOSFETs and the spike noise exceeding the device rating. The objective of this presentation here is to realize an HIFU transmission circuit suitable for “triggered HIFU” mode by:

1. suppressing the heating of MOSFET transmission circuit, inhibition of spike noise exceeding the device rating,
2. reducing the odd harmonic components of output voltage for preventing unwanted nearfield tissue heating.

**Methods**

If the load of the MOSFET has inductance components, such as cable inductance or PCB wiring, flyback voltage would be generated at the time of turn off the switching operation, which cause the spike noise exceeding the supply voltage. On the other hand, if it is a capacitive load as a HIFU transducer, the fall of the positive voltage or the rise of negative voltage, it is not possible to discharge the electric charge applied to the transducer. These phenomena bring the excessive heat generation of the MOSFETs, and the transmission waveform would be a rectangular shape. To solve these problems, we developed a new circuit for feeding back charges exceeding the supply voltage, to the power supply circuit. As a result, for the HIFU system, MOSFET damage and power supply, MOSFET and diode damage should be avoided, it is possible to build a HIFU transmission system with safety.

**Results**

In the proposed circuit, MOSFET maximum temperature rise reached 16.4°C, and total power consumption is 2.4W at 1.01MHz transmission. This result shows a 26.7% power consumption reduction and a MOSFET maximum temperature rise of 14.5°C less than the previous circuit. Furthermore, at 1.01 MHz transmission, the third and fifth harmonic attenuation rates of the fundamental wave were −30.0 dB and −62.0 dB respectively. As a result, in the transmission waveform, ringing and spikes exceeding the power supply voltage has been extremely suppressed. In spite of not only the inductance load of the cable, but also the capacitive load by the transducer. We got a prospect of significantly reduces the circuit temperature rise, and power consumption in the heating-mode by these results. Therefore, the possibility of MOSFET damage due to excessive heat was alleviated. As these results, our new proposed circuit should be safe and suitable for HIFU transmission during heating-mode, because the possibility of damage to the MOSFET was alleviated than the previous circuit.

**Conclusions**

We proposed a HIFU transmission circuit suitable for triggered-HIFU mode. The proposed circuit is effective for reducing power consumption, temperature rise suppression, odd harmonic reduction, help to HIFU transmitter circuit safety, during the heating-mode. Meanwhile, the proposed circuit utility of in trigger-mode was not disclosed on the basis of the experimental results. We will show the effectiveness of the proposed circuit in trigger-mode, construct a prototype system more than 100 channels using the proposed method, and deal with the ablation studies using array HIFU probe.

### P2 MRI guided focused ultrasound robotic system for animal experiments

#### Christakis Damianou, Marinos Yiannakou

##### Cyprus University of Technology, Limassol, Cyprus

**Objectives**

In this paper an MRI-guided focused ultrasound (MRgFUS) robotic system was developed that can be used for conducting experiments in animals. The robotic system is capable to scan the ultrasound transducer for performing MR-guided focused ultrasound experiments in in any size of animals.

**Methods**

A single element spherically focused transducer of 3 cm diameter, focusing at 10 cm and operating at 1 MHz was used. The positioning device incorporates only MRI compatible materials such as piezoelectric motors, Acrylonitrile Butadiene Styrene (ABS) plastic, brass screws, and brass rods. The propagation of ultrasound is a bottom to top approach.

**Results**

The system was tested successfully in freshly excised tissues from animals for various tasks (creation of single lesions, creation of overlapping lesions, accuracy, and MR compatibility).

**Conclusions**

A simple, cost effective, portable positioning device has been developed which can be used in virtually any clinical MRI scanner since it can be sited on the scanner's table. The system was proven accurate, reliable, safe and functional. This system has the potential to be marketed as a cost effective solution for performing experiments in small and large animals.

### P3 Development of an adaptive robotic-assisted preclinical MR-HIFU system on a clinical platform

#### Nicholas Ellens^1^, Ari Partanen^1, 2^, Dan Stoianovici^3^, Keyvan Farahani^1, 4^

##### ^1^Radiology, Johns Hopkins University, Baltimore, MD, United States; ^2^Philips, Andover, MA, United States; ^3^Urology, Johns Hopkins University, Baltimore, MD, United States; ^4^National Cancer Institute, Bethesda, MD, United States

**Objectives**

Magnetic resonance guided high-intensity focused ultrasound (MR-HIFU) has been used for the treatment of a variety of diseases and conditions. Clinically, these procedures are performed by commercial MR-HIFU platforms designed for particular anatomical targets: uterine fibroids for the InSighTec ExAblate 2000/2100 and the Philips Sonalleve; the brain for the InSighTec ExAblate 4000. The former class uses a transducer array with a robotic positioning system integrated with a MRI bed with limited mechanical translation and electronic steering. The latter is mechanically fixed but has a larger number of elements for electronic distortion correction and steering.

Liberating the transducer from the mechanical confines of the existing clinical systems may allow for easier access to more anatomical targets. The goal of this study is to develop an anatomically adaptive MR-HIFU system using a transducer mounted on a robotic arm that can be positioned within the MRI bore with a large range of motion and a versatile mechanism for acoustic coupling. As a precursor to an adaptive clinical system, this study discusses the capabilities and results of a preclinical system built on the Philips Sonalleve MR-HIFU software platform. The objectives are:

1. Integrate a preclinical-specific, multi-element transducer [1] with a novel MR-Safe robotic manipulation technology [2–3] for adaptable focused ultrasound in preclinical animal models.
2. Implement graphical overlays and sonication controls of this ultrasound system with the Philips Sonalleve clinical MR-HIFU treatment planning platform to plan the ultrasound focus prior to sonication and to monitor therapy in real-time using automatically-oriented MR-thermometry.
3. Assess the ability of the transducer to remain acoustically coupled to the target during movement and quantify sonication targeting accuracy.

**Methods**

A 5 cm, 3 MHz, f-number 1 transducer with 8 elements in a sector-vortex arrangement was selected for this study. A flexible polyethylene membrane was affixed to the transducer shell that could be inflated or deflated with degassed water to maintain acoustic coupling while changing the focal depth in the target tissue. The frequency used was desirable as it allowed higher attenuation than would be achieved at frequencies typically used clinically (1–1.5 MHz) so as to avoid far-field structures. The sector-vortex array design allowed for tight focusing (mode 0, all elements in phase) or more diffuse focusing (modes 1–3, with the phase difference (phase) between adjacent elements of 180°, 90°, and 45°, respectively) to emulate the focal size of clinical systems [1].

The Sonalleve software platform (Philips, Vantaa, Finland) was modified to interface with the transducer. First, a virtual model of the transducer was designed that could be positioned arbitrarily in the MR image space. By aligning the model with markers on the transducer using the therapy planning software, the ultrasound focus could be identified and MR-thermometry slices positioned automatically, both parallel and perpendicular to the ultrasound beam. Second, the software pipeline was modified to send power, phase, start, and stop commands to the amplifiers via a serial connection.

The feasibility of using an existing MR-safe robot [4–6] to move the transducer accurately and maintain acoustic coupling was assessed in vitro and ex vivo. An acrylamide-based tissue-mimicking gel phantom containing silica particles (1.0% w/v) and bovine serum albumin (BSA, 3% w/v) was sonicated (3 MHz, mode 0) at room temperature (22°C) in a regular grid pattern (15 mm spacing) at electrical powers of 4, 8, 16, and 32 W, for durations of 30, 60, and 90 s. A fresh, excised pig leg (27°C) was shaved and treated with a depilatory agent to remove all hair. Nine locations in the pig leg were sonicated with electrical powers ranging between 32 and 64 W for durations of 30 to 90 s. MRI T2 maps were acquired to identify volumes of protein denaturation, and the overlying tissue in the pig leg was dissected to observe volumes of visible thermal damage.

**Results**

**Fig. 138 (abstract P3).**
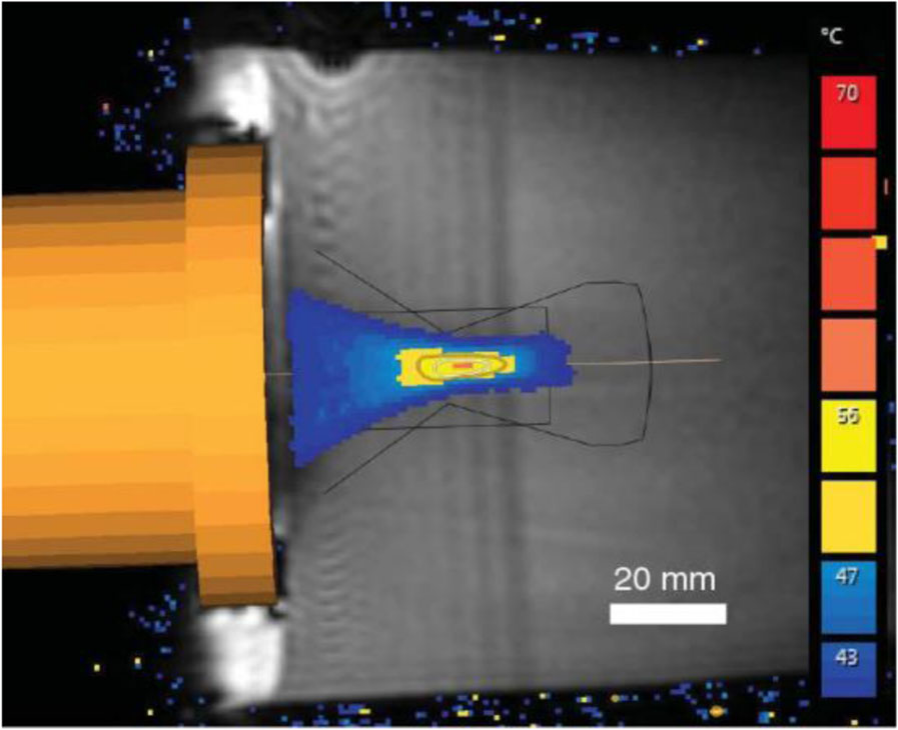
MR image showing transducer overlay (*gold colour*, *left*) and anticipated focal profile (contained with *dark-coloured lines*) along with the resulting temperature increase visualized using the parallel MR-thermometry slice, centred on the ultrasound focus. The transducer was driven in mode 1 (phase=180°) for 60 seconds at 16 W (electric)

**Fig. 139 (abstract P3).**
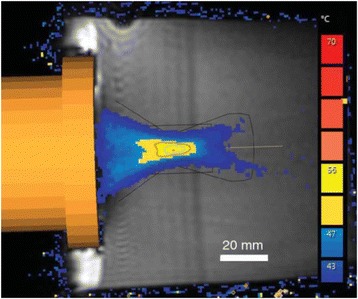
Heating profile produced by driving the transducer in mode 3 (phase=45°) for 60 seconds at 16 W electric

Screenshots showing the transducer overlay and sonication location are shown in Figs. 138 and 139. Through the use of added graphical overlays, it was possible to predict the location of peak heating to within 1.8 mm, based on the location of visible fiducial markers on the transducer case.

**Fig. 140 (abstract P3).**
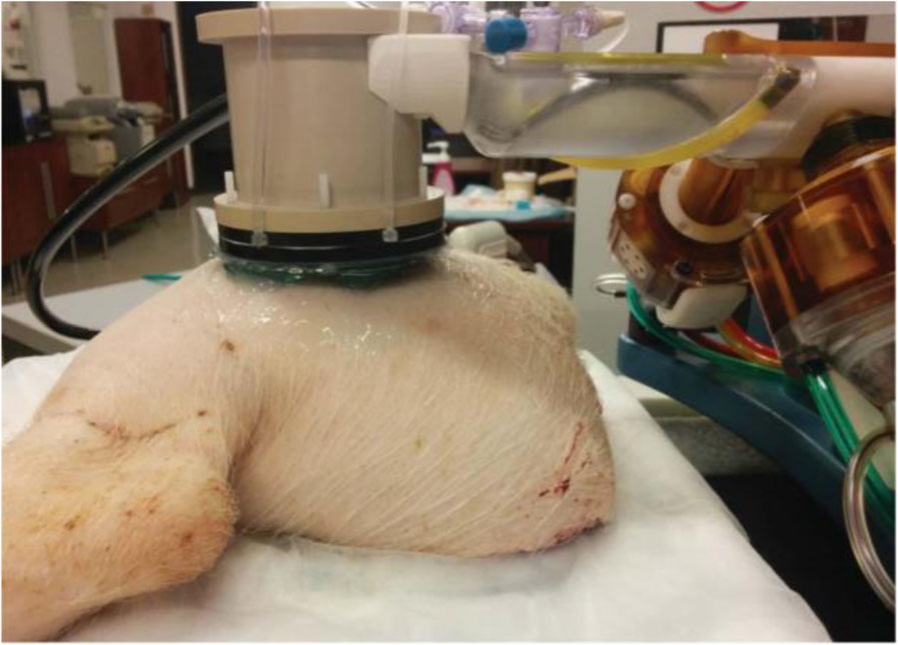
MR-safe robot with attached transducer coupled to an *ex-vivo* pig leg

**Fig. 141 (abstract P3).**
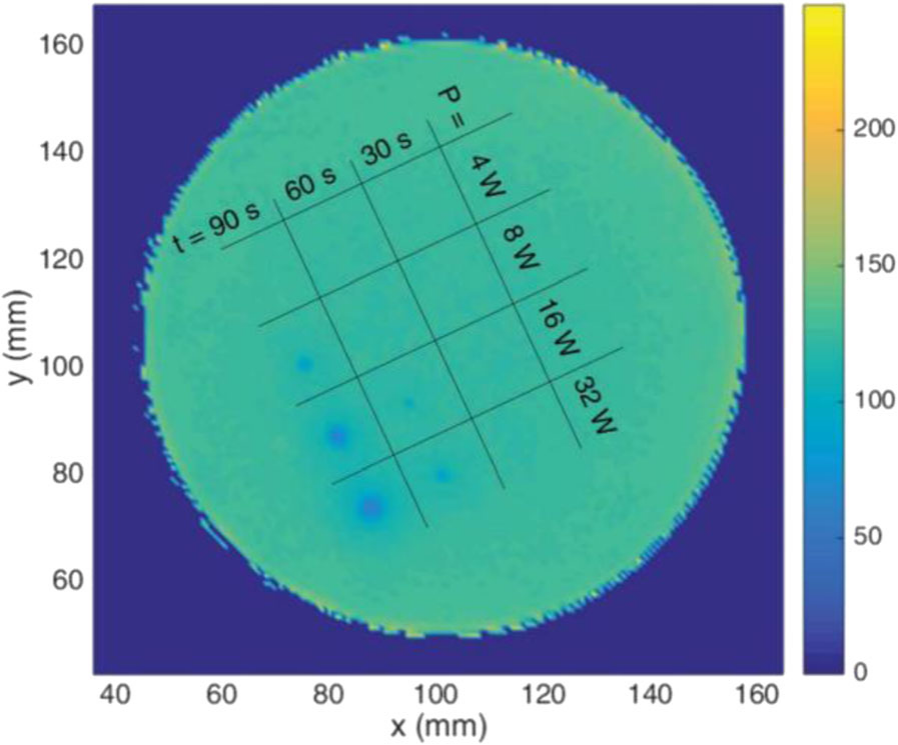
Map of post-sonication T2 values of the phantom with an overlaid grid indicating the duration and power used at that location. Lower T2 values (ms) indicate a greater degree of protein denaturation, visible only for longer durations (60 s, 90 s) and higher powers (8 – 32 W)

The flexible membrane was used to couple the transducer to the phantom and leg specimen, shown together with the robot in Fig. 140. The measured spatial error between the centre of the observed T2 decrease due to protein coagulation and the desired sonication locations was found to be 0.3 ± 0.1 mm in the phantom. A T2 map from one phantom experiment is shown in Fig. 141. The coupling layer was suitable for multiple sonications in the pig leg despite its curvature without any other intervention, demonstrating the feasibility of using this method for robotic surgery.

**Conclusions**

The initial integration of a preclinical HIFU transducer with the MR-safe robot and clinical MR-HIFU planning and control software was successful, though further integration is desirable to facilitate image-guided treatments. The transducer and robot are suitable for accurate sonication at depths relevant for many preclinical applications.

**References**

[1] Burke, C., et al. (2012). STM Annual Meeting. Portland, OR, USA.

[2] Stoianovici, D., et al. (2014). IEEE/ASME TM, 19(4), 1289–99.

[3] Stoianovici, D., et al. (2007). IEEE/ASME TM, 12(1), 98–106.

[4] Muntener, M., et al. (2006). Urology, 68(6), 1313–7.

[5] Muntener, M., et al. (2008). Radiology, 247(2), 543–549.

[6] Stoianovici, D., et al. (2007). MITAT, 16(4), 241–8.

### P4 Composite transducer with heavy matching layer to produce second harmonic as well as fundamental

#### Zulfadhli Zaini^1^, Ryo Takagi^2^, Shin Yoshizawa^2^, Shin-ichiro Umemura^1^

##### ^1^Biomedical Engineering, Tohoku University, Sendai, Japan; ^2^Communication Engineering, Tohoku University, Sendai, Japan

###### **Correspondence:** Zulfadhli Zaini

**Objectives**

Cavitation microbubbles are known to enhance therapeutic effects of ultrasound. They can be incepted in a controlled manner by short ultrasonic pulses with highly negative pressure. Such highly negative pressure exceeding the cavitation threshold is difficult to produce simply by focusing because of nonlinear propagation followed by focal phase shift. Superimposing the second harmonic to fundamental can significantly reduce the problem, but the conventional design of thickness mode transducer is not suitable to generate both the second harmonic and fundamental at the same time. The objective of this study is to overcome this problem.

**Methods**

**Fig. 142 (abstract P4).**
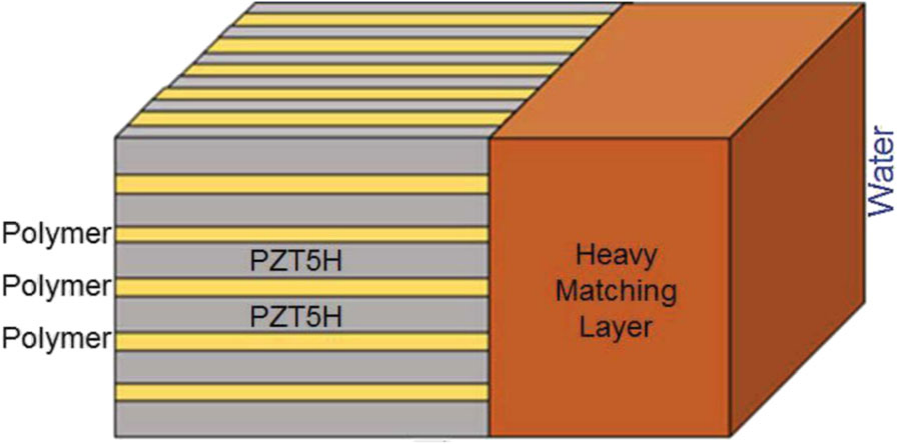
See text for description

We propose an approach of a high acoustic impedance matching layer. Unlike a conventional matching layer, the acoustic impedance is chosen nearly as high as that of the piezo-composite. Numerical simulation was performed using a code, PZFlex, to optimize the acoustic impedance and the thickness of the matching layer so that an air backed transducer, as schematically shown in Fig. 142, can produce both the second harmonic (2 MHz) and fundamental (1 MHz) at a similarly high efficiency. Here, the efficiency was defined as the produced acoustic power divided by the square of the drive voltage. Then, a prototype transducer was constructed based on the simulation result. The real and imaginary electrical admittance of a transducer element was measured, and the acoustic power from seven transducer elements was measured by radiation force.

**Results**

**Fig. 143 (abstract P4).**
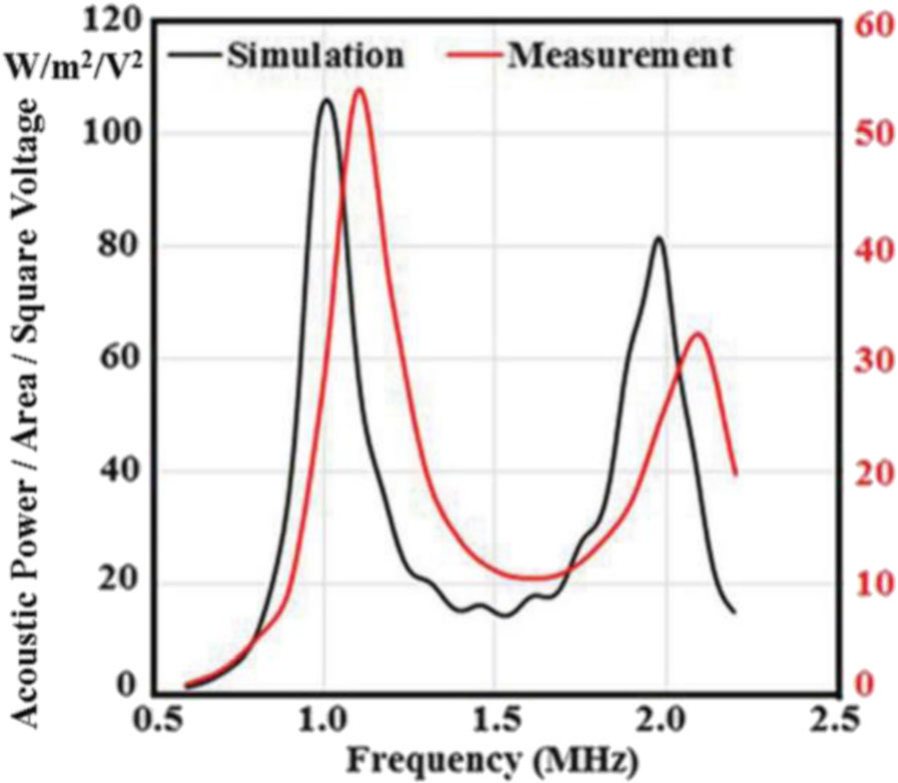
See text for description

The simulation and experimental results shown in Fig. 143 emphasize that the transducer with a heavy matching layer demonstrate to be able to practically produce both the fundamental and second harmonic at high efficiency. They also illustrate the efficacy of the aforementioned concept. The phase of electrical admittance at 1 and 2 MHz were designed near to zero so that the high power can be transferred with a maximum power factor. The fundamental and second harmonic peaks in efficiency from simulation were around 1.01 and 1.98 MHz while the measurements were around 1.1 and 2.1 MHz.

Further studies are needed to explain the relatively large difference in amplitude between the simulation and measurement.

**Conclusions**

Implementing a transducer with a high impedance matching layer with an appropriate thickness on piezocomposite was demonstrated to be able to produce both the fundamental and second harmonic at high efficiency, while the conventional air back transducer cannot. Even though there is significant difference in amplitude between the simulation and measurement results, the result produced from both simulation and measurement shows the capability of transducer with a high impedance matching layer to produce both the fundamental and second harmonic at the same time.

### P5 Compatibility evaluation between MR and ultrasonic probes in MR-guided FUS from SNR of images and B_0_ homogeneities

#### Shenyan Zong^1^, Guofeng Shen^2^

##### ^1^School of Biomedical Engineering, Shanghai Jiao Tong University, Biomedical Instrument Institute, Shanghai, China; ^2^Shanghai Jiao Tong University, Med-X Research Institute, Shanghai, China

**Objectives**

Magnetic resonance (MR) is the most widely used for focus targeting and temperature monitoring in focused ultrasound surgery (FUS). The aim of this study is to demonstrate the feasibility and the effectiveness of the two parameters, SNR and B_0_ homogeneities for compatibility evaluation.

**Methods**

In this study, we investigated the influence of ultrasonic probes on the MR imaging from SNR and B_0_ homogeneities. The SNR of images can be used to evaluate the level of electromagnetic interference from the ultrasonic probes. The images were acquired on the conditions that the ultrasonic probes worked on four different voltages, and the calculated SNRs were compared with the original SNR without power. For more scientific, each image was segmented to five sub-regions and the SNR of each part was calculated respectively. The B_0_ homogeneities can be used to guarantee the accuracy of thermometry. The effect of B_0_ inhomogeneities caused by the ultrasonic probes was estimated from B_0_ field mappings with and without the ultrasonic probes in the magnetic field. For more accurate, the B_0_ field mappings were measured from three slices near the ultrasonic probes and three slices away from the ultrasonic probes.

**Results**

Here, the SNR values of each same sub-region in different working-voltage images were smaller than that in no-power image. Meanwhile, the B_0_ field mappings of all slices exhibited that the B_0_ field transformed near the ultrasonic probes. However, the B_0_ field away from the ultrasonic probes almost did not change.

**Conclusions**

The two indicators, SNR of images and B_0_ homogeneities, are proved to be effective on the compatibility evaluation between MR and ultrasonic probes.

### P6 Immersion of MRI imaging coils in therapeutic ultrasound

#### Ron Watkins

##### Radiology, Stanford University, Stanford, CA, USA

**Objectives**

The objective of this work is to examine problems and opportunities of immersing MRI surface coils in water. We will look at issues of frequency shift and induced RF losses. The work examines the interaction of electric fields and water. We will discuss number design techniques to overcome these interactions and ways to minimize performance problems due to coils being under water. We will also discuss safety electrical issues involving connections to the MRI system in or around water used with therapeutic ultrasound.

**Methods**

We used very conventional methods of reducing local electric fields in MRI coils by choosing the number of capacitor breaks and looking at the value of each capacitor used in the design. We evaluated several coils with varying number of capacitors which in turn determines the value of each capacitor. We examined the centre frequency and Q of various coils with different numbers of capacitor breaks as well as several coatings for the coils. We derived equivalent circuit models to compare frequency shift on resistive losses due to the interaction of electric fields and water.

**Results**

We found that increasing the number of capacitor breaks increased the resistive losses slightly but significantly reduced the frequency shift induced by the presence of water. Almost all coating decreased frequency shift by a small amount and did not significantly increase losses.

**Conclusions**

When surface coils are immersed in water, the high permittivity of water can induce very large tuning shifts in MRI coils. This can be mitigates by increasing the number of capacitor breaks in the coil design. The water itself does add some resistive losses to the coil but not by a large amount. Several coatings were effective at reducing corrosion of copper from water. None of the coating did introduce a small shift in centre frequency.

### P7 The histopathology of pancreatic HIU ablation via endo-luminal applicator in a pig model

#### Aurea Pascal-Tenorio^1,9^, Matthew Adams^2,10^, Juan C. Plata^3^, Vasant Salgaonkar^4^, Peter Jones^5^, Kim Butts-Pauly^6^, Chris Diederich^7^, Donna Bouley^8^

##### ^1^Comparative Medicine, Stanford University, Palo Alto, CA, USA; ^2^Thermal Therapy Research Group, UCSF, San Francisco, CA, USA; ^3^Radiology, Stanford University, Palo Alto, CA, USA; ^4^Thermal Therapy Research Group, UCSF, San Francisco, CA, USA; ^5^Thermal Therapy Research Group, UCSF, San Francisco, CA, USA; ^6^Radiology, Stanford University, Palo Alto, CA, USA; ^7^Thermal Therapy Research Group, UCSF, San Francisco, CA, USA; ^8^Comparative Medicine, Stanford University, Palo Alto, CA, USA; ^9^Radiology, Stanford University, Palo Alto, CA, USA; ^10^Bioengineering, UC Berkeley-UCSF Joint, San Francisco, CA, USA

**Objectives**

Pancreatic lesions created during ablations, were examined histologically to determine the extent of tissue/cell death, in lesions created in the in vivo pigs. This current study used applicators positioned in the stomach via the oesophagus, with HIU delivered through the stomach wall to the pancreas. Our histopathologic analyses will help the interventional radiologists optimize applicator placement and thermal dose delivery necessary to ablate normal pancreatic tissue with the ultimate goal of ablating pancreatic cancer in human patients.

**Methods**

**Fig. 144 (abstract P7).**
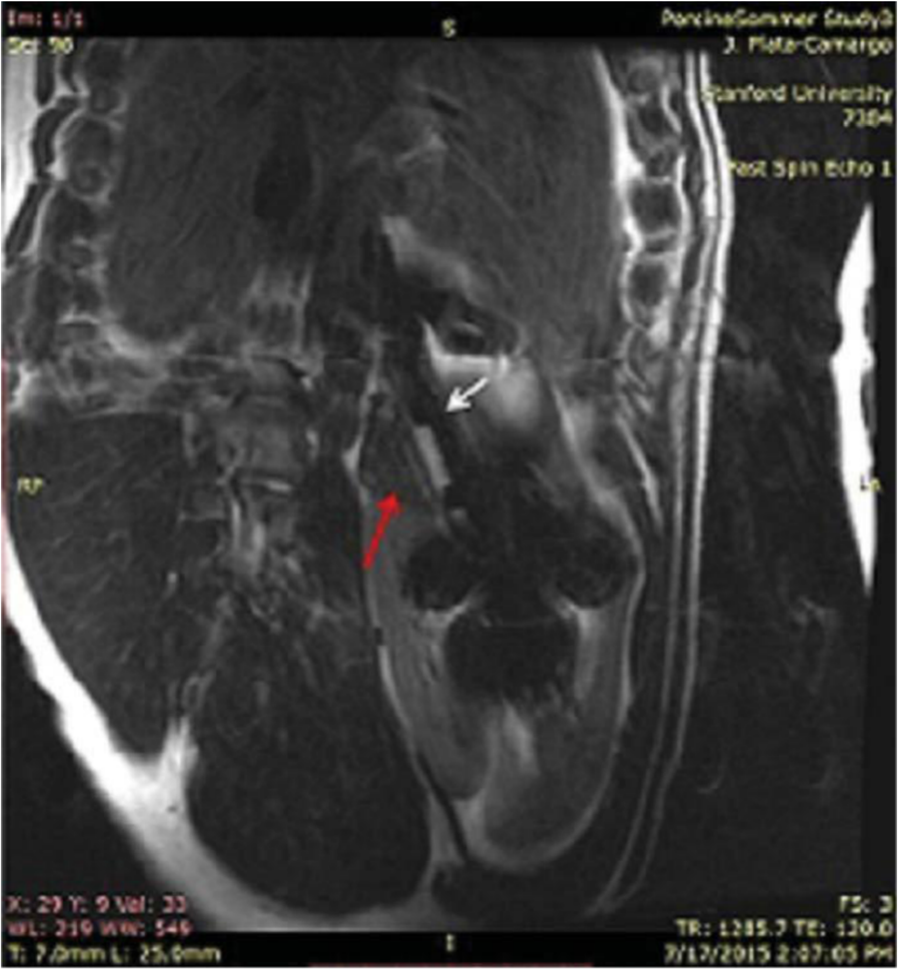
T2-weighted FSE image of the endoluminal ultrasound applicator (*white arrow*) positioned in the stomach lumen of an *in vivo* porcine model for treatment of adjacent pancreatic tissue (*red arrow*)

In vivo experiments: All ablation in vivo studies were performed in anesthetized pigs that were euthanized following post-treatment imaging. All animal studies were approved by Stanford’s Institutional Animal Use and Care Committee in accordance with the Guide for the Care and Use of Laboratory Animals. Three pigs were treated with an applicator introduced in to the stomach via the oesophagus (Fig. 144). HFU was directed through the stomach wall into the pancreas, a six minute treatment with approximate average intensity of 4 W/cm^2^ was performed to ablate the tissue. Following euthanasia, the pancreata and associated tissues (gastric wall) were removed and examined grossly, then incubated in TTC for better visualization of ablation lesions. Affected tissues were then fixed in 10% BNF, routinely processed for histology and H&E staining of microscopic slides, and examined under a light microscope by a board certified veterinary pathologist.

**Results**

**Fig. 145 (abstract P7).**
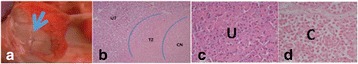
HIU ablation of pig pancreas. **a** Post treatment excised pancreas stained with TTC shows viable tissue red and ablated tissue as tan (blue arrow) **b** - **d**) Histology of treated pancreas (**b)** depiction of 3 regions, untreated (UT), transition zone (TZ), and coagulation necrosis (CN) 25x. **c** Untreated 200x, D) Coagulation necrosis of treated pancreas 200x. Slides are stained with H&E

HIU ablation lesions in all pancreata treatments were visible grossly post-TTC incubation as non-stained (white) regions within normal red-stained pancreatic tissue (Fig. 145a). Histologically, lesions consisted of a central area of coagulative necrosis (CN), characterized by a “ghosted” appearance of cells and nuclear dissolution (karyorrhexis and karyolysis) (Fig. 145d). Surrounding the central necrotic lesions was a thin zone of capillary congestion, scattered haemorrhages, and acinar fragmentation in the transition zone (TZ) where the normal architecture of the organ was disrupted, but individual cells were less severely affected.

**Conclusions**

Pig pancreata were successfully ablated in regions targeted by the endo-luminal HIU applicator. Via MR-guidance, HIU ablation resulted in discrete zones of coagulation necrosis in the pancreas with minimal damage to surrounding tissues. Continued studies using this model will help further optimize MR-imaging and detection of ablation lesions in vivo, and provide a minimally invasive treatment with improved outcome for patients with pancreatic cancer.

### P8 Combinational therapeutic treatment of superficial tissues

#### Andrey Rybyanets

##### Southern Federal University, Rostov on Don, Russian Federation

**Objectives**

Systems and methods for performing a surgical, therapeutic or aesthetic medical procedure in target tissues of patient’s body by using HIFU are well known in the art. Recently, the HIFU systems were proposed for body aesthetic therapy by adipose tissue lysis. The main disadvantage of HIFU application for treatment of large volumes of tissues is small treated volume in lateral direction. Other disadvantage of conventional HIFU treatment of superficial tissues is a restricted number of body areas suitable for the procedure. Therefore, the need exists for new methods and devices aimed at treatment of large volumes of tissue, as for example in the case of removing significant amounts of adipose tissue from arbitrary body parts. The need also exists for devices and methods for treating the skin and subcutaneous adipose tissue region using ultrasound energy, wherein the energy is applied in a more efficient, safe and effective manner. The paper introduces an innovative combinational treatment method for non-invasive surgical, therapeutic, lipolytic or cosmetic treatment of tissues including subcutaneous adipose tissue, cellulite or skin on arbitrary body part of patient.

**Methods**

The method is based on simultaneous or successive applying of constructively interfering physically and biologically sensed influences: ultrasonic standing waves (USW), radio-frequency (RF) heating, vacuum massage, and transdermal drags delivery.

Unlike all existing HIFU and non-focused systems, ultrasound energy in USW directed parallel to the body surface and fully localized in treated body region. Resulting USW efficiency is comparable with HIFU at huge increase of treated tissue volume. Continuous cyclic changes of the nodal pattern of USW with proper repetition rates corresponding to a specific resonant or relaxation times of living cells or tissue components provide effective dynamical influence of USW on tissues. Synergetic combination of USW with RF therapeutic heating and vacuum massage lowering cavitation threshold and intensifying a blood flow and clearance of disrupted cell debris along with inherent treatment process control and diagnostic possibilities offers a great future for the technology. The technology provides also an opportunity to use phonophoresis as an option for transdermal drags delivery using drug-loaded microcapsules.

**Results**

The paper provides the basic physical principles of USW as well as critical comparison of USW and HIFU therapeutic treatment methods. The results of finite-elements and finite-difference modelling of USW formation in tissues are presented. Therapeutic head design comprising USW and RF transducers, and vacuum system, as well as original ex vivo experiments on tissues are described. Physical and biological effects of USW - tissue interaction and synergetic aspects of USW, RF, and vacuum massage combinational influences are shown. The diagnostic capabilities of the USW and RF methods, as well as the possibility of transdermal drags delivery using developed therapeutic head are also demonstrated changes of the nodal pattern of USW with proper repetition rates corresponding to a specific resonant or relaxation times of living cells or tissue components provide effective dynamical influence of USW on tissues. Synergetic combination of USW with RF therapeutic heating and vacuum massage lowering cavitation threshold and intensifying a blood flow and clearance of disrupted cell debris along with inherent treatment process control and diagnostic possibilities offers a great future for the technology. The technology provides also an opportunity to use phonophoresis as an option for transdermal drags delivery using drug-loaded microcapsules.

**Conclusions**

Synergetic combination of USW influence, RF therapeutic heating, phonophoresis, and vacuum massage along with inherent therapeutic treatment process control and diagnostic possibilities offers a great future for the technology.

**Fig. 146 (abstract P8).**
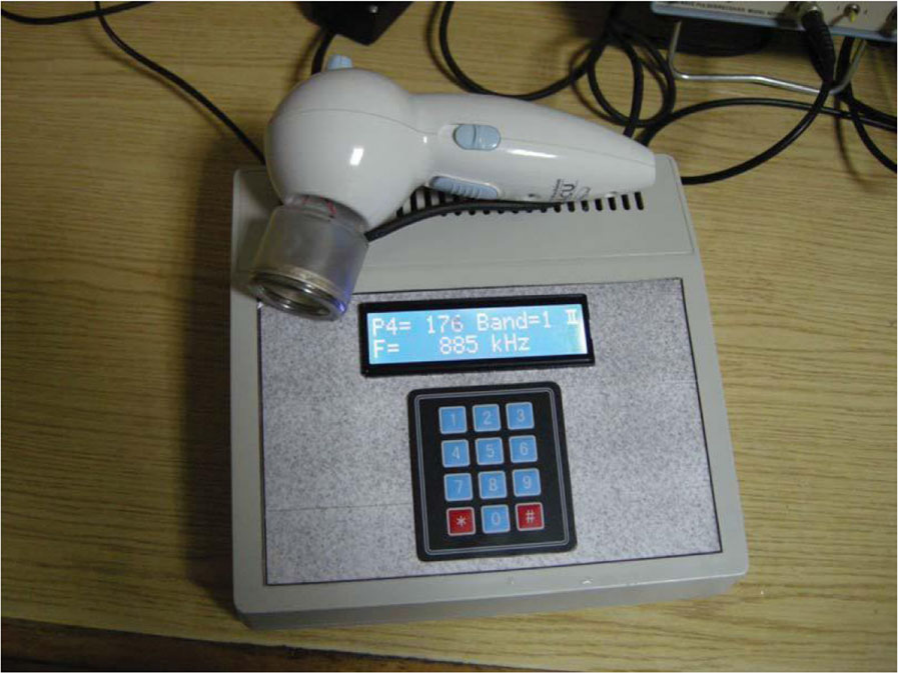
Combinational therapeutic treatment device

**Fig. 147 (abstract P8).**
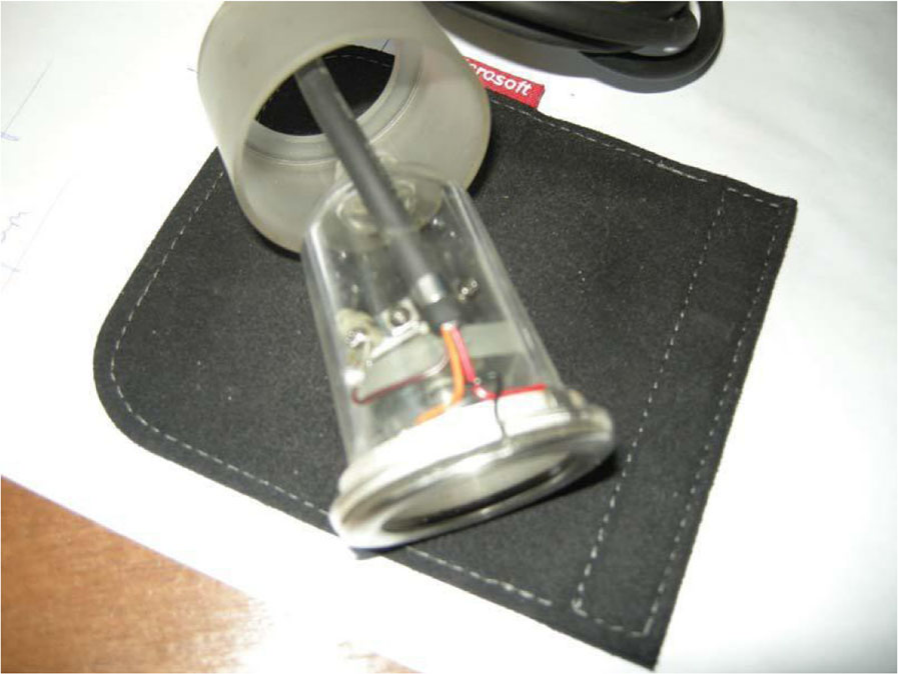
Vacuum cap

**Fig. 148 (abstract P8).**
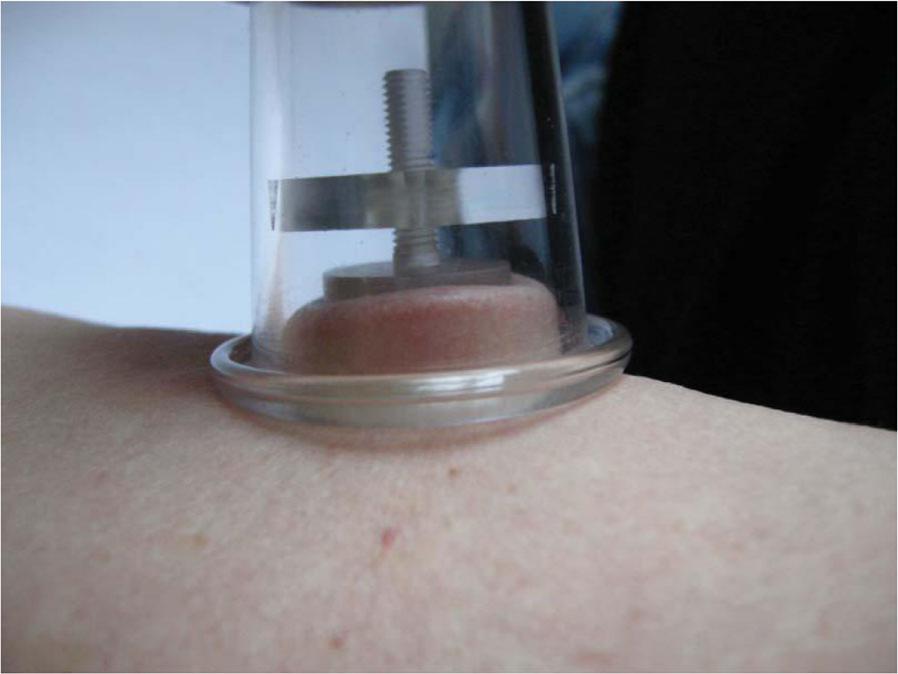
Therapeutic head

### P9 Ultrasound hyperthermia can improve sensitivity to chemotherapeutics in TCA8113 cells

#### Guoxin Ren, Wei Guo, Guofeng Shen, Yazhu Chen

##### Shangai Jiao Tong University, Shanghai, China

**Objectives**

To explore the influence of chemosensitivity of Tca8113 (a human tongue squamous carcinoma cell line) cells by modified MTT assay after the animal models of Tca8113 were treated by the ultrasound hyperthermia system.

**Methods**

To heat the tumours in Tca8113 nude mice (BALB/C) models by the ultrasound hyperthermia system in different heating temperature(39°C to 44oC, 30min) and different heating time(42°C, 15min to 75min) respectively(each group has 3 nude mice).Then to obtain the treated tumour specimen and prepare tumour cell suspension cultivated with 9 kinds of chemotherapeutics (CDDP, MTX, PTX, BLM, VCR, THP, 5-FU, HCPT, VM-26,to use the 0.9% saline as control drug). To detect the average suppression rate of the treated cells in each group by the MTT assay, which reflects the sensitivity to the chemotherapeutics. Last to analysis the differences between the treated group and control group by SAS6.12 statistics software.

**Results**

There were no significant differences in the chemosensitivity to the 9 kinds of drugs between the Tca8113 cells in the control group and the 39°C-treated group, or among the 41 to 44°C-treated groups, but existing significant difference between the 40°C-treated group and the 41or 42°C-treated group. In the heating-time grads test, there were no significant differences in the chemosensitivity to the 9 kinds of drugs between these three pairs of group (the control group and the 15min-treated group, the 30min-treated and the 45min-treated group, the 60min-treated and the 75min-treated group), but there were significant differences between the 30min-treated or the 45min-treated group and the 60min-treated or the 75min-treated group.

**Conclusions**

Ultrasound hyperthermia performed in 40~44°C for 30min or in 42°C for 30 ~ 45 minutes can improve the chemosensitivity of Tca8113 cells significantly, which confirms the rationale of synchronous combination of hyperthermia and chemotherapy from a chemosensitivity point of view.

### P10 Focused ultrasound-induced blood–brain barrier opening to stimulate gene expression and treatment as a therapeutic strategy in Parkinson's disease model

#### Chung-Yin Lin, Han-Yi Hsieh, Kuo-Chen Wei, Hao-Li Liu

##### Chang Gung University, Taoyuan, Taiwan

**Objectives**

Parkinson’s disease (PD) is a common neurodegenerative disorder with selected loss of middle brain dopaminergic neurons, however, blood–brain barrier (BBB) is the major drawback encountered for effectively delivering drugs/genes to the brain. In this study, we propose a reliable strategy to synergistically apply FUS-BBB opening for the non-invasive and targeted delivery of non-viral genes into the PD mouse model for therapeutic purpose.

**Methods**

In this study, we studied a novel gene-liposome system, in which the biotin-conjugated liposomes are designed to carry plasmid DNA (pDNA, containing both green fluorescent protein (GFP) reporter gene and glial cell-derived neurotrophic factor (GDNF) therapeutic gene) and avidin-conjugated targeting microbubbles (MBs) to form a liposomal-plasmid DNA microbubbles (LP-MBs). Eight-week-old Balb/c male mice, each weighing about 25 g, were used for all experiments. Mice (n=15 per group) received the intraperitoneal injection of MTPT-HCl (40 mg/kg in saline once a day for 15 days at 3 weeks interval, and were killed at selected times 0–21 days after the last injection. Control mice received saline only. Pulsed FUS exposure was delivered to induce BBB opening (500 kHz, burst length = 10 ms, 1% duty cycle, PRF = 1 Hz). The longitudinal expression of GFP and GDNF were quantitated via an Elisa protein Assay Kit. The gene expression level was confirmed via immunoblotting, and histological staining was used to identify transfected cells via fluorescent microscopy.

**Results**

Animals with FUS treatment showed significant promotion of pDNA release into the striatum and demonstrated enhanced expression of genes upon sonication with FUS-BBB opening, while both the GFP and GDNF protein expression were successfully measured via Western blotting and Elisa Assay Kits. The transfection efficiency was higher payloads of pDNA resulted in a higher transfection rate via FUS-BBB opening. The treatment was higher efficacy than carrier only, FUS only. Immunoblotting and histological staining confirmed the expression of reporter genes and therapeutic genes in neuronal cells.

**Conclusions**

This novel gene delivery system promises to provide more effective ultrasonic therapy and neurodegenerative treatment and has the potential to achieve non-invasive gene delivery for treatment of Parkinson's disease.

### P11 Impact of stiffness in ovarian cancers presenting the fibrosis signature

#### Camille Garnier^1,3^, Gilles Renault^2^

##### ^1^Université Paris 7, Paris, France, ^2^Institut Cochin, Paris, France, ^3^Institut Curie, Paris, France

**Objectives**

Epithelial ovarian cancers (EOC) are still today among the most aggressive tumours, making them the fifth causes of cancer death in women in western countries. In the perspective of better stratifying patients and deciphering the mechanisms that govern tumour progression and treatment failure, the "Stress and Cancer" laboratory recently discovered a dual molecular signature, associated to "Oxidative Stress Response" and "Fibrosis" processes. In all datasets studied so far, this signature predicts a partial debulking, a bad response to chemotherapy, and a poor survival for Fibrosis EOC patients (Mateescu, Nature Medicine, 2011: Batista, IJBCB, 2013). In this context, we aim at characterizing the mechano-molecular properties involved in the aggressive development of the "Fibrosis" tumours.

**Methods**

- GSEA analysis on patient transcriptomic datasets;
- Shear Wave Elastography on Patient-Derived-Xenografted mouse models;
- Immunohistological analysis (mono and tri-colorations);
- RNA and protein tumour extracts for qPCR and Western Blotting;
- Cell culture in vitro (spheroids or monolayers) on matrices recapitulating the tumour stiffness range.

**Results**

- By GSEA analysis on 3 patient transcriptomic datasets, we found in "Fibrosis" tumours, an activation signature of YAP and TAZ, two major mechano-transducers, suggesting that mechanical inputs such as stiffening can occur in Fibrosis EOC.
- In order to assess the tumour stiffness in vivo, we then applied a recent and innovative technic, called Shear Wave Elastography, on "Fibrosis" PDX (patient-derived xenografted) mouse models. We found a high correlation between tumour size and tumour stiffness (varying from 10kPa up to 70kPa).
- In addition, immunohistological analysis revealed that the advanced stiff tumours are characterized by a global stromal accumulation and collagen enrichment at the periphery, creating a fibrillar ring.
- To characterize the molecular pathways impacted by the tumour stiffening, we further performed RNA and protein extracts from soft versus stiff tumours. The YAP/TAZ target genes were found up-regulated by stiffness, and we also observed a specific activation of the MEK pathway in stiff conditions.
- We confirmed these data in vitro in a system recapitulating the tumour stiffness range.

**Conclusions**

In conclusion, our results suggest that the "Fibrosis" signature identifies one subgroup of EOC tumours that is highly sensitive to stiffness variations, as detected by Shear Wave Elastography, through the YAP/TAZ and MEK pathway. Moreover, the stromal organization and more precisely, the collagen deposition could be an important driver of the tumour stiffening. That paves the way to a better understanding of mechano-transduction processes in "Fibrosis" ovarian patients, which is essential for expanding possible therapeutic options.

**Fig. 149 (abstract P11).**
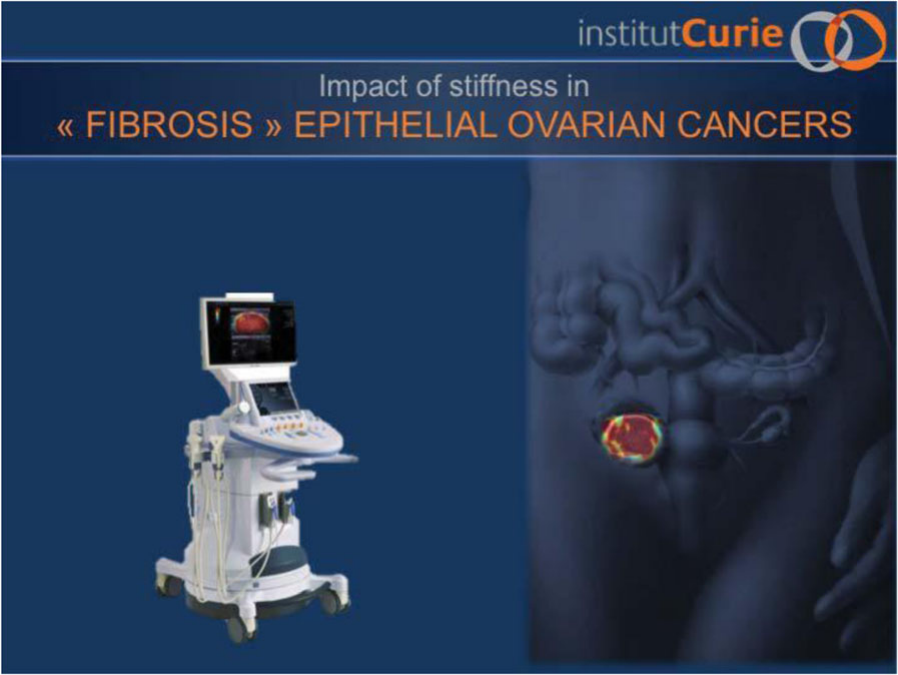
See text for description

### P12 Development of a tissue-mimicking rib phantom for MR-HIFU therapy

#### Navid Farr^1^, Ari Partanen^2^, Ayele H. Negussie^1^, Andrew Mikhail^1^, Reza Seifabadi^1^, Emmanuel Wilson^3^, Avinash Eranki^3^, Peter Kim^3^, Bradford Wood^1^

##### ^1^National Institutes of Health, Bethesda, MD, UAS, ^2^Clinical Science MR Therapy, Philips, Andover, MA, USA, ^3^Children's National Medical Center, Washington, DC, USA

**Objectives**

High intensity focused ultrasound (HIFU) enables highly localized, non-invasive thermoablation of various solid tumours. In the treatment of hepatic tumours the transmission of sufficient energy through the ribcage, while minimizing heating of the ribs and adjacent tissues, remains a problem. Several research groups have investigated the theoretical and experimental feasibility of transcostal HIFU ablation. Validation of theoretical models in controlled experiments using materials with similar shape and boundaries to human ribs may aid in clinical translation of HIFU for the treatment of upper abdominal tumours. The objective of this study was to develop and produce a tissue-mimicking gel phantom with embedded, 3D-printed rib mimics for the evaluation of heating in bone and adjacent tissues following transcostal HIFU.

**Methods**

A 3D-printable ribcage model was designed based on volume rendering of ribs from computed tomography (CT) scans of an adult male. The rib mimic was manufactured using a 3D printer (Connex 500, Stratasys Ltd., Eden Prairie, MN, USA) and a UV-curing photopolymer (VeroWhite Plus®, Stratasys Ltd.) with similar acoustic impedance and attenuation to human ribs (a = 3.0 dB/cm @ 1 MHz, c = 2370 m/s, rho = 1170 kg/m^3^). The section of rib was selected based on its relevance to MR-HIFU liver therapy. The 3D-printed rib mimic was fixed in a custom holder within a cylindrical plastic mold of 14 cm in diameter. Tissue-mimicking polyacrylamide gel containing thermochromic ink (color change temperature threshold of 60 °C), silica particles, and bovine serum albumin (BSA) was poured to fill the plastic mould, and let to solidify around the ribs at 4°C. Subsequently, the phantom was positioned on a clinical HIFU table (Sonalleve V2, Philips, Finland), maintained at 37 °C using a heated water bath, while acoustic coupling was achieved using degassed water. Sonications at 1.2 MHz frequency and 80W acoustic power for 60 seconds were performed under Magnetic Resonance Imaging (Achieva 1.5T, Philips, Best, the Netherlands) guidance. Prior to sonication, the rib mimic was visualized on T2-weighted MRI as a hypointense region, and manually segmented. 8 mm target locations 3 cm behind the ribs were sonicated with all elements enabled and the resulting temperature elevation within the target region and on the rib mimic measured. Then, the ribs were projected onto the transducer surface by ray tracing from the focal point. Power for the transducer elements in the “shadow” of the ribs were set to zero, while power for the other, remaining elements was compensated accordingly. MRI magnitude and phase images during sonications were acquired in 3 slices automatically placed at the focus in coronal and sagittal planes and one slice coincident at the rib-mimic plane, and temperature elevations were calculated using the proton resonance frequency shift (PRFS). Moreover, the thermochromic phantom enabled monitoring of the distribution of temperature changes in the area surrounding ribs and target location.

**Results**

Real time temperature changes in the area surrounding rib-mimic and target location were monitored successfully using MRI. Qualitative and quantitative assessment of temperature rise on those locations was obtained by colour change. The overall temperature rise in the area surrounding the 3D printed ribs was reduced as a result of selective deactivation of transducer elements whilst maintaining equivalent focus quality.

**Conclusions**

A 3D printed rib phantom embedded in thermochromic gel with realistic rib array location was developed and validated for use in MR-HIFU therapy. The potential of focusing techniques for minimizing the temperature rises on the surface of the rib-mimics during trans-costal sonication can be demonstrated by observation of heating patterns in the phantom. This phantom may be useful for thermal therapy characterization, and in comparison of numerical models with experimental data.

### P13 MRI based detection of pulsed HIFU induced cavitation

#### Dennis Lübke^1^, Jürgen W. Jenne^1,2^, Peter Huber^2^, Matthias Günther^1^

##### ^1^MRI Physics, Fraunhofer MEVIS, Bremen, Germany; ^2^Deutsches Krebsforschungszentrum, Heidelberg, Germany

**Objectives**

Besides acoustic radiation force during HIFU treatment, the detection of cavitation is a possible candidate for non-thermal dose standard of focused ultrasound setups. The detection and quantification of cavitation currently involves custom-made devices which often require complex setups or special measurement chambers and limit the detection of cavitation to laboratory environments. As the occurrence of cavitation is expected to cause changes in MR susceptibility, we propose here a method for passive and non-invasive detection of cavitation by using a multi-contrast segmented EPI sequence and evaluating the changes in T2* decay in MR images in the focal spot during high intensity ultrasound pulses. The aim of this study is to evaluate the possibilities for cavitation detection in a standard clinical MRI scanner.

**Methods**

**Fig. 150 (abstract P13).**
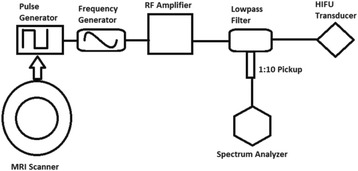
HIFU setup and signal path diagram

HIFU setup: A MR-compatible fixed focus HIFU setup (Siemens, 1.7 MHz, 60 mm Ø, f = 68 mm) with a calibrated signal chain (function generator, amplifier and adaptation (see Fig. 150)) and a dedicated basin to hold both the transducer and the phantom is used to generate series of varying short high intensity HIFU bursts (between 10 μs and 10 ms). According to calibration data, the maximum positive peak pressure is approximately 32 MPa. A real-time spectrum analyser is used as a passive detector to capture the occurrence of harmonics of the base frequency in the reflected signal.

Sample preparation: Three different phantoms made of gelatine and evaporated milk have been used as sonication targets [2]. The gels were made with distilled but non-degassed water to increase the probability for cavitation.

**Fig. 151 (abstract P13).**
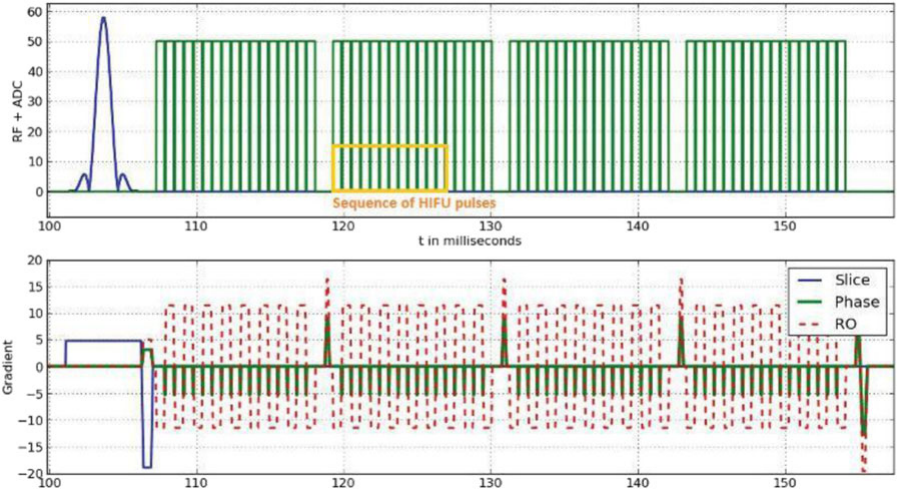
Multi-contrast EPI sequence diagram (4 contrasts with long HIFU burst)

Multi-contrast EPI sequence with external trigger signal: A segmented EPI sequence has been modified to allow the acquisition of multiple contrasts per image. Acquisition of up to 12 contrasts is possible depending on TE and EPI factor. As a trade-off between acquisition time, duty-cycle, echo time and in-plane resolution the following acquisition parameters have been used: FOV: 140x140 mm, FA: 80°, TR: 5000 ms, TE: 16 ms, Matrix: 128x128, Voxel size: 1.1x1.1x2.5 mm, EPI-factor: 16, 10 contrasts per acquisition. The long repetition time TR has been chosen to avoid heating of the phantom in the focal spot due to subsequent sonications. The sequence contains functionality to submit a trigger signal of 10 μs at the beginning of an arbitrary contrast ADC to allow precise triggering of the HIFU bursts (see Fig. 151). Shifting the trigger signal to different contrasts is expected to also shift the time of the expected change in susceptibility due to cavitation or other HIFU effects. Experiments were executed on a Siemens Skyra 3T MRI scanner (Siemens Healthcare AG, Germany).

To ensure proper placement of the multi-contrast EPI slice, a gradient echo thermometry measurement is executed in transversal and coronal orientation to locate the focal spot during a continuous 10 s sonication. A single transversal slice intersecting the centre of the focal spot is used for the multi-contrast EPI sequence with the same FOV and matrix size as for the transversal thermometry to use the same ROI of the focal spot for analysis.

A baseline approach is used to compare the effects that occur during sonication to a reference state. A new reference image was acquired after each fifth image with sonication. Sonication took place in two different focal spots per phantom.

For comparability, the HIFU burst was triggered at varying contrasts to test the influence of MR signal strength and to validate that the effects only occur during the HIFU bursts.

Multiple sonication strategies have been tested: a) continuous sonication over 1-10ms per TR and b) short pulses of 10 μs length with at least 490 μs pause between each pulse to avoid heating of the phantom. The number of HIFU pulses was set such that the series of pulses was only active during the selected contrast.

**Results**

Evaluation has been done by calculating the T2* fit over a 3x3 and 1x1 ROI in the focal spot for both the reference images and those with sonication. While the input values for the fitting show clear differences for certain contrasts, only a subtle change in the T2* fit is visible. For better visibility of the effect, a windowed fitting over certain intervals has been chosen which shows a more significant decrease in T2* only during the HIFU bursts.

**Fig. 152 (abstract P13).**
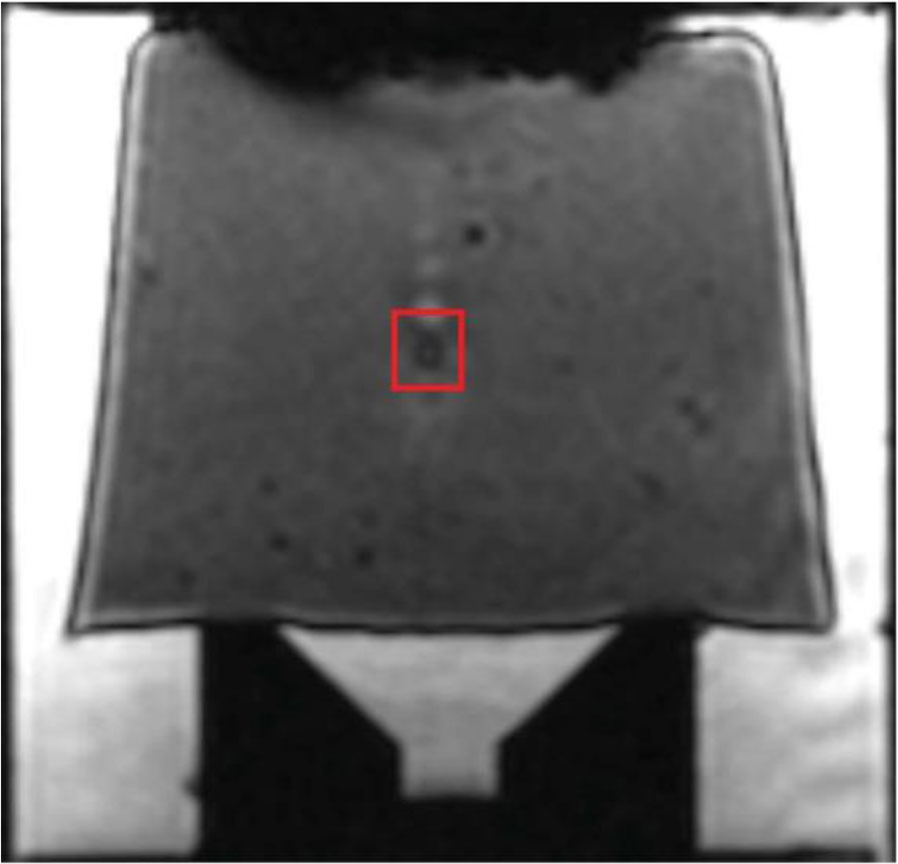
Visible HIFU effects after contrast 5

Sonications with more than 30 MPa pressure show visible echoes and harmonics in the spectrum analysis for all sonication strategies. Visible effects in the MR images could only be reproduced when using continuous sonications of at least 5 ms rather than the series of 10 μs pulses (see Fig. 152). The change in T2* decay could only be observed after playing the HIFU burst. All visible effects were fully reversible. No permanent change in the phantom could be detected when comparing subsequent reference images after multiple sonications.

While the input values for the fit show a clear difference for the ROI between reference and sonication, the fitted T2* values were only marginally different unless using the segmented fitting approach. Due to the microscopic extent of cavitation, evaluating the 1x1 ROI yields more distinct results. Best results could be obtained using an EPI factor 16 and 128x128 matrix size, short TE and 10 contrasts.

**Conclusions**

**Fig. 153 (abstract P13).**
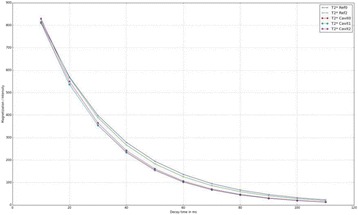
T2* evaluation with fit for reference and sonication over multiple segments shows visible T2* reduction for sonication

While all sonication strategies with a peak pressure above 30 MPa showed visible echoes and harmonics in the spectrum analysis, visible effects in the MR images only occurred with continuous sonications >= 5 ms. This may be caused by the rather limited power of the HIFU transducer used in the experiments. This also confirms the cavitation threshold described in [1]. Taking into account that cavitation only exists for microseconds within a very limited space, reducing the voxel size will help to achieve more unambiguous results and to avoid partial volume effects. We have successfully shown the detectability of the effects of short HIFU bursts in MR images using a multi-contrast EPI sequence. Despite the limited power of the HIFU transducer used, magnetic susceptibility is reduced only during the HIFU pulses and can be seen in the T2* fit (see Fig. 153). Further experiments will be executed with another transducer (Imasonic, France) with lower frequency and more power to exceed the cavitation threshold.

### P14 Comparison of MR thermometry and HIFU simulations in a calibrated setup

#### Dennis Lübke^1^, Joachim Georgii^1^, Michael Schwenke^1^, Caroline v. Dresky^1^, Julian Haller^2^, Matthias Günther^1^, Tobias Preusser^1^, Jürgen W. Jenne^1^

##### ^1^Fraunhofer MEVIS, Bremen, Germany, ^2^PTB Braunschweig, Braunschweig, Germany

**Objectives**

We present the results of a study to compare MR thermometry measurements using a calibrated HIFU setup with simulation results based on the free-field parameters that were assessed during the calibration process. The aim of this study is to demonstrate the coherence between measurements and simulation in order to validate the outcome of thermometry measurements through simulation. Further applications can be established in the process of HIFU dosimetry when using standardized methods for HIFU setup and gel calibration in order to predict expected measurement results from simulation. Another potential benefit of the simulation is to correct possible partial volume effects that can occur during MR thermometry due to imperfect slice orientation.

**Methods**

HIFU setup: A MR-compatible fixed focus HIFU setup (Siemens 1.7 MHz, 60 mm Ø, f = 68 mm) with a calibrated signal chain (function generator, amplifier and adaptation) and a dedicated basin to hold both the transducer and the phantom are used to sonicate custom made gelatine and agar based phantoms at varying power levels (between 6 and 20 W acoustic power) and sonication times (between 5 s and 20 s). State of the art calibration of the system includes acoustic power for continuous sonication, free-field line-scans for peak pressure and 2D near-field maps that allow to simulate sound propagation.

Sample preparation: Phantoms made of agar/gelatine (bloom value 225) and evaporated milk (50%) were used as sonication targets [1]. The gels are made with distilled and degassed water. The advantage of the gelatine phantoms is better homogeneity whereas the agar based gels have a higher melting point and allow longer sonications at higher power levels.

EPI thermometry sequence and measurement protocol: To achieve high temporal resolution a proton resonance frequency sensitive (PRF) EPI gradient echo MRI sequence is used for the MR thermometry measurements, allowing acquisition times of 110 ms per slice. The following parameters are used for image acquisition: TR: 15 ms, TE: 7.5 ms, Flip Angle: 12°, FOV: 140x140 mm, Matrix Size: 128x128, Slice Thickness: 2.5 mm, Voxel Size: 0.9 x 0.9 x 2.5 mm, EPI factor: 13. Thermometry measurements are executed with different numbers of slices and varying slice orientations to investigate reproducibility and partial volume effects. A single measurement consists of acquisition of at least 8 reference images (until a magnetic steady state is established) and subsequent acquisition of up to 500 images capturing the heating and cool-down in the focal spot. Sonication duration is varied from 5 to 20 s. Since in the transversal slices the extent of the focal spot cannot be determined, additional measurements with three coronal slices in parallel orientation to the sound direction were executed to obtain results (with possible partial volume effects) that can be compared to the simulation as discussed in the next chapter.

HIFU simulation methods: The HIFU simulation is based on the geometric arrangement of the transducer and phantoms, which is obtained from MR images. The simulation is performed in two steps. First, the pressure field is computed based on the acoustic power of the transducer and the known acoustic parameters of water as well as the phantom using the hybrid angular spectrum method. Second, the temperature field is computed by solving Pennes bioheat equation using the known thermal material parameters for the phantom (attenuation = 0.53 dB/cm/MHz, thermal conductivity = 0.55 J/(m*s*K)). Finally, the high resolution temperature grid is resampled to the thermometry image grid by averaging over each thermometry voxel to allow for comparison of the data.

**Results**

**Fig. 154 (abstract P14).**
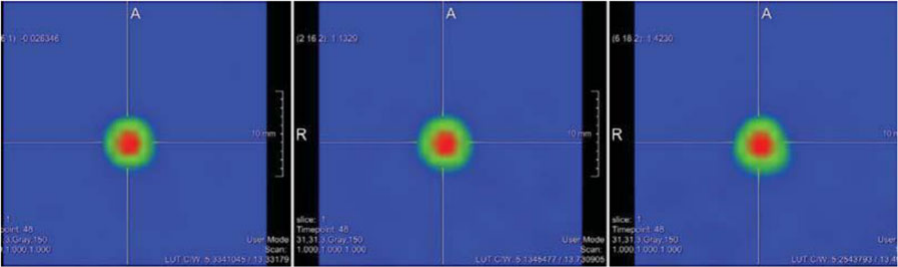
Transversal slice intersecting the focal spot (three sonications). Figure 155: Reproducibility test: Three subsequent sonications and cool-down (same voxel used for evaluation for each session)

**Fig. 155 (abstract P14).**
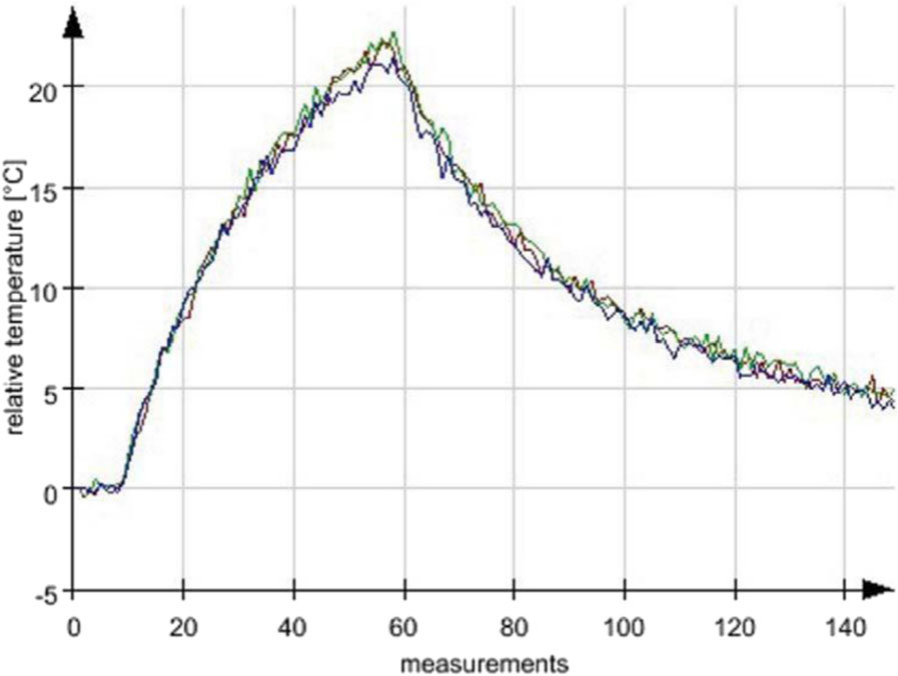
Reproducibility test: Three subsequent sonications and cool-down (same voxel used for evaluation for each session)

**Fig. 156 (abstract P14).**
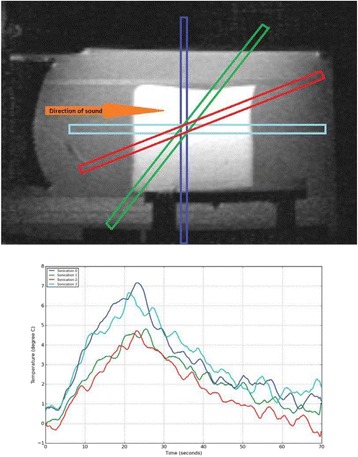
Possible influence of partial volume effects with imperfect slice orientation (top image shows the different slice orientations, bottom image shows temperature development in the same colors as the slices used above, voxel with maximum temperature shown)

MR thermometry results: Best results could be obtained with a single transversal slice orthogonal to the direction of sound, intersecting the focal point in its center. This also minimizes the influence of partial volume effects and reliably provides reproducible results over multiple measurements. Evaluation of the phase images obtained from PRF evaluated MR thermometry shows reproducible results over multiple measurements (see Figs. 154 and 155). Stepwise rotation of the thermometry slice from transversal to coronal orientation (in 30° steps) shows visible irregularities and lower maximum temperature (see Fig. 156) over time that might be caused by partial volume effects.

**Fig. 157 (abstract P14).**
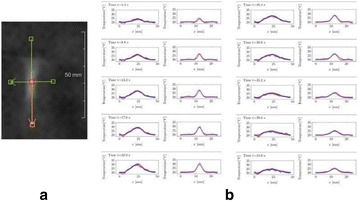
Comparison of MR thermometry measurements and simulation results over the heating and cooling phase of a sonication. **a** Geometrical setting of readout lines along and across the focus position, **b** temperature plots at different time-points comparing MR thermometry measurements (*red*) and simulated temperature (*blue*). For each time-point the left plot shows the data readout along the focus, respectively the right plot shows the data across the focus

Comparison of MR thermometry and simulation results: Fig. 157 shows the comparison of the MR measurements (red) and the simulation (blue) at two cuts (left: transversal, right: coronal) through the focal point at different time points of a sonication during heating and cooling phase. It can be seen that in both cutting planes, the temperature profiles match quite well. It is worth noting that the maximal temperature in the focal spot is higher, but due to the limited spatial and temporal resolution of the MR thermometry the temperature does not show up in the measurements.

**Conclusions**

In this study we have shown the coherence of MR thermometry and HIFU simulation by using as input only the free-field parameters of the HIFU setup and the known parameters of the phantom material. The simulation methods provide a good approximation of the measurements with the additional benefit of being immune to partial volume effects that can occur with imperfect MR slice orientation. Using standardized phantoms with known acoustic and thermal parameters, the HIFU simulation could serve in the process of HIFU dosimetry and calibration.

Our method is an effective means to present highly resolved temperature data from the simulation that are in accordance with the (coarse) measurements and can even capture regions where no thermometry slices have been measured (prediction). With proper assessment of the temperature deviation depending on the slice orientation it should be possible to derive a correction method to compensate for the influence of partial volume effects. The quantification of the temperature difference depending on the slice alignment is left as future work.

*Acknowledgement*

*Parts of the work were supported by EMRT HLT03 DUTy project. The EMRP is jointly funded by the EMRP participating countries within EURAMET and the European Union.*

### P15 Volumetric and thermal characterization of boiling histotripsy lesions based on a clinical MR-HIFU system

#### Avinash Eranki^1,2^, Navid Farr^2^, Ari Partanen^3^, Pavel Yarmolenko^1^, Ayele H. Negussie^2^, Karun Sharma^1^, Haydar Celik^2^, Bradford Wood^2^, Peter Kim^1^

##### ^1^Children's National Medical Center, Washington, DC, USA; ^2^National Institutes of Health, Bethesda, MD, USA; ^3^Clinical Science MR Therapy, Philips, Andover, MA, USA

**Objectives**

High intensity focused ultrasound (HIFU) is a non-invasive therapeutic technique used traditionally to destroy tissue via thermocoagulation. Alternate HIFU methods of boiling and cavitation histotripsy have been developed with the goal of mechanically disrupting tissue without inducing substantial thermal effects. Boiling histotripsy (BH) generates a millimetre-size boiling bubble, causing instantaneous tissue emulsification within the focal region. BH tissue emulsification is aided by waveform non-linearity at high acoustic pressures leading to a shock wave formation within the focal zone. This results in rapid boiling of tissue in a few milliseconds at the focal zone. The BH mechanism differs from traditional HIFU ablation techniques, since it causes little or no thermal damage outside the focal zone. Thus, nearby critical structures may be spared, potentially increasing the clinical applicability or safety of HIFU, especially when heat-sensitive critical structures are close to target region. This work characterized the effect of BH sonication parameters on the resulting volumetric lesions in a tissue-mimicking phantom. This initial study explores a wide range of sonication parameters, such as peak acoustic power, total sonication time, pulse width, and pulse repetition frequency (PRF) in gel phantoms. The study thus provides a reference point for future BH studies in tissue-mimicking phantoms, as well as for ex vivo and in vivo studies. This work also utilizes a clinical MR-HIFU system and can thus easily be translated and repeated at different research sites and in the clinic.

**Methods**

**Fig. 158 (abstract P15).**
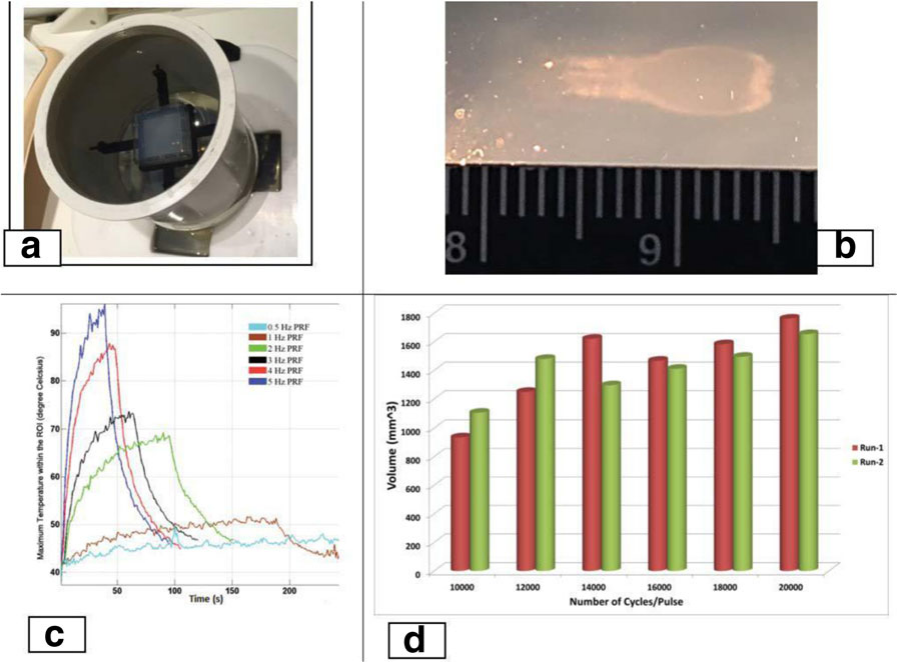
**a** MR-guided histotripsy experimental setup. The setup consisted of a polyacrylamide gel placed in a 3D-printed holder in a degassed water bath (white tank). This entire setup was placed over the HIFU transducer. **b** typical boiling histotripsy lesion produced using the clinical MR-HIFU system with 600 W, 1 Hz PRF and 15000 cycles/pulse. The lesion has a characteristic tadpole-looking shape. **c** maximum temperature value in each ROI was plotted as function of time for all different PRFs. An exponential temperature increase, but the time constants and decays are different between 0.5 Hz (cyan curve) and 5 Hz (dark blue curve) PRF. **d** Lesion volumes were somewhat reproducible and showed some consistency between the two repetitions. Lesion volumes substantially increased between 10000 cycles/pulse and 20000 cycles/pulse

Sonications were performed on a clinical MR-HIFU system (Sonalleve V2, Philips, Vantaa, Finland) with a 256-element, phased-array transducer (focal length = 14 cm, frequency 1.2 MHz). Polyacrylamide gel phantoms (7% w/w) were prepared using deionized and degassed water. Phantoms were placed in a custom 3D-printed holder within a water bath (Fig. 158a). MR imaging was used to obtain images of the phantom and to plan the sonications. Locations 30mm deep within the phantom were sonicated to produce a pattern consisting of 27 lesions separated by 1mm, in a 3 x 3 x 3 matrix. To relate the experiments conducted at room temperature to in vivo studies, reference temperature for MR-thermometry was set to 37.5°C, and temperature was calculated as a relative change. Temperature elevation and morphology of BH lesions were characterized across varied sonication parameters: peak acoustic power (500 to 650 W in steps of 50 W), pulse length (10,000-20,000 cycles/pulse in steps of 2000 cycles/pulse), total sonication time (136 to 820 s in increments of 137 s), and PRF (0.5 to 5 Hz in 0.5 Hz increments). For lesions made with varying peak acoustic power, the PRF was constant at 1Hz and pulse length at 15000cycles/pulse. Similarly, when the number of cycles/pulse varied, the peak acoustic power was kept constant at 600 W at 1 Hz PRF. While the PRF was varied, the peak acoustic power was set to 600 W and number of cycles/pulse was constant at 16000. Real-time MR thermometry was performed during sonication using an FFE-EPI sequence with three coronal slices centred on the target region. Parameters were: FOV=160x121 mm, voxel size=2.5x2.5x6 mm, EPI factor=5, TE=16 ms, TR=25 ms, flip angle=20°, dynamic scan time=1.8 s. Post-sonication, the lesions were individually scanned using a clinical diagnostic ultrasound system (iU22, Philips, Bothell, WA, USA) equipped with a 3D transducer (X6-1). Lesion volumes were computed following semi-automatic segmentation (TurtleSeg, The University of British Columbia, Canada). Sonications were performed in two independent sets of measurements (n=2). Temperature quantification was performed using a region of interest (ROI) within the centre MR thermometry slice, centred on the BH lesion. The maximum temperature value in each ROI (as a function of time) was plotted to observe and quantify differences between test parameters.

**Results**

Boiling histotripsy sonications produced ‘tadpole’ –shaped lesions in the gel phantoms with all tested parameters (Fig. 158b). Increasing the peak acoustic power from 500 to 650 W slightly increased the lesion volumes from 1025 to 1335 mm^3^. Interestingly, the lesion volumes increased substantially from 930 to 1762 mm^3^ by changing the number of cycles per pulse from 10000 to 20000 (Fig. 158d), while other parameters were kept constant as described in the methods section. A similar change in lesion volumes was observed when sonication time increased, with lesion volumes increasing from 934 to 1760 mm^3^. However, varying PRF from 0.5 to 5 Hz resulted in only a slight increase in volume from 1571 to 1838 mm^3^. Lesion shapes also varied from being 27 independent point lesions to a completely connected, single large lesion for different sonication parameters. For example, sonication time of 137 s yielded a lesion that consisted of 27 separate sub-lesions, while 820 s produced a contiguous single, large lesion. These differences could potentially have very different bio-effects in-vivo and therapeutically. Maximum temperature within each ROI increased until the end of the sonication. Peak relative temperature increases were higher for increasing powers and PRF (Fig. 158c), with temperatures increasing from 47 to 96°C, when the PRF was adjusted from 0.5 to 5 Hz with an exponential increase during the sonication period.

**Conclusions**

In this preliminary work a HIFU method to induce mechanical damage in tissue is examined in vitro. It was shown that BH can produce mechanical damage in a tissue mimicking phantom with varying degrees of temperature elevation, depending on the sonication parameters. While all the lesions had sharp boundaries, some sonication parameters produced one large lesion and others produced several independent lesions with the same sonication protocol. This could have profound effects in-vivo, especially while studying immune effects due to BH. Boiling histotripsy lesions in phantoms were repeatable in terms of their location and volumes. In some applications, BH could be better than traditional HIFU ablation due to its ability to produce lesions with sharp boundaries while limiting temperature elevations. The use of a commercial, clinical HIFU system to produce BH lesions may aid in clinical translation of this methodology compared to custom built, preclinical HIFU systems.

### P16 Non-invasive stimulation of mouse brain with high frequency (5MHz) focused ultrasound

#### Guofeng Li, Weibao Qiu, Hairong Zheng

##### Shenzhen Institutes of Advanced Technology, Chinese Academy of Sciences, Shenzhen, China

**Objectives**

Ultrasound has become an efficient approach for non-invasive brain stimulation. Currently low frequency ultrasound (<1 MHz) are preferable as its low ultrasonic attenuation when pass through the human skull. However, the stimulated area in the brain is hard to be specific located since the focal zone is relatively large when using low frequency ultrasound. It is important especially in mouse study due to small brain size. According to the principle of ultrasound engineering technique, higher ultrasound frequency has the ability to support smaller focal zone, which ultimately minimize the stimulation area. This study examines the feasibility of using higher frequency, i.e. 5 MHz, focused pulsed ultrasound to evoke motor responses of mouse.

**Methods**

5 BALB/c mice, 8 weeks old, 20 g (+/−25%) in weight were used. Mouse was anesthetized by intraperitoneal injection of chloral hydrate and fixed on a mouse stereotax (68028, RWD Life Science Co.). 5MHz ultrasonic pulses (80 circles/pulse, pulse repetition frequency of 1.2 kHz, 100 pulses) were excited every 1s, 3s, and 5s, by a single element transducer (5 MHz centre frequency, IL0506HP, Valpey Fisher), two function generators (AFG3102, Tektronix), and a RF power amplifier (AR150A100B, Amplifier Research). The transducer could be moved above the skull of mouse with 0.1 mm steps in three dimensional. The motor movements of mice were captured by camera (HD1080P, Aoni Ltd., Shenzhen). Electromyography (EMG) responses collected from the tail and forelimbs muscles were used to monitor the motor potentials evoked by motor cortices. Ultrasonic attenuation coefficients of skulls in high frequency were measured and calculated using 3D acoustic intensity measurement system (SN2010, Precision Acoustics).

**Results**

Our results indicate that 5MHz focused ultrasound can efficiently penetrate through skull of mouse and stimulate motor cortex to evoke motor potentials and movement responses, thus allow for more precise stimulation on brain of mouse. Attached picture shows the motion response of a mouse tail and the EMG signal acquired from the tail. The compensated acoustic intensities are similar (difference <5%) between 1 MHz and 5 MHz ultrasound. The peak EMG amplitude increases gradually when increasing the acoustic intensity with 5 MHz ultrasound. The acoustic intensities range from 130 mW/cm^2^ to 230 mW/cm^2^. The equivalent diameter of ultrasound stimulus with 5 MHz (0.29±0.08 mm) is significantly smaller than that of 1 MHz (0.83±0.11 mm). The quantity of successfully evoked waveforms of 1 MHz ultrasound is larger than that evoked by 5 MHz. Moreover, the EMG response latency of 1 MHz ultrasound is greatly larger than that of 5 MHz.

**Conclusions**

This study examined the feasibility and effectiveness of using higher frequency (5 MHz) ultrasound to evoke motor responses of mice. The results show that 5 MHz ultrasound can efficiently penetrate through mouse skull and stimulate brain to evoke motor potentials and movement responses. It provides a reduced focal region, which thus offers an improved anatomical specificity in neuro-stimulation in a non-invasive manner.

**Fig. 159 (abstract P16).**
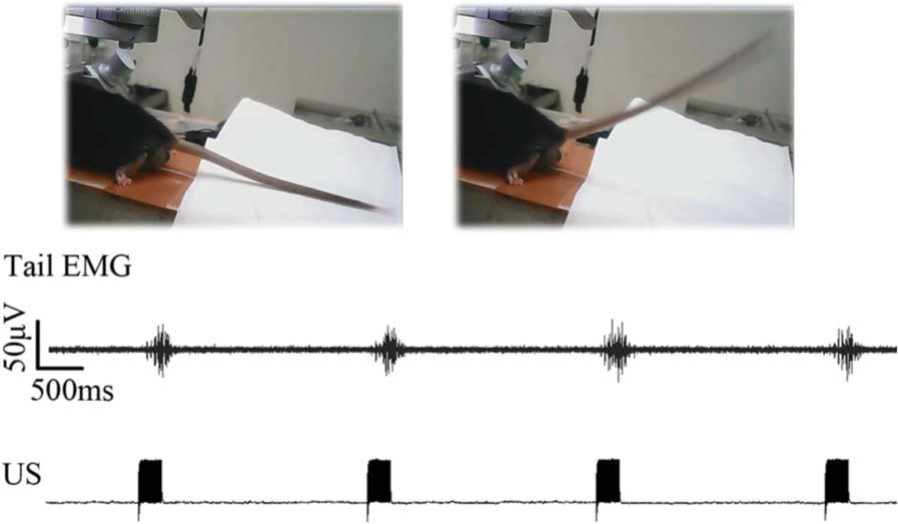
See text for description

### P17 Neuro-navigation-guided focused ultrasound-induced blood–brain barrier opening: feasibility in penetrating through human skull

#### Meng-Yen Tsai, Po-Chun Chu, Hao-Li Liu

##### Department of Electrical Engineering, Chang Gung University, Taoyuan, Taiwan

**Objectives**

Focused ultrasound (FUS) exposure with the presence of microbubbles has shown its promise in temporally and targeted open the blood–brain barrier (BBB), and brings potential in delivering therapeutic agents into the brain for CNS disease treatment. Feasibility in using neuronavigation system to guide the FUS-BBB procedure in large animals has been demonstrated successfully, and the guidance precision has been measured to be acceptably low. However, it is still unknown whether same precision and power compensation strategy is valid when FUS beam is intend to penetrate the human skull. The purpose of this study is to verify that using neuronavigation system combined with a prior treatment plan can successfully guide FUS-BBB opening with the consideration of involvement of human skull.

**Methods**

A human cadaver was employed to perform CT scan, and a treatment planning software was developed to simulate focal beam redistribution and pressure decay when transcranial FUS exposure were performed. During experiment, 4 groups of animals (n = 22) were conducted to verify the success of BBB-opening when incorporating with the in-prior treatment plan.

**Results**

The developed algorithm provides can well reconstruct the 3D human skull structure and perform reliable skull-insert pressure loss prediction. Data comparison shows that focal beam distortion and shifting can be well predicted. In animal experiments, we showed that success rate in inducing BBB opening in animal brain at the target position can be increased to 87% when compared to 0% in uncorrected experimental groups.

**Conclusions**

Neuronavigation guided FUS-BBB opening has the added advantage of not limiting neurosurgical intervention by MRI chamber space or non-magnetic properties. Combining neuronavigation guidance with the prior treatment planning information is feasible to precisely guide the FUS-BBB opening procedure.

**Fig. 160 (abstract P17).**
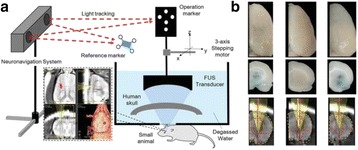
**a** Experimental setup of the neuronavigation-guided FUS-induced BBB opening in small animals when considering human skull cadaver insertion. **b** Representative comparisons of the FUS-induced BBB regions identified by *post mortem* observation of EB extravasations (upper: frontal view; middle: sectional view) as well as the real-time guided view via neuronavigation system during FUS exposure. (*left*) No skull; (*middle*) Skull insertion; (*right*) Skull insertion with compensation. Yellow = FUS energy target positions

### P18 The impact of skull temperature on focusing in transcranial MRgFUS

#### Taylor Webb^1^, Urvi Vyas^2^, Kim Butts Pauly^1,2^

##### ^1^Electrical Engineering, Stanford University, Palo Alto, CA, USA; ^2^Radiology, Stanford University, Palo Alto, CA, USA

**Objectives**

To measure the change in the speed of sound of skull bone in a pig model as a function of temperature and to use these values in simulation to predict if changes in speed of sound as a function of temperature could negatively impact treatment outcomes.

**Methods**

**Fig. 161 (abstract P18).**
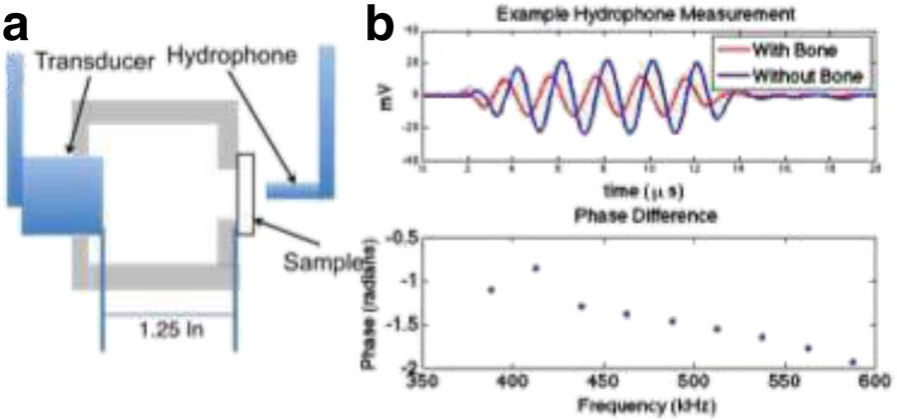
**a** Schematic of experimental setup. **b** Sample of measured signal as recorded by the hydrophone in the time domain (*top*) and the difference in phase (water – bone) as a function of frequency (*bottom*)

A holesaw was used to acquire a 0.5 inch diameter core from a dried, ex-vivo pig skull. The core was then cut and sanded further to remove trabecular bone and leave a smooth edge. The Fragment was attached to an aluminium plate that was fastened to an ultrasound transducer at the focal spot (Olympus, 500 KHz, 1 inch diameter, 1.25 inch focal length). Fig. 161a shows a schematic of the setup.

Acoustic measurements were acquired with a needle hydrophone (HNR-0500 Onda, Sunnyvale, CA), which was connected to an oscilloscope that digitized the data and passed it to a PC. Internal averaging in the oscilloscope was set such that each measurement was made 16 times and averaged.

Temperature was controlled using water heated in a separate water bath and circulated through the measurement tank. Measurements were performed at each temperature with and without the bone present. Temperature in the water was measured with an optical thermal probe and the temperature was allowed to equilibrate for one hour before measurements were taken.

Velocity measurements were repeated five times at each temperature. Each measurement was performed by acquiring acoustic recordings with the bone in place, removing the bone without disturbing the hydrophone, and then repeating the recordings without the bone.

Data Analysis: Phase velocity measurements were obtained by comparing the phases of the Fourier transform component nearest to 500 kHz as done by Fry and Barger [1]. The phase change is given by Φ = 2πω(d/cw-d/cb), where ω is the radial frequency, d is the width of the bone fragment, cw is the sound velocity in water, and cb is the sound velocity in the bone fragment. Solving for cb gives cb = 1/(1+Φcw/(2πdω)). Figure 161b shows example hydrophone measurements in the time domain and the phase difference, Φ, at frequencies surrounding 500 kHz.

Simulation: A CT of two patients (one “easy” and one “hard” as defined by the ratio of the temperature rise of the first sonication to the power of the first sonication) was segmented into water, skull, and brain. The skull was further segmented using the Hounsfield Unit Value and speed of sound and scattering values were assigned according to Vyas et al. [2].

Temperature simulation was done using a finite difference time domain implementation of the Pennes’ bioheat equation [3]. The beam pattern was recalculated once every second during the simulated sonication based on temperature changes reported by the bioheat simulation. In each skull, the acoustic power was set such that the temperature rise, assuming no variation in acoustic velocity, was 60°C.

All voxels in the skull were assumed to have the same absolute change in velocity with temperature as was measured in this fragment. This assumption should lead to an estimate of the worst-case scenario because, in the temperature range of interest, the velocity of sound in water and bone trend in opposite directions as a function of temperature. Therefore, in voxels composed primarily of trabecular bone, the overall change in velocity will be small compared to voxels composed primarily of cortical bone. Therefore assuming that all voxels segmented as bone change in the same way as cortical bone should overestimate the affect.

**Results**

**Fig. 162 (abstract P18).**
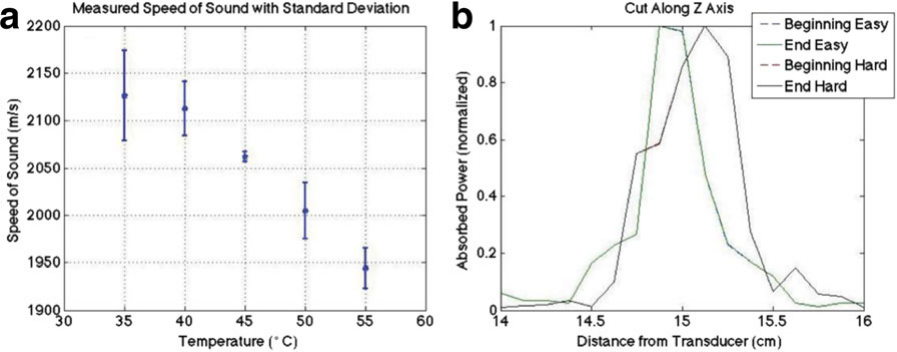
**a** Measured speed of sound as a function of temperature. Error bars represent standard deviation of the five measurements done at each temperature. **b** Change to the simulated focal spot shape and the simulated efficiency as a result of changes in the speed of sound in the skull. The plot shows a cut through the beam along the z-axis at the beginning and end of the sonication

Figure 162a shows the measured speed of sound as a function of temperature. Error bars represent the standard deviation of the five measurements acquired at each temperature. Fitting the data yields a slope of −9.5 m/s/C, a relatively small change.

Figure 162b shows results from simulation. The plot compares cuts along the z-axis of the absorbed power at the beginning of the sonication, before any skull heating has occurred, to the same cut at the end of the sonication. There is no appreciable change in the beam pattern or the power at the focal spot in either skull.

**Conclusions**

While the speed of sound in bone does have some dependence on temperature, this work provides evidence that it is not enough to significantly alter the focal spot during a sonication.

**References**

[1] Fry F and Barger J. *Acoustical properties of the human skull*. J. Acoust. Soc. Am., vol. 63, no. May 1978, pp. 1576– 1590, 1978.

[2] Vyas U, Ghanouni P., C. Halpern, Elias J, and Pauly KB. *Predicting Variation in Subject Thermal Response during Transcranial Magnetic Resonance Guided Focused Ultrasound Surgery: Comparison in Seventeen Subject Datasets*. Submitted, 2015. 1, no. 2, pp. 93–122, 1948.

### P19 *In vivo* and post-mortem brain analysis of diffusion tensor images and diffusivities – application and relevance to MR-guided focused ultrasound treatment

#### Matthew Walker^1,2^, Jidan Zhong^2^, Thomas Looi^3^, Adam C. Waspe^3^, James Drake^3^, Mojgan Hodaie^2,1^

##### ^1^Institute of Medical Science, University of Toronto, Toronto, Ontario, Canada; ^2^Krembil Research Institute, University Health Network, Toronto, Ontario, Canada; ^3^Centre for Image Guided Innovation and Therapeutic Intervention, Hospital for Sick Children, Toronto, Ontario, Canada

**Objectives**

Diffusion tensor imaging (DTI) is a valuable tool for localizing white matter fibres and measure metrics of diffusivity. Tractography algorithms can be used to visually reconstruct white matter fibres in order to delineate focused ultrasound (FUS) targets of interest. Animal models are useful in pre-clinical investigation stages of FUS application. However, in vivo studies can be logistically and financially challenging. Where possible, it would be desirable to use post-mortem animals prior to clinical testing of FUS in animals. We compared the imaging and diffusivity metrics of the body of the fornix in a young pig model. Specifically, we aimed to demonstrate that visualization of tracts post-mortem was feasible and compared to see which metrics of diffusivity were altered between in vivo and ex vivo models.

**Methods**

Six piglets were studied: three live, three *post mortem* (<24 hr), average weight 5.2 kg. T1 anatomical and diffusion-weighted imaging (DWI) scans were acquired. The DWI scanning parameters included 128 diffusion directions, 1.6mm isotropic voxel resolution, and diffusion weighting of b=800 s/mm^2^. Eddy current, motion, and field map corrections were performed. A one-direction reverse phase-encoded sequence was also incorporated for correcting diffusion gradient-associated image distortions. The T1 and DWI scans were co-registered for accurate anatomical localization. The fornix was chosen as a model white matter structure due to its central position within the brain and ease of visualization. Single-tensor tractography of the fornix was performed using 3D Slicer (version 4.4) using the following parameters in both living and post-mortem subjects: stopping fractional anisotropy (FA) value 0.05; step size 0.4 mm; seed spacing 0.2; stopping curvature 0.7. A region of interest (ROI) based analysis was performed in order to measure scalar diffusion metrics of fractional anisotropy (FA), radial (RD), axial (AD), and mean (MD) diffusivities. Three ROIs (3.2x3.2x3.2 mm^3^) were placed along the body of the fornix (designated anterior, middle, and posterior) to mimic potential FUS sonication target position and size. Comparisons were performed across subjects for each individual ROI placement as well as in a group, effectively covering the complete segment of the fornix body.

**Results**

The tracts of the fornix were successfully imaged in both settings, and included the anterior, middle, and posterior sub-regions of the fornix body. Measurement of ROI diffusivities demonstrated that, across all comparisons, the post-mortem subjects showed significantly decreased mean, axial, and radial diffusivity (p<0.0005). FA, however, was unaltered between both groups (p value), suggesting that the overall balance of diffusivities and directionality remains unchanged.

**Conclusions**

We observed a consistent and significant pattern in the diffusivities between in vivo and post mortem piglets. *Post mortem* subjects exhibited decreased MD, AD, and RD in the fornix compared to living subjects but showed no significant change in FA. The *post-mortem* decrease in diffusivity is indicative of a number of factors including lack of blood circulation, lack of cerebral spinal fluid, and decreased tissue temperature. Unaltered measures of FA exhibits that diffusion directionality remains consistent and that similar tractography algorithms and parameters may be readily applied to both groups. This shows that *post mortem* animal models may be sufficient for methodological development in DTI and FUS research. DTI is also shown to be an effective tool for visualizing white matter structures and extracting diffusion metrics which are useful measures for the assessment of focused ultrasound treatment.

**Fig. 163 (abstract P19).**
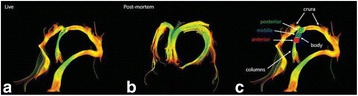
Reconstructed fornix models in **a** live and **b** post-mortem porcine subjects. **c** Fornix sub-regions of the columns, body, and crura are identified. Three regions of interest are indicated in the anterior (*red*), middle (*blue*), and posterior (*green*) body of the fornix

### P20 Protective effect of ultrasound on brain damage in rats with chronic cerebral hypoperfusion

#### Feng-Yi Yang, Sin-Luo Huang

##### Department of Biomedical Imaging and Radiological Sciences, National Yang-Ming University, Taipei, Taiwan

**Objectives**

The decreased cerebral blood flow is a prominent risk factor for cognitive dysfunction. Numerous clinical studies have indicated that the reduction of cerebral blood flow is often observed in patients with vascular dementia (VaD). Therapeutic drugs against VaD have faced many challenges, as it has no effective treatment with existing drugs. Thus, the development of proper strategies for treatment of VaD would be highly desirable. The aim of this study was to investigate the effects of ultrasound on VaD rats.

**Methods**

Low-intensity pulsed ultrasound (LIPUS) was generated by a 1-MHz focused piezoelectric transducer at a 5% duty cycle and a repetition frequency of 1 Hz. The focused transducer was positioned using the stereotaxic apparatus in order to direct the acoustic beam to the desired region (3.0 mm posterior and 2.5 mm lateral to the bregma) of the brain. A function generator was connected to a power amplifier to create the US excitation signal. The spatial-peak temporal-average intensity over the focused transducer head was 528 mW/cm^2^, and was measured in degassed water. LIPUS was transmitted from the top of the rat brain. Each rat hemisphere was treated by LIPUS with triple sonications. The duration of each sonicaton was 5 min and there was an interval of 5 min between each sonication.

All procedures involving animals were in accordance with the guidelines for the Care and Use of Laboratory Animals. Male Sprague–Dawley rats weighing from 160 to 170 g were used in this study Before bilateral common carotid artery occlusion(BCCAO) operation, each animal was anesthetized in the prone position by inhalation of 2% isoflurane in 2 l/min oxygen, and the body temperature was maintained at 37 °C using a heating pad. Two-vessel occlusion was produced in rats with permanent BCCAO. The bilateral common carotid arteries were ligated with 5–0 type surgical silk suture in BCCAO rats. The rat heads were mounted on a stereotaxic apparatus, and the top of the cranium was shaved for LIPUS stimulation. The animals were randomized into four groups (Sham, LIPUS, BCCAO, and BCCAO+LIPUS) for biochemical analysis and histological observation. The normal rats served as sham group. Animals in group of LIPUS were treated with LIPUS daily for 14 days. In BCCAO group, animals received BCCAO surgery. Two weeks after BCCAO, the BCCAO rats treated with LIPUS daily for 14 days were assessed in group of BCCAO+LIPUS.

**Results**

**Fig. 164 (abstract P20).**
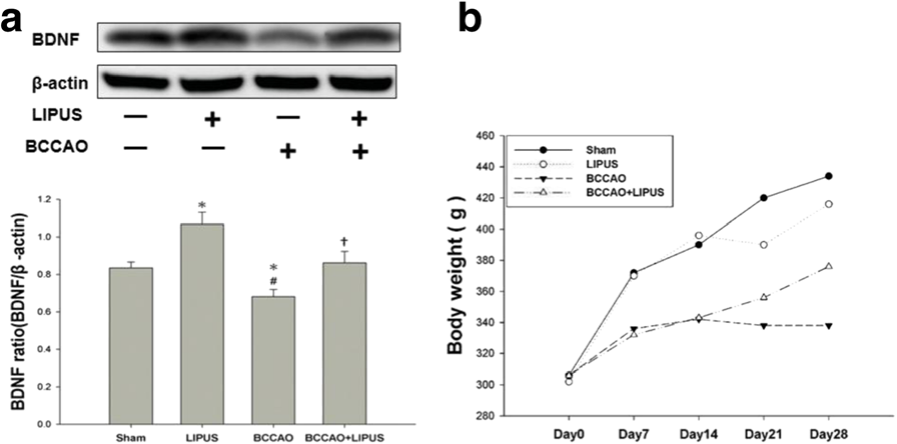
**a** Graph shows the protein expressions of BDNF in the hippocampus. **b** Graph shows the change of body weight in the LIPUS-treated BCCAO rats. *, #, and † denote significant differences compared to Sham, LIPUS, and BCCAO groups rats, respectively. (*,#,†, p<0.05)

To further confirm the effect of LIPUS on the protein levels of BDNF in the brain, bilateral rat hemispheres were exposed to multiple LIPUS stimulations. Western blot analysis was used to examine the endogenous protein expressions 24 h after the last LIPUS stimulation. The protein expressions of BDNF in the stimulated normal brains were significantly enhanced compared with the sham rats (Fig. 164a). There was a significant decrease of BDNF level in BCCAO rats as compared to the sham rats. However, no significant differences were found for the protein expressions of BDNF in the sonicated BCCAO rats as compared with sham rats. Figure 164b shows the change of body weight in BCCAO rats after LIPUS treatment. The body weight of BCCAO rats reached a plateau at day 7 after surgery. The body weight of LIPUS-treated BCCAO rats was significantly increased at two week after LIPUS treatment. Besides, no significant differences were found in the LIPUS-treated rats as compared to the sham group.

**Fig. 165 (abstract P20).**
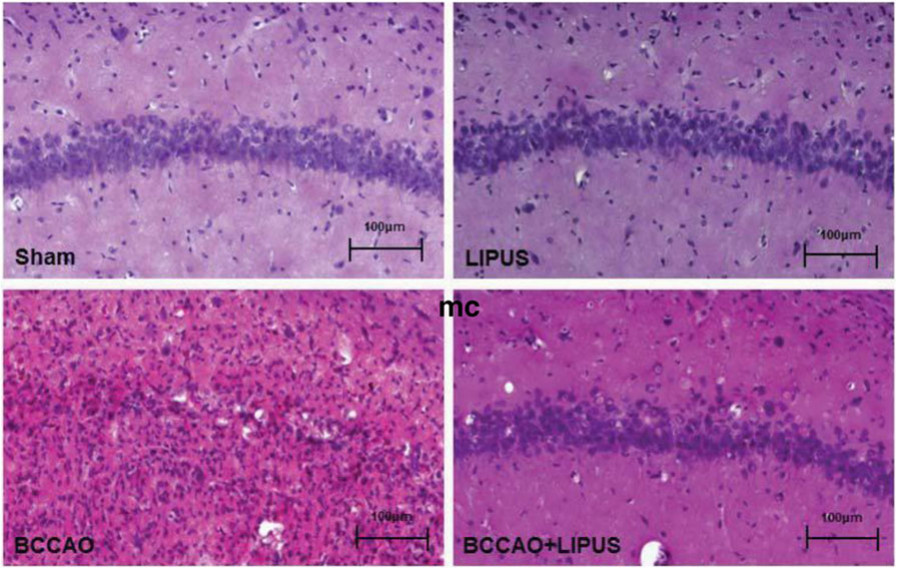
H&E staining shows the effects of LIPUS on brain damage in the hippocampus. The scale bar is 100 mm in amplified regions

Figure 165 shows H&E staining of neurons in the hippocampus of the four groups. The surviving cells of the hippocampus band in the dorsal hippocampus were observed. The hippocampal neurons in sham rats were arranged neatly, cell morphology was intact, and cell outlines were distinct. No significant difference was found in the cell morphology of neurons in the hippocampal region between sham rats and LIPUS rats. By contrast, hippocampal cells of BCCAO rats were indistinct and loosely arranged.

**Conclusions**

LIPUS plays the beneficial effects in the restoration treatment of VaD, which may be related to the mechanism that LIPUS can promote the protein levels of BDNF and affect the synaptic plasticity in the hippocampus of brain. However, further investigations are needed for the elucidation of the detailed mechanisms.

### P21 MR AFRI based on fast spin echo pulse sequence

#### Yuval Zur^1^, Alexander Volovick^2^, Benny Assif^2^

##### ^1^General Electric, Tirat Carmel, Israel; ^2^InSighTec, Tirat Carmel, Israel

**Objectives**

Magnetic Resonance Acoustic Radiation Force Imaging (MR ARFI) is a promising imaging modality that combines MR Imaging and Focused Ultrasound. The modality allows localizing the acoustic spot applying very low energy. This ability is especially beneficial for transcranial MRgFUS there intensity based measurements can be translated to phase delay for skull aberration correction (Hertzberg et al., Marsc et al.). Recently several new methods for focusing improvement were suggested that reduce the amount of needed samples – keyhole acceleration (Paquin et al.) and the use of full MR ARFI image instead of a single pixel for the auto-focusing (Grissom et al.). The new methods underline the need for MR ARFI that is not prone to geometrical distortions as the echo planar imaging from one hand but will allow same low amount of applied acoustic energy for single image generation.

The suggested method allows generating a full MR ARFI image using 4 ultrasonic pulses and significantly reduces the geometrical distortions of the image.

**Methods**

**Fig. 166 (abstract P21).**
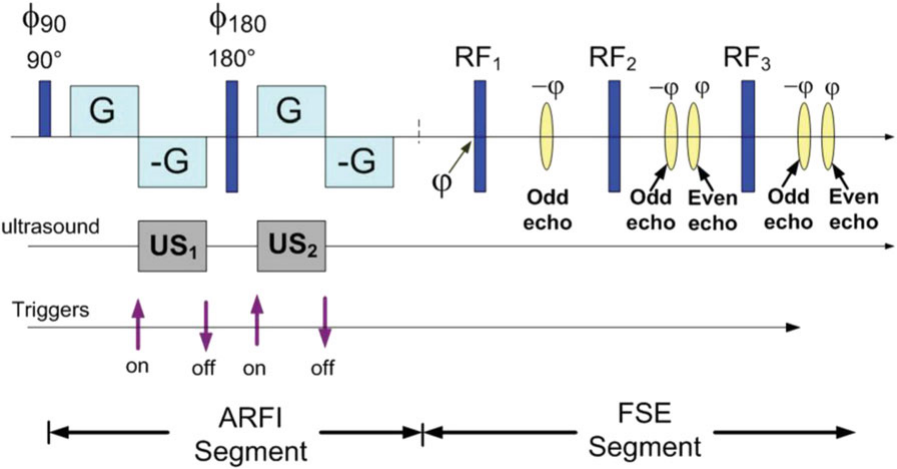
Fast Spin Echo based MR ARFI pulse sequence diagram

Fast Spin Echo Pulse sequence that is depicted in Fig. 166 was used on MR 1.5T (GE). ExAblate Neuro (InSighTec) system was the source of the ultrasonic pulses. Ultrasound compatible 2 channel head coil (InSighTec) was used for the imaging. In-house built gel phantom was used for displacement generation.

**Results**

**Fig. 167 (abstract P21).**
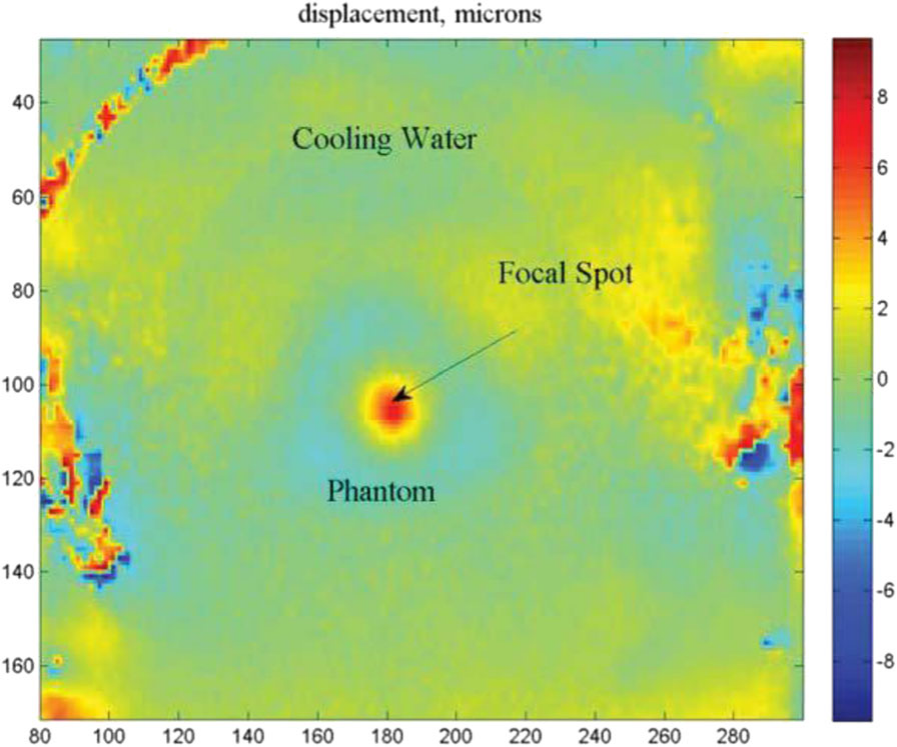
See text for description

MR-ARFI displacement image in perpendicular to the ultrasonic beam direction is presented on Fig. 167. The Fast Spin Echo scan is not affected by B0 inhomogeneity, producing symmetric displacement map in parallel to the beam direction, as can be seen on Fig. 167.

**Conclusions**

The new fast spin echo based sequence significantly reduced geometrical distortions, while improving the signal to noise ratio. The energy needed for single MR ARFI image generation is currently 2 times higher than for the EPI based sequence, however it can be further reduced using reduced field of view and parallel imaging techniques. The TE time is not ARFI pulse time depended, which allows higher image quality for different tissues.

### P22 Motor response elicitation and pupil dilation using Megahertz-range focused ultrasound neuromodulation

#### Christian Aurup^1^, Hermes Kamimura^2,1^, Shutao Wang^1^, Hong Chen^1^, Camilo Acosta^1^, Antonio A. Carneiro^2^, Elisa E. Konofagou^1^

##### ^1^Columbia University, New York, NY, USA; ^2^Universidade de São Paulo, São Paulo, Brasil

**Objectives**

Using transcranial focused ultrasound for the modulation of brain activity has been identified as a possible non-invasive means of treating neurological disorders. Most studies involving sedate rodents use frequencies in the kilohertz range, which allow for optimal transmission of acoustic power through the skull. The trade-off of using lower frequencies involves a lack of target specificity. Higher frequencies must be used in order to modulate activity in a more highly-specified manner. This study demonstrates that focused ultrasound in the megahertz range can be used to evoke motor- and cognitive-related responses in mice under deep anaesthesia by targeting specific brain structures. Contralateral-paired hind limb movements were observed when stimulating cortical regions, demonstrating the ability of MHz-range FUS to stimulate activity in highly-localized brain regions. Additionally, pupil dilation was observed when deep-seated anxiety-related structures were targeted, demonstrating the ability of FUS to modulate cognitive activity in a highly-specified manner.

**Methods**

For this study, wild-type adult male mice were anesthetized with intraperitoneal injections of sodium pentobarbital (65 mg/kg) and fixed in a stereotaxic frame. A single-element FUS transducer with fundamental frequency of 1.94 MHz was fixed to a 3D positioning system for accurate navigation through the brain. A 6x6 mm grid centred +2 mm rostral of the lambda skull suture was sonicated in a random order using a centre frequency of 1.9 MHz, pulse repetition frequency of 1 kHz, 50% duty cycle, 1 second pulse duration, 1 second inter-pulse interval for a total of 10 pulse repetitions. The acoustic pressure applied was varied in order to evaluate thresholds for eliciting physiological responses like motor movement, eye movement, or pupil dilation. Motor movements were validated using video recordings and electromyography via needle electrodes implanted into the biceps femoris of both hind limbs. Videos were recorded using a high-resolution camera focused at the right eye and processed to measure eye movements or changes in pupil size.

**Results**

The minimum acoustic pressure required to elicit motor movements was 1.45 MPa when targeting the somatosensory cortex, calibrated using an excised mouse skull. Higher pressures increased the success rate from 20% (at the 1.45 MPa threshold) to 70% (1.79 MPa). Targeting eye-motor and anxiety related regions of the brain elicited eye movements and pupil dilations up to 20%. Sonicating the superior colliculus resulted in both eye movement and pupil dilation at a lower threshold pressure (1.20 MPa) than the hippocampus and locus coeruleus which required pressures greater than 1.80 MPa.

**Conclusions**

This study successfully demonstrated that MHz-range transcranial focused ultrasound can be used to elicit motor- and cognitive-related physiological responses with high specificity in mice in vivo. It was also shown that the success rate of stimulation increased with acoustic pressure for motor movements associated with cortical activity modulation but highly depends on the region of the brain targeted. These findings emphasize the complex and yet to be determined mechanism of action involved in ultrasonic neuromodulation.

### P23 Pre-clinical *in-vivo* MR ARFI evaluation

#### Alexander Volovick^1^, Javier Grinfeld^1^, David Castel^2^

##### ^1^InSighTec, Tirat Carmel, Israel; ^2^Sheba Medical Center, Ramat Gan, Israel

**Objectives**

Transcranial Magnetic Resonance guided Focused Ultrasound Surgery (MRgFUS) is a novel non-invasive therapeutic alternative that is gaining interest both with physicians and patients. One of the remaining challenges for transcranial MRgFUS is skull aberration correction. Because of skull variance between patients the amount of acoustic power reaching the focal point and the focus quality are hard to establish. MR Acoustic Radiation Force Imaging (ARFI) is the potential imaging modality that will allow such measurements. MR ARFI measures the displacement of tissue, which is proportional to the acoustic intensity. Based on the displacement we will be able to determine the amount of acoustic power reaching the focal point (skull attenuation) and the focus quality (skull aberration). In order to determine the calibration slope for the power and displacement area, in-vivo experiments on brainy tissue for large animal model were performed.

**Methods**

Nine subjects underwent wide craniotomy procedure. MR ARFI was performed for each animal at 3 different locations using different acoustic powers. MR ARFI sequence was Echo Planar Imaging (EPI) based and the experiments were performed using ExAblate Neuro mid frequency system (InSighTec), 1.5T MRI (GE). The imaging was performed using integrated 2 channels Head Coil, by InSighTec.

**Results**

**Fig. 168 (abstract P23).**
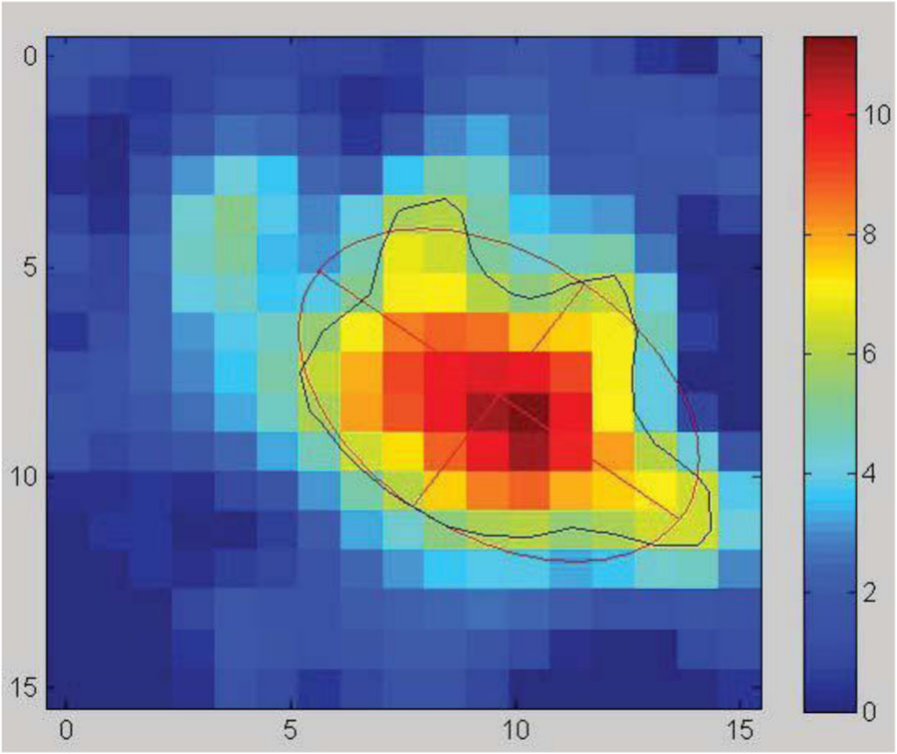
See text for description

**Fig. 169 (abstract P23).**
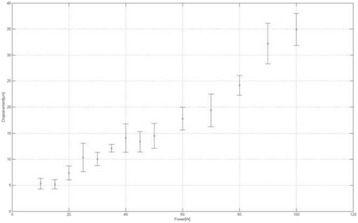
See text for description

Linear dependence of displacement at applied acoustic power is presented on Fig. 168. Typical displacement map is presented on Fig. 169. The shape of the spot is not symmetric because of EPI sensitivity to B0 field inhomogeneity. Spot diameter was observed to be 8.69±1.36 mm, almost 3 times higher than the acoustic intensity spot diameter.

**Conclusions**

As expected the displacement is linearly proportional to the applied acoustic power (intensity). The displacement diameter is higher than the acoustic intensity spot diameter because of the elastic properties of the brainy tissue. Spot diameter can be used as the focus quality measure. Taking into account expected skull attenuation and aberrations in patients; we expect the acoustic powers reaching the focus to be lower than 35W (which is 8 W/mm^2^ or 2.3MPa). Such knowledge can be a measure of safety for determining the proximity to cavitation threshold in the focus.

### P25 US and MR based motion tracking for HIFU and FUS

#### Sven Rothlübbers^1,2^, Julia Schwaab^1^, Christine Tanner^3^, Senay Mihcin^4^, Graeme Houston^4^, Matthias Günther^2,1^, Jürgen W. Jenne^2,1^

##### ^1^mediri GmbH, Heidelberg, Germany; ^2^Fraunhofer MEVIS, Bremen, Germany; ^3^ETH Zürich, Zürich, Switzerland; ^4^IMSAT, Dundee, UK

**Objectives**

One of the major challenges of liver or kidney tumour ablation with HIFU (High Intensity focused ultrasound) or FUS (Focused ultrasound surgery) is organ motion caused by respiration. A prerequisite for gapless and confluent tumour destruction is real-time knowledge of the target position. Diagnostic Ultrasound (US) and Magnetic Resonance Imaging (MRI) are suitable image guiding methods to provide nearly real-time position information. For several years, we have been developing and improving US-based motion tracking for MRgFUS (MR-guided FUS). The aim of the present study was to extend this approach to an MR-based motion tracking. Further goals were the automated instantiation of tracking features and the assessment of feature quality of a current tracking routine on US and MR, respectively.

**Methods**

For motion tracking, image streams are analyzed in real-time to track predefined structures (“features”). Basis for tracking are 2D/3D US or fast MR-EPI (Echo Planar Imaging) images of moving structures. In the first step, pronounced contours (e.g. diaphragm) or landmarks (e.g. vessels) are defined manually or automatically. A Particle Filter-based algorithm evaluates state hypotheses of local affine transformations to track these single positions through the image stream. With each position, an associated quality measure based on the probability distribution is provided. Tracking data is then sent to the treatment unit, e.g. MRgFUS/HIFU system. Based on this position data, real-time beam steering can be performed to compensate the motion. If the motion exceeds predefined limits, the therapy procedure can be interrupted immediately.

**Results**

The tracking algorithm was evaluated on liver US and MR data from volunteers. Automated detection of blood vessels as tracking feature on MR-EPI images yielded satisfying results. Computing time for tracking on MR-EPI image data was about 1ms per frame. A mean tracking error of 1.7 mm was achieved. Feature position estimation on US image streams was possible in less than 2 ms per frame with an averaged position error of 1.5 mm. Automated estimation of feature quality proved to be not always sufficient.

**Conclusions**

Real time liver motion tracking on 2D/3D US- or MR-data streams with sufficient spatial and temporal resolution for precise MRgFUS is possible. Further refinements for automated estimation of tracking feature quality are necessary. In the future, an automated replacement of insufficient tracking features will be implemented.

*Acknowledgements*

*Parts of the research leading to these results has received funding from the European Union's Seventh Framework Programme (FP7/2007-2013) under grant agreement no. 611889 (TRANS-FUSIMO).*

### P26 T2-mapping as a predictor for non-perfused volume in mrgfus treatments of desmoid tumours

#### Eugene Ozhinsky, Matthew D. Bucknor, Viola Rieke

##### Radiology and Biomedical Imaging, University of California San Francisco, San Francisco, CA, USA

**Objectives**

MR-guided focused ultrasound (MRgFUS) is a non-invasive ablation technique that has been successfully used for the treatment of uterine fibroids and bone metastases. More recently, focused ultrasound has been investigated for novel indications such as treatment of essential tremor, focal breast lesions, osteoid osteomas, and desmoid tumours.

Desmoid tumours are benign but locally aggressive soft tissue tumours that arise from fibroblast cells. Conventional therapies include surgical resection, radiation and chemotherapy. Despite these treatments, the tumours have a high recurrence rate of up to 50% in 5 years (Peng PD, et al. Ann Surg Oncol, 2012). As an alternative treatment, focused ultrasound has shown promising results in reduction of tumour volume without significant side effects (Wang Y, et al. Int J Hyperthermia, 2011). Visualization of the ablated volume remains a large problem in focused ultrasound therapy. Thermal dose maps are used during the treatment to visualize the treated volume, but fail to reliably predict the extent of ablation. Post-treatment contrast enhanced MR imaging allows assessment of the non-perfused volume (NPV), the gold standard assessment of the quantity of tumour ablation. However, safety concerns regarding heating of tissue after gadolinium injection prevent further treatment following the NPV assessment.

In this study we investigated rapid T2 mapping as a way to visualize tissue changes during MRgFUS treatment of patients with desmoid tumours.

**Methods**

MR-guided focused ultrasound ablation was performed in two patients with paediatric desmoid tumours in lower extremities using an ExAblate 2100 system (InSighTec, Haifa, Israel) integrated with a 3.0 Tesla MR scanner (GE Healthcare, Waukesha, WI, USA). Sonications were performed with duration of 20–30 sec and acoustic power between 49 and 107 W.

The patients received 68 (patient 1) and 65 (patient 2) treatment sonications with 2–4 verifications sonications over the course of about 3 hours.

Over the course of the treatment 4 (pt. 1) and 6 (pt. 2) double echo Fast Spin Echo images were acquired (TE = 35/186 ms, TR = 1500 ms, echo train length = 40, FOV = 24–28 cm, 128 x 128 matrix size, BW = 15.6 kHz, 10 mm slice thickness, 1–2 slices, 15 s acquisition time) before, during and after the treatment. T2 maps were generated with an exponential fit for two data points. At the end of the treatment pre- and post-contrast 3D FSPGR images were acquired. T2-maps were compared to post-contrast images.

**Results**

**Fig. 170 (abstract P26).**
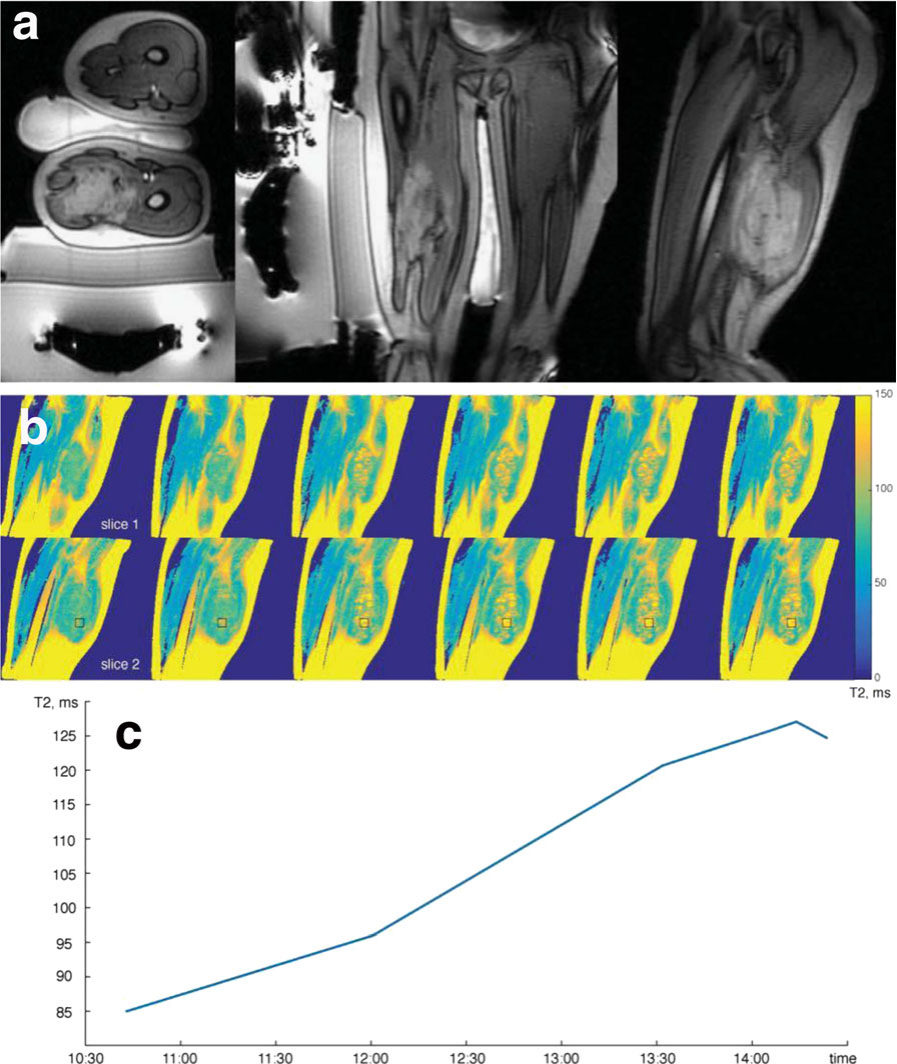
**a** Axial, sagittal and coronal localizer images for patient 2 showing the transducer, gel pad and cooling water bag; **b** coronal T2 maps of two slices acquired during the treatment over time (ROI shown in black on slice 2); **c** plot of T2 values within the ROI over time

**Fig. 171 (abstract P26).**
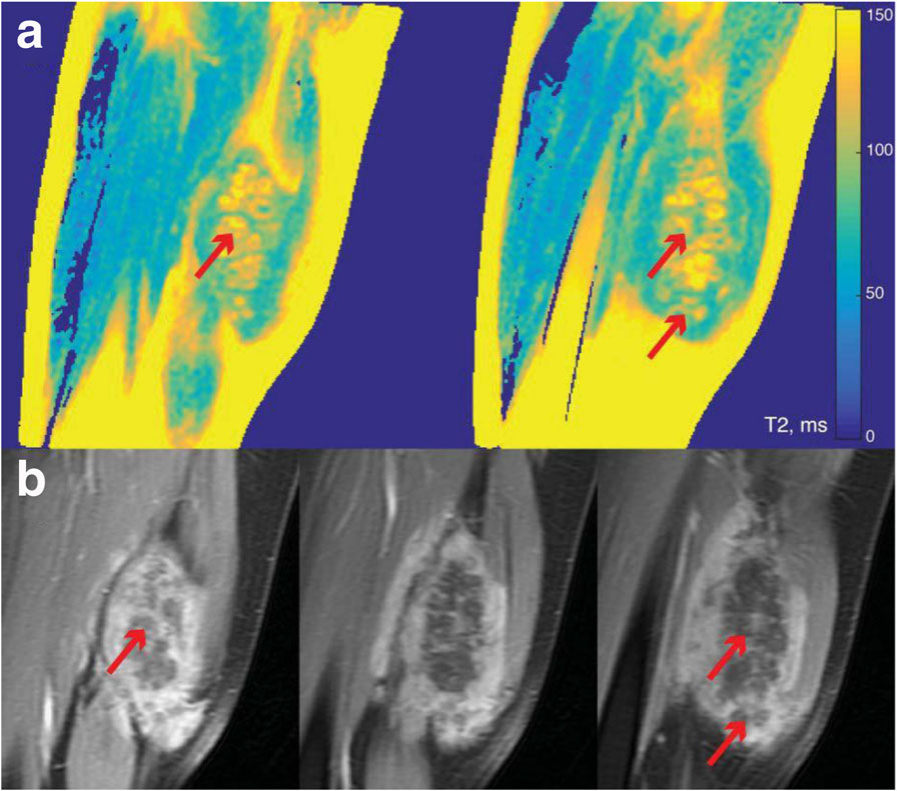
Comparison between T2 maps (**a**) and post-contrast images (**b**) for the same location for patient 2. The non-perfused volume (NPV) appears dark on the post-contrast images. Arrows show gaps in the NPV, which correspond to areas with low T2 in the T2 maps

Our results in Figs. 170 and 171 show that T2 mapping could be used to visualize the changes in tissue during focused ultrasound treatments of desmoid tumours. The areas of T2 elevation showed an excellent agreement with the non-perfused volumes in the post-contrast images. T2 values of tissues such as muscle and fat have been shown to increase with temperature (Graham SJ, et al. Magn Reson Med, 1998; Ozhinsky E, et al. J Ther Ultrasound, 2015). Although some of the observed T2 elevation could be caused by the heat from the previous sonications, the elevated T2 values were observed throughout the interior of the tumour. Future work will study the T2 values in the tumour after it returned to the baseline temperature.

**Conclusions**

We have shown that T2 mapping can be used to visualize the extent of ablation with focused ultrasound and be used as a predictor of NPV without the need for contrast injections. This could be used by physicians to ensure complete ablation of the tissue within the region of treatment.

### P27 HIFU thermal ablation monitoring using x-ray CT - potential and challenges

#### Haim Azhari^1^, Noam Weiss^1^, Jacob Sosna^2^, S. Nahum Goldberg^2,3^

##### ^1^Biomedical Engineering, Technion, IIT, Haifa, Israel; ^2^Department of Radiology, Hadassah, Hebrew University, Medical Center, Jerusalem, Israel; ^3^Radiology, Beth Israel Deaconess Medical Center, Boston, MA, USA

**Objectives**

X-ray CT offers a commonly available imaging modality, with high spatial and temporal resolution. CT is substantially less expensive than MRI and provides images which quality is superior to ultrasound. In addition, CT does not impose any restrictions on the equipment located in its vicinity as MRI does and unlike ultrasound can scan the in-vivo brain. The objective of this study was to examine the feasibility of using CT for non-invasive monitoring of high intensity focused ultrasound (HIFU) thermal ablation.

**Methods**

Ex-vivo tissue samples which included porcine fat and bovine liver were heated by HIFU while positioned within a CT scanner (Philips Healthcare). A thermocouple (Omega Engineering) which was inserted into the HIFU focal spot was used to register the temperature as a function of time during the HIFU heating and cooling processes. After the specimen was sufficiently cold, the thermocouple probe was removed to avoid image artefacts. Then, while using the same experimental setup and the same tissue, the HIFU was aimed at an untreated spot located several centimetres away from the previously treated area. The HIFU was activated again, this time while acquiring CT images at very short intervals during both heating and cooling stages. The set of scanned slices were positioned at and around the focal zone. The images were registered, time stamped and transferred to an external computer for further analysis. From the obtained data, the CT Hounsfield Units (HU) vs. temperature curves were plotted.

**Results**

The results have shown that the HU change in a nonlinear manner during the heating stage. Contrary to that, a linear relation of the HU-Temperature curve was observed during the cooling stage. This formed a hysteresis phenomenon [1] which may be attributed to irreversible changes in the tissue resulting from the thermal ablation. Furthermore, while the HU- Temperature loop obtained during heating and cooling for liver tissue progressed along the clockwise direction, the HU-Temperature loop progressed along the counter clockwise direction for fat [2]. Moreover, for fat, a clear nadir point was observed at about 44.5°C [2].

**Conclusions**

X-ray CT HU are sensitive to thermal changes, indicating their potential to serve as a tool for thermal monitoring. However, the HU-Temperature relation is nonlinear and its characteristics are different for different tissue types. This may pose a challenge when treating an organ which comprises of several tissue types. Another challenge stems from the need for a high rate of image acquisition. This may be currently associated with a high radiation dose. In conclusion, assuming that radiation dose can be reduced to an acceptable level, X-ray CT may be a valuable modality for non-invasive monitoring of HIFU thermal procedures.

**References**

[1] Weiss N, Goldberg SN, Sosna J, Azhari H. *Temperature - density hysteresis in X-ray CT during HIFU thermal Ablation: Heating and cooling phantom study*. International Journal of Hyperthermia. February 2014; 30 (1): pp. 27–35.

[2] Weiss N, Goldberg SN, Sosna J, Azhari H. *Non-invasive temperature monitoring and hyperthermic injury onset detection using X-ray CT during HIFU thermal treatment in ex-vivo fatty tissue*. International Journal of Hyperthermia. 2014; 30 (2): pp. 119–125.

### P28 Temperature measurement using ultrasonic power for non-invasive thermometry during HIFU ablation

#### Victor Barrere^1,2^, David Melodelima^1,2^

##### ^1^Inserm, Lyon, France; ^2^Université de Lyon, Lyon, France

**Objectives**

High intensity focused ultrasound (HIFU) causes selective tissue necrosis in a very well defined volume. Tissue necrosis is created at a variable distance from the transducer through heating and cavitation. Over the past decade, the use of HIFU has been investigated in many clinical settings with the most prominent applications in prostate cancer and more recently for the treatment of liver tumours, uterine fibroids and brain disorders, amongst others. Today, the only reliable technique allowing measuring the temperature during a HIFU treatment is Magnetic Resonance Imaging (MRI). The spatial resolution of MR images during thermometry is about a millimetre and the temporal resolution is about half a second. Recent work has also demonstrated that temperature monitoring using MRI is possible during breathing. Nevertheless, MRI requires a complex and expensive apparatus with limited availability. MR monitoring also implies a trade-off between frame rate versus spatial resolution and monitoring accuracy. Moreover, HIFU boiling can happen rapidly causing errors and artefacts. Finally it requires MR-compatible electronics and transducers. Otherwise, ultrasound imaging allows fast, cheap and portable devices. Temporal and spatial resolutions of ultrasound images are high but the contrast is low and all organs cannot be imaged (for example, brain and lungs). Today reliable temperature monitoring, specifically for temperature higher than 55°C, is not possible using ultrasound imaging. Many studies have suggested using the estimation of the speed of sound, attenuation coefficient or recently variations in the shear modulus to estimate temperature. The main limit of ultrasound based methods is that the range of measurable temperature is often limited by cell lysis threshold, which is around 50°C. However, many other acoustic parameters of tissues can be measured such as the backscattered energy or the backscatter coefficient that can be combined to the attenuation or the speed of sound to enable temperature measurement at higher values. In this work the relationship between changes in ultrasound backscattered power and temperature during HIFU treatments was studied.

**Methods**

A flat pulse-echo transducer working at 2.25 MHz (bandwidth: 1.4 – 2.9 MHz) was used. The active diameter of the pulse-echo transducers was 10 mm. A high intensity ultrasound transducer working at 3 MHz was used to create lesions in in vitro liver samples. The active diameter of this transducer was 4 cm. Experiments were conducted in a tank filled with degassed water at 37°C. The acoustic axis of the pulse-echo transducer was placed perpendicularly to the acoustic axis of the HIFU probe. An acoustic mirror was placed parallel to the surface of the pulse-echo transducer. The electrical power was delivered to the high intensity transducer in continuous mode via a power amplifier driven by a sinusoidal wave generator. A power meter was inserted between the amplifier output and the high intensity ultrasound transducer. The pulse-echo transducer was driven by a pulse-echo generator connected to an oscilloscope to acquire signals.

First a pulse was emitted by the pulse-echo transducer only in water. This pulse wave was reflected on the acoustical mirror and used as a reference for the total energy emitted by the transducer. Then a liver sample was placed in a holder between the pulse-echo transducer and the acoustic mirror. Liver tissues were warmed up to 37°C and then high intensity exposures were created to increase the temperature in liver samples. Temperature was recorded using a thin thermocouple placed on the heated zone in the liver. Long exposure time (30 minutes) was used to observe smooth temperature increase from 37 to 70°C. Pulse-echo acquisitions were performed every minute during heating. Four experiments were conducted to observe the reproducibility of the measurements. Acoustical signals were recorded using an oscilloscope and analysed using software specifically developed on MATLAB. From this signal, the attenuation of the liver, the energy of the backscattered signal, the backscatter coefficient, and the speed of sound were computed.

**Results**

The attenuation coefficient increases only when a coagulation necrosis is created in liver tissues. Its initial value for an untreated liver is about 0.08 Np.cm-1.MHz-1, and increases suddenly to 0.35 Np.cm-1.MHz-1 when necrosis is created (around 60°C). The speed of sound increases from an initial value of about 1478 m.s-1 to 1505 m.s-1 at 50°C and then decreases to its initial value. During heating, the energy of the backscattered signal increases roughly linearly with temperature. A linear increase of 3 dB was measured in ultrasound backscattered power during experiments. The tissue temperature increase estimated using backscattered energy correlated well (r=0.79) with temperature measurements performed using thermocouples. This linear relationship between changes in the backscattered energy and actual temperature was observed up to 70°C.

**Conclusions**

Other parameters such as acoustical impedance of the tissue, size and density of the scatterers, could also be correlated to these measurements in order to increase the accuracy of temperature estimation. Successful temperature estimation may allow creating 2D temperature maps during HIFU treatments using ultrasound imaging.

### P29 Investigation of cardiac responses to MR-guided pulsed focused ultrasound in a rat model

#### Kee W. Jang, Scott R. Burks, Zsofia I. Kovacs, Tsang-Wei Tu, Bobbi Lewis, Saejeong Kim, Matthew Nagle, Neekita Jikaria, Joseph A. Frank

##### Frank Laboratory, Radiology and Imaging Sciences, National Institutes of Health, Bethesda, MD, USA

**Objectives**

The use of pulsed focused ultrasound (pFUS) has been demonstrated as a non-invasive regenerative medicine to enhance homing permeability and retention (EHPR) of intravenously (i.v.) infused mesenchymal stem cells (MSCs) to target tissues [1–3]. The purpose of this study was to examine the effects of a non-invasive MR-guided pFUS on cardiac responses in a rat model.

**Methods**

**Fig. 172 (abstract P29).**
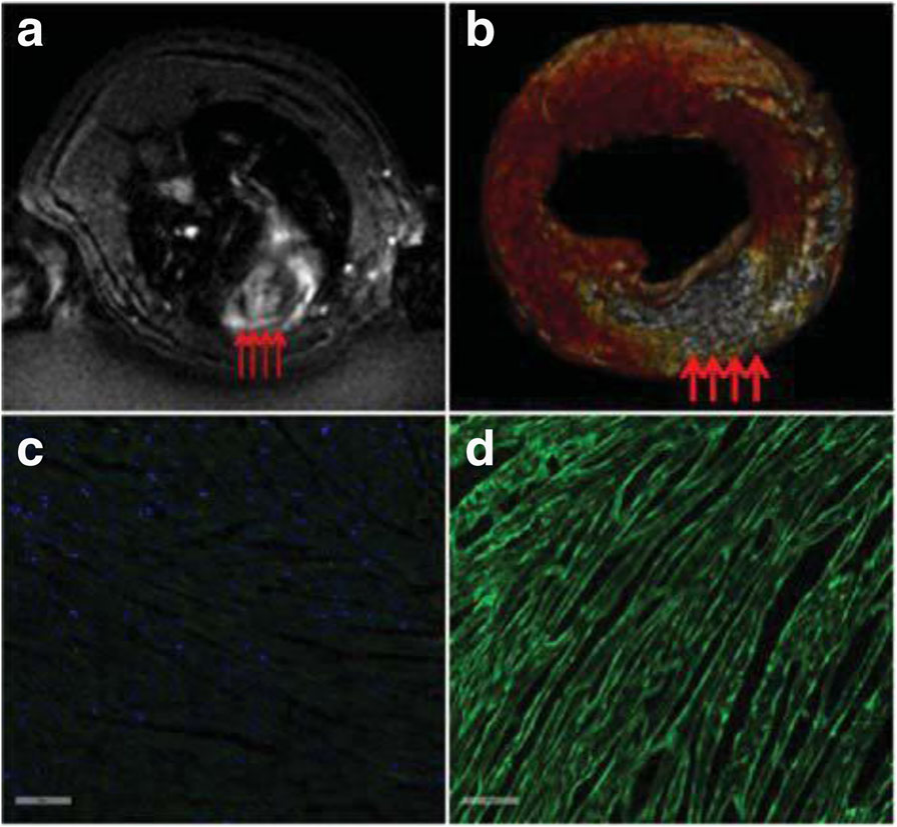
Myocardial oedema found in pFUS treated region. **a** An axial MR image that used as guidance for pFUS to target the rat cardiac tissue. **b** pFUS exposure resulted in myocardial oedema that found in 7T MR images. IHC analysis confirmed that significantly increased albumin was present in pFUS treated region (**d**) while little was found in SHAM (**c**). *Red arrows* represent the pFUS targeting regions. Bar = 100 μm

Eight to ten week old female Sprague Dawley rats were imaged on a 3T clinical MR scanner (Achieva, Philips Healthcare, USA) and T2-weighted MR images were acquired with 8.9ms repetition time (TR), 4.5ms echo time (TE) in 1mm slice thickness (Fig. 172a).

The images were used as guidance for pFUS to target the rat cardiac tissue (1 MHz; 0 to 8 MPa; 10 ms bursts; 0.7 Hz PRF; RK-100, FUS Instruments, CAN). The levels of cardiac troponin I (cTnI), a cardiac injury marker (Rat cTnI ELISA kit, LSBio, USA), and brain natriuretic peptide (BNP), a cardiac muscle stretch marker (BNP Enzyme Immunoassay kit, RayBio, USA), in blood plasma were evaluated after pFUS exposure at 0 to 8MPa with 100 pulses per 9 targets. In order to determine the accurate location of pFUS targeted region in hearts, the hearts were harvested after euthanasia to examine the immuno-pathologic changes using 7T MR microimaging system (Bruker, USA) with 7000 ms TR and 50 ms TE in 250 μm slice thickness.

**Results**

**Fig. 173 (abstract P29).**
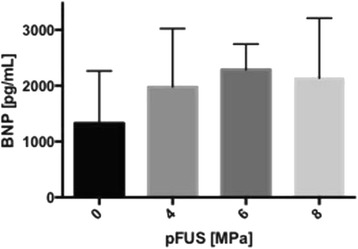
pFUS exposure to the hearts resulted in slightly increased BNP level in blood plasma. No correlation of pFUS pressure-dependent BNP levels was found between groups

pFUS exposure resulted in histological changes in targeted region of hearts. Myocardial oedema in pFUS targeted region was also found in 7T MR images (Fig. 172b) while there was no difference with the SHAM group. Immunohistochemical (IHC) analysis confirmed that serum albumin was present in pFUS treated regions (Fig. 172d) while little was found in SHAM group (Fig. 172c). No significant changes of cTnI levels were found in pFUS groups compared to SHAM; however, mean BNP levels were slightly increased by pFUS (Fig. 172). Qualitatively there was no tissue damage in pFUS exposed groups by haematoxylin and eosin stain (data not shown). IHC analysis shows that intercellular adhesion molecules (ICAM) were considerably enhanced by pFUS (Fig. 173a & c) compared to SHAM (Fig. 173b).

**Conclusions**

It has been previously demonstrated that pFUS exposure enhanced i.v. infused MSCs homing toward pFUS-targeted tissue. Combination of pFUS exposure and MSCs infusion has already proven to be effective in rescuing mice from cisplatin-induced acute kidney injury (AKI) by modulating anti-inflammatory immune cell profile [1–3]. We investigated the mechanical and histological effects of pFUS exposure on rat cardiac tissue in this study. Our data demonstrate that pFUS treatment can be non-invasively applied to cardiac tissue as mechanical stimuli without damages in order for triggering biologic responses. In conclusion, pFUS has potentials to be used as a non-invasive regenerative medicine for cardiac diseases in combination of MSCs by modulating anti-inflammatory responses.

**References**

[1] Burks et al., *Investigation of Cellular and Molecular Responses to Pulsed Focused Ultrasound in a Mouse Model*. PLoS One. 2011;6(9):e24730.

[2] Burks et al., *Noninvasive pulsed focused ultrasound allows spatiotemporal control of targeted homing for multiple stem cell types in murine skeletal muscle and the magnitude of cell homing can be increased through repeated applications*. Stem Cells. 2013 Nov;31(11): pp. 2551–60.

[3] Burks et al., *Pulsed focused ultrasound pretreatment improves mesenchymal stromal cell efficacy in preventing and rescuing established acute kidney injury in mice*. Stem Cells. 2015 Apr;33(4): pp. 1241–53.

**Fig. 174 (abstract P29).**
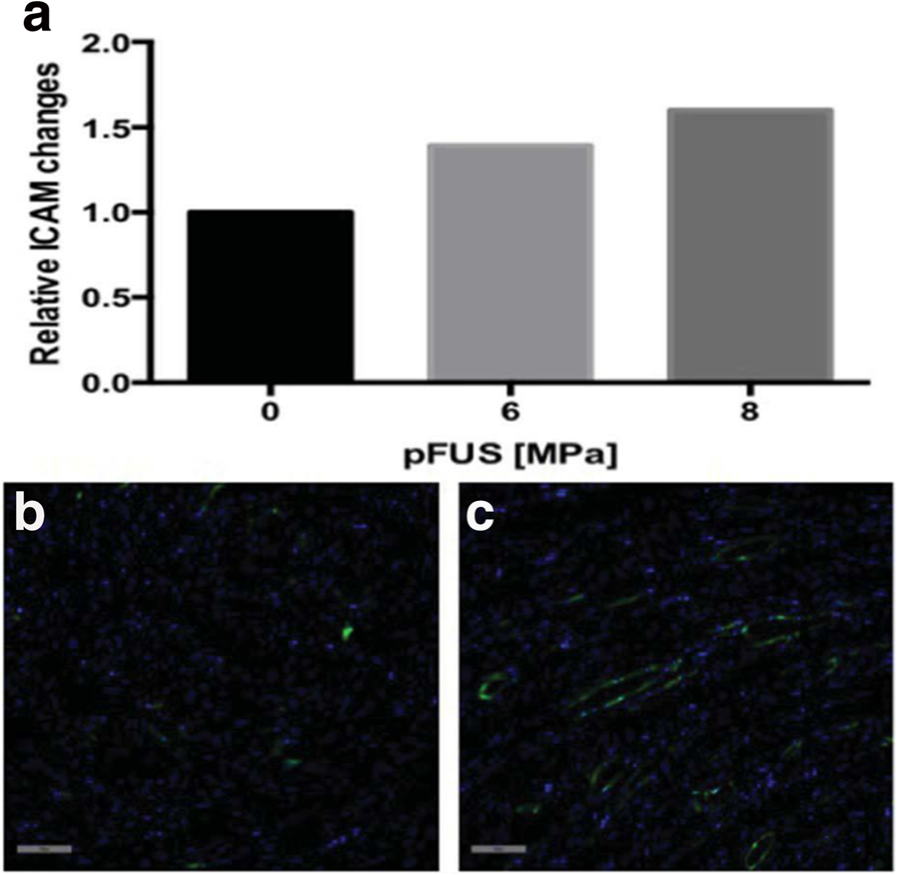
ICAM expression in pFUS exposed regions. Enhanced ICAM in pFUS treated regions were found by IHC analysis (**a**). Compared to SHAM (**b**), considerably higher ICAM stained regions were found in pFUS exposure groups (**c**). Bar = 100 μm

### P30 Material erosion by pulsed high-intensity focused ultrasound (_P_HIFU)

#### Yufeng Zhou, Xiaotong wang

##### Nanyang Technological University, Singapore, Singapore

**Objectives**

High-intensity focused ultrasound (HIFU) has been emerged as non-invasive and effective modality of cancer/tumour ablation utilizing its thermal effect. In the HIFU field, the high rarefactional pressure could also generate significant cavitation effect, which may lead to material erosion. The effects of pHIFU on the erosion of soft and hard material are studied to understand the underlying mechanisms.

**Methods**

Various ultrasound parameters were tried in the experiment. Cavitation signals were measured during pHIFU exposure by passive cavitation detection (PCD) approach. The volume and area of erosion at the interface of material and water were quantitatively assessed. High speed photography was carried out to capture the images of material erosion and bubble cavitation.

**Results**

The material erosion increases with the delivered acoustic power and pulse duration. At the same acoustic energy and duty cycle, low pulse repetition frequency (i.e., PRF=1 Hz) leads to more significant erosion (more than 10 folds) than that high PRF (i.e., 100~1000 Hz as histotripsy). It suggests that acoustic radiation force plays an important role in material erosion as well as bubble cavitation at the interface. Similar results were found in ex vivo experiments.

**Conclusions**

Ultrasound parameters could be optimized for the best material erosion using the same energy. Acoustic radiation force could push the ultrasound-induced bubble towards the interface for effective and strong cavitation, which results in more erosion. Utilizing pHIFU-associated mechanisms appropriately could improve the outcome of ultrasound.

### P31 Multiple treatments for pancreatic cancer using focused ultrasound in combination with gemcitabine: *in vivo* study in xenograft mouse model

#### Yun Deok Ahn, Eun-Joo Park, Dong-Hyuk Park, Soo Yeon Kang, Jae Young Lee

##### Radiology, Seoul National University Hospital, Seoul, Korea (the Republic of)

**Objectives**

In other study of our group, combination treatment of focused ultrasound (FUS) and gemcitabine (GEM) has shown that mechanical effects of FUS more effectively control the growth of pancreatic cancer in animal model. This study was designed to evaluate therapeutic effects of FUS+GEM treatment using the similar clinical protocol.

**Methods**

Human pancreatic cancer cells (CFPAC-1) were inoculated in BALB/c nude mouse as a pancreatic xenograft model. Animals were divided into two groups: GEM only, and FUS+GEM. For FUS+GEM group, animals were sonicated for 20 sec at 1 MHz in burst mode (acoustic power = 80.5 W, duty cycle = 5%). GEM was administered in IV immediately after sonication. One treatment cycle consisted of three weekly treatments and one week monitoring. The treatment cycle was repeated four times.

**Results**

For both treatment groups, tumour size reduced during the weekly treatments in each cycle and increased again during the monitoring period. Tumour in both groups showed similar growth pattern for each treatment cycle. However, the re-growth rate of tumour after the weekly treatments got higher as the treatment cycle repeated. Although pancreatic cancer treated by GEM combined with FUS showed reduction of the size, the efficacy of combination treatment became lower as the treatment cycle repeated.

**Conclusions**

In conclusion, chemotherapy combined with FUS shows feasibility of controlling pancreatic cancer growth. However, thorough studies for treatment efficacy and effective treatment protocol are required prior to clinical application.

### P32 Estimating acoustic radiation force induced tissue deformation during thermal ablation

#### Visa Suomi^1^, Elisa E. Konofagou^2^, David Edwards^1^, Robin Cleveland^1^

##### ^1^University of Oxford, Oxford, UK; ^2^Columbia University, New York City, NY, USA

**Objectives**

Acoustic radiation force (ARF) is dependent on tissue attenuation and sound speed, both of which are also known to change with temperature. Furthermore, the viscoelastic properties of tissue are also temperature dependent, which affect the displacements induced by ARF. The objective of this study is therefore to quantify the temperature dependent acoustic and viscoelastic changes in liver and measure their effect on ARF induced displacements both experimentally and with simulations.

**Methods**

The temperature dependent acoustic attenuation for liver was obtained from the literature and fit to a fourth order polynomial function. The temperature dependent viscoelastic properties of liver were experimentally measured and Zener-model of viscoelasticity was used to fit the data. The effect on ARF induced displacements was measured by using harmonic motion imaging (HMI) in ex vivo liver during high-intensity focused ultrasound (HIFU) thermal ablation. The measured displacements were then compared to finite element method (FEM) simulations with temperature effects incorporated.

**Results**

The measured peak-to-peak displacements initially decreased as tissue warmed, then reached a peak when the lesion was similar in size to the focal spot and in the end decreased as the lesion grew beyond the size of the focal spot. The characteristics experimental HMI data were captured well in the simulations. Based on the results a method for estimating lesion size based on this data was presented.

**Conclusions**

These results show that the displacement induced in ARF is dependent on both the acoustic and elastic properties of tissue and so any imaging modality exploiting these displacements needs to account for variations in both the acoustic and elastic properties of the tissue. The findings of this study benefit the research of ultrasound elasticity imaging modalities which are used in monitoring HIFU and other thermal ablation therapies.

**Fig. 175 (abstract P32).**
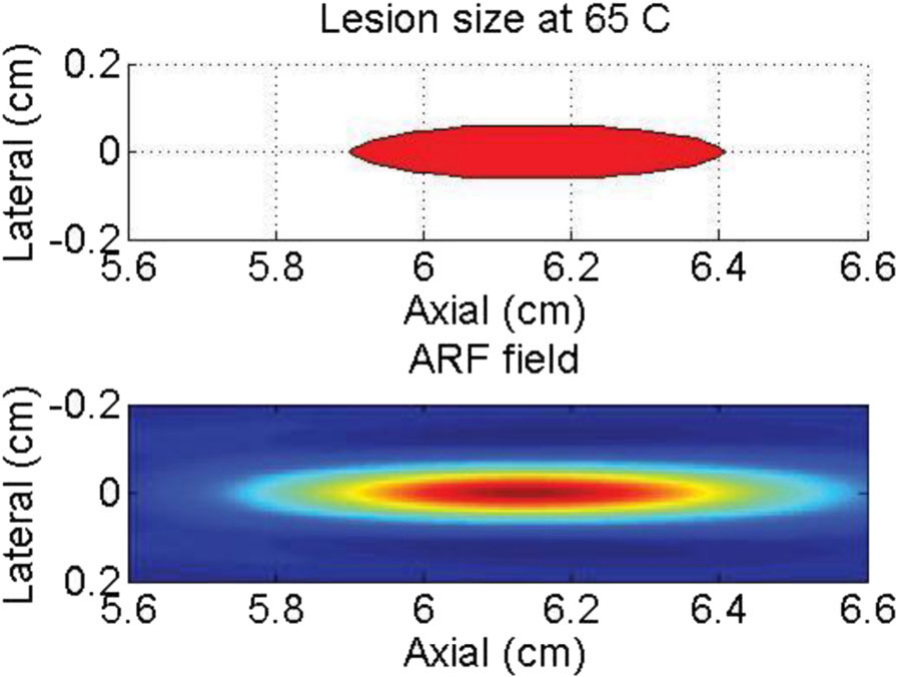
Lesion size versus ARF field during thermal ablation at 65°C

**Fig. 176 (abstract P32).**
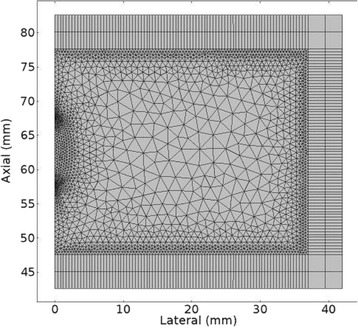
FEM mesh used in the modelling of ARF induced displacements

### P34 Low-cost thermochromic quality assurance phantom for focused ultrasound devices

#### Zahary Larrabee^1^, Matthew Eames^1, 2^, Arik Hananel^1^, Jean-Franҫois Aubry^3,4^

##### ^1^Focused Ultrasound Foundation, Charlottesville, VA, USA; ^2^Radiation Oncology, University of Virginia, Charlottesville, VA, USA; ^3^Institut Langevin, Paris, France, ^4^CNRS, Paris, France

**Objectives**

Errors in power output ranging from −100% to +210% have been reported in a multitude of physiotherapy transducers. Differences in power output can arise even after careful calibration on an annual or bi-annual schedule, which in the case of HIFU treatments can either result in harm to the patient or non-effective treatment. Many ultrasound phantoms require complex set-ups, with large water tanks and imaging systems to produce accurate data. Simpler phantoms have been developed for use in benchtop settings but still rely on complicated image analysis. We propose a simple, low cost and easy to use DQA phantom which allows the user to assess the power output of the focused ultrasound transducer, and determine if it has changed significantly after calibration. The proposed phantom will be a product with which the end-user can visually assess a transducer’s gross functionality without resorting to complicated image analysis.

**Methods**

The phantom is a highly attenuating ultrasound absorber with a surface layer of thermochromic liquid crystals (TLC) that change colour in response to heating. The acoustic absorber used was 3mm-thick AptFlex F28 (Precision Acoustics Ltd, Dorchester, Dorset, UK) and the TLC used was Hallcrest R35C5B (LCR Hallcrest, Glenview, IL, USA). Experimental results were generated by sonicating the absorbing surface of the phantom through a 35mm non-absorbing coupling gelatine layer using a 1.1 MHz single-element transducer with a fixed 23mm focus (Model XDR058, Sonic Concepts, Bothel, WA, USA). A custom-designed 3D printed jig was used to hold the transducer and phantom in known positions.

The phantom absorption and heating were modelled using a three-dimensional time-domain bioheat transfer simulation written in Matlab (Mathworks, Natick, MA, USA). Transducer output was measured with a needle hydrophone (Onda Corp., Sunnyvale, CA, USA) in a single plane parallel to the axis of acoustic propagation in order to provide an axisymmetric pressure field to the acoustic solver. The simulation was validated against experimental measurements of time to first colour change across four equivalent phantoms. The phantoms were allowed to cool for 8 minutes to return to a baseline resting temperature. Measurements were conducted with a 1.1 Mhz input with amplitude 57V and were repeated four times per phantom for each of three distinct sessions over the course of two weeks to test for consistency across distinct experimental sessions. Measurements were also collected input amplitudes of 74V and 142V for each phantom during a single experimental session to generate pressure dependent time-to-heating curves for the fabricated phantoms.

**Results**

Simulations were shown to be accurate within 15% of measured heating times. The phantoms demonstrated time to first colour change of 18s at the 57V amplitude excitation, 13s at the 74V amplitude excitation, and 8s at the 142V amplitude excitation, The individual phantoms had mean heating times of 17.4s (+/− 6%), 11.8s (+/− 5%), and 13.2s (+/− 7%) over all measurements and experimental sessions.

**Conclusions**

The phantom was shown to demonstrate the expected dependence on input voltage amplitude, with time-to-first-colour-change decreasing with increasing voltage. While there was noticeable phantom-to-phantom variability, each of the three phantoms was shown to perform consistently over time. It is hoped that further independent measurement of the absorbing material used will help explain the inter-phantom variability.

**Fig. 177 (abstract P34).**
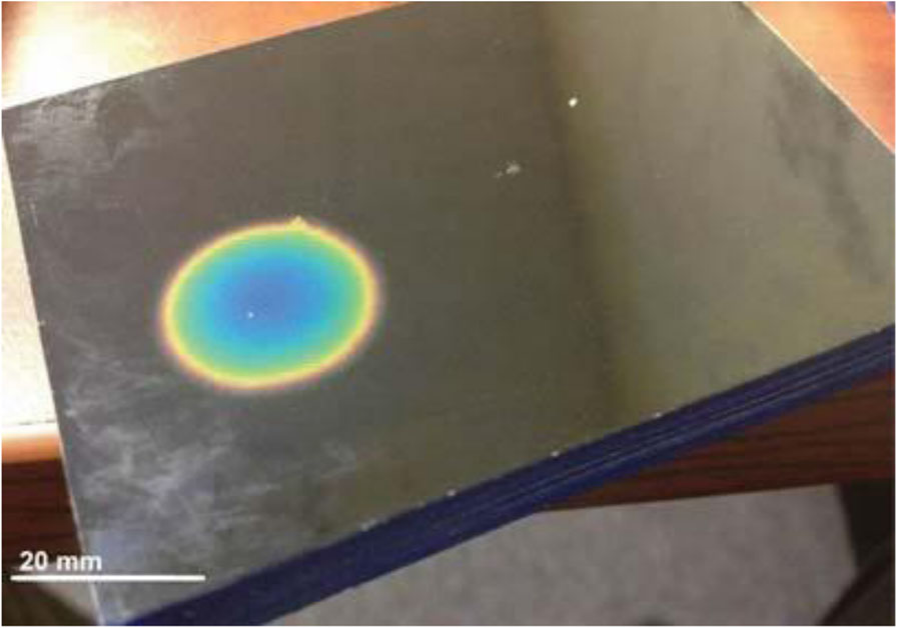
Thermochromic DQA phantom colour-change resulting from HIFU sonication

### P35 Cavitation localization and characterization using room acoustics analysis

#### Boaz Rafaely^1^, Alexander Volovick^2^, Javier Grinfeld^2^

##### ^1^Department of Electrical and Computer Engineering, Ben-Gurion University of the Negev, Beer-Sheva, Israel; ^2^InSighTec, Tirat Carmel, Israel

**Objectives**

Cavitation – bubbles oscillation and potential rupture is gaining a growing interest in therapeutic ultrasound, mainly for targeted drug delivery and blood brain barrier opening. The main challenges of cavitation based therapy are its localization and control. The localization problem is especially challenging for brain applications, there skull and inhomogeneous brainy tissue become both cloaking and unwanted cavitation generating factors. Recently, passive cavitation mapping was proposed (Miklós Gyöngy and Constantin-C. Coussios) and successfully integrated into clinical transcranial MRgFUS system (Arvanitis and McDannold). However the method requires large amount of passive receivers and in its current implementation allows only 2 dimensional monitoring.

Room acoustics analysis is a well-established field of research that allows localizing multiple speakers in highly reverberating environment (Nadiri and Rafaeli). In the presented work we suggest to utilize the methods studied in room acoustics analysis for cavitation localization.

**Methods**

ExAblate Neuro mid frequency (InSighTec) system was used for cavitation generation. Multiple 2 seconds continuous sonications were performed in degassed water (1 ppm level) using different acoustic powers at multiple locations. The acoustic data was recorded by 7 hydrophones and underwent localization using room acoustics analysis.

**Results**

**Fig. 178 (abstract P35).**
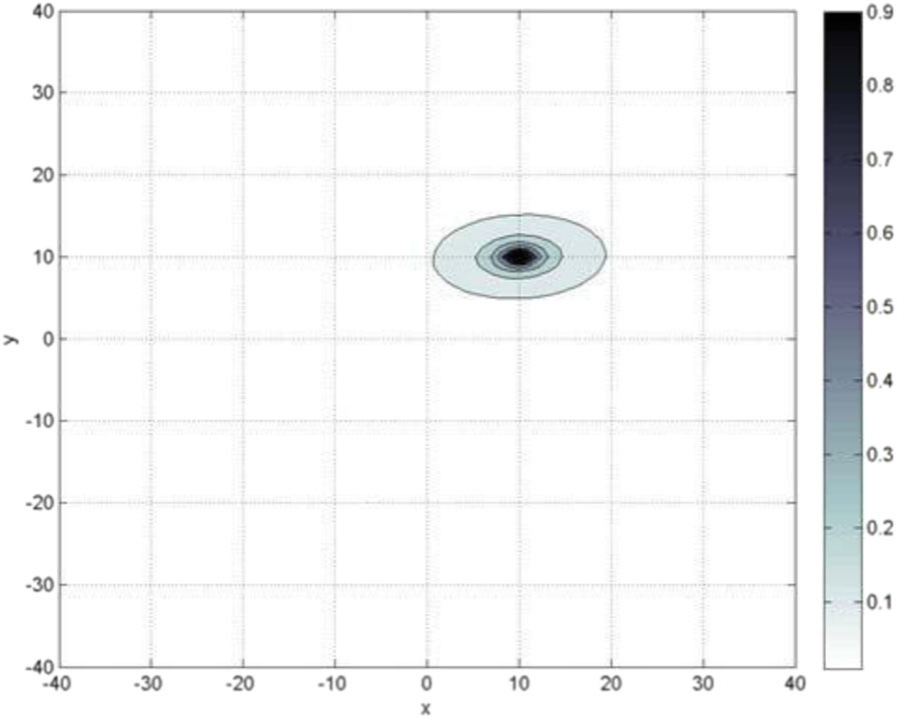
X-Y cost function for sonication at location of X=10, Y=10, Z=150

The cavitation was successfully localized with precision of up to 3 millimetres in the worst case using four receiving elements. X-Y plots of the cost function with the maximum at the identified position are presented for one of the sonications in Fig. 178. The localization results were sensitive to the choice of time segments inside each sonication changing during the sonication. This shows that the cavitation location in water can change during sonication.

**Conclusions**

The use of room acoustics analysis has proved to be accurate and consistent for cavitation localization. The use of significantly smaller amount of receivers for the localization is the biggest advantage relative to the existing methods. The cavitation cloud behaviour that changes the location during the sonication observation suggests that the method can be used both for localization and for characteristics.

### P36 Interferometric tracking of ultrasound-induced cell surface dynamics

#### Eitan Kimmel, Rasha Elaimy Debbiny

##### Biomedical Engineering, Technion – Israel Institute of Technology, Haifa, Israel

**Objectives**

The model of intra-membrane cavitation, which is denoted here as Bilayer Sonophore (BLS), based on ultrasound (US) induces pulsating gas pockets in the intra-membrane space between the two lipid leaflets. Resultantly, the acoustic energy is transformed into distortions and strains in the cellular structure. Moreover, the BLS may potentially explain both cavitational and non-cavitational US-induced bio-effects.

**Methods**

In order to validate the sonophore model and to study the response of cell membrane subjected to acoustic stimulation, a wide-field digital interferometry (WFDI) technique is used. WFDI is a label-free holographic technique that captures the complex wave front (phase and amplitude) of the sample field, containing the quantitative phase profile of the sample and gives a three-dimensional cell morphology map. A piezoelectric tube transducer of 1MHz frequency is used and acoustic pressure amplitudes in the range of 150kPa are quantified. The sonication time is ten minutes. Reconstructed images are obtained by an efficient digital process. We use the resulted images to quantify the cell morphological change. Three dimensional representations of the cell thickness are obtained. A quantitative analysis of the cross sectional area of the cell was performed.

**Results**

During the US transmission a morphological change of the examined endothelial cells (ECs) was observed. The measured cell thickness, based on phase profiles, was significantly increased in the nucleus region (9%± 2%). Consequently the cross-sectional area of cells became significantly smaller compared to their initial area.

**Conclusions**

Two mechanisms of force transmission between US and cells were considered as might be responsible for the morphological change: the tension force, formed in the membrane due to sonophore generation, and the radiation force. We predict that the geometrical change is attributed to the tension force formed in the membrane according to an estimated forces value, which might result in two elementary mechanisms: changing the membrane curvature due to tension, and changing the membrane curvature due to the detachment of focal adhesion (FA) contacts. However, we presume that most of the changes occur in the lower part interface, the surface between the cell and the plate, owing to the rearrangement (generation and detachment) of the FA points. Accordingly, the change in the cross-sectional area may be explained relying on the detachment of the FA contacts that led to higher values of cell thickness and to a more spherical shape.

### P37 Ultrasound backscatter as an indicator of intramembrane cavitation

#### Carmel Zeltser Dekel, Michael Assa, Eitan Kimmel

##### Biomedical Engineering, Technion, Haifa, Israel

**Objectives**

A possible mechanism of gas bubbles interactions with the acoustic field (cavitation) was explored using backscatter signal observations. The “Bilayer Sonophore” model (BLS model) suggests intramembrane cavitation as a unifying mechanism for US induced cavitation related bioeffects. It hypothesizes that the hydrophobic intramembrane space between the two bilayer leaflets of membranes is capable of inflating and deflating under an ultrasonic field thus creating cavitation nuclei of gas.

**Methods**

Using a focused US transducer (0.5MHz, λ=3mm) a sample (Bovine Aortic Endothelial Cells - BAEC or growth medium) was sonicated using a pulsatile regimen consisting of 15 cycles at a pulse repetition frequency (PRF) of 0.1 and 1 Hz and the backscatter signal was recorded as a function of time. As BAECs are significantly smaller than the US wavelength used, they constitute a very small scatterer. Combined with their fluidic consistency, the backscatter signal received from the BAEC is negligible and so it was hypothesized that any backscatter signal detected can only be the result of bubbles pulsation in the ultrasonic field, or more accurately, a bubble cluster. As more bubbles are formed, a higher backscatter signal which will be received and vice versa.

**Results**

The results showed a steady *increase* of the backscatter signal over time for BAEC in ~80% of cases (mean percent of change of 2.61% for 0.1Hz PRF and 4.69% for 1Hz PRF. The percent of change is calculated only for the cases showing increase of signal), with a *decrease* of the signal for control experiments using medium in 80% of cases for 0.1Hz PRF and 100% for 1Hz PRF (mean percent of change of −2.38% for 0.1Hz PRF and −11.72% for 1Hz PRF. The percent of change is calculated only for the cases showing decrease of signal). This was found to be statistically significant.

**Conclusions**

It is believed in this work that the decrease of the backscatter signal in the medium control case is the result of a mild degassing effect, in which free bubbles are expelled from the focal area due to the US pulse and thus less bubbles are available for pulsation, which resulted in backscatter signal decrease. Furthermore, it is believed that the increase of the backscatter signal in the BAEC case is possibly the result of sonophore formation in the intramembrane space thus creating stable encapsulated gas bubbles available for pulsation in the focal area, which resulted in the backscatter signal increase.

### P38 Bone phantom for evaluating mri-guided focused ultrasound thermal protocols

#### George Menikou^1^, Christakis Damianou^2^

##### ^1^City University, London, UK; ^2^Cyprus University of Technology, Limassol, Cyprus

**Objectives**

The goal of the proposed study was the development of a bone phantom that can be used for the evaluation of focused ultrasound protocols under magnetic resonance imaging (MRI) guidance.

**Methods**

High resolution CT images were used to segment femur bone. The segmented model was manufactured with (Acrylonitrile Butadiene Styrene) ABS plastic using a 3-D printer. The surrounding skeletal muscle tissue was mimicked using an agar-silica-evaporation milk gel.

**Results**

MR thermometry of focused ultrasound exposures were acquired using the bone phantom at a plane parallel and a plane perpendicular to the ultrasound beam.

**Conclusions**

Due to growing interest in using Focused ultrasound for pain palliation of bone cancer patients, the proposed phantom is considered as a very useful tool for evaluating ultrasonic exposures, thus minimizing the need for animal experiments.

### P39 Development of 3D collagen gel matrices suitable for cell exposure to high intensity focused ultrasound

#### Petros Mouratidis, Ian Rivens, Gail ter Haar

##### Radiotherapy and Imaging, The Institute of Cancer Research, Sutton, London, Surrey, UK

**Objectives**

The biological effects of High intensity focused ultrasound (HIFU) are often associated with the induction of high temperatures in target tissue. Traditional 2D cell culture models are not suitable for the investigation of HIFU’s thermal effects due to their lack of energy absorption. In contrast, 3D matrices could offer a tissue-mimicking biologically-compatible medium in which to demonstrate the thermal effects of HIFU. These gels can be designed to contain physiologically-relevant concentrations of extracellular matrix constituents including collagen. In this study colon cancer cells were cultured in 3D compressed collagen-based matrices. The effect of HIFU on cell viability was then investigated.

**Methods**

The human colon cancer cell line HT29 was used to form non-adherent cell spheroids, which enabled the cells to be contained in the narrow focus of the HIFU beam. These spheroids were embedded in 3D collagen gel matrices developed using Type I collagen from rat tail mixed with concentrated Minimum Essential Medium. The gel was allowed to set in a 2.5 cm wide cylindrical mold for 35 minutes. To increase collagen concentration a metallic plunger weighing 195 g compressed the gel twice. An ultrasound chamber was developed to allow exposure of the gel. The exposure system consisted of a 1.66 MHz HIFU transducer with a beam focal diameter of 1 mm and a focal length of 10 mm. Cavitation was detected using a passive cavitation detection sensor located in the central aperture of the HIFU transducer and connected to a data acquisition card. Cavitation data was processed using Matlab. The HIFU transducer was connected to an automated gantry which allowed computer controlled movement in 3D. The HIFU transducer targeted the wells of a purpose built, 3D printed 6-well acoustic plate. Wells were sealed at both ends with 19 mm thick Melinex films. The plate was held at the edge of a large water tank, and was connected to a fibre optic probe to record temperature. The compressed 3D collagen gel was placed in the middle of the well, fixed in place by two hydrogels on either side. A fibre optic probe was placed next to the cell spheroid. The gel was exposed to HIFU, transferred to a well of a 24-well plate and covered in medium. Cell viability was determined 1 and 4 days after exposure using the Cell Titer Luminescence assay.

**Results**

HT29 cells grew as spheroids in 2D medium and in 3D collagen matrices without a significant loss of viability for up to 7 days. Exposure of cells to heating these 3D matrices using a thermal cycler resulted in a reduction of cell viability that was dependent on applied thermal dose and on time elapsed after treatment. However those cells still viable after heat exposure did not retain their proliferative capacity. The 3D collagen gel matrices withstood HIFU exposures up to I_sp_ 1580 W/cm^2^. Pilot experiments showed that the viability of cells cultured in these matrices was reduced relative to sham exposed cells. Viability of cells in 2D medium was not significantly affected at these intensities. A full set of data will be presented.

**Conclusions**

A biologically and HIFU-compatible tissue-mimicking matrix has been created which allows investigation of the thermal effects of HIFU in vitro. Preliminary results suggest that HIFU exposure reduces the viability of cells cultured in this model.

### P40 VIM ablation using high intensity focused ultrasound. The CINAC experience

#### José A. Pineda-Pardo^1^, Marta Del Álamo de Pedro^1^, Raul Martinez^1^, Frida Hernandez^1^, Silvia Casas^2^, Carlos Oliver^2^, Patricia Pastor^2^, Lidia Vela^1^, Jose Obeso^1^

##### ^1^HM CINAC. Hospital Puerta del Sur, Móstoles, Spain; ^2^Hospital Puerta del Sur, Móstoles, Spain

**Objectives**

MRI-guided high-intensity focused ultrasound (HIFU) is a novel technology that is revolutionizing the concept of neurosurgery HIFU can potentially be used in many neurological and psychiatric disorders such as Parkinson’s disease, neuropathic pain or obsessive compulsive disorder among others. So far, one of the most successful applications arises for the treatment of Essential Tremor (ET). Several centres around the world have performed ablation of the ventral intermediate nucleus (VIM) of the thalamus for drug refractory ET. The Centre for Integrative Neuroscience (CINAC) in Hospital Puerta del Sur, Móstoles, has recently joined the HIFU community. This abstract summarizes our experience in the first five VIM ablation procedures.

**Methods**

Five patients, four with ET and one with tremor-dominant Parkinson’s disease (PD) were considered candidates for ablative thalamotomy after neurological evaluation. Pre-treatment evaluation included a general neurological exam and the clinical rating scale for tremor (CRST). MRI was performed in a 3T MR GE Discovery 750w. The imaging protocol consisted of 3D-T1w FSPGR, T2w TSE, T2 FLAIR and T2*. Pre-treatment radiological exam did not reveal any counter-indication for the procedure. Head CT was acquired in an Aquilion PRIME system (Toshiba) and Skull Density Ratios (SDR) were estimated. All patients presented a suitable skull density (see Table 6).

VIM ablations were performed with the clinical ExAblate 4000 system (InSighTec) fully compatible with the MR scanner. The ExAblate 4000 contains a hemispheric 1024 elements phased array transducer. The day of the procedure, the patients’ head was shaved and a stereotactic frame was attached using local anaesthesia. A flexible membrane was placed around the head, and was attached to the transducer, creating a close space, posteriorly filled with degassed water, which was circulating for cooling. 3D-T2w FIESTA images were acquired to define our target for ablation. VIM was localized in relation to anterior commissure and posterior commissure coordinates. Then the natural focus of the transducer was manually aligned to our target. Several low-power sonications (1500-2000J) were performed to confirm alignment between hot-spot and target. Once alignment was confirmed, energy administration was gradually raised in order to increase temperature in the target. Patients were awake during the procedure, and neurological examination was carried out after each sonication in order to evaluate tremor remission, and detect any possible side-effects, such as paraesthesias, ataxia or pyramidal features.

After procedure, patients were monitored at the ICU for 12h. The day after, post-treatment cranial MR was performed, using the same protocol as in the pre-treatment session. Clinical follow-up examination including the CRST was performed at 24 hours, one week, and one month.

**Results**

Tremor remitted in all five patients after the focused ultrasound procedure. One week later tremor evaluation showed a reduction in CRST of 63% for patient 1, 50% for patient 2, 76% for patient 4 and 53% for patient 5. Patient 3 was evaluated not on the whole CRST but just in the dominant hand, and showed full remission of tremor. One month after evaluation has been just carried in three of five patients, and CRST scores are stable with a maximum variation with respect to baseline of 6% (see Table 6).

**Fig. 179 (abstract P40).**
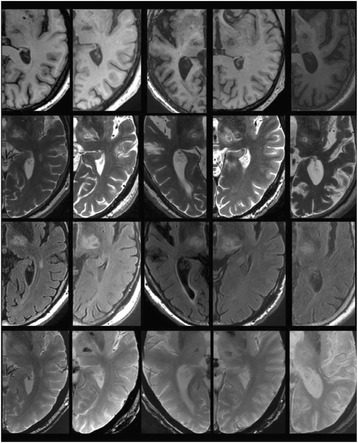
Axial view of MR images confirming the VIM ablation. Rows are sorted to MR contrasts, T1w, T2w, T2 FLAIR, and T2*. Columns are sorted according to patients’ ID

The procedure lasted in average 4.06 hours, 20.6 total sonications on average, and 2.8 average sonications exceeding an average on-target temperature of 55° (see Table 7). Side effects after the procedure included mild ataxia (patients 1 and 2), mild paresis (patient 3 and 5) and perioral paraesthesia (patient 4). Figure 179 shows different MR contrasts of the lesion 24 hours after the procedure in all the five patients.

**Conclusions**

Based in our experience, HIFU is a safe and effective treatment for tremor control in patients with ET. All patients showed marked improvement in their tremor that, while awaiting for a longer follow-up, was maintained for more than one month. Side-effects were transient and related to the ablative procedure.

**Table 6 (abstract P40).** Patients’ demography and clinical rating scale for tremor before and after the VIM ablative procedure

						CRST Baseline				CRST 1 Week				CRST 1 Month			
ID	Gender	Age	Diagnose	YoD	Laterality	A	B	C	T	A	B	C	T	A	B	C	T
01	F	74	ET	10	L	24	13	18	55	15	5	0	20	17	5	0	22
02	F	74	ET	15	L	9	11	14	34	5	7	5	17	5	7	3	15
03	F	77	PD	12	R	UPDRS Right Hand 10/12				UPDRS Right Hand 0/12							
04	M	64	ET	11	L	12	17	17	46	2	7	2	11	2	8	2	12
05	M	87	ET	25	L	28	36	27	91	18	18	6	42

**Table 7 (abstract P40).** Skull density ratios (SDR) and procedure related parameters, including procedure duration, number of sonications, total energy administered, and volume of the lesions in T1w and T2w MR contrasts

ID	SDR	Procedure duration	US elements	Total Sonic.	Sonic. (>45°)	Sonic. (>50°)	Sonic. (>55°)	Total Energy	T1 Lesion Volume (mm^3^)	T2 Lesion Volume (mm^3^)
01	0.41	3.5 h	945 (343 cm^2^)	16	13	8	4	66850 J	88.42	105.36
02	0.43	4.3 h	885 (326 cm^2^)	24	14	9	3	237200 J	262.36	332.31
0	0.34	3.6 h	875 (294 cm^2^)	16	14	8	3	96850 J	100.06	117.69
04	0.51	5.3 h	926 (354 cm^2^)	33	31	21	2	138250 J	225.99	274.07
05	0.34	3.6 h	916 (349 cm^2^)	14	12	7	2	101000 J	98.39	115.84

### P41 Tissue-mimicking phantom for HIFU lesion characterization by elastography

#### Paul Greillier, Ali Zorgani, Rémi Souchon, David Melodelima, Stefan Catheline, Cyril Lafon

##### LabTau - U1032, INSERM, LYON, France

**Objectives**

Polyacrylamide gel with bovine serum albumin (PAG-BSA) was proposed as a tissue-mimicking phantom for following thermal lesion during HIFU treatment (Lafon 2005). Indeed, the protein contained in the transparent phantom denatures and becomes opaque when heated. The aim of the present study was to assess if elastography can be used for monitoring HIFU lesions in PAG-BSA.

**Methods**

Guntur (2014) proposed a PAG-BSA phantom with glass beads (40-80μm) used as scatterers. BSA concentration was set to 18% (w/v) in order to improve thermal lesion contrast

Mechanical properties changes with thermal dose

Once polymerized, compression tests were performed on cylindrical samples of the gel (ø33x5mm) in order to measure the change of the Young modulus as a function of the thermal dose. Eighteen samples were heated in a 95°C water bath. Heating time ranged from 10s to 10 min.

Monitoring of HIFU lesion

A block of PAG-BSA (dimensions) was immersed in a water tank at a temperature of 37°C. Thermal lesions were generated with a 3.25 MHz focused transducer (50mm diameter and focal length). The focal spot was positioned 10 mm deep in the phantom. A focal intensity of 4000 W/cm^2^ (in water) was delivered for 15 s. The elasticity was measured with a 5MHz abdominal probe by two methods: passive elastography - PE - (Catheline 2013) performed with a Vantage 256 Verasonics system and transient elastography - SSI - (Bercoff 2004) with a clinical Aixplorer. B-mode, elastography and microscopy imaging were performed and compared just after sonications and 24h later.

**Results**

Compression test: Young modulus of the non-coagulated sample was 8.4 ± 1 kPa. Young modulus increased linearly with respect to heating time until it reached a steady-state of 24 kPa after 120s of heating.

Imaging: Shear waves propagated in the gel at a speed of 2.3 m/s according to PE and 1.8 m/s according to SSI. Just after sonication, lesions could be observed with all imaging methods. The lesions showed a tadpole shape in the sagittal plane of the block. Lesions were visible by SSI and PE. The surfaces of the lesion on the transverse focal plane were 21.7 mm^2^ and 23.9 mm^2^ for PE and SSI respectively. Shear waves propagation velocities in the lesion were 3.5 m/s with PE and 3.3 m/s with SSI. At 24h, the surface of the lesion decreased to 20.6 mm^2^ with PE. The shear wave’s speed was still 3.5 m/s with PE at 24h. One hour after HIFU exposure, microscopy evidenced, in the core of the lesions, the systematic presence of bubbles whose diameters ranged from 150 to 250 um. These dimensions are much larger than the glass beads. At 24 hours, no bubbles were detected except in the centre of the lesions.

**Conclusions**

Stiffness of the gel increased by a maximum factor of 2.8 with the coagulation of the BSA protein. Thermal lesions were visible in the PAG-BSA with both elastography methods tested in these experiments even when B-mode was no longer able to detect it. HIFU lesions increased the local stiffness of the PAG-BSA, at least by a factor of 2.2. The presence of bubbles did not disrupt the elastographic image.

*Acknowledgements*

*Work supported by the FUS Foundation.*

**Fig. 180 (abstract P41).**
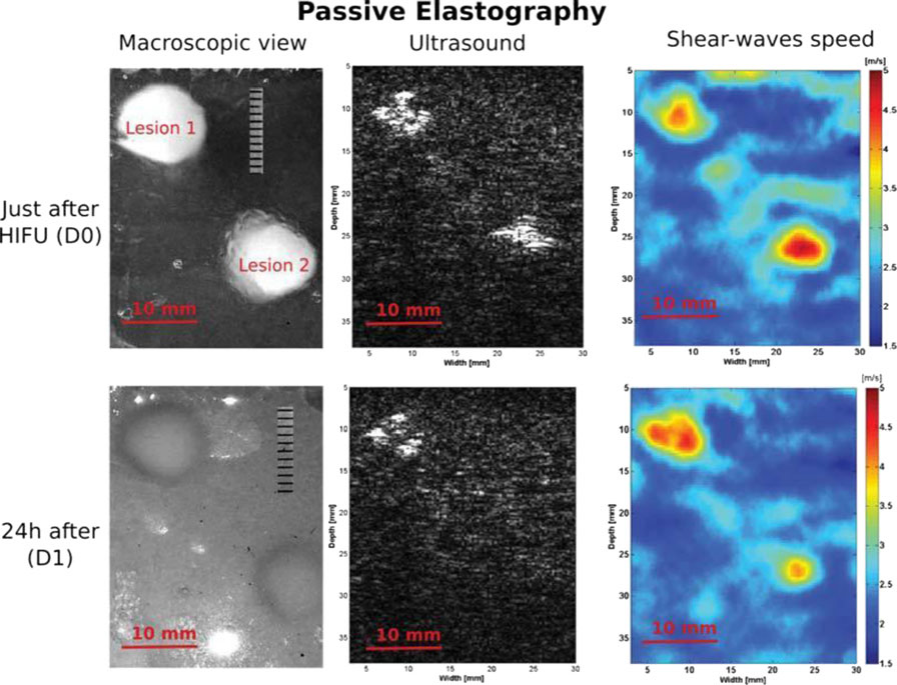
Comparison between macroscopic views, B-mode and PE maps. HIFU lesions were well-visible during 24h, even when B-mode did not show hyper-echoic signals

### P42 Robotic high-intensity focused ultrasound for the prostate cancer treatment of 1150 patients: 8 years single center experience

#### Vyacheslav Solovov, Michael O. Vozdvizhenskiy, Andrew E. Orlov

##### Samara Oncology Center, Samara, Russian Federation

**Objectives**

To report the results of the robotic high-intensity focused ultrasound (rHIFU) treatment for the prostate cancer and failure after external beam radiotherapy (EBRT) and radical prostatectomy (RPE), stratified by tumour recurrence risk according to D'Amico risk classification.

**Methods**

1150 patients were treated in our centre between Sep 2007 – November 2015: 339 with low risk progression, 355 with intermediate risk progression, 409 with high risk progression, 47 – after the EBRT and RPE failure. 925 patients underwent TURP+HIFU, 225 only HIFU (volume prostate < 40 cc). Mean follow-up is 62 months (range 3–98). The mean age of the whole group of patients was 69.2 (52–89) years.

**Results**

Median PSA level 12 months after rHIFU treatment were 0.03 (0.0-2.4) ng/ml – low risk group, 0.04 (0.1-4.2) ng/ml – intermediate risk group, high risk group - 0.5 (0–18.4) ng/ml, with failure after EBRT – 0.5 (0–3.2) ng/ml; 72 months after rHIFU treatment were 0.5 (0.0-3.6) ng/ml – low risk group, 1.04 (0.2-6.3) ng/ml – intermediate risk group, for high risk group – 3.2 (0–21.3) ng/ml, with failure after EBRT and RPE – 1.7 (0.0-9.8) ng/ml. Patients with low risk had 6.3% of progression, with intermediate risk had 8.2% of progression, with high risk PC – 37.4%, with failure after EBRT – 20.8%. Kaplan-Meir analyses of the total group indicated that the risk of progression after 8 year follow-up was 84.9% for all patients. Complications: incontinence I – 18.1%, incontinence II – 7.5%, stricture – 17.8%, fistula – 0.1%.

**Conclusions**

rHIFU ablation is a safe, minimally invasive treatment for localized and locally advanced prostate cancer, effective in 84.9% of cases with mild and transient side effects; rHIFU-therapy can also be successfully performed in patients with local recurrence after RPE and EBRT.

### P43 Ultrasound-triggered release of model drugs in hydrogels

#### Chueh-Hung Wu^1^, Ming-Kuan Sun^1, 3^, Tiffany T. Shih^2^, Wen-Shiang Chen^1, 3^

##### ^1^Physical Medicine & Rehabilitation, National Taiwan University Hospital, Taipei, Taiwan; ^2^Medical Imaging, National Taiwan University Hospital, Taipei, Taiwan; ^3^Biomedical Engineering and Nanomedicine, National Health Research Institute, Zhunan, Taiwan

**Objectives**

In biological systems, episodic release of bioactive compounds may elicit different effects from constant rate of release. For example, once-daily administration of pharmacological doses of parathyroid hormone has a bone anabolic effect, while continuous administration is detrimental for the skeleton due to stimulation of bone resorption. Conventional drug delivery systems allow approximate spatial localization of cues depending on polymer degradation and passive diffusion, but are unable to control release rates temporarily. In contrast, “on-demand” release systems which based on polymers or materials activated by external stimuli may provide such temporal flexibility and are beginning to be applied in the clinical setting. The current study aims at exploring the accelerating release rate of bovine serum albumin (BSA) embedded in the N-isopropylacrylamide (NIPAM) -based hydrogel in response to temperature rise provided by ultrasound (US) exposure with energy in therapeutic ranges.

**Methods**

The NIPAM-based reusable hydrogel phantoms were fabricated by crosslinking copolymerization of NIAPM and N,N'-methylenebisacrylamide (MBAm) with the addition of acrylic acid (AAc) to adjust the cloud point so that it fell in the temperature range of biological significance (37oC in this study). Hydrogels, before set, were mixed with BSA and added into a 6-well culture plate before heating by either water bath or US (1 MHz, 30% duty cycle, 3 W/cm2) for a certain period of time. The ability of BSA release from hydrogels was tested in various conditions including (1) different NIAPM (1, 2 and 3 g) and MBAm (0.02 to 0.10 g) compositions, (2) different target temperature from 25 to 45oC, and (3) water bath heating or ultrasound exposure (both thermal and mechanic effects). Moreover, the microstructure of the hydrogel below and above the cloud point was examined by electronic microscopy.

**Results**

**Fig. 181 (abstract P43).**
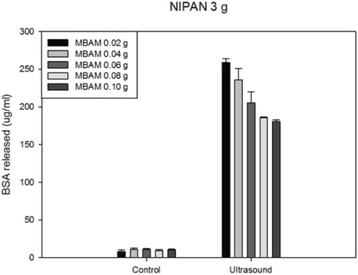
BSA release at different MBAm amount in water bath or after US exposure

**Fig. 182 (abstract P43).**
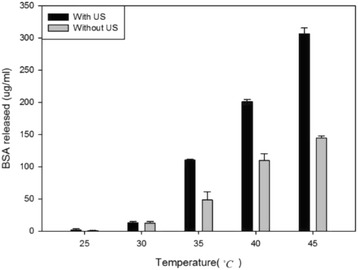
BSA release at different temperature in water bath or after US exposure

With the same MBAm amount, release of BSA reduced when NIPAM increased. With the same NIAPM amount, release of BSA increased with the increase of MBAm (Fig. 181). Both water bath heating and US exposure induced BSA release and is temperature dependent, but significantly more release was induced by US exposure (Fig. 182). At temperature below the cloud point, the hydrogel (NIPAM/MBAm=1 g/ 0.02 g) is transparent in colour and shows dense microstructure with small pores. When heated above the cloud point, the whitish hydrogel shows pores of increased sizes.

**Conclusions**

In this study, an ultrasound and temperature dual-responsive hydrogel suitable for controlled release is described. In certain formula of the NIPAM-based hydrogel, the ratio of BSA release between ultrasound-triggered release and release from diffusion could reach 32 folds. In another formula, transparent hydrogel became whitish when release from the hydrogel increased. By simply adjusting contents of NIPAM and MBAm, the ratio of BSA release between ultrasound-triggered release and release from diffusion can be adjusted according to clinical needs.

Ultrasound-triggered release has only been reported in few studies. By combing microbubbles, liposomes and the hydrogel, ultrasound-trigger release was demonstrated in this complicated system. The system would be structurally destructed by ultrasound exposure. Using a reversibly cross-linked alginate-based hydrogel, ultrasound induced a 10-fold increase in drug release. (PNAS 2014) In another study, using gold nanoparticles coupled with poly(ethylene glycol) incorporated in the alginate-based hydrogel, ultrasound stimulated the release rate by 110 folds. In this study, the ultrasound and temperature dual-responsive NIPAM-based hydrogel directly contained BSA without incorporating liposomes and nanoparticles and reached an increasing release rate by about 32 folds. Furthermore, the ratio of BSA release between ultrasound-triggered release and release from diffusion ranged from 1.5 folds to 32 folds by modifying contents of NIPAM and MBAm.

The mechanism of increased release responsive to temperature may be, at least partly, associated alteration of the hydrogel microstructure. Under SEM, sizes of the pores in the hydrogel increased when temperature increased to above the LCST. In such circumstance, the hydrogel became whitish, possibly due to light scattering; increased of BSA also increased possibly due to enlarged pores. However in the current study, even at the same temperature, release rate was much higher using ultrasound stimulation than using water bath. Such difference may be related to non-thermal effects of ultrasound, such as streaming or cavitation.

One possible clinical application of this dual-responsive hydrogel is that, after implantation into the body, there will be a two-step controlled release. The first step is achieved by heating the hydrogel. If a higher release is needed, applying ultrasound stimulation will achieve the second-step release. Another possible clinical application of the discolouring hydrogel associated with increased release is for designing a wound dressing material. One of the problems in treating poorly-healed wound is to determine the timing of changing wound dressing. Using this discolouring hydrogel as a medication-containing dressing, the healthcare-provider can change the dressing when it becomes whitish, because at this timing most medication has been released.

### P44 Enhancement of fluorescent probes penetration into tumours in vivo using unseeded inertial cavitation

#### Fabrice Prieur^1,2^, Arnaud Pillon^3^, Jean-Louis Mestas^2,4^, Valerie Cartron^3^, Patrick Cebe^3^, Nathalie Chansard^3^, Maxime Lafond^2^, Cyril Lafon^2,4^

##### ^1^University of Oslo, Oslo, Norway; ^2^U1032, LabTau, INSERM, Lyon, France; ^3^Centre de Recherche en Oncologie Experimentale, Institut de Recherche Pierre Fabre, Toulouse, France; ^4^Caviskills SAS, Vaulx-en-Velin, France

**Objectives**

In the context of ultrasound enhanced drug delivery, our aim was to assess the impact of sonication and inertial cavitation on the penetration of fluorescent probes into tumour cells. We also wanted to estimate the impact of parameters such as the peak negative pressure generated at focus, the timing of sonication, and its duration.

**Methods**

Female nude mice (Charles River, Saint-Germain-sur-l'Arbresle, France) were used for a subcutaneous implantation of WM-266-4 human metastatic melanoma xenografts. Tumours were exposed to ultrasound about 20 days after cell implantation when solid tumours had reached a volume about 500–1000 mm3. Sonication was applied either 30 minutes after (protocol 1) or 10 minutes before (protocol 2) the injection of 100 μL of fluorescent probe AngioSense 680 EX (PerkinElmer, Waltham, MA) at a concentration of 4 nMol/100 μL.

**Fig. 183 (abstract P44).**
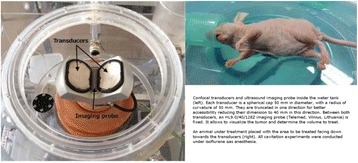
See text for description

The ultrasound system used for sonicating the animals and generating unseeded inertial cavitation inside the tumour consists of a two confocal transducers transmitting 40-μs long sinusoidal pulses at 1 MHz centre frequency every 4 ms. The transducer assembly is immersed in a tank filled with degassed water and can be positioned with motors ensuring translation in the three directions (see Fig. 183). This allows sonication of a predefined volume that includes the tumour using raster scans in stacked horizontal planes.

To estimate the penetration of fluorescent probes inside the tumour cells the animals were sacrificed and the tumours harvested. We used a cytometric analysis to quantify the fluorescence intensity of the non-necrotic cells due to the presence of fluorescent probes and compared the sonicated groups to the reference groups.

In the first series of tests, peak negative pressure was varied between 16 MPa, 19 MPa, and 21 MPa rarefactional pressures. In the second series of tests, protocols 1 and 2 were compared to assess if penetration was greater when probes were injected before or after sonication. In the last series of tests, the speed of the raster scan was increased from 1 mm/s to 2 mm/s and 4 mm/s to estimate the impact in probe penetration when reducing the sonication duration.

**Results**

**Fig. 184 (abstract P44).**
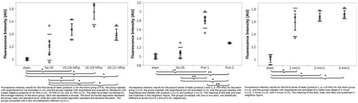
See text for description

The first series of tests showed that ultrasound generated inertial cavitation had a significant effect on the penetration of probes into tumour cells with a maximum improvement of 47%. The peak negative pressure level that gave the largest improvement was 19 MPa (see Fig. 184). At this pressure, macroscopic observation showed minor skin damages such as point like petechiae that disappeared a few days post-treatment.

In the second series of tests, both protocols showed a significant difference in probe penetration compared to the control group but protocol 1 where sonication was applied in the presence of the probes showed a much larger improvement than protocol 2 (68% vs. 20%, see Fig. 184).

The last series of tests showed that increasing the raster scan speed and decreasing the duration of sonication by a factor 4 did not have a significant impact (see Fig. 184). This means that the treatment time could be significantly reduced without reducing its efficiency.

**Conclusions**

We have presented an ultrasound device that generates a significant improvement in the penetration of fluorescent probes into in vivo tumour cells. The hypothesized mechanism at play is ultrasound-induced inertial cavitation. Our parametric study showed that ultrasound exposure improved the uptake of fluorescent probes. The optimal pressure level generated an improvement in probe penetration up to 68% with only minor damages on the skin. For best efficiency, the ultrasound should be applied in presence of the probes. The raster scan speed could be set up to 4 mm/s without adverse consequences.

### P45 Real-time monitoring and control of cavitation activity for enhancing ultrasound transfection and bubble-cell interactions

#### Claude Inserra^1^, Pauline Muleki Seya^2^, Wen-Shiang Chen^3^, Jean-Christophe Bera^1^

##### ^1^LabTAU Inserm U1032, Lyon, France; ^2^Laboratoire de Mécanique et d’Acoustique CNRS - UPR 7051, Marseille, France; ^3^National Taiwan University Hospital, Taipei, Taiwan

**Objectives**

Sonoporation process, at the origin of ultrasound cell transfection, is ruled by the interaction between cells and cavitating bubbles. Due to the stochastic behaviour of acoustic cavitation, there exists a need in ensuring a stable and reproducible state of cavitation within a medium during cell transfection to enhance transfection efficiency and to control mortality. In this study is described how stochastic the cavitation activity could be by describing a classical nonlinear effect (hysteresis effect), before the characterization of a controlled-cavitation device dedicated to the real-time monitoring, control and stabilization of inertial cavitation activity during cell sonication. The induced ultrasound strategy for sonication is discussed through the quantification of sonoporation efficiency for suspended cells or adherent cell monolayer, as well as the need of employing an initial distribution of gas nuclei thanks to ultrasound contrast agents in order to initiate cavitation process.

**Methods**

A specific ultrasound sonoporation chamber has been designed by mounting ultrasound transducers (frequency around 450 kHz) on a culture well located within a microscopic stage allowing real-time visualization of bubble activity atop an adherent cell monolayer during a sonication experiment. A needle hydrophone allows listening the cavitation broadband noise in order to quantify inertial cavitation activity. The hydrophone signal is then digitized and sent to a FPGA (Field Programmable Gate Array) that synthetizes the output signal feeding the ultrasound transducers, and modulate the applied acoustic intensity to the transducer to reach and maintain a fixed inertial cavitation activity during the experiment. For cell sonoporation experiments, HT29 adherent cells (human colorectal adenocarcinoma cell lines) were plated in the well at 2.10^6 cells/well, with 2 mL of RPMI 1640 completed media. Sonoporation experiments consisted in the internalization of siRNA coupled to the fluorochrome AlexaFluor-488 before analysis with a flow cytometer.

**Results**

First, when applying successive ultrasound shots for increasing and decreasing fixed acoustic intensity, it is shown that inertial cavitation activity exhibits hysteretical behaviour related to the fluctuation of the bubble size distribution. Besides its fundamental interest in nonlinear physics, this hysteretic effect finds applications in the design of appropriate control of the cavitation activity by taking advantage of the jump of the phenomenon from a metastable state to another. This strategy is applied thanks to FPGA programming allowing a permanent modulation of the applied signal amplitude to the transducer in an ultrafast regulation loop (smaller than 300μs). When using such real-time control, the inertial cavitation activity reveals increased time stability and reproducibility, with the possibility of controlling cavitation activity in pulsed wave from a pulse to another, and even within a single pulse. When studying cell transfection with or without regulation process, it is shown that transfection rate is more reproducible when using the cavitation-controlled device, even without attaining much higher transfection rate. This increase in reproducibility has been observed for both suspended and adherent cells experiments. Because the real-time cavitation control demonstrated its potential for maintaining a stable cavitation state during an ultrasound shot, the question arises of the interest of using ultrasound contrast agents as cavitation nuclei. It is shown in-vitro that, when using the cavitation-controlled device superimposed to the injection of ultrasound contrast agents, the reproducibility of inertial cavitation activity is even enhanced. Interestingly, when ultrasound contrast agents quickly collapse during sonication, the regulated-cavitation device can counterbalance their destruction to sustain a stable cavitation state. The interest of combining UCAs and cavitation control is then demonstrated for in-vitro cell transfection.

**Conclusions**

Lowering applied acoustic intensities while increasing the induced biological effects is of great importance for the growth of ultrasound-based drug delivery applications without increase of cell mortality. Such objective can be attained by the real-time monitoring and control of cavitation state during medium sonication, in-vitro and even in-vivo. In this study, the interest of using a cavitation-controlled sonoporation device is demonstrated by enhancing cell transfection reproducibility, as well as the possibility of counterbalancing the use of ultrasound contrast agents *in vitro*.

### P46 Theranostic nanocapsules for 19f-mri and ultrasound-triggered drug release

#### Tanguy Boissenot^1^, Benoit Larrat^2^, Elias Fattal^1^, Alexandre Bordat^1^, Helene Chacun^1^, Claire Guetin^1^, Nicolas Tsapis^1^

##### ^1^Institut Galien Paris-Sud, CNRS, Univ. Paris-Sud, Université Paris Saclay, Châtenay-Malabry, France; ^2^CEA, Neurospin, Saclay, France

**Objectives**

The combination of imaging and treatment is the prerogative of a new scientific concept called theranostic (contraction of therapeutic and diagnostic). While conventional imaging provides anatomical visualization of organs and tissues for the purpose of detecting and tracking the evolution of the disease, the future of imaging is expected to predict the efficacy of treatment and monitor the evolution of its activities throughout the disease for early detection of phenomena of resistance and to consider a rapid change from the first signs of relapse. Moreover it will have to deliver drugs only to the pathological site allowing increasing efficacy while reducing side effects. Progress of new therapeutic will therefore be done in conjunction with new imaging methods more sensitive, more specific, less invasive and less expensive, to allow an early and reliable diagnosis of the response related to the drug's action and active targeting with localized, effective, and safe new drug delivery platform. Micro-nanoparticulate vectors are particularly advantageous from this point of view as they allow to encapsulate in a single particle the contrast agent and the drug and hence to follow the action thereof in real time while limiting the number injections.

Our work will be to develop novel nanoparticle systems with both contrast agents for medical imaging properties and active drug delivery by high intensity focus ultrasound. We seek to design a theranostic agent for targeting tumours and with the ability of being observed by fluorine magnetic resonance imaging (19F MRI). This intravenously injectable agent with encapsulated drugs, should allow to visualize tumours. Then focused ultrasound will induce release of the encapsulated drug in order to achieve a local chemotherapy.

**Methods**

We have developed theranostic nanocapsules combining a diagnostic moiety to improve tumour detection and a therapeutic moiety to treat them. These nanocapsules are composed of a polymer shell of PLGA-PEG and a core of a perfluorocarbon namely perfluorooctyl bromide (PFOB) in which a cytotoxic drug (paclitaxel) is encapsulated. The goal is to use these capsules to select patients exhibiting the enhanced permeation and retention effect for further treatments with nanomedicines. In our case, PFOB has the interesting properties to respond to 19F MRI to allow early detection of the tumour, diagnosis of accumulation of EPR effect and follow up of the response to treatment. This liquid perfluocarbon is also responsive to ultrasound allowing an active targeting by high intensity focused ultrasound to actively deliver cytotoxic drug to tumours. Paclitaxel, an antimitotic drug, was encapsulated in an attempt to reduce side-effects associated with excipients such as Cremophor® used in the commercial formulation (Taxol®).

**Results**

We optimized the encapsulation of paclitaxel into nanocapsules by varying formulation parameters, focusing in particular in preventing or limiting paclitaxel recrystallization and nanocapsule aggregation. The optimized formulation was tested in vitro on CT-26 colon cancer cells and showed similar cytotoxicity as compared with Taxol®. Paclitaxel pharmacokinetics and biodistribution were evaluated in nude mice bearing CT-26 xenografted tumours comparing nanocapsules with Taxol®. For nanocapsules, pharmacokinetic parameters are improved leading to a longer circulation and resulting in an enhanced accumulation in tumours, as confirmed by 19F-MRI. In term of efficacy, this enhanced passive targeting allows a slower tumour growth in animals treated with paclitaxel-loaded nanocapsules compared to PBS and Taxol®. Ultrasounds were also used to further improve tumour targeting. In collaboration with Benoit Larrat’s team at CEA, we studied the influence of ultrasound on our nanoparticles to determine the most suitable ultrasonic sequences for our system. We showed that when applying a safe ultrasound sequence inducing mild hyperthermia, tumour growth was slower on our tumour model. In vitro studies showed that this decreased growth is due to mild hyperthermia favouring tumour perfusion and vascular leakage leading in an enhance accumulation of drugs inside the tumour.

**Conclusions**

Finally we showed that it is possible to combine within the same nanoparticles the diagnostic properties of a 19F MRI contrast agent and an active drug targeting mediated by High intensity focus ultrasound. The theranostic agent allows a better diagnosis and a higher anti-tumoural efficacy compared to available therapies.

**Fig. 185 (abstract P46).**
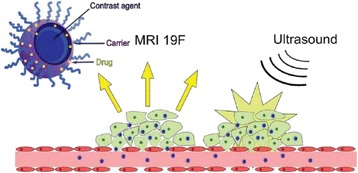
See text for description

### P47 Development of a new freeze-dried nanobubble for US theranostics

#### Kazuo Maruyama, Johan Unga, Ryo Suzuki

##### Department of DDS Research, Teikyo University, Tokyo, Japan

**Objectives**

To develop a novel bubble formulation for ultrasound (US) imaging and therapy with small particle size and a good stability and test the formulation as US contrast agent and for gene delivery *in vitro* and *in vivo*.

**Methods**

Lipid-stabilized bubbles were prepared by homogenization of lipid dispersion in the presence of perfluoropropane gas. Different phospholipid compositions were tested and evaluated. After bubble formation the bubbles were freeze-dried so that a dry cake containing bubbles was formed. After reconstitution of the samples they were analysed for size, gas content and US signal intensity and longevity. The bubbles were also evaluated as US contrast agents *in vivo*, and for US activated gene delivery of luciferase encoding pDNA in vitro on cell culture and *in vivo* in mice.

**Results**

Bubbles were in the size range 500–800 nm and could be preserved by freeze-drying and re-constituted by simple addition of water to the dry sample. Changes in the lipid composition had a big impact on the properties of the bubbles produced. A mixture of three different lipids in the stabilizing layer resulted in the most stable bubbles. *In vivo* imaging of B16BL6 tumours in mice, using the most stable bubbles showed half-lives substantially longer than for the commercial bubble Sonazoid. Also, the bubbles were well suited for visualization of tumour neovasculature. Bubbles together with pDNA and US exposure increased the luciferase activity by about 300 times *in vitro* and 2000 times *in vivo*, respectively, compared to only pDNA+US.

**Conclusions**

We believe this new formulation shows great promise for both diagnostic and therapeutic applications thanks to its good stability, relatively small bubble size and the simplicity of handling.

*Acknowledgements*

*This work was supported by MEXT-Supported Program for the Strategic Research Foundation at Private.*

### P48 Ultrasonic potentiation of doxorubicin-induced cell death in 4K1 cells *in vitro*

#### Cécile Fant, Maxime Lafond, Bernadette Rogez, Jacqueline Ngo, Cyril Lafon, Jean-Louis Mestas

##### LabTAU, INSERM U1032, Lyon, France

**Objectives**

Ultrasonic cavitation has been shown to be a promising strategy to potentiate chemotherapy drugs. In the case of doxorubicin (DOX), a potent drug routinely used in clinic, it can address to a twofold problem: severe side effects and acquired drug resistance. Under certain settings, the onset of ultrasonic cavitation entails profound physical and chemical changes on cell structure. These alterations can be exploited to improve the delivery of chemotherapy drugs, enhance their effects and overcome drug resistance in tumour cells. The aim of this study was first to evaluate the potentiation of DOX by stable cavitation in murine 4T1 mammary tumour cells in vitro. The mechanism of potentiation was also investigated.

**Methods**

An ultrasonic setup based on confocal ultrasound (US) beams separated by an angle of 90° was used. It operated at a frequency of 1.1 MHz The apparatus was able to generate stable cavitation (generation and oscillation of bubble without collapse) without adding cavitation nuclei such as ultrasound contrast agents. High-pressure pulses of 6600 cycles (6.7 MPa peak negative pressure – PNP at a pulse repetition frequency of 25 Hz) produced a cavitation cloud in the sample while lower pressure pulses (2.95 MPa PNP, PRF 25Hz) maintained a stable cavitation activity. Monitoring of cavitation activity based on the measurement of the subharmonic emergence at 550 kHz with an integrated PVDF hydrophone permitted to switch between the two pressure regimens. Four groups were used: control, DOX alone, US alone and DOX/US combination. The cell growth index (number of viable cells relatively to the number of viable cells at 1h post-US) is measured at 72h post-US. Flow cytometry measurements at 1h post-US were also performed to assess the DOX internalization. In a second time, the same experiment was reproduced adding radical scavenger (histidine and mannitol) to evaluate the role of radical oxygen species (ROS) in the potentiation.

**Results**

Measurements showed that stable cavitation was obtained with a high reproducibility, being 12.5±2.4 dB. At 72h, the cell growth indexes were 6.4±2.2 and 5.4±1.3 for US group and controls respectively and not statistically different. The DOX dosage chosen for this study lead to a cell growth index of 0.54±0.17 while DOX+US permitted to reduce the index to 0.31±0.092. No increase of DOX uptake could be observed at 1h post-US, discarding the hypothesis of sonoporation. Moreover, radical scavengers did not prevent the reduction of proliferation, excluding the role of the radical oxygen species produced by cavitation in the observed synergetic effect.

**Conclusions**

Unseeded stable cavitation was initiated and maintained using confocal ultrasound, an acoustic strategy based on two pressure levels and a passive cavitation detector for monitoring. The created cavitation was shown to act synergistically with doxorubicin in order to reduce the tumour cell proliferation. However neither sonoporation nor an action of free radicals could be evidenced as an underlying mechanism.

### P49 Effect of acoustic radiation force on the distribution of nanoparticles in solid tumours

#### Mercy Afadzi^1^, Ola Finneng Myhre^2^, Siri Vea^2^, Astrid Bjørkøy^1^, Petros Tesfamichael Yemane ^1^, Annemieke van Wamel^1^, Sigrid Berg^3, 2^, Rune Hansen^3, 2^, Bjørn Angelsen^2^, Catharina Davies^1^

##### ^1^Physics, Norwegian Univeristy of Science and Techonlogy, Trondhiem, Norway; ^2^Circulation and Medical Imaging, Norwegian Univeristy of Science and Techonlogy, Trondhiem, Norway; ^3^SINTEF Technology and Society, Trondhiem, Norway

**Objectives**

Encapsulating cytotoxic drugs in a particulate carrier such as liposomes, micelle or other nanoparticles (NPs) may reduce toxicity to normal tissues by delivering NPs selectively to tumour tissues. However, due to the heterogeneous fenestration of the tumour blood vessel and the poor penetration through the extracellular matrix (ECM), large areas of the tumour are not reached by the drug/NPs. To overcome these challenges in drug delivery, development of new and effective treatments is of great need. In recent years, there have been considerable efforts to develop therapeutic use of focused ultrasound (FUS) and it has shown significant potential in cancer therapy. Ultrasound –mediated mechanism such as cavitation and acoustic radiation force (ARF, transfer of momentum from US beam to the attenuating media) may enhance both extravasation of NPs across the capillaries and penetration of NPs through the ECM. The study aims to investigate the effect of ARF on the transport of NPs across the capillary wall and through the ECM.

**Methods**

Silica NP (70 nm) with half-life of 2h was injected intravenously into mice bearing subcutaneous prostate xenografts. The tumours were exposed to various FUS exposure parameters (5 or 10MHz frequency, 0.5 or 1μs pulse length and 1 5 66 kHz pulse repetition frequency) immediately or 4h after the injection of the NP Using a stepping robot the tumour was scanned (3×3 points) in the lateral plane for 1h and the distance between each scanned point corresponds to the focal width of the US probe. Total scanned areas were 1.8 × 1.8 mm and 0.9 ×0.9 mm for the 5 MHz and 1 0MHz respectively. Fluorescent labelled lectin was injected immediately after US treatment and allowed to circulate for 5mins to stain the blood vessels. Tumours were surgically removed and frozen sections were analysed by confocal laser scanning microscope. Confocal microscopy images were then analysed quantitatively using ImageJ and Matlab. The experimental work was supported by simulation of ARF on tissues using the exposure parameters of the experimental work.

**Results**

Preliminary results indicate an enhanced extravasation and improved microdistribution of NPs in the US exposed animals compared with non-exposed animals. Thus, tumours exposed to US showed increase mean distances between NPs and microvessels than non-exposed tumours.

**Conclusions**

The results demonstrate the potential of FUS to improve cancer therapy, and that ARF can be an important mechanism for improved delivery of NPs.

